# Blood metabolites as mediators in erectile dysfunction: insights from a multi-center proteomics and genetic study

**DOI:** 10.3389/fphar.2025.1568780

**Published:** 2025-06-02

**Authors:** Junhao Chen, Junxian Zhao, Zhi Zhang, Xingcheng Zhu, Jieming Zuo, Zuqing Nie, Yuanzhi Fu, Haifeng Wang, Mengjun Tang, Shi Fu

**Affiliations:** ^1^ Department of Urology, The Second Affiliated Hospital of Kunming Medical University, Kunming, Yunnan, China; ^2^ Department of Urology, 920th Hospital of Joint Logistics Support Force of Chinese People’s Liberation Army, Kunming, Yunnan, China; ^3^ Department of Urology, The Central Hospital of Yongzhou, Yongzhou, Hunan Province, China; ^4^ Department of Clinical Laboratory, The Second People’s Hospital of Qujing City, Qujing, Yunnan, China; ^5^ Department of central lab, The Second Affiliated Hospital of Kunming Medical University, Kunming, Yunnan, China; ^6^ Kunming University of Science and Technology, Kunming, China; ^7^ Orthopedic Department, The 967th Hospital of Joint Logistic Support Force of PLA, Dalian, China

**Keywords:** mendelian randomization, proteomics, erectile dysfunction, blood metabolites, precision therapy

## Abstract

**Objective:**

This study aims to identify circulating proteins causally associated with erectile dysfunction (ED) using Mendelian randomization (MR) analysis.

**Methods:**

We utilized two of the largest multi-center proteomics databases as exposures and the FinnGen database as the outcome source. A large-scale two-sample MR analysis, including coloc colocalization analysis and SMR (Summary data-based Mendelian Randomization) analysis, was conducted to evaluate the reliability of proteomic effects on ED outcomes. Additionally, MR mediation analysis involving 1,400 blood metabolites was performed to investigate how these proteins mediate the effect of blood metabolites on ED. Finally, protein-protein interaction analysis, pathway enrichment analysis, druggability assessments, and molecular docking were employed to further elucidate the mechanisms of ED and identify potential therapeutic targets.

**Results:**

Eight circulating proteins (AMN, ESM1, KIR2DL2, PIGR, SPINT1, SPP1, TNFRSF6B, TMEM9) were identified as causally associated with ED based on two-sample MR and coloc colocalization criteria. Among these, five proteins (AMN, ESM1, KIR2DL2, PIGR, TNFRSF6B) satisfied SMR validation, while SPINT1, TMEM9, and SPP1 were excluded. Several of these proteins were found to mediate the relationship between metabolites and ED. These proteins are recognized as either druggable targets or existing drug targets, with molecular docking results demonstrating favorable interactions with various drug candidates.

**Conclusion:**

Using MR analysis, we identified five proteins associated with ED, clarified protein-mediated mechanisms, and proposed promising therapeutic targets for ED.

## 1 Introduction

ED refers to the inability to achieve or maintain a sufficient penile erection for satisfactory sexual performance. As one of the most prevalent male sexual disorders, ED significantly impacts the quality of life and mental health of affected individuals (McMahon, 2019). The prevalence of ED increases substantially with age. For instance, an Indonesian study reported an ED prevalence of 6.5% among men aged 20–29 years, rising to 88.0% in men aged 60 years or older (Birowo et al., 2019). Similarly, a Spanish study found the prevalence of ED to be 18.9% in men over 40 years and 48.6% in men over 70 years (Ruiz-Garcia et al., 2019). Although not life-threatening, ED can severely compromise physical and mental wellbeing, reduce overall quality of life, and disrupt personal, family, and professional domains.

ED is often recognized as a vascular and inflammatory disease associated with various metabolic and physiological processes. Plasma proteins play an essential physiological role in the human body, as they are secreted and released into circulation to perform critical functions, including intercellular communication. These proteins are not only involved in blood transport but also play vital roles in immune responses, inflammation regulation, and cellular signaling (Zhong et al., 2023; Jamaluddin et al., 2019). Recent studies have gradually uncovered their potential roles in ED. First, in vascular function regulation: plasma proteins such as albumin and globulins regulate blood viscosity and vascular permeability, which are crucial for maintaining normal erectile function (Yao et al., 2020), as the erection process depends on the blood filling of the penile corpus cavernosum. Second, regarding inflammatory responses: certain plasma proteins, such as C-reactive protein (CRP), serve as markers of inflammation. Elevated CRP levels are associated with cardiovascular diseases, which are known risk factors for ED. Third, in immune modulation: immunoglobulins play a key role in immune responses. Immune dysregulation may increase chronic inflammatory reactions, further impairing vascular and neurological functions, thereby increasing the risk of ED (Chen et al., 2014). Lastly, in nutritional status and metabolic function: plasma protein levels reflect an individual’s nutritional and metabolic state. However, the proteome represents a vast and complex domain. Due to high costs, lengthy timelines, and the difficulty of eliminating confounding factors, traditional randomized controlled trials (RCTs) are challenging to conduct MR, a novel genetic epidemiological method, leverages genetic instrumental variables to minimize confounding and identifies causal relationships between circulating proteins and ED by utilizing pQTLs as genetic instruments.

## 2 Methods

### 2.1 Data sources

**FIGURE 1:**
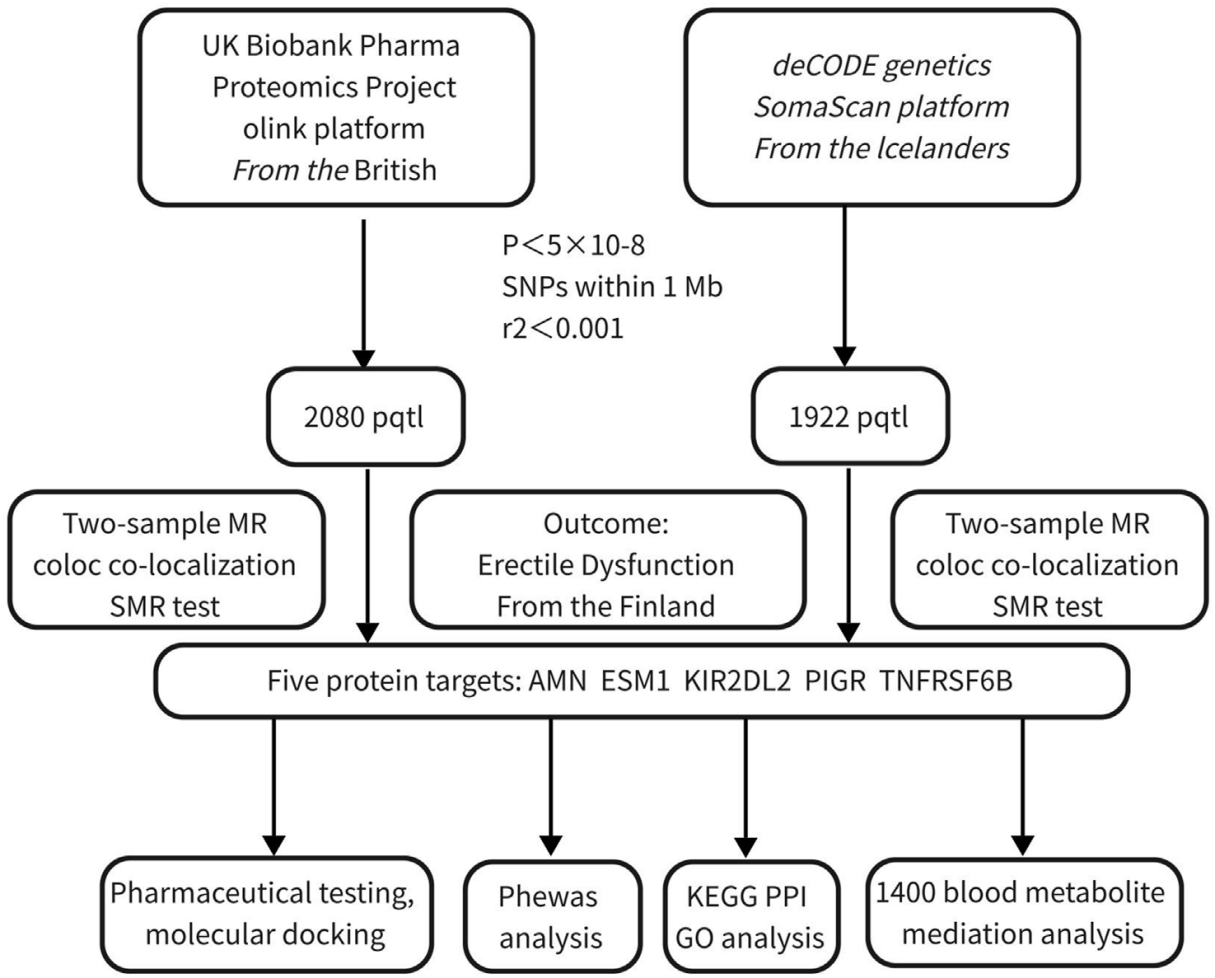
Workflow for identifying and validating protein targets associated with erectile dysfunction. This figure illustrates the multi-step process integrating Mendelian randomization, co-localization, and functional analyses to identify potential protein targets for ED.

In this study, the protein targets were selected from two of the largest publicly available proteomics databases: Olink and deCODE, originating from the United Kingdom and Iceland, respectively. ED outcome data were obtained from the latest Finnish dataset, the FinnGen R11 database, accessible at https://storage.googleapis.com/finngen-public-data-r11/summary_stats/finngen_R11_ERECTILE_DYSFUNCTION.gz. The database included 2,548 ED cases and 196,451 controls, all derived from the Finnish population. The mediation analysis included 1,400 blood metabolites sourced from large-scale genome-wide association studies (GWAS), comprising data on 1,091 metabolites and 309 metabolite ratios from 8,091 individuals in the Canadian Longitudinal Study on Aging (CLSA) cohort (Chen et al., 2023). All populations involved in this study were of European descent to minimize potential biases due to population heterogeneity. Furthermore, exposure, mediator, and outcome data were obtained from different countries to avoid sample overlap. This study did not require additional ethical approval or consent. The workflow is illustrated in Figure 1.

### 2.2 MR analysis

In the MR analysis, the proteomics data from the two databases were treated as exposures, while ED data served as the outcomes. Proteins from the Olink database were sourced from the UK Biobank, and those from the deCODE database were derived from Iceland, with ED data obtained from Finland. MR instruments were constructed using pQTLs, including both cis- and trans-pQTLs. When only one pQTL was available, the Wald ratio was used to estimate the MR effect, while the inverse-variance weighted (IVW) method was applied when two or more pQTLs were available. To ensure the robustness of the results and address potential heterogeneity or horizontal pleiotropy in the instruments, sensitivity analyses were performed. If heterogeneity was detected using Cochran’s Q test, the weighted median method allowing up to 50% invalid instruments was employed. Similarly, if horizontal pleiotropy was identified through MR-Egger, this method was used to correct for pleiotropy. The MR analysis was conducted using the TwoSampleMR (version 4.3.1) package. To enhance reliability, clumping utilized the latest 1000 Genomes Project data (https://cncr.nl/ctg/) instead of the default older version in the TwoSampleMR package. FDR correction was applied to all MR results.

### 2.3 Coloc colocalization analysis

Bayesian colocalization analysis was performed to evaluate whether the loci identified in MR shared a single causal variant influencing both protein levels and ED. This analysis estimated the posterior probability that each genomic locus contains a variant affecting both traits, rather than coincidentally sharing a variant due to correlation. The coloc R package (http://cran.r-project.org/web/packages/coloc) was used to test colocalization. The posterior probability for hypothesis 4 (PPH4) was employed, where PPH4/(PPH4 + PPH3) >0.7 indicated high colocalization probability, a threshold validated in a previous study (Zhao et al., 2023) Notably, a limitation of Bayesian colocalization analysis is its assumption of a single shared causal SNP; however, in reality, genetic loci may harbor multiple causal SNPs.

### 2.4 SMR analysis

As coloc colocalization analysis employed a relatively broad threshold, SMR analysis was conducted using ED outcome data for further validation. This analysis assessed whether the expression levels of identified genes were causally linked to ED. The heterogeneity in dependent instruments (HEIDI) test was used to evaluate whether observed associations were due to linkage disequilibrium between distinct causal variants. A significant SMR p-value (<0.05) combined with a non-significant HEIDI p-value (>0.05) suggested a causal relationship without pleiotropy or heterogeneity, indicating that observed associations might not be influenced by significant linkage disequilibrium between different genetic variants (Wu et al., 2018).

### 2.5 Phenome-wide association study

PheWAS analysis was performed using the AstraZeneca PheWAS portal (https://azphewas.com/) to infer potential side effects of identified drug targets. This portal utilized data from the UK Biobank, encompassing approximately 1.5k binary phenotypes and 450k continuous phenotypes derived from 15.5k exome-sequenced participants.

### 2.6 Mediation MR analysis

To enrich and deepen our understanding of the relationship between protein drug targets and disease outcomes, and given the significant association between metabolic processes and ED, 1,091 blood metabolites and 309 metabolite ratios were used as exposures. Protein targets meeting SMR and colocalization criteria were treated as mediators, with ED as the outcome (Chen et al., 2023). This analysis explored which metabolic substances drive the occurrence of ED through these protein targets.

### 2.7 KEGG, GO, and PPI analyses

The biological functions and pathways of the five drug target genes were explored using Gene Ontology (GO) and Kyoto Encyclopedia of Genes and Genomes (KEGG) enrichment analyses, implemented via the R package clusterProfiler. GO terms were divided into biological processes (BP), molecular functions (MF), and cellular components (CC). KEGG provided pathway information. The STRING database (https://string-db.org/) was used to construct a PPI network to explore potential interactions among the five drug target genes. The results were visualized using Cytoscape.

### 2.8 Drug target analysis

The DrugBank and DSigDB databases were utilized to study the interactions between identified proteins and drugs, further investigating whether the identified drug target genes could serve as effective therapeutic interventions. Comprehensive molecular information on drug mechanisms, interactions, and targets was collected, including drug names and the development process targeting the identified proteins.

### 2.9 Molecular docking

Following the druggability analysis, molecular docking was performed for drugs directly or indirectly related to ED. The 3D structures of core co-targets were downloaded from the PDB database (https://www.rcsb.org/) in. pdb format, while small-molecule drug structures were retrieved from the PubChem database (https://pubchem.ncbi.nlm.nih.gov/) in. sdf format. Docking simulations were conducted using the CB-Dock2 tool (Liu et al., 2022). A binding energy below 0 indicated spontaneous binding between the ligand molecule and the receptor protein, with lower binding energy suggesting a tighter interaction. The docking parameters were centered on the coordinates of compounds originally bound to the target protein pocket to construct grids.

## 3 Results

### 3.1 MR, coloc colocalization, and SMR results

**FIGURE 2:**
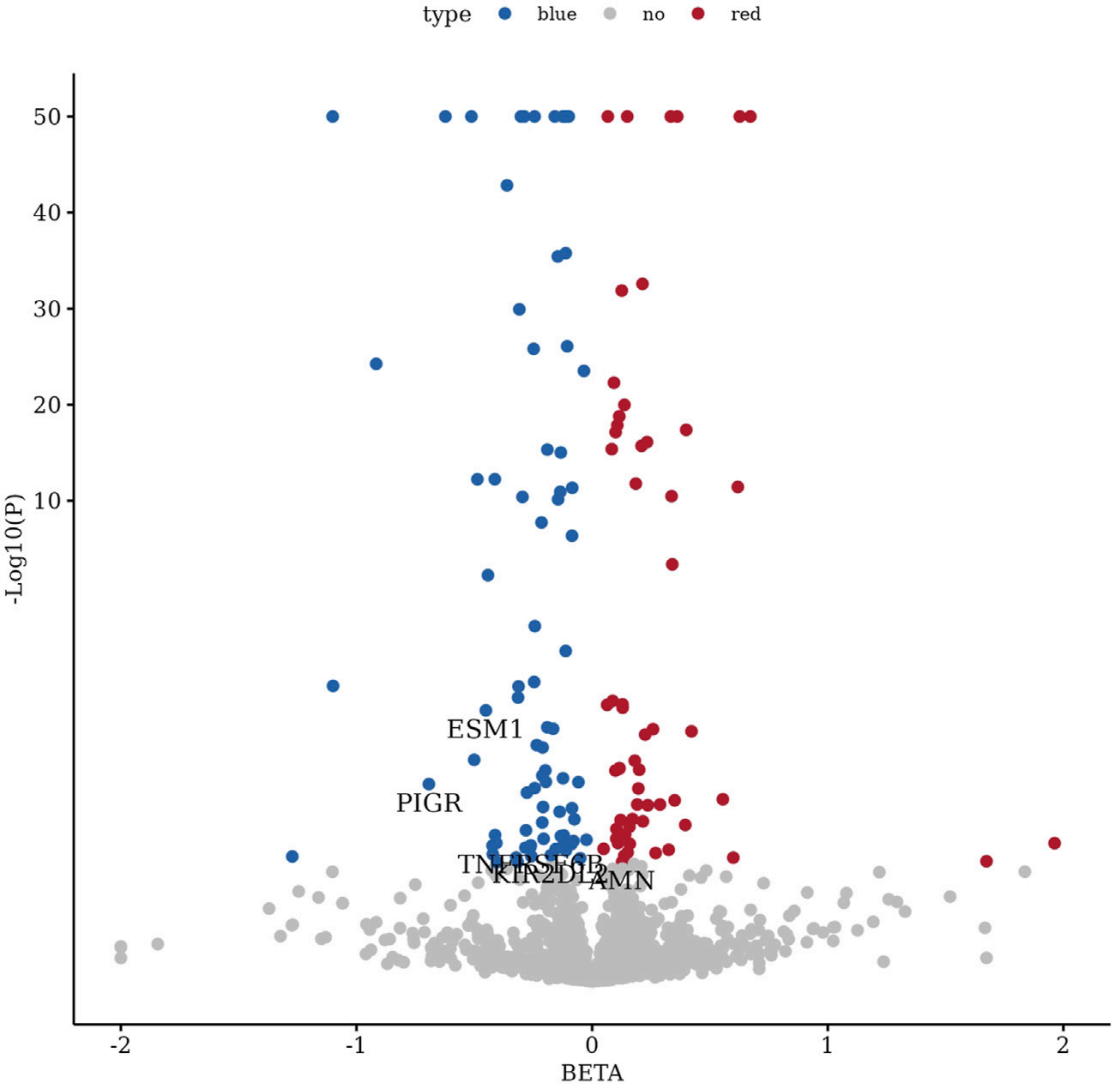
Volcano plot of differentially expressed genes with highlighted protein targets. This volcano plot displays the distribution of differentially expressed genes, with five protein targets (AMN, ESM1, KIR2DL2, PIGR, and TNFRSF6B) specifically annotated as satisfying two-sample Mendelian randomization, colocalization, and SMR analysis criteria.

**FIGURE 3:**
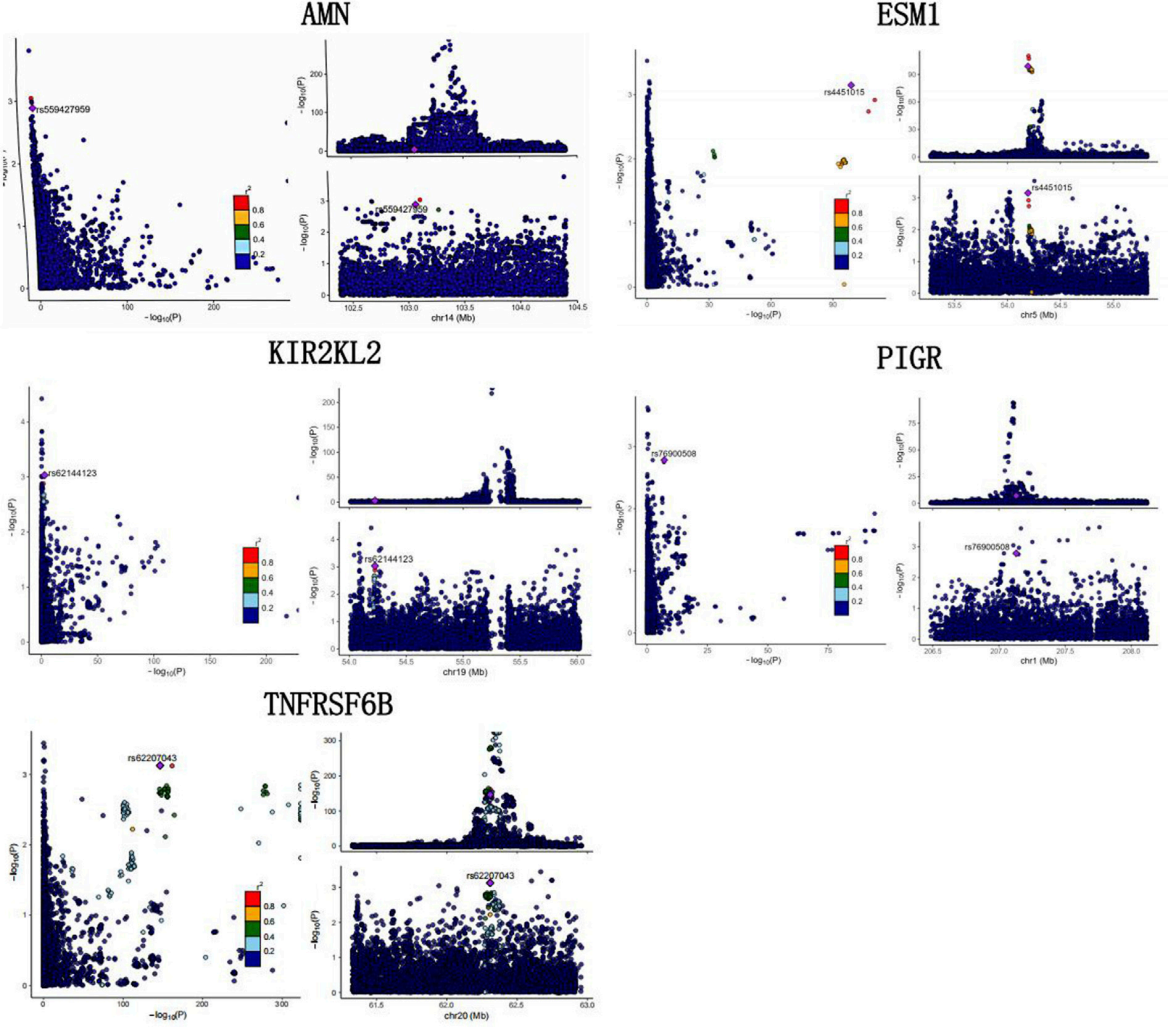
Colocalization analysis of genomic loci and protein targets. This figure presents the colocalization analysis of genomic loci with protein targets, highlighting key regions associated with AMN, ESM1, KIR2DL2, TNFRSF6B, and PIGR. The results demonstrate the overlap of significant loci with identified protein targets, supporting their potential causal role.

**FIGURE 4:**
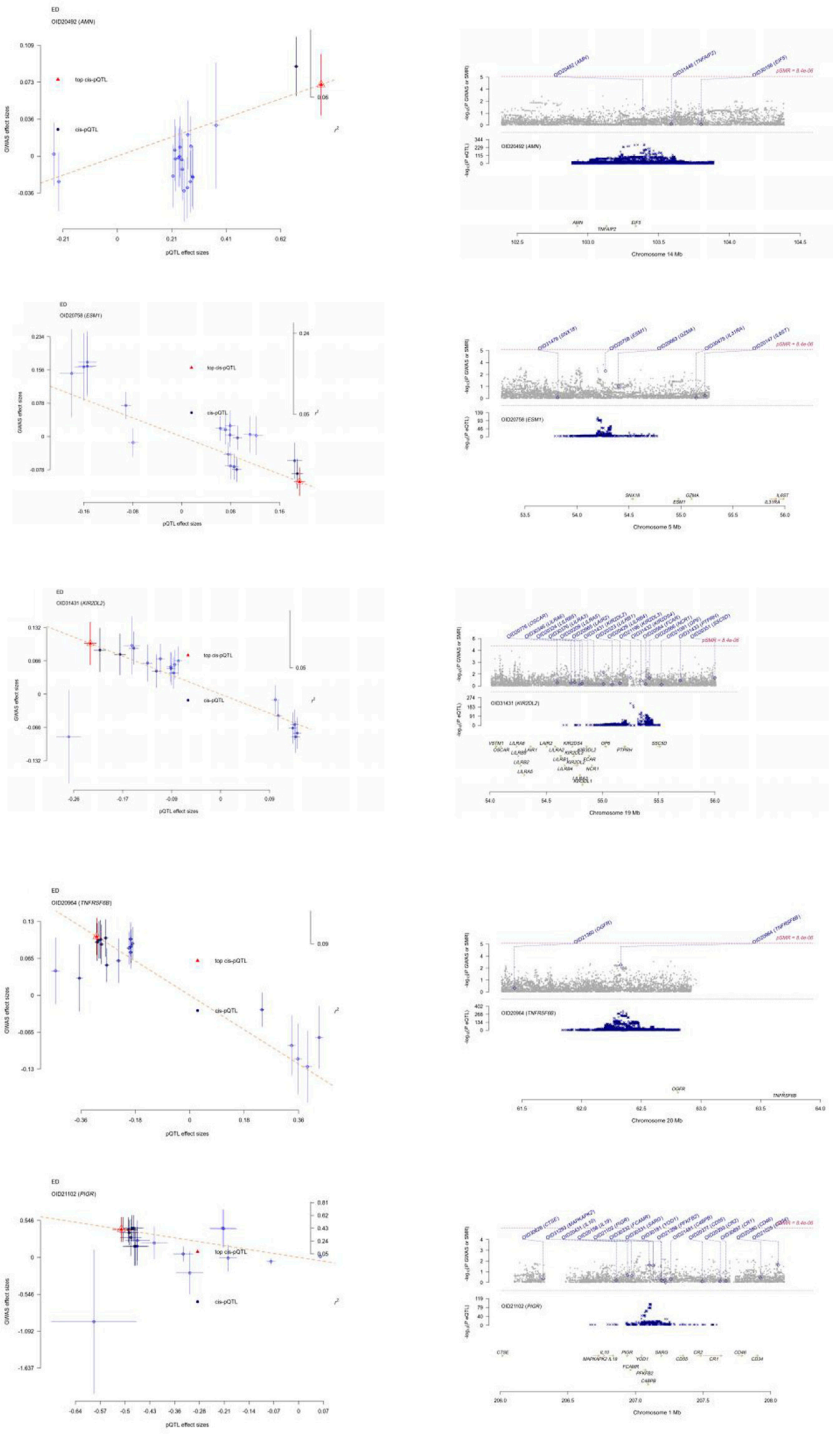
SMR analysis visualization of protein targets. This figure depicts the SMR (summary data-based Mendelian randomization) analysis results, highlighting the association between genetic loci and protein targets, including AMN, ESM1, KIR2DL2, TNFRSF6B, and PIGR, with significant evidence supporting their causal links.

We conducted large-scale two-sample MR analyses using proteins from Olink and deCODE as exposures and ED data from the FinnGen database as outcomes. To ensure accuracy, FDR correction was applied to the results (Supplementary Files 1, 2; Figure 2). Coloc colocalization analysis identified eight protein targets (AMN, ESM1, KIR2DL2, PIGR, SPINT1, SPP1, TNFRSF6B, TMEM9) that met the colocalization criteria (Tables 1, 2; Figure 3). To further validate the colocalization hypothesis, SMR colocalization analysis was performed, which confirmed only AMN, ESM1, KIR2DL2, PIGR, and TNFRSF6B met the criteria (Supplementary Files 3, 4; Figure 4).

**TABLE 1:** Colocalization results of proteins satisfying two-sample MR from deCODE.

SNP	PP.H1	PP.H2	PP.H3	PP.H4	lead_snp	protein	PP_H4_new
rs17658212	0.202362766	3.50E-88	0.014841112	0.782796122	rs17658212	SPINT1	0.797637234
rs116845898	0.202361018	3.50E-88	0.014849623	0.78278936	rs17658212	SPINT1	0.797638982
rs149882005	0.202064688	3.83E-88	0.016292239	0.781643073	rs17658212	SPINT1	0.797935312
rs4915486	0.243977523	1.36E-10	0.135835489	0.620186988	rs4915486	TMEM9	0.820328769

This table presents the colocalization analysis results for proteins identified through two-sample Mendelian randomization using deCODE data. Key metrics include the posterior probabilities (PP.H1, PP.H2, PP.H3, PP.H4) and the lead SNPs for each protein. Highlighted proteins include SPINT1 and TMEM9, with high PP.H4 values indicating strong colocalization evidence.

**TABLE 2:** Colocalization results of proteins satisfying two-sample MR from Olink.

SNP	PP.H1	PP.H2	PP.H3	PP.H4	lead_snp	protein	PP_H4_new
rs34341375	0.792668861	3.35E-302	0.056770104	0.150561035	rs2295828	AMN	0.726186313
rs572345833	0.792401376	3.36E-302	0.057088395	0.150510229	rs2295828	AMN	0.725005907
rs145444215	0.794747959	3.20E-302	0.054296097	0.150955943	rs2295828	AMN	0.735466224
rs183344017	0.796275254	3.09E-302	0.052478705	0.151246041	rs2295828	AMN	0.742403875
rs2295828	0.799276558	2.88E-302	0.048907327	0.151816114	rs2295828	AMN	0.756344715
rs34823966	0.799304904	2.88E-302	0.048873598	0.151821498	rs2295828	AMN	0.756478365
rs34334082	0.795418591	3.15E-302	0.053498084	0.151083325	rs2295828	AMN	0.738499776
rs375122321	0.789765648	3.55E-302	0.060224758	0.150009594	rs2295828	AMN	0.713535122
rs113468655	0.555510284	3.18E-105	0.056892499	0.387597216	rs4242051	ESM1	0.872004914
rs4242051	0.557691014	2.97E-105	0.053190206	0.38911878	rs4242051	ESM1	0.879744234
rs1848502	0.565614911	2.23E-105	0.039737553	0.394647536	rs4242051	ESM1	0.908519988
rs113420280	0.763312534	2.05E-302	0.051476441	0.185211026	rs1743325	KIR2DL2	0.782513026
rs17836409	0.767391591	1.85E-302	0.046407636	0.186200773	rs1743325	KIR2DL2	0.800490291
rs1743325	0.76995699	1.72E-302	0.043219767	0.186823244	rs1743325	KIR2DL2	0.812123105
rs10407012	0.781115187	1.17E-302	0.029354131	0.189530682	rs1743325	KIR2DL2	0.865892336
rs587595313	0.783213259	1.07E-302	0.026746981	0.19003976	rs1743325	KIR2DL2	0.876620773
rs35676942	0.782884537	1.08E-302	0.027155465	0.189959999	rs1743325	KIR2DL2	0.874926161
rs554343355	0.733373586	5.37E-89	0.063230736	0.203395678	rs291100	PIGR	0.762848943
rs291102	0.72904436	5.84E-89	0.068760641	0.202194999	rs291100	PIGR	0.746229158
rs189405663	0.729361328	5.80E-89	0.068355764	0.202282908	rs291100	PIGR	0.747427951
rs189699872	0.728920832	5.85E-89	0.068918428	0.20216074	rs291100	PIGR	0.745762728
rs76468397	0.729240523	5.81E-89	0.068510073	0.202249404	rs291100	PIGR	0.746970728
rs568240863	0.799725476	4.17E-150	0.050122895	0.15015163	rs139788985	PRTG	0.749729054
rs117454282	0.354944982	2.61E-301	0.021304445	0.623750573	rs17658212	SPINT1	0.96697267
rs17658212	0.354568336	2.74E-301	0.022342977	0.623088687	rs17658212	SPINT1	0.965382893
rs3189773	0.354478473	2.77E-301	0.022590756	0.622930771	rs17658212	SPINT1	0.965003869
rs187449482	0.353970433	2.93E-301	0.023991584	0.622037983	rs17658212	SPINT1	0.962863025
rs577552479	0.202539233	3.95E-301	0.013982022	0.783478746	rs17658212	SPINT1	0.982466822
rs537884673	0.787062096	1.16E-11	0.045988853	0.16694905	rs149807582	SPP1	0.784026927
rs140463299	0.723007523	4.36E-303	0.083032147	0.19396033	rs6062497	TNFRSF6B	0.700236817
rs111377612	0.723050649	4.36E-303	0.082977452	0.1939719	rs6062497	TNFRSF6B	0.700387629
rs546095294	0.724628068	4.26E-303	0.08097686	0.194395072	rs6062497	TNFRSF6B	0.705936405
rs6062497	0.72489733	4.24E-303	0.080635363	0.194467307	rs6062497	TNFRSF6B	0.706889928
rs550702011	0.724785786	4.25E-303	0.080776832	0.194437383	rs6062497	TNFRSF6B	0.706494696
rs192335495	0.727169341	4.09E-303	0.077753843	0.195076816	rs6062497	TNFRSF6B	0.715010609
rs189423538	0.725151201	4.22E-303	0.080313387	0.194535412	rs6062497	TNFRSF6B	0.707790658

This table provides the colocalization analysis results for proteins identified using Olink data through two-sample Mendelian randomization. The table highlights the posterior probabilities (PP.H1, PP.H2, PP.H3, PP.H4) and lead SNPs associated with each protein. Key findings include: AMN: Lead SNP rs2295828, PP.H4 values range from 0.1505 to 0.1518, indicating consistent colocalization. KIR2DL2: Lead SNP rs1743325, PP.H4 values range from 0.1852 to 0.1900, showing strong evidence of colocalization. PIGR: Lead SNP rs291100, PP.H4 values around 0.2022, supporting colocalization. SPINT1: Lead SNP rs17658212, PP.H4 values up to 0.965, indicating robust colocalization. TNFRSF6B: Lead SNP rs6062497, PP.H4 values around 0.1944, confirming colocalization evidence.

### 3.2 Phewas results

The PheWAS analysis provided insights into associations between the five protein targets and specific diseases or traits. AMN showed significant associations with inflammation-related phenotypes within the effective threshold (-log10P > 6–8), indicating a strong genetic link between AMN and inflammatory phenotypes. ESM1 exhibited a trend toward association with inflammatory phenotypes, although not reaching genome-wide significance in the current dataset. KIR2DL2 was not included in the database. PIGR demonstrated no genome-wide significant associations, but some phenotypes exhibited trends near P < 5e-5, particularly those related to proteomics and neurological traits. TNFRSF6B had multiple loci with P-values surpassing the genome-wide significance threshold (-log10P = 8), indicating strong associations with neurological diseases (Supplementary File 5).

### 3.3 Mediation analysis results

To identify upstream markers of protein targets and elucidate deeper mechanisms through which protein targets influence ED, 1,400 blood metabolites were used as exposures, five protein targets as mediators, and ED as the outcome. 1-Palmitoyl-2-linoleoyl-GPC (GPC) mediated AMN expression (P = 0.03, OR:1.059), exacerbating ED as a full mediator. N-formylmethionine levels (P = 0.03, OR:1.043) and the glycine-to-serine ratio (P = 0.0008, OR: 1.058) mediated ESM1 expression, reducing ED occurrence, acting as full mediators. Stearoyl sphingomyelin (d18:1/18:0) levels (SSI) increased TNFRSF6B expression, enhancing its protective effect against ED; however, the product-of-coefficients method yielded a non-significant result (P > 0.05). Hydantoin-5-propionate levels (HPL) promoted ED occurrence (P = 0.0004,OR:1.365) and decreased TNFRSF6B expression (P = 0.0001,OR:0.950),with the coefficient product method showing a significant mediation effect (P = 0.00015,beta indirect: 0.040). GPC promoted PIGR expression (P = 0.007, OR: 1.126) but lacked direct causal links with ED, acting as a full mediator.

### 3.4 KEGG, GO and PPI protein networks

**FIGURE 5:**
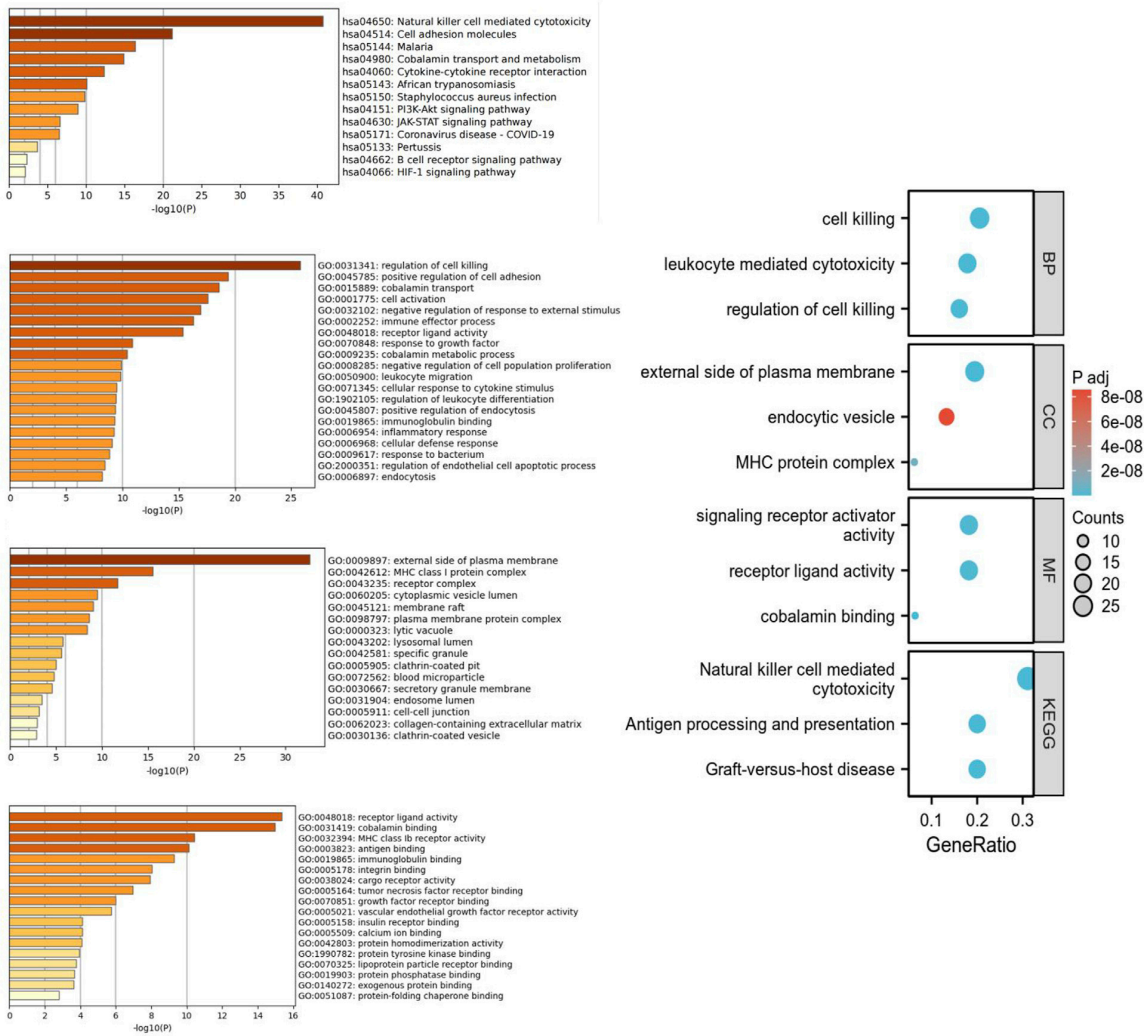
KEGG and GO enrichment analysis of significant protein targets. This figure illustrates the KEGG pathway and Gene Ontology (GO) enrichment analysis for the identified protein targets, highlighting their involvement in key biological processes, molecular functions, and relevant signaling pathways.

**FIGURE 6:**
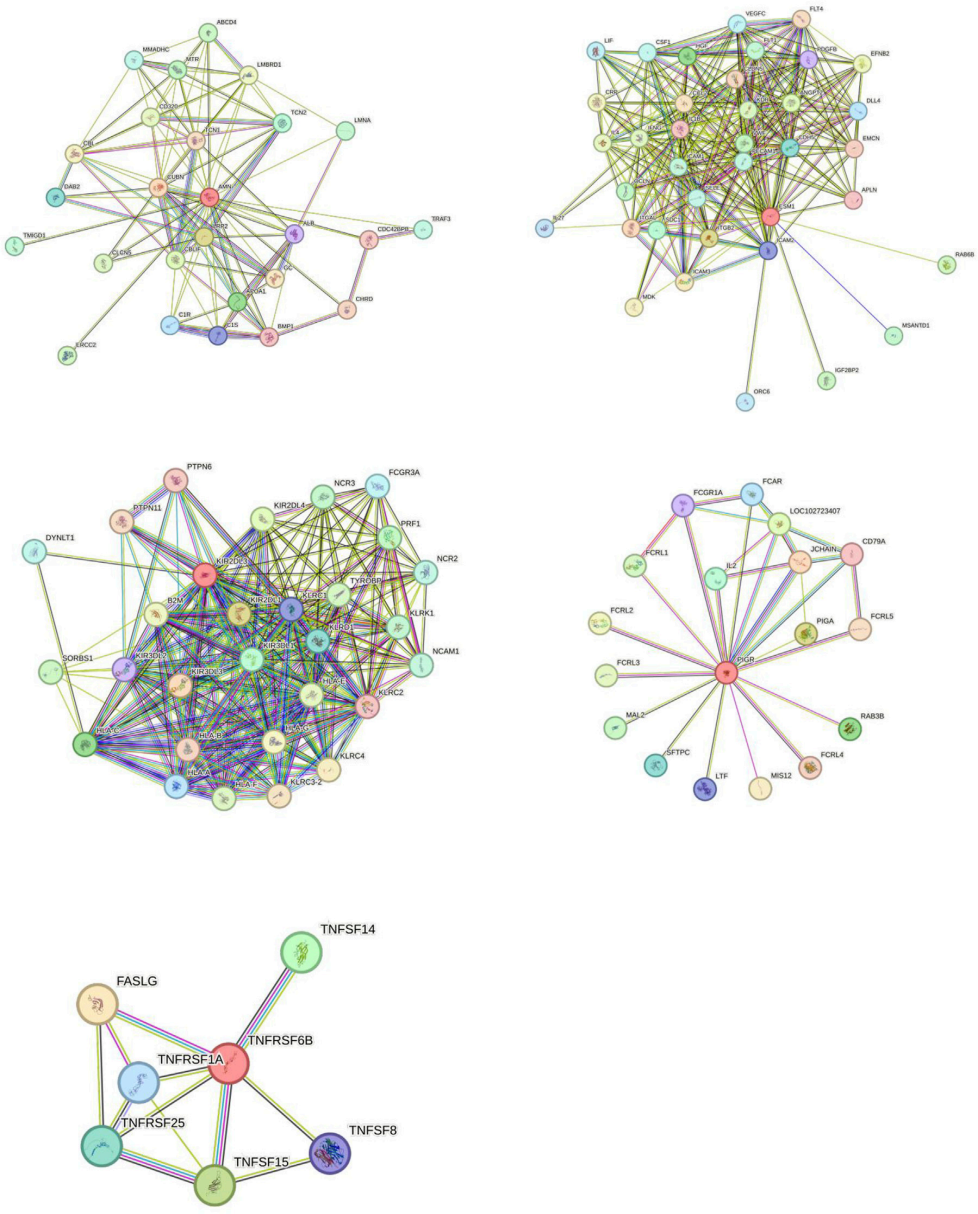
Protein-protein interaction (PPI) network of identified targets. This figure displays the protein-protein interaction (PPI) network for the five identified targets (AMN, ESM1, KIR2DL2, PIGR, and TNFRSF6B), illustrating their interconnected roles and potential interactions within biological systems. Key nodes represent the protein targets, and edges indicate predicted or known interactions, providing insights into their functional relationships.

The enrichment analysis of the five proteins using Gene Ontology (GO) and KEGG pathways revealed significant associations across biological processes (BP), cellular components (CC), molecular functions (MF), and KEGG pathways. BP: The proteins were mainly involved in processes related to cytotoxicity, leukocyte-mediated cytotoxicity, and regulation of cytotoxicity, highlighting their roles in immune surveillance and defense mechanisms. CC: Significant enrichment was observed in components such as the external side of the plasma membrane, endocytic vesicles, and MHC protein complexes, indicating roles in membrane dynamics, vesicle-mediated transport, and antigen presentation. MF: Enriched molecular functions included signal receptor activator activity, receptor ligand activity, and cobalamin binding, reflecting diverse roles in receptor-mediated signaling and vitamin B12 metabolism. KEGG Pathways: The KEGG pathway analysis linked these proteins to immune-related pathways such as natural killer cell-mediated cytotoxicity, antigen processing and presentation, and graft-versus-host disease, which are crucial in immune regulation, particularly in cancer immunotherapy and transplantation contexts (Figure 5). The PPI network revealed interactions among the five proteins and other related proteins (Figure 6).

### 3.5 Druggability analysis of protein targets

Using the DrugBank and DSigDB databases, we identified several drugs corresponding to the protein targets. AMN was associated with chlortetracycline and LEAD. KIR2DL2 was linked to clopamide, chlorzoxazone, dirithromycin, ajmaline, danazol, and latamoxef. PIGR was linked to isoguanine and AGN-PC-0JHFVD. TNFRSF6B was linked to cephaeline, topotecan, and atrazine.

### 3.6 Molecular docking

**FIGURE 7:**
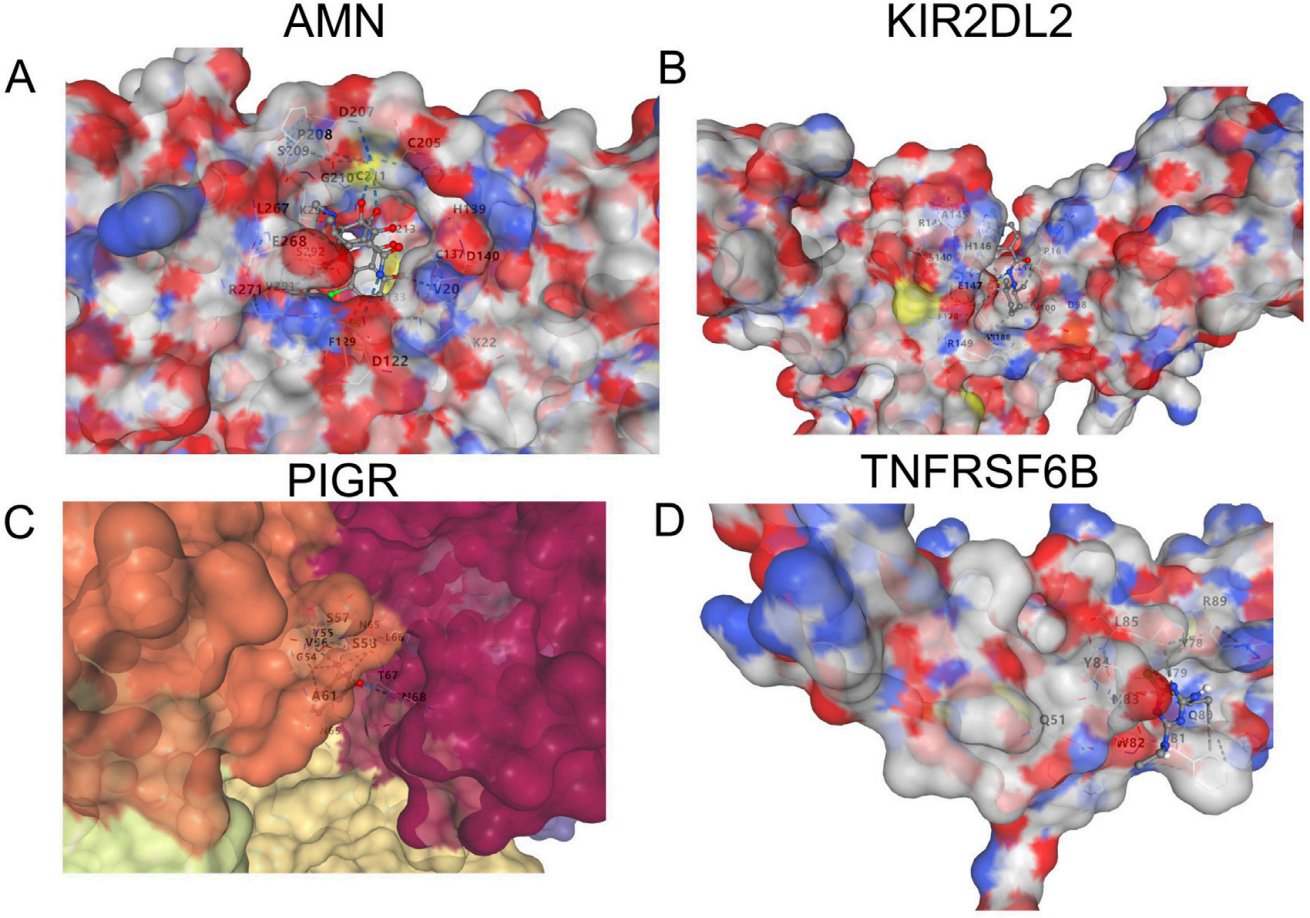
Structural analysis of protein targets with molecular docking. This figure shows the structural visualization and molecular docking results of the four identified protein targets: **(A)** AMN, **(B)** KIR2DL2, **(C)** PIGR, and **(D)** TNFRSF6B. The 3D surface representation highlights the binding sites and key residues involved in interactions, providing insights into the structural basis of their functional roles.

We conducted molecular docking experiments to examine the stability and interaction mechanisms between each protein and its most relevant drug candidate for ED. The docking results included Vina scores and active molecular residues involved in the binding: AMN with chlortetracycline (Vina score: 8.6; active residues: L267, E268, D122, P208), KIR2DL2 with clopamide (Vina score: 6.6; active residues: A145, S140, E147, F178, R149), PIGR with isoguanine (Vina score: 6.4; active residues: S58, S57, V56), and TNFRSF6B with atrazine (Vina score: 4.6; active residues: Q51, Y84, W82, L85) (Figure 7).

## 4 Discussion

ED is recognized as a chronic vascular inflammatory disease. With the increasing focus on the relationship between ED and inflammation or immune states, its association with chronic conditions such as cardiovascular diseases, diabetes, and metabolic syndrome has garnered attention. These conditions indirectly influence erectile function by impairing vascular and neural functions, thereby increasing ED risk (Terentes-Printzios et al., 2022; Yafi et al., 2016). Metabolic processes are particularly implicated in ED development. For instance, the key characteristics of metabolic syndrome (MetS), such as visceral obesity, hyperglycemia, hypertension, and abnormal lipid levels, are strongly associated with increased ED risk. These factors collectively contribute to endothelial dysfunction, considered a core pathological mechanism in ED. Moreover, components of MetS (e.g., hypertension and hyperglycemia) exacerbate chronic inflammatory states, further elevating ED risk (Besiroglu et al., 2015; Sanjay et al., 2015). In our study, large-scale two-sample MR, coloc colocalization, and SMR analyses identified five protein targets (AMN, ESM1, KIR2DL2, PIGR, TNFRSF6B) associated with ED.

AMN and TMEM9 are transmembrane and transport-related proteins. AMN, initially identified as being related to embryonic development, has since been found to play a pivotal role in metabolic regulation (Tanner et al., 2003). As a critical member of the metabolic regulation network, AMN is extensively involved in physiological metabolic processes, including the regulation of blood metabolites, energy metabolism, lipid metabolism, and inflammatory response-associated metabolites. These metabolites are directly or indirectly linked to vascular function, inflammatory response, and intercellular signaling. Given AMN’s central role in metabolic regulation, it is closely associated with metabolic syndrome, a major risk factor for ED (Sanchez et al., 2017; Vlachopoulos et al., 2006). Elevated AMN levels may lead to excessive regulation of certain metabolites, such as lipid-related or inflammation-associated metabolites, causing metabolic imbalances. This amplifies inflammatory responses, which have been shown to be closely linked to vascular dysfunction. Inflammation can impair endothelial cell function, reduce vascular dilation capacity, and disrupt penile blood flow, thereby increasing ED risk (Kaya-Sezginer and Gur, 2020). Our two-sample MR analysis identified AMN as a risk factor for ED. Coloc analysis confirmed that genetic variants regulating AMN expression also influence ED occurrence, providing robust evidence of their causal association.

TMEM9 (Transmembrane Protein 9) is a key transmembrane protein involved in multiple physiological processes, especially in lysosomal function regulation and mTORC1 signaling pathways. Although research on TMEM9 is still in its early stages, evidence suggests its significant role in cellular metabolism, intracellular transport, and stress response regulation (Zhang et al., 2023; Jung et al., 2021). Its association with metabolic regulation and cellular stress responses may profoundly impact endothelial health and metabolic syndrome. Dysregulated metabolism and vascular dysfunction are known risk factors for ED. TMEM9 influences lysosomal function and autophagy through mTORC1 signaling pathways, processes vital for endothelial cell health. Decreased TMEM9 expression may lead to metabolic imbalances, oxidative stress, and increased cellular stress responses, negatively affecting vascular health (Wang et al., 2024). Since vascular dysfunction, especially endothelial impairment, is a core mechanism in ED, TMEM9 expression may inversely correlate with ED occurrence, consistent with our MR results. Colocalization analysis confirmed that genetic variants regulating TMEM9 expression are shared with ED risk, further supporting TMEM9 as a protective factor in ED. However, SMR analysis indicated that TMEM9 did not pass the heterogeneity test. This may be attributed to TMEM9’s involvement in multiple pathways, such as mTORC1 signaling, metabolic regulation, lysosomal function, autophagy, and inflammatory responses (Zhang et al., 2023; Baek et al., 2024; Jung et al., 2018). Genetic instruments may simultaneously affect ED through multiple pathways. While two-sample MR focuses on TMEM9 expression pathways for causal inference, it overlooks potential effects through other mechanisms. The HEIDI test in SMR revealed these genetic variants’ broad effects, suggesting they influence ED beyond TMEM9 expression. In contrast, AMN passed the SMR analysis, as this protein primarily participates in metabolic and vascular function regulation, particularly in vitamin B12 absorption and metabolic syndrome (Fyfe et al., 2004). AMN’s metabolic roles are directly linked to metabolic diseases and vascular dysfunction, with metabolic syndrome being a significant risk factor for ED. Its physiological functions are relatively specific and dense, indicating that AMN’s effect on ED risk stems directly from regulatory mechanisms in its gene region. PheWAS analysis further revealed that among significant associations (-log10^6−8^), AMN variations were primarily linked to inflammation-related phenotypes, underscoring its close association with inflammatory and vascular inflammatory responses. In mediation MR analysis, GPC levels were found to regulate AMN expression, which subsequently promoted ED occurrence, establishing AMN as a complete mediator. GPC, a key glycerophospholipid, plays vital roles in cellular signaling and lipid metabolism (Okazaki et al., 2019). Firstly, excessive GPC levels can lead to phospholipid accumulation in the cell membrane, disrupting normal lipid balance and potentially activating specific signaling pathways. GPC accumulation activates the mTOR pathway, a central regulator of cell growth, proliferation, and metabolism (Anaokar et al., 2019; Laplante and Sabatini, 2009). Furthermore, GPC may stimulate AMPK (adenosine monophosphate-activated protein kinase), a critical sensor of energy metabolism. By regulating the AMPK pathway, GPC affects energy balance, lipid, and glucose metabolism (Sun et al., 2014), which are strongly linked to ED risk. Phospholipid metabolic imbalance may activate AMN expression through metabolic signaling pathways. As a metabolic regulator, AMN responds adaptively to lipid accumulation, yet its overexpression exacerbates metabolic dysregulation, further impairing vascular health and lipid metabolism. Secondly, oxidative stress can activate the NF-κB signaling pathway, extensively involved in cellular stress responses and inflammation-related protein expression. Studies show that oxidative stress induces pro-inflammatory cytokines such as TNF-α and IL-6 through the NF-κB pathway, which can impact specific protein levels (Palomer et al., 2013). AMN is likely implicated in this mechanism as a metabolic regulatory protein involved in lipid metabolism, vitamin B12 absorption, and cellular homeostasis. Under metabolic stress or oxidative conditions, AMN may be upregulated to counteract stress, maintaining metabolic balance and mitigating inflammatory damage. However, this adaptive response could amplify metabolic disturbances, creating a feedback loop that further impairs vascular health and exacerbates ED (Poma, 2020) Drug development analysis identified chlortetracycline as the only candidate targeting AMN. Molecular docking studies revealed a strong binding affinity between chlortetracycline and AMN, with a Vina score of −8.6. Key amino acid residues, including L267, E268, D122, and P208, located within or around AMN’s active pocket, formed stable interactions with chlortetracycline. These interactions suggest a potential regulatory effect on AMN activity, influencing its role in ED pathogenesis. However, no experimental evidence currently supports a direct link between chlortetracycline and ED. Further studies are required to validate its therapeutic potential.

ESM1 and SPINT1 are closely associated with angiogenesis and vascular function regulation. Our two-sample MR analysis identified both proteins as protective factors against ED. ESM1, an endothelial cell-specific molecule, is actively involved in angiogenesis and vascular permeability regulation. It plays a crucial role in endothelial cell health, particularly in maintaining vascular permeability and blood flow regulation (Rocha et al., 2014). SPINT1, on the other hand, is a serine protease inhibitor that regulates angiogenesis and extracellular matrix stability primarily by inhibiting specific serine proteases. SPINT1 contributes to tissue repair and angiogenesis by modulating vascular remodeling, thereby supporting normal vascular function (Aimes et al., 2003). Unlike ESM1, which directly regulates endothelial cell function and vascular permeability, SPINT1 exerts its effects mainly through protease inhibition. Both ESM1 and SPINT1 passed colocalization analysis, suggesting they share genetic loci with ED. This finding is consistent with their strong ties to angiogenesis and vascular function regulation, aligning with endothelial dysfunction as a core pathological mechanism of ED (Pomeshkina et al., 2010) However, SPINT1 did not pass SMR analysis, indicating a lack of detectable direct causal association between its protein expression and ED. This result aligns with studies suggesting that protein function regulation during vascular remodeling is not always mediated through a single gene’s expression. SPINT1’s primary function is relatively indirect, relying on serine protease inhibition for vascular remodeling and extracellular matrix stability, which may reduce its direct association with ED risk. In PheWAS analysis, two of the three most significant associations for ESM1 were related to inflammation and showed negative effect sizes. This finding aligns with ESM1’s function, suggesting that it mitigates vascular damage by modulating inflammatory factors, thus exerting protective effects. Subsequent mediation analysis revealed that N-formylmethionine levels and the glycine-to-serine ratio significantly promoted ESM1 expression, which subsequently mediated a reduction in ED risk. N-formylmethionine. N-formylmethionine, a metabolite involved in mitochondrial function, may reduce reactive oxygen species (ROS) production by regulating mitochondrial stress responses, thereby creating a favorable cellular environment for ESM1 expression. This upregulation enhances endothelial cell protection (Minton et al., 2018). Additionally, N-formylmethionine might activate the NF-κB signaling pathway, promoting inflammatory responses and increasing the release of pro-inflammatory factors like TNF-α and IL-6, which can damage endothelial cells. ESM1 expression may increase as a defensive mechanism against inflammation and vascular damage, facilitating angiogenesis and vascular permeability regulation to mitigate inflammation’s adverse effects The glycine-to-serine ratio reflects the state of one-carbon metabolism and methylation cycles, closely linked to cellular function and metabolic homeostasis. Glycine and serine, critical amino acids in one-carbon metabolism, contribute to the folate pathway and serve as precursors for methyl donor synthesis. Serine can be converted into glycine, releasing one-carbon units essential for generating S-adenosylmethionine (SAM), a key methyl donor for DNA methylation (Yu et al., 2019). Higher glycine levels accompanied by lower serine levels suggest an increased availability of one-carbon units for methylation reactions, enhancing SAM levels and promoting DNA methylation. This methylation may activate genes associated with vascular regulation and endothelial function, including ESM1. Such processes are critical in ED pathology, as they influence vascular health and endothelial repair. ESM1 expression appears to be positively regulated by methylation states. In drug development studies, various compounds were identified as potentially related to ESM1, but only retinoic acid and piroxicam showed potential relevance to ED treatment. Retinoic acid, with its antioxidant properties, reduces ROS production, preventing oxidative stress-induced damage to endothelial cells. Additionally, its anti-inflammatory effects decrease pro-inflammatory cytokine release, potentially alleviating ED symptoms (Shi et al., 2018). Piroxicam, a non-steroidal anti-inflammatory drug (NSAID), inhibits cyclooxygenase (COX) activity, reducing prostaglandin synthesis and alleviating systemic and localized inflammation. Given the association of ED with chronic inflammation (e.g., vascular endothelial inflammation from atherosclerosis), piroxicam may help relieve ED symptoms by improving vascular permeability and blood flow (Mazumder and Borah, 2015). The primary mechanisms of these drugs—antioxidant and anti-inflammatory effects—indirectly enhance vascular function, potentially benefiting ED. However, further research is needed to validate their specific effects and safety. Lastly, no small molecules matched ESM1 for molecular docking, precluding further exploration of its binding properties and interactions with potential drugs. Further research is warranted to elucidate ESM1’s role in ED treatment and its therapeutic potential.

KIR2DL2, TNFRSF6B, and SPP1 play significant roles in immune regulation and inflammatory responses. KIR2DL2 is an inhibitory receptor primarily expressed on natural killer (NK) cells and certain T cells. By binding to HLA-C class I molecules, it transmits inhibitory signals, regulating NK cell activity. This mechanism is crucial for maintaining immune system balance and preventing autoimmune responses. KIR2DL2 ensures NK cells do not attack normal cells, thereby preserving immune tolerance towards self-tissues (Barani et al., 2018) TNFRSF6B, also known as DcR3 (Decoy Receptor 3), is a member of the tumor necrosis factor receptor superfamily (TNFRSF) and plays a role in immune regulation. Its main function as a “decoy receptor” involves binding to ligands such as FasL, LIGHT, and TL1A, thereby blocking their interactions with other TNF receptors. This inhibits apoptosis and excessive immune cell activation (Liu et al., 2014) SPP1, a secreted phosphoprotein, is involved in extracellular matrix formation, tissue repair, and bone remodeling. It plays a critical role in cell adhesion and signal transduction and is actively engaged in inflammatory responses and immune regulation. SPP1 interacts with integrins on cell surfaces to regulate immune cell migration and the release of pro-inflammatory cytokines, particularly in bone marrow and immune cells (Zhao et al., 2024). All three proteins were identified through two-sample MR and coloc colocalization analysis, suggesting a potential causal relationship with ED and a shared genetic locus. However, SPP1 did not pass the SMR test. KIR2DL2 and TNFRSF6B are both involved in immune regulation and the suppression of excessive immune responses. KIR2DL2 regulates NK cell activity to prevent immune overactivation, while TNFRSF6B inhibits apoptosis and inflammation by blocking the binding of FasL and LIGHT to their receptors. These mechanisms of immune suppression and anti-inflammatory action may provide direct and clear protective effects in the pathogenesis of ED, particularly in preventing endothelial dysfunction and vascular damage caused by inflammation (Porrata et al., 2024; Filliol et al., 2017) In contrast, SPP1 is actively involved in extracellular matrix remodeling and inflammatory responses. It acts as a pro-inflammatory factor that plays critical roles in tissue repair, fibrosis, and bone remodeling, especially in the activation of macrophages and T cells (Zhao et al., 2024; Chen et al., 2024). The effects of extracellular matrix remodeling and angiogenesis on ED may be indirect, involving complex downstream mechanisms that are not captured by SMR analysis. This limitation, coupled with the fact that SPP1 expression may not be solely regulated by genetic variation, could explain the weaker association between SPP1 expression and ED risk observed in SMR tests. PheWAS analysis of TNFRSF6B revealed associations with multiple neurological diseases, highlighting its role in neuroinflammation regulation. Excessive immune activation can lead to neuronal damage and chronic inflammation. TNFRSF6B mitigates this by acting as a “decoy receptor,” blocking apoptotic and inflammatory signals such as FasL and LIGHT, reducing neuronal damage and apoptosis, and maintaining neural health. KIR2DL2 was not included in the database for similar analysis. In subsequent two-sample MR mediation analyses, we observed that SSI increased TNFRSF6B protein expression, thereby enhancing TNFRSF6B’s protective role against ED. SSI itself also showed a protective effect on ED. This effect may be mediated through SSI’s role in lipid metabolism and cellular membrane stability, which potentially regulate TNFRSF6B expression. In the lipid metabolism-inflammation regulatory pathway, SSI may enhance TNFRSF6B expression by influencing sphingomyelin metabolism. Metabolites of SSI might stimulate inflammatory responses, and TNFRSF6B upregulation acts as a feedback mechanism to suppress excessive inflammation and apoptotic responses. However, when mediation effects were tested using the two-sample product method, the p-value exceeded 0.05, indicating no significant mediation effect. This may suggest that SSI directly impacts ED through pathways other than TNFRSF6B or that TNFRSF6B’s regulatory effect represents only a minor portion of SSI’s influence on ED. Even though SSI and TNFRSF6B independently showed significant associations with ED in pairwise MR analyses, SSI’s effects may be distributed across multiple pathways, diluting its mediation through TNFRSF6B. Alternatively, SSI might affect ED through diverse independent mechanisms, while TNFRSF6B regulation accounts for only a small part. These complex pathological mechanisms and competing pathways could reduce statistical power, leading to a non-significant mediation effect. HPL was identified through two-sample mediation analysis as a key factor in promoting ED. This effect is primarily driven by HPL’s ability to activate the NF-κB signaling pathway, triggering a robust inflammatory response. The inflammatory response leads to the release of pro-inflammatory cytokines such as TNF-α, IL-6, and IL-1β, which contribute to endothelial dysfunction and reduced nitric oxide (NO) production, ultimately exacerbating ED development (Lin et al., 2021). In addition to promoting inflammation, HPL also reduces the expression of TNFRSF6B, a protein that plays a protective role in vascular health. TNFRSF6B, known as a “decoy receptor,” works by binding to ligands such as FasL and TL1A, thereby preventing their interaction with pro-apoptotic receptors. This mechanism helps to inhibit excessive apoptosis and immune activation, thus reducing vascular damage and mitigating ED risk. However, by lowering TNFRSF6B expression, HPL compromises this protective response, making endothelial cells more susceptible to damage and apoptosis, and thereby increasing the risk of ED (Fukuda et al., 2013). Thus, HPL not only exacerbates ED through its pro-inflammatory effects but also indirectly promotes ED by reducing TNFRSF6B levels, undermining its protective functions. This dual effect underscores the complexity of HPL’s role in ED pathogenesis, with its impact on both inflammation and vascular protection contributing to the disease (Spengler et al., 2011). In molecular docking studies, atrazine was identified as a potential drug with relevance to ED. The docking analysis yielded a Vina score of −4.6, indicating moderate binding affinity between atrazine and TNFRSF6B. The docking pocket volume was 66 Å^3^, classified as small. Atrazine’s small molecular size likely facilitates its fitting into this compact pocket. Key interactions involved residues such as Q51, Y84, W82, and L85, which formed hydrogen bonds or hydrophobic interactions with atrazine, stabilizing the ligand-protein complex. In further mediation analysis, KIR2DL2 did not exhibit any causal relationships with blood metabolites. This suggests that KIR2DL2’s impact on ED is primarily through direct immune regulation, inflammation suppression, and apoptosis reduction, rather than indirect effects mediated by blood metabolites. It is likely that KIR2DL2’s protective association with ED stems from its direct role in immune suppression and tissue protection. KIR2DL2 may regulate the JAK/STAT signaling pathway, reducing inflammatory mediators such as IFN-γ released by activated NK cells (Mishra and Ivashkiv, 2023; Banerjee et al., 2019). This regulation minimizes direct vascular endothelial damage and inflammation-mediated responses involving macrophages and T cells. By reducing pro-inflammatory signals, KIR2DL2 helps maintain normal vascular function, reducing vascular contraction responses and offering direct protection against ED. KIR2DL2 may also act in synergy with anti-inflammatory pathways such as TGF-β signaling, further reducing chronic inflammation and preserving endothelial function for adequate penile blood flow. As an immunosuppressive factor, TGF-β may complement KIR2DL2-mediated inhibitory pathways, preventing excessive immune activation in local tissues (Sanjabi et al., 2017) In ED pathogenesis, chronic inflammation is a core factor in endothelial dysfunction. By binding HLA-C class I molecules, KIR2DL2 transmits inhibitory signals to NK cells, reducing their activity and preventing attacks on normal cells, including vascular endothelial cells (Moradi et al., 2021). This mechanism may suppress the over-release of pro-inflammatory cytokines such as TNF-α, IL-6, and IL-1β, and inhibit the excessive activation of NF-κB and other inflammatory pathways, thereby mitigating local inflammatory responses. KIR2DL2 not only modulates inflammation but also directly reduces endothelial cell apoptosis, a primary contributor to vascular dysfunction in ED. Specifically, KIR2DL2 may block the Fas-FasL apoptotic pathway, reducing Fas receptor-mediated apoptosis signaling and preventing excessive apoptosis (Zhao et al., 2018). Moreover, KIR2DL2 may inhibit the activity of cytotoxic T cells and macrophages, reducing inflammation-induced apoptosis and maintaining vascular structural integrity and blood flow (Fontela et al., 2022). Interactions between NK cell and T cell apoptotic signals may also exist, with KIR2DL2 indirectly reducing T cell activation and pro-inflammatory signaling by suppressing NK cell activity. KIR2DL2’s immunosuppressive effects may further collaborate with the PI3K/AKT signaling pathway, which regulates cell survival and anti-apoptotic processes. The PI3K/AKT pathway reduces apoptosis through the modulation of Bax/Bcl-2 proteins (Zhou et al., 2018). Through these mechanisms, KIR2DL2 minimizes endothelial apoptosis, preserving vascular function and normal penile blood flow. Molecular docking identified clopamide as the most relevant drug candidate for ED associated with KIR2DL2. Clopamide, a diuretic commonly used to treat hypertension and edema, may indirectly improve vascular function by reducing blood pressure, which is a known contributor to endothelial damage and reduced blood flow, factors impacting erectile function. Additionally, by reducing fluid overload, clopamide alleviates cardiovascular strain, promoting healthy circulation critical for erectile function (55). Docking analysis yielded a Vina score of −6.6, indicating strong binding affinity. The binding pocket volume was 158 Å^3^, a compact space conducive to tight binding. Key residues involved in clopamide binding included A145, S140, E147, F178, and R149, which stabilized the complex through hydrogen bonding and hydrophobic interactions. Notably, E147 and R149 likely formed strong electrostatic interactions with clopamide’s polar groups, enhancing binding stability. Further experimental validation is required to confirm clopamide’s effects within biological systems.

PIGR (Polymeric Immunoglobulin Receptor) is a critical transmembrane receptor widely expressed in epithelial cells, particularly in the respiratory, gastrointestinal, and urogenital tracts. Its primary function involves the transport of polymeric immunoglobulins (IgA and IgM) to maintain mucosal immune barriers, preventing pathogen invasion and modulating local immune responses (Wei and Wang, 2021). However, PIGR’s role extends beyond mucosal immunity, playing a significant part in systemic inflammatory responses and vascular function regulation. By facilitating immunoglobulin transport and antibody clearance, PIGR may reduce the persistence of chronic low-grade inflammation, which is closely linked to ED. A major pathological process in ED is endothelial dysfunction, often mediated by inflammation. Through IgA transport, PIGR regulates the NF-κB signaling pathway, decreasing the production of pro-inflammatory cytokines such as TNF-α, IL-6, and IL-1β. IgA antibodies inhibit NF-κB-mediated inflammatory responses by binding to receptors on macrophages and dendritic cells, thereby mitigating endothelial damage and maintaining vascular health, ultimately reducing ED risk (Sheng et al., 2021; Krawczyk et al., 2019; Turula and Wobus, 2018) In addition to NF-κB signaling, PIGR may regulate the JAK/STAT signaling pathway via IgA and IgM transport. This pathway is intricately involved in cytokine signaling, particularly pro-inflammatory cytokines like IL-4 (Wei and Wang, 2021; Zegeye et al., 2018; Schjerven et al., 2000). By suppressing JAK/STAT activation, PIGR reduces cytokine release, curbing chronic systemic inflammation. This mechanism protects endothelial cells from inflammatory damage, preserving vascular function. Oxidative stress is another critical factor in endothelial dysfunction, as high levels of reactive oxygen species (ROS) inhibit nitric oxide (NO) production, which is essential for vascular dilation (Salisbury and Bronas, 2015) By mitigating inflammatory responses, particularly through NF-κB pathway modulation, PIGR may reduce ROS levels (Sheng et al., 2021). Moreover, PIGR may enhance the expression of antioxidant enzymes such as superoxide dismutase (SOD) and glutathione peroxidase (GPx), thereby minimizing oxidative stress-induced endothelial damage. This protective role supports vascular function and penile blood flow, reducing ED risk. PIGR expression is regulated by genetic variants, such as pQTLs, which may influence immunoglobulin transport and inflammatory responses, thus impacting its expression levels. These genetic variants may also relate to the pathological mechanisms of ED. Colocalization (Coloc) analysis revealed that genetic variants regulating PIGR expression are shared with ED, suggesting these variants influence ED through immune regulation and inflammation. By facilitating IgA transport, PIGR maintains immune barrier homeostasis and indirectly regulates vascular health (Pausder et al., 2022). In our study, SMR analysis demonstrated robust results, indicating that PIGR’s function is relatively specific, with its genetic instrumental variables predominantly regulating ED via PIGR expression without evidence of pleiotropy. By reducing chronic inflammation and oxidative stress, PIGR protects vascular function and prevents ED progression. PheWAS analysis did not reveal strong associations, possibly because PIGR’s primary roles are limited to mucosal immunity and local antibody transport. Its systemic effects may manifest through complex biological pathways, and these localized functions may not translate into systemic phenotypes detectable in large-scale genome-wide analyses. Further mediation analysis revealed that GPC promotes PIGR expression, although GPC itself lacks a direct causal relationship with ED. GPC is a phospholipid metabolite broadly involved in cell membrane construction and signaling. Phospholipid metabolites regulate signaling pathways, especially in immune and epithelial cells (King et al., 2024) GPC may enhance PIGR transcription or translation through pathways such as PI3K/Akt or mTOR, increasing PIGR’s functional activity. Elevated GPC may also strengthen PIGR’s role in mucosal immunity, facilitating immunoglobulin transport and reducing inflammatory responses. For example, PIGR-mediated IgA transport suppresses pro-inflammatory cytokine release, reducing endothelial damage (Pausder et al., 2022). This process likely involves NF-κB pathway suppression, further alleviating the chronic inflammation associated with ED. As a metabolite, GPC does not directly act on vascular or inflammatory pathways; instead, its role lies in regulating cellular metabolism and membrane functions (King et al., 2024). PIGR, however, is a key protein in modulating inflammatory responses and immune balance. Since ED’s core pathological mechanisms involve chronic inflammation and vascular dysfunction, PIGR plays a pivotal role in reducing chronic low-grade inflammation and maintaining vascular health (Akhtar and Sharma, 2022; Xu et al., 2021; Hendrikx et al., 2023). Thus, GPC must mediate PIGR upregulation to exert its indirect protective effect on ED. In molecular docking and drug development studies, we identified isoguanine as a promising ligand for PIGR. The docking analysis yielded a Vina score of −6.4, indicating moderate binding affinity between isoguanine and PIGR. The binding cavity had a volume of 6,645 Å^3^, providing ample space for isoguanine binding. Key interacting residues included S58, S57, and V56, which formed hydrogen bonds or hydrophobic interactions with isoguanine. This binding site appears to be a critical region of PIGR, though further studies are needed to confirm its activity.

Our study has certain limitations. As it primarily relies on genetic data from specific populations (e.g., European populations), the findings may not be fully generalizable to other ethnicities or regions. Genetic diversity and environmental factors across populations could limit the global applicability of the results. Thus, future research should include more diverse populations to validate the findings and enhance their broader applicability. While MR helps mitigate confounding factors, it relies on key assumptions regarding the association between genetic variants and exposures. The results may be affected by pleiotropy, where some genetic variants influence multiple traits. Although pleiotropy was assessed through tests such as HEIDI, some genetic variants may still affect multiple pathways, potentially introducing bias in causal inference. Therefore, the possibility of residual pleiotropic effects cannot be entirely excluded.

This study utilized two-sample MR, colocalization analysis, and mediation effect analysis to uncover causal associations between various metabolites, proteins, and ED. Specifically, it highlighted complex interactions involving metabolic regulation, inflammatory responses, and vascular function modulation. Future research should validate these causal relationships by incorporating more diverse genetic datasets and conducting functional studies on protein-drug interactions. These efforts will aid in translating the findings into clinical applications, facilitating the identification of potential therapeutic targets or interventions, and offering novel strategies for ED prevention and treatment.

## Data Availability

The original contributions presented in the study are included in the article/Supplementary Material, further inquiries can be directed to the corresponding authors.

## Supplementary material

SUPPLEMENTARY MATERIAL 1 Two-sample MR results of deCODE proteins associated with ED. This supplementary table details the Mendelian Randomization (MR) results for proteins identified from the deCODE in association with erectile dysfunction (ED). It includes effect estimates (b), standard errors (se), p-values, adjusted p-values (adjp), and the methods applied (e.g., Inverse Variance Weighted or Wald Ratio). Highlighted proteins demonstrate statistically significant associations, supporting their potential causal role in ED.

SUPPLEMENTARY MATERIAL 2 Two-sample MR results of Olink proteins associated with ED. This supplementary table details the Mendelian Randomization (MR) results for proteins identified from the Olink dataset in association with erectile dysfunction (ED). It includes effect estimates (b), standard errors (se), p-values, adjusted p-values (adjp), and the methods applied (e.g., Inverse Variance Weighted or Wald Ratio). Highlighted proteins demonstrate statistically significant associations, supporting their potential causal role in ED.

SUPPLEMENTARY MATERIAL 3 SMR results of deCODE proteins associated with erectile dysfunction. This table summarizes the results of SMR (Summary data-based Mendelian Randomization) analysis for proteins derived from the deCODE dataset associated with erectile dysfunction (ED). The table includes effect sizes (b_SMR), standard errors (se_SMR), p-values (p_SMR), HEIDI test p-values (p_HEIDI), and the number of SNPs used in HEIDI analysis (nsnp_HEIDI). Highlighted proteins with significant SMR results and non-significant HEIDI test results suggest robust causal evidence without heterogeneity.

SUPPLEMENTARY MATERIAL 4 SMR results of Olink proteins associated with erectile. This supplementary table summarizes the results of SMR (Summary data-based Mendelian Randomization) analysis for proteins derived from the Olink dataset, focusing on their association with erectile dysfunction (ED). The table includes effect sizes (b_SMR), standard errors (se_SMR), p-values (p_SMR), HEIDI test p-values (p_HEIDI), and the number of SNPs used in the HEIDI test (nsnp_HEIDI). Proteins with significant SMR results and non-significant HEIDI p-values highlight robust causal evidence, supporting their potential role in ED.

SUPPLEMENTARY MATERIAL 5 PheWAS analysis for key protein targets. This figure presents the results of Phenome-Wide Association Studies (PheWAS) for the identified protein targets (AMN, ESM1, PIGR, and TNFRSF6B). The analysis explores the associations of these proteins with a wide range of phenotypic traits, highlighting potential clinical implications and their relevance to disease mechanisms. Significant associations are indicated with annotated data points.

## References

[B1] Aimes R. T. Zijlstra A. Hooper J. D. Ogbourne S. M. Sit M. L. Fuchs S. (2003). Endothelial cell serine proteases expressed during vascular morphogenesis and angiogenesis. Thromb. Haemost. 89 (3), 561–572. 10.1055/s-0037-1613388 12624642

[B2] Akhtar S. Sharma A. (2022). Endothelial dysfunction sustains immune response in atherosclerosis: potential cause for ineffectiveness of prevailing drugs. Int. Rev. Immunol. 41 (2), 123–134. 10.1080/08830185.2020.1866568 33439070

[B3] Anaokar S. Kodali R. Jonik B. Renne M. F. Brouwers J. F. H. M. Lager I. (2019). The glycerophosphocholine acyltransferase Gpc1 is part of a phosphatidylcholine (PC)-remodeling pathway that alters PC species in yeast. J. Biol. Chem. 294 (4), 1189–1201. 10.1074/jbc.RA118.005232 30514764 PMC6349126

[B4] Baek S. Chang J. W. Yoo S. M. Choo J. Jung S. Nah J. (2024). TMEM9 activates Rab9-dependent alternative autophagy through interaction with Beclin1. Cell Mol. Life Sci. 81 (1), 322. 10.1007/s00018-024-05366-1 39078420 PMC11335249

[B5] Banerjee P. P. Pang L. Soldan S. S. Miah S. M. Eisenberg A. Maru S. (2019). KIR2DL4-HLAG interaction at human NK cell-oligodendrocyte interfaces regulates IFN-gamma-mediated effects. Mol. Immunol. 115, 39–55. 10.1016/j.molimm.2018.09.027 30482463 PMC6543535

[B6] Barani S. Khademi B. Ashouri E. Ghaderi A. (2018). KIR2DS1, 2DS5, 3DS1 and KIR2DL5 are associated with the risk of head and neck squamous cell carcinoma in Iranians. Hum. Immunol. 79 (4), 218–223. 10.1016/j.humimm.2018.01.012 29408295

[B7] Besiroglu H. Otunctemur A. Ozbek E. (2015). The relationship between metabolic syndrome, its components, and erectile dysfunction: a systematic review and a meta-analysis of observational studies. J. Sex. Med. 12 (6), 1309–1318. 10.1111/jsm.12885 25872648

[B8] Birowo P. Deswanto I. Rasyid N. (2019). Epidemiology of erectile dysfunction: a cross-sectional web-based survey conducted in an Indonesian national referral hospital. F1000Res. 8 (817), 817. 10.12688/f1000research.18930.1

[B9] Chen S. Deng B. Zhao F. You H. Liu Y. Xie L. (2024). Silencing SPP1 in M2 macrophages inhibits the progression of castration-resistant prostate cancer via the MMP9/TGFβ1 axis. Transl. Androl. Urol. 13 (7), 1239–1255. 10.21037/tau-24-127 39100821 PMC11291415

[B10] Chen Y. Lu T. Pettersson-Kymmer U. Stewart I. D. Butler-Laporte G. Nakanishi T. (2023). Genomic atlas of the plasma metabolome prioritizes metabolites implicated in human diseases. Nat. Genet. 55 (1), 44–53. 10.1038/s41588-022-01270-1 36635386 PMC7614162

[B11] Chen Y. Xin X. Zhang H. Xu J. Gao Y. Tan A. (2014). Immunization associated with erectile dysfunction based on cross-sectional and genetic analyses. PLoS One 9 (10), e111269. 10.1371/journal.pone.0111269 25343742 PMC4208848

[B12] Filliol A. Farooq M. Piquet-Pellorce C. Genet V. Dimanche-Boitrel M. T. Vandenabeele P. (2017). RIPK1 protects hepatocytes from death in Fas-induced hepatitis. Sci. Rep. 7 (1), 9205. 10.1038/s41598-017-09789-8 28835677 PMC5569041

[B13] Fontela M. G. Snedal S. Abate-Daga D. (2022). 225 Killer cell immunoglobulin-like receptor 2DL2 (KIR2DL2) immune checkpoint as a modulator of T-cell effector function. J J. Immunother. Cancer 10 (Suppl. 2), A239. 10.1136/jitc-2022-SITC2022.0225

[B14] Fukuda K. Miura Y. Maeda T. Takahashi M. Hayashi S. Kurosaka M. (2013). Decoy receptor 3 regulates the expression of various genes in rheumatoid arthritis synovial fibroblasts. Int. J. Mol. Med. 32 (4), 910–916. 10.3892/ijmm.2013.1461 23912906

[B15] Fyfe J. C. Madsen M. Hojrup P. Christensen E. I. Tanner S. M. de la Chapelle A. (2004). The functional cobalamin (vitamin B12)-intrinsic factor receptor is a novel complex of cubilin and amnionless. Blood 103 (5), 1573–1579. 10.1182/blood-2003-08-2852 14576052

[B16] Hendrikx T. Lang S. Rajcic D. Wang Y. McArdle S. Kim K. (2023). Hepatic pIgR-mediated secretion of IgA limits bacterial translocation and prevents ethanol-induced liver disease in mice. Gut 72 (10), 1959–1970. 10.1136/gutjnl-2022-328265 36690432 PMC10841342

[B17] Jamaluddin B. M. Srivastava G. K. Gupta N. P. (2019). Role of serum high-sensitivity C-reactive protein as a predictor of therapeutic response to tadalafil in patients with erectile dysfunction: a prospective observational study. J. Sex. Med. 16 (12), 1912–1921. 10.1016/j.jsxm.2019.09.006 31668733

[B18] Jung Y. S. Jun S. Kim M. J. Lee S. H. Suh H. N. Lien E. M. (2018). TMEM9 promotes intestinal tumorigenesis through vacuolar-ATPase-activated Wnt/β-catenin signalling. Nat. Cell Biol. 20 (12), 1421–1433. 10.1038/s41556-018-0219-8 30374053 PMC6261670

[B19] Jung Y. S. Stratton S. A. Lee S. H. Kim M. J. Jun S. Zhang J. (2021). TMEM9-v-ATPase activates wnt/β-catenin signaling via APC lysosomal degradation for liver regeneration and tumorigenesis. Hepatology 73 (2), 776–794. 10.1002/hep.31305 32380568 PMC7647947

[B20] Kaya-Sezginer E. Gur S. (2020). The inflammation network in the pathogenesis of erectile dysfunction: attractive potential therapeutic targets. Curr. Pharm. Des. 26 (32), 3955–3972. 10.2174/1381612826666200424161018 32329680

[B21] King W. R. Singer J. Warman M. Wilson D. Hube B. Lager I. (2024). The glycerophosphocholine acyltransferase Gpc1 contributes to phosphatidylcholine biosynthesis, long-term viability, and embedded hyphal growth in Candida albicans. J. Biol. Chem. 300 (1), 105543. 10.1016/j.jbc.2023.105543 38072057 PMC10790099

[B22] Krawczyk K. M. Nilsson H. Nystrom J. Lindgren D. Leandersson K. Swärd K. (2019). Localization and regulation of polymeric ig receptor in healthy and diseased human kidney. Am. J. Pathol. 189 (10), 1933–1944. 10.1016/j.ajpath.2019.06.015 31404540

[B23] Laplante M. Sabatini D. M. (2009). An emerging role of mTOR in lipid biosynthesis. Curr. Biol. CB 19 (22), R1046–R1052. 10.1016/j.cub.2009.09.058 19948145 PMC3390254

[B24] Lin X. Tago K. Okazaki N. So T. Takahashi K. Mashino T. (2021). The indole-hydantoin derivative exhibits anti-inflammatory activity by preventing the transactivation of NF-κB through the inhibition of NF-κB p65 phosphorylation at Ser276. Int. Immunopharmacol. 100, 108092. 10.1016/j.intimp.2021.108092 34474272

[B25] Liu W. Zhan C. Nathenson S. Almo S. (2014). Crystal structures of FasL:DcR3 and LIGHT:DcR3 complexes reveal the molecular basis for the broad specificity of DcR3 (CCR5P.255). J. Immunol. 192 (1_Suppl. ment), 181.9–181.189. 10.4049/jimmunol.192.supp.181.9

[B26] Liu Y. Yang X. Gan J. Chen S. Xiao Z. X. Cao Y. (2022). CB-Dock2: improved protein-ligand blind docking by integrating cavity detection, docking and homologous template fitting. Nucleic Acids Res. 50 (W1), W159–W164. 10.1093/nar/gkac394 35609983 PMC9252749

[B27] Mazumder M. K. Borah A. (2015). Piroxicam confer neuroprotection in cerebral ischemia by inhibiting cyclooxygenases, acid- sensing ion channel-1a and aquaporin-4: an *in silico* comparison with aspirin and nimesulide. Bioinformation 11 (4), 217–222. 10.6026/97320630011217 26124563 PMC4479049

[B28] McMahon C. G. (2019). Current diagnosis and management of erectile dysfunction. Med. J. Aust. 210 (10), 469–476. 10.5694/mja2.50167 31099420

[B29] Minton D. R. Nam M. McLaughlin D. J. Shin J. Bayraktar E. C. Alvarez S. W. (2018). Serine catabolism by SHMT2 is required for proper mitochondrial translation initiation and maintenance of formylmethionyl-tRNAs. Mol. Cell 69 (4), 610–621. 10.1016/j.molcel.2018.01.024 29452640 PMC5819360

[B30] Mishra B. Ivashkiv L. (2023). Regulation of inflammatory NF-kB target gene activation by jak-STAT signaling. J. Immunol. 210 (1_Suppl. ment), 162.01–162.101. 10.4049/jimmunol.210.supp.162.01

[B31] Moradi S. Stankovic S. O'Connor G. M. Pymm P. MacLachlan B. J. Faoro C. (2021). Structural plasticity of KIR2DL2 and KIR2DL3 enables altered docking geometries atop HLA-C. Nat. Commun. 12 (1), 2173. 10.1038/s41467-021-22359-x 33846289 PMC8041999

[B32] Okazaki Y. Nakamura K. Takeda S. Yoshizawa I. Yoshida F. Ohshima N. (2019). GDE5 inhibition accumulates intracellular glycerophosphocholine and suppresses adipogenesis at a mitotic clonal expansion stage. Am. J. Physiol. Cell Physiol. 316 (2), C162-C174–C174. 10.1152/ajpcell.00305.2018 30462540 PMC6397339

[B33] Palomer X. Salvado L. Barroso E. Vázquez-Carrera M. (2013). An overview of the crosstalk between inflammatory processes and metabolic dysregulation during diabetic cardiomyopathy. Int. J. Cardiol. 168 (4), 3160–3172. 10.1016/j.ijcard.2013.07.150 23932046

[B34] Pausder A. Fricke J. Schughart K. Schreiber J. Strowig T. Bruder D. (2022). Exogenous and endogenous triggers differentially stimulate pigr expression and antibacterial secretory immunity in the murine respiratory tract. Lung 200 (1), 119–128. 10.1007/s00408-021-00498-8 34825965 PMC8881272

[B35] Poma P. (2020). NF-κB and disease. Int. J. Mol. Sci. 21 (23), 9181. 10.3390/ijms21239181 33276434 PMC7730361

[B36] Pomeshkina S. A. Pomeshkin E. V. Barbarash O. L. Neĭmark A. I. (2010). Cardiovascular diseases and erectile dysfunction. Ter. arkhiv 82 (10), 37–40. 21341462

[B37] Porrata L. F. Ansell S. M. Micallef I. N. Johnston P. B. Villasboas J. C. Paludo J. (2024). Day 100 recovery of absolute number of inhibitory KIR2DL2 and activating NKp30 natural killer cells predicts survival post-autologous stem cell transplantation in lymphomas. Biomedicines 12 (8), 1808. 10.3390/biomedicines12081808 39200272 PMC11351217

[B38] Rocha S. F. Schiller M. Jing D. Butz S. Vestweber D. (2014). Esm1 modulates endothelial tip cell behavior and vascular permeability by enhancing VEGF bioavailability. Circ. Res. 115 (6), 581–590. 10.1161/CIRCRESAHA.115.304718 25057127

[B39] Ruiz-Garcia A. Arranz-Martinez E. Cabrera-Velez R. Palacios-Martínez D. Rivera-Teijido M. García-Álvarez J. C. (2019). Prevalence of erectile dysfunction in Spanish primary care setting and its association with cardiovascular risk factors and cardiovascular diseases. SIMETAP-ED study. SIMETAP-ED study. Clin. Investig. Arterioscler. 31 (3), 101–110. 10.1016/j.arteri.2019.01.002 30979438

[B40] Salisbury D. Bronas U. (2015). Reactive oxygen and nitrogen species: impact on endothelial dysfunction. Nurs. Res. 64 (1), 53–66. 10.1097/nnr.0000000000000068 25502061

[B41] Sanchez E. Pastuszak A. W. Khera M. (2017). Erectile dysfunction, metabolic syndrome, and cardiovascular risks: facts and controversies. Transl. Androl. Urol. 6 (1), 28–36. 10.21037/tau.2016.10.01 28217448 PMC5313297

[B42] Sanjabi S. Oh S. A. Li M. O. (2017). Regulation of the immune response by TGF-β: from conception to autoimmunity and infection. Cold Spring Harb. Perspect. Biol. 9 (6), a022236. 10.1101/cshperspect.a022236 28108486 PMC5453394

[B43] Sanjay S. Bharti G. S. Manish G. Rajeev P. Pankaj A. Puspalata A. (2015). Metabolic syndrome: an independent risk factor for erectile dysfunction. Indian J. Endocrinol. Metab. 19 (2), 277–282. 10.4103/2230-8210.149322 25729692 PMC4319270

[B44] Schjerven H. Brandtzaeg P. Johansen F. E. (2000). Mechanism of IL-4-mediated up-regulation of the polymeric Ig receptor: role of STAT6 in cell type-specific delayed transcriptional response. J. Immunol. 165 (7), 3898–3906. 10.4049/jimmunol.165.7.3898 11034397

[B45] Sheng X. Guo Y. Tang Q. Tang X. Xing J. Chi H. (2021). Upregulation of polymeric immunoglobulin receptor expression in flounder (*Paralichthys olivaceus*) gill cells by cytokine tumor necrosis factor-α via activating PI3K and NF-κB signaling pathways. Mol. Immunol. 135, 170–182. 10.1016/j.molimm.2021.04.011 33901762

[B46] Shi H. Yan S. Guo Y. Shi B. Guo X. (2018). The pre-protective effect of vitamin A on LPS-induced oxidative stress of bovine mammary epithelial cells. Italian J. Animal Sci. 17 (4), 959–966. 10.1080/1828051X.2018.1453757

[B47] Spengler G. Handzlik J. Ocsovszki I. Viveiros M. Kiec-Kononowicz K. Molnar J. (2011). Modulation of multidrug efflux pump activity by new hydantoin derivatives on colon adenocarcinoma cells without inducing apoptosis. Anticancer Res. 31 (10), 3285–3288. 21965738

[B48] Sun Y. Li J. Xiao N. Wang M. Kou J. Qi L. (2014). Pharmacological activation of AMPK ameliorates perivascular adipose/endothelial dysfunction in a manner interdependent on AMPK and SIRT1. Pharmacol. Res. 89, 19–28. 10.1016/j.phrs.2014.07.006 25108154

[B49] Tanner S. M. Aminoff M. Wright F. A. Liyanarachchi S. Kuronen M. Saarinen A. (2003). Amnionless, essential for mouse gastrulation, is mutated in recessive hereditary megaloblastic anemia. Nat. Genet. 33 (3), 426–429. 10.1038/ng1098 12590260

[B50] Terentes-Printzios D. Ioakeimidis N. Rokkas K. Vlachopoulos C. (2022). Interactions between erectile dysfunction, cardiovascular disease and cardiovascular drugs. Nat. Rev. Cardiol. 19 (1), 59–74. 10.1038/s41569-021-00593-6 34331033

[B51] Turula H. Wobus C. E. (2018). The role of the polymeric immunoglobulin receptor and secretory immunoglobulins during mucosal infection and immunity. Viruses 10 (5), 237. 10.3390/v10050237 29751532 PMC5977230

[B52] Vlachopoulos C. Aznaouridis K. Ioakeimidis N. Rokkas K. Vasiliadou C. Alexopoulos N. (2006). Unfavourable endothelial and inflammatory state in erectile dysfunction patients with or without coronary artery disease. Eur. Heart J. 27 (22), 2640–2648. 10.1093/eurheartj/ehl341 17056702

[B53] Wang Z. Zhao P. Tian K. Qiao Z. Dong H. Li J. (2024). TMEM9 promotes lung adenocarcinoma progression via activating the MEK/ERK/STAT3 pathway to induce VEGF expression. Cell Death Dis. 15 (4), 295. 10.1038/s41419-024-06669-8 38664392 PMC11045738

[B54] Wei H. Wang J. Y. (2021). Role of polymeric immunoglobulin receptor in IgA and IgM transcytosis. Int. J. Mol. Sci. 22 (5), 2284. 10.3390/ijms22052284 33668983 PMC7956327

[B55] Wu Y. Zeng J. Zhang F. Zhu Z. Qi T. Zheng Z. (2018). Integrative analysis of omics summary data reveals putative mechanisms underlying complex traits. Nat. Commun. 9 (1), 918. 10.1038/s41467-018-03371-0 29500431 PMC5834629

[B56] Xu S. Ilyas I. Little P. J. Kamato D. Zheng X. (2021). Endothelial dysfunction in atherosclerotic cardiovascular diseases and beyond: from mechanism to pharmacotherapies. Pharmacol. Rev. 73 (3), 924–967. 10.1124/pharmrev.120.000096 34088867

[B57] Yafi F. A. Jenkins L. Albersen M. Corona G. Isidori A. M. Goldfarb S. (2016). Erectile dysfunction. Nat. Rev. Dis. Prim. 2, 16003. 10.1038/nrdp.2016.3 27188339 PMC5027992

[B58] Yao C. Hwang S.-j. Huan T. Joehanes R. Levy D. (2020). Abstract 15554: mendelian randomization of 436 circulating proteins identifies putatively causal proteins for cardiovascular disease and its risk factors. Circulation 142 (Suppl. l_3), A15554. 10.1161/circ.142.suppl_3.15554

[B59] Yu W. Wang Z. Zhang K. Chi Z. Xu T. Jiang D. (2019). One-carbon metabolism supports S-adenosylmethionine and histone methylation to drive inflammatory macrophages. Mol. Cell 75 (6), 1147–1160. 10.1016/j.molcel.2019.06.039 31420217

[B60] Zegeye M. M. Lindkvist M. Falker K. Kumawat A. K. Paramel G. Grenegård M. (2018). Activation of the JAK/STAT3 and PI3K/AKT pathways are crucial for IL-6 trans-signaling-mediated pro-inflammatory response in human vascular endothelial cells. Cell Commun. Signal 16 (1), 55. 10.1186/s12964-018-0268-4 30185178 PMC6125866

[B61] Zhang S. Lee S. H. Nie L. Huang Y. Zou G. Jung Y. S. (2023). Lysosomal TMEM9-LAMTOR4-controlled mTOR signaling integrity is required for mammary tumorigenesis. Cancer Commun. (Lond) 43 (1), 159–163. 10.1002/cac2.12382 36336962 PMC9859727

[B62] Zhao S. S. Yiu Z. Z. N. Barton A. Bowes J. (2023). Association of lipid-lowering drugs with risk of psoriasis: a mendelian randomization study. JAMA Dermatol 159 (3), 275–280. 10.1001/jamadermatol.2022.6051 36696131 PMC9878432

[B63] Zhao T. Xu Y. Ren S. Liang C. Zhou X. Wu J. (2018). The siRNA silencing of DcR3 expression induces Fas ligand-mediated apoptosis in HepG2 cells. Exp. Ther. Med. 15 (5), 4370–4378. 10.3892/etm.2018.5964 29725377 PMC5920343

[B64] Zhao Y. Huang Z. Gao L. Ma H. Chang R. (2024). Osteopontin/SPP1: a potential mediator between immune cells and vascular calcification. Front. Immunol. 15, 1395596. 10.3389/fimmu.2024.1395596 38919629 PMC11196619

[B65] Zhong L. Zhan X. Luo X. (2023). Higher systemic immune-inflammation index is associated with increased risk of erectile dysfunction: result from NHANES 2001-2004. Med. Baltim. 102 (45), e35724. 10.1097/MD.0000000000035724 PMC10637557 37960751

[B66] Zhou P. Xie W. Luo Y. Lu S. Dai Z. Wang R. (2018). Protective effects of total saponins of aralia elata (miq.) on endothelial cell injury induced by TNF-α via modulation of the PI3K/akt and NF-κB signalling pathways. Int. J. Mol. Sci. 20 (1), 36. 10.3390/ijms20010036 30577658 PMC6337668

