# Supplementary material for: Blood metabolites as mediators in erectile dysfunction: insights from a multi-center proteomics and genetic study
Source: Front Pharmacol. 2025 Jun 2;16:1568780. doi: 10.3389/fphar.2025.1568780 (PMC12171135; doi:10.3389/fphar.2025.1568780)
Supplement: Supplementary file 1 [file Supplementaryfile1.docx]

| Table. | | | | | |
| --- | --- | --- | --- | --- | --- |
| b | se | pval | method | protein_ID | adjp |
| -0.49272 | 0.4367175 | 0.259220656 | Wald ratio | KCNAB2 | 0.875138353 |
| -0.036641472 | 0.045092706 | 0.416458373 | IVW | SIGLEC12 | 0.971782639 |
| -0.85900468 | 0.702030156 | 0.221102847 | IVW | SGK3 | 0.84001855 |
| -0.039955947 | 0.079577115 | 0.615594723 | IVW | PLCG2 | 1 |
| -0.842965416 | 0.867181751 | 0.331013402 | Wald ratio | BRD2 | 0.917801961 |
| -0.061731568 | 0.760358255 | 0.935292859 | Wald ratio | EIF2B1 | 1 |
| -0.125484968 | 0.057874261 | 0.030140851 | IVW | APRT | 0.338776118 |
| 0.727275551 | 1.023018069 | 0.477138933 | Wald ratio | STUB1 | 0.994044169 |
| -0.198121287 | 0.191789298 | 0.30159672 | IVW | ENO2 | 0.894057446 |
| -0.049648215 | 0.189815704 | 0.793660605 | Wald ratio | STAT3 | 1 |
| 0.279896105 | 0.083634892 | 0.000817965 | IVW | PDGFRA | 0.021536022 |
| 0.029253973 | 0.086643103 | 0.735636277 | IVW | STAT6 | 1 |
| -0.058584598 | 0.035355442 | 0.097515873 | IVW | ANGPTL3 | 0.613926381 |
| -0.273408393 | 0.193966792 | 0.158668789 | Wald ratio | MCL1 | 0.754854981 |
| -0.404938447 | 1.155525159 | 0.726010973 | Wald ratio | STX12 | 1 |
| 0.072328708 | 0.103552938 | 0.484882787 | IVW | SCARA5 | 0.994044169 |
| -0.093174053 | 0.518375278 | 0.857354749 | Wald ratio | NPDC1 | 1 |
| -0.006223481 | 0.032995804 | 0.850395112 | IVW | KIR2DS2 | 1 |
| 0.085382771 | 0.129310204 | 0.509065086 | IVW | CLMP | 0.994044169 |
| 0.056172497 | 0.034591951 | 0.104405619 | IVW | TMEM190 | 0.626325739 |
| -0.094468996 | 0.284026831 | 0.7394318 | IVW | APOM | 1 |
| 0.127899971 | 0.524751729 | 0.807436643 | IVW | NUCB1 | 1 |
| 0.004923833 | 0.514066202 | 0.992357813 | Wald ratio | INSL5 | 1 |
| -1.254466245 | 0.618812236 | 0.042640298 | Wald ratio | ANTXR1 | 0.413729933 |
| -0.001311523 | 0.194399601 | 0.994617089 | Wald ratio | RPN1 | 1 |
| 0.034611383 | 0.034757379 | 0.319347543 | IVW | TMEM106A | 0.901121115 |
| 0.106931395 | 0.056787221 | 0.059697847 | IVW | COL6A3 | 0.486183311 |
| -0.061303986 | 0.060053006 | 0.307334389 | IVW | CSF2RB | 0.894057446 |
| 0.133644681 | 0.204817021 | 0.514073715 | Wald ratio | PTGDS | 0.994044169 |
| 0.201090171 | 0.28838272 | 0.485613114 | Wald ratio | MXRA8 | 0.994044169 |
| 0.259654424 | 0.53363773 | 0.626560014 | Wald ratio | PARP1 | 1 |
| -1.231308052 | 1.950926079 | 0.527948799 | Wald ratio | GLB1 | 0.994044169 |
| -0.205655482 | 0.107063107 | 0.054746719 | IVW | PCDH9 | 0.467658643 |
| -0.255812813 | 0.42296156 | 0.545302998 | Wald ratio | PGLYRP3 | 0.994044169 |
| -0.365384693 | 0.220430367 | 0.097398856 | IVW | SLITRK3 | 0.613926381 |
| -0.024115127 | 0.574968153 | 0.966545194 | Wald ratio | MGAT1 | 1 |
| 0.028652476 | 0.48591444 | 0.952979114 | IVW | HTN3 | 1 |
| 0.099698171 | 0.365578392 | 0.78507344 | IVW | APMAP | 1 |
| 0.294168461 | 1.13027718 | 0.794661504 | Wald ratio | TOR1AIP1 | 1 |
| -0.180212284 | 0.037932784 | 2.03E-06 | IVW | PLOD3 | 8.65E-05 |
| 0.031877984 | 0.393038609 | 0.93535726 | Wald ratio | CASC4 | 1 |
| -0.013967709 | 0.123046294 | 0.909621568 | IVW | MSMB | 1 |
| 0.005605145 | 0.072052014 | 0.937992683 | IVW | APLP2 | 1 |
| -0.016797322 | 0.057664385 | 0.770826042 | IVW | HTATIP2 | 1 |
| 0.054363472 | 0.052300664 | 0.298599528 | IVW | GNPTG | 0.894057446 |
| -0.660155136 | 0.076248204 | 4.80E-18 | IVW | STX4 | 3.69E-16 |
| -0.085686186 | 0.091944262 | 0.351369503 | IVW | SFTPB | 0.936346055 |
| 0.077772438 | 0.084213157 | 0.355736879 | IVW | GNRH2 | 0.936346055 |
| -0.077572961 | 0.062563026 | 0.215006087 | IVW | ACE | 0.829192218 |
| -0.454439716 | 0.381101655 | 0.233089937 | Wald ratio | SYK | 0.852052837 |
| 0.694079545 | 0.364276515 | 0.056732705 | Wald ratio | SERPINB1 | 0.474088079 |
| 0.058153222 | 0.073317952 | 0.427681787 | IVW | PROK2 | 0.971955213 |
| 0.157432056 | 0.271057491 | 0.561370618 | Wald ratio | CSGALNACT2 | 0.998107611 |
| 0.042542576 | 0.546393896 | 0.937938913 | IVW | CLEC4G | 1 |
| 0.095687813 | 0.237381466 | 0.686876692 | IVW | SERPINH1 | 1 |
| -0.11686868 | 0.144093496 | 0.41733038 | IVW | KLRB1 | 0.971782639 |
| -0.825603092 | 0.041141624 | 1.42E-89 | IVW | HABP4 | 3.04E-87 |
| 0.003420581 | 0.04436192 | 0.93853903 | IVW | PILRA | 1 |
| 0.065614937 | 0.097180318 | 0.49955664 | IVW | SMPD1 | 0.994044169 |
| -0.004914596 | 0.027728283 | 0.859318882 | IVW | B4GALT6 | 1 |
| -0.146251604 | 0.269878783 | 0.587876441 | IVW | HHIP | 1 |
| 0.114015207 | 0.456766513 | 0.802886055 | Wald ratio | A4GNT | 1 |
| -0.005014717 | 0.057747903 | 0.930800219 | IVW | EBI3 | 1 |
| -0.011621001 | 0.259978273 | 0.964346519 | IVW | OSMR | 1 |
| 0.67313069 | 0.507668135 | 0.184864007 | Wald ratio | STX8 | 0.795137666 |
| -0.022626162 | 0.054799408 | 0.679686754 | IVW | NTM | 1 |
| -0.676499608 | 0.626075294 | 0.279901636 | Wald ratio | GALNT13 | 0.880697634 |
| -0.003982435 | 0.043649337 | 0.927304264 | IVW | PLA2R1 | 1 |
| -0.005615586 | 0.857893414 | 0.994777257 | Wald ratio | GNGT2 | 1 |
| 0.123871915 | 0.108791645 | 0.254863324 | IVW | CYB5D2 | 0.875138353 |
| 0.021129758 | 0.062695059 | 0.736098589 | IVW | CD58 | 1 |
| -0.002764786 | 0.681089365 | 0.99676111 | Wald ratio | PLD3 | 1 |
| 0.017804992 | 0.241028744 | 0.94111317 | Wald ratio | SPINK7 | 1 |
| 0.038281823 | 0.089999723 | 0.670577975 | IVW | UCMA | 1 |
| -0.050130137 | 0.099789181 | 0.615414005 | IVW | PDXK | 1 |
| -0.379024922 | 0.19986072 | 0.057901187 | IVW | REG4 | 0.47762267 |
| 0.01320793 | 0.098464607 | 0.893292774 | IVW | HSPB1 | 1 |
| -0.013302032 | 0.043721423 | 0.76094111 | IVW | CHI3L1 | 1 |
| -0.625239819 | 0.520268477 | 0.229455056 | Wald ratio | ENO1 | 0.850634127 |
| 0.004028979 | 0.07150737 | 0.955068125 | IVW | SVEP1 | 1 |
| 0.031960037 | 0.075236896 | 0.670988259 | IVW | SPATA20 | 1 |
| 0.610751708 | 0.661252847 | 0.35567998 | Wald ratio | TRIO | 0.936346055 |
| -0.164902275 | 0.182549638 | 0.36635162 | Wald ratio | MTRF1L | 0.943871065 |
| -0.057024834 | 0.142123372 | 0.688247304 | IVW | COL1A1 | 1 |
| -0.0416436 | 0.039548991 | 0.292358238 | IVW | ANGPTL1 | 0.887697526 |
| 0.089868006 | 0.089687183 | 0.316335791 | IVW | KIAA1549L | 0.896023899 |
| -0.127406786 | 0.074910142 | 0.088981512 | IVW | KLK13 | 0.581709071 |
| 0.951372937 | 0.581834983 | 0.102022843 | Wald ratio | SPG20 | 0.626325739 |
| -0.152471391 | 0.129641904 | 0.239556313 | IVW | KRT5 | 0.857406393 |
| -0.012454622 | 0.066753683 | 0.851993246 | IVW | SVEP1 | 1 |
| -0.007597482 | 0.059730776 | 0.89878572 | IVW | CLEC12A | 1 |
| 1.438181363 | 1.960981964 | 0.463315373 | Wald ratio | TINAGL1 | 0.994044169 |
| -0.093955033 | 0.3004776 | 0.754519501 | IVW | COL6A3 | 1 |
| -0.184802939 | 0.082015728 | 0.024242623 | IVW | NAGPA | 0.309241723 |
| 0.073643426 | 0.184929394 | 0.690464668 | IVW | TBCE | 1 |
| 0.093369589 | 0.088375415 | 0.290734861 | IVW | TXNDC5 | 0.884165194 |
| 0.144457707 | 0.682675439 | 0.832415013 | Wald ratio | DNAJB9 | 1 |
| -0.130876349 | 0.171880186 | 0.446394651 | IVW | FGFBP3 | 0.983911145 |
| 0.129787512 | 0.245253744 | 0.596668892 | IVW | PCOLCE | 1 |
| 0.168277256 | 0.10903641 | 0.122754971 | IVW | ASL | 0.67523297 |
| -0.010728186 | 0.088242542 | 0.903234724 | IVW | UROS | 1 |
| 0.182624357 | 0.068956412 | 0.008087309 | IVW | QDPR | 0.150451017 |
| 0.353941063 | 0.324757488 | 0.275773643 | Wald ratio | ALDH1A1 | 0.880697634 |
| -0.029912478 | 0.040828229 | 0.463776778 | IVW | SELPLG | 0.994044169 |
| -0.217836905 | 0.37726288 | 0.563659755 | IVW | GSTT2B | 0.998482994 |
| -0.015648681 | 0.186167353 | 0.93301106 | IVW | ATF6 | 1 |
| -0.478376721 | 0.187155064 | 0.01058695 | IVW | COL11A2 | 0.176940155 |
| 0.170407298 | 0.334267258 | 0.610196093 | Wald ratio | CYB5A | 1 |
| -0.077967664 | 0.080157207 | 0.330710185 | IVW | NT5C2 | 0.917801961 |
| 0.108941445 | 0.217447148 | 0.616369752 | Wald ratio | TNR | 1 |
| 0.221947209 | 0.163258882 | 0.17399449 | IVW | CREB3L4 | 0.779527761 |
| 0.111643182 | 0.084737566 | 0.187665395 | IVW | PCBD1 | 0.803324922 |
| 1.854030075 | 0.889243108 | 0.037073568 | Wald ratio | MRE11A | 0.377012687 |
| 0.027296889 | 0.189781682 | 0.885632257 | Wald ratio | ARHGAP25 | 1 |
| -0.375074914 | 0.377399083 | 0.320299983 | IVW | TALDO1 | 0.901341972 |
| -0.689016385 | 0.579182971 | 0.234189848 | IVW | NHEJ1 | 0.852052837 |
| -0.331959596 | 0.256818743 | 0.196155249 | Wald ratio | DGCR14 | 0.81076487 |
| -0.348632083 | 1.101340782 | 0.751583248 | Wald ratio | GRB10 | 1 |
| 0.208023636 | 0.519283636 | 0.688716614 | Wald ratio | RRM1 | 1 |
| -0.146394271 | 0.217349544 | 0.500601881 | IVW | ADH5 | 0.994044169 |
| 0.089435039 | 0.032934369 | 0.00661651 | IVW | ADH7 | 0.129764605 |
| -0.417005814 | 0.347761628 | 0.230483646 | Wald ratio | SYK | 0.850634127 |
| -0.164225682 | 0.308977257 | 0.595062767 | Wald ratio | BLVRA | 1 |
| -0.013546897 | 0.148641939 | 0.927383109 | IVW | ATF6B | 1 |
| 0.642225746 | 0.413396455 | 0.120295441 | Wald ratio | WFDC2 | 0.670167646 |
| -0.059409693 | 0.072471925 | 0.412352088 | IVW | CA8 | 0.971782639 |
| -0.028269992 | 0.05586571 | 0.612832242 | IVW | FAH | 1 |
| -0.081767833 | 0.181288785 | 0.651963344 | IVW | PDLIM1 | 1 |
| -0.159348893 | 0.114229157 | 0.163017903 | IVW | RECQL | 0.760307047 |
| 0.204991004 | 0.203239054 | 0.313156841 | IVW | PYGL | 0.894057446 |
| 0.231562747 | 0.238406472 | 0.331401957 | Wald ratio | ALDH3A1 | 0.917801961 |
| 0.667654611 | 0.553647378 | 0.227848223 | Wald ratio | DYNLL2 | 0.850634127 |
| -0.036154168 | 0.041003922 | 0.377925731 | IVW | APOL1 | 0.953842685 |
| -0.246337805 | 0.074060845 | 0.000880531 | IVW | CD59 | 0.022565085 |
| -0.561318721 | 0.260315982 | 0.031060436 | Wald ratio | FABP1 | 0.346399722 |
| 0.229269461 | 0.536676647 | 0.669232036 | Wald ratio | HMBS | 1 |
| 0.067439225 | 0.095903717 | 0.481932861 | IVW | C10orf54 | 0.994044169 |
| -0.558550473 | 0.540805994 | 0.301692274 | Wald ratio | FOXO3 | 0.894057446 |
| 0.815833613 | 0.486961345 | 0.093864403 | Wald ratio | LIMA1 | 0.597375438 |
| 0.103693119 | 0.439573394 | 0.813514231 | Wald ratio | MUSK | 1 |
| 1.255834808 | 0.936560472 | 0.17995262 | Wald ratio | HMG20B | 0.79153876 |
| -0.103862616 | 0.138855895 | 0.454467076 | IVW | FKBP1B | 0.992816625 |
| 0.286692157 | 0.567601961 | 0.613493048 | Wald ratio | DNAJB6 | 1 |
| -0.559838365 | 0.80901416 | 0.488936889 | Wald ratio | DAPP1 | 0.994044169 |
| 0.115988106 | 0.449169014 | 0.796230795 | Wald ratio | HSF1 | 1 |
| 0.78151334 | 0.304948773 | 0.010384103 | Wald ratio | TAGLN2 | 0.17507234 |
| 0.063592438 | 0.1937922 | 0.742800093 | Wald ratio | DTX3L | 1 |
| 0.290495565 | 0.150172701 | 0.053062745 | IVW | CHST9 | 0.457338998 |
| 0.010899717 | 0.101516787 | 0.914496552 | IVW | SMAP1 | 1 |
| 0.600899917 | 0.271698082 | 0.026991149 | IVW | EVL | 0.318738692 |
| -0.232777632 | 0.572475 | 0.684289868 | Wald ratio | CLINT1 | 1 |
| 0.247448705 | 0.31771399 | 0.436073339 | Wald ratio | AGFG1 | 0.97684494 |
| -0.006384547 | 0.102780681 | 0.950468732 | IVW | CRABP2 | 1 |
| 0.158110142 | 0.449504907 | 0.725031114 | Wald ratio | LCN1 | 1 |
| 0.112180769 | 0.063345798 | 0.076572882 | IVW | S100A2 | 0.552451523 |
| 0.008673037 | 0.135658774 | 0.949023786 | IVW | CD7 | 1 |
| 0.00146246 | 0.581905654 | 0.997994738 | IVW | CBL | 1 |
| 0.080376576 | 0.146760473 | 0.583917264 | IVW | HSPA1L | 1 |
| -0.672085954 | 0.299522013 | 0.024841305 | Wald ratio | RPS6KA1 | 0.312271593 |
| 0.14591779 | 0.326605478 | 0.655040557 | IVW | RPIA | 1 |
| -0.126952837 | 0.05917095 | 0.031910781 | IVW | SHMT1 | 0.346511417 |
| 0.065835235 | 0.05747726 | 0.252037871 | IVW | EGFLAM | 0.870512524 |
| -0.09278476 | 0.569546233 | 0.87058932 | Wald ratio | ACAP2 | 1 |
| 0.041625652 | 0.483998628 | 0.931463511 | Wald ratio | CCM2 | 1 |
| -0.301614115 | 0.278968836 | 0.279619301 | Wald ratio | SARS2 | 0.880697634 |
| -0.261558191 | 0.298239437 | 0.380482622 | Wald ratio | SRI | 0.957182721 |
| 0.0097684 | 0.389905201 | 0.980012474 | Wald ratio | SNAP29 | 1 |
| 0.025023589 | 0.00867794 | 0.003931749 | IVW | RRAS2 | 0.087870019 |
| 0.076000129 | 0.010647023 | 9.46E-13 | IVW | CRYGD | 6.20E-11 |
| 0.036894948 | 0.295191237 | 0.900534161 | IVW | APOF | 1 |
| -0.270696225 | 0.364562482 | 0.457770194 | Wald ratio | CDKN2D | 0.993644616 |
| -0.005727076 | 0.144745729 | 0.96843877 | IVW | TAPBP | 1 |
| -0.143476281 | 0.113512223 | 0.206240048 | IVW | CBR1 | 0.821672375 |
| -0.012915923 | 0.344229192 | 0.970069366 | Wald ratio | DDX58 | 1 |
| -0.079494134 | 0.092597956 | 0.390623652 | IVW | RNPEP | 0.960408371 |
| 0.310061414 | 0.198645423 | 0.118552369 | Wald ratio | PDLIM4 | 0.669395967 |
| -0.225221239 | 0.352369153 | 0.522717058 | Wald ratio | DARS2 | 0.994044169 |
| -0.198497902 | 0.105053112 | 0.058824804 | IVW | HIBCH | 0.4811118 |
| -0.148068928 | 0.36753126 | 0.687040641 | IVW | STAMBPL1 | 1 |
| -0.700350309 | 0.443520062 | 0.114319441 | Wald ratio | RAB22A | 0.657850196 |
| -0.012134392 | 0.16239019 | 0.940434567 | Wald ratio | MAX | 1 |
| 0.229794872 | 0.422777328 | 0.586760528 | Wald ratio | ALOX15B | 1 |
| -0.024452951 | 0.611663477 | 0.968110838 | Wald ratio | THYN1 | 1 |
| 0.608847393 | 0.414929701 | 0.142280675 | Wald ratio | MOB1A | 0.718729001 |
| 0.197239703 | 0.31229237 | 0.527656989 | Wald ratio | LYPLAL1 | 0.994044169 |
| 0.032272785 | 0.703450633 | 0.963407629 | Wald ratio | PELO | 1 |
| -0.303456415 | 0.232670421 | 0.19215419 | Wald ratio | CACYBP | 0.806376316 |
| -0.037092053 | 0.068926791 | 0.5904825 | IVW | GSTO1 | 1 |
| -0.119575374 | 0.350102417 | 0.732694099 | Wald ratio | ULK3 | 1 |
| -0.054605481 | 0.076421615 | 0.47489996 | IVW | MPG | 0.994044169 |
| -0.750716475 | 0.547793103 | 0.170550223 | Wald ratio | ANKRD27 | 0.774504164 |
| -0.018968702 | 0.070614107 | 0.788218412 | IVW | PPIH | 1 |
| 0.162075511 | 0.165585908 | 0.327678736 | IVW | PMVK | 0.914076242 |
| 0.172718157 | 0.309649503 | 0.576990525 | Wald ratio | ADI1 | 1 |
| 0.054348666 | 0.038794151 | 0.161229022 | IVW | PLEKHA1 | 0.756504336 |
| -0.618079223 | 0.488686099 | 0.205951069 | Wald ratio | MAPRE1 | 0.821672375 |
| -0.056421611 | 0.060437887 | 0.350537588 | IVW | CLIC5 | 0.936346055 |
| -0.018436462 | 0.125955691 | 0.883627259 | IVW | GLRX2 | 1 |
| -0.19523795 | 0.322322112 | 0.54469864 | Wald ratio | GABARAPL2 | 0.994044169 |
| 0.31124717 | 0.547858491 | 0.56995621 | Wald ratio | VPS24 | 1 |
| 0.095942112 | 0.006694252 | 1.38E-46 | IVW | PDCD5 | 2.41E-44 |
| 0.932205414 | 0.608007962 | 0.125223503 | Wald ratio | RAD23B | 0.67523297 |
| -0.079754256 | 0.186222949 | 0.668452395 | IVW | SAT2 | 1 |
| -0.000266326 | 0.045031423 | 0.995281165 | IVW | HPGDS | 1 |
| 0.171577612 | 0.571270149 | 0.763914707 | Wald ratio | RAD51L3 | 1 |
| -0.408888727 | 0.214074182 | 0.056128979 | Wald ratio | UBE2C | 0.472774182 |
| 0.264095837 | 0.216061099 | 0.221586579 | IVW | UBASH3B | 0.84001855 |
| 0.110526987 | 0.073300369 | 0.131589339 | IVW | NT5C | 0.690821526 |
| -0.133707308 | 0.286666961 | 0.640914639 | IVW | TNFAIP8 | 1 |
| 0.091454191 | 0.566802469 | 0.871816894 | Wald ratio | CCT5 | 1 |
| -0.253958366 | 0.207071199 | 0.220036838 | IVW | ARL3 | 0.839108737 |
| -0.137405063 | 0.07559167 | 0.069105782 | IVW | TRIM3 | 0.527068706 |
| -0.005279069 | 0.360037142 | 0.988301382 | IVW | MTHFD1 | 1 |
| 0.127800555 | 0.117210422 | 0.275558356 | IVW | GCA | 0.880697634 |
| -0.04650893 | 0.120417978 | 0.699326985 | IVW | PSMB1 | 1 |
| 0.717181308 | 0.593779439 | 0.227114805 | Wald ratio | NRBP1 | 0.850634127 |
| -0.269704319 | 0.47929402 | 0.573631264 | Wald ratio | SEPT11 | 1 |
| -0.055367664 | 0.445910256 | 0.901182473 | Wald ratio | PTPN9 | 1 |
| -0.018495483 | 0.120186581 | 0.877696667 | IVW | TRDMT1 | 1 |
| -0.024434634 | 0.271044906 | 0.928168205 | IVW | CRAT | 1 |
| 0.086592825 | 0.135471404 | 0.522695213 | IVW | INPP5B | 0.994044169 |
| 0.265286957 | 0.47673913 | 0.577895428 | Wald ratio | ARRB1 | 1 |
| 0.101517834 | 0.377660502 | 0.788078111 | Wald ratio | RPE | 1 |
| 0.281582393 | 0.326430023 | 0.388350935 | Wald ratio | KLC1 | 0.960408371 |
| -0.295361357 | 0.338927421 | 0.383503574 | IVW | TSTA3 | 0.957360905 |
| -0.050505994 | 0.081442288 | 0.535162599 | IVW | ECH1 | 0.994044169 |
| 0.354100594 | 0.396028641 | 0.371253009 | Wald ratio | RAD1 | 0.950618773 |
| 0.132611702 | 0.069784189 | 0.057392245 | IVW | DARS | 0.476612162 |
| -0.510267145 | 0.37969059 | 0.178979907 | Wald ratio | FLII | 0.790803177 |
| 0.071243219 | 0.416269414 | 0.864108261 | IVW | CCBL2 | 1 |
| -0.113243632 | 0.330013289 | 0.731486593 | Wald ratio | SCIN | 1 |
| -0.264193866 | 0.445845564 | 0.553470222 | Wald ratio | HOMER2 | 0.994044169 |
| 0.076458833 | 0.08055899 | 0.342567892 | IVW | MPST | 0.936346055 |
| 0.212939788 | 0.293770462 | 0.468543496 | IVW | DECR1 | 0.994044169 |
| 0.106688533 | 0.292120991 | 0.714946924 | Wald ratio | ARPC1B | 1 |
| -0.930342571 | 0.877205543 | 0.288882932 | Wald ratio | SHMT2 | 0.884165194 |
| -0.156244783 | 0.267016051 | 0.558446003 | Wald ratio | PIP4K2A | 0.994482737 |
| -0.045594872 | 0.111731599 | 0.683218617 | IVW | ACLY | 1 |
| 0.190782259 | 0.141572987 | 0.177790476 | Wald ratio | NEK7 | 0.789176199 |
| 0.623297909 | 0.700508711 | 0.373584588 | Wald ratio | H1FX | 0.950951101 |
| -0.015056007 | 0.210987901 | 0.943111583 | IVW | AP1G2 | 1 |
| 0.030579527 | 0.045690552 | 0.503320305 | IVW | PTGFRN | 0.994044169 |
| 0.029434324 | 0.12188425 | 0.809172213 | IVW | PLEKHA7 | 1 |
| -0.37481158 | 0.238573991 | 0.116171121 | IVW | ELMO1 | 0.662918394 |
| -0.076458048 | 0.091238329 | 0.402028684 | IVW | GPCPD1 | 0.967123738 |
| -0.509217778 | 0.68904 | 0.459891824 | Wald ratio | DNAJC27 | 0.993644616 |
| -1.477702744 | 0.772873476 | 0.055881396 | Wald ratio | IRF2 | 0.472774182 |
| -0.165020929 | 0.159753336 | 0.301616397 | IVW | ACYP2 | 0.894057446 |
| 0.809922006 | 0.699165738 | 0.24669593 | Wald ratio | EHBP1 | 0.870512524 |
| 0.022997524 | 0.052647375 | 0.662240555 | IVW | TESC | 1 |
| 0.024089757 | 0.116090848 | 0.835613297 | IVW | POLM | 1 |
| 0.092504571 | 0.511713333 | 0.85654481 | Wald ratio | CASS4 | 1 |
| 0.022228292 | 0.087806432 | 0.800151477 | IVW | ECI2 | 1 |
| 0.365115328 | 0.567017518 | 0.519625701 | Wald ratio | IFI16 | 0.994044169 |
| -0.407454815 | 0.23467037 | 0.082513326 | Wald ratio | ALDH3B1 | 0.564379401 |
| 0.407089716 | 0.664293217 | 0.539997807 | Wald ratio | KRT20 | 0.994044169 |
| 0.279178368 | 0.621601518 | 0.653339646 | Wald ratio | MAP3K3 | 1 |
| -0.347397828 | 0.195192125 | 0.075112989 | Wald ratio | TSG101 | 0.550638249 |
| 0.165131042 | 0.197588376 | 0.403305365 | IVW | RFK | 0.967123738 |
| 0.079080714 | 1.694474653 | 0.962776438 | Wald ratio | GMFG | 1 |
| -0.150195402 | 0.253658709 | 0.553772855 | Wald ratio | CCS | 0.994044169 |
| 0.011917332 | 0.442581298 | 0.978518057 | Wald ratio | UNC45A | 1 |
| 0.085164083 | 0.160605414 | 0.595925388 | IVW | VARS | 1 |
| 0.054400863 | 0.004221774 | 5.41E-38 | IVW | BTC | 7.43E-36 |
| -0.005997768 | 0.162210005 | 0.970504677 | IVW | S100A6 | 1 |
| -0.117800119 | 0.149950229 | 0.432104914 | IVW | SECTM1 | 0.971955213 |
| 0.046994301 | 0.161680346 | 0.771309986 | IVW | RSPO3 | 1 |
| -0.03323507 | 0.093055204 | 0.720976263 | IVW | REG1A | 1 |
| 0.100864866 | 0.057595899 | 0.07990271 | IVW | FAM3D | 0.563075399 |
| 0.572032555 | 0.252398443 | 0.023427698 | Wald ratio | LYPD3 | 0.308411198 |
| 0.301133728 | 0.140214542 | 0.031740455 | IVW | NEGR1 | 0.346511417 |
| 0.13154081 | 0.191226941 | 0.491529599 | Wald ratio | FSTL1 | 0.994044169 |
| -0.333000807 | 0.249290973 | 0.181617374 | IVW | SPP1 | 0.79153876 |
| 0.055901367 | 0.061026591 | 0.359658221 | IVW | LUM | 0.93822612 |
| 0.035120527 | 0.028986754 | 0.225663217 | IVW | CD177 | 0.847118559 |
| -0.464700521 | 0.164267894 | 0.004670556 | IVW | SMOC1 | 0.100863014 |
| 0.08164314 | 0.073702204 | 0.267972591 | IVW | SERPINA10 | 0.880697634 |
| -0.194484752 | 0.065139652 | 0.002829685 | IVW | FLRT2 | 0.066325055 |
| -0.127983271 | 0.051878022 | 0.013624895 | IVW | FLRT3 | 0.216421891 |
| -0.206293461 | 0.119025996 | 0.083063704 | IVW | ISLR2 | 0.565758098 |
| 0.020042102 | 0.029188279 | 0.492303965 | IVW | VTN | 0.994044169 |
| -0.033786316 | 0.149239177 | 0.820897798 | IVW | DSC2 | 1 |
| -0.45116144 | 0.569930314 | 0.428589235 | Wald ratio | HK2 | 0.971955213 |
| -0.054342987 | 0.036380642 | 0.135245269 | IVW | SEMA5A | 0.700650694 |
| -0.045689584 | 0.114936989 | 0.690985121 | IVW | LTBP4 | 1 |
| 0.208141482 | 0.048133518 | 1.53E-05 | IVW | SHANK3 | 0.000565696 |
| 0.137461043 | 0.075129733 | 0.067302437 | IVW | B4GALT1 | 0.523705601 |
| 0.269851726 | 0.225769804 | 0.231988731 | Wald ratio | FH | 0.852052837 |
| -0.017056025 | 0.05431172 | 0.753491038 | IVW | PCSK1 | 1 |
| 0.159700418 | 0.168576966 | 0.343463394 | IVW | DEFB4A | 0.936346055 |
| -0.01355307 | 0.0727553 | 0.852222897 | IVW | SPINK2 | 1 |
| -0.092206824 | 0.046326976 | 0.046552567 | IVW | TMEM132D | 0.430163625 |
| -0.404320043 | 0.308661638 | 0.190225103 | Wald ratio | FAM213A | 0.804842439 |
| 0.017557522 | 0.132114055 | 0.894275074 | IVW | MAN1C1 | 1 |
| -0.084695404 | 0.104696644 | 0.418538117 | IVW | PARVA | 0.971782639 |
| -0.17093177 | 0.655138593 | 0.794162432 | Wald ratio | CHRD | 1 |
| 1.084449259 | 0.729929304 | 0.137360923 | Wald ratio | SF3B4 | 0.701725102 |
| -0.902473943 | 0.491445428 | 0.066303464 | Wald ratio | USP8 | 0.518029501 |
| -0.31867376 | 0.078139742 | 4.54E-05 | IVW | CHAD | 0.001530015 |
| 0.143367609 | 0.176449807 | 0.416497826 | IVW | PXDN | 0.971782639 |
| 0.597939197 | 0.25914209 | 0.021033662 | IVW | RCAN1 | 0.286918722 |
| -0.098467838 | 0.121967911 | 0.419478819 | IVW | HDHD2 | 0.971782639 |
| 0.0951 | 0.411767832 | 0.817349459 | Wald ratio | ITPKA | 1 |
| -0.154898142 | 0.213020647 | 0.467133559 | Wald ratio | TCEA2 | 0.994044169 |
| 0.002741126 | 0.009015519 | 0.761093403 | IVW | ARFIP1 | 1 |
| 0.227846872 | 0.33903047 | 0.501549153 | IVW | ARHGEF25 | 0.994044169 |
| -0.073448837 | 0.534092223 | 0.890619084 | IVW | MYOM2 | 1 |
| -0.047160941 | 0.387191934 | 0.903055443 | Wald ratio | POLI | 1 |
| 0.102950503 | 0.232945862 | 0.658525143 | Wald ratio | HMHA1 | 1 |
| 0.021053084 | 0.146117634 | 0.885434839 | IVW | EIF1AD | 1 |
| -0.028199195 | 0.036431493 | 0.438911007 | IVW | SWAP70 | 0.97738713 |
| 0.463594771 | 0.745135879 | 0.533835754 | Wald ratio | DVL2 | 0.994044169 |
| 0.148610051 | 0.366870873 | 0.685422718 | Wald ratio | ABLIM3 | 1 |
| 0.296320243 | 0.103719003 | 0.004277301 | IVW | UAP1 | 0.094493942 |
| -0.168967633 | 0.167568591 | 0.313286911 | IVW | REXO2 | 0.894057446 |
| 0.284727186 | 0.068533008 | 3.26E-05 | IVW | RAB31 | 0.001118392 |
| -0.212694462 | 0.568336652 | 0.708225567 | Wald ratio | RFESD | 1 |
| 0.448405664 | 0.934545789 | 0.631361551 | Wald ratio | MAD1L1 | 1 |
| 0.398835218 | 0.480271405 | 0.406291668 | Wald ratio | AP2A2 | 0.968849361 |
| 0.169277419 | 0.445810323 | 0.704162725 | Wald ratio | PPP2R5A | 1 |
| 0.029360161 | 0.236042742 | 0.9010105 | Wald ratio | NADK | 1 |
| 0.198340938 | 0.411334601 | 0.629671832 | Wald ratio | ZYX | 1 |
| 0.65668031 | 0.383456858 | 0.086799552 | Wald ratio | IFIT3 | 0.576254043 |
| 0.015805934 | 0.092185635 | 0.863863914 | IVW | PNKP | 1 |
| 0.043320463 | 0.10338783 | 0.675209331 | IVW | CA10 | 1 |
| 0.077692641 | 0.20398558 | 0.70329734 | IVW | FGFR3 | 1 |
| 0.100782221 | 0.081169409 | 0.214373803 | IVW | ELANE | 0.829192218 |
| -0.081953915 | 0.057984867 | 0.157547754 | IVW | INHBB | 0.754698922 |
| -0.187104567 | 0.081764096 | 0.022117363 | IVW | CSF1R | 0.295351212 |
| -0.011778001 | 0.082403945 | 0.886345411 | IVW | IL5RA | 1 |
| 0.055810793 | 0.082202642 | 0.497174865 | IVW | WISP1 | 0.994044169 |
| -0.531628467 | 0.0010646 | 0 | IVW | GPD1 | 0 |
| -0.03076291 | 0.07531783 | 0.682949742 | IVW | ANXA2 | 1 |
| 0.08447421 | 0.063428373 | 0.182924345 | IVW | FCN2 | 0.795137666 |
| -0.665432203 | 0.643194915 | 0.300868289 | Wald ratio | PAK4 | 0.894057446 |
| -0.038789014 | 0.052899105 | 0.463397374 | IVW | PRTN3 | 0.994044169 |
| 0.055562687 | 0.08281838 | 0.502285504 | IVW | IL12B | 0.994044169 |
| 0.270015873 | 0.338340741 | 0.424836253 | Wald ratio | INHBA | 0.971782639 |
| -0.014849147 | 0.053497446 | 0.781344456 | IVW | FRZB | 1 |
| -0.016218011 | 0.051377571 | 0.752258119 | IVW | CCL8 | 1 |
| 0.056196521 | 0.067780571 | 0.40705075 | IVW | CROT | 0.969456681 |
| -0.051579331 | 0.145101272 | 0.722236578 | IVW | APOBEC3G | 1 |
| 0.2064237 | 0.243670929 | 0.396916385 | Wald ratio | PSMD9 | 0.962009196 |
| 0.013299822 | 0.072027545 | 0.85350426 | IVW | MCF2L | 1 |
| -0.349711306 | 0.392935236 | 0.373467326 | Wald ratio | IFI16 | 0.950951101 |
| 0.012530187 | 0.500453294 | 0.980024913 | Wald ratio | DPY30 | 1 |
| 0.087614127 | 0.100811949 | 0.384800782 | IVW | SULT1A3 | 0.957360905 |
| -0.093133811 | 0.081875067 | 0.255324621 | IVW | NUDT12 | 0.875138353 |
| -0.180280772 | 0.128497457 | 0.160619532 | IVW | GNPNAT1 | 0.756504336 |
| -0.162103245 | 0.146431312 | 0.268282761 | IVW | LAP3 | 0.880697634 |
| -0.443884123 | 0.446129805 | 0.319752654 | Wald ratio | KPNA6 | 0.901121115 |
| 0.90461731 | 0.099294655 | 8.21E-20 | IVW | HSD17B14 | 6.86E-18 |
| -0.273240349 | 0.73469863 | 0.709961297 | Wald ratio | TTL | 1 |
| -0.325388686 | 0.520948905 | 0.532228579 | Wald ratio | RGS18 | 0.994044169 |
| 0.054805823 | 0.044221127 | 0.215212814 | IVW | CRYZ | 0.829192218 |
| -0.40242612 | 0.011722879 | 2.97E-258 | IVW | LANCL1 | 9.52E-256 |
| -0.017255922 | 0.070915605 | 0.807749465 | IVW | NMRAL1 | 1 |
| 0.444406677 | 1.332246581 | 0.738698856 | Wald ratio | NSF | 1 |
| 0.217605818 | 0.098416804 | 0.027031429 | IVW | ADSSL1 | 0.318738692 |
| 0.23428627 | 0.038328482 | 9.80E-10 | IVW | GNMT | 5.23E-08 |
| 0.217149171 | 0.12960357 | 0.093838607 | IVW | TNFAIP3 | 0.597375438 |
| -0.075052787 | 0.107046358 | 0.483225535 | IVW | DHX58 | 0.994044169 |
| -0.271895031 | 0.070850384 | 0.000124246 | IVW | BDNF | 0.004047456 |
| -0.042058896 | 0.044530076 | 0.344911454 | IVW | IL1RAP | 0.936346055 |
| 0.070358778 | 0.081122591 | 0.385769861 | IVW | IL15RA | 0.957360905 |
| 0.069306082 | 0.053468866 | 0.194908274 | IVW | MAGI2 | 0.807701915 |
| -0.236131091 | 0.132781496 | 0.075347482 | IVW | PKP2 | 0.550638249 |
| -0.018236888 | 0.103133819 | 0.859643941 | IVW | CST4 | 1 |
| -0.058193764 | 0.055056759 | 0.290521784 | IVW | IL18R1 | 0.884165194 |
| 0.190027725 | 0.379611855 | 0.616663732 | Wald ratio | IGFBP6 | 1 |
| -0.061193989 | 0.206247073 | 0.76669377 | Wald ratio | DEF6 | 1 |
| -0.064296173 | 0.07292489 | 0.377951691 | IVW | CBR3 | 0.953842685 |
| -0.347340637 | 0.571762948 | 0.543525355 | Wald ratio | HBEGF | 0.994044169 |
| 0.084697665 | 0.079032294 | 0.283861873 | IVW | C1QC | 0.882777802 |
| -0.030726525 | 0.064979636 | 0.636310279 | IVW | CNTFR | 1 |
| -0.296264815 | 0.603742593 | 0.623628893 | Wald ratio | RAP1GDS1 | 1 |
| -0.0984029 | 0.067094167 | 0.142474375 | IVW | MTHFS | 0.718729001 |
| -0.202377856 | 0.366799649 | 0.581126841 | Wald ratio | THBS2 | 1 |
| -0.968574751 | 0.523272425 | 0.064170243 | Wald ratio | RELT | 0.509649614 |
| 0.188186263 | 0.174173458 | 0.279940433 | Wald ratio | PIANP | 0.880697634 |
| -0.065883394 | 0.284939961 | 0.81714505 | IVW | S100A4 | 1 |
| -0.004951698 | 0.069660849 | 0.943331746 | IVW | C10orf54 | 1 |
| -0.357975573 | 0.327133222 | 0.273831886 | IVW | EFNB2 | 0.880697634 |
| -0.079792339 | 0.08085715 | 0.323725517 | IVW | IL1R2 | 0.906997731 |
| -0.036961352 | 0.131331946 | 0.778377092 | IVW | AMIGO2 | 1 |
| -0.129093182 | 0.075633525 | 0.087854606 | IVW | ISG15 | 0.578275865 |
| -0.193880744 | 1.101881838 | 0.860329919 | Wald ratio | EFNA3 | 1 |
| 0.060463536 | 0.185945856 | 0.745054359 | Wald ratio | YWHAB | 1 |
| 0.262681158 | 0.259386585 | 0.31120281 | IVW | ANXA5 | 0.894057446 |
| 1.244495299 | 0.792281049 | 0.116234911 | Wald ratio | ANXA7 | 0.662918394 |
| -0.282818713 | 0.442649123 | 0.522873013 | Wald ratio | FOXJ2 | 0.994044169 |
| 0.009059791 | 0.339449835 | 0.978707284 | Wald ratio | HEXIM2 | 1 |
| -0.058971971 | 0.386016529 | 0.878579195 | IVW | RBP7 | 1 |
| -0.243084892 | 0.143525098 | 0.090327028 | IVW | DOK2 | 0.584540565 |
| -0.123118349 | 0.205937346 | 0.549944217 | IVW | RAB6B | 0.994044169 |
| 0.119788538 | 0.202980274 | 0.555090986 | IVW | PREP | 0.994044169 |
| 0.52083217 | 0.285220408 | 0.067839856 | IVW | VOPP1 | 0.524091257 |
| 0.368240741 | 0.504234568 | 0.465208966 | Wald ratio | SUMO3 | 0.994044169 |
| -0.072852832 | 0.039277389 | 0.063620678 | IVW | HRSP12 | 0.507381503 |
| -0.055120885 | 0.050594058 | 0.275945139 | IVW | DNAJC17 | 0.880697634 |
| 0.041082512 | 0.075162646 | 0.584666281 | IVW | CAPN2 | 1 |
| -0.342381085 | 1.266739917 | 0.78694083 | Wald ratio | PTPN7 | 1 |
| 0.00669 | 0.084713128 | 0.937054566 | IVW | C8G | 1 |
| -0.039072182 | 0.026476788 | 0.140020529 | IVW | CST6 | 0.711956232 |
| 0.153119488 | 0.044523623 | 0.000583734 | IVW | CRLF1 | 0.016259957 |
| -0.68034375 | 0.633693015 | 0.282994264 | Wald ratio | NETO1 | 0.882777802 |
| 0.295792657 | 0.616548596 | 0.63140117 | Wald ratio | MESDC2 | 1 |
| -0.008497399 | 0.289594412 | 0.976591501 | Wald ratio | CCDC134 | 1 |
| -0.132103553 | 0.094417611 | 0.161770698 | IVW | REG3A | 0.756504336 |
| -0.30317068 | 0.323029212 | 0.347975171 | IVW | SCGN | 0.936346055 |
| 0.047164449 | 0.10888122 | 0.6648888 | IVW | VWC2 | 1 |
| 0.011225888 | 0.09408582 | 0.90502548 | IVW | TXNL4B | 1 |
| -0.146682928 | 0.314736956 | 0.641180235 | IVW | CRADD | 1 |
| -0.485083079 | 0.067768743 | 8.19E-13 | IVW | FTL | 5.62E-11 |
| -0.174363435 | 0.242316616 | 0.471790633 | IVW | GBP1 | 0.994044169 |
| 0.085073129 | 0.122442177 | 0.487179088 | Wald ratio | SDF2 | 0.994044169 |
| 0.261110642 | 0.616239865 | 0.671773003 | Wald ratio | SELM | 1 |
| 0.172494798 | 0.280074772 | 0.53796819 | Wald ratio | CFL2 | 0.994044169 |
| -0.036135574 | 0.037638032 | 0.337014265 | IVW | KNG1 | 0.927192714 |
| 0.047819892 | 0.053782857 | 0.373933813 | IVW | APOA5 | 0.950951101 |
| -0.02051349 | 0.33941523 | 0.951807 | IVW | APOC1 | 1 |
| -0.04325204 | 0.089194219 | 0.627733258 | IVW | BPIFB1 | 1 |
| 0.045387627 | 0.152448315 | 0.765913298 | IVW | BMPER | 1 |
| -0.0654401 | 0.586022709 | 0.911086495 | IVW | BOLA1 | 1 |
| -1.062743326 | 0.589601643 | 0.07147041 | Wald ratio | BRD2 | 0.538690697 |
| 0.043323519 | 0.298795274 | 0.884715616 | IVW | CPB1 | 1 |
| -0.074006152 | 0.083426883 | 0.375036907 | IVW | CTSE | 0.950951101 |
| 0.051831603 | 0.075895661 | 0.494649583 | IVW | KL | 0.994044169 |
| 0.007977004 | 0.082045288 | 0.922546255 | IVW | FABP2 | 1 |
| -0.029247754 | 0.115184184 | 0.799556115 | IVW | NRP2 | 1 |
| 0.015244024 | 0.05037089 | 0.762167583 | IVW | FCGR3A | 1 |
| 0.011321734 | 0.103204013 | 0.912645351 | IVW | GAS6 | 1 |
| -0.368971845 | 0.188548085 | 0.050357919 | IVW | UNC5B | 0.446027284 |
| -0.016881946 | 0.046636117 | 0.717357287 | IVW | GSTM1 | 1 |
| 0.339546142 | 0.505709531 | 0.501949672 | Wald ratio | GFER | 0.994044169 |
| -0.001105264 | 0.540716029 | 0.998369065 | Wald ratio | LDHA | 1 |
| 0.01077642 | 0.214209202 | 0.959877011 | Wald ratio | SERPINB5 | 1 |
| -0.379511605 | 0.362538537 | 0.29518386 | IVW | NEU1 | 0.893454139 |
| -0.538147745 | 0.275580793 | 0.050846063 | IVW | LOXL3 | 0.447492971 |
| -0.032664989 | 0.120799608 | 0.786847604 | IVW | PNP | 1 |
| -0.670979243 | 0.34798779 | 0.053834118 | Wald ratio | PCSK2 | 0.461915959 |
| -0.066746163 | 0.142880497 | 0.640394873 | IVW | GM2A | 1 |
| 0.018245017 | 0.219060351 | 0.933622839 | IVW | SERPINB4 | 1 |
| -0.2224546 | 0.129289332 | 0.085324312 | IVW | SORD | 0.573403243 |
| 0.026006321 | 0.147763329 | 0.860294073 | IVW | TIMD4 | 1 |
| -0.043370986 | 0.077182944 | 0.574167471 | IVW | NT5E | 1 |
| -0.018829186 | 0.097007344 | 0.846097091 | IVW | AMBP | 1 |
| -0.094259701 | 0.070303356 | 0.179999437 | IVW | CD8A | 0.79153876 |
| -0.001574986 | 0.11392716 | 0.988969998 | IVW | COL9A1 | 1 |
| -0.000244648 | 0.057300406 | 0.996593388 | IVW | CFHR1 | 1 |
| 0.080293828 | 0.086273971 | 0.352016911 | IVW | HEXB | 0.936346055 |
| -0.014799129 | 0.039603125 | 0.708638027 | IVW | PNLIPRP2 | 1 |
| 0.028836531 | 0.053825233 | 0.592135686 | IVW | LRP11 | 1 |
| -0.042739021 | 0.163079236 | 0.793263479 | IVW | PLTP | 1 |
| -0.020021944 | 0.052490633 | 0.702877961 | IVW | REG3G | 1 |
| 0.024498705 | 0.04940324 | 0.619970132 | IVW | VNN2 | 1 |
| 0.862607927 | 0.850396365 | 0.310411076 | Wald ratio | CAMP | 0.894057446 |
| -0.064219244 | 0.083166675 | 0.440010672 | IVW | A2ML1 | 0.97738713 |
| 0.004842239 | 0.074486545 | 0.948167441 | IVW | AGRN | 1 |
| 0.003861392 | 0.054360228 | 0.943371175 | IVW | AOC1 | 1 |
| -1.24937276 | 0.564774194 | 0.026955382 | Wald ratio | CES1 | 0.318738692 |
| 0.095784741 | 0.183956113 | 0.602580346 | IVW | CD248 | 1 |
| 0.552440496 | 0.45251157 | 0.222149727 | Wald ratio | FGFBP1 | 0.84049562 |
| -0.018692503 | 0.034199255 | 0.584669846 | IVW | FOLR3 | 1 |
| -0.041538915 | 0.05899957 | 0.481398802 | IVW | ATRN | 0.994044169 |
| -0.048144021 | 0.111917934 | 0.667070145 | IVW | LEFTY2 | 1 |
| 0.034385594 | 0.054936051 | 0.531367171 | IVW | LEFTY2 | 0.994044169 |
| 0.913281709 | 0.587101469 | 0.119808643 | Wald ratio | LRP12 | 0.669395967 |
| 0.010226392 | 0.019528854 | 0.600518168 | IVW | NAGLU | 1 |
| 0.017244385 | 0.034190802 | 0.614010234 | IVW | NPTXR | 1 |
| -0.18897229 | 0.163285045 | 0.247143541 | IVW | PRSS8 | 0.870512524 |
| -0.016433573 | 0.064648134 | 0.79934084 | IVW | BCHE | 1 |
| -0.041556672 | 0.070247206 | 0.554133492 | IVW | SAA1 | 0.994044169 |
| -0.004587337 | 0.023786911 | 0.847075405 | IVW | SAA4 | 1 |
| -0.108483963 | 0.039451215 | 0.005962698 | IVW | CLSTN1 | 0.119378183 |
| 0.009498523 | 0.004706525 | 0.043574535 | IVW | GLIPR2 | 0.413729933 |
| 0.145978932 | 0.128811946 | 0.257100174 | IVW | ALAD | 0.875138353 |
| 0.179055073 | 0.02509558 | 9.68E-13 | IVW | ADH1C | 6.20E-11 |
| 0.28171587 | 0.056838015 | 7.18E-07 | IVW | GSS | 3.29E-05 |
| 0.44565108 | 0.256018795 | 0.081736751 | IVW | DLD | 0.563075399 |
| 0.105324635 | 0.298692067 | 0.724373748 | Wald ratio | CSRP1 | 1 |
| 0.161819835 | 0.0591223 | 0.00619956 | IVW | EPHB4 | 0.122840771 |
| -0.014782637 | 0.022743003 | 0.515701156 | IVW | MSR1 | 0.994044169 |
| 0.139622913 | 0.069221131 | 0.043689647 | IVW | PRSS27 | 0.413729933 |
| -0.04869866 | 0.519097442 | 0.92525683 | Wald ratio | SLITRK1 | 1 |
| 0.384568192 | 0.514932288 | 0.455164646 | IVW | VIM | 0.992816625 |
| 0.01883099 | 0.30812848 | 0.951268351 | IVW | CKMT1A | 1 |
| -0.08246997 | 0.063448789 | 0.193673665 | IVW | KLK14 | 0.807590253 |
| -0.040533624 | 0.061804219 | 0.51192765 | IVW | AMY2B | 0.994044169 |
| 0.109589189 | 0.044264314 | 0.013294138 | IVW | ENPEP | 0.212927779 |
| -0.120354162 | 0.087346537 | 0.168236396 | IVW | ANTXR2 | 0.768053097 |
| -0.012232565 | 0.039220836 | 0.75512453 | IVW | TCN2 | 1 |
| 0.298676207 | 0.133644052 | 0.025426315 | IVW | GUSB | 0.315286311 |
| -0.40282377 | 0.099762357 | 5.39E-05 | IVW | MUC16 | 0.001787697 |
| 0.067990847 | 0.135481446 | 0.615775723 | IVW | CR2 | 1 |
| -0.071135114 | 0.11952331 | 0.551738645 | IVW | NCAN | 0.994044169 |
| -0.124778374 | 0.059776501 | 0.036850625 | IVW | RNASE3 | 0.376738833 |
| -0.061754388 | 0.385893069 | 0.872857605 | IVW | ENPP6 | 1 |
| -0.061875925 | 0.071444644 | 0.386452833 | IVW | FCN1 | 0.957360905 |
| -0.05318769 | 0.058395342 | 0.362389821 | IVW | FCRLB | 0.93822612 |
| 0.020907651 | 0.03214413 | 0.515411598 | IVW | CFHR2 | 0.994044169 |
| -0.187599244 | 0.143493808 | 0.191087686 | IVW | FBLN5 | 0.804842439 |
| 0.257525661 | 0.149588793 | 0.085149766 | IVW | FOLR2 | 0.573403243 |
| 0.202423416 | 0.493765716 | 0.681836445 | IVW | GC | 1 |
| -1.3785138 | 3.091991507 | 0.655717427 | Wald ratio | GPX1 | 1 |
| -0.488304999 | 0.021235074 | 5.21E-117 | IVW | HTRA1 | 1.25E-114 |
| 0.065610971 | 0.346964571 | 0.850014507 | Wald ratio | HEXIM1 | 1 |
| 0.034290133 | 0.041901623 | 0.41315818 | IVW | IL6R | 0.971782639 |
| 0.110876078 | 0.327412716 | 0.734878562 | Wald ratio | ITGA2 | 1 |
| -0.079200385 | 0.144321826 | 0.583159034 | IVW | MAPK9 | 1 |
| 0.161945233 | 0.141104105 | 0.251092279 | IVW | PKLR | 0.870512524 |
| -0.160954098 | 0.143755926 | 0.262869535 | IVW | LAP3 | 0.879481929 |
| -0.043263244 | 0.063290811 | 0.49425128 | IVW | PNLIP | 0.994044169 |
| -0.029475289 | 0.0469825 | 0.530418434 | IVW | LILRA2 | 0.994044169 |
| -0.010253579 | 0.037029853 | 0.781856634 | IVW | LILRB3 | 1 |
| -0.03651194 | 0.140345785 | 0.794742661 | IVW | NLGN1 | 1 |
| -0.094744964 | 0.089110847 | 0.287679506 | IVW | HSPG2 | 0.884165194 |
| -0.016123175 | 0.074539558 | 0.828751102 | IVW | RBP4 | 1 |
| 0.016934515 | 0.064325869 | 0.792349142 | IVW | SMOC2 | 1 |
| -0.033007825 | 0.455809077 | 0.942270931 | Wald ratio | SORCS1 | 1 |
| 0.122323885 | 0.045832624 | 0.00760942 | IVW | TAGLN | 0.144804998 |
| -0.352002649 | 0.672660927 | 0.600766343 | Wald ratio | TMEFF1 | 1 |
| -0.073358705 | 0.066094908 | 0.267042338 | IVW | BTD | 0.880697634 |
| -0.063215088 | 0.19052056 | 0.740038831 | IVW | COL10A1 | 1 |
| 0.031425897 | 0.039523264 | 0.426541356 | IVW | INHBC | 0.971782639 |
| -0.085644511 | 0.568672655 | 0.880287919 | Wald ratio | GPT | 1 |
| -0.057136294 | 0.072722723 | 0.432058978 | IVW | FLT4 | 0.971955213 |
| 0.149871084 | 0.248831388 | 0.546974624 | IVW | OLR1 | 0.994044169 |
| 0.000661649 | 0.032175987 | 0.983593908 | IVW | CFHR5 | 1 |
| -0.000882095 | 0.088740999 | 0.992069072 | IVW | IGF2R | 1 |
| 0.022666476 | 0.067586835 | 0.737347346 | IVW | NID2 | 1 |
| -0.80827911 | 0.498037671 | 0.104604871 | Wald ratio | WIF1 | 0.626325739 |
| 0.365136364 | 0.704679144 | 0.604346821 | Wald ratio | GRAP2 | 1 |
| -0.076282784 | 0.264978022 | 0.773435965 | Wald ratio | TEC | 1 |
| -0.000417934 | 0.140740628 | 0.997630656 | IVW | EPHA4 | 1 |
| 0.005651102 | 0.553113019 | 0.991848233 | Wald ratio | LGR5 | 1 |
| 0.065225485 | 0.093379941 | 0.484867263 | IVW | TREM2 | 0.994044169 |
| -0.280867188 | 0.372824219 | 0.45123952 | Wald ratio | BPIFA2 | 0.988919449 |
| 0.167648967 | 0.837889358 | 0.841414221 | IVW | LGR4 | 1 |
| 0.132097341 | 0.360697844 | 0.71419559 | IVW | UNC5D | 1 |
| -0.060978295 | 0.553887597 | 0.91233684 | Wald ratio | DKKL1 | 1 |
| 0.207006042 | 0.150463535 | 0.168887039 | IVW | ACVRL1 | 0.76919642 |
| -0.11772476 | 0.115368287 | 0.307526632 | IVW | PACAP | 0.894057446 |
| 0.091034348 | 0.154736409 | 0.556318289 | IVW | TLR1 | 0.994210735 |
| -0.049614138 | 0.082365236 | 0.546929562 | IVW | MYOC | 0.994044169 |
| -0.139136813 | 0.086721817 | 0.108625074 | IVW | A1BG | 0.637742829 |
| 0.054880764 | 0.067947063 | 0.41926397 | IVW | NUDT2 | 0.971782639 |
| -0.196960786 | 0.115175097 | 0.08724762 | IVW | FAIM | 0.576254043 |
| 0.030447029 | 0.3745689 | 0.935214887 | Wald ratio | GLRX3 | 1 |
| 0.485886768 | 0.404048346 | 0.229152011 | Wald ratio | GPN1 | 0.850634127 |
| -0.174600478 | 0.154180383 | 0.257448281 | Wald ratio | C1QTNF9 | 0.875138353 |
| -0.296701199 | 0.251878046 | 0.238814238 | IVW | AKR1B1 | 0.857406393 |
| -0.305490169 | 0.448702495 | 0.495978995 | IVW | GSN | 0.994044169 |
| 0.027685513 | 0.049358914 | 0.574864354 | IVW | KIRREL2 | 1 |
| 0.269134612 | 0.150634558 | 0.073990448 | IVW | LRP10 | 0.550638249 |
| 0.137084079 | 0.116331951 | 0.238642288 | IVW | CDH17 | 0.857406393 |
| 0.290558697 | 0.199387469 | 0.145045573 | Wald ratio | RSPO1 | 0.72598331 |
| -0.009179166 | 0.090731987 | 0.919417161 | IVW | ENO3 | 1 |
| -0.002608102 | 0.060774655 | 0.965769846 | IVW | LY75 | 1 |
| -0.109564588 | 0.12910159 | 0.396065195 | IVW | APOA1BP | 0.962009196 |
| -0.124908186 | 0.32699115 | 0.702466885 | Wald ratio | NPPB | 1 |
| 0.038289179 | 0.069049376 | 0.579223818 | IVW | COL6A2 | 1 |
| 0.475660927 | 0.396574834 | 0.230363558 | Wald ratio | HDGF | 0.850634127 |
| -0.089185284 | 0.05393432 | 0.098210727 | IVW | LECT2 | 0.614808874 |
| -0.081349587 | 0.0847654 | 0.337204842 | IVW | REG1B | 0.927192714 |
| 0.032424566 | 0.139549 | 0.816264199 | IVW | SCUBE3 | 1 |
| 0.029262459 | 0.03151866 | 0.353191315 | IVW | ENGASE | 0.936346055 |
| 0.095533239 | 0.22328579 | 0.668758981 | IVW | DEFA5 | 1 |
| 0.053937877 | 0.044321371 | 0.223614791 | IVW | SIGLEC5 | 0.841071679 |
| -0.019185721 | 0.106025906 | 0.856404353 | IVW | PDE5A | 1 |
| -0.067999334 | 0.243168211 | 0.779754424 | IVW | NME4 | 1 |
| 0.156564143 | 0.014295333 | 6.49E-28 | IVW | CDCP1 | 6.93E-26 |
| -0.052196104 | 0.044358151 | 0.239316462 | IVW | APOL3 | 0.857406393 |
| 0.07131077 | 0.046472968 | 0.12491727 | IVW | ATXN3 | 0.67523297 |
| -0.023819011 | 0.03763691 | 0.526823056 | IVW | COL6A1 | 0.994044169 |
| 0.096362963 | 0.265893519 | 0.717044283 | Wald ratio | CFHR3 | 1 |
| -0.05716387 | 0.301879775 | 0.849810892 | Wald ratio | SCO2 | 1 |
| -0.042464481 | 0.109288582 | 0.697606335 | IVW | NRK1 | 1 |
| -0.250453934 | 0.19566577 | 0.200541993 | Wald ratio | MAPRE2 | 0.811456233 |
| 0.056558438 | 0.035272869 | 0.108834364 | IVW | GSTZ1 | 0.637742829 |
| -0.050175687 | 0.10790672 | 0.641937683 | IVW | PHPT1 | 1 |
| 0.066930048 | 0.063312945 | 0.290452012 | IVW | ADAMTSL1 | 0.884165194 |
| -0.07285843 | 0.194222559 | 0.707564852 | IVW | ENPP2 | 1 |
| -0.061543128 | 0.034078085 | 0.070927016 | IVW | MDGA1 | 0.536699701 |
| 0.751379715 | 0.385012573 | 0.050989053 | Wald ratio | OMG | 0.447492971 |
| -0.104116161 | 0.047182353 | 0.027336711 | IVW | RNASET2 | 0.320372913 |
| 0.133970073 | 0.0633611 | 0.034481976 | IVW | CD14 | 0.360186732 |
| -0.094002999 | 0.17430234 | 0.589672951 | Wald ratio | SEMA4A | 1 |
| -0.524464012 | 0.296325646 | 0.076745347 | IVW | SLITRK6 | 0.552451523 |
| 0.080789303 | 0.059073846 | 0.17143779 | IVW | TLR3 | 0.774504164 |
| 0.169349531 | 0.211021236 | 0.422250118 | IVW | DBI | 0.971782639 |
| -0.066667384 | 0.043272279 | 0.123402289 | IVW | GSTA1 | 0.67523297 |
| 0.045840335 | 0.098759645 | 0.642532692 | IVW | PDGFD | 1 |
| -0.301943933 | 0.19694012 | 0.125232412 | IVW | BLVRB | 0.67523297 |
| -0.298700699 | 0.430167832 | 0.487442825 | Wald ratio | IRF3 | 0.994044169 |
| 0.004900853 | 0.029799711 | 0.869369224 | IVW | KIR2DL3 | 1 |
| 0.167764551 | 0.030826468 | 5.26E-08 | IVW | DCLK1 | 2.66E-06 |
| -0.47710976 | 0.068373532 | 2.99E-12 | IVW | DDOST | 1.86E-10 |
| 0.008318053 | 0.119001064 | 0.944274062 | IVW | ANXA4 | 1 |
| -0.025227849 | 0.251849354 | 0.920209049 | IVW | CALCB | 1 |
| -1.448799232 | 0.618001921 | 0.019061398 | Wald ratio | BAG1 | 0.271377831 |
| 0.145357602 | 0.159854102 | 0.363184305 | IVW | TCL1A | 0.93822612 |
| -0.154501666 | 0.059734432 | 0.009696296 | IVW | OGN | 0.170975056 |
| 0.090796692 | 0.044673973 | 0.042110214 | IVW | LCP1 | 0.41084178 |
| 0.082592145 | 0.17148233 | 0.630064211 | IVW | GUK1 | 1 |
| 0.261736864 | 0.003616508 | 0 | IVW | CNPY3 | 0 |
| -0.18915206 | 0.081380748 | 0.020110218 | IVW | BDH2 | 0.284204703 |
| 0.032401388 | 0.060364873 | 0.591434101 | IVW | KREMEN1 | 1 |
| -0.097850239 | 0.252418596 | 0.698274563 | IVW | ADPRHL2 | 1 |
| -0.013943459 | 0.144315743 | 0.923029979 | Wald ratio | ACADM | 1 |
| -0.080957977 | 0.112136182 | 0.470318308 | IVW | ACAT2 | 0.994044169 |
| -0.048822792 | 0.067712358 | 0.470889695 | IVW | SNPH | 0.994044169 |
| 0.103936179 | 0.307364995 | 0.735248421 | Wald ratio | ZADH2 | 1 |
| -0.087706347 | 0.061895464 | 0.156480683 | IVW | CHMP2B | 0.754698922 |
| 0.149179306 | 0.452818123 | 0.741818292 | Wald ratio | SEPSECS | 1 |
| -0.582099792 | 0.596471933 | 0.329111669 | Wald ratio | ABHD10 | 0.916742939 |
| -0.059346523 | 0.085994011 | 0.490116272 | IVW | AKR1C3 | 0.994044169 |
| 0.097659042 | 0.13599144 | 0.472679359 | IVW | STARD5 | 0.994044169 |
| 0.129886986 | 0.191665851 | 0.497978342 | Wald ratio | PFKM | 0.994044169 |
| -0.066193381 | 0.06453841 | 0.305059799 | IVW | RBM17 | 0.894057446 |
| -0.33083113 | 0.44402842 | 0.456230935 | Wald ratio | ADH1A | 0.993064392 |
| -1.720106198 | 0.229603354 | 6.80E-14 | IVW | HMOX1 | 4.84E-12 |
| 0.008529221 | 0.051430857 | 0.86828398 | IVW | ACADSB | 1 |
| 0.346703642 | 0.542127483 | 0.522481979 | Wald ratio | PMM1 | 0.994044169 |
| -0.113918766 | 0.457513854 | 0.803364474 | Wald ratio | CETN3 | 1 |
| 0.290503079 | 0.577497284 | 0.614937556 | Wald ratio | TES | 1 |
| -0.689958478 | 0.499648212 | 0.167313242 | Wald ratio | ETFA | 0.767484151 |
| -0.14169794 | 0.1103372 | 0.199062765 | IVW | SFRP4 | 0.810778384 |
| 0.088568058 | 0.071848369 | 0.217684733 | IVW | CP | 0.832728025 |
| -0.24453281 | 0.114253512 | 0.032333345 | IVW | GOLM1 | 0.349127466 |
| 0.282106456 | 0.221459482 | 0.20271611 | IVW | MX1 | 0.816814179 |
| 0.076439159 | 0.104887086 | 0.466138882 | IVW | SH3BGRL3 | 0.994044169 |
| -0.193180404 | 0.179550201 | 0.281966147 | IVW | ANXA11 | 0.882777802 |
| -0.000117914 | 0.587146797 | 0.999839764 | Wald ratio | RAB21 | 1 |
| 0.459288145 | 0.126920475 | 0.000296077 | IVW | HSPA13 | 0.008493445 |
| 0.021634772 | 0.185433938 | 0.907120734 | IVW | GIF | 1 |
| 0.349011149 | 0.479208112 | 0.466425006 | IVW | ACOT13 | 0.994044169 |
| -0.177453922 | 0.220648134 | 0.42125873 | Wald ratio | CD160 | 0.971782639 |
| -0.014445209 | 0.076006118 | 0.849267411 | IVW | EPHB1 | 1 |
| 0.157634958 | 0.170249141 | 0.354494049 | IVW | CD46 | 0.936346055 |
| -0.030413967 | 0.248004424 | 0.902396314 | Wald ratio | APOA4 | 1 |
| 0.127028373 | 0.471762926 | 0.787727184 | Wald ratio | TBCB | 1 |
| -0.078967709 | 0.062109007 | 0.203573117 | IVW | TPP1 | 0.816842446 |
| 0.06025851 | 0.055029358 | 0.273505778 | IVW | BTN3A3 | 0.880697634 |
| -0.030752368 | 0.289036504 | 0.915268056 | IVW | PSME2 | 1 |
| -0.249181013 | 0.241385637 | 0.301934278 | Wald ratio | OVCA2 | 0.894057446 |
| -0.993667832 | 0.646524476 | 0.124308569 | Wald ratio | WBP2 | 0.67523297 |
| 0.087092499 | 0.031092465 | 0.005093181 | IVW | UGT1A1 | 0.10788087 |
| 0.62149537 | 0.484092593 | 0.199199436 | Wald ratio | PPP1R1A | 0.810778384 |
| 0.782109228 | 0.999171375 | 0.433769329 | Wald ratio | IDI1 | 0.972817562 |
| 0.730280679 | 0.375543285 | 0.051823394 | IVW | FKBP4 | 0.452469709 |
| -0.276729496 | 0.415671942 | 0.505577213 | Wald ratio | WWOX | 0.994044169 |
| -0.108979568 | 0.1442397 | 0.449922107 | IVW | SAR1A | 0.987157865 |
| 0.81766291 | 0.742947901 | 0.27108576 | Wald ratio | CHMP2A | 0.880697634 |
| -0.142766432 | 0.254602308 | 0.574972828 | IVW | IVD | 1 |
| 0.023139142 | 0.258996904 | 0.928810606 | Wald ratio | CRNN | 1 |
| -0.024672564 | 0.408792264 | 0.951873078 | Wald ratio | HADH | 1 |
| -0.105402418 | 0.102264418 | 0.302688487 | IVW | FIS1 | 0.894057446 |
| 0.112966088 | 0.053822611 | 0.035829305 | IVW | TP53I3 | 0.370010771 |
| -0.001176354 | 0.167221065 | 0.994387148 | Wald ratio | CRYBB1 | 1 |
| 0.022405893 | 0.15998572 | 0.888621017 | IVW | UGDH | 1 |
| -0.074838062 | 0.134941468 | 0.579170704 | IVW | DCTD | 1 |
| -0.088085793 | 0.317690529 | 0.781573422 | Wald ratio | DCXR | 1 |
| -0.466662465 | 0.401654062 | 0.245295699 | Wald ratio | NUDT5 | 0.870512524 |
| -0.568780561 | 0.286740481 | 0.047299615 | Wald ratio | RHOC | 0.430852421 |
| -0.079792827 | 0.122850309 | 0.516007088 | IVW | SNUPN | 0.994044169 |
| 0.159193455 | 0.075082709 | 0.033985708 | IVW | NCF1 | 0.358904016 |
| 0.156954657 | 0.349498775 | 0.653370341 | Wald ratio | PCNP | 1 |
| -0.263510316 | 0.393049519 | 0.502586752 | Wald ratio | NAPG | 0.994044169 |
| 0.081836788 | 0.370920984 | 0.82537951 | Wald ratio | PECR | 1 |
| -0.093523663 | 0.085904371 | 0.276288384 | IVW | SDSL | 0.880697634 |
| -1.2836875 | 0.579469758 | 0.026740883 | Wald ratio | MAP1LC3A | 0.318738692 |
| -0.046034554 | 0.106430477 | 0.665355025 | IVW | ACAA1 | 1 |
| -0.015351262 | 0.100892974 | 0.87906553 | IVW | MMAB | 1 |
| -0.098889454 | 0.312061626 | 0.751326475 | Wald ratio | RCL | 1 |
| -0.270585404 | 0.362404503 | 0.455281387 | Wald ratio | GGPS1 | 0.992816625 |
| 0.350221843 | 0.642020478 | 0.585410031 | Wald ratio | ECHS1 | 1 |
| 0.12040702 | 0.246900862 | 0.62578109 | Wald ratio | HPCAL1 | 1 |
| 0.204618882 | 0.084014029 | 0.014869897 | IVW | PMM2 | 0.230483396 |
| -0.322378638 | 0.192768988 | 0.094453873 | IVW | PGLS | 0.599143047 |
| 0.125681452 | 0.435529896 | 0.77290942 | IVW | NANS | 1 |
| 0.90825595 | 0.014373017 | 0 | IVW | NIT2 | 0 |
| -0.351466269 | 0.158788496 | 0.026868427 | IVW | BPHL | 0.318738692 |
| 0.189771635 | 0.076055743 | 0.012589854 | IVW | BPNT1 | 0.203342006 |
| -0.128457286 | 0.167260516 | 0.442482885 | IVW | FAHD1 | 0.980913617 |
| -0.573714286 | 0.454572751 | 0.206914375 | Wald ratio | NMT2 | 0.821672375 |
| 0.87589177 | 0.324915445 | 0.007022973 | Wald ratio | S100A14 | 0.136344996 |
| 0.107624121 | 0.198668069 | 0.588005674 | IVW | IDI2 | 1 |
| -0.329841341 | 0.105746092 | 0.001813537 | IVW | S100A16 | 0.044121751 |
| -0.198502439 | 0.307506341 | 0.51858827 | Wald ratio | PPCS | 0.994044169 |
| -0.171071271 | 0.295338926 | 0.562429182 | IVW | NT5M | 0.998143017 |
| -0.202026575 | 0.247744302 | 0.414806728 | IVW | IRAK4 | 0.971782639 |
| -0.12094894 | 0.146374 | 0.408633529 | IVW | AKR7A3 | 0.971782639 |
| -0.313494759 | 0.059068466 | 1.11E-07 | IVW | PDCD6IP | 5.48E-06 |
| -0.140144962 | 0.458829676 | 0.760031019 | IVW | VAT1 | 1 |
| 0.054137065 | 0.998519171 | 0.956761997 | Wald ratio | SARS | 1 |
| -0.947338419 | 0.458450763 | 0.038791598 | Wald ratio | RTN4IP1 | 0.388320059 |
| -0.210348428 | 0.608961006 | 0.729777265 | Wald ratio | TSSC4 | 1 |
| -0.249052341 | 0.368112053 | 0.49868094 | IVW | LDLRAP1 | 0.994044169 |
| 0.016793657 | 0.12505371 | 0.893172029 | IVW | ALDOB | 1 |
| -0.160695196 | 0.170086692 | 0.344769131 | Wald ratio | GATM | 0.936346055 |
| 0.580409784 | 0.518994282 | 0.263423686 | Wald ratio | CKMT2 | 0.879481929 |
| -0.762856144 | 0.386961039 | 0.048677801 | Wald ratio | HBQ1 | 0.437190343 |
| -0.157525892 | 0.185408419 | 0.395538814 | IVW | ADH6 | 0.962009196 |
| 0.464737662 | 0.40588906 | 0.252214578 | IVW | GCLM | 0.870512524 |
| -0.049518826 | 0.076296179 | 0.516316303 | IVW | THG1L | 0.994044169 |
| -0.010266171 | 0.041709306 | 0.805576786 | IVW | IL11RA | 1 |
| 0.05329023 | 0.075786577 | 0.481954888 | IVW | CNRIP1 | 0.994044169 |
| -0.204499256 | 0.114635 | 0.074437252 | IVW | SRA1 | 0.550638249 |
| 0.016661584 | 0.133829712 | 0.900920672 | IVW | SH3GLB2 | 1 |
| -0.018799773 | 0.044692868 | 0.674015288 | IVW | HEBP1 | 1 |
| -0.094350385 | 0.096951996 | 0.33047081 | IVW | PGP | 0.917801961 |
| 0.301071472 | 0.55342762 | 0.586432959 | Wald ratio | HSPC159 | 1 |
| -0.079545203 | 0.133827635 | 0.552254319 | IVW | CPOX | 0.994044169 |
| -0.705977218 | 0.357592326 | 0.048353143 | Wald ratio | CMPK1 | 0.436313333 |
| 1.235023324 | 0.786250921 | 0.116234911 | Wald ratio | ANXA7 | 0.662918394 |
| -0.074629905 | 0.178262643 | 0.67547092 | IVW | DTD2 | 1 |
| -0.047661079 | 0.033017698 | 0.148879397 | IVW | CCL15 | 0.731831718 |
| 0.146288927 | 0.858432852 | 0.864684608 | IVW | GRHPR | 1 |
| -0.009132068 | 0.328277989 | 0.977807234 | Wald ratio | PPA2 | 1 |
| -0.225239405 | 0.144745615 | 0.119683076 | IVW | SEPW1 | 0.669395967 |
| 0.220981541 | 0.131259285 | 0.092268711 | IVW | NDRG3 | 0.593111915 |
| -0.025059796 | 0.138920513 | 0.856846829 | IVW | ASRGL1 | 1 |
| -0.028920195 | 0.047329782 | 0.541175835 | IVW | RTP4 | 0.994044169 |
| -0.534130628 | 0.016893817 | 2.16E-219 | IVW | PDHX | 5.93E-217 |
| 0.208966236 | 0.107660932 | 0.05226237 | IVW | MTHFD2 | 0.452469709 |
| 0.556216386 | 0.706255701 | 0.430956069 | Wald ratio | SNX5 | 0.971955213 |
| 0.033478669 | 0.053195674 | 0.529120298 | IVW | PGM2 | 0.994044169 |
| -0.67627993 | 0.546871479 | 0.216222947 | Wald ratio | CPLX1 | 0.831161009 |
| 0.041164283 | 0.064723488 | 0.524774963 | IVW | IDH1 | 0.994044169 |
| -0.016250381 | 0.059614991 | 0.785169299 | IVW | PSMB4 | 1 |
| 0.038918665 | 0.310393854 | 0.900218937 | IVW | PSAT1 | 1 |
| 0.019549889 | 0.103523867 | 0.850214878 | IVW | DECR2 | 1 |
| -0.156134125 | 0.759097891 | 0.837037928 | IVW | ALB | 1 |
| -0.05113163 | 0.467962287 | 0.912992762 | Wald ratio | ALDH2 | 1 |
| 0.088016788 | 0.347777372 | 0.800203525 | Wald ratio | COMT | 1 |
| -0.266884024 | 0.435775148 | 0.540249883 | Wald ratio | GLRX | 0.994044169 |
| 0.193007634 | 0.552942748 | 0.727047744 | Wald ratio | ST13 | 1 |
| -0.048838403 | 0.550813688 | 0.929347417 | Wald ratio | AES | 1 |
| 0.055581634 | 0.0707923 | 0.432373417 | IVW | AKR1C4 | 0.971955213 |
| -0.774539898 | 0.618611205 | 0.210547087 | Wald ratio | AKR1D1 | 0.829192218 |
| -0.046086931 | 0.074817967 | 0.537902786 | IVW | ALKBH3 | 0.994044169 |
| 0.413353482 | 0.572441524 | 0.470240024 | Wald ratio | ARF4 | 0.994044169 |
| 1.13298893 | 0.567717712 | 0.045967605 | Wald ratio | ARL4D | 0.426810327 |
| -0.056195161 | 0.1192792 | 0.63755298 | IVW | MVD | 1 |
| -0.119104223 | 0.137202201 | 0.385343613 | IVW | RALB | 0.957360905 |
| 0.510200331 | 0.492557947 | 0.300287112 | Wald ratio | UBXN2B | 0.894057446 |
| -0.907439224 | 0.480132789 | 0.058761232 | Wald ratio | PTGES2 | 0.4811118 |
| 0.146128936 | 0.645419291 | 0.820883088 | Wald ratio | DDX19A | 1 |
| -0.038523604 | 0.072140371 | 0.593335342 | IVW | PPIC | 1 |
| -0.029574668 | 0.118405382 | 0.802761589 | IVW | ITLN1 | 1 |
| 0.001567278 | 0.08192573 | 0.98473702 | IVW | LRIG1 | 1 |
| -0.05375066 | 0.095191375 | 0.572305771 | IVW | SAA2 | 1 |
| -1.513278119 | 0.586366053 | 0.009857916 | Wald ratio | TG | 0.172244685 |
| -0.296590518 | 0.05385361 | 3.64E-08 | IVW | SERPINB13 | 1.89E-06 |
| 0.063961363 | 0.055396399 | 0.248249226 | IVW | PRSS3 | 0.870512524 |
| 0.025130629 | 0.086852579 | 0.772315004 | IVW | AIF1L | 1 |
| -0.270959883 | 0.537135029 | 0.613942421 | Wald ratio | CEACAM8 | 1 |
| -0.031802439 | 0.055900144 | 0.569413717 | IVW | COL2A1 | 1 |
| -0.16614095 | 0.40319262 | 0.680292904 | IVW | CHST4 | 1 |
| -0.084856606 | 0.049158256 | 0.084312811 | IVW | GREM1 | 0.570595858 |
| 0.237180052 | 0.446303109 | 0.595118989 | Wald ratio | COL3A1 | 1 |
| -0.008384338 | 0.071224734 | 0.906292164 | IVW | CD97 | 1 |
| -0.055394376 | 0.150208127 | 0.712288622 | IVW | CLSTN2 | 1 |
| 0.235413507 | 0.045992478 | 3.08E-07 | IVW | DNAJB4 | 1.44E-05 |
| -0.158326822 | 0.025225828 | 3.47E-10 | IVW | GBP2 | 1.90E-08 |
| 0.030136781 | 0.051550816 | 0.558814441 | IVW | GSTM4 | 0.994482737 |
| -0.340992117 | 0.297531532 | 0.251765974 | Wald ratio | HDGFRP3 | 0.870512524 |
| 0.040389631 | 0.031337544 | 0.197447606 | IVW | ITPA | 0.81076487 |
| 0.006632665 | 0.050781543 | 0.89608246 | IVW | AMY2A | 1 |
| 0.158858387 | 0.104485662 | 0.128414354 | IVW | PDE4A | 0.679923933 |
| -0.053907556 | 0.200853049 | 0.788396814 | IVW | CD68 | 1 |
| -0.036895517 | 0.473731474 | 0.93792131 | IVW | SLIT2 | 1 |
| -0.561152642 | 0.560266145 | 0.316545384 | Wald ratio | TGM4 | 0.896023899 |
| 0.070658584 | 0.499122211 | 0.887423064 | Wald ratio | TGM2 | 1 |
| -0.084459372 | 0.280377821 | 0.763235958 | Wald ratio | SH3BGRL2 | 1 |
| -0.14009447 | 0.454176651 | 0.757733773 | Wald ratio | CMBL | 1 |
| 0.315297777 | 0.160521959 | 0.04950651 | IVW | PPP1R14A | 0.440516257 |
| 0.063611904 | 0.126405568 | 0.614797957 | IVW | UBLCP1 | 1 |
| -0.062789869 | 0.058928191 | 0.286635283 | IVW | MTHFSD | 0.884165194 |
| 0.042865936 | 0.027740479 | 0.122286147 | IVW | SERPINB8 | 0.67523297 |
| -0.056739955 | 0.051491078 | 0.270488797 | IVW | NT5C3L | 0.880697634 |
| 0.152395561 | 0.162918823 | 0.349578105 | IVW | ALDH6A1 | 0.936346055 |
| -0.077304879 | 0.200849462 | 0.700319176 | IVW | CYB5R2 | 1 |
| -0.013397017 | 0.494701695 | 0.978395128 | Wald ratio | PRPSAP2 | 1 |
| 0.122280843 | 0.45461745 | 0.787948727 | IVW | MPPED2 | 1 |
| -0.125244904 | 0.087395035 | 0.151832271 | IVW | SERPINE2 | 0.740664023 |
| -0.571100698 | 0.065717065 | 3.61E-18 | IVW | USP15 | 2.90E-16 |
| -0.073553004 | 0.127698486 | 0.564622187 | IVW | ZFAND1 | 0.999034516 |
| 0.343572301 | 0.578232179 | 0.552393688 | Wald ratio | FAM49B | 0.994044169 |
| -0.428653251 | 2.089160217 | 0.837431735 | Wald ratio | RPL12 | 1 |
| -1.076205027 | 0.883814687 | 0.22334493 | Wald ratio | STAMBP | 0.841071679 |
| 0.150758267 | 0.188786203 | 0.424542036 | Wald ratio | NAP1L4 | 0.971782639 |
| 0.056863342 | 0.150244389 | 0.705079757 | Wald ratio | DTD1 | 1 |
| 0.360949868 | 0.332379068 | 0.277497361 | Wald ratio | ACAT1 | 0.880697634 |
| -0.107154157 | 0.188694108 | 0.570121684 | Wald ratio | NUDT1 | 1 |
| -0.265684492 | 0.542634581 | 0.624403149 | Wald ratio | ADK | 1 |
| 0.066494328 | 0.033798251 | 0.049138313 | IVW | SPINK4 | 0.439273662 |
| 0.230794796 | 0.104348794 | 0.026983049 | IVW | RAB1A | 0.318738692 |
| -0.024092845 | 0.081050144 | 0.766269153 | IVW | GSTT1 | 1 |
| -0.168619677 | 0.428475771 | 0.693925302 | Wald ratio | ATOX1 | 1 |
| 0.031706422 | 0.530116208 | 0.952306703 | Wald ratio | SUB1 | 1 |
| 0.380996334 | 0.268201048 | 0.155444005 | IVW | DDT | 0.752552589 |
| 0.212772781 | 0.194284615 | 0.273446452 | Wald ratio | RBP5 | 0.880697634 |
| -0.14409415 | 0.116281985 | 0.215279353 | IVW | SRGN | 0.829192218 |
| 0.033092978 | 0.052263482 | 0.526606282 | IVW | GMPR | 0.994044169 |
| 0.232308468 | 0.660635081 | 0.725105143 | Wald ratio | SDCBP2 | 1 |
| 0.40520332 | 0.609863071 | 0.506423531 | Wald ratio | ACADVL | 0.994044169 |
| -0.365804631 | 0.44770767 | 0.413893372 | Wald ratio | G3BP1 | 0.971782639 |
| -0.085790789 | 0.166038657 | 0.60537142 | IVW | HAGH | 1 |
| -0.061561489 | 0.043526817 | 0.157263647 | IVW | GSR | 0.754698922 |
| 0.102428593 | 0.137283374 | 0.455600553 | IVW | TSTD1 | 0.992816625 |
| 0.005137991 | 0.400850166 | 0.989773207 | Wald ratio | RBP1 | 1 |
| -0.025659765 | 0.001864105 | 4.13E-43 | IVW | UROD | 6.61E-41 |
| -0.11838566 | 0.166768061 | 0.477777256 | Wald ratio | VPS26A | 0.994044169 |
| -0.523799104 | 0.331180422 | 0.113738053 | Wald ratio | TXNDC12 | 0.656470082 |
| -0.068983373 | 0.499795131 | 0.890222004 | Wald ratio | LZIC | 1 |
| 0.111911452 | 0.634705521 | 0.860042335 | Wald ratio | CA12 | 1 |
| 0.101195442 | 0.045975839 | 0.027731987 | IVW | MATN3 | 0.323035635 |
| 0.231615907 | 1.295691562 | 0.858127316 | Wald ratio | BCAT2 | 1 |
| -0.213053055 | 0.298803788 | 0.475833426 | IVW | DCI | 0.994044169 |
| 0.098355706 | 0.072623593 | 0.175633773 | IVW | MDGA2 | 0.783220675 |
| -0.264373626 | 0.680725275 | 0.69774186 | Wald ratio | NNMT | 1 |
| -0.037792275 | 0.048729777 | 0.438015993 | IVW | OLFM2 | 0.97738713 |
| 0.525212963 | 0.645044444 | 0.415514601 | Wald ratio | CINP | 0.971782639 |
| 0.021460513 | 0.06742681 | 0.750273587 | IVW | VEGFA | 1 |
| -0.05104886 | 0.098205241 | 0.603190379 | IVW | GMPR2 | 1 |
| 0.289463126 | 0.213466791 | 0.175095954 | IVW | UPP1 | 0.782638193 |
| 0.061280316 | 0.162393119 | 0.705907697 | Wald ratio | HDHD3 | 1 |
| -0.082092796 | 0.269548276 | 0.760703626 | IVW | PDCD6 | 1 |
| -0.064260143 | 0.203773187 | 0.752494924 | IVW | NPL | 1 |
| 0.029680554 | 0.33323428 | 0.929027766 | IVW | DTYMK | 1 |
| -0.074044971 | 0.086375414 | 0.391308987 | IVW | PARK7 | 0.960408371 |
| 0.073538787 | 0.053246307 | 0.167246409 | IVW | CR1 | 0.767484151 |
| 0.084561895 | 0.03752844 | 0.024241817 | IVW | KLB | 0.309241723 |
| 0.017703427 | 0.074390802 | 0.811897613 | IVW | LRP4 | 1 |
| -0.282561685 | 0.331199599 | 0.393578224 | Wald ratio | PLXNA4 | 0.960408371 |
| 0.102948226 | 0.077466545 | 0.183868464 | IVW | PLXND1 | 0.795137666 |
| 0.633684245 | 0.208353364 | 0.002354871 | IVW | SEZ6L | 0.0558773 |
| -0.205896391 | 0.118889407 | 0.083303612 | IVW | DOK2 | 0.565758098 |
| -0.188204563 | 0.568460317 | 0.740585757 | Wald ratio | IGFBP5 | 1 |
| 0.006592956 | 0.03807571 | 0.862530497 | IVW | SFTPD | 1 |
| 0.080655369 | 0.505181208 | 0.873151816 | Wald ratio | JUND | 1 |
| 0.112550152 | 0.441536034 | 0.798795759 | IVW | IHH | 1 |
| 0.064523807 | 0.066961089 | 0.335245746 | IVW | TCN1 | 0.924450966 |
| 0.315626335 | 0.384130486 | 0.411267942 | Wald ratio | NT5C3A | 0.971782639 |
| 0.0038316 | 0.028481081 | 0.892982388 | IVW | PTGR1 | 1 |
| 0.272321238 | 0.341229456 | 0.424836253 | Wald ratio | INHBA | 0.971782639 |
| -0.08833139 | 0.059360347 | 0.136736707 | IVW | KNG1 | 0.700872481 |
| 0.614087866 | 0.673686192 | 0.362014082 | Wald ratio | FGL2 | 0.93822612 |
| 0.730338257 | 0.878367799 | 0.405707023 | Wald ratio | CRH | 0.968657016 |
| 0.05085942 | 0.099203178 | 0.608175643 | IVW | F11 | 1 |
| 0.171697917 | 0.589460938 | 0.770837526 | Wald ratio | CCL27 | 1 |
| -0.104996319 | 0.102472587 | 0.305538579 | IVW | COL18A1 | 0.894057446 |
| 0.143108736 | 0.310290001 | 0.644648214 | IVW | PLAT | 1 |
| -0.438483169 | 0.30314921 | 0.148057504 | IVW | TIMP2 | 0.731831718 |
| 0.225835891 | 0.376462392 | 0.548579783 | IVW | C5 | 0.994044169 |
| 0.167019952 | 0.147207579 | 0.256547986 | IVW | APOE | 0.875138353 |
| -0.297730294 | 0.374459042 | 0.426558582 | Wald ratio | BDNF | 0.971782639 |
| -0.163610628 | 0.140927776 | 0.245660715 | IVW | CXCL16 | 0.870512524 |
| -0.220722733 | 0.045556162 | 1.27E-06 | IVW | APCS | 5.53E-05 |
| 0.786650108 | 0.625825054 | 0.20876057 | Wald ratio | KIT | 0.827294466 |
| -0.036088411 | 0.046721436 | 0.439867749 | IVW | TIMP3 | 0.97738713 |
| 0.046240525 | 0.068659008 | 0.500641447 | IVW | GFRA2 | 0.994044169 |
| 0.022842044 | 0.284684651 | 0.936049316 | IVW | CCL21 | 1 |
| 0.223756892 | 0.532600908 | 0.674396973 | IVW | CFI | 1 |
| -0.069240351 | 0.060220358 | 0.250233214 | IVW | IGFBP3 | 0.870512524 |
| 0.256514523 | 0.324263189 | 0.428903789 | Wald ratio | MMP9 | 0.971955213 |
| -0.081695701 | 0.068144566 | 0.230582924 | IVW | MPO | 0.850634127 |
| -0.033295511 | 0.086384289 | 0.699915305 | IVW | ROR1 | 1 |
| -0.021550639 | 0.172723777 | 0.900706127 | IVW | VEGFA | 1 |
| -0.207619272 | 0.223467766 | 0.352848 | IVW | ANGPT2 | 0.936346055 |
| -0.107468353 | 0.141969834 | 0.449061724 | IVW | CST3 | 0.986396154 |
| -0.00116547 | 0.019403151 | 0.952103041 | IVW | TYRO3 | 1 |
| 0.006806679 | 0.075324687 | 0.927997552 | Wald ratio | EFNA4 | 1 |
| -0.870590062 | 0.467476708 | 0.062558348 | Wald ratio | EFNA5 | 0.503084287 |
| -0.054515222 | 0.108520217 | 0.61542075 | IVW | ERBB3 | 1 |
| -0.098073628 | 0.090743722 | 0.279796813 | IVW | IL6ST | 0.880697634 |
| 0.076613333 | 0.403889362 | 0.849552911 | Wald ratio | IL10RB | 1 |
| -0.13247914 | 0.385965866 | 0.731417493 | Wald ratio | IL12RB1 | 1 |
| -0.41399044 | 0.586942639 | 0.480602575 | Wald ratio | LAYN | 0.994044169 |
| -0.008295843 | 0.067339907 | 0.901953819 | IVW | MRC1 | 1 |
| 0.471132426 | 0.367587871 | 0.19995263 | Wald ratio | PRKCA | 0.810778384 |
| -0.409717058 | 0.312061287 | 0.18920391 | Wald ratio | GDI2 | 0.804634749 |
| -0.242037923 | 0.112785575 | 0.031872922 | IVW | PLAUR | 0.346511417 |
| -0.175865118 | 0.313732114 | 0.575098733 | Wald ratio | TNFRSF1A | 1 |
| 0.050336415 | 0.130975553 | 0.700741907 | IVW | NTRK3 | 1 |
| -0.098171929 | 0.554499399 | 0.859472217 | IVW | TNFRSF17 | 1 |
| 0.175167267 | 0.080236558 | 0.029025843 | IVW | EGFR | 0.332069463 |
| 0.33487619 | 0.259872222 | 0.197530699 | Wald ratio | HGF | 0.81076487 |
| -0.097814526 | 0.444396158 | 0.825788138 | Wald ratio | C3 | 1 |
| -0.023427969 | 0.020785881 | 0.259696084 | IVW | MIA | 0.875138353 |
| -0.034858825 | 0.049616181 | 0.482323965 | IVW | PLA2G2A | 0.994044169 |
| 0.016787412 | 0.315991652 | 0.957631415 | IVW | PF4 | 1 |
| -0.013666845 | 0.043180658 | 0.751620084 | IVW | PROS1 | 1 |
| 0.003758049 | 0.042031583 | 0.928755978 | IVW | CCL25 | 1 |
| -0.001233035 | 0.03459693 | 0.971569401 | IVW | MICA | 1 |
| -0.031651503 | 0.180177381 | 0.860554327 | IVW | POR | 1 |
| 0.934142675 | 0.480873885 | 0.052065245 | Wald ratio | NOV | 0.452469709 |
| 0.008159464 | 0.075104388 | 0.913486727 | IVW | SIGLEC6 | 1 |
| 0.031023789 | 0.008475086 | 0.000251638 | IVW | SIGLEC7 | 0.007328004 |
| 0.023522877 | 0.568141149 | 0.96697444 | IVW | SHH | 1 |
| -0.221630171 | 0.12163575 | 0.068442719 | IVW | APOA1 | 0.524091257 |
| -0.08280437 | 0.37620122 | 0.825788138 | Wald ratio | C3 | 1 |
| 0.20712097 | 0.306936616 | 0.499802546 | IVW | IGFBP1 | 0.994044169 |
| -0.061843297 | 0.0548134 | 0.259213746 | IVW | IL16 | 0.875138353 |
| 0.445036537 | 0.63946942 | 0.486462304 | Wald ratio | IL22 | 0.994044169 |
| 0.103361569 | 0.094740889 | 0.275276322 | IVW | LTF | 0.880697634 |
| 0.198284178 | 0.488536563 | 0.684835349 | IVW | CCL4L1 | 1 |
| 0.215130384 | 0.455480792 | 0.636702006 | Wald ratio | CCL3L1 | 1 |
| 0.02951269 | 0.11755787 | 0.801776857 | IVW | MMP7 | 1 |
| -1.195503432 | 0.407318078 | 0.00333482 | Wald ratio | APOB | 0.075406176 |
| -0.002977425 | 0.156538313 | 0.984824812 | IVW | ANGPT1 | 1 |
| -0.084580155 | 0.188840941 | 0.654231874 | IVW | AGRP | 1 |
| -0.077501348 | 0.085199451 | 0.363009182 | IVW | BCAM | 0.93822612 |
| 0.005290908 | 0.160027574 | 0.973624763 | IVW | CDH5 | 1 |
| -0.072217044 | 0.064240501 | 0.260942235 | IVW | CX3CL1 | 0.876802403 |
| -0.607065842 | 0.139517786 | 1.35E-05 | IVW | SPINT1 | 0.000510317 |
| -0.040274523 | 0.033741978 | 0.232633336 | IVW | KLK11 | 0.852052837 |
| -0.117862535 | 0.101622744 | 0.246128332 | IVW | KLK8 | 0.870512524 |
| 0.22909085 | 0.35512135 | 0.518858612 | Wald ratio | LCN2 | 0.994044169 |
| -0.018774812 | 0.092379923 | 0.838951539 | IVW | MET | 1 |
| 0.097380855 | 0.085060862 | 0.252276522 | IVW | SPINT2 | 0.870512524 |
| 0.018763166 | 0.061036678 | 0.758532806 | IVW | TIE1 | 1 |
| 0.094360143 | 0.315244858 | 0.764693349 | IVW | AIF1 | 1 |
| 0.601501782 | 0.157109235 | 0.000128896 | IVW | C5 | 0.004128981 |
| -0.282989305 | 0.466368984 | 0.543988479 | Wald ratio | MAP2K1 | 0.994044169 |
| 0.108107936 | 0.087746383 | 0.217930383 | IVW | SERPINA3 | 0.832728025 |
| -0.040716066 | 0.025078654 | 0.104475144 | IVW | C7 | 0.626325739 |
| -0.077909619 | 0.521459165 | 0.881232535 | Wald ratio | CCL28 | 1 |
| 0.001204384 | 0.03317399 | 0.971039117 | IVW | CCL14 | 1 |
| 0.095187502 | 0.146563246 | 0.516038728 | IVW | MDK | 0.994044169 |
| 0.050153623 | 0.054193251 | 0.354727291 | IVW | CCL23 | 0.936346055 |
| 0.330443895 | 0.305286242 | 0.279071822 | IVW | SERPINE1 | 0.880697634 |
| -0.163905117 | 0.043100265 | 0.000143024 | IVW | NBL1 | 0.004433741 |
| 0.042208039 | 0.10072502 | 0.675185256 | IVW | CFD | 1 |
| 0.03137608 | 0.176257938 | 0.858713318 | IVW | GHR | 1 |
| -0.537503597 | 0.517046763 | 0.298542153 | Wald ratio | IGFBP4 | 0.894057446 |
| -1.193597141 | 1.112721471 | 0.283413477 | Wald ratio | IGF1 | 0.882777802 |
| -0.11262571 | 0.19624389 | 0.566030847 | IVW | PROC | 0.999918464 |
| 0.033207683 | 0.423843325 | 0.937550475 | IVW | PTHLH | 1 |
| 0.009128046 | 0.053763137 | 0.865181096 | IVW | CLEC11A | 1 |
| -0.555151811 | 0.650739554 | 0.393598827 | Wald ratio | TNFSF15 | 0.960408371 |
| -0.249243631 | 0.527242038 | 0.636405141 | Wald ratio | BMP7 | 1 |
| -0.009384384 | 0.243353067 | 0.969238933 | IVW | CD36 | 1 |
| 0.051223244 | 0.144865135 | 0.723644198 | IVW | CNTN1 | 1 |
| -0.011986142 | 0.072623922 | 0.868909359 | IVW | EDAR | 1 |
| -0.304927595 | 0.230659747 | 0.186174778 | Wald ratio | CXCL5 | 0.798723043 |
| 0.314930582 | 0.537028143 | 0.557585128 | Wald ratio | LGALS4 | 0.994210735 |
| 0.001118758 | 0.044699346 | 0.980032218 | IVW | CXCL1 | 1 |
| 0.075224097 | 0.070152854 | 0.283590477 | IVW | IL1R1 | 0.882777802 |
| -0.041632757 | 0.028368629 | 0.142222902 | IVW | IL17RA | 0.718729001 |
| 0.056080982 | 0.369333742 | 0.879310258 | Wald ratio | IL18RAP | 1 |
| -0.105639712 | 0.11252421 | 0.347824385 | IVW | IL1RL2 | 0.936346055 |
| 0.045386091 | 0.066241438 | 0.493241945 | IVW | LSAMP | 0.994044169 |
| -0.053662286 | 0.042867073 | 0.210631833 | IVW | MBL2 | 0.829192218 |
| 0.425138677 | 0.202422921 | 0.035706882 | IVW | NCR3 | 0.370010771 |
| -0.126997199 | 0.097712746 | 0.19370401 | IVW | PDCD1LG2 | 0.807590253 |
| -0.004917062 | 0.049042825 | 0.92013746 | IVW | SIGLEC9 | 1 |
| 0.221790168 | 0.376176275 | 0.555464848 | IVW | TGFBR3 | 0.994044169 |
| 0.175162074 | 0.078881946 | 0.026380794 | IVW | SERPINF2 | 0.318738692 |
| 0.090310396 | 0.102185843 | 0.376811856 | IVW | FGF2 | 0.953842685 |
| 0.051946016 | 0.055290228 | 0.347466233 | IVW | CCL23 | 0.936346055 |
| -0.013311723 | 0.037973316 | 0.725922486 | IVW | CD209 | 1 |
| 0.131428277 | 0.103361361 | 0.203536015 | IVW | LGALS2 | 0.816842446 |
| -0.131734278 | 0.038533713 | 0.000629266 | IVW | CXCL11 | 0.01679791 |
| 0.035139032 | 0.03232162 | 0.276962548 | IVW | CCL3 | 0.880697634 |
| -0.176454803 | 0.169668875 | 0.298342164 | IVW | MRC2 | 0.894057446 |
| 0.928908982 | 0.548205238 | 0.090178899 | Wald ratio | SPARC | 0.584540565 |
| 0.031335286 | 0.046527084 | 0.500638882 | IVW | CCL18 | 0.994044169 |
| 0.156887699 | 0.09330235 | 0.092666259 | IVW | PTN | 0.593681833 |
| 0.009614012 | 0.060238837 | 0.873197567 | IVW | RETN | 1 |
| -0.797708978 | 0.596635449 | 0.181218598 | Wald ratio | PRSS1 | 0.79153876 |
| -0.087019105 | 0.028373258 | 0.002162616 | IVW | VWF | 0.051956857 |
| -0.059576305 | 0.047710274 | 0.211771093 | IVW | HP | 0.829192218 |
| 0.030088201 | 0.144042822 | 0.834539011 | IVW | TNFSF13B | 1 |
| 0.01951195 | 0.066971813 | 0.770786933 | IVW | C9 | 1 |
| 0.226528757 | 0.094803659 | 0.016873545 | IVW | LGALS3 | 0.251402742 |
| 0.289841838 | 0.166496902 | 0.081714397 | IVW | IL18BP | 0.563075399 |
| -0.02906553 | 0.042316063 | 0.492166211 | IVW | LBP | 0.994044169 |
| 0.183297 | 0.085229043 | 0.031504616 | IVW | F10 | 0.346511417 |
| -0.019284542 | 0.036493066 | 0.5971912 | IVW | RARRES2 | 1 |
| 0.023357401 | 0.152334158 | 0.878138021 | Wald ratio | VEGFC | 1 |
| 0.405942339 | 0.585637562 | 0.488207211 | Wald ratio | CD4 | 0.994044169 |
| -0.235342438 | 0.381165889 | 0.536952552 | Wald ratio | IL2RA | 0.994044169 |
| -0.131071356 | 0.397510991 | 0.741603875 | Wald ratio | TNFRSF1B | 1 |
| 0.00051775 | 0.056546343 | 0.992694512 | IVW | CD33 | 1 |
| -0.21880442 | 0.095624677 | 0.022128291 | IVW | ADAMTS5 | 0.295351212 |
| 0.05408631 | 0.04679215 | 0.247728499 | IVW | IDUA | 0.870512524 |
| 0.352716303 | 0.391616175 | 0.367764669 | Wald ratio | APP | 0.9449782 |
| 0.081494803 | 0.279711129 | 0.770781012 | IVW | ARSB | 1 |
| -0.028877814 | 0.054496529 | 0.596179958 | IVW | NAAA | 1 |
| 0.007520337 | 0.031748902 | 0.812758265 | IVW | ADAMTS13 | 1 |
| 0.083008903 | 0.112586209 | 0.460944842 | IVW | CTSS | 0.994044169 |
| 0.014906908 | 0.048078988 | 0.756522651 | IVW | F7 | 1 |
| 0.008078438 | 0.055455795 | 0.884179164 | IVW | C2 | 1 |
| 0.280112917 | 0.176778892 | 0.113071284 | IVW | WFIKKN1 | 0.654587372 |
| 0.111481616 | 0.073236913 | 0.127957654 | IVW | GP6 | 0.679923933 |
| -0.061775795 | 0.079778351 | 0.438728074 | IVW | GNLY | 0.97738713 |
| -0.106849185 | 0.061325035 | 0.081448616 | IVW | HAPLN1 | 0.563075399 |
| -0.285909682 | 0.206229046 | 0.165634072 | Wald ratio | KLK12 | 0.763426105 |
| -0.067520575 | 0.050165065 | 0.178312822 | IVW | LYVE1 | 0.78967107 |
| -0.821528244 | 0.442083969 | 0.063125267 | Wald ratio | METAP1 | 0.505528179 |
| -0.028205783 | 0.044901182 | 0.529889557 | IVW | ASAH2 | 0.994044169 |
| 0.027592237 | 0.130952883 | 0.833118604 | IVW | NID1 | 1 |
| 0.359227993 | 0.038295484 | 6.57E-21 | IVW | PIGR | 5.74E-19 |
| -0.05438406 | 0.052275876 | 0.298187513 | IVW | RET | 0.894057446 |
| -0.000226259 | 0.139049257 | 0.998701694 | IVW | SFRP1 | 1 |
| 0.128322441 | 0.188871982 | 0.496875003 | IVW | ACP5 | 0.994044169 |
| -0.195621175 | 0.184637895 | 0.289378725 | IVW | CCDC80 | 0.884165194 |
| -0.069033815 | 0.057943323 | 0.233495645 | IVW | WFIKKN2 | 0.852052837 |
| -0.401359873 | 0.009959011 | 0 | IVW | ACAN | 0 |
| -0.112894843 | 0.047808104 | 0.018205273 | IVW | TGFBI | 0.263086732 |
| -0.012978144 | 0.05916244 | 0.826366057 | IVW | C1R | 1 |
| -0.060152701 | 0.09060026 | 0.506731788 | IVW | CD109 | 0.994044169 |
| 0.005270593 | 0.05754387 | 0.927021728 | IVW | FCER2 | 1 |
| 0.024051267 | 0.101521162 | 0.812727465 | IVW | CD48 | 1 |
| -0.026354027 | 0.070601979 | 0.708943014 | IVW | CD5L | 1 |
| 0.048392634 | 0.037579904 | 0.197841228 | IVW | CNTN2 | 0.81076487 |
| -0.177917502 | 0.088840711 | 0.04521408 | IVW | CNTN4 | 0.423909569 |
| -0.089304556 | 0.067902651 | 0.188447699 | IVW | CNTN5 | 0.804634749 |
| 0.036317567 | 0.050855775 | 0.475147625 | IVW | CST7 | 0.994044169 |
| 0.650076881 | 0.501620538 | 0.194991513 | Wald ratio | DLL4 | 0.807701915 |
| -0.031163526 | 0.03598867 | 0.38653073 | IVW | FCGR2A | 0.957360905 |
| -0.085738172 | 0.032702941 | 0.008748557 | IVW | FCGR2B | 0.158629496 |
| -0.048045232 | 0.040373528 | 0.234039249 | IVW | FCGR3B | 0.852052837 |
| -0.030220471 | 0.050388766 | 0.548675387 | IVW | FCN2 | 0.994044169 |
| 0.170165319 | 0.160248736 | 0.288289059 | Wald ratio | GFRA1 | 0.884165194 |
| -0.116325329 | 0.063790212 | 0.068218501 | IVW | SERPIND1 | 0.524091257 |
| -0.095297514 | 0.130984808 | 0.466891352 | IVW | IGFBP7 | 0.994044169 |
| -0.095354841 | 0.054517049 | 0.080276685 | IVW | LRIG3 | 0.563075399 |
| -0.31414005 | 0.302007828 | 0.298260054 | IVW | LRP8 | 0.894057446 |
| -0.04019665 | 0.08410652 | 0.632703238 | IVW | LY9 | 1 |
| 0.043738016 | 0.172596848 | 0.799950286 | IVW | MATN2 | 1 |
| -0.125717864 | 0.212906394 | 0.554866068 | Wald ratio | CADM1 | 0.994044169 |
| -0.197343993 | 0.112807358 | 0.080223738 | IVW | NTN4 | 0.563075399 |
| 0.04384607 | 0.090303284 | 0.627291671 | IVW | PGLYRP1 | 1 |
| 0.366930423 | 0.162901025 | 0.024292295 | IVW | RGMB | 0.309241723 |
| 0.113531886 | 0.262283397 | 0.665116774 | Wald ratio | HFE2 | 1 |
| -0.145886447 | 0.691029304 | 0.832797767 | Wald ratio | TFPI | 1 |
| -0.024614171 | 0.04663036 | 0.597598455 | IVW | THBS2 | 1 |
| -0.323635376 | 0.140551263 | 0.021300543 | IVW | THBS4 | 0.288307345 |
| 0.09236968 | 0.117180378 | 0.430539276 | IVW | ACY1 | 0.971955213 |
| 0.093840725 | 0.623549865 | 0.880374617 | Wald ratio | SERPINC1 | 1 |
| -0.410681713 | 0.380101852 | 0.27994106 | Wald ratio | BMP1 | 0.880697634 |
| -0.036118779 | 0.047548176 | 0.447478816 | IVW | CA6 | 0.984043804 |
| 0.051450294 | 0.447226471 | 0.908411005 | Wald ratio | CSK | 1 |
| -0.190591465 | 0.094493573 | 0.043697803 | IVW | CTSV | 0.413729933 |
| 0.042014506 | 0.053047373 | 0.428349828 | IVW | ECM1 | 0.971955213 |
| 0.012662304 | 0.041894617 | 0.762467758 | IVW | FETUB | 1 |
| -0.001742674 | 0.059494486 | 0.976632222 | IVW | IL17RD | 1 |
| -0.051418248 | 0.122429717 | 0.674498777 | IVW | KLK7 | 1 |
| -0.067190076 | 0.08365149 | 0.42185086 | IVW | SERPINA5 | 0.971782639 |
| 0.339132231 | 0.589369835 | 0.565010676 | Wald ratio | PIK3CG | 0.999034516 |
| 0.055405424 | 0.110184882 | 0.6150764 | IVW | REN | 1 |
| 0.071216286 | 0.033642434 | 0.034271823 | IVW | TPSB2 | 0.359947781 |
| 0.087890678 | 0.301604873 | 0.770737762 | Wald ratio | IBSP | 1 |
| 0.114500456 | 0.655070615 | 0.861243851 | Wald ratio | CAMK2D | 1 |
| -0.14449296 | 0.143504915 | 0.313989998 | IVW | CA13 | 0.894057446 |
| 0.144328291 | 0.235155965 | 0.53937684 | IVW | TNFSF8 | 0.994044169 |
| 0.044892662 | 0.029778007 | 0.13166262 | IVW | EPHA1 | 0.690821526 |
| -0.17680264 | 0.219736015 | 0.421042551 | Wald ratio | FN1 | 0.971782639 |
| 0.181229936 | 0.222708917 | 0.415786756 | Wald ratio | FN1 | 0.971782639 |
| -0.014508562 | 0.108798638 | 0.893914668 | IVW | FSTL3 | 1 |
| -0.127467456 | 0.071775583 | 0.075746647 | IVW | GZMA | 0.551458541 |
| 0.029514234 | 0.559706204 | 0.957945718 | Wald ratio | IL15RA | 1 |
| -0.347669782 | 0.249907232 | 0.164166194 | IVW | CXCL8 | 0.760307047 |
| -0.268941259 | 0.439837762 | 0.54089801 | Wald ratio | INSR | 0.994044169 |
| 0.013074468 | 0.051033902 | 0.797802727 | IVW | SERPINA4 | 1 |
| 0.042095726 | 0.038193538 | 0.27038829 | IVW | POSTN | 0.880697634 |
| 0.018545351 | 0.042081464 | 0.659429332 | IVW | PDGFRB | 1 |
| -0.079606605 | 0.162796551 | 0.624845646 | IVW | BCAN | 1 |
| 0.213098235 | 0.294567377 | 0.469417086 | IVW | SELE | 0.994044169 |
| -0.005630719 | 0.674200669 | 0.993336388 | Wald ratio | THBS1 | 1 |
| 0.557712663 | 0.522821148 | 0.286090613 | IVW | XPNPEP1 | 0.884165194 |
| 0.017280528 | 0.133994817 | 0.897386016 | IVW | AGT | 1 |
| -0.169102299 | 0.078478 | 0.03117958 | IVW | CAT | 0.346399722 |
| -0.010723917 | 0.064805629 | 0.868567626 | IVW | CXCL6 | 1 |
| 0.445675199 | 0.444580629 | 0.316120498 | IVW | IL17B | 0.896023899 |
| -0.094061665 | 0.609908622 | 0.87743426 | IVW | HAMP | 1 |
| -0.138493149 | 0.120198884 | 0.249238913 | IVW | CCL22 | 0.870512524 |
| 0.312261199 | 0.073866944 | 2.36E-05 | IVW | CXCL12 | 0.000841633 |
| 0.024802233 | 0.054448039 | 0.648734359 | IVW | CPB2 | 1 |
| -0.144385459 | 0.1405082 | 0.304140591 | IVW | CCL17 | 0.894057446 |
| 0.017152907 | 0.101766215 | 0.866148973 | IVW | DKK1 | 1 |
| 0.036803845 | 0.141684178 | 0.795049111 | IVW | ADIPOQ | 1 |
| 0.030998907 | 0.068283422 | 0.649847455 | IVW | SERPINA1 | 1 |
| -0.008707484 | 0.083557253 | 0.917002866 | IVW | AHSG | 1 |
| 0.641956342 | 0.362500691 | 0.076575558 | Wald ratio | ARSA | 0.552451523 |
| 0.445530855 | 0.601774626 | 0.459080583 | Wald ratio | BMP10 | 0.993644616 |
| 0.353060036 | 0.146981322 | 0.016302403 | IVW | CASP3 | 0.246718256 |
| -0.034107909 | 0.040858852 | 0.403844952 | IVW | CHIT1 | 0.967123738 |
| -0.298083636 | 0.364298182 | 0.413219701 | Wald ratio | MASP1 | 0.971782639 |
| 0.084121945 | 0.093867431 | 0.370157601 | IVW | DKK3 | 0.949857021 |
| -0.001473921 | 0.03948236 | 0.970220981 | IVW | HGFAC | 1 |
| 0.268591949 | 0.187968549 | 0.153027336 | IVW | LGMN | 0.744603897 |
| 0.66911662 | 0.66051676 | 0.311050657 | Wald ratio | CADM3 | 0.894057446 |
| 0.071288498 | 0.08201015 | 0.384702567 | IVW | LRPAP1 | 0.957360905 |
| 0.003681386 | 0.049737306 | 0.940997179 | IVW | KDR | 1 |
| -0.050334938 | 0.167158744 | 0.76332255 | IVW | A2M | 1 |
| -0.05580384 | 0.051936546 | 0.282615715 | IVW | PLG | 0.882777802 |
| 0.399704225 | 0.375639602 | 0.287300133 | Wald ratio | CDKN1B | 0.884165194 |
| 0.179042447 | 0.367259675 | 0.62589757 | Wald ratio | PYY | 1 |
| -0.276980801 | 0.227459538 | 0.223332447 | IVW | TEK | 0.841071679 |
| 0.192312836 | 0.840784012 | 0.81907849 | Wald ratio | ADAM9 | 1 |
| 0.316124835 | 0.158328341 | 0.04586421 | IVW | ANGPTL4 | 0.426810327 |
| 0.084469417 | 0.152477754 | 0.579593473 | IVW | CA3 | 1 |
| -0.051530478 | 0.086284561 | 0.55036351 | IVW | CST5 | 0.994044169 |
| -0.471256216 | 0.309730792 | 0.128133751 | IVW | ESM1 | 0.679923933 |
| 0.075572544 | 0.175891911 | 0.667447842 | IVW | EPHA5 | 1 |
| 0.522622093 | 0.57827907 | 0.366125794 | Wald ratio | FGF23 | 0.943871065 |
| -0.166394962 | 0.111292442 | 0.134884517 | IVW | FGR | 0.700650694 |
| 0.019988498 | 0.196723984 | 0.919068768 | IVW | MAPKAPK2 | 1 |
| -0.191026643 | 0.526634103 | 0.716805658 | Wald ratio | MAPK8 | 1 |
| 0.022430344 | 0.868834081 | 0.97940362 | Wald ratio | PTEN | 1 |
| -0.069502456 | 0.4341523 | 0.872811966 | Wald ratio | TLR2 | 1 |
| 1.507608426 | 0.564314746 | 0.007549649 | Wald ratio | GAPDH | 0.144804998 |
| 1.719260038 | 0.582472275 | 0.003160709 | Wald ratio | DNAJB1 | 0.072320028 |
| 1.160435762 | 0.444388079 | 0.009019566 | Wald ratio | MDH1 | 0.16201501 |
| 0.114083741 | 0.164985263 | 0.489264968 | IVW | PRDX1 | 0.994044169 |
| 0.012861774 | 0.040474906 | 0.750658223 | IVW | ACP1 | 1 |
| 0.415234177 | 0.446088608 | 0.351939747 | Wald ratio | SBDS | 0.936346055 |
| -0.037579396 | 0.088464474 | 0.670985055 | IVW | LDHB | 1 |
| -0.027028602 | 0.084105118 | 0.747932356 | IVW | NAGK | 1 |
| -0.839181388 | 0.594116719 | 0.157806989 | Wald ratio | UBE2N | 0.754698922 |
| -1.152649899 | 0.705283702 | 0.102194462 | Wald ratio | HSPA1A | 0.626325739 |
| -0.08900191 | 0.05111438 | 0.081643208 | IVW | AGER | 0.563075399 |
| 0.029480028 | 0.106503304 | 0.781934304 | IVW | BPI | 1 |
| 0.072062613 | 0.097463632 | 0.459676578 | IVW | C6 | 0.993644616 |
| 0.144689993 | 0.105995014 | 0.172232891 | IVW | CFB | 0.775249689 |
| -0.159825568 | 0.198636364 | 0.421042551 | Wald ratio | FN1 | 0.971782639 |
| 1.074987469 | 0.361127193 | 0.002913159 | Wald ratio | GZMB | 0.067458944 |
| 0.076327184 | 0.074691501 | 0.306828618 | IVW | CXCL10 | 0.894057446 |
| -0.541218348 | 0.046894627 | 8.18E-31 | IVW | NTF3 | 9.83E-29 |
| -0.066285845 | 0.099860382 | 0.506827788 | IVW | PLG | 0.994044169 |
| -0.046147052 | 0.147818444 | 0.754898126 | IVW | KLKB1 | 1 |
| 0.102840082 | 0.077467138 | 0.18433286 | IVW | SERPINA3 | 0.795137666 |
| 0.082271334 | 0.061543904 | 0.181290882 | IVW | SELP | 0.79153876 |
| 0.026420925 | 0.04362247 | 0.544732321 | IVW | TNC | 0.994044169 |
| 0.151852444 | 0.255181333 | 0.551792258 | Wald ratio | F2 | 0.994044169 |
| -0.056375592 | 0.113180525 | 0.618411239 | IVW | PLAU | 1 |
| 0.073286158 | 0.113147198 | 0.517174897 | IVW | CFH | 0.994044169 |
| 0.143803099 | 0.359113947 | 0.688833472 | Wald ratio | MMP2 | 1 |
| -0.199725896 | 0.202714007 | 0.324496635 | IVW | TF | 0.907834835 |
| -0.311246713 | 0.305163015 | 0.307758863 | Wald ratio | PGD | 0.894057446 |
| -0.192303998 | 0.208068192 | 0.355363745 | IVW | AKR7A2 | 0.936346055 |
| -0.034138988 | 0.042953401 | 0.426734936 | IVW | AKR1A1 | 0.971782639 |
| 0.322358871 | 0.582629032 | 0.580069485 | Wald ratio | VTA1 | 1 |
| 0.257599738 | 0.397145669 | 0.516578956 | Wald ratio | FER | 0.994044169 |
| -0.07926514 | 0.355124006 | 0.823376648 | IVW | IGF1R | 1 |
| 0.03325981 | 0.05443106 | 0.541169997 | IVW | IL1RL1 | 0.994044169 |
| 0.132395266 | 0.707532468 | 0.851564563 | Wald ratio | PKM2 | 1 |
| -0.162048808 | 0.183877412 | 0.378162397 | Wald ratio | NME2 | 0.953842685 |
| 0.911083164 | 0.583492901 | 0.118422391 | Wald ratio | NSFL1C | 0.669395967 |
| -0.481517693 | 0.465577982 | 0.301025664 | Wald ratio | NUDCD3 | 0.894057446 |
| 0.113078374 | 0.055513629 | 0.041655257 | IVW | PEBP1 | 0.410571302 |
| -0.24592887 | 0.240172385 | 0.305850324 | Wald ratio | P4HB | 0.894057446 |
| 0.101826075 | 0.176265261 | 0.563475745 | IVW | SPHK1 | 0.998482994 |
| -0.017095629 | 0.090908208 | 0.850834523 | IVW | SPON1 | 1 |
| 0.758293594 | 0.577585409 | 0.189227319 | Wald ratio | TPI1 | 0.804634749 |
| -0.078625251 | 0.583408027 | 0.892794605 | Wald ratio | DCTPP1 | 1 |
| 0.053983834 | 0.05327138 | 0.310881533 | IVW | CST2 | 0.894057446 |
| 0.064236693 | 0.015214149 | 2.42E-05 | IVW | BOC | 0.000845553 |
| -0.387670492 | 0.254196701 | 0.127238962 | IVW | CLEC1B | 0.679923933 |
| 0.054149674 | 0.022035102 | 0.013993589 | IVW | CRP | 0.220456381 |
| 0.020915785 | 0.024531131 | 0.39386889 | IVW | ICAM1 | 0.960408371 |
| 0.102034716 | 0.155061069 | 0.510519347 | IVW | DAPK2 | 0.994044169 |
| 0.019062181 | 0.119868965 | 0.873649056 | IVW | GDF15 | 1 |
| -0.031369821 | 0.027554303 | 0.254923151 | IVW | MST1 | 0.875138353 |
| -0.041305253 | 0.02860251 | 0.148707224 | IVW | COLEC11 | 0.731831718 |
| 0.02794004 | 0.047143323 | 0.553407609 | IVW | ENPP7 | 0.994044169 |
| 0.070365783 | 0.053856699 | 0.191369924 | IVW | ENTPD5 | 0.804842439 |
| -0.162981132 | 0.115822064 | 0.159377501 | IVW | FCRL3 | 0.75537221 |
| 0.191197859 | 0.072340979 | 0.008217285 | IVW | MFGE8 | 0.150451017 |
| -0.11014747 | 0.049406213 | 0.025785609 | IVW | PCSK7 | 0.317691931 |
| 0.29656812 | 0.338847346 | 0.381450768 | IVW | SIGLEC1 | 0.957360905 |
| 0.009728143 | 0.076190668 | 0.898400985 | IVW | SPARCL1 | 1 |
| -0.037766294 | 0.00394861 | 1.13E-21 | IVW | CHST15 | 1.03E-19 |
| 0.04354344 | 0.063446296 | 0.492521492 | IVW | TGM3 | 0.994044169 |
| -0.070037742 | 0.076974336 | 0.362883518 | IVW | SERPING1 | 0.93822612 |
| -0.24017052 | 0.36216763 | 0.507236159 | Wald ratio | C3 | 0.994044169 |
| -0.018733763 | 0.045216679 | 0.678646424 | IVW | MMP12 | 1 |
| -0.070420293 | 0.055441838 | 0.204025794 | IVW | NCAM1 | 0.816953284 |
| -0.182743217 | 0.303577393 | 0.547196885 | IVW | PDGFA | 0.994044169 |
| 0.039672024 | 0.047545858 | 0.404058461 | IVW | CLEC11A | 0.967123738 |
| -1.331817898 | 1.070823199 | 0.213597933 | Wald ratio | ADAMTS15 | 0.829192218 |
| -0.046964933 | 0.085095104 | 0.581009256 | IVW | PRSS22 | 1 |
| 0.014307757 | 0.03940442 | 0.716529966 | IVW | BST1 | 1 |
| 0.076464526 | 0.086860683 | 0.378690165 | IVW | CDON | 0.953922015 |
| 0.129445319 | 0.05155368 | 0.01204292 | IVW | ADGRE2 | 0.196156715 |
| -0.04585097 | 0.04885716 | 0.348003019 | IVW | FUT3 | 0.936346055 |
| -0.01646748 | 0.031315691 | 0.598989679 | IVW | FUT5 | 1 |
| -0.069795135 | 0.067545729 | 0.301462608 | IVW | KYNU | 0.894057446 |
| -0.392904335 | 0.431895366 | 0.362969605 | Wald ratio | PLCG1 | 0.93822612 |
| -0.010994554 | 0.021188627 | 0.603837871 | IVW | PLXNC1 | 1 |
| 0.172210568 | 0.170004643 | 0.31107178 | IVW | SLITRK5 | 0.894057446 |
| 0.863494202 | 0.78915165 | 0.27386479 | Wald ratio | LTA | 0.880697634 |
| 0.043825444 | 0.342778909 | 0.898265002 | IVW | EPB41 | 1 |
| 0.185263171 | 0.144003437 | 0.198261961 | IVW | TFF3 | 0.81076487 |
| -0.037508978 | 0.102334039 | 0.713966112 | IVW | AFM | 1 |
| 0.105389378 | 0.006068991 | 1.51E-67 | IVW | SMPDL3A | 2.91E-65 |
| -0.160410519 | 0.280041193 | 0.56677305 | IVW | GSN | 1 |
| -0.158102354 | 0.084804967 | 0.062278836 | IVW | LPO | 0.502940851 |
| -0.020861299 | 0.059415792 | 0.725508096 | IVW | ITIH4 | 1 |
| 0.00137794 | 0.104412724 | 0.989470579 | IVW | SELL | 1 |
| 0.130596902 | 0.121974242 | 0.284307731 | IVW | EPHA2 | 0.882777802 |
| 0.044190386 | 0.295939575 | 0.881299298 | IVW | NTRK2 | 1 |
| 0.017969711 | 0.081985594 | 0.826508806 | IVW | VEGFA | 1 |
| -0.02853297 | 0.03349245 | 0.39425713 | IVW | ANG | 0.960408371 |
| 0.148599303 | 0.218382114 | 0.496216111 | Wald ratio | F10 | 0.994044169 |
| -0.034721883 | 0.043620269 | 0.426030051 | IVW | CCL7 | 0.971782639 |
| -0.09828649 | 0.44654041 | 0.825788138 | Wald ratio | C3 | 1 |
| 0.118824288 | 0.082494113 | 0.149755058 | IVW | F5 | 0.734200088 |
| 0.001011669 | 0.0251902 | 0.967964592 | IVW | ENG | 1 |
| 0.136882645 | 0.267618168 | 0.609011577 | IVW | GSTP1 | 1 |
| -0.127912323 | 0.093580075 | 0.171664295 | IVW | CCL16 | 0.774504164 |
| 0.165043769 | 0.059133709 | 0.005254112 | IVW | LYZ | 0.108584976 |
| 0.951517598 | 0.620927536 | 0.125420484 | Wald ratio | CCL19 | 0.67523297 |
| -0.027277344 | 0.053200634 | 0.608143048 | IVW | MMP1 | 1 |
| -0.447563107 | 0.488503236 | 0.359565825 | Wald ratio | MMP13 | 0.93822612 |
| 0.047424335 | 0.075038628 | 0.527387558 | IVW | SHBG | 0.994044169 |
| -1.209458167 | 0.509396813 | 0.01758251 | Wald ratio | STC1 | 0.259950641 |
| -0.136698106 | 0.196799732 | 0.487302789 | IVW | ANXA1 | 0.994044169 |
| 0.100816089 | 0.044934997 | 0.024858249 | IVW | ANXA2 | 0.312271593 |
| -0.028605761 | 0.062018344 | 0.644621573 | IVW | CDNF | 1 |
| -0.006777642 | 0.024939105 | 0.785800687 | IVW | ERAP1 | 1 |
| -0.066776173 | 0.074029594 | 0.367046251 | IVW | CAPG | 0.944394773 |
| 0.075473844 | 0.011511298 | 5.51E-11 | IVW | CA1 | 3.31E-09 |
| -0.122370298 | 0.098060044 | 0.212063313 | IVW | CTSZ | 0.829192218 |
| -0.504877095 | 0.430650838 | 0.241053224 | Wald ratio | DBNL | 0.861160403 |
| 0.034647575 | 0.098906139 | 0.726107844 | IVW | DPT | 1 |
| 0.000563655 | 0.109238905 | 0.99588306 | IVW | PI3 | 1 |
| -0.149808636 | 0.116780411 | 0.199553977 | IVW | ESD | 0.810778384 |
| -0.123611905 | 0.115978486 | 0.286506129 | IVW | FGG | 0.884165194 |
| -0.194862558 | 0.109268172 | 0.074530592 | IVW | GP1BA | 0.550638249 |
| 0.199903794 | 0.07699251 | 0.009420475 | IVW | GPC5 | 0.167649569 |
| -0.013482637 | 0.07041283 | 0.848149674 | IVW | GRN | 1 |
| 0.023792763 | 0.028925557 | 0.410763048 | IVW | HRG | 0.971782639 |
| 0.098400849 | 0.115196969 | 0.392996113 | IVW | LGALS3BP | 0.960408371 |
| -0.669149991 | 0.064530313 | 3.41E-25 | IVW | MAPK12 | 3.28E-23 |
| 0.04034654 | 0.299788062 | 0.892941153 | Wald ratio | MAPK13 | 1 |
| 0.413213433 | 0.188372799 | 0.028264614 | IVW | PLA2G7 | 0.326936167 |
| 0.578530945 | 0.332795603 | 0.082140849 | Wald ratio | PRDX6 | 0.563838258 |
| -0.110042198 | 0.017019587 | 1.01E-10 | IVW | PPA1 | 5.77E-09 |
| 0.209336034 | 0.244742879 | 0.392368241 | IVW | CD163 | 0.960408371 |
| -0.01629327 | 0.059584926 | 0.784510269 | IVW | FAP | 1 |
| -0.108476564 | 0.145897008 | 0.457170149 | IVW | PRSS2 | 0.993644616 |
| -0.020890244 | 0.050770562 | 0.680732549 | IVW | TNFAIP6 | 1 |
| 0.062377783 | 0.025464868 | 0.014302991 | IVW | CD274 | 0.223498766 |
| 0.353387232 | 0.547501552 | 0.518632823 | IVW | CD244 | 0.994044169 |
| -0.269327322 | 0.105033071 | 0.010340962 | IVW | CD300C | 0.17507234 |
| 0.003619341 | 0.065136764 | 0.955688143 | IVW | CD55 | 1 |
| -0.296882051 | 0.421582051 | 0.481302366 | Wald ratio | EPHA10 | 0.994044169 |
| 0.097697673 | 0.067263415 | 0.146372698 | IVW | EPHB6 | 0.730722924 |
| 0.174300643 | 0.551522508 | 0.75197563 | Wald ratio | IL20RA | 1 |
| -0.100294629 | 0.180555668 | 0.578568031 | IVW | IL22RA2 | 1 |
| 0.215404282 | 0.366610831 | 0.556830556 | Wald ratio | IL7R | 0.994210735 |
| -0.022743036 | 0.02947724 | 0.440383587 | IVW | LILRB1 | 0.97738713 |
| 0.043598073 | 0.02742473 | 0.111894395 | IVW | LILRB2 | 0.651700085 |
| -0.040376442 | 0.015817528 | 0.010691016 | IVW | JAG1 | 0.177139071 |
| -0.081701623 | 0.084568058 | 0.333991644 | IVW | KIR2DL4 | 0.922316005 |
| 0.072671271 | 0.117715293 | 0.537005378 | IVW | KIR3DS1 | 0.994044169 |
| -0.02856362 | 0.046361908 | 0.537827831 | IVW | MICB | 0.994044169 |
| -0.114744193 | 0.189245855 | 0.544300012 | IVW | CD200R1 | 0.994044169 |
| 0.050961582 | 0.08942879 | 0.568774992 | IVW | RTN4R | 1 |
| 0.306138838 | 0.178890906 | 0.087022828 | IVW | NOTCH1 | 0.576254043 |
| -0.00066688 | 0.293381525 | 0.998186346 | IVW | NOTCH3 | 1 |
| -0.36791458 | 0.461097202 | 0.424922075 | Wald ratio | NRCAM | 0.971782639 |
| 0.072556138 | 0.589556685 | 0.902052478 | Wald ratio | NRXN3 | 1 |
| 0.06357375 | 0.319428125 | 0.842244248 | Wald ratio | CD200 | 1 |
| -1.30671126 | 0.708895206 | 0.065284248 | Wald ratio | ROBO2 | 0.516363478 |
| 0.034117749 | 0.246453232 | 0.889896652 | IVW | SEMA6B | 1 |
| 0.069903629 | 0.076039208 | 0.357933281 | IVW | ICAM5 | 0.93822612 |
| -0.227870056 | 0.168870056 | 0.1772146 | Wald ratio | SLAMF6 | 0.788440882 |
| -0.019028441 | 0.09621775 | 0.843229439 | IVW | SCARF1 | 1 |
| -0.287944498 | 0.290502392 | 0.321590407 | Wald ratio | TNFRSF19 | 0.903650238 |
| -0.064922545 | 0.043630278 | 0.136746712 | IVW | IL27RA | 0.700872481 |
| -0.03261866 | 0.06151446 | 0.595931654 | IVW | HAVCR2 | 1 |
| 0.033687729 | 0.156728002 | 0.829811054 | IVW | UNC5C | 1 |
| -0.500381779 | 0.620763557 | 0.420199797 | Wald ratio | NMT1 | 0.971782639 |
| -0.169042268 | 0.103861731 | 0.103616031 | IVW | PPID | 0.626325739 |
| -0.629630485 | 0.345523095 | 0.068416611 | Wald ratio | GCKR | 0.524091257 |
| 0.365067585 | 0.029025118 | 2.80E-36 | IVW | PDK1 | 3.59E-34 |
| 0.094997045 | 0.057560474 | 0.098863854 | IVW | PCSK9 | 0.614808874 |
| -0.666726569 | 0.103147876 | 1.02E-10 | IVW | PPIE | 5.77E-09 |
| -0.045306692 | 0.094188005 | 0.630499431 | IVW | PDE5A | 1 |
| -0.163378451 | 0.383366874 | 0.669985896 | Wald ratio | VAV1 | 1 |
| 0.294196984 | 0.230388101 | 0.201615459 | IVW | CCL11 | 0.81408595 |
| 0.175936148 | 0.295652935 | 0.551792258 | Wald ratio | F2 | 0.994044169 |
| 0.294750202 | 0.311124852 | 0.343450516 | IVW | CD86 | 0.936346055 |
| 0.533960894 | 0.610729981 | 0.381955193 | Wald ratio | S100A9 | 0.957360905 |
| -0.008172459 | 0.036023583 | 0.820529387 | IVW | CPNE1 | 1 |
| -0.306765714 | 0.220320732 | 0.163813494 | IVW | DLL1 | 0.760307047 |
| 1.160625 | 0.661123016 | 0.079167734 | Wald ratio | GPC6 | 0.562759689 |
| 0.010108508 | 0.15051897 | 0.946456157 | IVW | IL1RN | 1 |
| -0.190015132 | 0.073938116 | 0.010172165 | IVW | TNFSF14 | 0.17507234 |
| 0.737854853 | 0.788073909 | 0.349130994 | Wald ratio | OMD | 0.936346055 |
| -0.096278826 | 0.043037743 | 0.025281186 | IVW | SEMA3E | 0.315286311 |
| 0.011305513 | 0.071717259 | 0.874740429 | IVW | FAS | 1 |
| 0.04133825 | 0.062836009 | 0.51061835 | IVW | LEPR | 0.994044169 |
| 0.228982536 | 0.183545435 | 0.212195043 | IVW | TNFRSF21 | 0.829192218 |
| 0.01290481 | 0.037981006 | 0.734029666 | IVW | SIRPA | 1 |
| -0.160167688 | 0.378295559 | 0.672008559 | Wald ratio | NPPA | 1 |
| -0.25078539 | 0.261969535 | 0.338412091 | IVW | ALCAM | 0.929182913 |
| -0.069704502 | 0.300527468 | 0.816584199 | IVW | ASGR1 | 1 |
| -0.018997231 | 0.037383084 | 0.611329073 | IVW | CNDP1 | 1 |
| 0.107127139 | 0.158605911 | 0.499402815 | IVW | COLEC12 | 0.994044169 |
| 0.066860505 | 0.061730113 | 0.278759586 | IVW | CST1 | 0.880697634 |
| 0.152234461 | 0.67238605 | 0.820883088 | Wald ratio | DDX19B | 1 |
| -0.020701009 | 0.081546182 | 0.799606606 | IVW | FCN3 | 1 |
| 0.271214636 | 0.127677175 | 0.033651602 | IVW | GAS1 | 0.358904016 |
| 0.206859638 | 0.074370804 | 0.00541145 | IVW | HS6ST1 | 0.110646888 |
| 0.089306593 | 0.479057387 | 0.852114239 | Wald ratio | PRKCB | 1 |
| -0.1518541 | 0.228807307 | 0.506897195 | IVW | FOLH1 | 0.994044169 |
| -0.068294122 | 0.107794598 | 0.526369368 | IVW | CCL5 | 0.994044169 |
| 0.049760831 | 0.569186364 | 0.930334099 | IVW | RGMA | 1 |
| -0.04921089 | 0.082354717 | 0.550141625 | IVW | SLAMF7 | 0.994044169 |
| -1.215291925 | 1.316993789 | 0.356123291 | Wald ratio | SRC | 0.936346055 |
| 0.009703143 | 0.26553708 | 0.970850529 | IVW | SPOCK2 | 1 |
| 0.110578229 | 0.098464122 | 0.261424469 | IVW | CTSD | 0.876889755 |
| -0.060428455 | 0.126878907 | 0.633882943 | IVW | EGF | 1 |
| -0.173994101 | 0.295104105 | 0.555457213 | IVW | FGFR1 | 0.994044169 |
| 0.007318947 | 0.077391544 | 0.924656088 | IVW | NRP1 | 1 |
| 0.059760948 | 0.033481597 | 0.074279255 | IVW | FGL1 | 0.550638249 |
| -0.018036566 | 0.033951102 | 0.595244663 | IVW | TCN2 | 1 |
| 0.634805907 | 0.306737342 | 0.038495225 | Wald ratio | MINPP1 | 0.388320059 |
| -0.04640126 | 0.033888735 | 0.17092936 | IVW | PDIA5 | 0.774504164 |
| 0.758635117 | 0.74622085 | 0.309326535 | Wald ratio | GREM2 | 0.894057446 |
| 0.106069899 | 0.088454554 | 0.230471291 | IVW | PGLYRP2 | 0.850634127 |
| -0.951630268 | 0.584681992 | 0.103609055 | Wald ratio | FAM19A5 | 0.626325739 |
| 0.686986913 | 1.999506246 | 0.731163463 | Wald ratio | FAM171A1 | 1 |
| 0.137373967 | 0.759144628 | 0.856399847 | Wald ratio | FAM172A | 1 |
| 0.024792078 | 0.089708563 | 0.7822697 | IVW | FAM3B | 1 |
| -0.021384242 | 0.035659211 | 0.548717154 | IVW | PAM | 0.994044169 |
| -0.111393527 | 0.133397088 | 0.403688338 | IVW | THSD1 | 0.967123738 |
| -0.044481439 | 0.111098318 | 0.688877352 | IVW | SEMA3G | 1 |
| -0.037581435 | 0.049073246 | 0.443781444 | IVW | CD300A | 0.982658912 |
| -0.049179972 | 0.057242573 | 0.390257613 | IVW | MLN | 0.960408371 |
| 0.039776273 | 0.088588719 | 0.653432164 | IVW | CRTAC1 | 1 |
| 0.101687212 | 0.088269515 | 0.249317673 | IVW | POFUT1 | 0.870512524 |
| -0.076560913 | 0.08262584 | 0.354135455 | IVW | MFAP4 | 0.936346055 |
| 0.066807474 | 0.047302774 | 0.157850659 | IVW | NTNG1 | 0.754698922 |
| 0.00883808 | 0.06190692 | 0.886476567 | IVW | COLGALT1 | 1 |
| -0.066468588 | 0.051054499 | 0.192945648 | IVW | RNASE4 | 0.807590253 |
| 0.035346283 | 0.050635509 | 0.48514387 | IVW | RNASE6 | 0.994044169 |
| -0.015938429 | 0.041818942 | 0.70310721 | IVW | CTRB2 | 1 |
| -0.047270856 | 0.061946196 | 0.445406517 | IVW | PSG4 | 0.983911145 |
| 0.456678493 | 0.415198123 | 0.271373514 | IVW | PSG7 | 0.880697634 |
| 0.120402707 | 0.497458647 | 0.808752571 | Wald ratio | ENDOU | 1 |
| -0.115597895 | 0.100301184 | 0.249112475 | IVW | ST3GAL1 | 0.870512524 |
| -0.15173279 | 0.035760141 | 2.20E-05 | IVW | F13B | 0.000799561 |
| 0.002438463 | 0.032580086 | 0.940337886 | IVW | SOD3 | 1 |
| -0.074500002 | 0.073950111 | 0.313725311 | IVW | PF4V1 | 0.894057446 |
| 0.025186298 | 0.089923865 | 0.779412528 | IVW | CTRB1 | 1 |
| 0.086040909 | 0.109280365 | 0.43108229 | IVW | ASIP | 0.971955213 |
| 0.134095826 | 0.088012991 | 0.127610841 | IVW | OBP2B | 0.679923933 |
| -0.09223769 | 0.394357868 | 0.815067761 | Wald ratio | VASN | 1 |
| 0.037316431 | 0.280728799 | 0.89425113 | Wald ratio | PRKCSH | 1 |
| -0.086967313 | 0.209882866 | 0.678609611 | IVW | CBLN4 | 1 |
| 0.074333128 | 0.081716241 | 0.363007452 | IVW | CRISPLD2 | 0.93822612 |
| 0.028251646 | 0.029922161 | 0.345082123 | IVW | TNXB | 0.936346055 |
| 0.013308296 | 0.253563136 | 0.958142138 | IVW | CLEC3B | 1 |
| 0.226553316 | 0.321909294 | 0.481569804 | IVW | OLFM1 | 0.994044169 |
| -0.313900183 | 0.144246747 | 0.029545277 | IVW | GZMM | 0.334035426 |
| -0.002444777 | 0.087958748 | 0.977825985 | IVW | LEAP2 | 1 |
| 0.883303333 | 0.5928 | 0.136210339 | Wald ratio | IFNL3 | 0.700872481 |
| -0.034192602 | 0.125091114 | 0.784590659 | IVW | PRCP | 1 |
| -0.16401881 | 0.118149206 | 0.165065634 | IVW | B3GALTL | 0.762634973 |
| 0.018292808 | 0.104203835 | 0.86064881 | IVW | FCRL1 | 1 |
| -0.100827974 | 0.05600239 | 0.071793919 | IVW | SPINK6 | 0.539015285 |
| 0.00619736 | 0.538190051 | 0.990812412 | Wald ratio | PVRL4 | 1 |
| -0.113095004 | 0.057013803 | 0.047295704 | IVW | TREML2 | 0.430852421 |
| -0.078770548 | 0.03810773 | 0.038729533 | IVW | SEMA4D | 0.388320059 |
| 0.0007865 | 0.148882546 | 0.995785042 | IVW | ROBO1 | 1 |
| -0.01837103 | 0.033606189 | 0.584614985 | IVW | ACP6 | 1 |
| -0.294325809 | 0.266878522 | 0.270094214 | IVW | MENT | 0.880697634 |
| 0.005238088 | 0.056033327 | 0.92552093 | IVW | CLPS | 1 |
| -0.041614717 | 0.111593411 | 0.709212637 | IVW | DEFB104A | 1 |
| -0.345910836 | 0.260518345 | 0.184251064 | Wald ratio | BID | 0.795137666 |
| 0.071288982 | 0.038816317 | 0.066273024 | IVW | C3 | 0.518029501 |
| 0.046404888 | 0.050906806 | 0.361997491 | IVW | TDGF1 | 0.93822612 |
| -0.336946825 | 0.071247404 | 2.25E-06 | IVW | IFNGR1 | 9.42E-05 |
| -0.104129916 | 0.171461307 | 0.543646173 | IVW | LIFR | 0.994044169 |
| 0.13784429 | 0.012148078 | 7.67E-30 | IVW | S100A12 | 8.68E-28 |
| -0.119711357 | 0.188759286 | 0.525949556 | IVW | ARG1 | 0.994044169 |
| -0.07949 | 0.331354639 | 0.810412409 | Wald ratio | HINT1 | 1 |
| 0.174052365 | 0.53139527 | 0.743260692 | Wald ratio | PSME1 | 1 |
| -0.02396723 | 0.084802298 | 0.777464494 | IVW | TNFSF12 | 1 |
| 0.167428053 | 0.442887545 | 0.705402816 | Wald ratio | DMKN | 1 |
| -0.299052028 | 0.251555556 | 0.234514022 | Wald ratio | BOLA3 | 0.852052837 |
| -0.353258941 | 0.537841202 | 0.511303737 | Wald ratio | TNFSF14 | 0.994044169 |
| 0.162495299 | 0.070120544 | 0.020483543 | IVW | CRHBP | 0.285285283 |
| 0.157240806 | 0.254975819 | 0.537439817 | Wald ratio | PTPRS | 0.994044169 |
| 0.006400321 | 0.0485486 | 0.895116165 | IVW | PIP | 1 |
| -0.016321179 | 0.086347453 | 0.850079106 | IVW | CECR1 | 1 |
| 0.125944221 | 0.068504598 | 0.065991948 | IVW | PCOLCE2 | 0.518029501 |
| -0.047287748 | 0.044461887 | 0.287529464 | IVW | CHRDL2 | 0.884165194 |
| -0.398875776 | 0.651447205 | 0.540344719 | Wald ratio | MAP2K3 | 0.994044169 |
| 0.097708627 | 0.135402353 | 0.470530044 | IVW | PSAP | 0.994044169 |
| -0.011155175 | 0.378288941 | 0.976474988 | IVW | GUCA2B | 1 |
| 0.088971456 | 0.057904374 | 0.124409933 | IVW | KLK10 | 0.67523297 |
| -0.009256108 | 0.458909938 | 0.983907943 | Wald ratio | PVRL2 | 1 |
| -0.005317119 | 0.031096963 | 0.86423543 | IVW | SIRPB1 | 1 |
| -0.000549017 | 0.033969948 | 0.987105272 | IVW | SCGB3A1 | 1 |
| 0.079135665 | 0.066811972 | 0.236233645 | IVW | CPXM1 | 0.856681255 |
| 0.200715023 | 0.145488736 | 0.167712458 | IVW | NPNT | 0.767484151 |
| 0.031971602 | 0.111347581 | 0.774010228 | IVW | POMGNT2 | 1 |
| -0.027772515 | 0.055166857 | 0.614663711 | IVW | TAPBPL | 1 |
| -0.010384529 | 0.045558029 | 0.819692347 | IVW | TXNDC15 | 1 |
| -0.184457851 | 0.109239565 | 0.09130327 | IVW | FMOD | 0.588875454 |
| 0.001726791 | 0.099394079 | 0.98613891 | IVW | XXYLT1 | 1 |
| 0.101222519 | 0.144028099 | 0.482182253 | IVW | ADAMTSL2 | 0.994044169 |
| 0.178231814 | 0.119247773 | 0.135009903 | IVW | MANBA | 0.700650694 |
| 0.908552901 | 0.517066553 | 0.078895736 | Wald ratio | TLL1 | 0.562759689 |
| -0.126533515 | 0.086624039 | 0.144092143 | IVW | VWA1 | 0.723094254 |
| -0.084619466 | 0.117219694 | 0.470363466 | IVW | CCDC126 | 0.994044169 |
| 0.081147388 | 0.024324292 | 0.000849737 | IVW | LILRA3 | 0.022070197 |
| -0.023712905 | 0.057549283 | 0.680306301 | IVW | WISP2 | 1 |
| 0.022391692 | 0.045759536 | 0.624605321 | IVW | HSP90B1 | 1 |
| 0.011804493 | 0.046907979 | 0.801309955 | IVW | PILRA | 1 |
| -0.065256474 | 0.17415875 | 0.707887048 | IVW | C1QL1 | 1 |
| 0.073372949 | 0.226902258 | 0.746416497 | IVW | INHBC | 1 |
| 0.07200381 | 0.04411539 | 0.102643707 | IVW | ADGRF5 | 0.626325739 |
| 0.116495244 | 0.118014254 | 0.323579608 | IVW | OAF | 0.906997731 |
| 0.037251671 | 0.081396135 | 0.647197476 | IVW | CPN2 | 1 |
| -0.045616631 | 0.069652998 | 0.512523979 | IVW | GKN2 | 0.994044169 |
| -0.070997086 | 0.058054928 | 0.221356307 | IVW | MMP19 | 0.84001855 |
| -0.089358252 | 0.043742733 | 0.041070888 | IVW | PCYOX1 | 0.406898178 |
| -0.08912236 | 0.050797063 | 0.079348531 | IVW | FAM20A | 0.562759689 |
| 0.417841564 | 0.081413189 | 2.86E-07 | IVW | AOC2 | 1.37E-05 |
| 0.045440649 | 0.067168836 | 0.49871444 | IVW | PSG3 | 0.994044169 |
| -0.060484282 | 0.226467443 | 0.78940975 | IVW | SEMA3C | 1 |
| 0.1303201 | 0.057857621 | 0.024295265 | IVW | ASPN | 0.309241723 |
| -0.493123556 | 0.396619556 | 0.213751303 | Wald ratio | HAPLN4 | 0.829192218 |
| -0.165091613 | 0.106485353 | 0.121052926 | IVW | APOC3 | 0.672438506 |
| -0.033879445 | 0.074470101 | 0.649152237 | IVW | TIMP4 | 1 |
| 0.035213256 | 0.074740239 | 0.637539429 | IVW | POGLUT1 | 1 |
| 0.008938378 | 0.203686734 | 0.964997694 | IVW | FBLN1 | 1 |
| -0.028941707 | 0.035561442 | 0.415730448 | IVW | CFHR4 | 0.971782639 |
| -0.016370391 | 0.107056019 | 0.878465895 | IVW | PMEL | 1 |
| -0.185781791 | 0.155375108 | 0.231814219 | IVW | IGLON5 | 0.852052837 |
| 0.049402001 | 0.08125556 | 0.543198739 | IVW | IGLL1 | 0.994044169 |
| 0.275543155 | 0.430486607 | 0.522124687 | Wald ratio | CPZ | 0.994044169 |
| -0.125181704 | 0.118308787 | 0.290013028 | IVW | DLK1 | 0.884165194 |
| -0.061926839 | 0.079635308 | 0.43678692 | IVW | NCAM2 | 0.977304378 |
| -0.058015339 | 0.055248976 | 0.293685545 | IVW | MGP | 0.890321164 |
| 0.116422442 | 0.521356572 | 0.823296774 | IVW | NPTX2 | 1 |
| -0.031202895 | 0.262835965 | 0.905500178 | Wald ratio | DUSP13 | 1 |
| -0.439031096 | 0.057175486 | 1.61E-14 | IVW | TRIL | 1.19E-12 |
| -0.020492256 | 0.061520001 | 0.739059492 | IVW | NELL1 | 1 |
| -0.012393603 | 0.150473731 | 0.934357346 | IVW | ICAM4 | 1 |
| -0.063189044 | 0.033109657 | 0.056329494 | IVW | SERPINA12 | 0.472774182 |
| 0.015975257 | 0.046164091 | 0.729302322 | IVW | ENPP5 | 1 |
| 0.045737661 | 0.092961764 | 0.622715747 | IVW | LRRC15 | 1 |
| -0.299115243 | 0.979031467 | 0.759968935 | Wald ratio | COLEC10 | 1 |
| -0.003757734 | 0.55617603 | 0.994609233 | Wald ratio | LRRTM4 | 1 |
| -0.121263498 | 0.081679564 | 0.137643269 | IVW | FAIM3 | 0.701725102 |
| 0.037368907 | 0.053533479 | 0.485147547 | IVW | ART4 | 0.994044169 |
| 0.048858087 | 0.051436749 | 0.342179673 | IVW | PZP | 0.936346055 |
| -0.045196485 | 0.116466353 | 0.697968125 | IVW | IGFALS | 1 |
| 0.520899379 | 0.372009938 | 0.161444533 | Wald ratio | CNP | 0.756504336 |
| 0.138058939 | 0.262437459 | 0.598843636 | Wald ratio | FCRL6 | 1 |
| 0.34735644 | 0.170884751 | 0.042083429 | IVW | LINGO1 | 0.41084178 |
| 0.266130552 | 0.05914363 | 6.80E-06 | IVW | CHST12 | 0.000261541 |
| -0.095554384 | 0.01955804 | 1.03E-06 | IVW | PNLIPRP1 | 4.61E-05 |
| -0.18967031 | 0.153783752 | 0.217442497 | IVW | DEFB1 | 0.832728025 |
| 0.070132524 | 0.064914552 | 0.279972037 | IVW | NTN1 | 0.880697634 |
| 0.07699387 | 0.554477011 | 0.889561932 | Wald ratio | ALPPL2 | 1 |
| -0.052081184 | 0.065925586 | 0.429528029 | IVW | LRP11 | 0.971955213 |
| 0.086038096 | 0.107750339 | 0.424583352 | IVW | ALPPL2 | 0.971782639 |
| 0.151708665 | 0.09552877 | 0.112265087 | IVW | B3GAT3 | 0.651883677 |
| -0.286543962 | 0.360473623 | 0.426666058 | Wald ratio | LRRTM2 | 0.971782639 |
| -0.108144275 | 0.220248855 | 0.623419746 | Wald ratio | MGAT2 | 1 |
| -0.028981577 | 0.05916118 | 0.624222422 | IVW | HBZ | 1 |
| -0.076306048 | 0.130132979 | 0.55762704 | IVW | GFRAL | 0.994210735 |
| -0.183588239 | 0.107816897 | 0.088609625 | IVW | PLOD2 | 0.581254945 |
| 0.141661316 | 0.085956449 | 0.099340523 | IVW | SNX8 | 0.614808874 |
| 0.086916497 | 0.025341827 | 0.000604105 | IVW | NDST1 | 0.016587009 |
| -0.317051362 | 0.691716732 | 0.646697894 | Wald ratio | ST8SIA6 | 1 |
| 0.04530698 | 0.021341105 | 0.033754584 | IVW | ST3GAL6 | 0.358904016 |
| 0.196586832 | 0.203290892 | 0.333532872 | Wald ratio | SNX1 | 0.922316005 |
| -0.120287353 | 0.057363625 | 0.036000007 | IVW | CNTNAP2 | 0.370010771 |
| 0.644521368 | 0.514709402 | 0.2104954 | Wald ratio | WFDC5 | 0.829192218 |
| -0.049050973 | 0.232457744 | 0.832879251 | IVW | IGSF8 | 1 |
| -0.197934836 | 0.629436475 | 0.753168987 | Wald ratio | HS3ST3B1 | 1 |
| -0.11445411 | 0.143159219 | 0.424007372 | IVW | SDF2L1 | 0.971782639 |
| -0.195850452 | 0.102510885 | 0.056064593 | IVW | ASPH | 0.472774182 |
| 0.868285676 | 1.028856235 | 0.398706875 | Wald ratio | GALNT10 | 0.963917753 |
| 0.700691742 | 0.309946672 | 0.02377878 | IVW | CD72 | 0.309241723 |
| 0.002945172 | 0.034157842 | 0.931289608 | IVW | LILRB5 | 1 |
| 0.179027765 | 0.084426959 | 0.033963504 | IVW | SEMA7A | 0.358904016 |
| -0.00898751 | 0.043630361 | 0.836797071 | IVW | ADAM23 | 1 |
| 0.27673602 | 0.235241776 | 0.239439157 | Wald ratio | NEGR1 | 0.857406393 |
| -0.024091518 | 0.023869651 | 0.312833199 | IVW | LILRA6 | 0.894057446 |
| -0.592419173 | 0.718023162 | 0.409332007 | Wald ratio | G6B | 0.971782639 |
| 0.169269797 | 0.2065272 | 0.412443887 | IVW | MATN4 | 0.971782639 |
| 0.048317374 | 0.410559724 | 0.906315992 | Wald ratio | SYT11 | 1 |
| -0.037520939 | 0.073202454 | 0.608256381 | IVW | RMDN1 | 1 |
| 0.311788536 | 0.117970836 | 0.008219228 | IVW | DNAJB11 | 0.150451017 |
| -0.215550106 | 0.118829373 | 0.069685987 | IVW | VWA2 | 0.52939315 |
| 0.025424174 | 0.406944253 | 0.950183922 | Wald ratio | CELA2A | 1 |
| -0.061182136 | 0.209405968 | 0.770156669 | IVW | MGAT4B | 1 |
| 0.066963779 | 0.050510437 | 0.184925357 | IVW | ITIH3 | 0.795137666 |
| -0.025577481 | 0.044123852 | 0.562134004 | IVW | FUT10 | 0.998143017 |
| 0.030157636 | 0.04706511 | 0.521675912 | IVW | H6PD | 0.994044169 |
| -0.064410633 | 0.08224808 | 0.433552961 | IVW | TMEM132C | 0.972817562 |
| -0.077418355 | 0.041205433 | 0.060266544 | IVW | NFASC | 0.488743868 |
| 0.098764282 | 0.174566289 | 0.571550485 | IVW | GP5 | 1 |
| 0.049514528 | 0.10685373 | 0.643087676 | IVW | IFNLR1 | 1 |
| -0.007859314 | 0.044704762 | 0.860447266 | IVW | FAM20B | 1 |
| 0.381863587 | 0.119445546 | 0.001388803 | IVW | FBP1 | 0.034665976 |
| -0.605246143 | 0.973846436 | 0.534270301 | Wald ratio | APLP1 | 0.994044169 |
| -0.055181598 | 0.08022772 | 0.491571101 | IVW | RNASE1 | 0.994044169 |
| 0.022271177 | 0.099952015 | 0.823676621 | IVW | ATP1B2 | 1 |
| -0.048947432 | 0.110410367 | 0.657532272 | IVW | COCH | 1 |
| -0.034639988 | 0.380468901 | 0.927456424 | IVW | ST6GALNAC6 | 1 |
| -0.078172784 | 0.054321007 | 0.1501252 | IVW | IGFLR1 | 0.734200088 |
| -0.10573223 | 0.067894441 | 0.119398516 | IVW | C1QTNF3 | 0.669395967 |
| -0.645689085 | 0.318983721 | 0.042948857 | Wald ratio | PRG2 | 0.413729933 |
| 0.200676551 | 0.230875716 | 0.38473922 | IVW | SERPINA9 | 0.957360905 |
| 0.039793893 | 0.614415574 | 0.948359465 | IVW | LRRC32 | 1 |
| 0.02671176 | 0.039706568 | 0.501119717 | IVW | CRELD1 | 0.994044169 |
| -0.724933271 | 0.360589286 | 0.04438753 | Wald ratio | MYBPC1 | 0.41820016 |
| 0.001481497 | 0.1249111 | 0.990536981 | IVW | NPPB | 1 |
| 0.077543164 | 0.260919244 | 0.766319778 | IVW | CHKB | 1 |
| -0.038057069 | 0.024926293 | 0.126814694 | IVW | SH3BP2 | 0.679923933 |
| -1.389790291 | 0.61263301 | 0.023295538 | Wald ratio | KIR2DL2 | 0.308411198 |
| 0.131867266 | 0.193958448 | 0.496584377 | IVW | CHST11 | 0.994044169 |
| 0.017246683 | 0.053519835 | 0.7472645 | IVW | KNG1 | 1 |
| 0.070385568 | 0.044091605 | 0.110411006 | IVW | LILRA5 | 0.645015054 |
| 0.225825734 | 0.350064288 | 0.518863749 | IVW | B4GALT7 | 0.994044169 |
| -0.052286656 | 0.095672581 | 0.584710863 | IVW | GLCE | 1 |
| 0.092900943 | 0.057699416 | 0.107379378 | IVW | C1QTNF5 | 0.63502512 |
| 0.017037739 | 0.589649425 | 0.976948578 | Wald ratio | HRASLS2 | 1 |
| 0.165321673 | 0.106009989 | 0.118880103 | IVW | ESAM | 0.669395967 |
| 0.050180805 | 0.110176378 | 0.648779176 | IVW | QPCT | 1 |
| -0.00519795 | 0.086288227 | 0.951964978 | IVW | FAM151A | 1 |
| 0.338323372 | 0.164565724 | 0.039796433 | IVW | ROR2 | 0.396314734 |
| 0.034999184 | 0.05920356 | 0.554408604 | IVW | DNAJC30 | 0.994044169 |
| 0.225528544 | 0.079204345 | 0.004407428 | IVW | TMEM132A | 0.096262241 |
| -0.227560876 | 0.049257462 | 3.84E-06 | IVW | PLEK | 0.000157057 |
| -0.009664136 | 0.153162299 | 0.94968898 | IVW | UGT1A6 | 1 |
| -0.047079246 | 0.051410203 | 0.359794441 | IVW | HP | 0.93822612 |
| -0.056495013 | 0.057566108 | 0.326398655 | IVW | S100A7 | 0.911828802 |
| 0.081980533 | 0.055052243 | 0.136450333 | IVW | AMY1A | 0.700872481 |
| -0.106595278 | 0.034060727 | 0.00175065 | IVW | FJX1 | 0.043137819 |
| -0.043614863 | 0.139360758 | 0.754308216 | IVW | SEMA4C | 1 |
| 0.059964019 | 0.16855532 | 0.722025737 | IVW | SPINT3 | 1 |
| 0.124327536 | 0.062973338 | 0.048348949 | IVW | TPST1 | 0.436313333 |
| -0.139776654 | 0.167813836 | 0.404885971 | IVW | ADAM22 | 0.96789905 |
| 0.021879718 | 0.116310489 | 0.850786702 | IVW | LARGE | 1 |
| -0.070579518 | 0.375075699 | 0.850740206 | IVW | SEMA6A | 1 |
| 0.472738971 | 0.432035386 | 0.273861333 | Wald ratio | ENTHD2 | 0.880697634 |
| 0.008866293 | 0.072751644 | 0.903001434 | IVW | GLTPD2 | 1 |
| -0.243698548 | 1.938841658 | 0.899975056 | Wald ratio | BTNL9 | 1 |
| -0.055190507 | 0.068019729 | 0.417141911 | IVW | ITIH1 | 0.971782639 |
| -0.029135367 | 0.077018569 | 0.705215058 | IVW | SCG3 | 1 |
| -0.13111217 | 0.076539027 | 0.086710579 | IVW | CRTAM | 0.576254043 |
| 0.001566077 | 0.049518383 | 0.974770165 | IVW | ART3 | 1 |
| 0.167733529 | 0.114731416 | 0.143750291 | IVW | B3GNT2 | 0.723094254 |
| 0.523858156 | 0.559073759 | 0.348752997 | Wald ratio | B3GALT6 | 0.936346055 |
| -0.132749416 | 0.439317643 | 0.762521077 | IVW | ERO1LB | 1 |
| 1.095333333 | 0.661474359 | 0.097742702 | Wald ratio | DOC2B | 0.613926381 |
| 0.029403218 | 0.227074582 | 0.896972269 | IVW | ENTPD1 | 1 |
| 0.014483689 | 0.022390544 | 0.517718468 | IVW | KIR2DL5A | 0.994044169 |
| 0.523116371 | 2.73169907 | 0.848135028 | Wald ratio | GAPDHS | 1 |
| -0.138168908 | 0.085273408 | 0.105166828 | IVW | MXRA7 | 0.627572467 |
| 0.187420168 | 0.343516206 | 0.585345476 | Wald ratio | DNAJB12 | 1 |
| 0.017845749 | 0.070328036 | 0.799688596 | IVW | CTSB | 1 |
| -0.003563392 | 0.112479912 | 0.974727043 | IVW | LMAN2L | 1 |
| 0.106050117 | 0.029843908 | 0.000380151 | IVW | MANEA | 0.010744866 |
| -0.212776533 | 0.015572431 | 1.67E-42 | IVW | TPST2 | 2.47E-40 |
| -0.261610889 | 0.23653803 | 0.268726836 | Wald ratio | SPINK5 | 0.880697634 |
| -0.392736439 | 0.282020862 | 0.16374702 | Wald ratio | C1orf198 | 0.760307047 |
| -0.01972416 | 0.065161832 | 0.762122321 | IVW | FAM177A1 | 1 |
| 0.040942565 | 0.091301776 | 0.653842032 | IVW | SPINK9 | 1 |
| 0.082630177 | 0.131734516 | 0.530496853 | IVW | COMP | 0.994044169 |
| -0.51898093 | 0.479221692 | 0.278823394 | Wald ratio | DNAJB14 | 0.880697634 |
| -0.035332047 | 0.240225197 | 0.883069778 | Wald ratio | PATE4 | 1 |
| 0.018656323 | 0.240767055 | 0.938236113 | Wald ratio | KIAA1161 | 1 |
| -0.390399038 | 0.460633013 | 0.396701295 | Wald ratio | MSMP | 0.962009196 |
| 0.048511164 | 0.079571315 | 0.542089427 | IVW | LIPN | 0.994044169 |
| 0.035846158 | 0.099450901 | 0.718517553 | IVW | SPON2 | 1 |
| -0.445859732 | 0.250681208 | 0.075306585 | Wald ratio | ADM2 | 0.550638249 |
| 0.238435762 | 0.215439131 | 0.268405036 | Wald ratio | ZG16 | 0.880697634 |
| -0.19738653 | 0.433919473 | 0.649186884 | Wald ratio | MIF | 1 |
| 0.18846787 | 0.247374616 | 0.446136218 | IVW | EPHB2 | 0.983911145 |
| 0.053333619 | 0.057538629 | 0.353968858 | IVW | GXYLT1 | 0.936346055 |
| -0.322937705 | 0.070626576 | 4.82E-06 | IVW | FLT1 | 0.000189068 |
| -0.096371171 | 0.0736387 | 0.190635053 | IVW | ITIH5 | 0.804842439 |
| 0.371813435 | 0.342466411 | 0.277614774 | Wald ratio | CHGB | 0.880697634 |
| -0.316860547 | 0.274434245 | 0.248256107 | Wald ratio | SPINK1 | 0.870512524 |
| -0.032152364 | 0.038485955 | 0.403475025 | IVW | FUT8 | 0.967123738 |
| 0.06605553 | 0.065752831 | 0.31508777 | IVW | ICAM5 | 0.895856055 |
| -0.06835068 | 0.028907179 | 0.018055007 | IVW | SIGLEC14 | 0.262891844 |
| -0.048538629 | 0.204251343 | 0.812159033 | IVW | PTPRJ | 1 |
| -0.047254469 | 0.162263866 | 0.770883136 | IVW | NOTUM | 1 |
| 0.12884102 | 0.237331188 | 0.587216303 | IVW | MRVI1 | 1 |
| -0.065844748 | 0.271647363 | 0.808477572 | IVW | UXS1 | 1 |
| 0.023939689 | 0.241827548 | 0.921142337 | IVW | ARSK | 1 |
| 0.275556789 | 0.243923997 | 0.258609829 | Wald ratio | STX7 | 0.875138353 |
| 0.040112757 | 0.116303712 | 0.73017209 | IVW | PEAR1 | 1 |
| 0.023862687 | 0.106810352 | 0.823215067 | IVW | APOH | 1 |
| 0.073574233 | 0.095086677 | 0.439072945 | IVW | GPNMB | 0.97738713 |
| 0.037342914 | 0.076492902 | 0.625416707 | IVW | KDELC2 | 1 |
| 0.499644605 | 0.43971062 | 0.255829673 | IVW | DNAJC10 | 0.875138353 |
| -0.039815977 | 0.052235336 | 0.445915473 | IVW | LILRA4 | 0.983911145 |
| 0.078664244 | 0.560390963 | 0.888364609 | Wald ratio | PEX14 | 1 |
| 0.080144088 | 0.125424127 | 0.52283294 | IVW | TNFRSF11B | 0.994044169 |
| -0.112605467 | 0.104611417 | 0.281740977 | IVW | HYAL1 | 0.882777802 |
| 0.144429268 | 0.161595122 | 0.371443652 | Wald ratio | ZG16B | 0.950618773 |
| 0.425815485 | 0.152068457 | 0.005107783 | IVW | TFF3 | 0.10788087 |
| 2.405659091 | 1.664890909 | 0.148476153 | Wald ratio | ADH4 | 0.731831718 |
| -0.038785663 | 0.107198059 | 0.717491697 | IVW | DPEP2 | 1 |
| -0.013686707 | 0.032792689 | 0.67640729 | IVW | GRAMD1C | 1 |
| -0.09550938 | 0.020787028 | 4.33E-06 | IVW | PTPRU | 0.000173561 |
| -0.050794097 | 0.059231873 | 0.39114282 | IVW | GPX7 | 0.960408371 |
| -0.172644669 | 0.071470918 | 0.015709599 | IVW | DPP7 | 0.24155079 |
| 0.077271451 | 0.092407342 | 0.403039775 | IVW | PRSS57 | 0.967123738 |
| -0.015079753 | 0.09350234 | 0.871875441 | IVW | OXT | 1 |
| -0.188590628 | 0.078319664 | 0.016041933 | IVW | NCR1 | 0.244703138 |
| -0.039765229 | 0.073961539 | 0.5908205 | IVW | UST | 1 |
| 0.04107804 | 0.149667178 | 0.783729156 | IVW | TNFRSF1B | 1 |
| -0.010714401 | 0.134023552 | 0.936281663 | IVW | LHB | 1 |
| 0.125020266 | 0.199075472 | 0.530001079 | Wald ratio | RNASE2 | 0.994044169 |
| 0.023112187 | 0.055816365 | 0.678818849 | IVW | QSOX2 | 1 |
| -0.030474911 | 0.017759048 | 0.086158078 | IVW | RARRES1 | 0.576254043 |
| -0.038321878 | 0.083145419 | 0.644869016 | IVW | CYTL1 | 1 |
| 0.035860459 | 0.109560896 | 0.743432861 | IVW | RSPO3 | 1 |
| -0.02162979 | 0.047279664 | 0.647321676 | IVW | NTM | 1 |
| 0.074121219 | 0.204329053 | 0.716788521 | Wald ratio | GHRL | 1 |
| -0.699934924 | 0.626585683 | 0.263967907 | Wald ratio | SNCA | 0.879481929 |
| -0.062812539 | 0.165793284 | 0.704791401 | IVW | RSPO4 | 1 |
| -0.012524656 | 0.02586554 | 0.628228525 | IVW | CTSH | 1 |
| -0.94245902 | 0.408501922 | 0.021048668 | IVW | KLK3 | 0.286918722 |
| 0.96053791 | 0.852742095 | 0.259991675 | IVW | IGFBP2 | 0.875138353 |
| -0.15094675 | 0.040577431 | 0.000199248 | IVW | CHGA | 0.006078642 |
| -0.020470982 | 0.047685749 | 0.66771271 | IVW | MMP10 | 1 |
| 0.351029373 | 0.032120664 | 8.43E-28 | IVW | EFEMP1 | 8.52E-26 |
| -0.064346645 | 0.056107207 | 0.251442925 | IVW | GPNMB | 0.870512524 |
| 0.771911483 | 0.705453748 | 0.27386479 | Wald ratio | G6B | 0.880697634 |
| -0.402255292 | 0.266993364 | 0.13191025 | IVW | OLFML3 | 0.690821526 |
| 0.427679558 | 0.466827151 | 0.359592873 | Wald ratio | PKDCC | 0.93822612 |
| 0.317143369 | 0.268744624 | 0.237963643 | Wald ratio | TMEM106B | 0.857406393 |
| -0.129222016 | 0.055709317 | 0.020363804 | IVW | GPC1 | 0.285285283 |
| 0.052483849 | 0.106040918 | 0.620642759 | IVW | A4GALT | 1 |
| 0.068343778 | 0.051077844 | 0.180886083 | IVW | LILRA5 | 0.79153876 |
| -0.83360746 | 0.541394316 | 0.123623303 | Wald ratio | BCL10 | 0.67523297 |
| -0.022549119 | 0.072718031 | 0.756492681 | IVW | EMILIN3 | 1 |
| -0.096648649 | 0.085492502 | 0.258268679 | IVW | NOG | 0.875138353 |
| -0.315240214 | 0.204321716 | 0.122864258 | Wald ratio | FAM171B | 0.67523297 |
| 0.03686303 | 0.071745415 | 0.607389534 | IVW | DPEP1 | 1 |
| 0.256450723 | 0.411494087 | 0.533140954 | Wald ratio | TFRC | 0.994044169 |
| -0.219640732 | 0.093198998 | 0.018438853 | IVW | PSAPL1 | 0.264473694 |
| -0.02336057 | 0.042961329 | 0.58660796 | IVW | C1S | 1 |
| -0.165356213 | 0.44027924 | 0.707235687 | IVW | CILP2 | 1 |
| 0.008337842 | 0.064596728 | 0.89729807 | IVW | GRAMD1C | 1 |
| 0.300919912 | 0.207410861 | 0.146824501 | Wald ratio | CA11 | 0.731079512 |
| 0.036277717 | 0.07920639 | 0.646941644 | IVW | QPCTL | 1 |
| -0.276331996 | 0.139201602 | 0.047131033 | IVW | CLN5 | 0.430852421 |
| 0.384253602 | 0.415556196 | 0.355136018 | Wald ratio | EVA1C | 0.936346055 |
| 0.080072819 | 0.430462231 | 0.852432293 | IVW | CACNA2D3 | 1 |
| 0.153297455 | 0.08071444 | 0.057530708 | IVW | TMEM132B | 0.476612162 |
| -1.54997076 | 0.585640351 | 0.00812993 | Wald ratio | PEAR1 | 0.150451017 |
| 0.582332344 | 0.576259644 | 0.31223754 | Wald ratio | PARP1 | 0.894057446 |
| -0.361755874 | 0.26548323 | 0.172998602 | IVW | HNRNPAB | 0.776876898 |
| 1.749761995 | 1.347793729 | 0.1942043 | Wald ratio | NEO1 | 0.807701915 |
| 0.041525131 | 0.308233438 | 0.892833458 | Wald ratio | STIM1 | 1 |
| 0.012131033 | 0.095066588 | 0.898461065 | IVW | GALNT16 | 1 |
| -0.108899958 | 0.193902095 | 0.574372617 | IVW | RRM2B | 1 |
| -0.056383558 | 0.280703335 | 0.840803907 | Wald ratio | ENTPD6 | 1 |
| 0.48075502 | 0.400821954 | 0.230363558 | Wald ratio | HDGF | 0.850634127 |
| -0.353119537 | 0.236019923 | 0.134616415 | Wald ratio | SCARF2 | 0.700650694 |
| -0.107103689 | 0.361686939 | 0.76713643 | Wald ratio | ERLEC1 | 1 |
| 0.080724957 | 0.079586755 | 0.310438956 | IVW | CHL1 | 0.894057446 |
| 0.026632726 | 0.036597295 | 0.466782626 | IVW | ERAP2 | 0.994044169 |
| 1.519041435 | 1.752096475 | 0.385950206 | Wald ratio | VTI1B | 0.957360905 |
| 0.170908811 | 0.121228932 | 0.158598106 | IVW | CD14 | 0.754854981 |
| 0.311511848 | 0.439526066 | 0.47848281 | Wald ratio | RIPK2 | 0.994044169 |
| -6.50E-05 | 0.032014748 | 0.998379052 | IVW | FCRL4 | 1 |
| -0.060993356 | 0.123930745 | 0.622608517 | IVW | COL15A1 | 1 |
| -0.099605086 | 0.185782688 | 0.591863451 | IVW | THBS3 | 1 |
| -0.308397281 | 0.152726624 | 0.043458491 | IVW | GOLM1 | 0.413729933 |
| 0.028765824 | 0.17837059 | 0.871880754 | IVW | SCUBE1 | 1 |
| -0.042839072 | 0.096205844 | 0.656113411 | IVW | TMEM2 | 1 |
| -0.132790533 | 0.084909393 | 0.117839026 | IVW | SERPINA11 | 0.669395967 |
| 0.024245682 | 0.159896701 | 0.879476079 | IVW | PLXNA1 | 1 |
| -0.155478009 | 0.392521991 | 0.692031167 | Wald ratio | PRG3 | 1 |
| 0.00512462 | 0.027635284 | 0.852885831 | IVW | LCT | 1 |
| -0.038388344 | 0.397284786 | 0.923022799 | IVW | PCDH10 | 1 |
| -0.038336041 | 0.089248762 | 0.667529251 | IVW | HAVCR1 | 1 |
| 0.630940193 | 0.388047493 | 0.103963378 | Wald ratio | BTNL8 | 0.626325739 |
| 0.784640565 | 2.065706033 | 0.704063187 | Wald ratio | CRIP2 | 1 |
| 0.009503529 | 0.056314655 | 0.865987268 | IVW | PENK | 1 |
| 0.051714443 | 0.060523941 | 0.39285788 | IVW | MAN1A2 | 0.960408371 |
| -0.173096878 | 0.234075044 | 0.459607634 | IVW | ANGPTL1 | 0.993644616 |
| -0.007557907 | 0.052882985 | 0.886355289 | IVW | CLEC4C | 1 |
| -0.001033724 | 0.038216056 | 0.978420274 | IVW | NT5DC3 | 1 |
| 0.282751463 | 0.129014893 | 0.028407045 | IVW | MMP8 | 0.326936167 |
| 0.160615428 | 0.23861228 | 0.500869005 | IVW | PGM1 | 0.994044169 |
| -0.004386159 | 0.173367432 | 0.979815847 | IVW | DSCAM | 1 |
| -0.054183366 | 0.054249576 | 0.317901497 | IVW | FAM3B | 0.898539232 |
| -0.020536882 | 0.059630713 | 0.730544672 | IVW | IFNAR1 | 1 |
| 0.195073891 | 0.119565894 | 0.102781097 | IVW | TFF1 | 0.626325739 |
| -0.132121867 | 0.422148 | 0.754299482 | Wald ratio | CXCL9 | 1 |
| 0.030550901 | 0.104921028 | 0.770913688 | IVW | TFF2 | 1 |
| -0.128554825 | 0.247640351 | 0.603677722 | Wald ratio | LGALS9 | 1 |
| 0.789092672 | 0.30790625 | 0.010384103 | Wald ratio | TAGLN2 | 0.17507234 |
| 0.017631362 | 0.287602385 | 0.951116592 | IVW | POMC | 1 |
| -0.095525736 | 0.040399843 | 0.018054057 | IVW | SERPINF1 | 0.262891844 |
| -0.1534178 | 0.176985373 | 0.386030304 | IVW | CTSF | 0.957360905 |
| 0.177474012 | 0.051849463 | 0.000619635 | IVW | FTCD | 0.016773789 |
| 0.036386772 | 0.089845777 | 0.685483793 | IVW | PLXNB2 | 1 |
| 0.449754808 | 0.506496394 | 0.37455561 | Wald ratio | MANF | 0.950951101 |
| 0.004672166 | 0.062193726 | 0.940117021 | IVW | IMPAD1 | 1 |
| 0.600156107 | 0.475380165 | 0.206777514 | Wald ratio | TFPI2 | 0.821672375 |
| -0.269078799 | 0.006556413 | 0 | IVW | TWSG1 | 0 |
| -0.031476222 | 0.204343461 | 0.877581456 | Wald ratio | SIRPG | 1 |
| -0.045187778 | 0.027430302 | 0.099482601 | IVW | PPT1 | 0.614808874 |
| -1.613978659 | 0.439442073 | 0.000239916 | Wald ratio | TMEM9 | 0.007094137 |
| -0.021420465 | 0.052412987 | 0.682769954 | IVW | MAN2B2 | 1 |
| 0.020894223 | 0.032170351 | 0.516023679 | IVW | ABO | 0.994044169 |
| -0.101329907 | 0.088372005 | 0.25153497 | IVW | NPTX1 | 0.870512524 |
| 1.548139754 | 0.711076915 | 0.029467421 | IVW | TACO1 | 0.334035426 |
| -0.078467009 | 0.023928694 | 0.001041013 | IVW | SIAE | 0.02632666 |
| -0.074196679 | 0.316338257 | 0.814559147 | IVW | CTSO | 1 |
| -0.603685681 | 0.346613504 | 0.081566539 | Wald ratio | GLIPR1 | 0.563075399 |
| 0.051146628 | 0.104931459 | 0.625954401 | IVW | TREM1 | 1 |
| -0.079965603 | 0.028615349 | 0.005197999 | IVW | CPA4 | 0.108584976 |
| -0.105351015 | 0.142625429 | 0.460116393 | IVW | CRISP2 | 0.993644616 |
| -0.127812417 | 0.097656869 | 0.190605207 | IVW | FKBP7 | 0.804842439 |
| -0.158416018 | 0.359562257 | 0.659516925 | Wald ratio | MFAP2 | 1 |
| 0.028291855 | 0.054936095 | 0.606555991 | IVW | B3GNT8 | 1 |
| -0.029879065 | 0.012497101 | 0.016808059 | IVW | AZGP1 | 0.251402742 |
| 0.014200001 | 0.057221138 | 0.804010572 | IVW | CBLN1 | 1 |
| 0.069100171 | 0.082676847 | 0.403275168 | IVW | PSG5 | 0.967123738 |
| -0.048128353 | 0.056925514 | 0.397852996 | IVW | WFDC1 | 0.963064808 |
| 0.009783833 | 0.102583276 | 0.924017339 | IVW | NMB | 1 |
| -0.026901268 | 0.217355043 | 0.901500175 | Wald ratio | RCN1 | 1 |
| 0.044672992 | 0.046792545 | 0.339727813 | IVW | ITIH2 | 0.931464844 |
| -0.153999691 | 0.40254022 | 0.702038841 | IVW | TREML1 | 1 |
| -0.017556869 | 0.16874779 | 0.917135965 | IVW | PSG9 | 1 |
| 0.031670446 | 0.13626672 | 0.816215604 | IVW | TAC1 | 1 |
| -0.296503513 | 0.648775176 | 0.647656358 | Wald ratio | FKBP2 | 1 |
| 0.058993662 | 0.097263366 | 0.544159367 | IVW | PDGFD | 0.994044169 |
| 0.141832645 | 0.295321281 | 0.631038564 | Wald ratio | IL2RB | 1 |
| -0.072766088 | 0.058682035 | 0.214973096 | IVW | C1RL | 0.829192218 |
| 0.079875 | 0.614985294 | 0.896660455 | Wald ratio | FSTL4 | 1 |
| 0.051477531 | 0.308821565 | 0.867613809 | IVW | CREG1 | 1 |
| -0.07892316 | 0.200711039 | 0.694158598 | Wald ratio | DLK2 | 1 |
| 0.127016116 | 0.121690054 | 0.296593001 | IVW | EDIL3 | 0.894057446 |
| 0.426808911 | 0.303445764 | 0.159563536 | IVW | LRRC4C | 0.75537221 |
| 0.006803745 | 0.081779315 | 0.933695375 | IVW | GGH | 1 |
| 0.322913537 | 0.431289366 | 0.454027996 | IVW | PLA2G12B | 0.992816625 |
| 0.897588511 | 0.529688159 | 0.090158869 | Wald ratio | CHI3L2 | 0.584540565 |
| 0.319326168 | 0.314805607 | 0.310411076 | Wald ratio | CAMP | 0.894057446 |
| 0.010272875 | 0.058147104 | 0.859767253 | IVW | GAA | 1 |
| -1.003292476 | 0.569463592 | 0.078099676 | Wald ratio | MCEE | 0.5601029 |
| 0.107379635 | 0.070945103 | 0.130137599 | IVW | CPQ | 0.687155127 |
| 0.299615588 | 0.474187583 | 0.527484672 | Wald ratio | GALP | 0.994044169 |
| -0.011221358 | 0.026934717 | 0.676961384 | IVW | TPSAB1 | 1 |
| -0.101871122 | 0.114554696 | 0.373852985 | IVW | CPM | 0.950951101 |
| 0.148306856 | 0.058897806 | 0.011801091 | IVW | C4BPA | 0.193860662 |
| -0.008117139 | 0.086296121 | 0.92506034 | IVW | FAS | 1 |
| 0.05953564 | 0.216034832 | 0.782867968 | IVW | METTL24 | 1 |
| 0.032019444 | 0.369823045 | 0.931005009 | Wald ratio | PRPSAP1 | 1 |
| -0.607949695 | 0.684488516 | 0.374443397 | IVW | NUDT9 | 0.950951101 |
| -0.062420946 | 0.103263593 | 0.545523476 | IVW | DSG2 | 0.994044169 |
| 0.303650204 | 0.577005158 | 0.598713026 | IVW | PTK7 | 1 |
| 0.130862021 | 0.199996516 | 0.512904695 | Wald ratio | GZMK | 0.994044169 |
| 0.03818432 | 0.037223245 | 0.304976809 | IVW | MANSC1 | 0.894057446 |
| -0.303876034 | 0.079580355 | 0.000134276 | IVW | BIN1 | 0.004230801 |
| 0.046114732 | 0.089037234 | 0.604509637 | IVW | MANSC4 | 1 |
| 0.050228608 | 0.081236526 | 0.53637708 | IVW | LAMC2 | 0.994044169 |
| 0.196813045 | 0.053022287 | 0.000205715 | IVW | B4GALT2 | 0.006177882 |
| 0.126303816 | 0.290554032 | 0.663780253 | IVW | PIANP | 1 |
| -0.518385266 | 0.417018116 | 0.213839781 | Wald ratio | TIGIT | 0.829192218 |
| -0.112678679 | 0.070199489 | 0.108467163 | IVW | PDGFRL | 0.637742829 |
| 0.042373059 | 0.186307048 | 0.820084189 | IVW | IGSF3 | 1 |
| -0.27286123 | 0.440281831 | 0.535427528 | Wald ratio | MMP16 | 0.994044169 |
| 0.458034833 | 0.4218418 | 0.277568528 | Wald ratio | DNAJA4 | 0.880697634 |
| 0.785475 | 0.5612875 | 0.161688116 | Wald ratio | MARK3 | 0.756504336 |
| 0.057157835 | 0.044373034 | 0.197703856 | IVW | GSTM3 | 0.81076487 |
| 0.03620391 | 0.04217773 | 0.390690644 | IVW | NQO2 | 0.960408371 |
| 0.016469299 | 0.531348718 | 0.975273309 | Wald ratio | IDO1 | 1 |
| 0.119629606 | 0.419748412 | 0.775641753 | Wald ratio | NDE1 | 1 |
| -0.056410803 | 0.104644969 | 0.589839612 | IVW | DNER | 1 |
| -0.222757825 | 0.556850437 | 0.689133172 | IVW | NLGN2 | 1 |
| 0.017212153 | 0.112726533 | 0.878643175 | IVW | BRSK2 | 1 |
| 0.028608752 | 0.053692335 | 0.594153 | IVW | IGDCC4 | 1 |
| 0.00569422 | 0.184928474 | 0.975435842 | IVW | CEL | 1 |
| -0.281739439 | 0.101971237 | 0.005728497 | IVW | ISOC1 | 0.115896531 |
| 0.088384593 | 0.381200403 | 0.816647838 | IVW | FHIT | 1 |
| -0.155319061 | 0.656490331 | 0.812974863 | Wald ratio | LDHC | 1 |
| 0.144581483 | 0.190096363 | 0.446914282 | Wald ratio | SULT2A1 | 0.983928121 |
| -0.09545034 | 0.424131263 | 0.821941113 | IVW | HGD | 1 |
| -0.341991701 | 0.468045182 | 0.46497405 | Wald ratio | ADH1B | 0.994044169 |
| 0.11287798 | 0.048861313 | 0.020878698 | IVW | NQO1 | 0.286918722 |
| -0.934352 | 0.577148 | 0.105466133 | Wald ratio | SMAD1 | 0.627572467 |
| -0.130278301 | 0.127053423 | 0.30518295 | IVW | TIRAP | 0.894057446 |
| -0.58645379 | 0.496301142 | 0.237344969 | Wald ratio | PAICS | 0.857406393 |
| -0.097528868 | 0.342524403 | 0.775846766 | Wald ratio | CCNH | 1 |
| -0.696668033 | 0.683831967 | 0.308311797 | Wald ratio | TPM4 | 0.894057446 |
| -0.023488494 | 0.016264034 | 0.148683126 | IVW | FBP2 | 0.731831718 |
| 0.287945205 | 0.451054795 | 0.523225127 | Wald ratio | NFKB1 | 0.994044169 |
| 0.082665563 | 0.087910775 | 0.347045844 | IVW | WARS | 0.936346055 |
| 0.05712664 | 0.124087774 | 0.645248631 | IVW | ALDOC | 1 |
| 1.505420262 | 0.912122084 | 0.098849057 | Wald ratio | CRKL | 0.614808874 |
| 0.075802031 | 0.113301902 | 0.503478256 | IVW | GLO1 | 0.994044169 |
| -0.017004123 | 0.0662348 | 0.797391137 | IVW | PPIL1 | 1 |
| 0.028462419 | 0.236717815 | 0.90429481 | Wald ratio | XRCC4 | 1 |
| 1.673355049 | 1.175982628 | 0.154752975 | Wald ratio | MYO6 | 0.751099036 |
| -0.110040594 | 0.729572309 | 0.880110682 | Wald ratio | EGLN1 | 1 |
| 0.043694193 | 0.069200464 | 0.527769385 | IVW | SPOCK3 | 0.994044169 |
| 0.156143396 | 0.139797922 | 0.264027613 | IVW | LRRC4B | 0.879481929 |
| 0.051508547 | 0.428038798 | 0.904216857 | IVW | KRT1 | 1 |
| 0.072521997 | 0.157078891 | 0.644302529 | IVW | DUSP28 | 1 |
| -0.056160896 | 0.800910978 | 0.944097137 | Wald ratio | NXT1 | 1 |
| 0.018553776 | 0.177114661 | 0.916569635 | IVW | LAG3 | 1 |
| -0.871128205 | 0.540578348 | 0.107076348 | Wald ratio | CDHR5 | 0.63502512 |
| 0.066418855 | 0.44397905 | 0.881080939 | Wald ratio | MYZAP | 1 |
| 0.036733174 | 0.097104948 | 0.7052202 | IVW | KIAA1467 | 1 |
| 0.0445197 | 0.153303281 | 0.771508111 | IVW | NPW | 1 |
| 0.560311961 | 0.495430333 | 0.258071867 | Wald ratio | DUT | 0.875138353 |
