# Supplementary figures and images for: Blood metabolites as mediators in erectile dysfunction: insights from a multi-center proteomics and genetic study
Source: Front Pharmacol. 2025 Jun 2;16:1568780. doi: 10.3389/fphar.2025.1568780 (PMC12171135; doi:10.3389/fphar.2025.1568780)

AMN


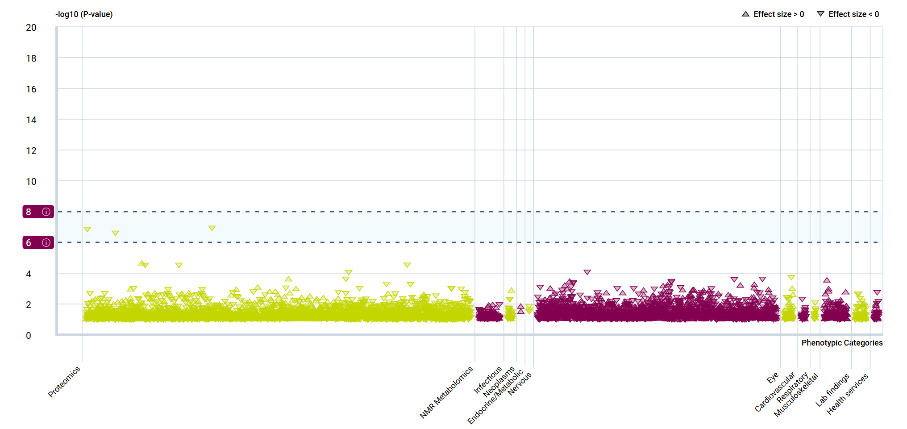


ESM1


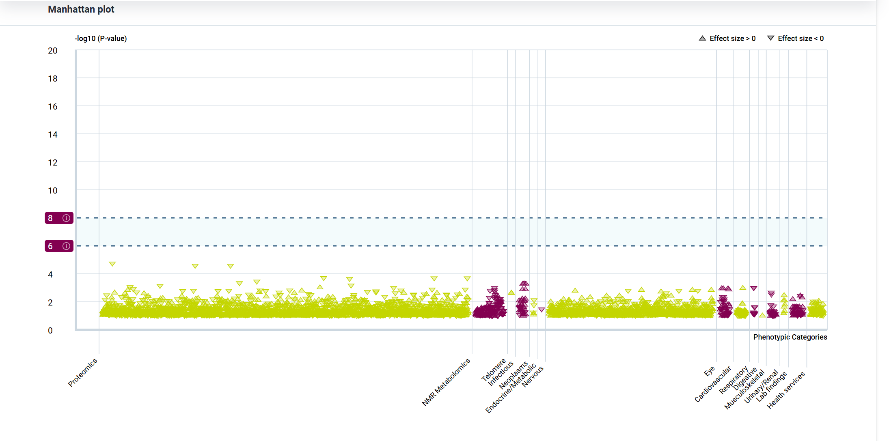


PIGR


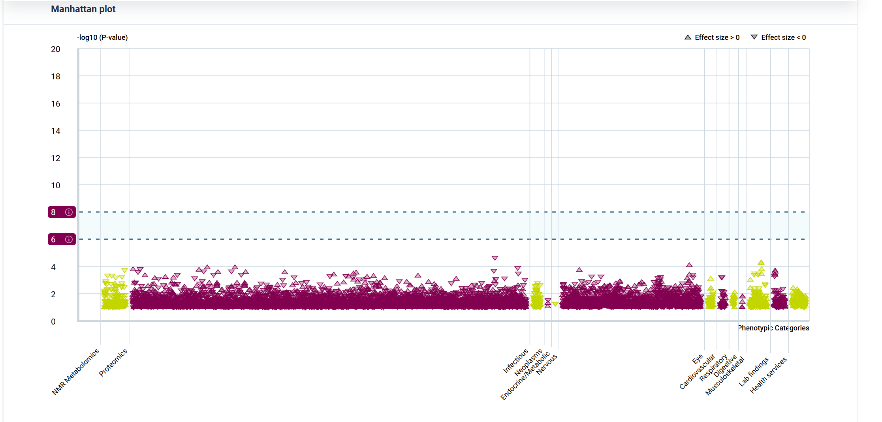


TNFRSF6B


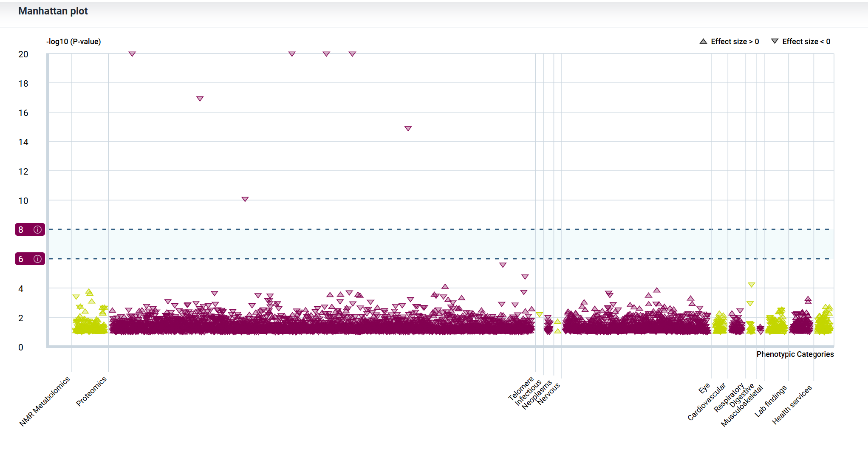

Supplement: Supplementary file 2 [file Supplementaryfile5.docx]
