# Supplementary material for: Blood metabolites as mediators in erectile dysfunction: insights from a multi-center proteomics and genetic study
Source: Front Pharmacol. 2025 Jun 2;16:1568780. doi: 10.3389/fphar.2025.1568780 (PMC12171135; doi:10.3389/fphar.2025.1568780)
Supplement: Supplementary file 3 [file Supplementaryfile3.docx]

| Table. | | | | | |
| --- | --- | --- | --- | --- | --- |
| Gene | b_SMR | se_SMR | p_SMR | p_HEIDI | nsnp_HEIDI |
| ISG15 | -0.11505 | 0.0929115 | 0.2156125 | 0.7048289 | 20 |
| AGRN | 0.0358772 | 0.120843 | 0.7665492 | 0.1196663 | 20 |
| B3GALT6 | 0.523858 | 0.565305 | 0.3540918 | 0.9354917 | 20 |
| MXRA8 | 0.121273 | 0.347235 | 0.7269001 | 0.5062983 | 20 |
| VWA1 | -0.121813 | 0.126361 | 0.335041 | 0.3174528 | 20 |
| NADK | 0.0293602 | 0.236051 | 0.9010138 | 0.44112 | 20 |
| KCNAB2 | -0.49272 | 0.440682 | 0.2635304 | 0.1779692 | 20 |
| PARK7 | -0.109167 | 0.26191 | 0.6768171 | 0.2345143 | 20 |
| CA6 | -0.117744 | 0.0544085 | 0.03045888 | 0.4065995 | 20 |
| H6PD | -0.039958 | 0.0651579 | 0.5397117 | 0.6562864 | 20 |
| CLSTN1 | -0.0673693 | 0.222348 | 0.7618973 | 0.9763324 | 17 |
| LZIC | -0.0689834 | 0.499916 | 0.8902484 | 0.4233137 | 8 |
| RBP7 | -0.24491 | 0.249934 | 0.3271369 | 0.28862 | 20 |
| PGD | -0.311247 | 0.307272 | 0.3110912 | 0.6830728 | 20 |
| PEX14 | 0.0786642 | 0.560527 | 0.8883915 | 0.1178734 | 20 |
| NPPA | -0.160168 | 0.379001 | 0.6725834 | 0.2303157 | 14 |
| NPPB | -0.124908 | 0.327179 | 0.70263 | 0.7645973 | 20 |
| NPPB | -0.0435637 | 0.114052 | 0.7024879 | 0.7909561 | 20 |
| TNFRSF1B | -0.131071 | 0.397637 | 0.7416831 | 0.3955408 | 20 |
| TNFRSF1B | -0.086301 | 0.261769 | 0.7416389 | 0.6803939 | 20 |
| CELA2A | 0.0254242 | 0.40695 | 0.9501846 | 0.07561492 | 20 |
| EPHA2 | -0.103375 | 0.224164 | 0.6446848 | 0.9884612 | 20 |
| MFAP2 | -0.00625297 | 0.10117 | 0.9507167 | 0.107539 | 20 |
| MFAP2 | -0.00746025 | 0.120703 | 0.9507168 | 0.1456271 | 20 |
| AKR7A3 | -0.186849 | 0.150167 | 0.2134002 | 0.04077389 | 20 |
| AKR7A2 | -0.286935 | 0.208232 | 0.1682169 | 0.04936495 | 20 |
| NBL1 | -0.0290563 | 0.542983 | 0.9573236 | 0.8059031 | 20 |
| PLA2G2A | -0.05209 | 0.0418846 | 0.2136274 | 0.764423 | 20 |
| DDOST | -0.143125 | 0.434311 | 0.741743 | 0.7583086 | 20 |
| HSPG2 | -0.0320812 | 0.128509 | 0.8028648 | 0.9700868 | 20 |
| C1QC | 0.00783359 | 0.0615317 | 0.8986951 | 0.4582201 | 20 |
| EPHB2 | 0.083568 | 0.0900607 | 0.3534556 | 0.8804785 | 19 |
| IFNLR1 | 0.0595638 | 0.171689 | 0.7286452 | 0.8602487 | 10 |
| LDLRAP1 | -0.0760771 | 0.309392 | 0.8057655 | 0.8931239 | 13 |
| MAN1C1 | 0.11194 | 0.151336 | 0.4594956 | 0.810546 | 20 |
| SH3BGRL3 | 0.04034 | 0.111123 | 0.716588 | 0.2789227 | 20 |
| RPS6KA1 | -0.672086 | 0.304294 | 0.02719723 | 0.3567195 | 20 |
| FCN3 | -0.0642524 | 0.0711753 | 0.3666671 | 0.1505878 | 20 |
| FGR | -0.13198 | 0.223832 | 0.555432 | 0.1706758 | 20 |
| STX12 | -0.0419695 | 1.01075 | 0.9668788 | 0.9774344 | 12 |
| EPB41 | -0.210078 | 0.420192 | 0.6171057 | 0.3044427 | 16 |
| PTPRU | -0.0772747 | 0.217351 | 0.7221923 | 0.2341001 | 18 |
| TINAGL1 | -0.791645 | 0.312328 | 0.01125561 | 0.03371899 | 20 |
| KPNA6 | -0.443884 | 0.450082 | 0.3240206 | 0.07347503 | 20 |
| ADPRHL2 | 0.0249225 | 0.145319 | 0.8638286 | 0.07429535 | 3 |
| RSPO1 | 0.290559 | 0.199845 | 0.1459683 | 0.1674 | 20 |
| EPHA10 | -0.296882 | 0.422923 | 0.4826937 | 0.7665291 | 20 |
| INPP5B | 0.144802 | 0.179423 | 0.4196423 | 0.1374637 | 20 |
| PPIE | -0.0872845 | 0.102798 | 0.395831 | 0.1708296 | 20 |
| PPT1 | -0.0962637 | 0.207034 | 0.6419555 | 0.7061643 | 20 |
| GUCA2B | -0.196513 | 0.237881 | 0.408748 | 0.3152462 | 20 |
| PPCS | -0.198502 | 0.307939 | 0.5191767 | 0.6966898 | 20 |
| PPIH | -0.514751 | 0.577271 | 0.3725554 | 0.7501568 | 4 |
| TIE1 | -0.0725978 | 0.154423 | 0.6382668 | 0.1020397 | 20 |
| B4GALT2 | 0.202888 | 0.158582 | 0.2007607 | 0.4049336 | 20 |
| UROD | -0.0261282 | 0.121173 | 0.8292778 | 0.2728907 | 20 |
| PRDX1 | 0.160932 | 0.196039 | 0.4116937 | 0.147496 | 7 |
| AKR1A1 | -0.0689035 | 0.0491674 | 0.1610931 | 0.3701712 | 20 |
| CMPK1 | -0.705977 | 0.363913 | 0.05238419 | 0.1795705 | 19 |
| TXNDC12 | -0.523799 | 0.334583 | 0.1174586 | 0.5179217 | 20 |
| GPX7 | -0.0443277 | 0.0562065 | 0.4303128 | 0.1199776 | 20 |
| LRP8 | -0.292291 | 0.164062 | 0.07481726 | 0.8435014 | 20 |
| FAM151A | -0.0808576 | 0.120839 | 0.5034098 | 0.5678107 | 20 |
| PCSK9 | 0.115985 | 0.0862075 | 0.1784907 | 0.2688044 | 11 |
| ANGPTL3 | -0.249404 | 0.147864 | 0.09165975 | 0.3480872 | 20 |
| PGM1 | 0.0579389 | 0.26732 | 0.8284115 | 0.3407117 | 20 |
| ROR1 | -0.0710629 | 0.10406 | 0.4946691 | 0.3879164 | 20 |
| LEPR | -0.0130236 | 0.031533 | 0.6795957 | 0.7359539 | 20 |
| INSL5 | 0.00569314 | 0.514067 | 0.9911638 | 0.5943446 | 20 |
| NEGR1 | 0.254547 | 0.216977 | 0.2407358 | 0.9915987 | 20 |
| NEGR1 | 0.276736 | 0.235986 | 0.2409236 | 0.9591149 | 20 |
| CRYZ | -0.0515953 | 0.0399122 | 0.1961079 | 0.9113804 | 20 |
| ACADM | -0.0139435 | 0.144317 | 0.9230305 | 0.6688175 | 20 |
| DNAJB4 | 0.305978 | 0.198931 | 0.1240206 | 0.459187 | 20 |
| BCL10 | -0.833607 | 0.556028 | 0.1338169 | 0.9727058 | 20 |
| CCBL2 | 0.105202 | 0.100055 | 0.2930574 | 0.5170107 | 20 |
| GBP1 | 0.00746552 | 0.099351 | 0.940101 | 0.3855017 | 6 |
| GBP2 | -0.169124 | 0.361837 | 0.6402111 | 0.97247 | 20 |
| TGFBR3 | 0.520375 | 0.307955 | 0.09107038 | 0.3880046 | 20 |
| GCLM | 0.755777 | 0.340504 | 0.02644708 | 0.9016757 | 20 |
| AMY2B | -0.00363035 | 0.102318 | 0.9716961 | 0.6556085 | 20 |
| AMY2A | -0.0634849 | 0.0635118 | 0.3175156 | 0.9761327 | 20 |
| AMY1A | 0.00232951 | 0.0627876 | 0.9704041 | 0.005447377 | 20 |
| NTNG1 | 0.0453847 | 0.0455238 | 0.3187913 | 0.505966 | 20 |
| GSTM4 | 0.0356426 | 0.0611581 | 0.5600316 | 0.8944014 | 20 |
| GSTM1 | -0.0508318 | 0.076272 | 0.505121 | 0.9614056 | 20 |
| GSTM3 | 0.0316724 | 0.050229 | 0.5283277 | 0.9905269 | 20 |
| CHI3L2 | 0.897589 | 0.544171 | 0.0990535 | 0.7868123 | 15 |
| RHOC | -0.568781 | 0.290503 | 0.05023966 | 0.6167757 | 20 |
| OLFML3 | -0.464418 | 0.343498 | 0.1763679 | 0.3000047 | 20 |
| CD58 | -0.0337782 | 0.120997 | 0.7801167 | 0.9991601 | 11 |
| IGSF3 | 0.0905555 | 0.259846 | 0.7274675 | 0.9918761 | 20 |
| PTGFRN | 0.000169678 | 0.0388927 | 0.9965191 | 0.3734444 | 20 |
| MAN1A2 | 0.0263159 | 0.153205 | 0.8636193 | 0.9380273 | 20 |
| HMGCS2 | 0.106577 | 0.525135 | 0.8391733 | 0.1822864 | 11 |
| REG4 | -0.105985 | 0.256546 | 0.6795181 | 0.4266115 | 20 |
| ACP6 | -0.0127719 | 0.034296 | 0.7095929 | 0.8040441 | 20 |
| BOLA1 | -0.0707577 | 0.06392 | 0.2683054 | 0.5902152 | 20 |
| ECM1 | 0.0709712 | 0.0430302 | 0.09907915 | 0.4163599 | 20 |
| MCL1 | -0.180189 | 0.314224 | 0.5663458 | 0.6271807 | 12 |
| CTSS | 0.083799 | 0.0818552 | 0.3059545 | 0.9102837 | 20 |
| MENT | -0.0683982 | 0.363547 | 0.8507661 | 0.6995451 | 6 |
| PSMB4 | -0.110564 | 0.0628061 | 0.0783395 | 0.03522432 | 20 |
| CRNN | 0.0231391 | 0.259001 | 0.9288117 | 0.886387 | 20 |
| S100A9 | 0.533961 | 0.617811 | 0.3874348 | 0.6468716 | 7 |
| S100A12 | 0.132749 | 0.151945 | 0.3823034 | 0.5066116 | 11 |
| S100A7 | -0.0574014 | 0.0410857 | 0.1623794 | 0.7910901 | 20 |
| S100A6 | -0.0415426 | 0.636903 | 0.947994 | 0.08628657 | 5 |
| S100A4 | -0.061978 | 0.333078 | 0.8523846 | 0.7126125 | 20 |
| S100A2 | 0.195406 | 0.451647 | 0.6652682 | 0.1014464 | 20 |
| S100A16 | -0.415037 | 0.155538 | 0.007621384 | 0.1966292 | 20 |
| S100A14 | 0.875892 | 0.334608 | 0.008853368 | 0.1710572 | 20 |
| CREB3L4 | 0.22021 | 0.143758 | 0.1255704 | 0.8811431 | 20 |
| IL6R | 0.0314171 | 0.0307324 | 0.3066483 | 0.9794867 | 20 |
| PMVK | 0.235902 | 0.342818 | 0.491373 | 0.532261 | 3 |
| EFNA3 | -0.193881 | 1.1023 | 0.8603824 | 0.6739185 | 20 |
| THBS3 | 0.574444 | 0.765425 | 0.452959 | 0.653213 | 20 |
| PKLR | 0.00883824 | 0.218179 | 0.9676873 | 0.5254334 | 20 |
| SYT11 | 0.0483174 | 0.410603 | 0.9063258 | 0.5185686 | 20 |
| SEMA4A | -0.094003 | 0.174371 | 0.5898203 | 0.9043494 | 20 |
| APOA1BP | -0.0740101 | 0.183381 | 0.6865176 | 0.5249475 | 17 |
| BCAN | -0.0301381 | 0.156052 | 0.8468585 | 0.4431273 | 13 |
| CRABP2 | -0.0568012 | 0.102889 | 0.580905 | 0.1635915 | 20 |
| HDGF | 0.475661 | 0.400342 | 0.2347793 | 0.1026811 | 20 |
| HDGF | 0.480755 | 0.404699 | 0.2348603 | 0.102327 | 20 |
| PEAR1 | -0.148369 | 0.154261 | 0.3361486 | 0.7180855 | 20 |
| PEAR1 | -1.54997 | 0.636433 | 0.01487517 | 0.5811648 | 14 |
| FCRL4 | -0.00184201 | 0.0290873 | 0.9495061 | 0.4183111 | 20 |
| FCRL3 | -0.0343077 | 0.051664 | 0.5066562 | 0.5195145 | 20 |
| FCRL1 | 0.0436128 | 0.0758557 | 0.5653288 | 0.2669123 | 20 |
| CD5L | -0.0243095 | 0.0948635 | 0.7977523 | 0.5865747 | 20 |
| IFI16 | 0.365372 | 0.569897 | 0.5214448 | 0.4764256 | 20 |
| IFI16 | -0.349711 | 0.394907 | 0.3758583 | 0.05792301 | 20 |
| CADM3 | 0.669117 | 0.665565 | 0.3147353 | 0.6746541 | 5 |
| APCS | -0.268433 | 0.148293 | 0.07027277 | 0.7173464 | 14 |
| CRP | 0.0457415 | 0.17998 | 0.7993809 | 0.7016757 | 20 |
| FCRL6 | 0.138059 | 0.26261 | 0.5990841 | 0.3957813 | 20 |
| TAGLN2 | 0.781513 | 0.312756 | 0.01246175 | 0.5854588 | 18 |
| TAGLN2 | 0.789093 | 0.315787 | 0.01246096 | 0.679252 | 17 |
| IGSF8 | -0.167957 | 0.199163 | 0.3990527 | 0.3235524 | 20 |
| SLAMF6 | -0.22787 | 0.169244 | 0.1781732 | 0.2443956 | 15 |
| CD48 | 0.0117963 | 0.131398 | 0.9284654 | 0.8074963 | 20 |
| SLAMF7 | 0.0141384 | 0.0359487 | 0.6941025 | 0.2041165 | 20 |
| LY9 | -0.0260936 | 0.0653938 | 0.6898767 | 0.811442 | 20 |
| CD244 | 1.32002 | 0.874291 | 0.1310899 | 0.09258149 | 5 |
| ITLN1 | 1.07432 | 0.794179 | 0.1761398 | 0.3563543 | 9 |
| TSTD1 | 0.170833 | 0.0839916 | 0.04195823 | 0.356227 | 20 |
| PVRL4 | 0.00619736 | 0.538191 | 0.9908124 | 0.9170485 | 20 |
| FCGR2A | -0.0246134 | 0.0230219 | 0.2850107 | 0.3754628 | 20 |
| FCGR3A | -0.0155 | 0.0251806 | 0.5381888 | 0.4488181 | 20 |
| FCGR3B | -0.0371207 | 0.0777392 | 0.633004 | 0.2816706 | 20 |
| FCGR2B | -0.0205557 | 0.029077 | 0.4796037 | 0.5912348 | 20 |
| FCRLB | -0.0552688 | 0.104526 | 0.5969752 | 0.5526654 | 20 |
| ATF6 | -0.540304 | 0.585468 | 0.3560809 | 0.2626857 | 19 |
| UAP1 | 0.454143 | 0.535056 | 0.3960051 | 0.523726 | 18 |
| CREG1 | -0.165841 | 0.219601 | 0.4501338 | 0.1025542 | 20 |
| XCL2 | 0.188192 | 0.450372 | 0.67605 | 0.176687 | 12 |
| DPT | 0.0226326 | 0.0837753 | 0.7870384 | 0.4412505 | 20 |
| F5 | 0.172419 | 0.110584 | 0.1189569 | 0.005890894 | 11 |
| SELP | 0.22665 | 0.110988 | 0.04113952 | 0.3318737 | 20 |
| SELL | -0.0628371 | 0.0856584 | 0.463206 | 0.9031761 | 20 |
| SELE | -0.0910225 | 0.262472 | 0.72875 | 0.4750131 | 20 |
| MYOC | 0.0144484 | 0.0961624 | 0.8805672 | 0.1408115 | 20 |
| PRDX6 | 0.578531 | 0.337136 | 0.08615944 | 0.9106033 | 20 |
| DARS2 | -0.225221 | 0.353034 | 0.5235 | 0.2147853 | 20 |
| SERPINC1 | -0.353435 | 0.557996 | 0.5264717 | 0.261909 | 11 |
| CACYBP | -0.303456 | 0.233568 | 0.1938682 | 0.3836253 | 20 |
| TNR | 0.108941 | 0.21755 | 0.6165369 | 0.6961079 | 20 |
| ANGPTL1 | -0.0633328 | 0.0948296 | 0.5042236 | 0.4297937 | 20 |
| ANGPTL1 | -0.00378253 | 0.395969 | 0.9923783 | 0.6137289 | 20 |
| FAM20B | 0.136668 | 0.596609 | 0.8188106 | 0.9995723 | 4 |
| GLUL | 0.0499612 | 0.563581 | 0.9293604 | 0.2342975 | 19 |
| NPL | -0.250747 | 0.151295 | 0.09745187 | 0.5344695 | 20 |
| LAMC2 | 0.0533685 | 0.048226 | 0.2684527 | 0.5522757 | 20 |
| RGS18 | -0.325389 | 0.523117 | 0.5339295 | 0.3370517 | 14 |
| GLRX2 | -0.12942 | 0.212667 | 0.5428171 | 0.04649869 | 13 |
| CFH | -0.0197621 | 0.147283 | 0.8932623 | 0.2650919 | 20 |
| CFHR3 | 0.096363 | 0.265989 | 0.7171415 | 0.8862297 | 20 |
| CFHR1 | -0.00934541 | 0.0500808 | 0.8519691 | 0.979657 | 20 |
| CFHR4 | -0.0291694 | 0.0420181 | 0.4875511 | 0.1112068 | 20 |
| CFHR2 | 0.028061 | 0.0332386 | 0.3985412 | 0.9957225 | 20 |
| CFHR5 | 0.0128761 | 0.0919188 | 0.8885961 | 0.128326 | 20 |
| F13B | -0.168189 | 0.0851687 | 0.04829383 | 0.1473466 | 20 |
| NEK7 | -0.132761 | 0.421207 | 0.7526162 | 0.4419361 | 16 |
| TMEM9 | -1.61398 | 0.484527 | 0.000865216 | 0.007553002 | 20 |
| CSRP1 | 0.105325 | 0.298848 | 0.724512 | 0.9292448 | 20 |
| RNPEP | -0.103518 | 0.108679 | 0.3408392 | 0.7804717 | 20 |
| PTPN7 | -0.394632 | 0.523117 | 0.4506177 | 0.6386644 | 11 |
| CHI3L1 | -0.0151652 | 0.0284169 | 0.5935713 | 0.6343951 | 20 |
| CHIT1 | 0.027214 | 0.0344214 | 0.4291714 | 0.6346345 | 20 |
| FMOD | -0.313849 | 0.198505 | 0.113864 | 0.4667482 | 20 |
| REN | 0.0439841 | 0.133395 | 0.7416052 | 0.8340115 | 12 |
| NFASC | -0.148497 | 0.101672 | 0.1441376 | 0.4500881 | 20 |
| CNTN2 | -0.0742743 | 0.0464519 | 0.1098316 | 0.594843 | 20 |
| CTSE | -0.309037 | 0.378032 | 0.4136496 | 0.6678783 | 12 |
| MAPKAPK2 | 0.0944161 | 0.174175 | 0.5877663 | 0.4174788 | 20 |
| FAIM3 | -0.0371602 | 0.270528 | 0.8907448 | 0.3356616 | 20 |
| PIGR | 0.365175 | 0.205226 | 0.07517706 | 0.8632906 | 20 |
| C4BPA | 0.0944412 | 0.0963676 | 0.3270813 | 0.8610605 | 17 |
| CD55 | 0.0216447 | 0.0786791 | 0.7832389 | 0.7851722 | 20 |
| CR2 | -0.0622347 | 0.221512 | 0.7787458 | 0.2299184 | 20 |
| CR1 | 0.0245428 | 0.0708557 | 0.7290589 | 0.4475416 | 20 |
| CD46 | 0.114964 | 0.120139 | 0.3386062 | 0.7290062 | 20 |
| PPP2R5A | 0.169277 | 0.446465 | 0.704576 | 0.2936702 | 20 |
| LYPLAL1 | 0.19724 | 0.312682 | 0.5281722 | 0.4373606 | 20 |
| BPNT1 | 0.0777226 | 0.511129 | 0.879139 | 0.3381897 | 20 |
| CAPN2 | 0.045506 | 0.142686 | 0.7497844 | 0.7495951 | 20 |
| LEFTY2 | -0.0702587 | 0.162142 | 0.6647859 | 0.8593938 | 20 |
| LEFTY2 | -0.0167678 | 0.0412091 | 0.6840849 | 0.8020957 | 20 |
| PARP1 | 0.259654 | 0.535418 | 0.6277074 | 0.6390159 | 20 |
| PARP1 | 0.582332 | 0.584138 | 0.3188091 | 0.5794795 | 16 |
| GUK1 | 0.0162128 | 0.163467 | 0.9209949 | 0.920416 | 20 |
| AGT | 0.191849 | 0.163768 | 0.2414114 | 0.7837763 | 20 |
| C1orf198 | -0.392736 | 0.283917 | 0.1665787 | 0.2273477 | 20 |
| EGLN1 | 0.0284405 | 0.53061 | 0.9572541 | 0.7219203 | 20 |
| GGPS1 | -0.270585 | 0.363234 | 0.4563119 | 0.6703028 | 20 |
| NID1 | 0.0181282 | 0.172113 | 0.916116 | 0.9454061 | 4 |
| ERO1LB | -0.189889 | 0.171626 | 0.26855 | 0.9906916 | 20 |
| GREM2 | 0.758635 | 0.754822 | 0.3148718 | 0.8399097 | 6 |
| FH | 0.269852 | 0.226331 | 0.2331481 | 0.6480979 | 20 |
| ACP1 | -0.0105335 | 0.0272388 | 0.69897 | 0.0268717 | 20 |
| PXDN | 0.0434849 | 0.208147 | 0.8345152 | 0.2772461 | 20 |
| ADI1 | 0.172718 | 0.310171 | 0.5776315 | 0.8146297 | 20 |
| COLEC11 | -0.0448511 | 0.035238 | 0.2030873 | 0.1390428 | 20 |
| HPCAL1 | 0.130349 | 0.246951 | 0.5976158 | 0.1698067 | 14 |
| MATN3 | 0.0597057 | 0.0536331 | 0.2656125 | 0.5882498 | 20 |
| APOB | -1.1955 | 0.439885 | 0.006572494 | 0.1631131 | 20 |
| FKBP1B | -0.0561442 | 0.155279 | 0.7176744 | 0.2856482 | 20 |
| TP53I3 | 0.108235 | 0.0672321 | 0.1074247 | 0.8404084 | 20 |
| DNAJC27 | -0.509218 | 0.695247 | 0.4639081 | 0.4030093 | 20 |
| POMC | -0.564786 | 0.474202 | 0.2336444 | 0.5551295 | 3 |
| NRBP1 | 0.717181 | 0.607801 | 0.2380155 | 0.2604067 | 15 |
| GCKR | -0.62963 | 0.350869 | 0.07273488 | 0.6942782 | 20 |
| GPN1 | 0.484906 | 0.408463 | 0.2351689 | 0.5845978 | 20 |
| DPY30 | 0.0125302 | 0.500456 | 0.980025 | 0.6126723 | 20 |
| VIT | -0.0211924 | 0.0804869 | 0.7923175 | 0.5013492 | 20 |
| QPCT | 0.0873926 | 0.151422 | 0.5638407 | 0.05500325 | 20 |
| PKDCC | 0.42768 | 0.468763 | 0.3615803 | 0.9202022 | 13 |
| ERLEC1 | -0.107104 | 0.361875 | 0.7672541 | 0.5244755 | 20 |
| ACYP2 | 0.0271581 | 0.131865 | 0.8368272 | 0.4640665 | 20 |
| EFEMP1 | 0.310078 | 0.319139 | 0.3312456 | 0.02341342 | 20 |
| B3GNT2 | 0.251947 | 0.181084 | 0.1641264 | 0.02433333 | 20 |
| EHBP1 | 0.809922 | 0.713422 | 0.2562646 | 0.03158396 | 16 |
| MDH1 | 1.16044 | 0.463572 | 0.01230593 | 0.06551705 | 20 |
| HSPC159 | 0.301071 | 0.554579 | 0.5872104 | 0.3469035 | 14 |
| RAB1A | 0.252458 | 0.520463 | 0.6276305 | 0.9644711 | 16 |
| CNRIP1 | 0.0420267 | 0.0595653 | 0.4804643 | 0.6104839 | 20 |
| PLEK | -0.0182406 | 0.248061 | 0.9413822 | 0.6442442 | 20 |
| ARHGAP25 | 0.0272969 | 0.189787 | 0.8856357 | 0.736256 | 20 |
| GKN2 | -0.0917006 | 0.0802914 | 0.2534136 | 0.8953571 | 20 |
| ANTXR1 | -1.25447 | 0.658904 | 0.05692738 | 0.9573261 | 20 |
| ANXA4 | -0.00969996 | 0.112486 | 0.9312816 | 0.7910484 | 20 |
| PCYOX1 | -0.160894 | 0.0674532 | 0.0170666 | 0.07698588 | 20 |
| NAGK | 0.0284176 | 0.174488 | 0.8706262 | 0.4307716 | 20 |
| MCEE | -1.00329 | 0.582844 | 0.08518275 | 0.681988 | 20 |
| BOLA3 | -0.299052 | 0.252471 | 0.2362154 | 0.3566404 | 20 |
| MTHFD2 | -0.0593232 | 0.254368 | 0.8155921 | 0.1092376 | 7 |
| LOXL3 | -0.66519 | 0.321183 | 0.03835318 | 0.5310857 | 20 |
| HK2 | -0.451161 | 0.572107 | 0.4303479 | 0.9864897 | 9 |
| LRRTM4 | -0.00375773 | 0.556176 | 0.9946092 | 0.8480165 | 20 |
| REG3G | 0.014449 | 0.0583537 | 0.8044355 | 0.575846 | 20 |
| REG1B | -0.0933211 | 0.178506 | 0.601121 | 0.7186645 | 20 |
| REG1A | -0.093693 | 0.179218 | 0.6011215 | 0.7256239 | 20 |
| REG3A | 0.00884247 | 0.194933 | 0.9638192 | 0.7790259 | 20 |
| CAPG | -0.148012 | 0.096647 | 0.1256539 | 0.5012463 | 11 |
| SFTPB | -0.117197 | 0.0735139 | 0.110887 | 0.8587947 | 8 |
| GNLY | 0.0173675 | 0.0394428 | 0.659704 | 0.656081 | 20 |
| VPS24 | 0.311247 | 0.550025 | 0.5714766 | 0.8274052 | 20 |
| CD8A | -0.127193 | 0.119628 | 0.2876736 | 0.7841503 | 20 |
| FABP1 | -0.561319 | 0.264024 | 0.03350222 | 0.9045251 | 20 |
| RPIA | 0.344043 | 0.246573 | 0.1629254 | 0.9331143 | 20 |
| LMAN2L | 0.0258155 | 0.263545 | 0.9219683 | 0.7021876 | 20 |
| SEMA4C | 0.0725699 | 0.142446 | 0.6104318 | 0.06829493 | 16 |
| IL1R2 | -0.100626 | 0.0692279 | 0.146072 | 0.4311863 | 20 |
| IL1R1 | -0.0895981 | 0.171446 | 0.6012499 | 0.803287 | 20 |
| IL1RL2 | -0.0328668 | 0.182464 | 0.8570526 | 0.3079672 | 20 |
| IL1RL1 | 0.00773884 | 0.0409616 | 0.8501483 | 0.7767993 | 20 |
| IL18R1 | 0.0229349 | 0.0511176 | 0.6536707 | 0.4582579 | 20 |
| IL18RAP | 0.056081 | 0.36938 | 0.8793253 | 0.4480895 | 20 |
| UXS1 | -0.179899 | 0.221449 | 0.4165777 | 0.6783155 | 20 |
| EDAR | -0.0997828 | 0.101467 | 0.3254105 | 0.9820806 | 15 |
| TTL | -0.27324 | 0.735673 | 0.7103279 | 0.9990464 | 20 |
| IL1RN | 0.0652791 | 0.171207 | 0.7029895 | 0.5717558 | 20 |
| DBI | -0.0427374 | 0.13437 | 0.750442 | 0.6042383 | 20 |
| RALB | -0.114015 | 0.121124 | 0.3465481 | 0.8158009 | 20 |
| INHBB | -0.102131 | 0.0839594 | 0.2238203 | 0.8882191 | 20 |
| BIN1 | -0.275263 | 0.227197 | 0.2256805 | 0.5649956 | 20 |
| PROC | -0.0716069 | 0.207754 | 0.7303418 | 0.7344226 | 20 |
| HS6ST1 | 0.311376 | 0.194522 | 0.109437 | 0.3248891 | 20 |
| LCT | -0.024574 | 0.0361171 | 0.4962528 | 0.9956285 | 20 |
| DARS | 0.0878743 | 0.347977 | 0.8006322 | 0.7801384 | 20 |
| KYNU | 0.0117211 | 0.0850576 | 0.8903966 | 0.379254 | 20 |
| TNFAIP6 | 0.0713979 | 0.056823 | 0.208936 | 0.1633735 | 20 |
| GALNT13 | -0.6765 | 0.633992 | 0.2859502 | 0.2484503 | 20 |
| LY75 | 0.0294458 | 0.0338376 | 0.3841855 | 0.003422528 | 20 |
| PLA2R1 | 0.0566702 | 0.0372113 | 0.1277768 | 0.2453925 | 20 |
| FAP | -0.0217671 | 0.150838 | 0.8852577 | 0.9279643 | 13 |
| GCA | 0.146976 | 0.100881 | 0.1451363 | 0.7215688 | 20 |
| PDK1 | -0.0696333 | 0.149532 | 0.6414471 | 0.5415123 | 20 |
| OLA1 | -0.0596532 | 0.236796 | 0.8011044 | 0.3047052 | 20 |
| FKBP7 | -0.176434 | 0.114251 | 0.1225236 | 0.4315537 | 20 |
| DNAJC10 | 0.27736 | 0.541077 | 0.6082265 | 0.8499198 | 6 |
| FRZB | -0.0263877 | 0.112786 | 0.8150137 | 0.648621 | 20 |
| FAM171B | -0.31524 | 0.205136 | 0.1243575 | 0.2236888 | 20 |
| TFPI | -0.292507 | 0.783022 | 0.7087306 | 0.4732309 | 3 |
| COL3A1 | 0.23718 | 0.447548 | 0.5961437 | 0.3751951 | 20 |
| HIBCH | -0.182517 | 0.0893738 | 0.04113417 | 0.0885051 | 20 |
| NRP2 | 0.3243 | 0.210581 | 0.1235541 | 0.7105078 | 20 |
| ADAM23 | -0.0818256 | 0.0869346 | 0.346586 | 0.2515546 | 20 |
| CRYGD | 0.0756873 | 0.261426 | 0.7721854 | 0.3350502 | 20 |
| IDH1 | 0.177295 | 0.226578 | 0.4339253 | 0.319275 | 20 |
| RPE | 0.101518 | 0.377816 | 0.7881634 | 0.4582287 | 20 |
| LANCL1 | -0.394467 | 0.239861 | 0.1000595 | 0.9692938 | 20 |
| FN1 | -0.176803 | 0.219927 | 0.4214457 | 0.003962732 | 20 |
| FN1 | -0.18123 | 0.222912 | 0.4162116 | 0.001670806 | 20 |
| FN1 | -0.159826 | 0.198776 | 0.421368 | 0.002745176 | 20 |
| PECR | 0.0818368 | 0.370985 | 0.8254094 | 0.6857194 | 20 |
| IGFBP2 | 1.33094 | 0.587666 | 0.02352585 | 0.1957937 | 6 |
| IGFBP5 | -0.188205 | 0.569238 | 0.7409275 | 0.8744935 | 20 |
| NHEJ1 | -0.951543 | 0.485489 | 0.04999942 | 0.6077665 | 10 |
| EPHA4 | -0.118836 | 0.24445 | 0.6268693 | 0.8769534 | 20 |
| SERPINE2 | -0.0241224 | 0.0443678 | 0.5866534 | 0.4156234 | 20 |
| AGFG1 | 0.247449 | 0.318425 | 0.4370982 | 0.2059053 | 20 |
| DNER | 0.0790368 | 0.171521 | 0.6449416 | 0.8255304 | 20 |
| ALPPL2 | 0.0769939 | 0.554605 | 0.8895873 | 0.80949 | 20 |
| ALPPL2 | -0.0105864 | 0.16156 | 0.9477551 | 0.8096943 | 20 |
| UGT1A6 | 0.0800151 | 0.125598 | 0.5240764 | 0.2959416 | 20 |
| UGT1A1 | 0.0790921 | 0.130976 | 0.5459323 | 0.3476095 | 20 |
| COL6A3 | 0.0942506 | 0.265655 | 0.7227501 | 0.9235084 | 20 |
| COL6A3 | 0.080175 | 0.431802 | 0.8526993 | 0.6789448 | 15 |
| UBE2F | 0.164313 | 0.433001 | 0.7043362 | 0.1408823 | 20 |
| GPC1 | -0.133488 | 0.0684096 | 0.05102142 | 0.944307 | 20 |
| DUSP28 | 0.116409 | 0.130064 | 0.3707814 | 0.02463864 | 20 |
| DTYMK | 0.117534 | 0.629524 | 0.8518936 | 0.8201738 | 20 |
| CHL1 | 0.198835 | 0.129392 | 0.1243694 | 0.3723568 | 20 |
| CNTN4 | -0.211785 | 0.112647 | 0.06009808 | 0.5225812 | 20 |
| IL5RA | 0.124092 | 0.100112 | 0.2151486 | 0.103991 | 20 |
| CRELD1 | 0.026185 | 0.0436166 | 0.5482758 | 0.8391809 | 20 |
| TIMP4 | -0.00471842 | 0.0925493 | 0.9593392 | 0.06890395 | 20 |
| BTD | -0.0795395 | 0.0481101 | 0.09827353 | 0.09371634 | 20 |
| GLB1 | -0.236253 | 0.654175 | 0.7179902 | 0.2723455 | 20 |
| PDCD6IP | -0.319728 | 0.29921 | 0.2852619 | 0.8588282 | 20 |
| ACAA1 | 0.00364917 | 0.143295 | 0.9796832 | 0.3611928 | 20 |
| POMGNT2 | -0.0274311 | 0.0879654 | 0.7551625 | 0.0623126 | 20 |
| TGM4 | -0.561153 | 0.567218 | 0.3225132 | 0.3352409 | 20 |
| CLEC3B | 0.0938127 | 0.174193 | 0.5901942 | 0.236192 | 20 |
| CDCP1 | 0.150081 | 0.122952 | 0.2222173 | 0.4485996 | 20 |
| LTF | 0.0920897 | 0.190318 | 0.628476 | 0.04129131 | 11 |
| TDGF1 | 0.0472956 | 0.0234272 | 0.04350434 | 0.8275745 | 20 |
| CAMP | -0.51425 | 0.323951 | 0.1124154 | 0.5699788 | 20 |
| CAMP | 0.326807 | 0.390348 | 0.40247 | 0.5336325 | 9 |
| MST1 | -0.015122 | 0.023644 | 0.5224511 | 0.4610776 | 20 |
| HYAL1 | -0.142909 | 0.245466 | 0.5604362 | 0.7715305 | 20 |
| MANF | 0.427965 | 0.490889 | 0.383308 | 0.8112025 | 20 |
| ACY1 | -0.0706113 | 0.219224 | 0.7473801 | 0.857659 | 7 |
| SEMA3G | -0.171085 | 0.146741 | 0.2436556 | 0.07581418 | 20 |
| ITIH1 | -0.0205501 | 0.0573071 | 0.7198976 | 0.9152299 | 20 |
| ITIH3 | 0.0506185 | 0.0593593 | 0.3937997 | 0.9942476 | 20 |
| ITIH4 | 0.389066 | 0.340239 | 0.2528268 | 0.6946679 | 20 |
| CACNA2D3 | 0.150919 | 0.13152 | 0.2511763 | 0.6347324 | 20 |
| IL17RD | -0.0470877 | 0.0737824 | 0.5233451 | 0.7279402 | 20 |
| ARF4 | -0.267045 | 0.371475 | 0.4722168 | 0.2782003 | 20 |
| SLMAP | -0.478527 | 0.669112 | 0.474506 | 0.2787034 | 12 |
| FAM3D | 0.151519 | 0.094645 | 0.1093949 | 0.9846056 | 20 |
| FHIT | 0.249229 | 0.402422 | 0.535703 | 0.9671748 | 20 |
| LRIG1 | 0.0357066 | 0.0490781 | 0.4668913 | 0.1723758 | 20 |
| PROK2 | 0.0305954 | 0.0525257 | 0.5602402 | 0.2845992 | 20 |
| ROBO2 | -1.30671 | 0.743327 | 0.0787608 | 0.6528994 | 20 |
| ROBO1 | 0.0154673 | 0.147987 | 0.9167584 | 0.517377 | 20 |
| CHMP2B | -0.072005 | 0.118755 | 0.5442927 | 0.2272793 | 20 |
| PROS1 | -0.754152 | 0.635279 | 0.2351803 | 0.0184633 | 20 |
| CPOX | -0.130038 | 0.178853 | 0.4671873 | 0.6088054 | 20 |
| ST3GAL6 | -0.00538829 | 0.0336715 | 0.8728612 | 0.7476954 | 20 |
| NIT2 | 0.146658 | 0.310267 | 0.6364389 | 0.5002516 | 20 |
| PCNP | 0.156955 | 0.349849 | 0.6536951 | 0.8922302 | 20 |
| ALCAM | 0.0131391 | 0.33249 | 0.9684778 | 0.6079501 | 17 |
| ABHD10 | -0.5821 | 0.60453 | 0.3355998 | 0.5448717 | 20 |
| CD200 | 0.0619971 | 0.31952 | 0.846151 | 0.3211247 | 20 |
| CCDC80 | -0.0785481 | 0.325637 | 0.8093893 | 0.758192 | 20 |
| CD200R1 | -0.0858912 | 0.265712 | 0.7465066 | 0.904531 | 20 |
| BOC | 0.0792478 | 0.117072 | 0.4984615 | 0.6994376 | 20 |
| GRAMD1C | -0.0238223 | 0.200889 | 0.9056047 | 0.4561194 | 20 |
| GRAMD1C | -0.00555626 | 0.0829836 | 0.9466167 | 0.4694267 | 20 |
| TIGIT | -0.518385 | 0.420863 | 0.2180543 | 0.7886166 | 18 |
| LSAMP | 0.0398288 | 0.171941 | 0.8168159 | 0.03854513 | 20 |
| POGLUT1 | 0.00397906 | 0.144202 | 0.9779863 | 0.6692833 | 20 |
| FSTL1 | 0.131541 | 0.19137 | 0.4918545 | 0.626158 | 20 |
| HGD | 0.0283762 | 0.262463 | 0.9139043 | 0.9915288 | 20 |
| CD86 | 0.868822 | 0.553567 | 0.1165319 | 0.297545 | 20 |
| DTX3L | 0.482863 | 0.558396 | 0.3871864 | 0.8134935 | 20 |
| PDIA5 | -0.0347859 | 0.0464939 | 0.4543504 | 0.3897821 | 20 |
| PLXNA1 | -0.0037781 | 0.121388 | 0.9751706 | 0.5088902 | 20 |
| RPN1 | -0.00131152 | 0.1944 | 0.9946171 | 0.8823075 | 20 |
| H1FX | 0.623298 | 0.708695 | 0.3791301 | 0.7084249 | 8 |
| PLXND1 | 0.076783 | 0.143006 | 0.5913228 | 0.9279761 | 20 |
| TF | -0.309436 | 0.213475 | 0.147194 | 0.1333615 | 20 |
| RAB6B | -0.111461 | 0.135187 | 0.409657 | 0.1671903 | 20 |
| EPHB1 | 0.0627945 | 0.104596 | 0.5482718 | 0.8874376 | 20 |
| A4GNT | 0.114015 | 0.457011 | 0.8029892 | 0.5743254 | 18 |
| RBP1 | 0.00513799 | 0.400851 | 0.9897732 | 0.4350152 | 20 |
| CLSTN2 | 0.0179244 | 0.164881 | 0.9134314 | 0.375749 | 20 |
| PCOLCE2 | 0.117207 | 0.0657051 | 0.07445148 | 0.1865229 | 20 |
| PLOD2 | -0.249183 | 0.350874 | 0.4775945 | 0.3338108 | 20 |
| CPB1 | 0.102532 | 0.143619 | 0.4752783 | 0.3423327 | 20 |
| RARRES1 | -0.0115279 | 0.0384188 | 0.7641317 | 0.8068782 | 20 |
| SLITRK3 | 0.131323 | 0.0859262 | 0.1264336 | 0.2292387 | 20 |
| BCHE | 0.0760817 | 0.0723877 | 0.2932449 | 0.2723294 | 20 |
| NLGN1 | -0.114378 | 0.230115 | 0.6191562 | 0.05550697 | 17 |
| CHRD | -0.170932 | 0.655332 | 0.7942219 | 0.707001 | 12 |
| DNAJB11 | 0.391279 | 0.281368 | 0.1643373 | 0.6906128 | 17 |
| AHSG | 0.0179768 | 0.0843809 | 0.8312931 | 0.6800783 | 20 |
| FETUB | -0.103541 | 0.167232 | 0.5358223 | 0.8330676 | 20 |
| HRG | 0.00896184 | 0.0349593 | 0.7976801 | 0.9641883 | 20 |
| KNG1 | -0.0516493 | 0.0826343 | 0.5319487 | 0.9749511 | 20 |
| KNG1 | -0.0768052 | 0.122902 | 0.5320164 | 0.9969306 | 20 |
| KNG1 | 0.024497 | 0.123491 | 0.8427552 | 0.6101153 | 20 |
| ADIPOQ | 0.186546 | 0.145646 | 0.2002569 | 0.9565853 | 14 |
| MASP1 | -0.298084 | 0.365525 | 0.4147895 | 0.7438762 | 20 |
| RTP4 | -0.0118783 | 0.0884864 | 0.8932141 | 0.8361774 | 20 |
| IL1RAP | -0.0336188 | 0.0289818 | 0.2460506 | 0.4713736 | 20 |
| CCDC50 | 0.641 | 0.575595 | 0.2654378 | 0.1269917 | 14 |
| CPN2 | 0.040691 | 0.059627 | 0.4949692 | 0.9948031 | 20 |
| LRRC15 | 0.032837 | 0.0845581 | 0.6977669 | 0.8364588 | 20 |
| GP5 | 0.0391425 | 0.283652 | 0.8902448 | 0.5462086 | 20 |
| XXYLT1 | -0.0619124 | 0.14733 | 0.6743185 | 0.7765749 | 20 |
| ACAP2 | -0.0927848 | 0.569739 | 0.8706328 | 0.8765182 | 20 |
| TFRC | 0.256451 | 0.412571 | 0.5342099 | 0.9285338 | 20 |
| CPLX1 | -0.67628 | 0.555126 | 0.2231309 | 0.537255 | 20 |
| IDUA | 0.00623143 | 0.0642912 | 0.9227858 | 0.1962879 | 20 |
| SPON2 | 0.0591054 | 0.100664 | 0.5570996 | 0.8671042 | 20 |
| FGFR3 | -0.0803707 | 0.18614 | 0.6659052 | 0.994295 | 20 |
| SH3BP2 | -0.0428161 | 0.110909 | 0.6994624 | 0.7938207 | 20 |
| HGFAC | 0.000317452 | 0.0375327 | 0.9932516 | 0.06301641 | 20 |
| LRPAP1 | -0.0927655 | 0.156676 | 0.5537938 | 0.6872275 | 5 |
| CYTL1 | 0.0657027 | 0.0972222 | 0.499168 | 0.06545198 | 20 |
| MAN2B2 | -0.0419043 | 0.0376186 | 0.2653114 | 0.5601289 | 20 |
| PSAPL1 | -0.284647 | 0.091334 | 0.001829804 | 0.004026721 | 20 |
| CPZ | 0.275543 | 0.431741 | 0.5233343 | 0.567713 | 20 |
| BST1 | -0.0139876 | 0.0293452 | 0.6336076 | 0.3595569 | 20 |
| FGFBP1 | 0.55244 | 0.456781 | 0.2265007 | 0.1589543 | 20 |
| QDPR | 0.193368 | 0.0851783 | 0.02319828 | 0.5621777 | 20 |
| LAP3 | -0.0300794 | 0.264181 | 0.9093497 | 0.9791236 | 9 |
| LAP3 | -0.0307887 | 0.270411 | 0.9093498 | 0.9790396 | 9 |
| SLIT2 | 0.24827 | 0.298231 | 0.4051409 | 0.9481979 | 20 |
| SOD3 | -0.049859 | 0.0546361 | 0.3614715 | 0.8656671 | 8 |
| SEPSECS | 0.149179 | 0.45325 | 0.7420556 | 0.9541206 | 20 |
| PGM2 | -0.372472 | 0.32888 | 0.2574045 | 0.8823039 | 12 |
| TLR1 | 0.13308 | 0.286094 | 0.6418147 | 0.9308243 | 20 |
| KLB | 0.0140366 | 0.0537353 | 0.7939251 | 0.3484428 | 20 |
| UGDH | -0.0120297 | 0.10315 | 0.907158 | 0.3948732 | 20 |
| TEC | -0.0762828 | 0.265039 | 0.7734866 | 0.2337384 | 20 |
| KIT | 0.78665 | 0.641721 | 0.2202571 | 0.4451649 | 20 |
| KDR | 0.0149011 | 0.0883876 | 0.8661202 | 0.6215022 | 9 |
| PAICS | -0.586454 | 0.500664 | 0.241457 | 0.01221428 | 7 |
| SPINK2 | 0.119283 | 0.146258 | 0.4147483 | 0.5475563 | 12 |
| IGFBP7 | 0.035304 | 0.10426 | 0.7348993 | 0.1238927 | 20 |
| EPHA5 | 0.301167 | 0.343708 | 0.3809057 | 0.8569243 | 20 |
| HTN3 | -0.139159 | 0.251203 | 0.5795988 | 0.8665084 | 15 |
| GC | 0.0900804 | 0.25325 | 0.7220671 | 0.7178913 | 20 |
| AFM | -0.0859858 | 0.164922 | 0.6021071 | 0.03286559 | 15 |
| CXCL8 | -1.6894 | 0.821296 | 0.03968743 | 0.08278192 | 9 |
| CXCL6 | 0.00746157 | 0.0449859 | 0.8682635 | 0.2116487 | 20 |
| PF4V1 | -0.0868452 | 0.0968992 | 0.3701232 | 0.6780651 | 20 |
| CXCL1 | 0.0419045 | 0.0431326 | 0.3312859 | 0.5824341 | 20 |
| PF4 | 0.327904 | 0.347918 | 0.3459502 | 0.1920831 | 6 |
| CXCL5 | -0.304928 | 0.231274 | 0.1873459 | 0.8538619 | 20 |
| BTC | 0.0564031 | 0.199505 | 0.7773953 | 0.8922169 | 18 |
| NAAA | -0.0278408 | 0.0510505 | 0.5855076 | 0.4232638 | 20 |
| CXCL9 | -0.132122 | 0.422492 | 0.7544929 | 0.4264711 | 20 |
| CXCL10 | 0.803377 | 0.425012 | 0.05872529 | 0.3203353 | 20 |
| CXCL11 | -0.134671 | 0.0873127 | 0.1229767 | 0.5877544 | 20 |
| ART3 | 0.0105348 | 0.124643 | 0.9326433 | 0.08514278 | 20 |
| 11-Sep | -0.269704 | 0.480619 | 0.5746883 | 0.4551857 | 20 |
| ANTXR2 | -0.162848 | 0.175467 | 0.3533642 | 0.4935648 | 20 |
| HPSE | -0.413449 | 0.254983 | 0.1049162 | 0.5227003 | 20 |
| NUDT9 | -0.21981 | 0.313097 | 0.482648 | 0.1838998 | 20 |
| SPARCL1 | 0.00382361 | 0.0526598 | 0.9421168 | 0.1706548 | 20 |
| IBSP | 0.0878907 | 0.3017 | 0.7708079 | 0.446042 | 20 |
| SPP1 | -0.071805 | 0.622777 | 0.9082089 | 0.982279 | 4 |
| SNCA | -0.699935 | 0.638448 | 0.2729445 | 0.8698823 | 20 |
| HPGDS | -0.0259375 | 0.0645777 | 0.6879436 | 0.2526939 | 20 |
| UNC5C | -0.0496368 | 0.171587 | 0.7723667 | 0.4314566 | 20 |
| RAP1GDS1 | -0.296265 | 0.605962 | 0.6249008 | 0.7349072 | 20 |
| METAP1 | -0.821528 | 0.454305 | 0.07055673 | 0.7484352 | 20 |
| ADH5 | -0.286027 | 0.215087 | 0.183577 | 0.6545317 | 20 |
| ADH4 | 0.36807 | 0.268957 | 0.1711524 | 0.5644767 | 20 |
| ADH6 | -0.282952 | 0.212754 | 0.1835356 | 0.6087137 | 20 |
| ADH1A | -0.330831 | 0.444562 | 0.4567721 | 0.2161081 | 10 |
| ADH1C | 0.220355 | 0.223037 | 0.3231656 | 0.4032893 | 20 |
| ADH7 | 0.104219 | 0.0985536 | 0.290291 | 0.4588228 | 20 |
| DAPP1 | -0.084853 | 0.346773 | 0.8066937 | 0.4137768 | 20 |
| DNAJB14 | -0.518981 | 0.48496 | 0.2845505 | 0.2891669 | 20 |
| NFKB1 | 0.287945 | 0.452522 | 0.5245728 | 0.8242671 | 20 |
| MANBA | -0.0891345 | 0.0659249 | 0.1763557 | 0.2852989 | 20 |
| BDH2 | -0.231537 | 0.14381 | 0.1073945 | 0.05724513 | 20 |
| PPA2 | -0.00913207 | 0.328279 | 0.9778073 | 0.456717 | 20 |
| NPNT | 0.306412 | 0.167524 | 0.06738968 | 0.8575523 | 16 |
| HADH | -0.0246726 | 0.408802 | 0.9518743 | 0.8672969 | 20 |
| CFI | 0.17783 | 0.145815 | 0.2226307 | 0.4509009 | 20 |
| EGF | -0.411578 | 1.00728 | 0.6828313 | 0.9846782 | 12 |
| ENPEP | 0.10098 | 0.0848685 | 0.2341069 | 0.520237 | 14 |
| CAMK2D | 0.1145 | 0.6554 | 0.861313 | 0.7121585 | 11 |
| FABP2 | -0.00358943 | 0.0401221 | 0.9287143 | 0.6664838 | 20 |
| PDE5A | -0.0202271 | 0.165772 | 0.902885 | 0.960596 | 20 |
| PDE5A | -0.0189497 | 0.155303 | 0.9028848 | 0.9825009 | 20 |
| ANXA5 | 0.439909 | 0.245112 | 0.07269791 | 0.7581354 | 20 |
| FGF2 | 0.01254 | 0.0606698 | 0.8362493 | 0.7677942 | 20 |
| PCDH10 | -0.329499 | 0.218068 | 0.1307913 | 0.07567636 | 10 |
| HHIP | -0.258494 | 0.215766 | 0.2309052 | 0.2623697 | 20 |
| SMAD1 | -0.934352 | 0.597664 | 0.1179724 | 0.05963211 | 20 |
| ARFIP1 | 0.00117516 | 0.128464 | 0.9927012 | 0.0916375 | 20 |
| TLR2 | -0.0695025 | 0.434289 | 0.8728516 | 0.8930105 | 14 |
| FGG | -0.323198 | 0.214387 | 0.131672 | 0.748932 | 20 |
| PPID | -0.173538 | 0.12538 | 0.1663282 | 0.02116033 | 20 |
| TLL1 | 0.908553 | 0.534151 | 0.08895627 | 0.7091227 | 11 |
| SPOCK3 | 0.115927 | 0.113765 | 0.3081997 | 0.4227795 | 16 |
| VEGFC | 0.0233574 | 0.152343 | 0.8781451 | 0.9060998 | 20 |
| DCTD | -0.150013 | 0.342367 | 0.6612674 | 0.445271 | 20 |
| ENPP6 | 0.246574 | 0.540696 | 0.6483681 | 0.4092482 | 20 |
| IRF2 | -1.4777 | 0.812582 | 0.06898356 | 0.3638591 | 10 |
| CASP3 | 0.246286 | 0.189788 | 0.1943923 | 0.7877472 | 20 |
| TLR3 | 0.0625069 | 0.049112 | 0.2031097 | 0.1834442 | 20 |
| KLKB1 | -0.0361795 | 0.0723649 | 0.6171033 | 0.7557751 | 20 |
| F11 | 0.0568233 | 0.086404 | 0.5107654 | 0.9029041 | 20 |
| PDCD6 | -0.000368402 | 0.40263 | 0.9992699 | 0.07217551 | 3 |
| SEMA5A | -0.0697212 | 0.0463554 | 0.1325668 | 0.0396444 | 20 |
| CCT5 | 0.0914542 | 0.566963 | 0.8718529 | 0.2883448 | 17 |
| CMBL | 0.104669 | 0.194692 | 0.5908452 | 0.5506476 | 20 |
| TRIO | 0.610752 | 0.67006 | 0.362038 | 0.9130828 | 20 |
| SUB1 | 0.0317064 | 0.530148 | 0.9523095 | 0.6725507 | 14 |
| C1QTNF3 | -0.134106 | 0.210997 | 0.525047 | 0.9102903 | 20 |
| IL7R | 0.216769 | 0.369641 | 0.5575854 | 0.3173208 | 20 |
| LIFR | -0.028075 | 0.236954 | 0.9056852 | 0.2995401 | 17 |
| OSMR | 0.0993857 | 0.256971 | 0.698935 | 0.06631488 | 20 |
| C9 | -0.184042 | 0.331997 | 0.579339 | 0.2150317 | 6 |
| C7 | -0.0409616 | 0.0406599 | 0.3137324 | 0.8788337 | 17 |
| C6 | -0.0661128 | 0.235876 | 0.7792576 | 0.5753718 | 14 |
| GHR | 0.0942772 | 0.154823 | 0.5425676 | 0.4938527 | 20 |
| CCL28 | -0.0779096 | 0.521588 | 0.8812618 | 0.8108368 | 19 |
| PELO | 0.0322728 | 0.703473 | 0.9634088 | 0.8657526 | 4 |
| ITGA2 | 0.110876 | 0.327565 | 0.7349975 | 0.1217433 | 20 |
| ESM1 | -0.715673 | 0.225694 | 0.001519237 | 0.1664751 | 20 |
| GZMK | 0.130862 | 0.200134 | 0.5131949 | 0.3964396 | 20 |
| GZMA | 0.11449 | 0.235411 | 0.6267262 | 0.4908933 | 7 |
| IL6ST | -0.0779353 | 0.118871 | 0.5120641 | 0.7950179 | 20 |
| HEXB | 0.0904042 | 0.115512 | 0.433841 | 0.3268428 | 20 |
| CRHBP | 0.0898063 | 0.0553 | 0.104379 | 0.06170227 | 20 |
| ARSB | -0.0322689 | 0.223219 | 0.8850569 | 0.8948941 | 20 |
| THBS4 | -0.287922 | 0.129568 | 0.02627181 | 0.8458699 | 20 |
| CKMT2 | 0.58041 | 0.529544 | 0.2730546 | 0.1953331 | 11 |
| XRCC4 | 0.0284624 | 0.236729 | 0.9042992 | 0.8282646 | 20 |
| HAPLN1 | -0.0887709 | 0.282914 | 0.753693 | 0.4639368 | 14 |
| EDIL3 | 0.226881 | 0.47944 | 0.6360558 | 0.9977383 | 20 |
| CCNH | -0.0975289 | 0.34267 | 0.7759392 | 0.4702798 | 20 |
| CETN3 | -0.113919 | 0.457689 | 0.803438 | 0.9694504 | 20 |
| FAM172A | 0.137374 | 0.759392 | 0.8564461 | 0.8834543 | 20 |
| ARSK | -0.381358 | 0.339704 | 0.2616001 | 0.9745848 | 13 |
| RFESD | -0.212611 | 0.569026 | 0.7086717 | 0.3261265 | 6 |
| GLRX | -0.266884 | 0.436718 | 0.5411247 | 0.2712655 | 20 |
| PCSK1 | -0.00882403 | 0.0356495 | 0.8045045 | 0.4853568 | 20 |
| ERAP1 | 0.000322108 | 0.0296137 | 0.9913216 | 0.5177149 | 20 |
| ERAP2 | -0.0162321 | 0.0269864 | 0.5475119 | 0.1338879 | 20 |
| RGMB | 0.21457 | 0.20128 | 0.2864117 | 0.985074 | 20 |
| PAM | -0.0724894 | 0.0631805 | 0.2512421 | 0.9500581 | 20 |
| NUDT12 | -0.0817762 | 0.0852279 | 0.3373067 | 0.3298982 | 20 |
| EFNA5 | 0.12548 | 0.23119 | 0.5872954 | 0.1170329 | 20 |
| FER | 0.2576 | 0.398163 | 0.5176514 | 0.2381029 | 20 |
| SEMA6A | -0.831129 | 0.352264 | 0.01830475 | 0.09746837 | 20 |
| TNFAIP8 | -0.0168966 | 0.192448 | 0.9300372 | 0.8895893 | 20 |
| PPIC | -0.12027 | 0.0705831 | 0.08838948 | 0.3354107 | 20 |
| ISOC1 | -0.316157 | 0.190441 | 0.09688857 | 0.1446991 | 20 |
| HINT1 | -0.07949 | 0.331451 | 0.8104663 | 0.05593435 | 20 |
| PDLIM4 | 0.310061 | 0.199465 | 0.1200743 | 0.623126 | 20 |
| LEAP2 | 0.0248772 | 0.102169 | 0.8076252 | 0.7457001 | 20 |
| FSTL4 | 0.945 | 0.551009 | 0.08633888 | 0.5558128 | 5 |
| TXNDC15 | 0.00379979 | 0.0751994 | 0.9597005 | 0.1419038 | 20 |
| PCBD2 | -0.300581 | 0.935284 | 0.7479225 | 0.3851072 | 6 |
| IL9 | -0.419119 | 0.371388 | 0.2591003 | 0.9059225 | 20 |
| LECT2 | -0.0113302 | 0.0511596 | 0.824728 | 0.1204566 | 20 |
| TGFBI | -0.0828278 | 0.0617577 | 0.1798645 | 0.9134195 | 20 |
| LRRTM2 | -0.286544 | 0.361778 | 0.4283351 | 0.8354215 | 20 |
| PACAP | 0.0406139 | 0.252651 | 0.8722897 | 0.9874283 | 18 |
| HBEGF | -0.347341 | 0.574721 | 0.5456014 | 0.4485072 | 20 |
| SRA1 | -0.171085 | 0.256748 | 0.5051862 | 0.08109485 | 20 |
| CD14 | 0.0948902 | 0.131579 | 0.4708085 | 0.5830716 | 20 |
| CD14 | 0.119486 | 0.165753 | 0.4709907 | 0.5139728 | 20 |
| GNPDA1 | -0.127782 | 0.210539 | 0.5438997 | 0.9504697 | 20 |
| SPINK1 | -0.316861 | 0.275346 | 0.2498265 | 0.307199 | 20 |
| SPINK5 | -0.261611 | 0.237234 | 0.2701343 | 0.5815944 | 20 |
| SPINK6 | -0.0478235 | 0.0479203 | 0.3182883 | 0.4545863 | 19 |
| SPINK7 | 0.017805 | 0.241032 | 0.941114 | 0.02717126 | 20 |
| ABLIM3 | 0.14861 | 0.36702 | 0.6855436 | 0.517869 | 18 |
| IL17B | -0.22316 | 0.455455 | 0.6241533 | 0.6645937 | 12 |
| CSF1R | -0.487551 | 0.271104 | 0.07211482 | 0.04001704 | 20 |
| PDGFRB | 0.0201178 | 0.0246261 | 0.4139691 | 0.2303575 | 20 |
| NDST1 | 0.0758734 | 0.213326 | 0.7220889 | 0.3249156 | 20 |
| GM2A | -0.0236455 | 0.128414 | 0.8539076 | 0.6375442 | 20 |
| SPARC | 0.501146 | 1.21317 | 0.6795415 | 0.2404077 | 5 |
| ATOX1 | -0.16025 | 0.427898 | 0.7080281 | 0.5739357 | 20 |
| G3BP1 | -0.365805 | 0.450156 | 0.416437 | 0.9038595 | 20 |
| TIMD4 | 0.0208004 | 0.124817 | 0.8676481 | 0.7753582 | 20 |
| HAVCR1 | 0.0247487 | 0.0873439 | 0.77691 | 0.96313 | 20 |
| HAVCR2 | -0.000266197 | 0.0593646 | 0.9964222 | 0.1099952 | 20 |
| THG1L | -0.0710313 | 0.0813977 | 0.3828569 | 0.9871071 | 20 |
| CLINT1 | -0.232778 | 0.573527 | 0.6848379 | 0.7954016 | 11 |
| UBLCP1 | 0.18136 | 0.230209 | 0.4308123 | 0.9794413 | 8 |
| IL12B | 0.0624032 | 0.0801993 | 0.4365096 | 0.03238869 | 20 |
| CPLX2 | 0.145028 | 0.520792 | 0.7806464 | 0.993564 | 14 |
| LMAN2 | 0.271618 | 0.320105 | 0.3961435 | 0.3552453 | 20 |
| B4GALT7 | 0.323628 | 0.464795 | 0.4862521 | 0.4206965 | 16 |
| HNRNPAB | -0.26068 | 0.351312 | 0.4580747 | 0.9320881 | 13 |
| MGAT4B | -0.17397 | 0.216625 | 0.4219213 | 0.1907678 | 20 |
| MAPK9 | -0.0469334 | 0.195244 | 0.8100334 | 0.1172982 | 20 |
| SCGB3A1 | 0.0605709 | 0.250142 | 0.808667 | 0.9399126 | 20 |
| FLT4 | -0.0471166 | 0.230744 | 0.8382015 | 0.396594 | 20 |
| BTNL8 | -0.0440518 | 0.192186 | 0.8187018 | 0.3719208 | 20 |
| GMDS | -0.752059 | 0.626231 | 0.2297787 | 0.3148711 | 8 |
| SERPINB1 | 0.69408 | 0.371345 | 0.06160922 | 0.7113416 | 20 |
| NQO2 | 0.0146596 | 0.0429442 | 0.7328303 | 0.08353409 | 20 |
| BPHL | -0.251463 | 0.261062 | 0.3354308 | 0.02966731 | 20 |
| ECI2 | -0.0132772 | 0.0767131 | 0.8625918 | 0.3807738 | 20 |
| TXNDC5 | 0.489138 | 0.337424 | 0.1471631 | 0.006558077 | 5 |
| GMPR | 0.0273147 | 0.0245986 | 0.2668185 | 0.106463 | 20 |
| ACOT13 | -0.529528 | 0.525039 | 0.3131904 | 0.1996284 | 20 |
| SCGN | -0.124918 | 0.453739 | 0.7830791 | 0.3977706 | 20 |
| BTN3A1 | 0.882486 | 0.80998 | 0.2759262 | 0.3538251 | 20 |
| BTN3A3 | 0.0270827 | 0.0436434 | 0.5348983 | 0.2859355 | 20 |
| LTA | 0.863494 | 0.804434 | 0.2830834 | 0.1451525 | 20 |
| MLN | -0.0420742 | 0.0661582 | 0.5248005 | 0.744013 | 20 |
| SCUBE3 | -0.00812868 | 0.157031 | 0.9587162 | 0.9427743 | 20 |
| DEF6 | -0.0740658 | 0.206846 | 0.7202893 | 0.9269199 | 20 |
| CLPS | 0.0022445 | 0.0784849 | 0.9771853 | 0.9776321 | 20 |
| MAPK13 | 0.0403465 | 0.299805 | 0.8929473 | 0.5912227 | 20 |
| PPIL1 | -0.0659156 | 0.074003 | 0.3730821 | 0.9675468 | 9 |
| MDGA1 | -0.0213785 | 0.0273403 | 0.4342491 | 0.1708143 | 20 |
| GLO1 | 0.00924111 | 0.305106 | 0.9758372 | 0.9344325 | 20 |
| TREML1 | -0.0621905 | 0.168383 | 0.7118751 | 0.9241059 | 8 |
| TREML2 | -0.088796 | 0.0656446 | 0.1761582 | 0.7572542 | 20 |
| TREM1 | -0.031529 | 0.0994937 | 0.7513238 | 0.7031048 | 20 |
| CNPY3 | 0.261981 | 0.264927 | 0.3227227 | 0.486682 | 20 |
| GNMT | 0.241478 | 0.130914 | 0.0651022 | 0.8282192 | 20 |
| PTK7 | 0.711926 | 0.349822 | 0.04184009 | 0.2941306 | 20 |
| RCL | -0.0988895 | 0.312182 | 0.7514189 | 0.8792425 | 20 |
| DLK2 | -0.0789232 | 0.200752 | 0.6942172 | 0.392669 | 20 |
| VEGFA | 0.00231657 | 0.0961284 | 0.9807739 | 0.5246826 | 20 |
| VEGFA | 0.00125438 | 0.052052 | 0.9807739 | 0.524445 | 20 |
| ENPP5 | 0.00896462 | 0.0436271 | 0.8371951 | 0.9687228 | 20 |
| PLA2G7 | 0.351717 | 0.409612 | 0.3905281 | 0.4784723 | 3 |
| ADGRF5 | 0.0347253 | 0.0413855 | 0.4014307 | 0.04488827 | 20 |
| TNFRSF21 | 0.32172 | 0.285547 | 0.2598792 | 0.9179262 | 18 |
| CRISP2 | -0.0487298 | 0.0505613 | 0.3351586 | 0.8083991 | 20 |
| GSTA1 | -0.0421994 | 0.0796459 | 0.5962246 | 0.1033662 | 20 |
| GFRAL | 0.0205118 | 0.209784 | 0.9221105 | 0.9957277 | 20 |
| COL9A1 | -0.418155 | 0.446333 | 0.3488269 | 0.54119 | 20 |
| SMAP1 | -0.00663416 | 0.0942382 | 0.943877 | 0.8814851 | 20 |
| CD109 | 0.0905904 | 0.055004 | 0.09956262 | 0.3424377 | 20 |
| SH3BGRL2 | -0.0844594 | 0.280462 | 0.7633047 | 0.08124505 | 20 |
| NT5E | -0.0804786 | 0.0888493 | 0.3650486 | 0.5298676 | 6 |
| MANEA | 0.0584705 | 0.0394199 | 0.138002 | 0.3242902 | 20 |
| PREP | 0.028569 | 0.178068 | 0.8725354 | 0.1329562 | 20 |
| RTN4IP1 | -0.947338 | 0.473036 | 0.04521189 | 0.8162034 | 20 |
| FOXO3 | -0.55855 | 0.548473 | 0.3085002 | 0.1164693 | 20 |
| METTL24 | -0.297582 | 0.31197 | 0.3401451 | 0.5587973 | 5 |
| COL10A1 | -0.0563822 | 0.138823 | 0.6846355 | 0.1290802 | 20 |
| ASF1A | 0.00442107 | 0.251152 | 0.9859555 | 0.9000502 | 20 |
| SMPDL3A | 0.104979 | 0.122535 | 0.3915961 | 0.3190199 | 15 |
| RSPO3 | -0.00908113 | 0.134101 | 0.9460095 | 0.0108539 | 20 |
| RSPO3 | -0.00826087 | 0.121988 | 0.9460095 | 0.009342811 | 20 |
| ARG1 | -0.0594741 | 0.303139 | 0.8444582 | 0.4413332 | 20 |
| STX7 | 0.275557 | 0.244694 | 0.2601106 | 0.2465998 | 20 |
| VNN2 | 0.0647765 | 0.0428496 | 0.1306059 | 0.332583 | 20 |
| IL20RA | -0.174301 | 0.552104 | 0.7522283 | 0.6869731 | 15 |
| IL22RA2 | -0.0355278 | 0.280239 | 0.899117 | 0.7261892 | 18 |
| IFNGR1 | -0.350199 | 0.471246 | 0.4574004 | 0.9331015 | 8 |
| TNFAIP3 | 0.181019 | 0.26874 | 0.5005767 | 0.302791 | 20 |
| VTA1 | 0.319151 | 0.584777 | 0.5852268 | 0.8350897 | 20 |
| UST | -0.249499 | 0.224378 | 0.2661552 | 0.1897566 | 20 |
| LRP11 | 0.050012 | 0.0508435 | 0.3252902 | 0.8981829 | 20 |
| LRP11 | 0.00809171 | 0.0353334 | 0.8188611 | 0.2938843 | 20 |
| MTRF1L | -0.164902 | 0.182734 | 0.3668347 | 0.8791569 | 20 |
| ACAT2 | -0.0639613 | 0.108744 | 0.5564105 | 0.2942448 | 20 |
| IGF2R | -0.100742 | 0.0755697 | 0.1825011 | 0.03692231 | 20 |
| PLG | -0.0889632 | 0.13505 | 0.5100593 | 0.008236572 | 20 |
| PLG | -0.100805 | 0.15304 | 0.5100968 | 0.02115788 | 20 |
| RNASET2 | -0.110696 | 0.100826 | 0.2722489 | 0.4048094 | 20 |
| SMOC2 | 0.0101021 | 0.0990053 | 0.9187278 | 0.6551202 | 20 |
| THBS2 | -0.202378 | 0.367353 | 0.5816964 | 0.4180608 | 20 |
| THBS2 | -0.0318425 | 0.0594776 | 0.5923941 | 0.3209644 | 20 |
| DLL1 | -0.18606 | 0.291886 | 0.5238384 | 0.1916418 | 20 |
| PSMB1 | -0.0117486 | 0.0926796 | 0.8991255 | 0.8978678 | 20 |
| PDGFA | -0.994212 | 0.598303 | 0.09656894 | 0.2720625 | 8 |
| SNX8 | 0.113064 | 0.284788 | 0.6913586 | 0.3483916 | 20 |
| CHST12 | 0.279432 | 0.190956 | 0.1433774 | 0.04920914 | 20 |
| COL28A1 | 0.47861 | 0.647593 | 0.4598704 | 0.6640742 | 20 |
| TMEM106B | 0.319819 | 0.26975 | 0.2357761 | 0.1890663 | 20 |
| SCIN | 0.126796 | 0.331325 | 0.701948 | 0.3471418 | 20 |
| GPNMB | -0.018512 | 0.134707 | 0.8906955 | 0.1003653 | 20 |
| GPNMB | -0.0860799 | 0.0771986 | 0.2648317 | 0.5030889 | 20 |
| CCDC126 | -0.116924 | 0.118094 | 0.32213 | 0.5119065 | 20 |
| NT5C3A | 0.315626 | 0.385541 | 0.4129814 | 0.231292 | 20 |
| BMPER | -0.522414 | 0.305114 | 0.08686125 | 0.5587201 | 20 |
| ELMO1 | -0.600232 | 0.318648 | 0.05960734 | 0.751365 | 20 |
| SFRP4 | -0.0799926 | 0.145039 | 0.5812753 | 0.4883076 | 17 |
| INHBA | 0.270016 | 0.339374 | 0.4262478 | 0.1305619 | 20 |
| INHBA | 0.272321 | 0.342287 | 0.4262688 | 0.1204952 | 20 |
| BLVRA | -0.164226 | 0.309703 | 0.5959254 | 0.9213299 | 4 |
| DBNL | -0.504877 | 0.434729 | 0.2454951 | 0.3553255 | 20 |
| POLM | -0.0464181 | 0.133693 | 0.7284412 | 0.3007699 | 20 |
| NUDCD3 | -0.481518 | 0.468923 | 0.304487 | 0.882632 | 20 |
| CCM2 | 0.0416257 | 0.484037 | 0.931469 | 0.9698335 | 20 |
| IGFBP1 | 0.0463287 | 0.388604 | 0.9051025 | 0.3770888 | 20 |
| IGFBP3 | -0.119318 | 0.0833315 | 0.1521872 | 0.1195858 | 10 |
| UPP1 | 0.179824 | 0.275316 | 0.5136564 | 0.8486266 | 20 |
| VWC2 | 0.242121 | 0.139802 | 0.08329374 | 0.1850498 | 11 |
| GRB10 | -0.348632 | 1.10319 | 0.7519861 | 0.9549215 | 11 |
| EGFR | 0.122045 | 0.28165 | 0.6647806 | 0.2884338 | 20 |
| VOPP1 | 0.724967 | 0.417325 | 0.08235584 | 0.9345342 | 12 |
| GUSB | -0.0941263 | 0.208759 | 0.6520726 | 0.6463797 | 20 |
| ASL | 0.204899 | 0.229924 | 0.3728425 | 0.494906 | 17 |
| TPST1 | 0.116585 | 0.100736 | 0.2471384 | 0.3776682 | 12 |
| SBDS | 0.415234 | 0.447095 | 0.3530246 | 0.3453405 | 20 |
| NCF1 | 0.047652 | 0.140081 | 0.7337247 | 0.4975434 | 20 |
| POR | -0.11686 | 0.15893 | 0.4621637 | 0.1584933 | 20 |
| HSPB1 | 0.00979853 | 0.0696916 | 0.8881871 | 0.4752344 | 20 |
| FGL2 | 0.614088 | 0.680207 | 0.3666345 | 0.2758062 | 20 |
| MAGI2 | 0.285722 | 0.406845 | 0.4825003 | 0.1246905 | 20 |
| CD36 | -0.0882303 | 0.176618 | 0.6173881 | 0.3975137 | 20 |
| SEMA3C | 0.177276 | 0.272162 | 0.5148124 | 0.7901595 | 20 |
| HGF | 0.334876 | 0.26123 | 0.1998704 | 0.09817197 | 20 |
| SEMA3E | -0.0716536 | 0.0427317 | 0.09357645 | 0.3641067 | 20 |
| ADAM22 | 0.235415 | 0.105228 | 0.02527428 | 0.004407173 | 20 |
| SRI | -0.260651 | 0.298887 | 0.3831687 | 0.07911026 | 20 |
| TFPI2 | 0.600156 | 0.483654 | 0.2146504 | 0.8074916 | 6 |
| TAC1 | 0.0608367 | 0.165858 | 0.7137686 | 0.5499449 | 20 |
| NPTX2 | -0.0221575 | 0.254191 | 0.9305373 | 0.4369407 | 20 |
| ARPC1B | 0.106689 | 0.292316 | 0.7151283 | 0.5982421 | 20 |
| AZGP1 | -0.0290303 | 0.125996 | 0.8177761 | 0.6750936 | 20 |
| AP4M1 | 0.491413 | 0.650944 | 0.4502942 | 0.3604571 | 20 |
| PCOLCE | 0.0831034 | 0.178061 | 0.6407046 | 0.4957485 | 20 |
| EPO | 0.335559 | 0.415292 | 0.419086 | 0.8733768 | 20 |
| EPHB4 | 0.186869 | 0.2592 | 0.4709442 | 0.1281361 | 20 |
| SERPINE1 | 0.481567 | 0.481497 | 0.3172398 | 0.4718136 | 20 |
| PLOD3 | -0.20189 | 0.278827 | 0.4690229 | 0.5881926 | 8 |
| FIS1 | -0.0963892 | 0.1911 | 0.613987 | 0.3728068 | 20 |
| PIK3CG | 0.339132 | 0.592143 | 0.5668343 | 0.2627797 | 8 |
| NRCAM | -0.367915 | 0.464115 | 0.4279396 | 0.3730817 | 20 |
| DNAJB9 | 0.173075 | 0.565153 | 0.7594184 | 0.6317739 | 4 |
| TES | 0.290503 | 0.578653 | 0.6156443 | 0.9800168 | 7 |
| MET | 0.0458949 | 0.223614 | 0.8373837 | 0.129253 | 20 |
| CPA4 | -0.0533964 | 0.0245507 | 0.02963443 | 0.441563 | 20 |
| PLXNA4 | -0.282562 | 0.332477 | 0.3953986 | 0.6442607 | 20 |
| AKR1B1 | -0.0736157 | 0.329955 | 0.8234512 | 0.4508665 | 11 |
| BPGM | 1.31354 | 0.629519 | 0.03692683 | 0.4547218 | 12 |
| PTN | 0.137965 | 0.150631 | 0.3597103 | 0.01145664 | 20 |
| AKR1D1 | 0.77454 | 0.634185 | 0.2219663 | 0.4165055 | 8 |
| PRSS2 | -0.103809 | 0.140496 | 0.459982 | 0.1952862 | 20 |
| EPHB6 | 0.150911 | 0.149943 | 0.3141978 | 0.51297 | 20 |
| PIP | -0.02074 | 0.066387 | 0.7547285 | 0.09036286 | 20 |
| ZYX | 0.198341 | 0.411944 | 0.6301787 | 0.6935746 | 20 |
| EPHA1 | 0.000522807 | 0.0334578 | 0.9875329 | 0.3417265 | 20 |
| CNTNAP2 | -0.0955274 | 0.0705575 | 0.1757702 | 0.4089749 | 20 |
| RARRES2 | -0.00999342 | 0.129273 | 0.9383812 | 0.5628177 | 20 |
| AOC1 | -0.0197876 | 0.0389863 | 0.6117676 | 0.664079 | 20 |
| SHH | 0.314808 | 0.310903 | 0.3112703 | 0.9166295 | 20 |
| DNAJB6 | 0.286692 | 0.569417 | 0.6146241 | 0.7173197 | 20 |
| MYOM2 | 3.51754 | 2.56712 | 0.1706155 | 0.1049998 | 20 |
| ANGPT2 | -0.223786 | 0.225317 | 0.3206103 | 0.2654291 | 20 |
| DEFB1 | -0.157921 | 0.0821905 | 0.05468138 | 0.1492156 | 20 |
| DEFA5 | -0.031776 | 0.432543 | 0.9414375 | 0.6454595 | 3 |
| DEFB104A | 0.0344755 | 0.269838 | 0.898336 | 0.9858624 | 7 |
| DEFB4A | -0.281978 | 0.258354 | 0.2750789 | 0.7509605 | 6 |
| CTSB | 0.0438796 | 0.0586564 | 0.4544129 | 0.5606155 | 20 |
| MSR1 | -0.0278904 | 0.166579 | 0.8670316 | 0.2735827 | 20 |
| PDGFRL | -0.0974775 | 0.0850061 | 0.2515007 | 0.9946832 | 20 |
| FGL1 | -0.011795 | 0.0584138 | 0.8399789 | 0.8518035 | 20 |
| GFRA2 | 0.0897051 | 0.0930106 | 0.3348146 | 0.9707253 | 20 |
| DOK2 | 2.06262 | 1.02001 | 0.0431597 | 0.3081957 | 17 |
| BMP1 | -0.410682 | 0.383181 | 0.2838233 | 0.665048 | 19 |
| STC1 | -1.20829 | 0.526212 | 0.02166368 | 0.04254241 | 20 |
| SCARA5 | 0.204921 | 0.114745 | 0.07411742 | 0.6584244 | 20 |
| GSR | -0.252935 | 0.309202 | 0.4133438 | 0.6070443 | 20 |
| FUT10 | -0.00109317 | 0.0427035 | 0.9795771 | 0.9911738 | 20 |
| UNC5D | 0.0315517 | 0.288299 | 0.9128528 | 0.8195782 | 20 |
| FGFR1 | -0.340943 | 0.315155 | 0.2793295 | 0.1232708 | 20 |
| ADAM9 | 0.192313 | 0.841451 | 0.8192194 | 0.6025934 | 3 |
| IDO1 | 0.0164693 | 0.531354 | 0.9752736 | 0.418296 | 15 |
| SFRP1 | 0.142635 | 0.181481 | 0.4318975 | 0.8317082 | 20 |
| PLAT | 0.108492 | 0.271821 | 0.6897977 | 0.5376572 | 20 |
| PENK | -0.00289461 | 0.0598315 | 0.9614139 | 0.07255411 | 20 |
| IMPAD1 | -0.0459295 | 0.101966 | 0.6523948 | 0.9025728 | 20 |
| UBXN2B | 0.5102 | 0.497469 | 0.3050837 | 0.4838393 | 19 |
| CA8 | -0.097552 | 0.0689011 | 0.1568265 | 0.2089256 | 20 |
| ASPH | -0.260794 | 0.318085 | 0.41228 | 0.2989806 | 20 |
| GGH | 0.00857443 | 0.076674 | 0.9109585 | 0.3332682 | 20 |
| CRH | 0.730338 | 0.887396 | 0.4105008 | 0.524577 | 16 |
| SGK3 | -0.346772 | 1.32168 | 0.7930342 | 0.7504003 | 18 |
| FABP4 | 0.461558 | 0.409295 | 0.2594509 | 0.4681674 | 20 |
| ZFAND1 | 0.0552627 | 0.177355 | 0.7553491 | 0.7692629 | 20 |
| CA13 | -0.108079 | 0.114019 | 0.3431796 | 0.2993841 | 20 |
| CA1 | 0.331094 | 0.243945 | 0.1747015 | 0.1041767 | 20 |
| CA3 | 0.301555 | 0.195922 | 0.1237659 | 0.7591777 | 20 |
| RMDN1 | -0.0425903 | 0.103298 | 0.6801163 | 0.8305702 | 20 |
| MMP16 | -0.0366583 | 0.449828 | 0.935049 | 0.3580652 | 20 |
| RIPK2 | 0.311512 | 0.441683 | 0.4806334 | 0.7394313 | 20 |
| DECR1 | -0.109876 | 0.253242 | 0.6643782 | 0.7651518 | 7 |
| CDH17 | 0.232999 | 0.111554 | 0.03673797 | 0.6764068 | 20 |
| CPQ | -0.0276369 | 0.0962616 | 0.774034 | 0.7543421 | 20 |
| MATN2 | 0.509844 | 0.344736 | 0.1391559 | 0.3087655 | 20 |
| HRSP12 | -0.073395 | 0.113314 | 0.5171692 | 0.9345541 | 20 |
| RRM2B | -0.0153667 | 0.215514 | 0.9431569 | 0.3367208 | 20 |
| LRP12 | 0.913282 | 0.605975 | 0.1317777 | 0.7903137 | 20 |
| ANGPT1 | 0.262609 | 0.43563 | 0.5466243 | 0.881161 | 20 |
| TNFRSF11B | 0.216368 | 0.19079 | 0.256768 | 0.5686646 | 20 |
| COLEC10 | -0.299115 | 0.979958 | 0.7601888 | 0.6650602 | 14 |
| NOV | 0.934143 | 0.493739 | 0.05849432 | 0.6178374 | 20 |
| ENPP2 | -0.0353524 | 0.184502 | 0.8480479 | 0.2474556 | 20 |
| FAM49B | 0.343572 | 0.580902 | 0.5542216 | 0.9381537 | 20 |
| TG | -0.220434 | 0.382385 | 0.5642973 | 0.1458695 | 20 |
| WISP1 | 0.012816 | 0.0754999 | 0.8652077 | 0.8133778 | 20 |
| ST3GAL1 | -0.0890224 | 0.116789 | 0.4459124 | 0.3455857 | 20 |
| HSF1 | 0.115988 | 0.44941 | 0.7963376 | 0.574577 | 20 |
| GPT | -0.0856445 | 0.568847 | 0.8803244 | 0.7330065 | 20 |
| CD274 | 0.0598065 | 0.1318 | 0.6499952 | 0.7116368 | 20 |
| PDCD1LG2 | -0.0543902 | 0.0932499 | 0.559709 | 0.7609462 | 20 |
| ADAMTSL1 | -0.0489343 | 0.117523 | 0.6771305 | 0.19238 | 20 |
| TEK | -0.242235 | 0.209564 | 0.2477217 | 0.3043571 | 20 |
| DDX58 | 0.208168 | 0.169487 | 0.2193617 | 0.9765048 | 20 |
| B4GALT1 | 0.177543 | 0.148664 | 0.2323782 | 0.8999928 | 20 |
| SPINK4 | 0.0629717 | 0.145646 | 0.6654792 | 0.710501 | 20 |
| PRSS3 | -0.0646675 | 0.0635162 | 0.3086184 | 0.7324768 | 20 |
| NUDT2 | 0.0879183 | 0.111092 | 0.4287103 | 0.6685629 | 20 |
| KIAA1161 | 0.0186563 | 0.24077 | 0.9382369 | 0.2611401 | 20 |
| CNTFR | -0.0286128 | 0.115413 | 0.8041996 | 0.2737918 | 15 |
| IL11RA | -0.00930374 | 0.0857283 | 0.9135786 | 0.7380213 | 20 |
| CCL27 | 0.171698 | 0.590272 | 0.7711436 | 0.6919546 | 11 |
| CCL19 | 0.951518 | 0.643475 | 0.1392162 | 0.1595768 | 20 |
| CCL21 | 0.177101 | 0.267313 | 0.5076368 | 0.5816415 | 20 |
| CD72 | 0.755632 | 0.386401 | 0.05051655 | 0.7141454 | 20 |
| MSMP | -0.390399 | 0.463627 | 0.3997574 | 0.8145636 | 20 |
| GLIPR2 | 0.0109115 | 0.115441 | 0.9246959 | 0.01056967 | 20 |
| GRHPR | 0.0768391 | 0.112882 | 0.4960586 | 0.9313534 | 20 |
| TMEM2 | -0.073288 | 0.199419 | 0.7132408 | 0.7708032 | 20 |
| ALDH1A1 | 0.353941 | 0.326382 | 0.2781699 | 0.7352406 | 20 |
| ANXA1 | -0.0158933 | 0.229357 | 0.9447549 | 0.5144098 | 17 |
| NRK1 | -0.0803275 | 0.202924 | 0.6922157 | 0.5794171 | 20 |
| RFK | 0.271218 | 0.282659 | 0.3372961 | 0.2219934 | 20 |
| PSAT1 | 0.205226 | 0.242901 | 0.3981687 | 0.8562537 | 20 |
| NTRK2 | 0.335544 | 0.490606 | 0.4940146 | 0.0293781 | 20 |
| GOLM1 | -0.390248 | 0.1636 | 0.01706175 | 0.1467558 | 20 |
| GOLM1 | -0.564645 | 0.237815 | 0.0175821 | 0.1297844 | 20 |
| GAS1 | 0.163499 | 0.338398 | 0.6289839 | 0.8210206 | 20 |
| SEMA4D | -0.047931 | 0.0378107 | 0.2049203 | 0.4945294 | 20 |
| SYK | -0.454443 | 0.383455 | 0.2359669 | 0.6622357 | 20 |
| SYK | -0.417006 | 0.350018 | 0.2335035 | 0.7619363 | 20 |
| ROR2 | 0.38652 | 0.209727 | 0.06533409 | 0.5337521 | 20 |
| OGN | -0.14929 | 0.080002 | 0.06203022 | 0.2141638 | 20 |
| OMD | 0.737855 | 0.795596 | 0.3537065 | 0.8360311 | 20 |
| ASPN | 0.140961 | 0.0809443 | 0.08160272 | 0.1955391 | 20 |
| FBP2 | 0.0309 | 0.237389 | 0.8964351 | 0.3431846 | 20 |
| FBP1 | 0.317933 | 0.344926 | 0.3566632 | 0.9060266 | 20 |
| HABP4 | -0.0604036 | 0.0972408 | 0.5344839 | 0.4906512 | 20 |
| CTSV | -0.210054 | 0.179792 | 0.2426799 | 0.06945235 | 20 |
| NANS | -0.164467 | 0.702974 | 0.8150168 | 0.8038407 | 9 |
| COL15A1 | 0.214138 | 0.197267 | 0.2776903 | 0.4749736 | 20 |
| TMEFF1 | -0.352003 | 0.674803 | 0.6019229 | 0.9132549 | 20 |
| ALDOB | -0.316449 | 0.310456 | 0.3080581 | 0.9981303 | 11 |
| RAD23B | 0.932205 | 0.624916 | 0.1357704 | 0.9941496 | 20 |
| SVEP1 | -0.141693 | 0.0950195 | 0.1359089 | 0.4893548 | 15 |
| SVEP1 | -0.131902 | 0.0884402 | 0.1358509 | 0.4081343 | 15 |
| MUSK | 0.104865 | 0.439717 | 0.8115067 | 0.9202633 | 20 |
| PTGR1 | 0.0299842 | 0.0716949 | 0.6757866 | 0.07128495 | 20 |
| HDHD3 | 0.0612803 | 0.162419 | 0.7059521 | 0.138263 | 18 |
| ALAD | 0.133332 | 0.127968 | 0.297448 | 0.5866531 | 17 |
| AMBP | 0.0251259 | 0.274418 | 0.927047 | 0.8153292 | 20 |
| TNFSF15 | -0.555152 | 0.657037 | 0.3981484 | 0.6728865 | 20 |
| TNFSF8 | 0.0563677 | 0.210633 | 0.7889983 | 0.9674607 | 20 |
| TNC | 0.00641273 | 0.0330595 | 0.8461951 | 0.3968418 | 20 |
| C5 | -0.0370096 | 0.254633 | 0.8844386 | 0.2797764 | 20 |
| C5 | 0.52236 | 0.278238 | 0.06046472 | 0.6805728 | 20 |
| ENG | -0.0750011 | 0.155116 | 0.6287296 | 0.8768491 | 20 |
| AK1 | -0.836257 | 0.738008 | 0.2571609 | 0.3807117 | 20 |
| ST6GALNAC6 | -0.569063 | 0.60021 | 0.3430754 | 0.5750354 | 20 |
| PTGES2 | -0.907439 | 0.489766 | 0.06391066 | 0.4308752 | 7 |
| LCN2 | 0.229091 | 0.356533 | 0.520515 | 0.4186191 | 9 |
| SH3GLB2 | 0.0368935 | 0.0850332 | 0.6643811 | 0.3296199 | 20 |
| CRAT | 0.342819 | 0.371451 | 0.3560494 | 0.3673246 | 20 |
| AIF1L | -0.0126645 | 0.0597322 | 0.8320904 | 0.6594977 | 20 |
| CEL | 0.384986 | 0.29394 | 0.1902826 | 0.2446057 | 8 |
| OBP2B | 0.0956835 | 0.0824655 | 0.2459328 | 0.9678386 | 20 |
| ADAMTS13 | 0.0607486 | 0.065388 | 0.3528641 | 0.05244421 | 20 |
| ADAMTSL2 | -0.0203742 | 0.257057 | 0.9368261 | 0.8069865 | 20 |
| FCN2 | -0.0372513 | 0.0453272 | 0.4111738 | 0.6696844 | 20 |
| FCN2 | -0.0579382 | 0.0705036 | 0.4112041 | 0.7872604 | 20 |
| FCN1 | -0.00490717 | 0.0879564 | 0.9555084 | 0.8466643 | 20 |
| OLFM1 | 0.0579427 | 0.232818 | 0.8034569 | 0.5407399 | 16 |
| LCN1 | 0.15811 | 0.449907 | 0.7252671 | 0.3235913 | 20 |
| NOTCH1 | 0.488319 | 0.300031 | 0.1036175 | 0.5811469 | 20 |
| PHPT1 | -0.09166 | 0.151344 | 0.5447547 | 0.1482386 | 20 |
| C8G | 0.0861387 | 0.0612404 | 0.1595561 | 0.2546605 | 20 |
| PTGDS | 0.133645 | 0.204963 | 0.5143737 | 0.4938097 | 20 |
| NPDC1 | 0.0931741 | 0.51856 | 0.8574049 | 0.3999676 | 15 |
| DPP7 | -0.0542386 | 0.0651083 | 0.4048152 | 0.3339229 | 20 |
| IDI2 | 0.0233261 | 0.206411 | 0.9100242 | 0.3532116 | 20 |
| IDI1 | 0.782109 | 1.0063 | 0.4370339 | 0.5613635 | 20 |
| AKR1C3 | -0.24771 | 0.22567 | 0.2723506 | 0.9731685 | 20 |
| AKR1C4 | 0.0643782 | 0.0616774 | 0.2965831 | 0.697178 | 20 |
| GDI2 | -0.409717 | 0.31402 | 0.1919787 | 0.6541384 | 20 |
| IL15RA | 0.0829406 | 0.0770171 | 0.2815198 | 0.5100117 | 20 |
| IL15RA | 0.0295142 | 0.559724 | 0.957947 | 0.3594653 | 17 |
| IL2RA | 0.0358348 | 0.537298 | 0.946825 | 0.6554217 | 20 |
| RBM17 | -0.0739216 | 0.126068 | 0.5576331 | 0.8111735 | 20 |
| ITIH5 | 0.134747 | 0.0893388 | 0.1314848 | 0.07545904 | 20 |
| ITIH2 | 0.0507117 | 0.0472641 | 0.2832967 | 0.7170522 | 20 |
| NUDT5 | -0.466662 | 0.405083 | 0.2493141 | 0.6401352 | 20 |
| UCMA | 0.209321 | 0.158723 | 0.187243 | 0.07931183 | 20 |
| CDNF | 0.0480199 | 0.0864319 | 0.578497 | 0.09299037 | 7 |
| ACBD7 | -0.364889 | 0.649833 | 0.5744482 | 0.3788822 | 20 |
| NMT2 | -0.573714 | 0.461105 | 0.2134196 | 0.7816721 | 20 |
| TRDMT1 | -0.0186684 | 0.0924135 | 0.8399094 | 0.02498041 | 20 |
| VIM | -0.141954 | 0.396796 | 0.7205298 | 0.4520422 | 20 |
| ST8SIA6 | -0.317051 | 0.69369 | 0.6476346 | 0.974849 | 10 |
| PIP4K2A | -0.156245 | 0.26725 | 0.5587898 | 0.116139 | 20 |
| NRP1 | 0.0490668 | 0.0983808 | 0.6179603 | 0.04864131 | 20 |
| RET | -0.0553904 | 0.089065 | 0.5340016 | 0.1089282 | 20 |
| CSGALNACT2 | 0.157432 | 0.2713 | 0.5617204 | 0.6543596 | 20 |
| CXCL12 | 0.274538 | 0.220388 | 0.2128741 | 0.5458986 | 20 |
| CXCL12 | 0.506236 | 0.453069 | 0.2638456 | 0.3402781 | 15 |
| MAPK8 | -0.191027 | 0.527496 | 0.7172482 | 0.8012676 | 20 |
| ASAH2 | -0.0492113 | 0.0666656 | 0.4604039 | 0.8643556 | 20 |
| DKK1 | -0.248473 | 0.204319 | 0.2239463 | 0.9856121 | 5 |
| MBL2 | -0.0141937 | 0.0338891 | 0.6753408 | 0.3143369 | 20 |
| SRGN | -0.0203503 | 0.188505 | 0.9140304 | 0.6523773 | 20 |
| VPS26A | -0.118386 | 0.166879 | 0.47807 | 0.7506898 | 20 |
| SAR1A | -0.0768833 | 0.195226 | 0.6937171 | 0.1806392 | 20 |
| PPA1 | -0.103697 | 0.163387 | 0.5256444 | 0.3269256 | 20 |
| PCBD1 | 0.0437705 | 0.248991 | 0.8604576 | 0.7302223 | 20 |
| UNC5B | -0.728195 | 0.357388 | 0.04159514 | 0.559688 | 13 |
| C10orf54 | 0.0346494 | 0.115978 | 0.7651237 | 0.3186214 | 20 |
| C10orf54 | 0.0278142 | 0.0930968 | 0.7651187 | 0.3481318 | 20 |
| PSAP | 0.136379 | 0.104567 | 0.1921561 | 0.01064699 | 20 |
| SPOCK2 | 0.0869947 | 0.154217 | 0.5726823 | 0.105937 | 20 |
| DNAJB12 | 0.18742 | 0.344069 | 0.5859484 | 0.7336323 | 20 |
| PLA2G12B | 0.672537 | 0.40499 | 0.09678849 | 0.7775874 | 20 |
| ANXA7 | 1.2445 | 0.801062 | 0.12029 | 0.07442885 | 9 |
| ANXA7 | 1.23502 | 0.794816 | 0.1202206 | 0.07147817 | 9 |
| PLAU | -0.131939 | 0.107893 | 0.2213812 | 0.792719 | 13 |
| ADK | -0.265684 | 0.544394 | 0.6255232 | 0.8637445 | 20 |
| SFTPD | 0.0579134 | 0.0557561 | 0.2989483 | 0.6482788 | 20 |
| ANXA11 | -0.0713252 | 0.194043 | 0.7131909 | 0.1447426 | 20 |
| FAM213A | -0.40432 | 0.310562 | 0.1929513 | 2.88E-05 | 20 |
| MINPP1 | 0.634806 | 0.311614 | 0.04163483 | 0.1806355 | 20 |
| PTEN | 0.0224303 | 0.868841 | 0.9794038 | 0.0854979 | 11 |
| LIPN | -0.022018 | 0.0411197 | 0.5923309 | 0.4620702 | 20 |
| STAMBPL1 | -0.345385 | 0.272904 | 0.2056602 | 0.663648 | 20 |
| FAS | 0.0075802 | 0.125657 | 0.9518971 | 0.2627501 | 20 |
| FAS | 0.00721312 | 0.119572 | 0.9518971 | 0.2578873 | 20 |
| IFIT3 | 0.65668 | 0.388962 | 0.09135545 | 0.8914533 | 20 |
| FGFBP3 | -0.12038 | 0.109398 | 0.2711642 | 0.4728155 | 20 |
| RBP4 | -0.213414 | 0.356007 | 0.548862 | 0.3676798 | 20 |
| PDLIM1 | -0.212146 | 0.422304 | 0.6154183 | 0.1097573 | 3 |
| ENTPD1 | 0.0429704 | 0.213074 | 0.8401756 | 0.7479898 | 20 |
| CRTAC1 | 0.0840499 | 0.0823058 | 0.3071639 | 0.4259646 | 19 |
| ARL3 | -0.116268 | 0.239566 | 0.6274434 | 0.1756758 | 20 |
| AS3MT | 0.783754 | 0.657218 | 0.2330524 | 0.4538832 | 20 |
| NT5C2 | -0.396465 | 0.265024 | 0.1346645 | 0.4651816 | 20 |
| GSTO1 | -0.0461747 | 0.0387753 | 0.2337212 | 0.281874 | 20 |
| SORCS1 | -0.0330078 | 0.455829 | 0.9422734 | 0.8189673 | 20 |
| XPNPEP1 | 0.760968 | 0.489755 | 0.1202386 | 0.2466936 | 20 |
| VWA2 | -0.113892 | 0.179063 | 0.5247457 | 0.7814364 | 20 |
| GFRA1 | 0.170165 | 0.160443 | 0.2888744 | 0.5017688 | 20 |
| PNLIP | -0.00861952 | 0.275951 | 0.9750816 | 0.07166874 | 4 |
| PNLIPRP1 | -0.104192 | 0.196008 | 0.5950248 | 0.5946265 | 20 |
| PLEKHA1 | 0.0462557 | 0.103652 | 0.6554108 | 0.2616122 | 20 |
| HTRA1 | -0.499193 | 0.296915 | 0.09271169 | 0.6313839 | 20 |
| ACADSB | 0.0459885 | 0.289608 | 0.8738297 | 0.4436917 | 20 |
| CHST15 | -0.0347225 | 0.304384 | 0.9091786 | 0.02024775 | 20 |
| UROS | -0.0879723 | 0.170065 | 0.6049565 | 0.5006413 | 20 |
| GLRX3 | 0.030447 | 0.374582 | 0.9352172 | 0.5480522 | 20 |
| ECHS1 | 0.350222 | 0.644291 | 0.5867322 | 0.4743436 | 20 |
| CDHR5 | -0.871128 | 0.556134 | 0.1172548 | 0.4141264 | 20 |
| TALDO1 | -0.686287 | 0.678955 | 0.3121124 | 0.7145944 | 10 |
| AP2A2 | 0.398835 | 0.483255 | 0.4091956 | 0.3233133 | 16 |
| BRSK2 | -0.0167378 | 0.171026 | 0.9220381 | 0.3987599 | 20 |
| CTSD | 0.145806 | 0.189084 | 0.4406393 | 0.3737684 | 20 |
| NAP1L4 | 0.150758 | 0.188988 | 0.4250375 | 0.4023736 | 20 |
| STIM1 | 0.0415251 | 0.308256 | 0.8928412 | 0.84328 | 20 |
| RRM1 | 0.208024 | 0.520189 | 0.6892303 | 0.5975892 | 20 |
| SMPD1 | 0.134228 | 0.096754 | 0.165348 | 0.6966833 | 20 |
| TRIM3 | -0.0980568 | 0.315801 | 0.7561791 | 0.9962153 | 20 |
| TPP1 | -0.216455 | 0.220857 | 0.3270524 | 0.4071388 | 20 |
| CYB5R2 | -0.0478527 | 0.138706 | 0.7300992 | 0.654033 | 20 |
| SWAP70 | -0.0336238 | 0.0762289 | 0.659148 | 0.9994652 | 20 |
| LYVE1 | -0.0512424 | 0.132398 | 0.6987315 | 0.3022237 | 20 |
| MRVI1 | -0.0559283 | 0.27599 | 0.8394115 | 0.4731283 | 20 |
| DKK3 | 0.159416 | 0.0900505 | 0.07667854 | 0.2378131 | 20 |
| PARVA | -0.0601192 | 0.488049 | 0.9019626 | 0.7632139 | 20 |
| CALCB | -0.00167291 | 0.145841 | 0.9908478 | 0.1792602 | 20 |
| PLEKHA7 | 0.0590339 | 0.0808649 | 0.465371 | 0.2191939 | 20 |
| SAA4 | 0.0100452 | 0.0532497 | 0.8503724 | 0.9939948 | 18 |
| SAA2 | -0.00945107 | 0.0814971 | 0.9076777 | 0.7061718 | 20 |
| SAA1 | -0.00755427 | 0.0651408 | 0.9076776 | 0.7165464 | 20 |
| LDHA | -0.00110526 | 0.540716 | 0.9983691 | 0.9514363 | 11 |
| LDHC | -0.155319 | 0.657049 | 0.8131309 | 0.7792588 | 12 |
| TSG101 | -0.347398 | 0.196233 | 0.07667133 | 0.5874177 | 20 |
| HTATIP2 | -0.00233296 | 0.0521531 | 0.9643202 | 0.6486308 | 20 |
| HTATIP2 | -0.0123708 | 0.27655 | 0.9643204 | 0.5798391 | 20 |
| NELL1 | 0.103376 | 0.061969 | 0.09527747 | 0.2058304 | 20 |
| LGR4 | 0.0173429 | 0.288449 | 0.9520565 | 0.6969503 | 20 |
| BDNF | -0.237085 | 0.299109 | 0.4279891 | 0.9013316 | 10 |
| BDNF | -0.29773 | 0.376234 | 0.4287436 | 0.830297 | 8 |
| MPPED2 | -0.0590932 | 0.261023 | 0.8208974 | 0.1363804 | 20 |
| RCN1 | -0.0269013 | 0.217362 | 0.9015035 | 0.8757348 | 20 |
| KIAA1549L | 0.0525771 | 0.0886126 | 0.5529557 | 0.9041542 | 20 |
| CD59 | -0.200771 | 0.160868 | 0.2120139 | 0.1708632 | 20 |
| CAT | -0.128641 | 0.166233 | 0.4390143 | 0.07381223 | 20 |
| PDHX | -0.556335 | 0.444839 | 0.2110648 | 0.6372915 | 5 |
| FJX1 | -0.0861354 | 0.163549 | 0.5984274 | 0.4827052 | 20 |
| LRRC4C | -0.13133 | 0.167014 | 0.4316654 | 0.3892777 | 20 |
| ALKBH3 | 0.013537 | 0.128311 | 0.9159777 | 0.3421783 | 20 |
| MDK | -0.00348076 | 0.467039 | 0.9940536 | 0.5459287 | 7 |
| F2 | 0.151852 | 0.255432 | 0.5521829 | 0.02085436 | 20 |
| F2 | 0.175936 | 0.296 | 0.5522587 | 0.04404702 | 20 |
| LRP4 | 0.0399272 | 0.0697822 | 0.5672072 | 0.1974993 | 20 |
| FOLH1 | -0.0771883 | 0.204543 | 0.7058994 | 0.4589604 | 6 |
| PRG3 | -0.155478 | 0.392842 | 0.6922691 | 0.2134314 | 20 |
| SERPING1 | -0.0430703 | 0.0453912 | 0.3426876 | 0.5128179 | 20 |
| GIF | 0.152427 | 0.154388 | 0.3234957 | 0.5755719 | 20 |
| TCN1 | 0.0737244 | 0.0946503 | 0.4360308 | 0.2282042 | 20 |
| TMEM132A | 0.0177837 | 0.0576563 | 0.7577453 | 0.9712145 | 20 |
| ASRGL1 | -0.0470052 | 0.146271 | 0.7479393 | 0.5306081 | 20 |
| B3GAT3 | 0.0763962 | 0.133508 | 0.5671721 | 0.7525911 | 20 |
| HRASLS2 | 0.0170377 | 0.589656 | 0.9769488 | 0.2960165 | 13 |
| FKBP2 | -0.296504 | 0.650616 | 0.6485861 | 0.6758557 | 19 |
| EIF1AD | 0.00793684 | 0.151681 | 0.9582691 | 0.2841132 | 20 |
| CST6 | -0.0285352 | 0.176324 | 0.8714362 | 0.1448178 | 20 |
| CD248 | -0.655275 | 0.572609 | 0.2524712 | 0.8940159 | 16 |
| CTSF | -0.109602 | 0.174637 | 0.5302656 | 0.06810107 | 20 |
| CCS | -0.150195 | 0.253884 | 0.5541248 | 0.4756445 | 20 |
| GSTP1 | 0.230485 | 0.226229 | 0.3082926 | 0.7183129 | 20 |
| IL18BP | 0.501695 | 0.439028 | 0.2531464 | 0.9881422 | 10 |
| FOLR3 | 0.0499064 | 0.0331154 | 0.1317996 | 0.5259063 | 20 |
| FOLR2 | -0.0822494 | 0.302904 | 0.7859789 | 0.4601801 | 20 |
| RELT | -0.968575 | 0.542397 | 0.07414299 | 0.9297552 | 20 |
| CHRDL2 | -0.0209905 | 0.114237 | 0.8542127 | 0.2733557 | 20 |
| ARRB1 | 0.265287 | 0.477631 | 0.578606 | 0.3322486 | 20 |
| SERPINH1 | 0.291283 | 0.233708 | 0.2126346 | 0.797455 | 20 |
| LRRC32 | 0.390719 | 0.476131 | 0.4118679 | 0.9540252 | 13 |
| PRCP | -0.0802057 | 0.183055 | 0.6612765 | 0.05892586 | 20 |
| MRE11A | 1.85403 | 0.940707 | 0.04873656 | 0.9189248 | 6 |
| CNTN5 | -0.125539 | 0.110343 | 0.2552384 | 0.9054382 | 20 |
| MMP7 | -0.0849979 | 0.101433 | 0.4020451 | 0.3937506 | 20 |
| MMP8 | 0.281283 | 0.152867 | 0.06576143 | 0.6341801 | 20 |
| MMP10 | -0.00317516 | 0.105082 | 0.9758948 | 0.8468818 | 20 |
| MMP1 | 0.00418426 | 0.0685935 | 0.9513585 | 0.6696008 | 20 |
| MMP13 | -0.447563 | 0.493001 | 0.3639663 | 0.6744351 | 19 |
| PDGFD | 0.0198074 | 0.0854447 | 0.8166812 | 0.2307777 | 20 |
| PDGFD | 0.0230011 | 0.0992227 | 0.8166834 | 0.1084725 | 20 |
| AASDHPPT | 0.060477 | 0.870309 | 0.9446003 | 0.4060946 | 10 |
| ACAT1 | 0.36095 | 0.333916 | 0.2797149 | 0.9933377 | 20 |
| KDELC2 | -0.146753 | 0.15341 | 0.3387658 | 0.7790453 | 20 |
| LAYN | -0.41399 | 0.590628 | 0.4833451 | 0.4531135 | 20 |
| NCAM1 | 0.0397046 | 0.130097 | 0.7602198 | 0.1677163 | 17 |
| NNMT | 0.264374 | 0.681771 | 0.6981826 | 0.7924217 | 15 |
| REXO2 | -0.262711 | 0.298474 | 0.3787629 | 0.9871418 | 20 |
| CADM1 | -0.125718 | 0.213027 | 0.5550908 | 0.5535197 | 20 |
| APOA5 | 0.0532276 | 0.046223 | 0.2495103 | 0.09181202 | 20 |
| APOA4 | -0.00737301 | 0.327596 | 0.982044 | 0.6789172 | 5 |
| APOC3 | 0.141601 | 0.191871 | 0.4605144 | 0.01477758 | 20 |
| APOA1 | 0.139 | 0.106347 | 0.1911994 | 0.4778877 | 5 |
| TAGLN | 0.153929 | 0.320277 | 0.6307928 | 0.7098254 | 14 |
| PCSK7 | -0.0138344 | 0.0385447 | 0.719656 | 0.2423029 | 20 |
| CBL | 0.0203062 | 0.33889 | 0.9522196 | 0.7162758 | 20 |
| C1QTNF5 | 0.0696334 | 0.130436 | 0.5934454 | 0.2564073 | 20 |
| OAF | -0.0273435 | 0.0691251 | 0.6924252 | 0.3438003 | 20 |
| UBASH3B | 0.120624 | 0.259959 | 0.6426391 | 0.47748 | 20 |
| CRTAM | -0.101502 | 0.126058 | 0.420705 | 0.1184167 | 20 |
| CLMP | 0.0442061 | 0.143105 | 0.7573923 | 0.258229 | 20 |
| SIAE | -0.100756 | 0.292804 | 0.7307663 | 0.9020586 | 16 |
| ESAM | 0.144946 | 0.155035 | 0.349828 | 0.6961518 | 20 |
| PATE4 | -0.035332 | 0.240238 | 0.8830762 | 0.3109386 | 20 |
| CDON | 0.052015 | 0.0651727 | 0.4248064 | 0.6809468 | 20 |
| TIRAP | -0.184247 | 0.136571 | 0.1773075 | 0.8293424 | 20 |
| APLP2 | 0.208519 | 0.279818 | 0.456153 | 0.4874472 | 20 |
| NTM | -0.0308454 | 0.0814671 | 0.7049671 | 0.8461677 | 20 |
| NTM | -0.0267989 | 0.0707793 | 0.7049655 | 0.6131999 | 20 |
| OPCML | 0.0631226 | 0.284542 | 0.8244392 | 0.2273362 | 20 |
| THYN1 | -0.0244529 | 0.611675 | 0.9681114 | 0.0787269 | 20 |
| FKBP4 | 0.905895 | 0.482017 | 0.0601919 | 0.0406096 | 13 |
| FGF23 | 0.522622 | 0.584212 | 0.3710141 | 0.1539942 | 14 |
| NTF3 | -0.296572 | 0.405102 | 0.4641118 | 0.2192503 | 20 |
| VWF | 0.174096 | 0.272481 | 0.522868 | 0.1826148 | 20 |
| TAPBPL | -0.0153219 | 0.0237939 | 0.5196124 | 0.003221925 | 20 |
| GAPDH | 1.50761 | 0.615116 | 0.01424861 | 0.589073 | 20 |
| PIANP | 0.188186 | 0.174402 | 0.2805708 | 0.07511331 | 20 |
| PIANP | 0.17097 | 0.158411 | 0.2804632 | 0.04499191 | 20 |
| LAG3 | 0.11338 | 0.224292 | 0.6132058 | 0.09155609 | 10 |
| CD4 | 0.405942 | 0.589253 | 0.49088 | 0.4710532 | 7 |
| TPI1 | 0.518273 | 0.625083 | 0.4070326 | 0.2349536 | 4 |
| ENO2 | -0.256131 | 0.222472 | 0.2496089 | 0.4281633 | 5 |
| C1S | -0.205287 | 0.37262 | 0.581683 | 0.6167025 | 10 |
| C1RL | -0.241121 | 0.284181 | 0.3961729 | 0.1327648 | 4 |
| CLEC4C | -0.312622 | 0.410545 | 0.4463695 | 0.6583349 | 10 |
| PZP | -0.0982432 | 0.278817 | 0.7245703 | 0.7625162 | 20 |
| KLRB1 | -1.77468 | 0.95015 | 0.06179098 | 0.4071624 | 3 |
| CLEC12A | 0.0227975 | 0.02353 | 0.3326101 | 0.4736591 | 20 |
| CLEC1B | -0.427541 | 0.192844 | 0.02662093 | 0.6957398 | 20 |
| OLR1 | -0.238446 | 0.275323 | 0.386458 | 0.9444659 | 20 |
| MANSC1 | -0.0142063 | 0.121912 | 0.9072337 | 0.1803797 | 20 |
| CDKN1B | 0.399704 | 0.378227 | 0.2906096 | 0.6149837 | 19 |
| HEBP1 | 0.0452045 | 0.0699932 | 0.5183819 | 0.577988 | 7 |
| KIAA1467 | -0.0409474 | 0.121911 | 0.736961 | 0.09701008 | 15 |
| ART4 | 0.00287281 | 0.0349711 | 0.9345289 | 0.05816668 | 20 |
| MGP | -0.116654 | 0.128779 | 0.3650153 | 0.5035279 | 20 |
| RECQL | 0.0178082 | 0.346943 | 0.9590635 | 0.2179616 | 20 |
| LDHB | -0.103901 | 0.392407 | 0.7911809 | 0.3947084 | 6 |
| MANSC4 | 0.0276493 | 0.0615158 | 0.6530953 | 0.03023415 | 20 |
| PTHLH | -0.176051 | 0.27368 | 0.5200483 | 0.8419928 | 20 |
| PKP2 | -0.176609 | 0.255532 | 0.4894765 | 0.776455 | 20 |
| CNTN1 | 0.089187 | 0.142993 | 0.532813 | 0.7479859 | 20 |
| GXYLT1 | 0.00891168 | 0.0609096 | 0.8836768 | 0.5153214 | 7 |
| IRAK4 | 0.235337 | 0.456394 | 0.6061027 | 0.4443425 | 20 |
| AMIGO2 | -0.178874 | 0.298869 | 0.5495046 | 0.1606652 | 18 |
| ENDOU | 0.120403 | 0.497706 | 0.8088459 | 0.4666438 | 20 |
| COL2A1 | 0.031497 | 0.0407941 | 0.4400567 | 0.7096475 | 20 |
| PFKM | 0.129887 | 0.191775 | 0.4982222 | 0.8531298 | 20 |
| GPD1 | 0.45034 | 0.713803 | 0.5281043 | 0.9552325 | 3 |
| LIMA1 | 0.815834 | 0.499173 | 0.1021813 | 0.4487794 | 20 |
| ACVRL1 | 0.166117 | 0.145423 | 0.2533282 | 0.4466544 | 20 |
| KRT5 | -0.108184 | 0.214011 | 0.6132029 | 0.06203148 | 20 |
| KRT1 | 0.753003 | 0.689597 | 0.2748569 | 0.2818316 | 20 |
| IGFBP6 | 0.190028 | 0.380373 | 0.6173686 | 0.6167293 | 20 |
| PPP1R1A | 0.621495 | 0.494183 | 0.2085288 | 0.2161559 | 19 |
| LACRT | 0.528058 | 0.539036 | 0.3272666 | 0.4915478 | 20 |
| MMP19 | -0.0286349 | 0.277141 | 0.917707 | 0.8994902 | 20 |
| PMEL | -0.213814 | 0.129904 | 0.09977602 | 0.1421316 | 20 |
| ERBB3 | -0.444941 | 0.275135 | 0.1058414 | 0.3527909 | 20 |
| APOF | -0.0372675 | 0.200576 | 0.8526 | 0.7282683 | 20 |
| STAT6 | -0.143081 | 0.323651 | 0.6584294 | 0.8116144 | 5 |
| INHBC | 0.0237076 | 0.0268013 | 0.3763896 | 0.2409131 | 20 |
| INHBC | -0.10611 | 0.282343 | 0.707051 | 0.4296544 | 20 |
| ARHGEF25 | 0.0850769 | 0.257365 | 0.7409704 | 0.8813968 | 18 |
| USP15 | -0.540435 | 0.290044 | 0.06242154 | 0.4817425 | 20 |
| WIF1 | -0.808279 | 0.510501 | 0.1133518 | 0.888086 | 20 |
| IL22 | 0.445037 | 0.64256 | 0.4885612 | 0.9233587 | 11 |
| CPM | -0.203904 | 0.332809 | 0.5400897 | 0.3882916 | 20 |
| LYZ | 0.14415 | 0.0591417 | 0.01479446 | 0.6083573 | 20 |
| YEATS4 | 1.46089 | 0.667901 | 0.02872176 | 0.447493 | 19 |
| LGR5 | 0.0056511 | 0.553114 | 0.9918482 | 0.7295705 | 17 |
| RAB21 | -0.000117914 | 0.587147 | 0.9998398 | 0.233171 | 3 |
| GLIPR1 | -0.603686 | 0.351248 | 0.08567106 | 0.6242868 | 20 |
| PAWR | 1.24503 | 0.693288 | 0.07252002 | 0.8170945 | 7 |
| MGAT4C | -0.332 | 0.616318 | 0.5901055 | 0.7418542 | 20 |
| UBE2N | -0.839181 | 0.61235 | 0.1705534 | 0.9337869 | 14 |
| CRADD | 0.228223 | 0.553141 | 0.6799038 | 0.9596052 | 15 |
| PLXNC1 | -0.0102514 | 0.0394579 | 0.7950132 | 0.1911012 | 3 |
| NTN4 | -0.139746 | 0.149365 | 0.3494788 | 0.109466 | 20 |
| MYBPC1 | -0.724933 | 0.368987 | 0.04945404 | 0.987682 | 20 |
| NT5DC3 | -0.0536088 | 0.170256 | 0.7528586 | 0.7814498 | 20 |
| HSP90B1 | -0.00745419 | 0.0438697 | 0.8650759 | 0.618928 | 20 |
| CHST11 | 0.131437 | 0.103901 | 0.2058619 | 0.3408784 | 20 |
| SELPLG | -0.0276348 | 0.119229 | 0.8167089 | 0.545077 | 20 |
| MMAB | -0.00462576 | 0.102862 | 0.964131 | 0.7793715 | 20 |
| ALDH2 | 0.222946 | 0.380437 | 0.5578571 | 0.6348987 | 20 |
| SDSL | -0.0975801 | 0.0976857 | 0.317834 | 0.69716 | 13 |
| TESC | -0.0136705 | 0.0591368 | 0.8171843 | 0.4242836 | 20 |
| PEBP1 | -0.0122052 | 0.133363 | 0.9270806 | 0.5274347 | 16 |
| PSMD9 | 0.206424 | 0.244078 | 0.397705 | 0.8909304 | 20 |
| EIF2B1 | -0.0617316 | 0.760408 | 0.9352971 | 0.6019426 | 20 |
| TMEM132B | 0.128992 | 0.188944 | 0.4947976 | 0.9748353 | 20 |
| TMEM132C | -0.12622 | 0.117179 | 0.2814103 | 0.4892548 | 20 |
| TMEM132D | -0.0849559 | 0.0714979 | 0.2347433 | 0.8984086 | 20 |
| TNFRSF19 | -0.297576 | 0.291442 | 0.307232 | 0.939867 | 20 |
| C1QTNF9 | -0.1746 | 0.154351 | 0.2579737 | 0.7116603 | 20 |
| FLT1 | -0.359875 | 0.580284 | 0.5351449 | 0.3551124 | 20 |
| B3GALTL | -0.177538 | 0.115297 | 0.1236014 | 0.4170955 | 20 |
| KL | -0.0517686 | 0.172558 | 0.7641724 | 0.8453896 | 20 |
| DCLK1 | 0.181454 | 0.157873 | 0.2504048 | 0.9800313 | 20 |
| SPG20 | 0.951373 | 0.60364 | 0.1150122 | 0.363536 | 9 |
| POSTN | -0.0499851 | 0.276878 | 0.8567354 | 0.6162503 | 13 |
| CPB2 | 0.0826905 | 0.0592438 | 0.1627849 | 0.5185294 | 20 |
| LCP1 | 0.8607 | 0.274463 | 0.001713024 | 0.02907078 | 20 |
| ESD | 0.013625 | 0.164617 | 0.9340358 | 0.4545943 | 20 |
| THSD1 | -0.0863409 | 0.0834529 | 0.3008532 | 0.8033725 | 20 |
| PCDH9 | -0.098029 | 0.137248 | 0.4750756 | 0.4496821 | 20 |
| CLN5 | -0.326409 | 0.279548 | 0.2429554 | 0.2167562 | 20 |
| SLITRK1 | -0.0486987 | 0.519138 | 0.9252627 | 0.8433905 | 20 |
| SLITRK6 | -0.083152 | 0.158019 | 0.5987386 | 0.9235794 | 20 |
| SLITRK5 | 0.425119 | 0.241477 | 0.07832409 | 0.6209293 | 15 |
| GPC5 | -0.0781733 | 0.0561907 | 0.1641606 | 0.001570253 | 20 |
| GPC6 | 1.16062 | 0.692272 | 0.09363139 | 0.5467679 | 19 |
| HS6ST3 | -0.0836564 | 0.307024 | 0.7852563 | 0.4008915 | 20 |
| EFNB2 | -0.641471 | 0.497427 | 0.197197 | 0.8363554 | 8 |
| TNFSF13B | -0.0848653 | 0.482799 | 0.8604687 | 0.9289665 | 10 |
| MCF2L | -0.00678564 | 0.136856 | 0.9604553 | 0.4517445 | 20 |
| F7 | 0.0363689 | 0.0428003 | 0.3954728 | 0.007915338 | 20 |
| F10 | -0.0462336 | 0.215075 | 0.8297943 | 0.3239781 | 20 |
| F10 | -0.0473561 | 0.220297 | 0.8297947 | 0.2626505 | 20 |
| GAS6 | 0.0167439 | 0.115205 | 0.8844421 | 0.2339763 | 20 |
| PNP | -0.116174 | 0.221864 | 0.6005376 | 0.3772642 | 20 |
| ANG | -0.0848335 | 0.0726196 | 0.2427301 | 0.4022308 | 20 |
| RNASE6 | 0.0114777 | 0.0328852 | 0.7270722 | 0.09424381 | 20 |
| RNASE1 | -0.100298 | 0.143774 | 0.4854197 | 0.2095665 | 10 |
| RNASE3 | -0.150189 | 0.109067 | 0.1684995 | 0.172939 | 20 |
| RNASE2 | 0.12502 | 0.199193 | 0.5302442 | 0.8571887 | 20 |
| LRP10 | 0.782719 | 0.497474 | 0.1156293 | 0.8427833 | 16 |
| AP1G2 | 0.180048 | 0.198198 | 0.3636527 | 0.9987938 | 15 |
| PSME1 | 0.174052 | 0.532087 | 0.7435827 | 0.8670134 | 15 |
| PSME2 | -0.31999 | 0.451353 | 0.4783507 | 0.4865096 | 6 |
| GMPR2 | -0.186211 | 0.135716 | 0.1700449 | 0.3849248 | 20 |
| GZMB | 1.07499 | 0.376914 | 0.004343516 | 0.4661308 | 20 |
| COCH | 0.0192485 | 0.106918 | 0.8571284 | 0.9894795 | 20 |
| DTD2 | -0.196117 | 0.300418 | 0.5138765 | 0.6955207 | 20 |
| CFL2 | 0.172495 | 0.280351 | 0.5383693 | 0.8932596 | 20 |
| FAM177A1 | -0.0595518 | 0.0547771 | 0.2769637 | 0.5159456 | 20 |
| MDGA2 | 0.0300678 | 0.080636 | 0.7092361 | 0.1653452 | 20 |
| MGAT2 | -0.108144 | 0.220349 | 0.6235775 | 0.2801397 | 20 |
| PYGL | 0.380072 | 0.25168 | 0.1310083 | 0.6980106 | 20 |
| NID2 | 0.0225136 | 0.0583735 | 0.6997327 | 0.5190787 | 20 |
| GNPNAT1 | -0.358852 | 0.468285 | 0.4434916 | 0.4831678 | 20 |
| LGALS3 | 0.169146 | 0.093971 | 0.07186454 | 0.3975917 | 20 |
| MTHFD1 | 0.266803 | 0.303443 | 0.379265 | 0.2410085 | 20 |
| MAX | -0.0121344 | 0.162391 | 0.9404349 | 0.7210275 | 20 |
| FUT8 | 0.0338779 | 0.0346034 | 0.3275629 | 0.3526564 | 20 |
| VTI1B | 1.51907 | 1.76951 | 0.3906348 | 0.4919051 | 7 |
| GALNT16 | -0.0885579 | 0.171734 | 0.6060862 | 0.6429983 | 20 |
| SMOC1 | -0.477961 | 0.216447 | 0.02722945 | 0.03182907 | 20 |
| ENTPD5 | 0.082075 | 0.0608686 | 0.177531 | 0.8017356 | 20 |
| ALDH6A1 | 0.183133 | 0.359357 | 0.6103231 | 0.8338782 | 20 |
| GSTZ1 | 0.0279672 | 0.0252431 | 0.2678995 | 0.8332414 | 20 |
| NRXN3 | 0.0682345 | 0.209211 | 0.7443096 | 0.7072406 | 20 |
| NRXN3 | 0.0687892 | 0.210913 | 0.7443108 | 0.7087333 | 20 |
| FLRT2 | -0.221069 | 0.124926 | 0.07679265 | 0.1024778 | 20 |
| FBLN5 | -0.102806 | 0.380876 | 0.7872209 | 0.3543397 | 20 |
| ATXN3 | 0.0833493 | 0.124543 | 0.5033407 | 0.00805654 | 20 |
| LGMN | 0.199872 | 0.232502 | 0.3899768 | 0.8588047 | 20 |
| CHGA | -0.151073 | 0.121215 | 0.2126445 | 0.3901394 | 20 |
| SERPINA10 | 0.0811939 | 0.0449644 | 0.07095914 | 0.03276907 | 20 |
| SERPINA1 | 0.100381 | 0.0781664 | 0.1990741 | 0.3969761 | 20 |
| SERPINA11 | -0.106753 | 0.126515 | 0.398784 | 0.4339322 | 11 |
| SERPINA9 | 0.390914 | 0.200714 | 0.05146068 | 0.04435803 | 20 |
| SERPINA12 | -0.132688 | 0.0639576 | 0.03802117 | 0.81322 | 20 |
| SERPINA4 | 0.0743675 | 0.0714947 | 0.2982559 | 0.4772199 | 20 |
| SERPINA5 | -0.0479346 | 0.130597 | 0.713587 | 0.08295282 | 20 |
| SERPINA3 | 0.12519 | 0.168907 | 0.4585867 | 0.458631 | 20 |
| TCL1A | 0.0983879 | 0.14754 | 0.5048658 | 0.5084085 | 20 |
| EVL | -0.159545 | 0.203659 | 0.4333958 | 0.175707 | 19 |
| WARS | 0.308447 | 0.136105 | 0.02343649 | 0.8657798 | 20 |
| DLK1 | 0.0150374 | 0.0632267 | 0.81201 | 0.5130546 | 20 |
| DLK1 | 0.0153216 | 0.0644215 | 0.81201 | 0.5134433 | 20 |
| CINP | 0.525213 | 0.650448 | 0.4193994 | 0.816629 | 20 |
| MARK3 | 0.785475 | 0.574639 | 0.1716564 | 0.4530715 | 20 |
| KLC1 | 0.281582 | 0.327587 | 0.3900287 | 0.03924272 | 20 |
| ADSSL1 | 0.296044 | 0.116951 | 0.01136235 | 0.02918663 | 20 |
| GREM1 | -0.0144846 | 0.0397443 | 0.715527 | 0.5445204 | 20 |
| THBS1 | 0.00563072 | 0.674201 | 0.9933364 | 0.8815232 | 20 |
| IVD | -0.319603 | 0.259763 | 0.2185612 | 0.5026269 | 20 |
| DNAJC17 | -0.289108 | 0.288873 | 0.3169171 | 0.6404266 | 20 |
| SPINT1 | -0.731036 | 0.213543 | 0.000618502 | 0.04362209 | 20 |
| DLL4 | 0.650077 | 0.510379 | 0.2027647 | 0.9709887 | 20 |
| ITPKA | 0.0951 | 0.411914 | 0.8174131 | 0.7756865 | 20 |
| TYRO3 | 0.00732238 | 0.287801 | 0.979702 | 0.2824181 | 20 |
| CKMT1A | 0.152924 | 0.344046 | 0.6566911 | 0.2124619 | 20 |
| CASC4 | -1.22611 | 1.84575 | 0.5065065 | 0.2263738 | 9 |
| SORD | -0.251483 | 0.141887 | 0.07632432 | 0.07627859 | 20 |
| GATM | -0.16191 | 0.170288 | 0.3417041 | 0.2985313 | 20 |
| DUT | 0.560312 | 0.501649 | 0.2640202 | 0.518414 | 20 |
| USP8 | -0.902474 | 0.500883 | 0.07158228 | 0.01158387 | 20 |
| SCG3 | -0.00612819 | 0.086508 | 0.9435254 | 0.04037677 | 20 |
| ANXA2 | 0.0618309 | 0.0541688 | 0.2536831 | 0.7360881 | 20 |
| ANXA2 | 0.0758157 | 0.0664244 | 0.2537106 | 0.529655 | 20 |
| CA12 | 0.111911 | 0.634999 | 0.8601064 | 0.4077375 | 20 |
| DAPK2 | 0.110574 | 0.112534 | 0.3258095 | 0.07170477 | 20 |
| SNX1 | 0.196587 | 0.203726 | 0.334567 | 0.02474494 | 20 |
| IGDCC4 | 0.0185797 | 0.0985517 | 0.8504632 | 0.07612422 | 15 |
| MAP2K1 | -0.282989 | 0.468176 | 0.5455439 | 0.9026564 | 20 |
| GLCE | -0.0735317 | 0.0546473 | 0.1784414 | 0.6505721 | 20 |
| PKM2 | 0.0746569 | 0.635215 | 0.9064399 | 0.831009 | 12 |
| NEO1 | 0.147162 | 0.175784 | 0.4024961 | 0.6787669 | 20 |
| ISLR2 | -0.109382 | 0.148323 | 0.4608461 | 0.9713533 | 20 |
| SEMA7A | 0.446061 | 0.4301 | 0.2996838 | 0.5054938 | 10 |
| CSK | 0.0514503 | 0.447279 | 0.9084218 | 0.07743301 | 20 |
| ULK3 | -0.119575 | 0.350322 | 0.7328554 | 0.115402 | 20 |
| PTPN9 | -0.0553677 | 0.445964 | 0.9011944 | 0.1607458 | 20 |
| SNUPN | 0.151236 | 0.240462 | 0.5293873 | 0.5042203 | 20 |
| ETFA | -0.689958 | 0.509698 | 0.1758441 | 0.4738219 | 20 |
| LINGO1 | 0.250836 | 0.349111 | 0.4724506 | 0.7223529 | 20 |
| DNAJA4 | 0.458035 | 0.42542 | 0.2816301 | 0.7965015 | 20 |
| CTSH | -0.0126178 | 0.0292755 | 0.6664674 | 0.65472 | 20 |
| MTHFS | -0.116883 | 0.0884217 | 0.1862066 | 0.376856 | 20 |
| FAH | 0.0154315 | 0.0615237 | 0.8019519 | 0.8921357 | 17 |
| MESDC2 | 0.295793 | 0.618796 | 0.632641 | 0.347832 | 20 |
| IL16 | -0.0593862 | 0.0642242 | 0.3551377 | 0.4108292 | 20 |
| STARD5 | 0.202828 | 0.161551 | 0.209296 | 0.3540109 | 20 |
| HOMER2 | -0.264194 | 0.44702 | 0.5545129 | 0.2388943 | 20 |
| NMB | 0.0591531 | 0.223255 | 0.7910422 | 0.1277682 | 20 |
| NTRK3 | 0.244397 | 0.191544 | 0.2019797 | 0.5179625 | 20 |
| ACAN | -0.407353 | 0.304152 | 0.1804705 | 0.6779974 | 19 |
| MFGE8 | 0.180101 | 0.12934 | 0.1637823 | 0.3052041 | 20 |
| RGMA | 0.126689 | 0.164417 | 0.4409802 | 0.4674765 | 20 |
| IGF1R | 0.167623 | 0.280353 | 0.5499081 | 0.9702859 | 20 |
| MPG | -0.0442256 | 0.306434 | 0.885245 | 0.7443875 | 16 |
| HBZ | -0.0364614 | 0.040801 | 0.3715141 | 0.5114668 | 20 |
| HBQ1 | -0.762856 | 0.394338 | 0.05304897 | 0.471672 | 20 |
| NME4 | 0.147681 | 0.190074 | 0.4371807 | 0.1887613 | 20 |
| WFIKKN1 | 0.385694 | 0.215044 | 0.07288386 | 0.2911073 | 20 |
| STUB1 | 0.925899 | 1.29293 | 0.4739147 | 0.9564897 | 3 |
| TPSB2 | 0.023267 | 0.0279283 | 0.4047902 | 0.2264742 | 20 |
| TPSAB1 | 0.0272413 | 0.055406 | 0.622955 | 0.1254021 | 20 |
| GNPTG | -0.0140213 | 0.111673 | 0.9000825 | 0.04952383 | 20 |
| IGFALS | -0.10157 | 0.29396 | 0.7297016 | 0.258484 | 14 |
| HAGH | -0.135379 | 0.18736 | 0.46995 | 0.9224168 | 20 |
| FAHD1 | -0.0839473 | 0.152659 | 0.5823863 | 0.4841612 | 20 |
| GFER | 0.339546 | 0.508329 | 0.5041556 | 0.7036832 | 20 |
| NPW | 0.0600275 | 0.0650893 | 0.3564071 | 0.1281094 | 20 |
| PGP | 0.071165 | 0.128305 | 0.5791315 | 0.9318564 | 20 |
| DCI | -0.061907 | 0.186434 | 0.7398455 | 0.2591067 | 20 |
| PRSS27 | 0.164074 | 0.147127 | 0.2647731 | 0.9031352 | 20 |
| ZG16B | 0.144429 | 0.161733 | 0.3718517 | 0.651198 | 20 |
| PRSS22 | 0.0299402 | 0.133069 | 0.8219811 | 0.2817902 | 20 |
| SRL | -0.252325 | 0.245196 | 0.3034447 | 0.2657603 | 20 |
| VASN | -0.0922377 | 0.394504 | 0.815135 | 0.1415207 | 20 |
| NMRAL1 | -0.0588758 | 0.0576779 | 0.3073638 | 0.7935539 | 20 |
| NAGPA | -0.216594 | 0.0844198 | 0.01029745 | 0.9137147 | 20 |
| PMM2 | 0.374645 | 0.21864 | 0.08661701 | 0.306271 | 20 |
| TNFRSF17 | -0.156473 | 0.222571 | 0.4820406 | 0.1514406 | 20 |
| NDE1 | 0.119724 | 0.420011 | 0.7756055 | 0.8436593 | 20 |
| PRKCB | 0.0893066 | 0.479172 | 0.8521493 | 0.7794872 | 20 |
| MAPK3 | 0.0157267 | 0.119943 | 0.8956818 | 0.8949579 | 20 |
| SULT1A3 | 0.0768549 | 0.234986 | 0.7436216 | 0.9748722 | 20 |
| DCTPP1 | -0.0786253 | 0.583547 | 0.89282 | 0.8387907 | 17 |
| PRSS8 | -0.355048 | 1.31146 | 0.7866012 | 0.5627706 | 20 |
| CBLN1 | -0.0480241 | 0.261161 | 0.8541022 | 0.7675085 | 3 |
| MMP2 | -0.0572801 | 0.303082 | 0.8500993 | 0.7236462 | 20 |
| CES1 | -1.24937 | 0.607287 | 0.03965732 | 0.6974999 | 3 |
| CCL22 | 0.279421 | 0.344068 | 0.4167296 | 0.2336224 | 7 |
| CX3CL1 | -0.257992 | 0.362052 | 0.4761044 | 0.5633383 | 20 |
| CCL17 | -0.108738 | 0.16404 | 0.5074116 | 0.7442412 | 20 |
| GPR56 | -0.568206 | 0.461913 | 0.2186546 | 0.347788 | 20 |
| CDH5 | -0.0394864 | 0.19851 | 0.8423303 | 0.7250891 | 20 |
| AGRP | -0.0373638 | 0.142746 | 0.7935135 | 0.01283968 | 20 |
| DPEP2 | -0.319149 | 0.262773 | 0.2245403 | 0.08217594 | 20 |
| NQO1 | 0.0998244 | 0.0423966 | 0.01854573 | 0.08132829 | 20 |
| CALB2 | 0.599096 | 0.607823 | 0.3243088 | 0.7143111 | 20 |
| CHST4 | -0.307968 | 0.372018 | 0.4077666 | 0.02885372 | 20 |
| HP | -0.0112387 | 0.0508554 | 0.8250977 | 0.5100609 | 20 |
| TXNL4B | 0.088529 | 0.224196 | 0.6929364 | 0.9241402 | 20 |
| CTRB2 | -0.0313479 | 0.0522878 | 0.5488218 | 0.4375175 | 20 |
| CTRB1 | 0.108338 | 0.08842 | 0.2204762 | 0.4923178 | 20 |
| GABARAPL2 | -0.195238 | 0.322848 | 0.545354 | 0.2564603 | 20 |
| WWOX | -0.276729 | 0.416908 | 0.5068399 | 0.9424684 | 20 |
| PLCG2 | -0.02576 | 0.230794 | 0.911129 | 0.3255047 | 20 |
| WFDC1 | -0.0731993 | 0.071801 | 0.3079775 | 0.06730697 | 20 |
| CRISPLD2 | 0.174036 | 0.362134 | 0.6308126 | 0.9412894 | 12 |
| MTHFSD | 0.0777528 | 0.0946268 | 0.4112599 | 0.1810972 | 20 |
| MVD | -0.321178 | 1.33532 | 0.8099234 | 0.9120517 | 13 |
| APRT | -0.00620481 | 0.370842 | 0.9866507 | 0.8390546 | 5 |
| DPEP1 | 0.00382146 | 0.0599557 | 0.9491788 | 0.3505435 | 20 |
| SCARF1 | 0.0191837 | 0.074782 | 0.7975432 | 0.9512611 | 20 |
| SERPINF1 | -0.112878 | 0.101114 | 0.264274 | 0.369916 | 20 |
| OVCA2 | 0.269758 | 0.60646 | 0.6564585 | 0.9399898 | 4 |
| TAX1BP3 | 0.0856083 | 0.3234 | 0.79123 | 0.2318949 | 20 |
| CYB5D2 | 0.0638658 | 0.103001 | 0.5352235 | 0.7892097 | 13 |
| CXCL16 | 0.0244612 | 0.182057 | 0.8931181 | 0.4664507 | 20 |
| GLTPD2 | -0.0721451 | 0.148945 | 0.6281206 | 0.6732736 | 20 |
| GP1BA | 0.000842823 | 0.184871 | 0.9963625 | 0.5791907 | 20 |
| ENO3 | -0.0270993 | 0.0911268 | 0.7661766 | 0.2794633 | 20 |
| ASGR1 | -0.217018 | 0.293072 | 0.4590012 | 0.249857 | 17 |
| ACADVL | 0.405203 | 0.613486 | 0.5089377 | 0.2803101 | 20 |
| NLGN2 | -0.204786 | 0.232158 | 0.377725 | 0.4611346 | 19 |
| TNFSF12 | -0.0475065 | 0.0898633 | 0.597046 | 0.9625 | 20 |
| CD68 | 0.188589 | 0.537781 | 0.7258286 | 0.7439306 | 3 |
| SHBG | 0.0142577 | 0.174547 | 0.934898 | 0.44807 | 20 |
| SAT2 | 0.0176394 | 0.141563 | 0.9008363 | 0.5779751 | 20 |
| ATP1B2 | 0.00180861 | 0.095109 | 0.9848282 | 0.6481001 | 20 |
| ALOX15B | 0.229795 | 0.423621 | 0.5875054 | 0.2647749 | 20 |
| NTN1 | 0.0859409 | 0.0807687 | 0.2873119 | 0.904779 | 20 |
| STX8 | 0.673131 | 0.516658 | 0.1926243 | 0.1040047 | 13 |
| HS3ST3B1 | -0.197935 | 0.630307 | 0.7534989 | 0.6410143 | 9 |
| NT5M | -0.100295 | 0.254344 | 0.6933386 | 0.9757913 | 20 |
| FLII | -0.510267 | 0.383413 | 0.1832363 | 0.6333929 | 8 |
| SHMT1 | -0.00104839 | 0.0451265 | 0.981465 | 0.6655049 | 20 |
| PRPSAP2 | -0.013397 | 0.494705 | 0.9783953 | 0.3517872 | 20 |
| GRAP | 0.228993 | 0.467429 | 0.6242048 | 0.7468426 | 20 |
| MFAP4 | -0.0774128 | 0.156635 | 0.6211475 | 0.3129856 | 5 |
| ALDH3A1 | 0.231563 | 0.238886 | 0.3323734 | 0.864381 | 20 |
| MAP2K3 | -0.398876 | 0.655287 | 0.5427205 | 0.7259867 | 15 |
| LGALS9 | -0.127918 | 0.247766 | 0.6056552 | 0.980177 | 20 |
| VTN | 0.0155762 | 0.0224182 | 0.4871799 | 0.9823745 | 20 |
| ALDOC | 0.0135315 | 0.222889 | 0.9515907 | 0.6390529 | 14 |
| SDF2 | 0.0850731 | 0.122476 | 0.4872982 | 0.9895327 | 20 |
| OMG | 0.745305 | 0.391144 | 0.05672195 | 0.9512003 | 20 |
| CCL7 | -0.0181739 | 0.0675317 | 0.7878402 | 0.6449428 | 20 |
| CCL11 | 0.182686 | 0.252888 | 0.4700495 | 0.8130696 | 20 |
| CCL8 | -0.00948259 | 0.0352358 | 0.7878389 | 0.679742 | 20 |
| RAD51L3 | 0.171578 | 0.571993 | 0.7642041 | 0.990464 | 15 |
| IGFBP4 | -0.537504 | 0.522897 | 0.3039806 | 0.9545193 | 12 |
| KRT20 | -0.12243 | 0.509707 | 0.8101782 | 0.3513416 | 11 |
| NT5C3L | -0.0722603 | 0.048571 | 0.1368236 | 0.9004969 | 20 |
| CNP | 0.520899 | 0.37641 | 0.1664008 | 0.9695681 | 20 |
| DHX58 | -0.0842882 | 0.178827 | 0.6373989 | 0.8580093 | 20 |
| STAT3 | -0.0496482 | 0.189834 | 0.7936803 | 0.9019264 | 20 |
| NAGLU | -0.0144376 | 0.0376763 | 0.7015706 | 0.8529767 | 20 |
| AOC2 | 0.411693 | 0.513389 | 0.4226037 | 0.8577856 | 20 |
| VAT1 | -0.341922 | 0.441147 | 0.4382955 | 0.1299237 | 20 |
| TMEM106A | -0.381598 | 0.501932 | 0.4470994 | 0.1849435 | 20 |
| ARL4D | 1.13299 | 0.594372 | 0.05662492 | 0.2155751 | 20 |
| PYY | -0.238905 | 0.394882 | 0.5451766 | 0.2561355 | 20 |
| GRN | -0.0456051 | 0.13807 | 0.741169 | 0.3053363 | 20 |
| C1QL1 | -0.109371 | 0.141203 | 0.4385961 | 0.2976836 | 20 |
| NMT1 | -0.500382 | 0.62707 | 0.4248889 | 0.221692 | 19 |
| HEXIM1 | 0.065611 | 0.347027 | 0.8500411 | 0.1983796 | 20 |
| HEXIM2 | 0.00905979 | 0.339451 | 0.9787074 | 0.2660047 | 20 |
| NSF | -0.189757 | 0.282142 | 0.5012276 | 0.821003 | 11 |
| CALCOCO2 | 0.277759 | 0.33668 | 0.4093752 | 0.7739629 | 20 |
| GIP | 0.0783643 | 0.63096 | 0.9011582 | 0.4062046 | 18 |
| GNGT2 | -0.00561559 | 0.857894 | 0.9947773 | 0.5634828 | 5 |
| COL1A1 | -0.231456 | 0.153981 | 0.1328026 | 0.2846409 | 6 |
| ACSF2 | 0.131546 | 0.721527 | 0.8553351 | 0.5971261 | 13 |
| CHAD | -0.288829 | 0.623412 | 0.6431466 | 0.1470401 | 17 |
| SPATA20 | 0.00950253 | 0.094557 | 0.9199512 | 0.005451575 | 20 |
| WFIKKN2 | -0.00780774 | 0.0564558 | 0.8900047 | 0.01067497 | 20 |
| NME2 | -0.162049 | 0.18413 | 0.3788177 | 0.2042145 | 20 |
| CA10 | 0.0957663 | 0.103122 | 0.3530589 | 0.5519252 | 5 |
| NOG | -0.0441118 | 0.104675 | 0.6734503 | 0.6601361 | 11 |
| LPO | -0.148685 | 0.106222 | 0.1615834 | 0.6334013 | 20 |
| MPO | 0.108232 | 0.098262 | 0.2706974 | 0.9457167 | 20 |
| MRC2 | 0.233164 | 0.559601 | 0.676926 | 0.5643328 | 6 |
| ACE | -0.0598219 | 0.0458095 | 0.1915919 | 0.1718185 | 20 |
| MAP3K3 | 0.279178 | 0.623352 | 0.6542495 | 0.1368674 | 20 |
| APOH | 0.0975841 | 0.0768259 | 0.2040143 | 0.2700123 | 20 |
| PRKCA | 0.471132 | 0.370899 | 0.2039971 | 0.956083 | 20 |
| KPNA2 | 0.0545472 | 0.463094 | 0.906235 | 0.5677567 | 14 |
| FAM20A | -0.16244 | 0.209068 | 0.4371749 | 0.8774938 | 20 |
| CD300A | 0.0353944 | 0.0515246 | 0.4921197 | 0.3656061 | 20 |
| CD300C | -0.193031 | 0.13841 | 0.1631265 | 0.1284235 | 19 |
| NT5C | 0.104638 | 0.0694201 | 0.1317312 | 0.8006741 | 20 |
| WBP2 | -0.993668 | 0.664647 | 0.1349063 | 0.193976 | 20 |
| PRPSAP1 | 0.0320194 | 0.369837 | 0.9310077 | 0.3483733 | 20 |
| SPHK1 | 0.415493 | 0.471115 | 0.3778115 | 0.9620662 | 8 |
| MXRA7 | -0.18002 | 0.102544 | 0.07916893 | 0.3147418 | 20 |
| TIMP2 | -0.657787 | 0.448219 | 0.142225 | 0.2090245 | 20 |
| LGALS3BP | -0.044063 | 0.192217 | 0.8186856 | 0.9635112 | 20 |
| ENGASE | -0.0633128 | 0.071901 | 0.3785587 | 0.1774075 | 20 |
| ENPP7 | 0.0181657 | 0.0322541 | 0.5732945 | 0.9605622 | 20 |
| GAA | 0.193311 | 0.574734 | 0.7366084 | 0.3813585 | 3 |
| NPTX1 | -0.156585 | 0.11362 | 0.1681595 | 0.5438111 | 20 |
| P4HB | 0.0886921 | 0.240502 | 0.712292 | 0.274825 | 20 |
| NOTUM | -0.137771 | 0.261922 | 0.5988889 | 0.3436258 | 15 |
| DCXR | -0.0880858 | 0.31779 | 0.7816403 | 0.3083868 | 20 |
| FASN | -0.0403583 | 0.185744 | 0.8279908 | 0.4051433 | 20 |
| CD7 | -0.0741544 | 0.167979 | 0.6588878 | 0.07568915 | 20 |
| SECTM1 | -0.0223449 | 0.154422 | 0.8849478 | 0.2055945 | 20 |
| COLEC12 | 0.530484 | 0.239482 | 0.02675097 | 0.07718767 | 20 |
| TWSG1 | -0.272124 | 0.340096 | 0.42363 | 0.6473526 | 20 |
| RAB31 | 0.270968 | 0.239222 | 0.2573381 | 0.5624117 | 20 |
| NAPG | -0.26351 | 0.394165 | 0.5037971 | 0.8426724 | 20 |
| CHST9 | 0.225016 | 0.127186 | 0.07686145 | 0.9950255 | 20 |
| DSC2 | 0.0969331 | 0.204955 | 0.6362501 | 0.7405432 | 20 |
| DSG2 | -0.130833 | 0.152046 | 0.3895259 | 0.9541677 | 20 |
| B4GALT6 | 0.00240558 | 0.0918325 | 0.9791016 | 0.8649583 | 20 |
| MAPRE2 | -0.250454 | 0.196152 | 0.2016591 | 0.9322152 | 20 |
| HDHD2 | -0.0622463 | 0.101753 | 0.540711 | 0.02180783 | 20 |
| POLI | -0.0471609 | 0.387231 | 0.9030652 | 0.3864441 | 18 |
| SERPINB5 | 0.0107764 | 0.21421 | 0.9598772 | 0.9824647 | 20 |
| SERPINB13 | -0.257904 | 0.285733 | 0.3667344 | 0.7263181 | 11 |
| SERPINB4 | -0.0431372 | 0.262997 | 0.8697142 | 0.5283883 | 6 |
| NETO1 | 0.107128 | 0.495148 | 0.8287108 | 0.7028958 | 20 |
| CYB5A | 0.170407 | 0.334695 | 0.6106531 | 0.2475096 | 20 |
| CNDP1 | -0.0148826 | 0.10503 | 0.8873182 | 0.8097928 | 20 |
| ZADH2 | 0.103936 | 0.307493 | 0.7353547 | 0.2410291 | 20 |
| GZMM | -0.730536 | 0.519218 | 0.1594295 | 0.8287384 | 3 |
| FSTL3 | -0.000130096 | 0.378613 | 0.9997258 | 0.5810898 | 19 |
| PRSS57 | 0.0108191 | 0.0640426 | 0.8658469 | 0.2845667 | 20 |
| PRTN3 | -0.0823679 | 0.0835874 | 0.3244227 | 0.08352621 | 20 |
| CFD | -0.380795 | 0.356611 | 0.2856024 | 0.005369938 | 10 |
| HMHA1 | 0.102951 | 0.233045 | 0.6586616 | 0.3842704 | 20 |
| AES | -0.0488384 | 0.550864 | 0.9293539 | 0.1185197 | 6 |
| EBI3 | -0.0264606 | 0.0275473 | 0.336779 | 0.6259863 | 20 |
| SEMA6B | -0.164146 | 0.323826 | 0.6122282 | 0.4701664 | 10 |
| PTPRS | 0.157241 | 0.255313 | 0.5379769 | 0.6499272 | 20 |
| FUT3 | -0.0492936 | 0.0456583 | 0.2803113 | 0.9835982 | 20 |
| FUT5 | -0.019809 | 0.0513414 | 0.6996232 | 0.9318563 | 20 |
| TNFSF14 | -0.164209 | 0.192725 | 0.3941948 | 0.6394635 | 20 |
| TNFSF14 | -0.353259 | 0.53993 | 0.5129389 | 0.5065321 | 7 |
| C3 | -0.0978145 | 0.444629 | 0.8258779 | 0.2240286 | 15 |
| C3 | -0.0828044 | 0.37634 | 0.8258515 | 0.3133679 | 18 |
| C3 | -0.240171 | 0.362831 | 0.5080127 | 0.665618 | 13 |
| C3 | -0.0982865 | 0.446772 | 0.8258771 | 0.2593935 | 16 |
| C3 | 0.062053 | 0.357461 | 0.8621846 | 0.5374098 | 20 |
| VAV1 | -0.163378 | 0.383876 | 0.670398 | 0.7097203 | 20 |
| INSR | -0.268941 | 0.441098 | 0.5420547 | 0.8572121 | 20 |
| RETN | 0.0664297 | 0.15015 | 0.6581828 | 0.2394451 | 5 |
| MCEMP1 | -0.390709 | 0.597665 | 0.5132888 | 0.6042799 | 8 |
| FCER2 | -0.0150479 | 0.106321 | 0.8874494 | 0.8716226 | 18 |
| CLEC4G | -0.436072 | 0.272276 | 0.1092481 | 0.9928221 | 20 |
| CD209 | -0.00100114 | 0.0874002 | 0.9908607 | 0.4372092 | 20 |
| CCL25 | 0.0126365 | 0.0451464 | 0.7795546 | 0.2467844 | 20 |
| ANGPTL4 | 0.267544 | 0.268873 | 0.3197076 | 0.6516332 | 5 |
| MUC16 | -0.372725 | 0.232493 | 0.1088977 | 0.4050245 | 11 |
| OLFM2 | -0.0269965 | 0.0482449 | 0.5757714 | 0.9694772 | 20 |
| ICAM1 | -0.0105276 | 0.0250677 | 0.6745096 | 0.7196093 | 20 |
| ICAM4 | -0.109433 | 0.273858 | 0.6894537 | 0.7592131 | 20 |
| ICAM5 | -0.00691383 | 0.0418568 | 0.8688037 | 0.3689253 | 20 |
| ICAM5 | -0.00702547 | 0.0425327 | 0.8688037 | 0.3563642 | 20 |
| PDE4A | 0.295842 | 0.222562 | 0.1837636 | 0.9514477 | 9 |
| PRKCSH | 0.0373164 | 0.280747 | 0.8942579 | 0.2543241 | 13 |
| CNN1 | 0.216234 | 0.2549 | 0.3962675 | 0.7399149 | 20 |
| ACP5 | 0.151655 | 0.140356 | 0.2799169 | 0.2767009 | 20 |
| IL27RA | -0.0053096 | 0.0352954 | 0.8804231 | 0.9701156 | 20 |
| CD97 | 0.0777781 | 0.240361 | 0.7462499 | 0.07088223 | 6 |
| DNAJB1 | 1.71926 | 0.641917 | 0.00739922 | 0.006919908 | 19 |
| ADGRE2 | 0.144908 | 0.0811701 | 0.07422313 | 0.9726966 | 17 |
| NOTCH3 | 0.249922 | 0.443163 | 0.5727879 | 0.06824671 | 20 |
| PGLYRP2 | 0.0788104 | 0.103418 | 0.4460276 | 0.3086706 | 20 |
| TPM4 | -0.706922 | 0.689738 | 0.3054041 | 0.8921248 | 3 |
| PGLS | -0.353306 | 0.456738 | 0.4392015 | 0.5498389 | 8 |
| COLGALT1 | -0.140446 | 0.224368 | 0.5313382 | 0.1871804 | 20 |
| IL12RB1 | -0.132479 | 0.386234 | 0.7315968 | 0.4599604 | 20 |
| JUND | -0.0806554 | 0.505306 | 0.8731829 | 0.6657911 | 20 |
| GDF15 | -0.0158145 | 0.0713548 | 0.8246007 | 0.5438549 | 20 |
| CRLF1 | 0.143472 | 0.128319 | 0.2635284 | 0.484635 | 20 |
| COMP | 0.13357 | 0.1672 | 0.4243666 | 0.8082992 | 20 |
| NCAN | -0.116608 | 0.169139 | 0.4905583 | 0.8068881 | 20 |
| HAPLN4 | -0.895541 | 0.432267 | 0.03829076 | 0.2863609 | 20 |
| CILP2 | -0.893442 | 0.410202 | 0.02940202 | 0.6556407 | 20 |
| PDCD5 | 0.0969891 | 0.102096 | 0.3421228 | 0.4627929 | 20 |
| ANKRD27 | -0.750716 | 0.560103 | 0.1801414 | 0.8011288 | 20 |
| DMKN | 0.167428 | 0.443595 | 0.705851 | 0.9429171 | 20 |
| IGFLR1 | -0.0426559 | 0.0815323 | 0.6008505 | 0.7718803 | 20 |
| KIRREL2 | 0.0428931 | 0.0621772 | 0.4902865 | 0.6898001 | 20 |
| TBCB | 0.127028 | 0.472108 | 0.7878786 | 0.9279297 | 8 |
| PPP1R14A | 0.354003 | 0.144859 | 0.01453473 | 0.01972775 | 20 |
| SPINT2 | 0.104922 | 0.0426741 | 0.01394486 | 0.009425638 | 20 |
| LGALS4 | 0.314931 | 0.539141 | 0.5591304 | 0.4061194 | 20 |
| ECH1 | -0.055519 | 0.107689 | 0.6061696 | 0.0279965 | 20 |
| SARS2 | -0.301614 | 0.279883 | 0.2811937 | 0.1718279 | 20 |
| PAK4 | -0.665432 | 0.654521 | 0.3093101 | 0.6413828 | 17 |
| IFNL3 | 0.883303 | 0.61087 | 0.1481842 | 0.8775016 | 5 |
| PLD3 | -0.00276479 | 0.681089 | 0.9967611 | 0.7919849 | 20 |
| BLVRB | 0.246966 | 0.379529 | 0.5152295 | 0.5887906 | 4 |
| LTBP4 | -0.0492656 | 0.386277 | 0.8985135 | 0.5256017 | 15 |
| MIA | 0.0373244 | 0.0334414 | 0.2643734 | 0.4638036 | 20 |
| B3GNT8 | 0.0305208 | 0.0361606 | 0.3986506 | 0.2311414 | 20 |
| CEACAM8 | 0.890574 | 0.437441 | 0.04176327 | 0.4609293 | 15 |
| PSG3 | 0.0199637 | 0.0529508 | 0.7061553 | 0.8417301 | 20 |
| PSG7 | 0.556752 | 0.340534 | 0.102062 | 0.8940439 | 20 |
| PSG5 | 0.0665453 | 0.0736116 | 0.3659926 | 0.2705268 | 20 |
| PSG4 | 0.0236413 | 0.0476786 | 0.6200024 | 0.2476477 | 20 |
| PSG9 | -0.143566 | 0.171876 | 0.4035555 | 0.9773381 | 20 |
| LYPD3 | 0.572033 | 0.25566 | 0.02525555 | 0.1665212 | 20 |
| PLAUR | -0.145177 | 0.18718 | 0.4379847 | 0.1068408 | 18 |
| BCAM | -0.0479797 | 0.0890042 | 0.5898365 | 0.6128434 | 12 |
| PVRL2 | -0.00925611 | 0.458913 | 0.983908 | 0.5655329 | 6 |
| APOE | 0.0976245 | 0.126329 | 0.4396537 | 0.8519571 | 20 |
| APOC1 | 0.121684 | 0.104787 | 0.2455371 | 0.3533087 | 20 |
| QPCTL | 0.0175306 | 0.117259 | 0.8811563 | 0.7420272 | 20 |
| PGLYRP1 | -0.00912929 | 0.178197 | 0.959141 | 0.8912991 | 20 |
| SEPW1 | 0.270983 | 0.229225 | 0.2371369 | 0.4726011 | 20 |
| SULT2A1 | 0.144581 | 0.190236 | 0.4472472 | 0.1266117 | 20 |
| SPHK2 | -0.541089 | 0.424353 | 0.2022765 | 0.002814958 | 20 |
| CA11 | 0.30092 | 0.208165 | 0.1482934 | 0.05928739 | 20 |
| BCAT2 | -0.11859 | 0.228203 | 0.6032958 | 0.6308968 | 20 |
| HSD17B14 | 0.822052 | 0.457436 | 0.07232161 | 0.26755 | 18 |
| NUCB1 | 0.19575 | 0.228075 | 0.3907414 | 0.1149435 | 20 |
| LHB | 0.0456211 | 0.0933457 | 0.6250307 | 0.6218427 | 20 |
| DKKL1 | 0.191768 | 0.670205 | 0.7747759 | 0.6782762 | 20 |
| IRF3 | -0.298701 | 0.43157 | 0.4888587 | 0.2468102 | 20 |
| PNKP | -0.0430958 | 0.290367 | 0.8820127 | 0.3313827 | 20 |
| LRRC4B | 0.0570012 | 0.405683 | 0.8882597 | 0.9666224 | 8 |
| CLEC11A | 0.00305729 | 0.0929042 | 0.973748 | 0.1003761 | 10 |
| CLEC11A | 0.00281901 | 0.0856635 | 0.973748 | 0.09174667 | 10 |
| KLK3 | 1.34245 | 0.676695 | 0.04727379 | 0.6703109 | 20 |
| KLK7 | -0.0392796 | 0.0876269 | 0.6539662 | 0.3895275 | 13 |
| KLK8 | -0.112131 | 0.133455 | 0.4007897 | 0.5157494 | 7 |
| KLK10 | 0.0456453 | 0.0471552 | 0.3330538 | 0.2547465 | 20 |
| KLK11 | -0.0322115 | 0.0321561 | 0.3164769 | 0.2955918 | 20 |
| KLK12 | -0.28591 | 0.206933 | 0.1670774 | 0.05644928 | 20 |
| KLK13 | -0.152832 | 0.0670319 | 0.02260866 | 0.4025573 | 20 |
| KLK14 | -0.0423998 | 0.172607 | 0.805958 | 0.5536882 | 14 |
| SIGLEC9 | 0.00124887 | 0.02363 | 0.9578505 | 0.6886598 | 20 |
| SIGLEC7 | 0.0357495 | 0.105086 | 0.7337102 | 0.2749707 | 14 |
| CD33 | 0.0168901 | 0.0277076 | 0.5421384 | 0.9379233 | 20 |
| IGLON5 | -0.0426658 | 0.261666 | 0.8704756 | 0.9340107 | 20 |
| SIGLEC12 | 0.0139933 | 0.0312696 | 0.6545116 | 0.09502787 | 20 |
| SIGLEC6 | 0.0664672 | 0.0497784 | 0.1817907 | 0.5353298 | 20 |
| SIGLEC5 | -0.00435449 | 0.0318497 | 0.8912521 | 0.2524585 | 20 |
| SIGLEC5 | -0.00417225 | 0.0305167 | 0.8912521 | 0.2165271 | 20 |
| SIGLEC14 | -0.00426799 | 0.031217 | 0.8912521 | 0.1917062 | 20 |
| LILRA6 | 0.0087893 | 0.0348215 | 0.8007238 | 0.3222843 | 20 |
| LILRB5 | -0.024767 | 0.0258104 | 0.3372687 | 0.2604517 | 20 |
| LILRB2 | 0.0124664 | 0.0308657 | 0.6862918 | 0.495258 | 20 |
| LILRA2 | 0.0016811 | 0.0732053 | 0.9816788 | 0.0417207 | 20 |
| LILRB1 | 0.0108148 | 0.0364467 | 0.7666728 | 0.8476513 | 20 |
| NCR1 | -0.191539 | 0.110315 | 0.08251333 | 0.7411354 | 20 |
| GP6 | 0.0482528 | 0.115389 | 0.675819 | 0.7407029 | 20 |
| TMEM190 | 0.0180397 | 0.0282974 | 0.5237981 | 0.1910413 | 20 |
| GALP | 0.299616 | 0.475697 | 0.5287959 | 0.9088437 | 3 |
| A1BG | -0.0252095 | 0.0957528 | 0.7923369 | 0.130407 | 20 |
| SNPH | -0.109302 | 0.473006 | 0.8172522 | 0.7830527 | 10 |
| SDCBP2 | 0.232308 | 0.661937 | 0.7256238 | 0.9920244 | 20 |
| NSFL1C | 0.911083 | 0.603584 | 0.1311823 | 0.5654832 | 20 |
| SIRPB1 | -0.0137062 | 0.0365653 | 0.7077781 | 0.9653436 | 20 |
| SIRPG | -0.0314762 | 0.204351 | 0.8775861 | 0.2326443 | 20 |
| SIRPA | 0.0071215 | 0.0234454 | 0.7613197 | 0.9534094 | 20 |
| TGM3 | -0.0290719 | 0.0878109 | 0.7405889 | 0.2082089 | 20 |
| CPXM1 | 0.0392775 | 0.0855574 | 0.6461783 | 0.004878264 | 20 |
| GNRH2 | 0.107215 | 0.178716 | 0.548561 | 0.8148619 | 20 |
| OXT | 0.0704878 | 0.0856358 | 0.4104448 | 0.03518302 | 20 |
| ITPA | 0.00870055 | 0.0447023 | 0.8456803 | 0.7509187 | 20 |
| ATRN | -0.0641852 | 0.0526589 | 0.2228874 | 0.9086 | 20 |
| SIGLEC1 | 0.890762 | 0.857523 | 0.2989154 | 0.1998387 | 4 |
| GPCPD1 | 0.0116112 | 0.40424 | 0.9770851 | 0.08750113 | 20 |
| JAG1 | -0.0334578 | 0.4324 | 0.9383236 | 0.746735 | 20 |
| FLRT3 | -0.0263764 | 0.0389067 | 0.4978103 | 0.4675495 | 20 |
| PCSK2 | -0.670979 | 0.354104 | 0.05811001 | 0.4320984 | 20 |
| DTD1 | 0.0568633 | 0.150263 | 0.7051144 | 0.7214801 | 20 |
| CST3 | -0.149376 | 0.135368 | 0.2698188 | 0.1671911 | 20 |
| CST4 | -0.0766547 | 0.142895 | 0.5916552 | 0.4319848 | 20 |
| CST1 | -0.052192 | 0.0653226 | 0.4242976 | 0.892793 | 20 |
| CST2 | -0.0529149 | 0.0662272 | 0.4242962 | 0.7367135 | 20 |
| CST5 | 0.0567158 | 0.0490585 | 0.2476463 | 0.06010234 | 20 |
| CST7 | -0.0313228 | 0.0690073 | 0.6498972 | 0.5687148 | 18 |
| APMAP | -0.0518827 | 0.405538 | 0.8982001 | 0.8290314 | 20 |
| ENTPD6 | -0.0563836 | 0.28073 | 0.8408191 | 0.7763472 | 20 |
| POFUT1 | 0.14699 | 0.101028 | 0.1456862 | 0.0747515 | 20 |
| MAPRE1 | -0.618079 | 0.495474 | 0.2122322 | 0.531902 | 20 |
| BPIFA2 | -0.280867 | 0.374026 | 0.4526954 | 0.0273654 | 20 |
| BPIFB1 | -0.0838842 | 0.0797231 | 0.2927102 | 0.1597041 | 20 |
| ASIP | 0.0900381 | 0.144611 | 0.5335333 | 0.219352 | 20 |
| MAP1LC3A | -1.28369 | 0.616776 | 0.03740778 | 0.1263303 | 20 |
| GSS | -0.349605 | 0.173411 | 0.04379511 | 0.4139724 | 20 |
| CPNE1 | 0.00229723 | 0.0496724 | 0.963113 | 0.1203059 | 20 |
| NDRG3 | 0.334182 | 0.278535 | 0.2302241 | 0.9986328 | 20 |
| TGM2 | -0.0188003 | 0.160106 | 0.906524 | 0.984409 | 20 |
| BPI | 0.0108011 | 0.0736562 | 0.883414 | 0.4300194 | 20 |
| LBP | -0.00675221 | 0.0393769 | 0.8638493 | 0.8832484 | 20 |
| PLCG1 | -0.392904 | 0.434439 | 0.3657865 | 0.1947141 | 20 |
| EMILIN3 | -0.0556472 | 0.161595 | 0.7305741 | 0.1460955 | 14 |
| WISP2 | -0.122932 | 0.122144 | 0.3141991 | 0.3723735 | 20 |
| YWHAB | 0.0604635 | 0.185971 | 0.7450876 | 0.5576936 | 20 |
| WFDC5 | 0.644521 | 0.524048 | 0.2187382 | 0.09758634 | 20 |
| PI3 | -0.0970328 | 0.106512 | 0.3622917 | 0.1736183 | 20 |
| SLPI | -0.661563 | 0.273478 | 0.0155603 | 0.1610683 | 20 |
| MATN4 | 0.0194609 | 0.124325 | 0.8756131 | 0.001562338 | 20 |
| WFDC2 | 0.642226 | 0.423162 | 0.1290943 | 0.7942969 | 20 |
| SPINT3 | -0.0325899 | 0.0944378 | 0.7300234 | 0.08595658 | 20 |
| UBE2C | -0.408889 | 0.21544 | 0.05770642 | 0.1741154 | 20 |
| PLTP | 0.127767 | 0.0742574 | 0.08532302 | 0.2311156 | 20 |
| MMP9 | -0.256515 | 0.325734 | 0.4309911 | 0.2459871 | 20 |
| CBLN4 | 0.306466 | 0.211235 | 0.1468274 | 0.425436 | 11 |
| CASS4 | -0.467675 | 0.61401 | 0.4462554 | 0.1990114 | 20 |
| BMP7 | -0.249244 | 0.528628 | 0.6372895 | 0.08547928 | 18 |
| RAB22A | -0.706558 | 0.453707 | 0.1193995 | 0.5543242 | 19 |
| CTSZ | -0.027191 | 0.106742 | 0.7989266 | 0.8493702 | 20 |
| TCEA2 | -0.154898 | 0.213217 | 0.467543 | 0.003778227 | 20 |
| HSPA13 | 0.435862 | 0.305529 | 0.1536999 | 0.9819663 | 20 |
| NCAM2 | 0.0140451 | 0.0708881 | 0.842943 | 0.9756915 | 20 |
| APP | 0.352716 | 0.393632 | 0.3702224 | 0.8116629 | 20 |
| ADAMTS5 | -0.242927 | 0.0707339 | 0.000593918 | 0.197026 | 20 |
| IL10RB | 0.0766133 | 0.403986 | 0.8495883 | 0.5972229 | 9 |
| IFNAR1 | -0.0146797 | 0.0666822 | 0.8257594 | 0.4197461 | 20 |
| CBR1 | -0.220972 | 0.169482 | 0.192298 | 0.5818273 | 20 |
| CBR3 | -0.0229559 | 0.0821514 | 0.7799121 | 0.602865 | 20 |
| DSCAM | -0.065946 | 0.180825 | 0.7153387 | 0.7778017 | 20 |
| FAM3B | 0.0557124 | 0.128171 | 0.6637996 | 0.4420297 | 14 |
| FAM3B | -0.061903 | 0.0983369 | 0.5290219 | 0.5325569 | 19 |
| MX1 | 0.00694162 | 0.0879165 | 0.9370668 | 0.8608202 | 20 |
| TFF3 | -0.0228706 | 0.336826 | 0.9458648 | 0.4839556 | 10 |
| TFF3 | -0.0285071 | 0.41984 | 0.9458653 | 0.496508 | 10 |
| TFF2 | 0.0901331 | 0.337905 | 0.7896688 | 0.5715052 | 20 |
| TFF1 | 0.161205 | 0.0979223 | 0.09971046 | 0.975747 | 20 |
| PDXK | 0.197387 | 0.232897 | 0.3967021 | 0.4444653 | 20 |
| ICOSLG | -0.38347 | 0.704041 | 0.5859808 | 0.6588797 | 6 |
| SUMO3 | 0.368241 | 0.506996 | 0.4676435 | 0.9327856 | 20 |
| COL18A1 | 0.263085 | 0.234887 | 0.2626924 | 0.1799342 | 20 |
| COL6A1 | 0.0151637 | 0.0261243 | 0.5616156 | 0.6324942 | 20 |
| COL6A2 | 0.140015 | 0.13141 | 0.2866568 | 0.9024712 | 20 |
| FTCD | 0.174957 | 0.0673248 | 0.009357914 | 0.2836247 | 6 |
| IL17RA | 0.0219178 | 0.0296787 | 0.4602096 | 0.2880093 | 20 |
| CECR1 | -0.00955721 | 0.0439834 | 0.8279812 | 0.3878258 | 20 |
| BID | -0.360796 | 0.283828 | 0.2036658 | 0.5969656 | 5 |
| DGCR14 | -0.33196 | 0.258874 | 0.1997309 | 0.2377615 | 10 |
| COMT | 0.0880168 | 0.34789 | 0.8002669 | 0.7032399 | 20 |
| RTN4R | 0.1152 | 0.114652 | 0.3150052 | 0.06306426 | 20 |
| SCARF2 | -0.35312 | 0.23708 | 0.1363681 | 0.448537 | 20 |
| SERPIND1 | 0.0262716 | 0.660513 | 0.9682729 | 0.4775314 | 20 |
| SNAP29 | 0.0097684 | 0.389906 | 0.9800125 | 0.2469151 | 20 |
| SDF2L1 | -1.01531 | 0.579381 | 0.07970362 | 0.9034671 | 4 |
| IGLL1 | 0.12533 | 0.10515 | 0.233292 | 0.801072 | 17 |
| MIF | -0.200319 | 0.440994 | 0.6496525 | 0.4910924 | 20 |
| DDT | 0.964675 | 0.353807 | 0.006399838 | 0.04288336 | 20 |
| SEZ6L | 1.05374 | 0.492775 | 0.03248579 | 0.1502779 | 10 |
| TPST2 | -0.218814 | 0.191145 | 0.2523099 | 0.6047403 | 20 |
| CRYBB1 | -0.00117635 | 0.167221 | 0.9943871 | 0.05800039 | 20 |
| KREMEN1 | -0.222678 | 0.141995 | 0.1168337 | 0.6498537 | 20 |
| TCN2 | -0.015703 | 0.0237257 | 0.5080654 | 0.8856189 | 20 |
| TCN2 | -0.0385182 | 0.0581993 | 0.5080787 | 0.4471624 | 20 |
| TIMP3 | -0.022891 | 0.0412101 | 0.5785728 | 0.7219342 | 20 |
| LARGE | 0.0371131 | 0.267279 | 0.8895646 | 0.3622072 | 20 |
| HMOX1 | 0.391514 | 0.509557 | 0.4422841 | 0.5956524 | 14 |
| APOL3 | 0.0277524 | 0.0427606 | 0.5163269 | 0.9435577 | 20 |
| APOL1 | 0.086882 | 0.0897976 | 0.3332783 | 0.1395322 | 19 |
| CSF2RB | -0.0936834 | 0.051464 | 0.06870287 | 0.8382507 | 20 |
| MPST | 0.381701 | 0.5952 | 0.5213285 | 0.8319522 | 3 |
| IL2RB | 0.141833 | 0.295566 | 0.6313217 | 0.7836542 | 20 |
| MFNG | -0.0463556 | 0.149071 | 0.7558288 | 0.1000094 | 13 |
| LGALS2 | 0.18774 | 0.145004 | 0.1954133 | 0.9421704 | 20 |
| NPTXR | -0.0131398 | 0.0445865 | 0.7682203 | 0.2820394 | 17 |
| APOBEC3G | 0.0237345 | 0.242229 | 0.921945 | 0.4543655 | 20 |
| GRAP2 | 0.365136 | 0.706152 | 0.6051011 | 0.926722 | 15 |
| ST13 | 0.193008 | 0.553702 | 0.7274073 | 0.02243217 | 20 |
| PMM1 | 0.346704 | 0.545557 | 0.5250999 | 0.4114302 | 20 |
| CCDC134 | -0.0084974 | 0.289595 | 0.9765916 | 0.3937903 | 20 |
| A4GALT | 0.0459532 | 0.0910791 | 0.6138803 | 0.08454592 | 20 |
| SCUBE1 | -0.195676 | 0.242801 | 0.420295 | 0.9713671 | 20 |
| FBLN1 | -0.196714 | 0.196063 | 0.3157051 | 0.03838159 | 20 |
| FAM19A5 | -0.95163 | 0.606888 | 0.1168698 | 0.2740445 | 14 |
| MAPK12 | -0.0838719 | 0.136794 | 0.5397945 | 0.2699471 | 20 |
| PLXNB2 | 0.0575411 | 0.0647425 | 0.3741269 | 0.1634258 | 20 |
| ADM2 | -0.44586 | 0.252851 | 0.07784478 | 0.8105713 | 20 |
| CHKB | 0.441983 | 0.474623 | 0.3517352 | 0.2579617 | 10 |
| ARSA | -0.7626 | 0.511947 | 0.136327 | 0.1973646 | 20 |
