# Supplementary material for: Blood metabolites as mediators in erectile dysfunction: insights from a multi-center proteomics and genetic study
Source: Front Pharmacol. 2025 Jun 2;16:1568780. doi: 10.3389/fphar.2025.1568780 (PMC12171135; doi:10.3389/fphar.2025.1568780)
Supplement: Supplementary file 4 [file Supplementaryfile2.docx]

| Table. | | | | | |
| --- | --- | --- | --- | --- | --- |
| b | se | pval | method | protein_ID | adjp |
| -0.292925875 | 0.128539229 | 0.022674016 | Inverse variance weighted (multiplicative random effects) | A1BG | 0.218342377 |
| -0.01478429 | 0.042092592 | 0.725413871 | Inverse variance weighted (multiplicative random effects) | AAMDC | 1 |
| 0.67147898 | 0.017018732 | 0 | Inverse variance weighted (multiplicative random effects) | AARSD1 | 0 |
| 0.626382182 | 0.500950777 | 0.211158325 | Wald ratio | ABCA2 | 0.770542661 |
| 0.020876666 | 0.05028111 | 0.677996247 | Inverse variance weighted (multiplicative random effects) | ABHD14B | 1 |
| 0.467118317 | 0.435542573 | 0.283496639 | Wald ratio | ABL1 | 0.848450373 |
| -0.015420491 | 0.05645833 | 0.78475292 | Inverse variance weighted (multiplicative random effects) | ABO | 1 |
| 0.144651189 | 0.057779699 | 0.012297341 | Inverse variance weighted (multiplicative random effects) | ACAA1 | 0.142896474 |
| -0.028837825 | 0.229208949 | 0.899878709 | Wald ratio | ACADM | 1 |
| 0.010128769 | 0.024720002 | 0.681996818 | Inverse variance weighted (multiplicative random effects) | ACADSB | 1 |
| 0.051701067 | 0.298661329 | 0.86256548 | Inverse variance weighted (multiplicative random effects) | ACAN | 1 |
| -0.071291844 | 0.116849593 | 0.541784755 | Inverse variance weighted (multiplicative random effects) | ACE | 0.972958495 |
| -0.072937075 | 0.114054202 | 0.52250106 | Inverse variance weighted (multiplicative random effects) | ACHE | 0.967500298 |
| 0.505217719 | 0.541811606 | 0.351098784 | Inverse variance weighted (multiplicative random effects) | ACOT13 | 0.898859417 |
| 0.006487083 | 0.307399565 | 0.983163414 | Inverse variance weighted (multiplicative random effects) | ACOX1 | 1 |
| 0.055050019 | 0.040871852 | 0.178014629 | Inverse variance weighted (multiplicative random effects) | ACP1 | 0.738570212 |
| 0.074097566 | 0.115108424 | 0.519757182 | Inverse variance weighted (multiplicative random effects) | ACP5 | 0.964751452 |
| -0.041197398 | 0.05199171 | 0.428136816 | Inverse variance weighted (multiplicative random effects) | ACP6 | 0.93094332 |
| 0.344180316 | 0.198626599 | 0.083131094 | Wald ratio | ACRBP | 0.516101454 |
| 0.203502695 | 0.604131945 | 0.736228919 | Wald ratio | ACRV1 | 1 |
| -0.102158002 | 0.474247298 | 0.82944703 | Wald ratio | ACTA2 | 1 |
| 0.093801567 | 0.094828322 | 0.322578758 | Inverse variance weighted (multiplicative random effects) | ACVRL1 | 0.874789851 |
| 0.063710868 | 0.157620548 | 0.686062656 | Inverse variance weighted (multiplicative random effects) | ACY1 | 1 |
| 0.106728814 | 0.054159706 | 0.048766058 | Inverse variance weighted (multiplicative random effects) | ACY3 | 0.387150385 |
| 0.00594476 | 0.060282869 | 0.921444422 | Inverse variance weighted (multiplicative random effects) | ACYP1 | 1 |
| -0.098024457 | 0.050781103 | 0.053565543 | Inverse variance weighted (multiplicative random effects) | ADA | 0.411130367 |
| -0.115318598 | 0.161699113 | 0.47574194 | Inverse variance weighted (multiplicative random effects) | ADA2 | 0.940832265 |
| 0.019141853 | 0.069401218 | 0.782690653 | Inverse variance weighted (multiplicative random effects) | ADAM12 | 1 |
| 0.04182919 | 0.055292313 | 0.449343447 | Inverse variance weighted (multiplicative random effects) | ADAM15 | 0.938113829 |
| -0.048516392 | 0.084583879 | 0.566245879 | Inverse variance weighted (multiplicative random effects) | ADAM22 | 0.987251825 |
| 0.044502005 | 0.059410791 | 0.453823502 | Inverse variance weighted (multiplicative random effects) | ADAM23 | 0.940832265 |
| 0.006274241 | 0.090554803 | 0.944761432 | Inverse variance weighted (multiplicative random effects) | ADAM8 | 1 |
| 0.242796142 | 0.381999044 | 0.525041353 | Inverse variance weighted (multiplicative random effects) | ADAM9 | 0.967500298 |
| -0.150351905 | 0.789767179 | 0.849015308 | Wald ratio | ADAMTS1 | 1 |
| -0.077259447 | 0.076980605 | 0.31556073 | Inverse variance weighted (multiplicative random effects) | ADAMTS13 | 0.873862934 |
| -0.191893579 | 0.235004319 | 0.414183686 | Inverse variance weighted (multiplicative random effects) | ADAMTS15 | 0.923405266 |
| -0.139031111 | 0.091555911 | 0.12887888 | Inverse variance weighted (multiplicative random effects) | ADAMTS16 | 0.642610785 |
| 0.060457839 | 0.242365544 | 0.803013498 | Wald ratio | ADAMTS4 | 1 |
| 0.027190343 | 0.08926119 | 0.760659002 | Inverse variance weighted (multiplicative random effects) | ADAMTS8 | 1 |
| 0.102737345 | 0.132138757 | 0.436866239 | Inverse variance weighted (multiplicative random effects) | ADAMTSL2 | 0.933907539 |
| 0.155506618 | 0.055091053 | 0.004761814 | Inverse variance weighted (multiplicative random effects) | ADAMTSL4 | 0.068781751 |
| 0.072004092 | 0.07701275 | 0.349807231 | Inverse variance weighted (multiplicative random effects) | ADAMTSL5 | 0.898270421 |
| -0.21165771 | 0.060743856 | 0.000493187 | Inverse variance weighted (multiplicative random effects) | ADD1 | 0.010575548 |
| -0.062436752 | 0.033014422 | 0.05859815 | Inverse variance weighted (multiplicative random effects) | ADGRB3 | 0.431167608 |
| -0.067819189 | 0.041410815 | 0.101480778 | Inverse variance weighted (multiplicative random effects) | ADGRD1 | 0.562880046 |
| 0.061402727 | 0.064529894 | 0.341330697 | Inverse variance weighted (multiplicative random effects) | ADGRE1 | 0.897122605 |
| 0.02353106 | 0.089813105 | 0.793321217 | Inverse variance weighted (multiplicative random effects) | ADGRE2 | 1 |
| -0.03522226 | 0.07327328 | 0.630731731 | Inverse variance weighted (multiplicative random effects) | ADGRE5 | 0.998066246 |
| 0.224823077 | 0.223601715 | 0.314674332 | Inverse variance weighted (multiplicative random effects) | ADGRG1 | 0.873862934 |
| -0.017660759 | 0.08817068 | 0.841244442 | Inverse variance weighted (multiplicative random effects) | ADH1B | 1 |
| 0.345946756 | 0.222795362 | 0.120481522 | Wald ratio | ADH4 | 0.618769297 |
| 0.084691082 | 0.136321883 | 0.534429777 | Inverse variance weighted (multiplicative random effects) | ADIPOQ | 0.971281608 |
| -0.002163187 | 0.179604573 | 0.990390378 | Wald ratio | ADM | 1 |
| 0.003333687 | 0.042328793 | 0.937225948 | Inverse variance weighted (multiplicative random effects) | AFAP1 | 1 |
| -0.013812807 | 0.162602983 | 0.932302687 | Inverse variance weighted (multiplicative random effects) | AFM | 1 |
| -0.302077765 | 0.017276351 | 1.86E-68 | Inverse variance weighted (multiplicative random effects) | AFP | 2.98E-66 |
| -0.155011037 | 0.090898124 | 0.08813343 | Inverse variance weighted (multiplicative random effects) | AGER | 0.523764385 |
| -0.445167274 | 0.265311011 | 0.093365204 | Wald ratio | AGR2 | 0.547041194 |
| 0.062884748 | 0.052637966 | 0.23221785 | Inverse variance weighted (multiplicative random effects) | AGRN | 0.793187763 |
| -0.384636931 | 0.423470433 | 0.363721318 | Inverse variance weighted (multiplicative random effects) | AGRP | 0.901939968 |
| -0.03514673 | 0.061910559 | 0.570237534 | Inverse variance weighted (multiplicative random effects) | AGT | 0.989304274 |
| -0.041723212 | 0.093240031 | 0.654527263 | Inverse variance weighted (multiplicative random effects) | AGXT | 0.998066246 |
| 0.060695462 | 0.059241723 | 0.305580709 | Inverse variance weighted (multiplicative random effects) | AHCY | 0.869694804 |
| 0.173836842 | 0.254767284 | 0.495026632 | Wald ratio | AHNAK | 0.95074367 |
| 0.001895822 | 0.10002783 | 0.984878646 | Inverse variance weighted (multiplicative random effects) | AHNAK2 | 1 |
| -0.015269397 | 0.062063506 | 0.805660098 | Inverse variance weighted (multiplicative random effects) | AHSG | 1 |
| -0.424293353 | 0.579991159 | 0.464442412 | Wald ratio | AHSP | 0.940832265 |
| 0.164458737 | 0.197712941 | 0.405518668 | Inverse variance weighted (multiplicative random effects) | AIF1 | 0.922595089 |
| 0.033974069 | 0.109852341 | 0.757115906 | Inverse variance weighted (multiplicative random effects) | AIF1L | 1 |
| 0.026470134 | 0.923222765 | 0.977126628 | Wald ratio | AK2 | 1 |
| -0.846350131 | 0.839147794 | 0.313174702 | Wald ratio | AKAP12 | 0.872182762 |
| 0.158293673 | 0.046158151 | 0.00060497 | Inverse variance weighted (multiplicative random effects) | AKR1B1 | 0.012710481 |
| 1.02349807 | 0.575977924 | 0.075572439 | Wald ratio | AKR1C4 | 0.495761534 |
| -0.232625726 | 0.263232003 | 0.37684258 | Inverse variance weighted (multiplicative random effects) | AKR7L | 0.909318524 |
| -0.088733909 | 0.521594371 | 0.864915351 | Wald ratio | AKT3 | 1 |
| -0.014400044 | 0.171125682 | 0.932938019 | Inverse variance weighted (multiplicative random effects) | ALCAM | 1 |
| 0.395244362 | 0.369724423 | 0.285058758 | Wald ratio | ALDH1A1 | 0.849458763 |
| -0.016988166 | 0.110252463 | 0.877543319 | Inverse variance weighted (multiplicative random effects) | ALDH3A1 | 1 |
| -0.031549314 | 0.187541008 | 0.866405319 | Inverse variance weighted (multiplicative random effects) | ALDH5A1 | 1 |
| -0.058913106 | 0.059130952 | 0.319096696 | Inverse variance weighted (multiplicative random effects) | ALPP | 0.873862934 |
| -0.033214367 | 0.390939528 | 0.932292896 | Wald ratio | AMBN | 1 |
| -0.08419171 | 0.189938976 | 0.657580122 | Inverse variance weighted (multiplicative random effects) | AMBP | 0.998066246 |
| -0.28088463 | 0.731745985 | 0.701085642 | Wald ratio | AMFR | 1 |
| 0.104531921 | 0.622413346 | 0.866625648 | Wald ratio | AMIGO1 | 1 |
| -0.136310257 | 0.159795361 | 0.393642598 | Inverse variance weighted (multiplicative random effects) | AMIGO2 | 0.915857498 |
| 0.127367243 | 0.043273351 | 0.003247144 | Inverse variance weighted (multiplicative random effects) | AMN | 0.049995613 |
| -0.106372507 | 0.476934221 | 0.82350911 | Wald ratio | AMOTL2 | 1 |
| -0.062921175 | 0.058289064 | 0.280379185 | Inverse variance weighted (multiplicative random effects) | AMPD3 | 0.847831902 |
| 0.01764402 | 0.059562283 | 0.767055915 | Inverse variance weighted (multiplicative random effects) | AMY1A | 1 |
| 0.004578629 | 0.059461901 | 0.938622703 | Inverse variance weighted (multiplicative random effects) | AMY1B | 1 |
| 0.013193809 | 0.055049755 | 0.810585607 | Inverse variance weighted (multiplicative random effects) | AMY1C | 1 |
| -0.03504305 | 0.054820745 | 0.522673492 | Inverse variance weighted (multiplicative random effects) | AMY2A | 0.967500298 |
| -0.024586458 | 0.053431449 | 0.645408869 | Inverse variance weighted (multiplicative random effects) | AMY2B | 0.998066246 |
| 0.00134913 | 0.029873175 | 0.963978237 | Inverse variance weighted (multiplicative random effects) | ANG | 1 |
| 0.212383805 | 0.167972664 | 0.206088268 | Inverse variance weighted (multiplicative random effects) | ANGPT1 | 0.769129582 |
| -0.076865961 | 0.094068293 | 0.413854526 | Inverse variance weighted (multiplicative random effects) | ANGPT2 | 0.923405266 |
| -0.035408685 | 0.043105123 | 0.411390231 | Inverse variance weighted (multiplicative random effects) | ANGPTL1 | 0.923405266 |
| 0.326324845 | 0.691748015 | 0.637112758 | Wald ratio | ANGPTL2 | 0.998066246 |
| -0.079934558 | 0.150432246 | 0.595165281 | Inverse variance weighted (multiplicative random effects) | ANGPTL3 | 0.989304274 |
| -0.059334628 | 0.102133085 | 0.561271433 | Inverse variance weighted (multiplicative random effects) | ANGPTL4 | 0.982697459 |
| 0.037131434 | 0.02924173 | 0.204152402 | Inverse variance weighted (multiplicative random effects) | ANGPTL7 | 0.767524355 |
| -0.050099163 | 0.363754045 | 0.890455289 | Wald ratio | ANKMY2 | 1 |
| -0.634664999 | 0.410699911 | 0.122267577 | Wald ratio | ANKRD54 | 0.623053939 |
| -0.041455921 | 0.071708994 | 0.563187648 | Inverse variance weighted (multiplicative random effects) | ANPEP | 0.983568688 |
| 0.554680616 | 0.563200029 | 0.324686357 | Wald ratio | ANXA10 | 0.877074835 |
| 0.194810133 | 0.467107606 | 0.676637285 | Wald ratio | ANXA11 | 1 |
| 0.080010542 | 0.053386907 | 0.133953497 | Inverse variance weighted (multiplicative random effects) | ANXA2 | 0.647227654 |
| -0.276713117 | 0.071877782 | 0.000118228 | Inverse variance weighted (multiplicative random effects) | ANXA3 | 0.002927539 |
| 0.049585363 | 0.56470681 | 0.93002986 | Wald ratio | ANXA4 | 1 |
| -0.055502082 | 0.273030137 | 0.838914711 | Wald ratio | ANXA5 | 1 |
| 0.106953575 | 0.012152132 | 1.35E-18 | Inverse variance weighted (multiplicative random effects) | AOC1 | 9.08E-17 |
| -0.043226812 | 0.117434017 | 0.712802796 | Inverse variance weighted (multiplicative random effects) | AOC3 | 1 |
| 0.125446335 | 0.075273719 | 0.095606775 | Inverse variance weighted (multiplicative random effects) | AP1G2 | 0.554700584 |
| 0.911321676 | 0.637967194 | 0.153154514 | Wald ratio | AP3B1 | 0.689161822 |
| 0.004325193 | 0.099982447 | 0.965494654 | Inverse variance weighted (multiplicative random effects) | APBB1IP | 1 |
| -0.19112788 | 0.08145214 | 0.018950395 | Inverse variance weighted (multiplicative random effects) | APCS | 0.192277183 |
| -0.057889455 | 0.071178118 | 0.416043728 | Inverse variance weighted (multiplicative random effects) | APEX1 | 0.923975972 |
| 0.638131512 | 0.4519747 | 0.157986944 | Wald ratio | APOA1 | 0.698760494 |
| 0.021378486 | 0.435213235 | 0.960822179 | Wald ratio | APOA2 | 1 |
| -0.060435632 | 0.032418753 | 0.062291056 | Inverse variance weighted (multiplicative random effects) | APOBR | 0.449879849 |
| -0.080400891 | 0.503649836 | 0.873167435 | Inverse variance weighted (multiplicative random effects) | APOC1 | 1 |
| -0.014723024 | 0.061518002 | 0.810850705 | Inverse variance weighted (multiplicative random effects) | APOD | 1 |
| -0.023181829 | 0.034690776 | 0.503979203 | Inverse variance weighted (multiplicative random effects) | APOE | 0.956220371 |
| -0.0331338 | 0.178321816 | 0.852594538 | Wald ratio | APOF | 1 |
| -0.019265802 | 0.075970346 | 0.799807417 | Inverse variance weighted (multiplicative random effects) | APOH | 1 |
| 0.091703564 | 0.123785068 | 0.45879715 | Wald ratio | APOL1 | 0.940832265 |
| -0.159226212 | 0.238839309 | 0.504985059 | Inverse variance weighted (multiplicative random effects) | APOM | 0.956220371 |
| 0.437333495 | 0.417279993 | 0.29461204 | Wald ratio | APP | 0.864306126 |
| -0.042257278 | 0.028678194 | 0.140616645 | Inverse variance weighted (multiplicative random effects) | APPL2 | 0.657666752 |
| 0.213634926 | 0.017766759 | 2.64E-33 | Inverse variance weighted (multiplicative random effects) | APRT | 2.62E-31 |
| 0.260711707 | 0.200875392 | 0.194329338 | Wald ratio | AREG | 0.753096526 |
| 0.001642047 | 0.179501769 | 0.992701208 | Wald ratio | ARFIP1 | 1 |
| -0.099282867 | 0.133428963 | 0.456823345 | Inverse variance weighted (multiplicative random effects) | ARG1 | 0.940832265 |
| -0.083990177 | 0.188590193 | 0.65606096 | Inverse variance weighted (multiplicative random effects) | ARG2 | 0.998066246 |
| -0.108510073 | 0.378608534 | 0.77441686 | Inverse variance weighted (multiplicative random effects) | ARHGAP25 | 1 |
| -0.137875398 | 0.038084941 | 0.000294367 | Inverse variance weighted (multiplicative random effects) | ARHGEF10 | 0.006655245 |
| 0.002225197 | 0.064344057 | 0.972412427 | Inverse variance weighted (multiplicative random effects) | ARHGEF5 | 1 |
| 0.314910117 | 0.689423893 | 0.647834322 | Inverse variance weighted (multiplicative random effects) | ARL2BP | 0.998066246 |
| 0.054918965 | 0.06705945 | 0.412809597 | Inverse variance weighted (multiplicative random effects) | ARSA | 0.923405266 |
| 0.021412981 | 0.056113662 | 0.702757978 | Inverse variance weighted (multiplicative random effects) | ARSB | 1 |
| 0.078907106 | 0.116496032 | 0.498191979 | Inverse variance weighted (multiplicative random effects) | ART3 | 0.955940329 |
| -0.179562036 | 0.211122802 | 0.395041653 | Inverse variance weighted (multiplicative random effects) | ART5 | 0.916038616 |
| 0.0876881 | 0.073666641 | 0.233914112 | Inverse variance weighted (multiplicative random effects) | ASAH1 | 0.793187763 |
| -0.068712426 | 0.03706118 | 0.063735303 | Inverse variance weighted (multiplicative random effects) | ASAH2 | 0.454367649 |
| -0.125905206 | 0.169644652 | 0.457984218 | Wald ratio | ASGR1 | 0.940832265 |
| 0.094101751 | 0.076234411 | 0.217063688 | Inverse variance weighted (multiplicative random effects) | ASGR2 | 0.778234308 |
| 0.214405387 | 0.123440018 | 0.082401363 | Wald ratio | ASPN | 0.516101454 |
| -0.066344094 | 0.139187472 | 0.633609403 | Wald ratio | ASPSCR1 | 0.998066246 |
| -0.016496799 | 0.038467787 | 0.668034494 | Inverse variance weighted (multiplicative random effects) | ASRGL1 | 0.999439337 |
| -0.189998706 | 0.023402825 | 4.72E-16 | Inverse variance weighted (multiplicative random effects) | ATOX1 | 2.65E-14 |
| -0.176991571 | 0.271716284 | 0.5147988 | Wald ratio | ATP5IF1 | 0.958622654 |
| -0.131290121 | 0.241890755 | 0.587290803 | Wald ratio | ATP6V1G2 | 0.989304274 |
| 0.146438597 | 0.110882688 | 0.186613956 | Inverse variance weighted (multiplicative random effects) | ATRAID | 0.745928931 |
| 0.0435857 | 0.035269551 | 0.216537248 | Inverse variance weighted (multiplicative random effects) | ATRN | 0.778234308 |
| 0.235141805 | 0.30560532 | 0.441638272 | Wald ratio | ATXN10 | 0.934538753 |
| 0.97973356 | 0.588716852 | 0.096075531 | Wald ratio | ATXN2L | 0.554700584 |
| -0.124329769 | 0.130088061 | 0.339205917 | Inverse variance weighted (multiplicative random effects) | ATXN3 | 0.897122605 |
| 0.332006786 | 0.599870138 | 0.579945428 | Wald ratio | AXIN1 | 0.989304274 |
| 0.261275584 | 0.195196209 | 0.18072439 | Inverse variance weighted (multiplicative random effects) | AXL | 0.740254766 |
| -0.176819377 | 0.246620597 | 0.47339309 | Inverse variance weighted (multiplicative random effects) | AZU1 | 0.940832265 |
| 0.077015238 | 0.095668761 | 0.420808305 | Inverse variance weighted (multiplicative random effects) | B3GNT7 | 0.927204739 |
| 0.116313681 | 0.06811071 | 0.087689237 | Inverse variance weighted (multiplicative random effects) | B4GALT1 | 0.522617802 |
| -0.096058015 | 0.096775179 | 0.320910103 | Inverse variance weighted (multiplicative random effects) | B4GAT1 | 0.873862934 |
| 0.201717721 | 0.694305202 | 0.771409448 | Wald ratio | BACH1 | 1 |
| 0.555070558 | 0.436917708 | 0.203933789 | Wald ratio | BAG3 | 0.767524355 |
| -0.129654858 | 0.349346616 | 0.710536766 | Wald ratio | BAIAP2 | 1 |
| -0.960143425 | 0.868246302 | 0.268794814 | Wald ratio | BAMBI | 0.835714817 |
| 0.014559546 | 0.309432446 | 0.962471446 | Inverse variance weighted (multiplicative random effects) | BANK1 | 1 |
| -0.716722602 | 0.365036988 | 0.049596858 | Wald ratio | BAP18 | 0.389288546 |
| 0.014698124 | 0.135027233 | 0.91331906 | Inverse variance weighted (multiplicative random effects) | BCAM | 1 |
| -0.035938391 | 0.065168027 | 0.581309638 | Inverse variance weighted (multiplicative random effects) | BCAN | 0.989304274 |
| -0.034718591 | 0.075576738 | 0.645959893 | Inverse variance weighted (multiplicative random effects) | BCAT1 | 0.998066246 |
| -0.000237269 | 0.075032525 | 0.997476925 | Inverse variance weighted (multiplicative random effects) | BCHE | 1 |
| -0.580719792 | 0.796041517 | 0.465690103 | Wald ratio | BCL2 | 0.940832265 |
| -0.408124076 | 0.149291613 | 0.006261995 | Inverse variance weighted (multiplicative random effects) | BCL2L15 | 0.082964721 |
| -1.102070475 | 0.39463836 | 0.005228491 | Wald ratio | BCR | 0.072988338 |
| -0.268316463 | 0.385228787 | 0.486108262 | Wald ratio | BDNF | 0.943195135 |
| 0.214983632 | 0.290864597 | 0.45983457 | Inverse variance weighted (multiplicative random effects) | BGLAP | 0.940832265 |
| -0.392472585 | 0.295585733 | 0.184251064 | Wald ratio | BID | 0.741887316 |
| 0.24351185 | 0.304962887 | 0.424582325 | Wald ratio | BIN2 | 0.92774274 |
| -0.015432487 | 0.416244397 | 0.970424772 | Wald ratio | BIRC2 | 1 |
| 0.09756245 | 0.077660509 | 0.209018642 | Inverse variance weighted (multiplicative random effects) | BLMH | 0.769129582 |
| -0.120523687 | 0.438135026 | 0.783252139 | Wald ratio | BLNK | 1 |
| -0.191151849 | 0.30370646 | 0.529089368 | Inverse variance weighted (multiplicative random effects) | BLVRB | 0.96875518 |
| 0.101919852 | 0.083061967 | 0.219809839 | Inverse variance weighted (multiplicative random effects) | BMP10 | 0.782884359 |
| -0.171939448 | 0.207681135 | 0.407726492 | Inverse variance weighted (multiplicative random effects) | BMP6 | 0.92281948 |
| -0.047673827 | 0.103481718 | 0.645015248 | Inverse variance weighted (multiplicative random effects) | BMPER | 0.998066246 |
| 0.521946438 | 0.580149242 | 0.368292617 | Wald ratio | BNIP3L | 0.907640574 |
| 0.174607732 | 0.162049414 | 0.281258361 | Inverse variance weighted (multiplicative random effects) | BOC | 0.847831902 |
| 0.353284561 | 0.319865435 | 0.269385537 | Wald ratio | BOLA1 | 0.836301367 |
| 0.045340235 | 0.170733964 | 0.790577539 | Inverse variance weighted (multiplicative random effects) | BOLA2 | 1 |
| 0.09114407 | 0.180585756 | 0.613760072 | Inverse variance weighted (multiplicative random effects) | BOLA2B | 0.994282983 |
| 0.237947807 | 0.318415273 | 0.454889934 | Inverse variance weighted (multiplicative random effects) | BPIFA2 | 0.940832265 |
| 0.005318401 | 0.085799914 | 0.950573915 | Inverse variance weighted (multiplicative random effects) | BPIFB1 | 1 |
| 0.069907625 | 0.057575887 | 0.224678099 | Inverse variance weighted (multiplicative random effects) | BPIFB2 | 0.786751592 |
| -0.010920562 | 0.059922569 | 0.85539081 | Inverse variance weighted (multiplicative random effects) | BRSK2 | 1 |
| -0.211204027 | 0.356240134 | 0.553268272 | Inverse variance weighted (multiplicative random effects) | BSG | 0.977576895 |
| 0.036080143 | 0.030664031 | 0.239344208 | Inverse variance weighted (multiplicative random effects) | BST1 | 0.793187763 |
| -0.165681106 | 0.036462075 | 5.52E-06 | Inverse variance weighted (multiplicative random effects) | BST2 | 0.000179449 |
| 0.025153552 | 0.057437277 | 0.661436455 | Inverse variance weighted (multiplicative random effects) | BTC | 0.998066246 |
| 0.015312324 | 0.112657076 | 0.891884703 | Inverse variance weighted (multiplicative random effects) | BTD | 1 |
| -0.214946835 | 0.034075255 | 2.83E-10 | Inverse variance weighted (multiplicative random effects) | BTN1A1 | 1.22E-08 |
| -0.021264655 | 0.12325966 | 0.863029387 | Inverse variance weighted (multiplicative random effects) | BTN2A1 | 1 |
| 0.055571941 | 0.07222404 | 0.441633167 | Inverse variance weighted (multiplicative random effects) | BTN3A2 | 0.934538753 |
| 0.000270724 | 0.067301872 | 0.996790496 | Inverse variance weighted (multiplicative random effects) | C1QA | 1 |
| 0.126122553 | 0.111515458 | 0.258060488 | Inverse variance weighted (multiplicative random effects) | C1QL2 | 0.815451469 |
| 0.067946455 | 0.038266162 | 0.075794311 | Inverse variance weighted (multiplicative random effects) | C1QTNF1 | 0.495761534 |
| 0.10698488 | 0.222210329 | 0.630191216 | Wald ratio | C1QTNF5 | 0.998066246 |
| -0.757832539 | 0.501926955 | 0.131082656 | Wald ratio | C1QTNF6 | 0.643958937 |
| -0.044532048 | 0.042310649 | 0.292569237 | Inverse variance weighted (multiplicative random effects) | C1QTNF9 | 0.861960357 |
| 0.003702866 | 0.050292024 | 0.941306953 | Inverse variance weighted (multiplicative random effects) | C1R | 1 |
| -0.041382477 | 0.094510424 | 0.661486681 | Inverse variance weighted (multiplicative random effects) | C1RL | 0.998066246 |
| -0.053773198 | 0.064929936 | 0.407572724 | Inverse variance weighted (multiplicative random effects) | C1S | 0.92281948 |
| 0.112506296 | 0.078997519 | 0.154395805 | Inverse variance weighted (multiplicative random effects) | C2 | 0.690630697 |
| -0.240482422 | 0.532684487 | 0.651662535 | Wald ratio | C2CD2L | 0.998066246 |
| 0.00978293 | 0.423810163 | 0.98158384 | Wald ratio | C2orf69 | 1 |
| -0.067437125 | 0.222261399 | 0.761574704 | Wald ratio | C3 | 1 |
| -0.160310874 | 0.215685929 | 0.457323861 | Inverse variance weighted (multiplicative random effects) | C4BPB | 0.940832265 |
| 0.353967521 | 0.200151763 | 0.07697808 | Wald ratio | C5 | 0.496242433 |
| -0.07760964 | 0.056345044 | 0.168388744 | Inverse variance weighted (multiplicative random effects) | C7 | 0.716254779 |
| 0.066930926 | 0.074700763 | 0.370259614 | Inverse variance weighted (multiplicative random effects) | C7orf50 | 0.909083377 |
| -0.006477995 | 0.071093859 | 0.927398108 | Inverse variance weighted (multiplicative random effects) | C8B | 1 |
| 0.009640197 | 0.145797519 | 0.947281931 | Inverse variance weighted (multiplicative random effects) | C9 | 1 |
| -0.25517483 | 0.34195852 | 0.455537101 | Wald ratio | CA1 | 0.940832265 |
| -0.133973385 | 0.400451525 | 0.737960183 | Wald ratio | CA11 | 1 |
| -0.074167089 | 0.062463621 | 0.235083856 | Inverse variance weighted (multiplicative random effects) | CA12 | 0.793187763 |
| -0.048136447 | 0.130650017 | 0.712546574 | Inverse variance weighted (multiplicative random effects) | CA13 | 1 |
| 0.127360506 | 0.37625057 | 0.734987052 | Inverse variance weighted (multiplicative random effects) | CA14 | 1 |
| -0.161212951 | 0.576493385 | 0.779750599 | Inverse variance weighted (multiplicative random effects) | CA2 | 1 |
| 0.177430611 | 0.131629328 | 0.177672366 | Inverse variance weighted (multiplicative random effects) | CA3 | 0.738570212 |
| 0.040258606 | 0.04836997 | 0.405236372 | Inverse variance weighted (multiplicative random effects) | CA4 | 0.922595089 |
| 0.033683069 | 0.106621389 | 0.752068641 | Inverse variance weighted (multiplicative random effects) | CA5A | 1 |
| -0.235012426 | 0.053704692 | 1.21E-05 | Inverse variance weighted (multiplicative random effects) | CA6 | 0.00036972 |
| -0.250430528 | 0.13095373 | 0.055830179 | Inverse variance weighted (multiplicative random effects) | CA9 | 0.423820336 |
| -0.102726173 | 0.308872241 | 0.739447815 | Wald ratio | CACNB3 | 1 |
| -0.484062534 | 0.302430542 | 0.109471251 | Wald ratio | CACYBP | 0.590852599 |
| -0.473986868 | 0.408651703 | 0.246097735 | Wald ratio | CALB1 | 0.799734834 |
| -0.162647702 | 0.467840598 | 0.728098366 | Wald ratio | CALB2 | 1 |
| -0.036407137 | 0.28573881 | 0.898612701 | Wald ratio | CALCA | 1 |
| 0.061495799 | 0.278381033 | 0.825166302 | Inverse variance weighted (multiplicative random effects) | CALCB | 1 |
| -0.541200943 | 0.225651268 | 0.016467083 | Wald ratio | CALCOCO1 | 0.175806724 |
| -0.428206951 | 0.505788265 | 0.397210832 | Wald ratio | CALCOCO2 | 0.917383701 |
| 0.055372299 | 0.089081052 | 0.534208483 | Inverse variance weighted (multiplicative random effects) | CAMKK1 | 0.971281608 |
| 0.345737893 | 0.379247494 | 0.361957276 | Inverse variance weighted (multiplicative random effects) | CANT1 | 0.900699853 |
| -0.112568066 | 0.04446608 | 0.011355991 | Inverse variance weighted (multiplicative random effects) | CAPG | 0.133468106 |
| -0.182870788 | 1.272038459 | 0.885688414 | Wald ratio | CAPN3 | 1 |
| 0.019306992 | 0.378913048 | 0.959362478 | Inverse variance weighted (multiplicative random effects) | CAPS | 1 |
| 0.526733125 | 0.267927094 | 0.049303587 | Inverse variance weighted (multiplicative random effects) | CARHSP1 | 0.389288546 |
| -0.441817648 | 0.523975504 | 0.399114994 | Wald ratio | CASP1 | 0.917383701 |
| 0.025588164 | 0.156421928 | 0.870058442 | Wald ratio | CASP10 | 1 |
| 0.254123824 | 0.248954589 | 0.307366376 | Wald ratio | CASP3 | 0.869694804 |
| -0.205433439 | 0.116098662 | 0.076814985 | Inverse variance weighted (multiplicative random effects) | CASP7 | 0.496242433 |
| -0.181584526 | 0.118022107 | 0.123910855 | Inverse variance weighted (multiplicative random effects) | CASP8 | 0.624054668 |
| -0.388584914 | 0.360392242 | 0.280932132 | Wald ratio | CASP9 | 0.847831902 |
| -0.137356323 | 0.086265084 | 0.111326608 | Inverse variance weighted (multiplicative random effects) | CAT | 0.594296188 |
| -0.261261904 | 0.570861512 | 0.647194937 | Inverse variance weighted (multiplicative random effects) | CBLIF | 0.998066246 |
| -0.113119796 | 0.119111707 | 0.342267237 | Inverse variance weighted (multiplicative random effects) | CBLN4 | 0.897122605 |
| 0.100113591 | 0.345623473 | 0.772075706 | Inverse variance weighted (multiplicative random effects) | CC2D1A | 1 |
| -0.150300992 | 0.187697567 | 0.42326971 | Inverse variance weighted (multiplicative random effects) | CCDC50 | 0.92774274 |
| -0.312398018 | 0.063055384 | 7.26E-07 | Inverse variance weighted (multiplicative random effects) | CCDC80 | 2.70E-05 |
| 0.074654261 | 0.049724725 | 0.133264821 | Inverse variance weighted (multiplicative random effects) | CCER2 | 0.647227654 |
| -0.060475748 | 0.041341746 | 0.143515214 | Inverse variance weighted (multiplicative random effects) | CCL13 | 0.66475921 |
| 0.07749867 | 0.042552981 | 0.068572241 | Inverse variance weighted (multiplicative random effects) | CCL14 | 0.466783731 |
| -0.05048987 | 0.043639394 | 0.247280862 | Inverse variance weighted (multiplicative random effects) | CCL15 | 0.799734834 |
| 0.005516394 | 0.036932027 | 0.881264737 | Inverse variance weighted (multiplicative random effects) | CCL16 | 1 |
| -0.121576004 | 0.092329164 | 0.187916711 | Inverse variance weighted (multiplicative random effects) | CCL17 | 0.745928931 |
| 0.018776833 | 0.053085874 | 0.723558655 | Inverse variance weighted (multiplicative random effects) | CCL18 | 1 |
| 0.1893997 | 0.37505275 | 0.613562467 | Wald ratio | CCL19 | 0.994282983 |
| 0.437306869 | 0.510726984 | 0.39186288 | Wald ratio | CCL2 | 0.915857498 |
| -0.06666253 | 0.343815899 | 0.846261866 | Wald ratio | CCL20 | 1 |
| 0.552549092 | 0.305433539 | 0.070440923 | Wald ratio | CCL21 | 0.474165438 |
| -0.156533403 | 0.140961693 | 0.266797551 | Inverse variance weighted (multiplicative random effects) | CCL22 | 0.830746866 |
| 0.028552257 | 0.068107183 | 0.675051449 | Inverse variance weighted (multiplicative random effects) | CCL23 | 1 |
| 0.049112441 | 0.047170951 | 0.297801951 | Inverse variance weighted (multiplicative random effects) | CCL24 | 0.866332948 |
| 0.023573037 | 0.058592392 | 0.687446708 | Inverse variance weighted (multiplicative random effects) | CCL25 | 1 |
| 0.048291167 | 0.017567777 | 0.005980493 | Inverse variance weighted (multiplicative random effects) | CCL26 | 0.080807974 |
| 0.122712725 | 0.225226256 | 0.585862188 | Inverse variance weighted (multiplicative random effects) | CCL27 | 0.989304274 |
| -0.064332406 | 0.504099582 | 0.898450931 | Wald ratio | CCL28 | 1 |
| -0.016927442 | 0.098683514 | 0.863804981 | Wald ratio | CCL3 | 1 |
| 0.003048199 | 0.049447759 | 0.950845675 | Inverse variance weighted (multiplicative random effects) | CCL4 | 1 |
| -0.035170595 | 0.171256569 | 0.837284728 | Wald ratio | CCL5 | 1 |
| 0.008337008 | 0.582113798 | 0.988573123 | Wald ratio | CCL7 | 1 |
| -0.005136483 | 0.043401756 | 0.905792436 | Inverse variance weighted (multiplicative random effects) | CCL8 | 1 |
| -0.316057785 | 0.107457092 | 0.003268944 | Inverse variance weighted (multiplicative random effects) | CCN1 | 0.049995613 |
| -0.035177485 | 0.034379744 | 0.306211489 | Inverse variance weighted (multiplicative random effects) | CCN2 | 0.869694804 |
| -0.110678372 | 0.145856803 | 0.44796306 | Inverse variance weighted (multiplicative random effects) | CCN3 | 0.936445391 |
| 0.083273807 | 0.069479255 | 0.230706077 | Inverse variance weighted (multiplicative random effects) | CCN4 | 0.793187763 |
| -0.097489034 | 0.071534627 | 0.172938305 | Inverse variance weighted (multiplicative random effects) | CCN5 | 0.72933915 |
| -0.052183533 | 0.042738702 | 0.222089759 | Inverse variance weighted (multiplicative random effects) | CCS | 0.783552201 |
| 0.043622409 | 0.034663734 | 0.208230802 | Inverse variance weighted (multiplicative random effects) | CD101 | 0.769129582 |
| 0.120862242 | 0.034369686 | 0.000437217 | Inverse variance weighted (multiplicative random effects) | CD109 | 0.009572756 |
| 0.091622744 | 0.106393773 | 0.389146393 | Inverse variance weighted (multiplicative random effects) | CD14 | 0.91224413 |
| 0.356267815 | 0.255109904 | 0.162555919 | Inverse variance weighted (multiplicative random effects) | CD163 | 0.701473133 |
| 0.369395637 | 0.273355227 | 0.176586796 | Inverse variance weighted (multiplicative random effects) | CD164 | 0.738468749 |
| -0.059940346 | 0.064034332 | 0.349239332 | Inverse variance weighted (multiplicative random effects) | CD164L2 | 0.898270421 |
| -0.018548884 | 0.034137423 | 0.586882491 | Inverse variance weighted (multiplicative random effects) | CD177 | 0.989304274 |
| -0.179598553 | 0.199107464 | 0.367047208 | Inverse variance weighted (multiplicative random effects) | CD1C | 0.905644357 |
| 0.332286228 | 0.776882579 | 0.668856851 | Wald ratio | CD2 | 0.999439337 |
| -0.115743258 | 0.144963475 | 0.424620716 | Inverse variance weighted (multiplicative random effects) | CD200 | 0.92774274 |
| -0.065508327 | 0.054265215 | 0.227359671 | Inverse variance weighted (multiplicative random effects) | CD200R1 | 0.78949602 |
| 0.005771345 | 0.07871841 | 0.941554399 | Inverse variance weighted (multiplicative random effects) | CD207 | 1 |
| -0.025380511 | 0.046649685 | 0.586395999 | Inverse variance weighted (multiplicative random effects) | CD209 | 0.989304274 |
| 0.012451653 | 0.119273978 | 0.916855587 | Inverse variance weighted (multiplicative random effects) | CD22 | 1 |
| 0.041647703 | 0.582236715 | 0.942975525 | Inverse variance weighted (multiplicative random effects) | CD226 | 1 |
| -0.084567024 | 0.012217615 | 4.46E-12 | Inverse variance weighted (multiplicative random effects) | CD244 | 2.16E-10 |
| 0.484117569 | 0.172265695 | 0.004949598 | Inverse variance weighted (multiplicative random effects) | CD248 | 0.070392204 |
| 0.090303761 | 0.07659248 | 0.238391793 | Inverse variance weighted (multiplicative random effects) | CD27 | 0.793187763 |
| 0.023236178 | 0.064206972 | 0.717430548 | Inverse variance weighted (multiplicative random effects) | CD274 | 1 |
| -0.174343926 | 0.075555171 | 0.02102669 | Inverse variance weighted (multiplicative random effects) | CD276 | 0.207412682 |
| -0.198720911 | 0.048423869 | 4.06E-05 | Inverse variance weighted (multiplicative random effects) | CD28 | 0.001120354 |
| 0.095173801 | 0.040125521 | 0.017696786 | Inverse variance weighted (multiplicative random effects) | CD2AP | 0.184046576 |
| -0.20628029 | 0.091746343 | 0.024552214 | Inverse variance weighted (multiplicative random effects) | CD300A | 0.230038765 |
| -0.018291416 | 0.066108858 | 0.78202098 | Inverse variance weighted (multiplicative random effects) | CD300C | 1 |
| 0.025699978 | 0.125318643 | 0.837511925 | Inverse variance weighted (multiplicative random effects) | CD300E | 1 |
| 0.053385033 | 0.067320171 | 0.427776637 | Inverse variance weighted (multiplicative random effects) | CD300LF | 0.93094332 |
| -0.183583568 | 0.086575218 | 0.033963105 | Inverse variance weighted (multiplicative random effects) | CD300LG | 0.290713001 |
| 0.168102812 | 0.097565216 | 0.084892333 | Inverse variance weighted (multiplicative random effects) | CD302 | 0.516101454 |
| 0.163273955 | 0.083482743 | 0.05049097 | Inverse variance weighted (multiplicative random effects) | CD33 | 0.394816605 |
| -1.161103403 | 0.490469273 | 0.017916872 | Wald ratio | CD34 | 0.185408429 |
| -0.019614793 | 0.178135067 | 0.912320622 | Inverse variance weighted (multiplicative random effects) | CD36 | 1 |
| -0.137535729 | 0.109839616 | 0.210515032 | Inverse variance weighted (multiplicative random effects) | CD38 | 0.769545283 |
| 0.012606998 | 0.117848384 | 0.914807688 | Inverse variance weighted (multiplicative random effects) | CD4 | 1 |
| -0.045025148 | 0.038831422 | 0.246251247 | Inverse variance weighted (multiplicative random effects) | CD40 | 0.799734834 |
| 0.295486267 | 0.323784684 | 0.361452363 | Wald ratio | CD46 | 0.900699853 |
| 0.060589206 | 0.061102974 | 0.321396704 | Inverse variance weighted (multiplicative random effects) | CD48 | 0.873862934 |
| 0.008689736 | 0.097242957 | 0.928794953 | Inverse variance weighted (multiplicative random effects) | CD5 | 1 |
| -0.028815575 | 0.095840547 | 0.76367253 | Inverse variance weighted (multiplicative random effects) | CD55 | 1 |
| -0.102933015 | 0.143852517 | 0.474272064 | Inverse variance weighted (multiplicative random effects) | CD58 | 0.940832265 |
| -0.185067804 | 0.069312061 | 0.007583625 | Inverse variance weighted (multiplicative random effects) | CD59 | 0.095654148 |
| -0.106596846 | 0.119514337 | 0.372437527 | Inverse variance weighted (multiplicative random effects) | CD5L | 0.909083377 |
| 0.000707358 | 0.065573195 | 0.991393141 | Inverse variance weighted (multiplicative random effects) | CD6 | 1 |
| -0.183114793 | 0.633445531 | 0.77252214 | Wald ratio | CD63 | 1 |
| -0.085460123 | 0.527112446 | 0.871204405 | Wald ratio | CD69 | 1 |
| -0.01124504 | 0.133212548 | 0.932727062 | Inverse variance weighted (multiplicative random effects) | CD7 | 1 |
| 0.105880544 | 0.051894286 | 0.04131924 | Inverse variance weighted (multiplicative random effects) | CD70 | 0.343776075 |
| 0.186466621 | 0.447615513 | 0.676987356 | Inverse variance weighted (multiplicative random effects) | CD72 | 1 |
| 1.126281671 | 0.656729262 | 0.086347743 | Wald ratio | CD74 | 0.516101454 |
| 0.215969247 | 0.18040572 | 0.23125557 | Wald ratio | CD79B | 0.793187763 |
| 0.05625578 | 0.061580848 | 0.360965333 | Inverse variance weighted (multiplicative random effects) | CD80 | 0.900699853 |
| -0.018264132 | 0.225199039 | 0.935360695 | Inverse variance weighted (multiplicative random effects) | CD83 | 1 |
| 0.136000409 | 0.225948896 | 0.547235495 | Wald ratio | CD84 | 0.975364036 |
| 0.185214932 | 0.234287575 | 0.429209402 | Inverse variance weighted (multiplicative random effects) | CD86 | 0.931333119 |
| -0.109798843 | 0.072756949 | 0.131268553 | Inverse variance weighted (multiplicative random effects) | CD8A | 0.643958937 |
| -0.04212021 | 0.072277003 | 0.560054285 | Inverse variance weighted (multiplicative random effects) | CDA | 0.981392512 |
| -0.038003161 | 0.393251737 | 0.923013677 | Wald ratio | CDC27 | 1 |
| 0.141838025 | 0.058091802 | 0.014621585 | Inverse variance weighted (multiplicative random effects) | CDCP1 | 0.160914794 |
| 0.763211237 | 0.618726243 | 0.217381795 | Wald ratio | CDH1 | 0.778234308 |
| -0.037582252 | 0.043091023 | 0.383121286 | Inverse variance weighted (multiplicative random effects) | CDH15 | 0.91151664 |
| 0.092943072 | 0.037637368 | 0.013532622 | Inverse variance weighted (multiplicative random effects) | CDH17 | 0.152150566 |
| -0.334013593 | 0.20130828 | 0.097072602 | Inverse variance weighted (multiplicative random effects) | CDH2 | 0.554700584 |
| -0.13901946 | 0.257677736 | 0.589535683 | Inverse variance weighted (multiplicative random effects) | CDH23 | 0.989304274 |
| 0.238992454 | 0.490649397 | 0.626191608 | Inverse variance weighted (multiplicative random effects) | CDH3 | 0.998066246 |
| 0.00546201 | 0.111884513 | 0.961064113 | Inverse variance weighted (multiplicative random effects) | CDH5 | 1 |
| -0.086855099 | 0.060839607 | 0.153404771 | Inverse variance weighted (multiplicative random effects) | CDH6 | 0.689161822 |
| 0.196096628 | 0.050301083 | 9.68E-05 | Inverse variance weighted (multiplicative random effects) | CDHR1 | 0.002426017 |
| -0.124017844 | 0.279411736 | 0.657148591 | Wald ratio | CDHR2 | 0.998066246 |
| 0.008925923 | 0.066618736 | 0.893414294 | Inverse variance weighted (multiplicative random effects) | CDHR5 | 1 |
| 0.011762857 | 0.047515482 | 0.804476098 | Inverse variance weighted (multiplicative random effects) | CDKN1A | 1 |
| -0.0154554 | 0.04730428 | 0.74387735 | Inverse variance weighted (multiplicative random effects) | CDNF | 1 |
| -0.042321282 | 0.262162407 | 0.871753523 | Inverse variance weighted (multiplicative random effects) | CDON | 1 |
| -0.013263302 | 0.040188351 | 0.741378405 | Inverse variance weighted (multiplicative random effects) | CDSN | 1 |
| -0.039240682 | 0.056802501 | 0.489674488 | Inverse variance weighted (multiplicative random effects) | CEACAM1 | 0.946123472 |
| -0.014530945 | 0.050279979 | 0.77258091 | Inverse variance weighted (multiplicative random effects) | CEACAM16 | 1 |
| 0.105604291 | 0.1662701 | 0.525339074 | Inverse variance weighted (multiplicative random effects) | CEACAM19 | 0.967500298 |
| 0.100668864 | 0.276975779 | 0.716263318 | Inverse variance weighted (multiplicative random effects) | CEACAM20 | 1 |
| -0.040901565 | 0.0322319 | 0.204448996 | Inverse variance weighted (multiplicative random effects) | CEACAM21 | 0.767524355 |
| 0.069847521 | 0.076076448 | 0.358554586 | Inverse variance weighted (multiplicative random effects) | CEACAM5 | 0.900699853 |
| 0.545814623 | 0.260081329 | 0.035849481 | Wald ratio | CEACAM6 | 0.301890369 |
| -0.148900145 | 0.501232808 | 0.766414721 | Wald ratio | CEACAM8 | 1 |
| -0.356052503 | 0.576580367 | 0.536888826 | Wald ratio | CEBPB | 0.971913628 |
| -0.251103413 | 0.625131955 | 0.687919063 | Inverse variance weighted (multiplicative random effects) | CELA2A | 1 |
| -0.06061209 | 0.043281526 | 0.161389142 | Inverse variance weighted (multiplicative random effects) | CELA3A | 0.700812975 |
| 0.020056888 | 0.079978871 | 0.801986266 | Inverse variance weighted (multiplicative random effects) | CELSR2 | 1 |
| 0.215424918 | 0.061567606 | 0.00046701 | Inverse variance weighted (multiplicative random effects) | CEMIP2 | 0.010118559 |
| 0.371841692 | 0.350848554 | 0.289219522 | Inverse variance weighted (multiplicative random effects) | CENPF | 0.854512224 |
| 0.030429066 | 0.125494698 | 0.808413738 | Wald ratio | CEP112 | 1 |
| 0.264895881 | 0.428169445 | 0.53613333 | Wald ratio | CEP152 | 0.971391399 |
| -0.234310013 | 0.435800331 | 0.590815591 | Wald ratio | CEP170 | 0.989304274 |
| -0.307979066 | 0.255895051 | 0.228768727 | Inverse variance weighted (multiplicative random effects) | CEP20 | 0.791745345 |
| 1.218794422 | 0.437377457 | 0.005326478 | Wald ratio | CEP43 | 0.073860498 |
| 0.080804544 | 0.352719925 | 0.818799107 | Wald ratio | CEP85 | 1 |
| -0.254186037 | 0.555473437 | 0.647238042 | Wald ratio | CERT | 0.998066246 |
| 0.008113758 | 0.036506269 | 0.824114155 | Inverse variance weighted (multiplicative random effects) | CES1 | 1 |
| 0.385784861 | 0.408658759 | 0.345155903 | Inverse variance weighted (multiplicative random effects) | CES2 | 0.897122605 |
| 0.041622954 | 0.086453083 | 0.630195748 | Inverse variance weighted (multiplicative random effects) | CES3 | 0.998066246 |
| -0.052203536 | 0.209656772 | 0.803364474 | Wald ratio | CETN3 | 1 |
| 0.136162826 | 0.044906862 | 0.002428457 | Inverse variance weighted (multiplicative random effects) | CFB | 0.039462424 |
| -0.12240746 | 0.048353751 | 0.011357622 | Inverse variance weighted (multiplicative random effects) | CFD | 0.133468106 |
| 0.02090559 | 0.031296583 | 0.504144348 | Inverse variance weighted (multiplicative random effects) | CFH | 0.956220371 |
| 0.054069012 | 0.031459616 | 0.08567266 | Inverse variance weighted (multiplicative random effects) | CFHR2 | 0.516101454 |
| -0.000259555 | 0.034773169 | 0.994044468 | Inverse variance weighted (multiplicative random effects) | CFHR4 | 1 |
| 0.021223135 | 0.086700341 | 0.806621192 | Inverse variance weighted (multiplicative random effects) | CFHR5 | 1 |
| 0.016387197 | 0.132182658 | 0.901335956 | Wald ratio | CFI | 1 |
| -1.322597441 | 0.837786169 | 0.114409154 | Wald ratio | CGA | 0.597917185 |
| -1.059012111 | 0.467950108 | 0.023630287 | Wald ratio | CGB3 | 0.223413619 |
| -1.059012111 | 0.467950108 | 0.023630287 | Wald ratio | CGB5 | 0.223413619 |
| -1.059012111 | 0.467950108 | 0.023630287 | Wald ratio | CGB8 | 0.223413619 |
| -0.046505627 | 0.062933946 | 0.459931686 | Inverse variance weighted (multiplicative random effects) | CGREF1 | 0.940832265 |
| -0.218393965 | 0.267681462 | 0.414573016 | Inverse variance weighted (multiplicative random effects) | CHAC2 | 0.923405266 |
| -0.145746079 | 0.011608955 | 3.75E-36 | Inverse variance weighted (multiplicative random effects) | CHAD | 3.90E-34 |
| -0.4421671 | 0.074871437 | 3.51E-09 | Inverse variance weighted (multiplicative random effects) | CHCHD10 | 1.43E-07 |
| -0.143914449 | 0.122099711 | 0.238532274 | Wald ratio | CHCHD6 | 0.793187763 |
| -0.050501864 | 0.373426706 | 0.892422893 | Inverse variance weighted (multiplicative random effects) | CHGA | 1 |
| -0.043595228 | 0.019940927 | 0.028799605 | Inverse variance weighted (multiplicative random effects) | CHGB | 0.253827031 |
| 0.032159164 | 0.04022823 | 0.424048178 | Inverse variance weighted (multiplicative random effects) | CHI3L1 | 0.92774274 |
| -0.017930117 | 0.030212614 | 0.55287027 | Inverse variance weighted (multiplicative random effects) | CHIT1 | 0.977576895 |
| 0.128195585 | 0.052975117 | 0.01552389 | Inverse variance weighted (multiplicative random effects) | CHL1 | 0.1684005 |
| 0.324444674 | 0.255504899 | 0.20414953 | Inverse variance weighted (multiplicative random effects) | CHMP1A | 0.767524355 |
| -0.028256232 | 0.105445976 | 0.788723328 | Inverse variance weighted (multiplicative random effects) | CHMP6 | 1 |
| -0.033916914 | 0.125221444 | 0.786502188 | Inverse variance weighted (multiplicative random effects) | CHRDL2 | 1 |
| 0.258699744 | 0.511815949 | 0.613239554 | Wald ratio | CIAPIN1 | 0.994282983 |
| 0.066475929 | 0.002267725 | 6.89E-189 | Inverse variance weighted (multiplicative random effects) | CILP | 1.79E-186 |
| 0.395195701 | 0.114451401 | 0.000554479 | Inverse variance weighted (multiplicative random effects) | CIT | 0.011768525 |
| 0.088008539 | 0.031010945 | 0.004539958 | Inverse variance weighted (multiplicative random effects) | CKAP4 | 0.066197083 |
| 0.07158463 | 0.240065909 | 0.765560135 | Wald ratio | CKMT1A | 1 |
| 0.07158463 | 0.240065909 | 0.765560135 | Wald ratio | CKMT1B | 1 |
| 0.414402128 | 0.3837662 | 0.280218038 | Wald ratio | CLC | 0.847831902 |
| -0.048949539 | 0.047996031 | 0.30779183 | Inverse variance weighted (multiplicative random effects) | CLEC10A | 0.869694804 |
| 0.053119431 | 0.060095026 | 0.376737279 | Inverse variance weighted (multiplicative random effects) | CLEC11A | 0.909318524 |
| 0.116782632 | 0.050615325 | 0.021040421 | Inverse variance weighted (multiplicative random effects) | CLEC12A | 0.207412682 |
| -0.097549668 | 0.343442623 | 0.776383687 | Wald ratio | CLEC14A | 1 |
| -0.001658629 | 0.043588251 | 0.969646055 | Inverse variance weighted (multiplicative random effects) | CLEC1A | 1 |
| -0.318110869 | 0.235314216 | 0.176421475 | Inverse variance weighted (multiplicative random effects) | CLEC1B | 0.738468749 |
| 0.136123124 | 0.103754704 | 0.189530049 | Inverse variance weighted (multiplicative random effects) | CLEC3B | 0.748050287 |
| -0.062348632 | 0.122852967 | 0.611799746 | Inverse variance weighted (multiplicative random effects) | CLEC4A | 0.994174588 |
| 0.005128267 | 0.05701605 | 0.928331496 | Inverse variance weighted (multiplicative random effects) | CLEC4C | 1 |
| 0.107316936 | 0.06333562 | 0.090186116 | Inverse variance weighted (multiplicative random effects) | CLEC4D | 0.532917956 |
| -0.257693332 | 0.085211522 | 0.00249324 | Inverse variance weighted (multiplicative random effects) | CLEC4G | 0.040201081 |
| 0.033671431 | 0.073480674 | 0.646783472 | Inverse variance weighted (multiplicative random effects) | CLEC4M | 0.998066246 |
| 0.04929824 | 0.169246661 | 0.770836855 | Inverse variance weighted (multiplicative random effects) | CLEC5A | 1 |
| 0.038375273 | 0.043924452 | 0.382300506 | Inverse variance weighted (multiplicative random effects) | CLEC6A | 0.91151664 |
| -0.012759552 | 0.044866304 | 0.776111108 | Inverse variance weighted (multiplicative random effects) | CLEC7A | 1 |
| 0.06998161 | 0.053013369 | 0.186810063 | Inverse variance weighted (multiplicative random effects) | CLGN | 0.745928931 |
| 0.050855182 | 0.245843415 | 0.836119153 | Wald ratio | CLIC5 | 1 |
| -0.132509313 | 0.162439099 | 0.41464448 | Inverse variance weighted (multiplicative random effects) | CLIP2 | 0.923405266 |
| 0.091665942 | 0.081655256 | 0.26160871 | Inverse variance weighted (multiplicative random effects) | CLMP | 0.821562438 |
| -0.008537175 | 0.069667406 | 0.902469883 | Inverse variance weighted (multiplicative random effects) | CLPS | 1 |
| 0.016801407 | 0.06746562 | 0.803332493 | Inverse variance weighted (multiplicative random effects) | CLSTN2 | 1 |
| -0.136669338 | 0.049233951 | 0.005504634 | Inverse variance weighted (multiplicative random effects) | CLSTN3 | 0.075825429 |
| -0.039056625 | 0.267456147 | 0.883897693 | Inverse variance weighted (multiplicative random effects) | CLU | 1 |
| 0.020426786 | 0.032981417 | 0.535690882 | Inverse variance weighted (multiplicative random effects) | CLUL1 | 0.971391399 |
| -0.227921896 | 0.474689431 | 0.631121088 | Wald ratio | CMC1 | 0.998066246 |
| -0.007742596 | 0.046505917 | 0.867774301 | Inverse variance weighted (multiplicative random effects) | CNDP1 | 1 |
| 0.487205 | 0.350898844 | 0.165000493 | Wald ratio | CNP | 0.70617495 |
| 0.085591223 | 0.12086694 | 0.47885571 | Inverse variance weighted (multiplicative random effects) | CNPY4 | 0.940832265 |
| 0.101498605 | 0.114051333 | 0.373499226 | Inverse variance weighted (multiplicative random effects) | CNTN1 | 0.909083377 |
| -0.080636332 | 0.042478495 | 0.057658419 | Inverse variance weighted (multiplicative random effects) | CNTN2 | 0.429854876 |
| 0.09669388 | 0.145810108 | 0.5072349 | Inverse variance weighted (multiplicative random effects) | CNTN3 | 0.956220371 |
| -0.079574325 | 0.072746332 | 0.274016247 | Inverse variance weighted (multiplicative random effects) | CNTN4 | 0.84116049 |
| -0.031077795 | 0.038571065 | 0.420399573 | Inverse variance weighted (multiplicative random effects) | CNTN5 | 0.927204739 |
| -0.105935901 | 0.009883136 | 8.30E-27 | Inverse variance weighted (multiplicative random effects) | CNTNAP2 | 7.20E-25 |
| 0.610387411 | 0.591268907 | 0.30191535 | Wald ratio | CNTNAP4 | 0.869694804 |
| 0.086818913 | 0.039394494 | 0.027536042 | Inverse variance weighted (multiplicative random effects) | COCH | 0.249021595 |
| -0.141674144 | 0.25289664 | 0.575339126 | Inverse variance weighted (multiplicative random effects) | COL15A1 | 0.989304274 |
| -0.003856116 | 0.163168695 | 0.981145591 | Inverse variance weighted (multiplicative random effects) | COL18A1 | 1 |
| 0.127805816 | 0.266419351 | 0.63142884 | Wald ratio | COL1A1 | 0.998066246 |
| -0.047289499 | 0.043113759 | 0.272705224 | Inverse variance weighted (multiplicative random effects) | COL24A1 | 0.84116049 |
| -0.01197221 | 0.03946467 | 0.761611523 | Inverse variance weighted (multiplicative random effects) | COL28A1 | 1 |
| 0.01832733 | 0.043983679 | 0.676908977 | Inverse variance weighted (multiplicative random effects) | COL2A1 | 1 |
| -0.380667302 | 0.577973132 | 0.510136885 | Wald ratio | COL3A1 | 0.956220371 |
| 0.181385093 | 0.110461256 | 0.100575539 | Inverse variance weighted (multiplicative random effects) | COL4A1 | 0.562880046 |
| 0.167034028 | 0.227744158 | 0.463297199 | Inverse variance weighted (multiplicative random effects) | COL5A1 | 0.940832265 |
| 0.159922831 | 0.106199742 | 0.132100971 | Inverse variance weighted (multiplicative random effects) | COL6A3 | 0.645000048 |
| -0.040781413 | 0.089683088 | 0.64930492 | Inverse variance weighted (multiplicative random effects) | COL9A1 | 0.998066246 |
| 0.102804471 | 0.140400095 | 0.464031199 | Inverse variance weighted (multiplicative random effects) | COLEC12 | 0.940832265 |
| -0.000268172 | 0.113063278 | 0.998107517 | Wald ratio | COMMD1 | 1 |
| 0.144329384 | 0.096813725 | 0.136015414 | Inverse variance weighted (multiplicative random effects) | COMP | 0.653045329 |
| 0.062638577 | 0.069731455 | 0.369034723 | Inverse variance weighted (multiplicative random effects) | COMT | 0.908393163 |
| 0.292282206 | 0.250641153 | 0.2435586 | Wald ratio | COQ7 | 0.795293389 |
| 0.118442661 | 0.180829746 | 0.51247068 | Wald ratio | CPA1 | 0.956342919 |
| -0.01093108 | 0.096348827 | 0.909671288 | Inverse variance weighted (multiplicative random effects) | CPA2 | 1 |
| -0.107940409 | 0.045663769 | 0.018088155 | Inverse variance weighted (multiplicative random effects) | CPA4 | 0.186254274 |
| 0.192343943 | 0.265171746 | 0.468233598 | Wald ratio | CPB1 | 0.940832265 |
| 0.082966197 | 0.046252545 | 0.072850686 | Inverse variance weighted (multiplicative random effects) | CPB2 | 0.485144086 |
| -0.078308569 | 0.096986762 | 0.419427414 | Inverse variance weighted (multiplicative random effects) | CPE | 0.927108418 |
| 0.371744156 | 0.202479495 | 0.066363645 | Inverse variance weighted (multiplicative random effects) | CPM | 0.462375861 |
| -0.082242777 | 0.187370712 | 0.660711678 | Inverse variance weighted (multiplicative random effects) | CPOX | 0.998066246 |
| -0.111713515 | 0.035958134 | 0.001891459 | Inverse variance weighted (multiplicative random effects) | CPPED1 | 0.031985649 |
| 0.071018544 | 0.061755819 | 0.250148194 | Inverse variance weighted (multiplicative random effects) | CPQ | 0.804185848 |
| 0.016434646 | 0.518276776 | 0.974703182 | Wald ratio | CPTP | 1 |
| 0.062379773 | 0.083396615 | 0.45446663 | Inverse variance weighted (multiplicative random effects) | CPVL | 0.940832265 |
| 0.021992089 | 0.054608418 | 0.687151698 | Inverse variance weighted (multiplicative random effects) | CPXM1 | 1 |
| 0.093030915 | 0.035002787 | 0.007864918 | Inverse variance weighted (multiplicative random effects) | CPXM2 | 0.097958257 |
| 0.081823745 | 0.063058382 | 0.194429247 | Inverse variance weighted (multiplicative random effects) | CR1 | 0.753096526 |
| 0.006850222 | 0.184460004 | 0.970376072 | Inverse variance weighted (multiplicative random effects) | CR2 | 1 |
| -0.057855207 | 0.031428812 | 0.065645978 | Inverse variance weighted (multiplicative random effects) | CRACR2A | 0.462375861 |
| 0.201679479 | 0.408155472 | 0.621218497 | Wald ratio | CRADD | 0.998066246 |
| 0.012955277 | 0.2873855 | 0.964043714 | Inverse variance weighted (multiplicative random effects) | CREG1 | 1 |
| 0.09929101 | 0.060436804 | 0.100405735 | Inverse variance weighted (multiplicative random effects) | CRELD1 | 0.562880046 |
| -0.090554837 | 0.055959541 | 0.105615241 | Inverse variance weighted (multiplicative random effects) | CRELD2 | 0.575460061 |
| -0.040603759 | 0.088395931 | 0.645990254 | Inverse variance weighted (multiplicative random effects) | CRH | 0.998066246 |
| 0.15054434 | 0.048889815 | 0.002075171 | Inverse variance weighted (multiplicative random effects) | CRHBP | 0.034809321 |
| -0.232073618 | 0.142304895 | 0.102928381 | Inverse variance weighted (multiplicative random effects) | CRIM1 | 0.568814265 |
| -0.019184466 | 0.060774004 | 0.752253791 | Inverse variance weighted (multiplicative random effects) | CRIP2 | 1 |
| -0.080197986 | 0.059672886 | 0.178961244 | Inverse variance weighted (multiplicative random effects) | CRISP2 | 0.738570212 |
| 0.017967628 | 0.042003414 | 0.668821569 | Inverse variance weighted (multiplicative random effects) | CRISP3 | 0.999439337 |
| 0.014390803 | 0.055204354 | 0.794337445 | Inverse variance weighted (multiplicative random effects) | CRNN | 1 |
| -0.028459302 | 0.074588048 | 0.702792864 | Inverse variance weighted (multiplicative random effects) | CRTAC1 | 1 |
| -0.037036144 | 0.08288777 | 0.655003023 | Inverse variance weighted (multiplicative random effects) | CRTAM | 0.998066246 |
| -0.001296415 | 0.184287965 | 0.994387148 | Wald ratio | CRYBB1 | 1 |
| 0.186396327 | 0.418645027 | 0.656148368 | Wald ratio | CRYBB2 | 0.998066246 |
| -0.048944922 | 0.064077413 | 0.444962314 | Inverse variance weighted (multiplicative random effects) | CRYGD | 0.934870317 |
| 0.061313717 | 0.074602554 | 0.411150004 | Inverse variance weighted (multiplicative random effects) | CRYM | 0.923405266 |
| -0.447173924 | 0.258719958 | 0.08391491 | Inverse variance weighted (multiplicative random effects) | CRYZL1 | 0.516101454 |
| -0.038893508 | 0.074313634 | 0.60071697 | Inverse variance weighted (multiplicative random effects) | CSF1 | 0.989304274 |
| 0.00587757 | 0.349514998 | 0.986583118 | Inverse variance weighted (multiplicative random effects) | CSF1R | 1 |
| -0.089953119 | 0.124996821 | 0.471744676 | Wald ratio | CSF2 | 0.940832265 |
| -0.029695654 | 0.053266099 | 0.577188378 | Inverse variance weighted (multiplicative random effects) | CSF2RB | 0.989304274 |
| 0.36855139 | 0.387692989 | 0.3417938 | Wald ratio | CSF3 | 0.897122605 |
| 0.013039786 | 0.041499183 | 0.753355559 | Inverse variance weighted (multiplicative random effects) | CSF3R | 1 |
| 0.05864127 | 0.148888355 | 0.693683896 | Inverse variance weighted (multiplicative random effects) | CSPG4 | 1 |
| -0.034433567 | 0.077006348 | 0.654765088 | Inverse variance weighted (multiplicative random effects) | CST1 | 0.998066246 |
| -0.124662487 | 0.00576698 | 1.25E-103 | Inverse variance weighted (multiplicative random effects) | CST3 | 2.37E-101 |
| 0.072936643 | 0.102534965 | 0.476877066 | Inverse variance weighted (multiplicative random effects) | CST5 | 0.940832265 |
| -0.037050497 | 0.052941888 | 0.484031398 | Inverse variance weighted (multiplicative random effects) | CST6 | 0.942682873 |
| -0.038588168 | 0.062674701 | 0.538099339 | Inverse variance weighted (multiplicative random effects) | CST7 | 0.972459772 |
| 0.029210143 | 0.040868702 | 0.474775059 | Inverse variance weighted (multiplicative random effects) | CSTB | 0.940832265 |
| -0.027246283 | 0.026811665 | 0.309529377 | Inverse variance weighted (multiplicative random effects) | CTBS | 0.869694804 |
| 0.834173937 | 0.554574592 | 0.132537845 | Wald ratio | CTF1 | 0.645617606 |
| 0.128066325 | 0.745072329 | 0.863528395 | Inverse variance weighted (multiplicative random effects) | CTHRC1 | 1 |
| 0.177633467 | 0.061057963 | 0.003622859 | Inverse variance weighted (multiplicative random effects) | CTRB1 | 0.05421256 |
| -0.010726093 | 0.054500655 | 0.843978826 | Inverse variance weighted (multiplicative random effects) | CTRC | 1 |
| -0.17637226 | 0.784122684 | 0.822034183 | Inverse variance weighted (multiplicative random effects) | CTRL | 1 |
| -0.013191354 | 0.052327806 | 0.800970952 | Inverse variance weighted (multiplicative random effects) | CTSB | 1 |
| 0.0018889 | 0.064192973 | 0.97652536 | Inverse variance weighted (multiplicative random effects) | CTSC | 1 |
| 0.20769833 | 0.158972367 | 0.191380585 | Inverse variance weighted (multiplicative random effects) | CTSD | 0.748540623 |
| -0.160510383 | 0.121638796 | 0.186980063 | Inverse variance weighted (multiplicative random effects) | CTSE | 0.745928931 |
| -0.232687837 | 0.167433443 | 0.164609868 | Inverse variance weighted (multiplicative random effects) | CTSF | 0.705955724 |
| -0.029149512 | 0.024600433 | 0.236049613 | Inverse variance weighted (multiplicative random effects) | CTSH | 0.793187763 |
| -0.001828648 | 0.157168523 | 0.990716862 | Inverse variance weighted (multiplicative random effects) | CTSO | 1 |
| 0.104230193 | 0.102137894 | 0.307498483 | Inverse variance weighted (multiplicative random effects) | CTSS | 0.869694804 |
| 0.196310475 | 0.129272656 | 0.128869017 | Inverse variance weighted (multiplicative random effects) | CTSV | 0.642610785 |
| -0.031830677 | 0.071659726 | 0.656903774 | Inverse variance weighted (multiplicative random effects) | CTSZ | 0.998066246 |
| 0.697391434 | 0.521891591 | 0.181458937 | Wald ratio | CWC15 | 0.741106653 |
| -0.027194317 | 0.100736429 | 0.787194762 | Inverse variance weighted (multiplicative random effects) | CX3CL1 | 1 |
| -0.064601765 | 0.608570361 | 0.915460705 | Inverse variance weighted (multiplicative random effects) | CXADR | 1 |
| 0.119924508 | 0.083899587 | 0.152894646 | Inverse variance weighted (multiplicative random effects) | CXCL1 | 0.689161822 |
| 0.100867997 | 0.061511234 | 0.101040416 | Inverse variance weighted (multiplicative random effects) | CXCL10 | 0.562880046 |
| -0.162127311 | 0.094471564 | 0.086134717 | Inverse variance weighted (multiplicative random effects) | CXCL11 | 0.516101454 |
| 0.078056304 | 0.087385409 | 0.371727675 | Inverse variance weighted (multiplicative random effects) | CXCL12 | 0.909083377 |
| -0.072071104 | 0.525394409 | 0.890892282 | Wald ratio | CXCL13 | 1 |
| -0.663431746 | 0.619516249 | 0.284220352 | Wald ratio | CXCL14 | 0.848563578 |
| -0.104023812 | 0.175287357 | 0.552881831 | Inverse variance weighted (multiplicative random effects) | CXCL16 | 0.977576895 |
| -0.081951343 | 0.481887345 | 0.86496037 | Inverse variance weighted (multiplicative random effects) | CXCL17 | 1 |
| -0.086935646 | 0.068083839 | 0.201640706 | Inverse variance weighted (multiplicative random effects) | CXCL5 | 0.767524355 |
| -0.001463341 | 0.051016981 | 0.977117093 | Inverse variance weighted (multiplicative random effects) | CXCL6 | 1 |
| -0.406472805 | 0.126564753 | 0.001320073 | Inverse variance weighted (multiplicative random effects) | CXCL8 | 0.024708167 |
| -0.422865358 | 0.133165194 | 0.001495813 | Inverse variance weighted (multiplicative random effects) | CXCL8 | 0.02659223 |
| -0.421628077 | 0.138047841 | 0.002256487 | Inverse variance weighted (multiplicative random effects) | CXCL8 | 0.037249946 |
| -0.411930617 | 0.124080452 | 0.000900603 | Inverse variance weighted (multiplicative random effects) | CXCL8 | 0.018186934 |
| -0.031563518 | 0.211500766 | 0.881367452 | Wald ratio | CXCL9 | 1 |
| 0.101978196 | 0.094569643 | 0.28088221 | Inverse variance weighted (multiplicative random effects) | CYB5R2 | 0.847831902 |
| -0.088603948 | 0.027727138 | 0.00139556 | Inverse variance weighted (multiplicative random effects) | CYTL1 | 0.025241441 |
| 0.139996673 | 0.483362793 | 0.772098827 | Wald ratio | DAG1 | 1 |
| 0.056389271 | 0.099879561 | 0.572364455 | Inverse variance weighted (multiplicative random effects) | DAPK2 | 0.989304274 |
| -0.068845642 | 0.285474009 | 0.809429129 | Wald ratio | DAPP1 | 1 |
| -0.051112021 | 0.064390899 | 0.427325212 | Inverse variance weighted (multiplicative random effects) | DARS1 | 0.93094332 |
| -0.018982246 | 0.053246439 | 0.721467693 | Inverse variance weighted (multiplicative random effects) | DBH | 1 |
| -0.003524288 | 0.078930383 | 0.964385819 | Inverse variance weighted (multiplicative random effects) | DBI | 1 |
| 0.483303139 | 0.608359087 | 0.426940907 | Wald ratio | DBN1 | 0.93094332 |
| -0.628009083 | 0.533554046 | 0.239183559 | Wald ratio | DBNL | 0.793187763 |
| -0.025390735 | 0.104195705 | 0.807476243 | Inverse variance weighted (multiplicative random effects) | DCBLD2 | 1 |
| 0.268846746 | 0.142310525 | 0.058870962 | Inverse variance weighted (multiplicative random effects) | DCC | 0.431167608 |
| 0.760739811 | 0.714775375 | 0.287190043 | Wald ratio | DCN | 0.853474736 |
| -0.402341508 | 0.551779338 | 0.46589705 | Wald ratio | DCTD | 0.940832265 |
| 0.530562042 | 0.564916832 | 0.3476352 | Wald ratio | DCTPP1 | 0.897122605 |
| -0.058127747 | 0.193879904 | 0.764319676 | Wald ratio | DCXR | 1 |
| -0.254770934 | 0.166698544 | 0.126429809 | Inverse variance weighted (multiplicative random effects) | DDAH1 | 0.635202903 |
| -0.040428039 | 0.066055391 | 0.540516324 | Inverse variance weighted (multiplicative random effects) | DDC | 0.972958495 |
| 0.077744142 | 0.334358932 | 0.81613647 | Wald ratio | DDHD2 | 1 |
| -0.820555143 | 0.904492936 | 0.364301539 | Wald ratio | DDI2 | 0.902080002 |
| 0.048255951 | 0.090978606 | 0.59582778 | Inverse variance weighted (multiplicative random effects) | DDR1 | 0.989304274 |
| -0.110861535 | 0.007172103 | 6.73E-54 | Inverse variance weighted (multiplicative random effects) | DDT | 8.24E-52 |
| 0.129502685 | 0.027249777 | 2.01E-06 | Inverse variance weighted (multiplicative random effects) | DDX58 | 6.85E-05 |
| 0.574257721 | 0.379636552 | 0.130368259 | Inverse variance weighted (multiplicative random effects) | DECR1 | 0.642802656 |
| -0.614094554 | 0.370008783 | 0.096979728 | Inverse variance weighted (multiplicative random effects) | DEFA1 | 0.554700584 |
| -0.614094554 | 0.370008783 | 0.096979728 | Inverse variance weighted (multiplicative random effects) | DEFA1B | 0.554700584 |
| 0.374634516 | 0.230859476 | 0.104636096 | Inverse variance weighted (multiplicative random effects) | DEFB104A | 0.572744944 |
| 0.009163811 | 0.13336671 | 0.945219359 | Inverse variance weighted (multiplicative random effects) | DEFB104B | 1 |
| -0.018827981 | 0.319621027 | 0.953026019 | Inverse variance weighted (multiplicative random effects) | DEFB4A | 1 |
| -0.205881199 | 0.062970743 | 0.001077477 | Inverse variance weighted (multiplicative random effects) | DFFA | 0.020945345 |
| -0.060430343 | 0.148347113 | 0.68374527 | Inverse variance weighted (multiplicative random effects) | DHRS4L2 | 1 |
| 0.059059303 | 0.519681779 | 0.909519111 | Inverse variance weighted (multiplicative random effects) | DKK1 | 1 |
| 0.089673563 | 0.056405435 | 0.111879081 | Inverse variance weighted (multiplicative random effects) | DKK3 | 0.594296188 |
| -0.055690226 | 0.243250518 | 0.818914106 | Wald ratio | DKK4 | 1 |
| -0.01328108 | 0.038695877 | 0.73143527 | Inverse variance weighted (multiplicative random effects) | DKKL1 | 1 |
| -0.016061335 | 0.05585657 | 0.773694265 | Inverse variance weighted (multiplicative random effects) | DLK1 | 1 |
| -0.103279645 | 0.143968525 | 0.47314178 | Inverse variance weighted (multiplicative random effects) | DLL1 | 0.940832265 |
| 0.467002003 | 1.084344375 | 0.666703365 | Wald ratio | DMP1 | 0.999439337 |
| 0.115189996 | 0.012745925 | 1.60E-19 | Inverse variance weighted (multiplicative random effects) | DNAJA4 | 1.11E-17 |
| 1.673512043 | 0.566973201 | 0.003160709 | Wald ratio | DNAJB1 | 0.049061749 |
| -0.629287807 | 0.581077937 | 0.278823394 | Wald ratio | DNAJB14 | 0.847831902 |
| 0.104442976 | 0.120390238 | 0.385647893 | Inverse variance weighted (multiplicative random effects) | DNAJB6 | 0.91224413 |
| -0.512211685 | 0.268916049 | 0.056815555 | Wald ratio | DNAJC21 | 0.426847399 |
| 0.000809382 | 0.368989913 | 0.998249835 | Wald ratio | DNAJC6 | 1 |
| 0.010415233 | 0.110644034 | 0.925003663 | Inverse variance weighted (multiplicative random effects) | DNER | 1 |
| 0.076293719 | 0.090487371 | 0.399148934 | Inverse variance weighted (multiplicative random effects) | DNM1 | 0.917383701 |
| 0.192079705 | 0.337483026 | 0.569251854 | Inverse variance weighted (multiplicative random effects) | DNMBP | 0.989304274 |
| 0.190807247 | 0.100866505 | 0.058533501 | Inverse variance weighted (multiplicative random effects) | DNPEP | 0.431167608 |
| 0.020606838 | 0.149564109 | 0.890414845 | Inverse variance weighted (multiplicative random effects) | DNPH1 | 1 |
| -0.815292923 | 0.462339796 | 0.077831983 | Wald ratio | DOC2B | 0.496668857 |
| -0.154519828 | 0.460950716 | 0.737459252 | Wald ratio | DOK2 | 1 |
| 0.039529624 | 0.062550869 | 0.527413261 | Inverse variance weighted (multiplicative random effects) | DPEP1 | 0.967500298 |
| -0.113002201 | 0.09765005 | 0.24718412 | Inverse variance weighted (multiplicative random effects) | DPEP2 | 0.799734834 |
| -0.105886058 | 0.17940722 | 0.555056881 | Inverse variance weighted (multiplicative random effects) | DPP10 | 0.977576895 |
| -0.088528414 | 0.095765359 | 0.355262246 | Inverse variance weighted (multiplicative random effects) | DPP4 | 0.900699853 |
| 0.180463344 | 0.358362851 | 0.614558551 | Inverse variance weighted (multiplicative random effects) | DPP6 | 0.994282983 |
| -0.096257318 | 0.061874413 | 0.119782159 | Inverse variance weighted (multiplicative random effects) | DPP7 | 0.618230496 |
| 0.000947593 | 0.090267165 | 0.991624241 | Inverse variance weighted (multiplicative random effects) | DPT | 1 |
| -0.680400096 | 0.662110516 | 0.304127128 | Wald ratio | DPY30 | 0.869694804 |
| 0.028302835 | 0.113284898 | 0.802712886 | Inverse variance weighted (multiplicative random effects) | DRAXIN | 1 |
| 0.045068864 | 0.098071853 | 0.645839663 | Inverse variance weighted (multiplicative random effects) | DSC2 | 0.998066246 |
| -0.002233947 | 0.199684389 | 0.99107394 | Wald ratio | DSCAM | 1 |
| -0.082294211 | 0.131955966 | 0.532858099 | Inverse variance weighted (multiplicative random effects) | DSG2 | 0.971281608 |
| 0.065079121 | 0.104935736 | 0.535138809 | Inverse variance weighted (multiplicative random effects) | DSG3 | 0.971281608 |
| 0.364224592 | 0.279501637 | 0.192533285 | Inverse variance weighted (multiplicative random effects) | DSG4 | 0.748540623 |
| 0.063314302 | 0.181824327 | 0.727677301 | Wald ratio | DTD1 | 1 |
| 1.673562056 | 1.702526303 | 0.325613596 | Wald ratio | DTNB | 0.878188209 |
| 0.053431761 | 0.307388022 | 0.862002899 | Wald ratio | DTX3 | 1 |
| -1.101101692 | 0.067362492 | 4.65E-60 | Inverse variance weighted (multiplicative random effects) | DTYMK | 6.05E-58 |
| 0.049876923 | 0.107432119 | 0.642458229 | Wald ratio | DUSP13 | 0.998066246 |
| -0.034707305 | 0.208027536 | 0.867495979 | Wald ratio | DUSP29 | 1 |
| 0.054273584 | 0.039499284 | 0.169429358 | Inverse variance weighted (multiplicative random effects) | DXO | 0.719210334 |
| 0.22628341 | 0.430028638 | 0.598745472 | Wald ratio | EBAG9 | 0.989304274 |
| -0.12735997 | 0.122246962 | 0.297492712 | Inverse variance weighted (multiplicative random effects) | EBI3 | 0.866332948 |
| -0.62279022 | 0.032253364 | 4.49E-83 | Inverse variance weighted (multiplicative random effects) | ECE1 | 7.77E-81 |
| 0.060620999 | 0.060872958 | 0.319317735 | Inverse variance weighted (multiplicative random effects) | ECHDC3 | 0.873862934 |
| 0.162617185 | 0.191085845 | 0.39476025 | Inverse variance weighted (multiplicative random effects) | ECHS1 | 0.916038616 |
| 0.000445331 | 0.09574605 | 0.996288919 | Inverse variance weighted (multiplicative random effects) | ECI2 | 1 |
| 0.023602462 | 0.078441982 | 0.763497851 | Inverse variance weighted (multiplicative random effects) | ECM1 | 1 |
| -0.067368319 | 0.126191726 | 0.593440566 | Inverse variance weighted (multiplicative random effects) | EDAR | 0.989304274 |
| 0.124183924 | 0.211975981 | 0.55798331 | Wald ratio | EDDM3B | 0.98024095 |
| -0.143905136 | 0.68325805 | 0.833186859 | Wald ratio | EDF1 | 1 |
| 0.351136694 | 0.20466173 | 0.08621825 | Inverse variance weighted (multiplicative random effects) | EDIL3 | 0.516101454 |
| 0.134894626 | 0.118253354 | 0.253984138 | Inverse variance weighted (multiplicative random effects) | EDN1 | 0.807778299 |
| 0.142117193 | 0.358625768 | 0.691896478 | Inverse variance weighted (multiplicative random effects) | EFCAB14 | 1 |
| 0.000612028 | 0.121320024 | 0.995974895 | Inverse variance weighted (multiplicative random effects) | EFCAB2 | 1 |
| 0.337161662 | 0.14769256 | 0.022438527 | Inverse variance weighted (multiplicative random effects) | EFEMP1 | 0.217079699 |
| -0.044658749 | 0.106667013 | 0.675454098 | Inverse variance weighted (multiplicative random effects) | EFHD1 | 1 |
| 0.069971138 | 0.091169679 | 0.442794663 | Inverse variance weighted (multiplicative random effects) | EFNA1 | 0.934538753 |
| 0.064694692 | 0.131101298 | 0.621679807 | Inverse variance weighted (multiplicative random effects) | EFNA4 | 0.998066246 |
| -0.146495845 | 0.494262632 | 0.766930185 | Inverse variance weighted (multiplicative random effects) | EGF | 1 |
| -0.044290263 | 0.14961254 | 0.767204811 | Inverse variance weighted (multiplicative random effects) | EGFL7 | 1 |
| 0.189230885 | 0.098686299 | 0.05517456 | Inverse variance weighted (multiplicative random effects) | EGFLAM | 0.420377603 |
| 0.049654499 | 0.150659579 | 0.741716759 | Inverse variance weighted (multiplicative random effects) | EGFR | 1 |
| -0.314306679 | 0.064810229 | 1.24E-06 | Inverse variance weighted (multiplicative random effects) | EGLN1 | 4.51E-05 |
| 0.247968503 | 0.194961303 | 0.203413715 | Wald ratio | EHBP1 | 0.767524355 |
| -0.093263426 | 0.165824465 | 0.573828127 | Wald ratio | EHD3 | 0.989304274 |
| -0.231190998 | 0.190881983 | 0.225829321 | Wald ratio | EIF2AK2 | 0.786975851 |
| -0.005203315 | 0.240539533 | 0.982741628 | Wald ratio | EIF2AK3 | 1 |
| -0.39775268 | 0.39894122 | 0.318754432 | Wald ratio | EIF4G3 | 0.873862934 |
| -0.092504573 | 0.417703585 | 0.824734397 | Wald ratio | EIF5 | 1 |
| 0.146947709 | 0.281657379 | 0.601861784 | Inverse variance weighted (multiplicative random effects) | ELAC1 | 0.989848745 |
| 0.602280244 | 0.435525849 | 0.166701482 | Wald ratio | ELN | 0.711989902 |
| 0.474910569 | 0.337278512 | 0.159111384 | Wald ratio | ELOA | 0.698760494 |
| 0.005899327 | 0.280359654 | 0.983212153 | Wald ratio | ENAH | 1 |
| -0.099728786 | 0.079501053 | 0.209684479 | Inverse variance weighted (multiplicative random effects) | ENDOU | 0.769187318 |
| -0.060315703 | 0.0951097 | 0.525969992 | Inverse variance weighted (multiplicative random effects) | ENG | 0.967500298 |
| -0.150400072 | 0.114800924 | 0.190163828 | Inverse variance weighted (multiplicative random effects) | ENO1 | 0.748536643 |
| -0.176830092 | 0.326603407 | 0.588217121 | Inverse variance weighted (multiplicative random effects) | ENO2 | 0.989304274 |
| 0.104493297 | 0.324514031 | 0.747453211 | Wald ratio | ENO3 | 1 |
| 0.513670208 | 0.431633499 | 0.234022404 | Wald ratio | ENPEP | 0.793187763 |
| 0.010226604 | 0.176761743 | 0.953863889 | Inverse variance weighted (multiplicative random effects) | ENPP2 | 1 |
| 0.056644012 | 0.040377212 | 0.160655367 | Inverse variance weighted (multiplicative random effects) | ENPP5 | 0.700551705 |
| 0.031106476 | 0.050105475 | 0.534718132 | Inverse variance weighted (multiplicative random effects) | ENPP6 | 0.971281608 |
| 0.005309831 | 0.034020863 | 0.875973328 | Inverse variance weighted (multiplicative random effects) | ENPP7 | 1 |
| -0.35621745 | 0.489554347 | 0.466836333 | Wald ratio | ENSA | 0.940832265 |
| -0.037338145 | 0.052513495 | 0.477071854 | Inverse variance weighted (multiplicative random effects) | ENTPD2 | 0.940832265 |
| 0.090014041 | 0.178838189 | 0.614734575 | Inverse variance weighted (multiplicative random effects) | ENTPD5 | 0.994282983 |
| -0.100982663 | 0.09051048 | 0.264549999 | Inverse variance weighted (multiplicative random effects) | ENTPD6 | 0.82622222 |
| 0.292153172 | 0.468437061 | 0.532840079 | Inverse variance weighted (multiplicative random effects) | ENTR1 | 0.971281608 |
| -0.87932438 | 0.592591144 | 0.137845132 | Wald ratio | EPGN | 0.653345785 |
| 0.209756268 | 0.025498709 | 1.93E-16 | Inverse variance weighted (multiplicative random effects) | EPHA1 | 1.15E-14 |
| -0.08972698 | 0.201905783 | 0.656753235 | Inverse variance weighted (multiplicative random effects) | EPHA2 | 0.998066246 |
| 0.105781032 | 0.073640088 | 0.150871523 | Inverse variance weighted (multiplicative random effects) | EPHA4 | 0.685180717 |
| 0.137269624 | 0.185274055 | 0.458753825 | Wald ratio | EPHB4 | 0.940832265 |
| -0.028074684 | 0.126859549 | 0.824854764 | Inverse variance weighted (multiplicative random effects) | EPHB6 | 1 |
| 0.111133508 | 0.187823814 | 0.554058067 | Inverse variance weighted (multiplicative random effects) | EPHX2 | 0.977576895 |
| 0.152032433 | 0.228907765 | 0.50658497 | Inverse variance weighted (multiplicative random effects) | EPO | 0.956220371 |
| -0.023449618 | 0.069115228 | 0.734396589 | Inverse variance weighted (multiplicative random effects) | EPPK1 | 1 |
| 0.222493811 | 0.144091428 | 0.122560348 | Inverse variance weighted (multiplicative random effects) | EPS8L2 | 0.623053939 |
| 0.005205019 | 0.059208361 | 0.929948052 | Inverse variance weighted (multiplicative random effects) | ERBB2 | 1 |
| -0.085470186 | 0.077489024 | 0.270028331 | Inverse variance weighted (multiplicative random effects) | ERBB3 | 0.837047584 |
| 0.259056245 | 0.139314257 | 0.062954892 | Inverse variance weighted (multiplicative random effects) | ERBB4 | 0.453100951 |
| 0.371828887 | 0.972414044 | 0.702181655 | Wald ratio | EREG | 1 |
| 0.836841831 | 0.413547076 | 0.043014321 | Wald ratio | ERI1 | 0.351441367 |
| 0.672730323 | 0.314540194 | 0.032453993 | Wald ratio | ERMAP | 0.280100852 |
| -0.24356089 | 0.245395221 | 0.320941493 | Wald ratio | ERN1 | 0.873862934 |
| -0.150270032 | 0.1653557 | 0.363472401 | Inverse variance weighted (multiplicative random effects) | ERP44 | 0.901939968 |
| 0.208725286 | 0.07258011 | 0.004030154 | Inverse variance weighted (multiplicative random effects) | ESAM | 0.059876581 |
| -0.451018112 | 0.095428299 | 2.29E-06 | Inverse variance weighted (multiplicative random effects) | ESM1 | 7.67E-05 |
| 0.307165898 | 0.333389856 | 0.356872179 | Inverse variance weighted (multiplicative random effects) | ESYT2 | 0.900699853 |
| 0.016679616 | 0.22901306 | 0.941939329 | Wald ratio | EVI5 | 1 |
| 0.118939754 | 0.090884192 | 0.190637435 | Inverse variance weighted (multiplicative random effects) | EXTL1 | 0.748536643 |
| 0.261074468 | 0.223222491 | 0.242173805 | Inverse variance weighted (multiplicative random effects) | F10 | 0.793187763 |
| -0.062224636 | 0.174683763 | 0.721681022 | Inverse variance weighted (multiplicative random effects) | F11 | 1 |
| 0.016904981 | 0.471815998 | 0.971418227 | Wald ratio | F11R | 1 |
| 0.042176075 | 0.04271582 | 0.323464082 | Inverse variance weighted (multiplicative random effects) | F12 | 0.875587163 |
| 0.020168233 | 0.100115321 | 0.840346709 | Inverse variance weighted (multiplicative random effects) | F13B | 1 |
| 0.080795511 | 0.09281146 | 0.384008769 | Inverse variance weighted (multiplicative random effects) | F2 | 0.91151664 |
| 0.115713521 | 0.028019239 | 3.63E-05 | Inverse variance weighted (multiplicative random effects) | F2R | 0.001048949 |
| -0.110945746 | 0.119157101 | 0.351808051 | Inverse variance weighted (multiplicative random effects) | F3 | 0.898968975 |
| 0.171872378 | 0.074840751 | 0.021646856 | Inverse variance weighted (multiplicative random effects) | F7 | 0.212384251 |
| -0.279168773 | 0.212761026 | 0.18947842 | Inverse variance weighted (multiplicative random effects) | FABP1 | 0.748050287 |
| 0.004808569 | 0.142099835 | 0.973005239 | Inverse variance weighted (multiplicative random effects) | FABP2 | 1 |
| 0.330942542 | 0.896351454 | 0.711970686 | Inverse variance weighted (multiplicative random effects) | FABP3 | 1 |
| 0.387118819 | 0.342986103 | 0.259036216 | Wald ratio | FABP4 | 0.816356558 |
| -0.144496838 | 0.022165453 | 7.08E-11 | Inverse variance weighted (multiplicative random effects) | FABP5 | 3.13E-09 |
| 0.19254785 | 0.216619755 | 0.374070348 | Wald ratio | FABP6 | 0.909083377 |
| 0.095920964 | 0.328296428 | 0.770150602 | Inverse variance weighted (multiplicative random effects) | FABP9 | 1 |
| 0.169893869 | 0.359260171 | 0.636285053 | Wald ratio | FADD | 0.998066246 |
| -0.068480407 | 0.124203189 | 0.581388367 | Inverse variance weighted (multiplicative random effects) | FAM13A | 0.989304274 |
| -0.19202277 | 0.075802115 | 0.011302281 | Inverse variance weighted (multiplicative random effects) | FAM171B | 0.133468106 |
| -0.185589282 | 0.669999742 | 0.781780847 | Inverse variance weighted (multiplicative random effects) | FAM172A | 1 |
| 0.048710831 | 0.015557325 | 0.001741799 | Inverse variance weighted (multiplicative random effects) | FAM20A | 0.030191175 |
| 0.011478889 | 0.050937971 | 0.821706737 | Inverse variance weighted (multiplicative random effects) | FAM3B | 1 |
| -0.03537356 | 0.290578721 | 0.903109003 | Wald ratio | FAM3C | 1 |
| 0.146745212 | 0.09670184 | 0.129140052 | Inverse variance weighted (multiplicative random effects) | FAM3D | 0.642610785 |
| 0.022633069 | 0.066758611 | 0.734588294 | Inverse variance weighted (multiplicative random effects) | FAP | 1 |
| 0.82661503 | 0.510920402 | 0.105685453 | Wald ratio | FARSA | 0.575460061 |
| -0.125695524 | 0.05431168 | 0.020649249 | Inverse variance weighted (multiplicative random effects) | FAS | 0.206492488 |
| -0.71133612 | 0.425576564 | 0.094629965 | Wald ratio | FASLG | 0.551345452 |
| -0.204837238 | 0.11050355 | 0.063786228 | Inverse variance weighted (multiplicative random effects) | FBLN2 | 0.454367649 |
| -0.283133286 | 0.089986487 | 0.00165295 | Inverse variance weighted (multiplicative random effects) | FBN2 | 0.028891902 |
| 0.096653921 | 0.052682359 | 0.066556917 | Inverse variance weighted (multiplicative random effects) | FBP1 | 0.462375861 |
| -0.138715924 | 0.092472417 | 0.133593961 | Inverse variance weighted (multiplicative random effects) | FCAMR | 0.647227654 |
| 0.04880866 | 0.06365678 | 0.443231815 | Inverse variance weighted (multiplicative random effects) | FCAR | 0.934538753 |
| 0.115008806 | 0.216127533 | 0.594633198 | Inverse variance weighted (multiplicative random effects) | FCER1A | 0.989304274 |
| 0.072575647 | 0.049243666 | 0.14053363 | Inverse variance weighted (multiplicative random effects) | FCER2 | 0.657666752 |
| 0.014028991 | 0.041761369 | 0.736921955 | Inverse variance weighted (multiplicative random effects) | FCGR2A | 1 |
| 0.112583553 | 0.053072567 | 0.03389543 | Inverse variance weighted (multiplicative random effects) | FCGR2B | 0.290713001 |
| -0.052963211 | 0.059329656 | 0.372021223 | Inverse variance weighted (multiplicative random effects) | FCGR3B | 0.909083377 |
| 0.093131361 | 0.060375728 | 0.122944895 | Inverse variance weighted (multiplicative random effects) | FCN1 | 0.623053939 |
| 0.001403678 | 0.036193066 | 0.969063349 | Inverse variance weighted (multiplicative random effects) | FCN2 | 1 |
| -0.067491214 | 0.031305283 | 0.031091091 | Inverse variance weighted (multiplicative random effects) | FCRL1 | 0.271295065 |
| 0.009022431 | 0.061286819 | 0.882961133 | Inverse variance weighted (multiplicative random effects) | FCRL2 | 1 |
| 0.004594756 | 0.055184846 | 0.933643855 | Inverse variance weighted (multiplicative random effects) | FCRL3 | 1 |
| 0.041976328 | 0.05466729 | 0.442575767 | Inverse variance weighted (multiplicative random effects) | FCRL5 | 0.934538753 |
| -0.008785032 | 0.045383419 | 0.846509761 | Inverse variance weighted (multiplicative random effects) | FCRL6 | 1 |
| 0.024548514 | 0.061096051 | 0.687829764 | Inverse variance weighted (multiplicative random effects) | FCRLB | 1 |
| 0.193640021 | 0.547530449 | 0.723593153 | Wald ratio | FDX1 | 1 |
| -0.243053258 | 0.217557814 | 0.263913402 | Wald ratio | FES | 0.825473499 |
| 0.046906002 | 0.044201043 | 0.288600504 | Inverse variance weighted (multiplicative random effects) | FETUB | 0.854512224 |
| -0.216991249 | 0.278148437 | 0.435315823 | Inverse variance weighted (multiplicative random effects) | FGA | 0.933284251 |
| -0.003057024 | 0.081796344 | 0.970187123 | Inverse variance weighted (multiplicative random effects) | FGF2 | 1 |
| 0.36108553 | 0.007612499 | 0 | Inverse variance weighted (multiplicative random effects) | FGF21 | 0 |
| 0.248991746 | 0.275508282 | 0.366125794 | Wald ratio | FGF23 | 0.904443766 |
| 0.058839579 | 0.062063059 | 0.343098288 | Inverse variance weighted (multiplicative random effects) | FGF5 | 0.897122605 |
| -0.448672771 | 0.327715876 | 0.170970913 | Inverse variance weighted (multiplicative random effects) | FGFBP1 | 0.724275964 |
| -0.169587316 | 0.058205649 | 0.003573004 | Inverse variance weighted (multiplicative random effects) | FGFBP2 | 0.053853974 |
| -0.084181951 | 0.065474921 | 0.198543275 | Inverse variance weighted (multiplicative random effects) | FGFBP3 | 0.759210165 |
| 0.252196394 | 0.256988284 | 0.326418368 | Wald ratio | FGFR2 | 0.878188209 |
| -0.089971138 | 0.060275733 | 0.135526371 | Inverse variance weighted (multiplicative random effects) | FGFR4 | 0.652534377 |
| -0.025632957 | 0.043150425 | 0.552487243 | Inverse variance weighted (multiplicative random effects) | FGL1 | 0.977576895 |
| -0.131049895 | 0.264260599 | 0.619956786 | Inverse variance weighted (multiplicative random effects) | FGR | 0.99750597 |
| 0.110862539 | 0.338531816 | 0.743304554 | Wald ratio | FHIT | 1 |
| -0.071400417 | 0.174551371 | 0.682502215 | Wald ratio | FIS1 | 1 |
| -0.076176095 | 0.597323797 | 0.898521737 | Wald ratio | FKBP14 | 1 |
| -0.170406254 | 0.178691045 | 0.340267813 | Inverse variance weighted (multiplicative random effects) | FKBP1B | 0.897122605 |
| 1.030586071 | 0.577282551 | 0.07422291 | Wald ratio | FKBP4 | 0.488713364 |
| -0.12336786 | 0.178423036 | 0.489292468 | Inverse variance weighted (multiplicative random effects) | FKBP5 | 0.946123472 |
| -0.372073678 | 0.44730227 | 0.405512189 | Inverse variance weighted (multiplicative random effects) | FKBP7 | 0.922595089 |
| -0.367544769 | 0.528371656 | 0.486668169 | Inverse variance weighted (multiplicative random effects) | FKBPL | 0.943401482 |
| -0.073595737 | 0.036105566 | 0.041515066 | Inverse variance weighted (multiplicative random effects) | FLRT2 | 0.344029236 |
| 0.434867644 | 0.345612479 | 0.208300581 | Wald ratio | FLT3 | 0.769129582 |
| -0.354924257 | 0.364625617 | 0.330357699 | Wald ratio | FLT3LG | 0.881012811 |
| 0.090981399 | 0.221619774 | 0.681417754 | Inverse variance weighted (multiplicative random effects) | FLT4 | 1 |
| -0.067456297 | 0.037696753 | 0.07354305 | Inverse variance weighted (multiplicative random effects) | FN1 | 0.487164152 |
| 0.159177177 | 0.064061106 | 0.012963481 | Inverse variance weighted (multiplicative random effects) | FNDC1 | 0.148154064 |
| 0.385198862 | 0.490420745 | 0.432192298 | Wald ratio | FNTA | 0.931458519 |
| -0.164746214 | 0.385033365 | 0.66874201 | Wald ratio | FOLH1 | 0.999439337 |
| 0.27853333 | 0.230671668 | 0.227244165 | Inverse variance weighted (multiplicative random effects) | FOLR1 | 0.78949602 |
| 0.175521276 | 0.187053777 | 0.348066318 | Inverse variance weighted (multiplicative random effects) | FOLR2 | 0.897122605 |
| 0.040036249 | 0.037292137 | 0.283009085 | Inverse variance weighted (multiplicative random effects) | FOLR3 | 0.848211666 |
| -0.155440394 | 0.410851565 | 0.70518018 | Wald ratio | FOXJ3 | 1 |
| -0.500608712 | 0.118632443 | 2.44E-05 | Inverse variance weighted (multiplicative random effects) | FOXO1 | 0.000726475 |
| 0.025317632 | 0.067767892 | 0.708706906 | Inverse variance weighted (multiplicative random effects) | FRZB | 1 |
| -0.329084403 | 0.225859957 | 0.145108503 | Inverse variance weighted (multiplicative random effects) | FSHB | 0.667755944 |
| 0.279933697 | 0.242120844 | 0.247610208 | Inverse variance weighted (multiplicative random effects) | FST | 0.799734834 |
| -0.099323969 | 0.369468264 | 0.788060891 | Inverse variance weighted (multiplicative random effects) | FSTL1 | 1 |
| 0.076842602 | 0.116094556 | 0.508037438 | Inverse variance weighted (multiplicative random effects) | FSTL3 | 0.956220371 |
| 0.002575443 | 0.152067736 | 0.986487548 | Inverse variance weighted (multiplicative random effects) | FTCD | 1 |
| 0.122413491 | 0.106815469 | 0.251783672 | Inverse variance weighted (multiplicative random effects) | FUCA1 | 0.805707749 |
| -0.002476398 | 0.480069375 | 0.995884197 | Inverse variance weighted (multiplicative random effects) | FUOM | 1 |
| 0.376926863 | 0.150340376 | 0.012170678 | Inverse variance weighted (multiplicative random effects) | FURIN | 0.142219162 |
| 0.014272779 | 0.032561936 | 0.661149178 | Inverse variance weighted (multiplicative random effects) | FUT3 | 0.998066246 |
| 0.014272779 | 0.032561936 | 0.661149178 | Inverse variance weighted (multiplicative random effects) | FUT5 | 0.998066246 |
| -0.006100694 | 0.052370173 | 0.907262811 | Inverse variance weighted (multiplicative random effects) | FUT8 | 1 |
| -0.433145289 | 0.367947203 | 0.239118029 | Wald ratio | FXN | 0.793187763 |
| 0.084405428 | 0.068202367 | 0.215874319 | Inverse variance weighted (multiplicative random effects) | FXYD5 | 0.778234308 |
| -0.454534033 | 0.9912074 | 0.646545842 | Inverse variance weighted (multiplicative random effects) | GAL | 0.998066246 |
| 0.148622559 | 0.067962413 | 0.028754597 | Inverse variance weighted (multiplicative random effects) | GALNT10 | 0.253827031 |
| -0.083130806 | 0.108644145 | 0.444172704 | Inverse variance weighted (multiplicative random effects) | GALNT2 | 0.934538753 |
| -0.076316653 | 0.111073918 | 0.492032399 | Inverse variance weighted (multiplicative random effects) | GALNT3 | 0.947617954 |
| -0.112255885 | 0.021280023 | 1.33E-07 | Inverse variance weighted (multiplicative random effects) | GALNT5 | 5.21E-06 |
| -0.25831711 | 0.72207265 | 0.720534952 | Inverse variance weighted (multiplicative random effects) | GALNT7 | 1 |
| -0.221778659 | 0.285250428 | 0.436871171 | Wald ratio | GART | 0.933907539 |
| -0.600991432 | 0.269888092 | 0.025959497 | Wald ratio | GAS2 | 0.23682348 |
| 0.031325286 | 0.03667127 | 0.392983797 | Inverse variance weighted (multiplicative random effects) | GAS6 | 0.915857498 |
| -0.000865487 | 0.054557471 | 0.987343082 | Inverse variance weighted (multiplicative random effects) | GASK1A | 1 |
| -0.024639514 | 0.049462068 | 0.618378537 | Inverse variance weighted (multiplicative random effects) | GATD3 | 0.99638775 |
| -0.158432234 | 0.005772661 | 7.91E-166 | Inverse variance weighted (multiplicative random effects) | GBP1 | 1.83E-163 |
| -0.089297624 | 0.26338259 | 0.73457848 | Wald ratio | GBP2 | 1 |
| -0.005576924 | 0.07593587 | 0.941453963 | Inverse variance weighted (multiplicative random effects) | GBP4 | 1 |
| -0.002044051 | 0.027414064 | 0.940563111 | Inverse variance weighted (multiplicative random effects) | GC | 1 |
| 1.068193506 | 0.470345745 | 0.023142047 | Wald ratio | GCLM | 0.221822382 |
| 0.161781822 | 0.093779863 | 0.084505266 | Inverse variance weighted (multiplicative random effects) | GCNT1 | 0.516101454 |
| 0.337156276 | 0.050829188 | 3.29E-11 | Inverse variance weighted (multiplicative random effects) | GDF15 | 1.52E-09 |
| 0.19664639 | 0.125578434 | 0.117366212 | Inverse variance weighted (multiplicative random effects) | GDNF | 0.607267961 |
| -0.216883635 | 0.292117035 | 0.457811953 | Inverse variance weighted (multiplicative random effects) | GFAP | 0.940832265 |
| -0.170488606 | 0.37284859 | 0.647484448 | Wald ratio | GFER | 0.998066246 |
| 0.047836504 | 0.123697009 | 0.698961137 | Inverse variance weighted (multiplicative random effects) | GFRA1 | 1 |
| -0.033587844 | 0.041362123 | 0.416766657 | Inverse variance weighted (multiplicative random effects) | GFRA2 | 0.923975972 |
| -0.091829905 | 0.674217703 | 0.89166147 | Wald ratio | GFRA3 | 1 |
| 0.002977133 | 0.051008601 | 0.953457637 | Inverse variance weighted (multiplicative random effects) | GFRAL | 1 |
| 0.017070276 | 0.087091941 | 0.844607864 | Inverse variance weighted (multiplicative random effects) | GGACT | 1 |
| 0.386953165 | 0.259932194 | 0.136574384 | Wald ratio | GGCT | 0.653045329 |
| 0.01158431 | 0.062636364 | 0.853271827 | Inverse variance weighted (multiplicative random effects) | GGH | 1 |
| -0.003806349 | 0.108254045 | 0.97195115 | Inverse variance weighted (multiplicative random effects) | GGT1 | 1 |
| 0.005272423 | 0.044858315 | 0.906436096 | Inverse variance weighted (multiplicative random effects) | GGT5 | 1 |
| -0.124874727 | 0.055834564 | 0.025318188 | Inverse variance weighted (multiplicative random effects) | GHR | 0.233016954 |
| -0.026473507 | 0.037926126 | 0.485159542 | Inverse variance weighted (multiplicative random effects) | GHRL | 0.943195135 |
| 0.021827708 | 0.042600897 | 0.608387384 | Inverse variance weighted (multiplicative random effects) | GIMAP7 | 0.992506477 |
| 0.01574765 | 0.108061815 | 0.884135989 | Inverse variance weighted (multiplicative random effects) | GIMAP8 | 1 |
| 1.51897112 | 0.637846008 | 0.017246632 | Wald ratio | GIP | 0.181176745 |
| 0.114004231 | 0.169908834 | 0.502237764 | Inverse variance weighted (multiplicative random effects) | GIPC2 | 0.956220371 |
| 0.0348475 | 0.122124756 | 0.775381023 | Inverse variance weighted (multiplicative random effects) | GIPC3 | 1 |
| -1.148859333 | 0.758905025 | 0.13006691 | Wald ratio | GIT1 | 0.642802656 |
| -0.077554462 | 0.039187662 | 0.047810032 | Inverse variance weighted (multiplicative random effects) | GLB1 | 0.382480255 |
| -0.120140477 | 0.059867748 | 0.044774735 | Inverse variance weighted (multiplicative random effects) | GLO1 | 0.360974611 |
| 0.003417922 | 0.053546223 | 0.949104608 | Inverse variance weighted (multiplicative random effects) | GLRX | 1 |
| -0.328264492 | 0.319360486 | 0.304005931 | Inverse variance weighted (multiplicative random effects) | GLRX5 | 0.869694804 |
| -0.047829552 | 0.07786962 | 0.539065935 | Inverse variance weighted (multiplicative random effects) | GLT8D2 | 0.972469337 |
| -0.055907283 | 0.059271883 | 0.345560984 | Inverse variance weighted (multiplicative random effects) | GM2A | 0.897122605 |
| 0.306532133 | 0.437527121 | 0.483551777 | Wald ratio | GMFG | 0.942682873 |
| -0.179033162 | 0.400038577 | 0.654485399 | Inverse variance weighted (multiplicative random effects) | GMPR | 0.998066246 |
| 0.159030831 | 0.109142281 | 0.145089756 | Inverse variance weighted (multiplicative random effects) | GMPR2 | 0.667755944 |
| -0.276542856 | 0.384593855 | 0.472109081 | Wald ratio | GNAS | 0.940832265 |
| 0.030687869 | 0.081048886 | 0.704959627 | Inverse variance weighted (multiplicative random effects) | GNLY | 1 |
| -0.197407317 | 0.185600469 | 0.287503453 | Inverse variance weighted (multiplicative random effects) | GNPDA1 | 0.853474736 |
| -0.025165474 | 0.094008554 | 0.788935296 | Inverse variance weighted (multiplicative random effects) | GNPDA2 | 1 |
| 0.127434265 | 0.33891246 | 0.706909919 | Inverse variance weighted (multiplicative random effects) | GOLGA3 | 1 |
| 0.019430707 | 0.23957029 | 0.93535726 | Wald ratio | GOLM2 | 1 |
| -0.8594679 | 0.570671791 | 0.132050898 | Wald ratio | GORASP2 | 0.645000048 |
| -0.195731782 | 0.18651193 | 0.293978858 | Inverse variance weighted (multiplicative random effects) | GP1BA | 0.864306126 |
| 0.103150722 | 0.08485952 | 0.224157593 | Inverse variance weighted (multiplicative random effects) | GP1BB | 0.786751592 |
| -0.12019819 | 0.123060553 | 0.328697785 | Wald ratio | GP2 | 0.879911704 |
| 0.033721439 | 0.244352475 | 0.890237838 | Wald ratio | GP5 | 1 |
| 0.029530825 | 0.090595071 | 0.744450816 | Inverse variance weighted (multiplicative random effects) | GP6 | 1 |
| 0.018757898 | 0.275578293 | 0.94573198 | Inverse variance weighted (multiplicative random effects) | GPA33 | 1 |
| -0.154715029 | 0.04947242 | 0.001764206 | Inverse variance weighted (multiplicative random effects) | GPC1 | 0.030326855 |
| 0.045658326 | 0.045451604 | 0.315114461 | Inverse variance weighted (multiplicative random effects) | GPC5 | 0.873862934 |
| 0.074184701 | 0.268138327 | 0.782036744 | Inverse variance weighted (multiplicative random effects) | GPD1 | 1 |
| 0.176776181 | 0.18042989 | 0.327209549 | Inverse variance weighted (multiplicative random effects) | GPHA2 | 0.878188209 |
| 0.044913709 | 0.167288354 | 0.78832926 | Inverse variance weighted (multiplicative random effects) | GPIHBP1 | 1 |
| -0.050340794 | 0.06971209 | 0.47021806 | Inverse variance weighted (multiplicative random effects) | GPNMB | 0.940832265 |
| 0.100800659 | 0.454722146 | 0.824566675 | Wald ratio | GPR158 | 1 |
| -0.051232963 | 0.1087549 | 0.63757898 | Inverse variance weighted (multiplicative random effects) | GPR37 | 0.998066246 |
| -0.217264721 | 0.252251839 | 0.389072372 | Wald ratio | GPRC5C | 0.91224413 |
| 0.169564111 | 0.327242927 | 0.604346821 | Wald ratio | GRAP2 | 0.991807207 |
| 0.128222361 | 0.107855858 | 0.234506274 | Inverse variance weighted (multiplicative random effects) | GRHPR | 0.793187763 |
| -0.070300898 | 0.153011552 | 0.645912266 | Inverse variance weighted (multiplicative random effects) | GRIK2 | 0.998066246 |
| -0.106934819 | 0.19423549 | 0.581947596 | Inverse variance weighted (multiplicative random effects) | GRK5 | 0.989304274 |
| -0.060507943 | 0.084179148 | 0.472264369 | Inverse variance weighted (multiplicative random effects) | GRN | 0.940832265 |
| 0.038072707 | 0.049640717 | 0.443102156 | Inverse variance weighted (multiplicative random effects) | GRP | 0.934538753 |
| 0.027415787 | 0.536009757 | 0.959207648 | Wald ratio | GRPEL1 | 1 |
| -0.425993286 | 0.15142325 | 0.004904166 | Inverse variance weighted (multiplicative random effects) | GSN | 0.070349421 |
| -0.297904565 | 0.291726503 | 0.307170311 | Wald ratio | GSR | 0.869694804 |
| -0.321630996 | 0.481667904 | 0.504296829 | Wald ratio | GSTA1 | 0.956220371 |
| -0.325000989 | 0.486714737 | 0.504296829 | Wald ratio | GSTA3 | 0.956220371 |
| 0.196168592 | 0.193029834 | 0.30950536 | Inverse variance weighted (multiplicative random effects) | GSTM4 | 0.869694804 |
| -0.011166836 | 0.292387749 | 0.9695347 | Inverse variance weighted (multiplicative random effects) | GSTP1 | 1 |
| -0.030861355 | 0.051222567 | 0.5468445 | Inverse variance weighted (multiplicative random effects) | GSTT2B | 0.975364036 |
| -0.245966492 | 0.049235341 | 5.86E-07 | Inverse variance weighted (multiplicative random effects) | GUCA2A | 2.26E-05 |
| -0.117450342 | 0.006849382 | 6.55E-66 | Inverse variance weighted (multiplicative random effects) | GUSB | 9.73E-64 |
| -0.750608325 | 0.29010104 | 0.00967024 | Inverse variance weighted (multiplicative random effects) | GZMA | 0.117626306 |
| -0.058728973 | 0.12162581 | 0.629190854 | Inverse variance weighted (multiplicative random effects) | GZMB | 0.998066246 |
| 0.371926328 | 0.253371221 | 0.142128479 | Inverse variance weighted (multiplicative random effects) | GZMH | 0.660737897 |
| 0.632368379 | 0.68820492 | 0.358165455 | Wald ratio | HADH | 0.900699853 |
| 0.059011762 | 0.199315717 | 0.767175251 | Inverse variance weighted (multiplicative random effects) | HAGH | 1 |
| -0.021101684 | 0.053397157 | 0.692707352 | Inverse variance weighted (multiplicative random effects) | HAVCR1 | 1 |
| -0.019150838 | 0.033039236 | 0.562157925 | Inverse variance weighted (multiplicative random effects) | HAVCR2 | 0.983421769 |
| 0.67711777 | 0.577912116 | 0.241332713 | Wald ratio | HBEGF | 0.793187763 |
| -0.054287878 | 0.038566507 | 0.159236767 | Inverse variance weighted (multiplicative random effects) | HBQ1 | 0.698760494 |
| -0.060167483 | 0.037856594 | 0.111980769 | Inverse variance weighted (multiplicative random effects) | HBZ | 0.594296188 |
| 0.043357924 | 0.034503063 | 0.208884079 | Inverse variance weighted (multiplicative random effects) | HCG22 | 0.769129582 |
| -0.161434272 | 0.367961891 | 0.660860434 | Wald ratio | HCLS1 | 0.998066246 |
| -0.05096103 | 0.03044039 | 0.094105957 | Inverse variance weighted (multiplicative random effects) | HDDC2 | 0.549832558 |
| -0.067004526 | 0.025187955 | 0.007809861 | Inverse variance weighted (multiplicative random effects) | HDGF | 0.097858503 |
| 0.208769785 | 0.299521597 | 0.485796041 | Wald ratio | HEBP1 | 0.943195135 |
| 0.038814728 | 0.435581121 | 0.928994318 | Inverse variance weighted (multiplicative random effects) | HEG1 | 1 |
| -2.113186573 | 1.634610198 | 0.196088175 | Wald ratio | HEPACAM2 | 0.758110417 |
| -0.284474251 | 0.537296432 | 0.596489894 | Inverse variance weighted (multiplicative random effects) | HGF | 0.989304274 |
| -0.027734671 | 0.066492095 | 0.676596264 | Inverse variance weighted (multiplicative random effects) | HGFAC | 1 |
| -0.235030668 | 0.676673728 | 0.728341659 | Wald ratio | HIP1 | 1 |
| 0.002269857 | 0.16546951 | 0.989055223 | Inverse variance weighted (multiplicative random effects) | HIP1R | 1 |
| -0.020654383 | 0.04785968 | 0.666060342 | Inverse variance weighted (multiplicative random effects) | HLA-A | 0.999439337 |
| 0.023647336 | 0.060335865 | 0.695111157 | Inverse variance weighted (multiplicative random effects) | HLA-DRA | 1 |
| -0.015593616 | 0.06829141 | 0.819382439 | Inverse variance weighted (multiplicative random effects) | HLA-E | 1 |
| -0.127647675 | 0.112888423 | 0.258163719 | Inverse variance weighted (multiplicative random effects) | HMBS | 0.815451469 |
| 0.008949508 | 0.037308709 | 0.810425469 | Inverse variance weighted (multiplicative random effects) | HMCN2 | 1 |
| -0.125877973 | 0.432771827 | 0.771154933 | Wald ratio | HMOX1 | 1 |
| -0.077417235 | 0.272123088 | 0.776032256 | Wald ratio | HMOX2 | 1 |
| -0.026433004 | 0.05438468 | 0.626940448 | Inverse variance weighted (multiplicative random effects) | HNMT | 0.998066246 |
| -0.015930028 | 0.173134098 | 0.92669028 | Inverse variance weighted (multiplicative random effects) | HPCAL1 | 1 |
| 0.013613951 | 0.046071094 | 0.767612911 | Inverse variance weighted (multiplicative random effects) | HPGDS | 1 |
| 0.086725981 | 0.089863916 | 0.334504115 | Inverse variance weighted (multiplicative random effects) | HPSE | 0.888593306 |
| -0.28351852 | 0.301291013 | 0.346698605 | Inverse variance weighted (multiplicative random effects) | HRC | 0.897122605 |
| 0.024536494 | 0.059723807 | 0.681195201 | Inverse variance weighted (multiplicative random effects) | HRG | 1 |
| 0.006100348 | 0.125021038 | 0.961083011 | Inverse variance weighted (multiplicative random effects) | HS1BP3 | 1 |
| 0.015041538 | 0.065757044 | 0.819067795 | Inverse variance weighted (multiplicative random effects) | HS3ST3B1 | 1 |
| 0.098960021 | 0.024124045 | 4.09E-05 | Inverse variance weighted (multiplicative random effects) | HS6ST1 | 0.001120354 |
| -0.079323681 | 0.024491171 | 0.001200049 | Inverse variance weighted (multiplicative random effects) | HSBP1 | 0.022900019 |
| 0.112228309 | 0.040679658 | 0.005800849 | Inverse variance weighted (multiplicative random effects) | HSD17B14 | 0.079380045 |
| -0.088730952 | 0.094336739 | 0.346921755 | Inverse variance weighted (multiplicative random effects) | HSDL2 | 0.897122605 |
| -0.335864679 | 0.477506261 | 0.481823762 | Wald ratio | HSPA1A | 0.94102669 |
| 0.183099804 | 0.337859421 | 0.58785929 | Inverse variance weighted (multiplicative random effects) | HSPA2 | 0.989304274 |
| 0.099625658 | 0.062274811 | 0.109648607 | Inverse variance weighted (multiplicative random effects) | HSPB1 | 0.590852599 |
| 0.296719099 | 0.726750146 | 0.683066556 | Wald ratio | HSPB6 | 1 |
| -0.163640828 | 0.099699987 | 0.100728415 | Inverse variance weighted (multiplicative random effects) | HSPG2 | 0.562880046 |
| -0.058579733 | 0.038711502 | 0.130219102 | Inverse variance weighted (multiplicative random effects) | HYAL1 | 0.642802656 |
| 0.109727083 | 0.170534712 | 0.519945374 | Wald ratio | HYOU1 | 0.964751452 |
| 0.039119272 | 0.035733826 | 0.273630277 | Inverse variance weighted (multiplicative random effects) | ICAM1 | 0.84116049 |
| 0.284544226 | 0.514562019 | 0.580274816 | Wald ratio | ICAM2 | 0.989304274 |
| 0.044341514 | 0.081690402 | 0.587267824 | Inverse variance weighted (multiplicative random effects) | ICAM3 | 0.989304274 |
| -0.011619546 | 0.38152533 | 0.97570378 | Inverse variance weighted (multiplicative random effects) | ICAM4 | 1 |
| 0.042598456 | 0.058824474 | 0.468966211 | Inverse variance weighted (multiplicative random effects) | ICAM5 | 0.940832265 |
| -0.173987609 | 0.117665126 | 0.139229176 | Inverse variance weighted (multiplicative random effects) | ICOSLG | 0.657666752 |
| 0.010130598 | 0.113692147 | 0.928998052 | Inverse variance weighted (multiplicative random effects) | IDI2 | 1 |
| 0.108742782 | 0.137973911 | 0.430614718 | Inverse variance weighted (multiplicative random effects) | IDO1 | 0.931458519 |
| 0.178673944 | 0.230358507 | 0.437964983 | Inverse variance weighted (multiplicative random effects) | IDO1 | 0.934538753 |
| 0.112931483 | 0.141406891 | 0.424506225 | Inverse variance weighted (multiplicative random effects) | IDO1 | 0.92774274 |
| 0.159356233 | 0.220894758 | 0.470655775 | Inverse variance weighted (multiplicative random effects) | IDO1 | 0.940832265 |
| 0.07093949 | 0.053838721 | 0.187627688 | Inverse variance weighted (multiplicative random effects) | IDUA | 0.745928931 |
| 0.046629627 | 0.077189703 | 0.545782949 | Inverse variance weighted (multiplicative random effects) | IFI30 | 0.975364036 |
| 0.548667743 | 0.319696564 | 0.086122869 | Wald ratio | IFIT3 | 0.516101454 |
| 0.031050377 | 0.079424839 | 0.695841304 | Inverse variance weighted (multiplicative random effects) | IFNAR1 | 1 |
| -1.27418683 | 0.697694679 | 0.067807882 | Wald ratio | IFNG | 0.466783731 |
| -0.099185159 | 0.113935782 | 0.384007747 | Inverse variance weighted (multiplicative random effects) | IFNGR1 | 0.91151664 |
| 0.013976227 | 0.040317718 | 0.728852606 | Inverse variance weighted (multiplicative random effects) | IFNGR2 | 1 |
| -0.473466451 | 0.667448391 | 0.478096214 | Wald ratio | IFNL1 | 0.940832265 |
| 0.053694829 | 0.101381671 | 0.596368116 | Inverse variance weighted (multiplicative random effects) | IFNLR1 | 0.989304274 |
| 0.028309349 | 0.092144423 | 0.758669818 | Inverse variance weighted (multiplicative random effects) | IGDCC4 | 1 |
| -0.022416002 | 0.229958438 | 0.922346412 | Inverse variance weighted (multiplicative random effects) | IGF1R | 1 |
| -0.111878173 | 0.076242418 | 0.142267101 | Inverse variance weighted (multiplicative random effects) | IGF2R | 0.660737897 |
| 0.076523301 | 0.64182461 | 0.905094927 | Wald ratio | IGFBP1 | 1 |
| 1.079170664 | 0.441748321 | 0.014567609 | Wald ratio | IGFBP2 | 0.160914794 |
| 0.053794928 | 0.064770513 | 0.406229821 | Inverse variance weighted (multiplicative random effects) | IGFBP3 | 0.922595089 |
| 0.027376115 | 0.679077632 | 0.967843052 | Wald ratio | IGFBP4 | 1 |
| 0.192366511 | 0.325914542 | 0.555032408 | Inverse variance weighted (multiplicative random effects) | IGFBP6 | 0.977576895 |
| -0.131599069 | 0.099071384 | 0.184070523 | Inverse variance weighted (multiplicative random effects) | IGFBP7 | 0.741887316 |
| -0.016189814 | 0.072832983 | 0.824090592 | Inverse variance weighted (multiplicative random effects) | IGFBPL1 | 1 |
| 0.111429012 | 0.364972928 | 0.760131882 | Wald ratio | IGHMBP2 | 1 |
| -0.050620975 | 0.016893655 | 0.00273144 | Inverse variance weighted (multiplicative random effects) | IGLC2 | 0.04271726 |
| 0.097983754 | 0.075153374 | 0.1923073 | Inverse variance weighted (multiplicative random effects) | IGSF21 | 0.748540623 |
| 0.088595379 | 0.244825007 | 0.71744749 | Inverse variance weighted (multiplicative random effects) | IGSF3 | 1 |
| -0.175137786 | 0.20737993 | 0.398375425 | Wald ratio | IGSF8 | 0.917383701 |
| -0.019772783 | 0.154106499 | 0.897906846 | Inverse variance weighted (multiplicative random effects) | IGSF9 | 1 |
| 0.354817675 | 0.280343175 | 0.20563671 | Wald ratio | IL10 | 0.769129582 |
| 0.378984429 | 0.188426982 | 0.044293084 | Wald ratio | IL10RA | 0.358480992 |
| 0.02664136 | 0.050554501 | 0.598205428 | Inverse variance weighted (multiplicative random effects) | IL10RB | 0.989304274 |
| 0.014749089 | 0.068617666 | 0.829809459 | Inverse variance weighted (multiplicative random effects) | IL12B | 1 |
| -0.010459675 | 0.070775002 | 0.882510313 | Inverse variance weighted (multiplicative random effects) | IL12B | 1 |
| -0.114807309 | 0.104805639 | 0.273328014 | Inverse variance weighted (multiplicative random effects) | IL12RB1 | 0.84116049 |
| -2.581193376 | 2.632880534 | 0.326904209 | Wald ratio | IL15 | 0.878188209 |
| -0.071414478 | 0.053494315 | 0.181878991 | Inverse variance weighted (multiplicative random effects) | IL15RA | 0.741106653 |
| -0.072713601 | 0.054074996 | 0.178728381 | Inverse variance weighted (multiplicative random effects) | IL16 | 0.738570212 |
| -0.014638557 | 0.237526436 | 0.950858146 | Wald ratio | IL17C | 1 |
| -0.042860254 | 0.077726536 | 0.581343256 | Inverse variance weighted (multiplicative random effects) | IL17D | 0.989304274 |
| -0.052954456 | 0.066047797 | 0.422692026 | Inverse variance weighted (multiplicative random effects) | IL17F | 0.92774274 |
| 0.030060047 | 0.058136146 | 0.605112218 | Inverse variance weighted (multiplicative random effects) | IL17RA | 0.991807207 |
| -0.049608919 | 0.056874878 | 0.383073864 | Inverse variance weighted (multiplicative random effects) | IL17RB | 0.91151664 |
| -0.019185604 | 0.054935209 | 0.72690871 | Inverse variance weighted (multiplicative random effects) | IL18 | 1 |
| 0.250458921 | 0.150642388 | 0.096391352 | Inverse variance weighted (multiplicative random effects) | IL18BP | 0.554700584 |
| -0.025194611 | 0.057550829 | 0.661545655 | Inverse variance weighted (multiplicative random effects) | IL18R1 | 0.998066246 |
| -0.023593563 | 0.044632672 | 0.597071575 | Inverse variance weighted (multiplicative random effects) | IL19 | 0.989304274 |
| -0.132171769 | 0.150828184 | 0.38086325 | Inverse variance weighted (multiplicative random effects) | IL1R1 | 0.91151664 |
| -0.131836773 | 0.080039615 | 0.099528446 | Inverse variance weighted (multiplicative random effects) | IL1R2 | 0.562880046 |
| -0.051510713 | 0.041643233 | 0.21610461 | Inverse variance weighted (multiplicative random effects) | IL1RAP | 0.778234308 |
| 0.057206655 | 0.048204875 | 0.235330183 | Inverse variance weighted (multiplicative random effects) | IL1RL1 | 0.793187763 |
| -0.054671414 | 0.04302534 | 0.203842724 | Inverse variance weighted (multiplicative random effects) | IL1RL2 | 0.767524355 |
| -0.034111117 | 0.182651728 | 0.851852758 | Inverse variance weighted (multiplicative random effects) | IL1RN | 1 |
| -0.018639699 | 0.780499596 | 0.980946928 | Wald ratio | IL20RA | 1 |
| -0.096607809 | 0.249419543 | 0.69851151 | Wald ratio | IL20RB | 1 |
| -0.168794829 | 0.346355671 | 0.626013609 | Inverse variance weighted (multiplicative random effects) | IL22 | 0.998066246 |
| 0.006365129 | 0.092784007 | 0.945306769 | Inverse variance weighted (multiplicative random effects) | IL22RA1 | 1 |
| -0.361927986 | 0.405182105 | 0.371724536 | Wald ratio | IL27 | 0.909083377 |
| -0.078844922 | 0.062798886 | 0.209291992 | Inverse variance weighted (multiplicative random effects) | IL2RA | 0.769129582 |
| -0.017073616 | 0.05521131 | 0.757138002 | Inverse variance weighted (multiplicative random effects) | IL31RA | 1 |
| 0.269393562 | 0.087703757 | 0.002128928 | Inverse variance weighted (multiplicative random effects) | IL32 | 0.03542537 |
| 0.288126334 | 0.935998268 | 0.758213329 | Wald ratio | IL33 | 1 |
| -0.070714222 | 0.040577835 | 0.081389353 | Inverse variance weighted (multiplicative random effects) | IL34 | 0.512778355 |
| 0.194332437 | 0.558678966 | 0.72795827 | Wald ratio | IL36A | 1 |
| 0.204002176 | 0.499265596 | 0.682829917 | Wald ratio | IL36G | 1 |
| 0.024628257 | 0.067995111 | 0.717197877 | Inverse variance weighted (multiplicative random effects) | IL4R | 1 |
| 0.077080011 | 0.162774046 | 0.635828018 | Inverse variance weighted (multiplicative random effects) | IL5RA | 0.998066246 |
| 0.015949892 | 0.040181875 | 0.691409888 | Inverse variance weighted (multiplicative random effects) | IL6R | 1 |
| -0.111807452 | 0.168137922 | 0.506066719 | Wald ratio | IL6ST | 0.956220371 |
| 0.234382846 | 0.199537198 | 0.240141838 | Inverse variance weighted (multiplicative random effects) | IL7 | 0.793187763 |
| 0.129912946 | 0.046254442 | 0.004974834 | Inverse variance weighted (multiplicative random effects) | IL7R | 0.070392204 |
| 0.324402836 | 0.248562727 | 0.19185384 | Wald ratio | IMMT | 0.748540623 |
| -0.128420053 | 0.086383724 | 0.137114342 | Inverse variance weighted (multiplicative random effects) | IMPA1 | 0.653345785 |
| -0.619209837 | 0.540841214 | 0.252250024 | Wald ratio | ING1 | 0.805960139 |
| -0.081974441 | 0.069964801 | 0.241336557 | Inverse variance weighted (multiplicative random effects) | INHBB | 0.793187763 |
| 0.011281656 | 0.075809602 | 0.881699089 | Inverse variance weighted (multiplicative random effects) | INHBC | 1 |
| 0.068585421 | 0.103469874 | 0.507424059 | Inverse variance weighted (multiplicative random effects) | INPP1 | 0.956220371 |
| -0.043969769 | 0.19226219 | 0.819104495 | Wald ratio | INPP5D | 1 |
| 0.099797403 | 0.011587395 | 7.14E-18 | Inverse variance weighted (multiplicative random effects) | INSL4 | 4.50E-16 |
| -0.023505924 | 0.107683286 | 0.827205049 | Inverse variance weighted (multiplicative random effects) | INSL5 | 1 |
| 0.775578905 | 0.569029431 | 0.172887057 | Wald ratio | INSR | 0.72933915 |
| -0.020775997 | 0.40485795 | 0.959073116 | Inverse variance weighted (multiplicative random effects) | IPCEF1 | 1 |
| 0.59895427 | 0.199345727 | 0.002659299 | Wald ratio | IQGAP2 | 0.041904104 |
| -0.067088249 | 0.231477351 | 0.771949045 | Inverse variance weighted (multiplicative random effects) | IRAK4 | 1 |
| -0.114083498 | 0.145372647 | 0.432590831 | Inverse variance weighted (multiplicative random effects) | ISLR2 | 0.931458519 |
| 0.068270324 | 0.083162282 | 0.411686748 | Inverse variance weighted (multiplicative random effects) | ISM1 | 0.923405266 |
| -0.40043425 | 0.271311795 | 0.139965621 | Wald ratio | IST1 | 0.657666752 |
| -0.225298238 | 0.082477488 | 0.006302128 | Inverse variance weighted (multiplicative random effects) | ITGA11 | 0.082964721 |
| 0.05064181 | 0.066631178 | 0.447235613 | Inverse variance weighted (multiplicative random effects) | ITGA2 | 0.935865266 |
| 0.182465092 | 0.483668606 | 0.70598612 | Wald ratio | ITGA5 | 1 |
| -0.133406482 | 0.143845792 | 0.35370484 | Inverse variance weighted (multiplicative random effects) | ITGA6 | 0.900497022 |
| -0.066022871 | 0.089662554 | 0.461518746 | Inverse variance weighted (multiplicative random effects) | ITGAM | 0.940832265 |
| 0.087321786 | 0.340208623 | 0.797432537 | Inverse variance weighted (multiplicative random effects) | ITGAV | 1 |
| -0.13639031 | 0.237083216 | 0.565098872 | Wald ratio | ITGB2 | 0.986078568 |
| 0.412312568 | 0.150853785 | 0.006272349 | Inverse variance weighted (multiplicative random effects) | ITGB5 | 0.082964721 |
| -0.144679347 | 0.157344661 | 0.357831013 | Inverse variance weighted (multiplicative random effects) | ITGB6 | 0.900699853 |
| 0.054729963 | 0.109075781 | 0.61583571 | Inverse variance weighted (multiplicative random effects) | ITGB7 | 0.994517296 |
| -0.210972559 | 0.090943596 | 0.020350745 | Inverse variance weighted (multiplicative random effects) | ITGBL1 | 0.204490576 |
| -0.107698341 | 0.209550906 | 0.607288106 | Wald ratio | ITIH1 | 0.992207403 |
| 0.085371727 | 0.087709253 | 0.330379804 | Inverse variance weighted (multiplicative random effects) | ITIH3 | 0.881012811 |
| 0.079338945 | 0.02925805 | 0.00669399 | Inverse variance weighted (multiplicative random effects) | ITIH4 | 0.085947528 |
| 0.112874326 | 0.244127511 | 0.643824559 | Wald ratio | ITIH5 | 0.998066246 |
| -0.029743261 | 0.040879705 | 0.466870652 | Inverse variance weighted (multiplicative random effects) | ITPA | 0.940832265 |
| 0.079390909 | 0.401965466 | 0.843430969 | Wald ratio | ITPR1 | 1 |
| -0.504538062 | 0.248478142 | 0.042304435 | Inverse variance weighted (multiplicative random effects) | JAM2 | 0.349179466 |
| 0.232860597 | 0.027937243 | 7.74E-17 | Inverse variance weighted (multiplicative random effects) | JAM3 | 4.74E-15 |
| -0.080242706 | 0.635151419 | 0.899465691 | Wald ratio | JPT2 | 1 |
| 0.049494856 | 0.025348393 | 0.050868963 | Inverse variance weighted (multiplicative random effects) | KAZALD1 | 0.396282559 |
| -0.022052969 | 0.076139236 | 0.772091655 | Inverse variance weighted (multiplicative random effects) | KDR | 1 |
| 0.07034872 | 0.027095251 | 0.009422039 | Inverse variance weighted (multiplicative random effects) | KEL | 0.115281421 |
| 0.008251149 | 0.095114327 | 0.930870389 | Inverse variance weighted (multiplicative random effects) | KHK | 1 |
| -0.057044734 | 0.107106349 | 0.594311197 | Inverse variance weighted (multiplicative random effects) | KIAA0319 | 0.989304274 |
| 0.287365044 | 0.870502842 | 0.741313939 | Wald ratio | KIF1C | 1 |
| 0.388985984 | 0.632898568 | 0.538812105 | Wald ratio | KIFBP | 0.972469337 |
| -0.175256842 | 0.057638592 | 0.002360945 | Inverse variance weighted (multiplicative random effects) | KIR2DL2 | 0.038667445 |
| 0.015211043 | 0.06689248 | 0.820116286 | Inverse variance weighted (multiplicative random effects) | KIR2DL3 | 1 |
| -0.037172241 | 0.045201997 | 0.410872959 | Inverse variance weighted (multiplicative random effects) | KIR2DS4 | 0.923405266 |
| 0.044218431 | 0.04105476 | 0.281453561 | Inverse variance weighted (multiplicative random effects) | KIR3DL1 | 0.847831902 |
| -0.028839177 | 0.603856374 | 0.961908839 | Wald ratio | KIR3DL2 | 1 |
| 0.001672977 | 0.101613528 | 0.986864132 | Inverse variance weighted (multiplicative random effects) | KIRREL2 | 1 |
| -0.522647011 | 0.423371051 | 0.21702065 | Inverse variance weighted (multiplicative random effects) | KIT | 0.778234308 |
| -0.265825819 | 0.558265064 | 0.633957426 | Wald ratio | KITLG | 0.998066246 |
| 0.05080512 | 0.038829542 | 0.190732895 | Inverse variance weighted (multiplicative random effects) | KLB | 0.748536643 |
| 0.071011076 | 0.050690036 | 0.161247495 | Inverse variance weighted (multiplicative random effects) | KLK1 | 0.700812975 |
| 0.06466683 | 0.040851521 | 0.113427753 | Inverse variance weighted (multiplicative random effects) | KLK10 | 0.594296188 |
| 0.04835592 | 0.056765386 | 0.394294165 | Inverse variance weighted (multiplicative random effects) | KLK11 | 0.916038616 |
| -0.028451735 | 0.056958415 | 0.617414742 | Inverse variance weighted (multiplicative random effects) | KLK12 | 0.996293765 |
| -0.120524813 | 0.036354391 | 0.000915529 | Inverse variance weighted (multiplicative random effects) | KLK13 | 0.018310588 |
| -0.024393595 | 0.084040727 | 0.771617945 | Inverse variance weighted (multiplicative random effects) | KLK14 | 1 |
| -0.002306717 | 0.043288695 | 0.957503387 | Inverse variance weighted (multiplicative random effects) | KLK15 | 1 |
| 1.25884425 | 0.539163176 | 0.019553283 | Wald ratio | KLK3 | 0.197431208 |
| 0.031033288 | 0.040573841 | 0.444355205 | Inverse variance weighted (multiplicative random effects) | KLK4 | 0.934538753 |
| -0.166752569 | 0.28228318 | 0.554702707 | Inverse variance weighted (multiplicative random effects) | KLK6 | 0.977576895 |
| 0.001037141 | 0.062499967 | 0.986760301 | Inverse variance weighted (multiplicative random effects) | KLK7 | 1 |
| -0.153048182 | 0.074022273 | 0.038677993 | Inverse variance weighted (multiplicative random effects) | KLK8 | 0.323093279 |
| -0.023925349 | 0.083591268 | 0.774710942 | Inverse variance weighted (multiplicative random effects) | KLKB1 | 1 |
| -0.193934562 | 0.187628375 | 0.30131853 | Inverse variance weighted (multiplicative random effects) | KLRB1 | 0.869694804 |
| 0.10659996 | 0.067197439 | 0.112655544 | Inverse variance weighted (multiplicative random effects) | KLRD1 | 0.594296188 |
| 0.134156566 | 0.155359158 | 0.387848708 | Inverse variance weighted (multiplicative random effects) | KLRF1 | 0.91224413 |
| -0.066091661 | 0.076422712 | 0.387139241 | Inverse variance weighted (multiplicative random effects) | KLRK1 | 0.91224413 |
| 0.376902072 | 0.616798379 | 0.541158513 | Wald ratio | KRT18 | 0.972958495 |
| 0.038798611 | 0.202419229 | 0.847997158 | Wald ratio | KRT19 | 1 |
| -0.459053682 | 0.291426769 | 0.115211684 | Wald ratio | KRT5 | 0.600602264 |
| 0.04219997 | 0.156345837 | 0.787226223 | Inverse variance weighted (multiplicative random effects) | KYAT1 | 1 |
| -0.103713248 | 0.081173633 | 0.201365233 | Inverse variance weighted (multiplicative random effects) | KYNU | 0.767524355 |
| -0.052793945 | 0.453249736 | 0.907273158 | Wald ratio | LACRT | 1 |
| 0.007651902 | 0.183398002 | 0.966719574 | Inverse variance weighted (multiplicative random effects) | LACTB2 | 1 |
| 0.053222477 | 0.135150625 | 0.693727825 | Inverse variance weighted (multiplicative random effects) | LAG3 | 1 |
| -0.042894136 | 0.081764745 | 0.599858305 | Inverse variance weighted (multiplicative random effects) | LAIR1 | 0.989304274 |
| 0.030075521 | 0.056183692 | 0.592437618 | Inverse variance weighted (multiplicative random effects) | LAIR2 | 0.989304274 |
| 0.040738836 | 0.022501991 | 0.070225297 | Inverse variance weighted (multiplicative random effects) | LAMA4 | 0.474165438 |
| -0.083403757 | 0.059441071 | 0.160577037 | Inverse variance weighted (multiplicative random effects) | LAMB1 | 0.700551705 |
| 0.161188448 | 0.455479693 | 0.723423396 | Wald ratio | LAMP1 | 1 |
| 0.149593699 | 0.094502511 | 0.113430571 | Inverse variance weighted (multiplicative random effects) | LAMP3 | 0.594296188 |
| -0.799617094 | 0.951032729 | 0.4004666 | Wald ratio | LAP3 | 0.917383701 |
| -0.081039204 | 0.681708018 | 0.905373036 | Wald ratio | LAT | 1 |
| 1.293552683 | 0.565621367 | 0.022198236 | Wald ratio | LAT2 | 0.215758557 |
| -0.436612141 | 0.455865416 | 0.338181049 | Wald ratio | LATS1 | 0.897122605 |
| -0.136006514 | 0.147846253 | 0.357615313 | Inverse variance weighted (multiplicative random effects) | LAYN | 0.900699853 |
| 0.007771493 | 0.029581127 | 0.792767977 | Inverse variance weighted (multiplicative random effects) | LBP | 1 |
| -0.126268447 | 0.17929279 | 0.481271889 | Inverse variance weighted (multiplicative random effects) | LCAT | 0.940832265 |
| -0.005272367 | 0.038174647 | 0.890152109 | Inverse variance weighted (multiplicative random effects) | LCN15 | 1 |
| -0.12802449 | 0.046576087 | 0.005982898 | Inverse variance weighted (multiplicative random effects) | LCP1 | 0.080807974 |
| 0.708891345 | 1.185949667 | 0.550012423 | Wald ratio | LDLR | 0.977576895 |
| -0.113702138 | 0.462294239 | 0.805719481 | Wald ratio | LDLRAP1 | 1 |
| 0.065802852 | 0.054318945 | 0.225735921 | Inverse variance weighted (multiplicative random effects) | LECT2 | 0.786975851 |
| 0.005323741 | 0.04906778 | 0.913600908 | Inverse variance weighted (multiplicative random effects) | LEFTY2 | 1 |
| -0.088358326 | 0.082133705 | 0.282022781 | Inverse variance weighted (multiplicative random effects) | LEPR | 0.847831902 |
| -0.097209215 | 0.066253443 | 0.142312778 | Inverse variance weighted (multiplicative random effects) | LGALS1 | 0.660737897 |
| -0.027746964 | 0.078527539 | 0.723833146 | Inverse variance weighted (multiplicative random effects) | LGALS3 | 1 |
| 0.084823686 | 0.072068938 | 0.239203559 | Inverse variance weighted (multiplicative random effects) | LGALS3BP | 0.793187763 |
| 0.676012376 | 0.389680461 | 0.08277864 | Inverse variance weighted (multiplicative random effects) | LGALS4 | 0.516101454 |
| 0.043761764 | 0.117935337 | 0.710588559 | Inverse variance weighted (multiplicative random effects) | LGALS7 | 1 |
| 0.043761764 | 0.117935337 | 0.710588559 | Inverse variance weighted (multiplicative random effects) | LGALS7B | 1 |
| -0.07444297 | 0.053342947 | 0.162848805 | Inverse variance weighted (multiplicative random effects) | LGALS8 | 0.701473133 |
| 0.045710038 | 0.0706796 | 0.517812016 | Inverse variance weighted (multiplicative random effects) | LGALS9 | 0.963371193 |
| 0.19625023 | 0.118452708 | 0.097563703 | Inverse variance weighted (multiplicative random effects) | LGMN | 0.555979456 |
| 0.209319845 | 0.416648028 | 0.61539314 | Wald ratio | LHB | 0.994517296 |
| -0.006639576 | 0.062624596 | 0.915565015 | Inverse variance weighted (multiplicative random effects) | LHPP | 1 |
| -0.041548328 | 0.112636195 | 0.712223202 | Inverse variance weighted (multiplicative random effects) | LIFR | 1 |
| -0.014726172 | 0.064271984 | 0.818773536 | Inverse variance weighted (multiplicative random effects) | LILRA2 | 1 |
| 0.027552225 | 0.03990295 | 0.489891817 | Inverse variance weighted (multiplicative random effects) | LILRA3 | 0.946123472 |
| 0.11834414 | 0.088346443 | 0.180392929 | Inverse variance weighted (multiplicative random effects) | LILRA5 | 0.740254766 |
| -0.019580019 | 0.042466543 | 0.644748888 | Inverse variance weighted (multiplicative random effects) | LILRA6 | 0.998066246 |
| -0.014587315 | 0.044913004 | 0.74533967 | Inverse variance weighted (multiplicative random effects) | LILRB1 | 1 |
| 0.01350665 | 0.035020298 | 0.699733229 | Inverse variance weighted (multiplicative random effects) | LILRB2 | 1 |
| 0.069131535 | 0.156082397 | 0.657825827 | Inverse variance weighted (multiplicative random effects) | LILRB4 | 0.998066246 |
| -0.074146486 | 0.057646453 | 0.198363477 | Inverse variance weighted (multiplicative random effects) | LILRB5 | 0.759210165 |
| 0.162694222 | 0.068314996 | 0.01724088 | Inverse variance weighted (multiplicative random effects) | LIPF | 0.181176745 |
| 0.311491493 | 0.729386792 | 0.669336056 | Wald ratio | LMNB1 | 0.999439337 |
| -0.662797829 | 0.704101728 | 0.34653159 | Wald ratio | LMNB2 | 0.897122605 |
| 0.114395924 | 0.162367028 | 0.481089424 | Wald ratio | LMOD1 | 0.940832265 |
| 0.12221361 | 0.173463003 | 0.481089424 | Wald ratio | LMOD1 | 0.940832265 |
| 0.113861036 | 0.161607839 | 0.481089424 | Wald ratio | LMOD1 | 0.940832265 |
| 0.120424636 | 0.170923837 | 0.481089424 | Wald ratio | LMOD1 | 0.940832265 |
| 0.058028734 | 0.164702514 | 0.724594905 | Wald ratio | LONP1 | 1 |
| 0.132956796 | 0.067245734 | 0.048021497 | Inverse variance weighted (multiplicative random effects) | LPA | 0.382700053 |
| -0.019753695 | 0.032731016 | 0.546165403 | Inverse variance weighted (multiplicative random effects) | LPCAT2 | 0.975364036 |
| -0.104158104 | 0.110601999 | 0.346326797 | Inverse variance weighted (multiplicative random effects) | LPL | 0.897122605 |
| -0.13513237 | 0.019915754 | 1.16E-11 | Inverse variance weighted (multiplicative random effects) | LPO | 5.48E-10 |
| -0.483542008 | 0.419031588 | 0.248520172 | Wald ratio | LRCH4 | 0.801429393 |
| 0.083387815 | 0.089859499 | 0.353417956 | Inverse variance weighted (multiplicative random effects) | LRG1 | 0.900497022 |
| 0.018698183 | 0.066150014 | 0.777434935 | Inverse variance weighted (multiplicative random effects) | LRIG1 | 1 |
| 0.376864798 | 0.406265432 | 0.353598515 | Wald ratio | LRIG3 | 0.900497022 |
| -0.449118728 | 0.505977572 | 0.374742092 | Inverse variance weighted (multiplicative random effects) | LRP1 | 0.909083377 |
| -0.02739606 | 0.109715775 | 0.802819141 | Inverse variance weighted (multiplicative random effects) | LRP11 | 1 |
| 0.092017541 | 0.0654891 | 0.159996961 | Inverse variance weighted (multiplicative random effects) | LRPAP1 | 0.700551705 |
| -0.028386568 | 0.125817038 | 0.821498666 | Inverse variance weighted (multiplicative random effects) | LRRC25 | 1 |
| -0.008954869 | 0.042782236 | 0.834204064 | Inverse variance weighted (multiplicative random effects) | LRRC37A2 | 1 |
| -0.036543469 | 0.044085404 | 0.407147165 | Inverse variance weighted (multiplicative random effects) | LRRC38 | 0.92281948 |
| -0.376201789 | 0.568593905 | 0.50820499 | Wald ratio | LRRFIP1 | 0.956220371 |
| -0.028043607 | 0.033354421 | 0.400473269 | Inverse variance weighted (multiplicative random effects) | LRRN1 | 0.917383701 |
| -0.06960648 | 0.142829627 | 0.626017952 | Inverse variance weighted (multiplicative random effects) | LRTM2 | 0.998066246 |
| -0.013287715 | 0.053969748 | 0.805522215 | Inverse variance weighted (multiplicative random effects) | LTA | 1 |
| -0.098403469 | 0.283678384 | 0.728678529 | Wald ratio | LTA4H | 1 |
| 0.022854166 | 0.356201356 | 0.948842185 | Inverse variance weighted (multiplicative random effects) | LTB | 1 |
| 0.152109346 | 0.23906641 | 0.524604378 | Inverse variance weighted (multiplicative random effects) | LTBP2 | 0.967500298 |
| 0.012462796 | 0.064594276 | 0.847006245 | Inverse variance weighted (multiplicative random effects) | LTBP3 | 1 |
| -0.043061617 | 0.100769967 | 0.669141973 | Wald ratio | LTBR | 0.999439337 |
| -0.078244328 | 0.098785378 | 0.428323441 | Inverse variance weighted (multiplicative random effects) | LUZP2 | 0.93094332 |
| -0.194014372 | 0.180516001 | 0.282474764 | Inverse variance weighted (multiplicative random effects) | LXN | 0.847831902 |
| 0.095092014 | 0.097216797 | 0.328003174 | Inverse variance weighted (multiplicative random effects) | LY6D | 0.879183767 |
| -0.07825918 | 0.042140834 | 0.06329883 | Inverse variance weighted (multiplicative random effects) | LY75 | 0.454005402 |
| -0.044378554 | 0.078620314 | 0.572436511 | Inverse variance weighted (multiplicative random effects) | LY9 | 0.989304274 |
| 0.245419572 | 0.630401406 | 0.697049186 | Inverse variance weighted (multiplicative random effects) | LY96 | 1 |
| -0.943314738 | 0.545686724 | 0.083867363 | Wald ratio | LYAR | 0.516101454 |
| 0.099115023 | 0.099786415 | 0.320577564 | Inverse variance weighted (multiplicative random effects) | LYPD3 | 0.873862934 |
| 0.002493553 | 0.10647591 | 0.981316093 | Inverse variance weighted (multiplicative random effects) | LYPD8 | 1 |
| 0.475871614 | 0.525846971 | 0.365485432 | Wald ratio | LYSMD3 | 0.903935432 |
| -0.123654482 | 0.030789866 | 5.92E-05 | Inverse variance weighted (multiplicative random effects) | LYVE1 | 0.001577996 |
| 0.016150133 | 0.130946999 | 0.90184313 | Wald ratio | LYZL2 | 1 |
| -0.071545814 | 0.158955241 | 0.652638063 | Wald ratio | LZTFL1 | 0.998066246 |
| -0.117042641 | 0.21913638 | 0.593266351 | Inverse variance weighted (multiplicative random effects) | M6PR | 0.989304274 |
| 0.054966879 | 0.103950751 | 0.596959404 | Inverse variance weighted (multiplicative random effects) | MAD1L1 | 0.989304274 |
| -0.086000238 | 0.081095206 | 0.288924074 | Inverse variance weighted (multiplicative random effects) | MAMDC2 | 0.854512224 |
| -0.190274352 | 0.041737894 | 5.14E-06 | Inverse variance weighted (multiplicative random effects) | MAMDC4 | 0.000169863 |
| -0.012989966 | 0.074729034 | 0.862000982 | Inverse variance weighted (multiplicative random effects) | MAN1A2 | 1 |
| 0.044514985 | 0.066703449 | 0.504544398 | Inverse variance weighted (multiplicative random effects) | MAN2B2 | 0.956220371 |
| 0.37643447 | 0.429099759 | 0.380342376 | Wald ratio | MANF | 0.911419519 |
| -0.071872881 | 0.091748183 | 0.433409182 | Inverse variance weighted (multiplicative random effects) | MANSC4 | 0.932255531 |
| -0.395586469 | 0.462723921 | 0.392601977 | Inverse variance weighted (multiplicative random effects) | MAP2 | 0.915857498 |
| -0.755669758 | 0.535138787 | 0.157920367 | Wald ratio | MAP2K1 | 0.698760494 |
| -0.040295574 | 0.351462595 | 0.908721672 | Inverse variance weighted (multiplicative random effects) | MAP4K5 | 1 |
| -0.043795709 | 0.530891861 | 0.934253416 | Wald ratio | MAPK13 | 1 |
| -0.060572735 | 0.251962834 | 0.810017503 | Wald ratio | MAPK9 | 1 |
| 0.086732232 | 0.124450183 | 0.485850794 | Inverse variance weighted (multiplicative random effects) | MAPKAPK2 | 0.943195135 |
| 0.013200402 | 0.038483622 | 0.731588303 | Inverse variance weighted (multiplicative random effects) | MARCO | 1 |
| 0.0577297 | 0.106339776 | 0.587212671 | Inverse variance weighted (multiplicative random effects) | MASP1 | 0.989304274 |
| 0.092285929 | 0.075491594 | 0.221531343 | Inverse variance weighted (multiplicative random effects) | MATN2 | 0.783552201 |
| 0.102030003 | 0.052544393 | 0.052162927 | Inverse variance weighted (multiplicative random effects) | MATN3 | 0.403794718 |
| 0.146131636 | 0.332872787 | 0.660660487 | Wald ratio | MAVS | 0.998066246 |
| 0.0614669 | 0.368074097 | 0.8673732 | Wald ratio | MAX | 1 |
| 0.054854422 | 0.762669489 | 0.942662201 | Wald ratio | MB | 1 |
| 0.008425021 | 0.040942262 | 0.836964249 | Inverse variance weighted (multiplicative random effects) | MBL2 | 1 |
| -0.403708183 | 0.137426954 | 0.00330742 | Inverse variance weighted (multiplicative random effects) | MCAM | 0.05021485 |
| 0.082778936 | 0.151953764 | 0.585915862 | Wald ratio | MCEE | 0.989304274 |
| -0.010417682 | 0.21613677 | 0.961557256 | Inverse variance weighted (multiplicative random effects) | MCEMP1 | 1 |
| 0.024221885 | 0.214293272 | 0.910005601 | Wald ratio | MCFD2 | 1 |
| -0.005270627 | 0.043209386 | 0.902915854 | Inverse variance weighted (multiplicative random effects) | MDGA1 | 1 |
| 0.000743747 | 0.085724278 | 0.993077606 | Inverse variance weighted (multiplicative random effects) | MDK | 1 |
| -0.059881312 | 0.157318507 | 0.703472304 | Wald ratio | MDM1 | 1 |
| -0.188507201 | 0.499387771 | 0.705819447 | Inverse variance weighted (multiplicative random effects) | MECR | 1 |
| -0.147770362 | 0.220773957 | 0.503285775 | Inverse variance weighted (multiplicative random effects) | MEGF10 | 0.956220371 |
| 0.118860445 | 0.168117208 | 0.479560641 | Inverse variance weighted (multiplicative random effects) | MEGF11 | 0.940832265 |
| 0.083209249 | 0.010231755 | 4.21E-16 | Inverse variance weighted (multiplicative random effects) | MEGF9 | 2.43E-14 |
| 0.076786352 | 0.046858053 | 0.101275483 | Inverse variance weighted (multiplicative random effects) | MELTF | 0.562880046 |
| 0.064925994 | 1.169768375 | 0.955737509 | Wald ratio | MENT | 1 |
| -0.098685496 | 0.161940333 | 0.542263197 | Inverse variance weighted (multiplicative random effects) | MEP1A | 0.972958495 |
| -0.001442497 | 0.030924016 | 0.962794973 | Inverse variance weighted (multiplicative random effects) | MEP1B | 1 |
| -0.141738702 | 0.073012145 | 0.052221528 | Inverse variance weighted (multiplicative random effects) | MEPE | 0.403794718 |
| 0.087700984 | 0.113451743 | 0.439507847 | Wald ratio | MERTK | 0.934538753 |
| 0.709034483 | 0.870285572 | 0.415235614 | Wald ratio | MESD | 0.923732703 |
| 0.023467674 | 0.248681286 | 0.924816456 | Wald ratio | MET | 1 |
| 0.295054584 | 0.329768961 | 0.370930787 | Inverse variance weighted (multiplicative random effects) | METAP1D | 0.909083377 |
| -0.053030131 | 0.044691013 | 0.235387418 | Inverse variance weighted (multiplicative random effects) | MFAP4 | 0.793187763 |
| -0.079197037 | 0.054520451 | 0.146331632 | Inverse variance weighted (multiplicative random effects) | MFAP5 | 0.670958453 |
| 0.100161853 | 0.128403733 | 0.435359055 | Inverse variance weighted (multiplicative random effects) | MFGE8 | 0.933284251 |
| 0.444380197 | 0.476799327 | 0.351333032 | Wald ratio | MGLL | 0.898859417 |
| 0.120411878 | 0.064260184 | 0.060955541 | Inverse variance weighted (multiplicative random effects) | MGMT | 0.442304515 |
| 0.018447497 | 0.02914585 | 0.526774552 | Inverse variance weighted (multiplicative random effects) | MIA | 0.967500298 |
| -0.014552009 | 0.026450787 | 0.582213685 | Inverse variance weighted (multiplicative random effects) | MICA | 0.989304274 |
| 0.180987523 | 0.042988616 | 2.55E-05 | Inverse variance weighted (multiplicative random effects) | MICALL2 | 0.000747708 |
| 0.011465349 | 0.030216879 | 0.704364667 | Inverse variance weighted (multiplicative random effects) | MICB | 1 |
| -0.391763966 | 0.383818766 | 0.307396408 | Inverse variance weighted (multiplicative random effects) | MIF | 0.869694804 |
| 0.02349201 | 0.029047248 | 0.418658011 | Inverse variance weighted (multiplicative random effects) | MILR1 | 0.926392194 |
| -0.034880005 | 0.003433603 | 3.04E-24 | Inverse variance weighted (multiplicative random effects) | MINDY1 | 2.34E-22 |
| -0.432697112 | 0.298247449 | 0.146835827 | Inverse variance weighted (multiplicative random effects) | MINK1 | 0.671249496 |
| 0.314771658 | 0.38456552 | 0.413064648 | Wald ratio | MITD1 | 0.923405266 |
| -0.055579375 | 0.054751486 | 0.310048225 | Inverse variance weighted (multiplicative random effects) | MLN | 0.869694804 |
| 0.087633784 | 0.18649423 | 0.638425908 | Inverse variance weighted (multiplicative random effects) | MME | 0.998066246 |
| 0.031599888 | 0.075650683 | 0.676160693 | Inverse variance weighted (multiplicative random effects) | MMP1 | 1 |
| 0.022395469 | 0.075856245 | 0.767813859 | Inverse variance weighted (multiplicative random effects) | MMP10 | 1 |
| -0.013364059 | 0.053755318 | 0.8036632 | Inverse variance weighted (multiplicative random effects) | MMP12 | 1 |
| -1.131162638 | 0.72932132 | 0.120906524 | Wald ratio | MMP13 | 0.619422586 |
| -0.102329639 | 0.062325557 | 0.100619687 | Inverse variance weighted (multiplicative random effects) | MMP3 | 0.562880046 |
| 0.035360418 | 0.131260722 | 0.787629039 | Inverse variance weighted (multiplicative random effects) | MMP7 | 1 |
| 0.109445686 | 0.067284058 | 0.103817537 | Inverse variance weighted (multiplicative random effects) | MMP8 | 0.571271106 |
| 0.131255943 | 0.199493516 | 0.510572886 | Wald ratio | MMP9 | 0.956220371 |
| 0.100423695 | 0.286352287 | 0.72581356 | Wald ratio | MMUT | 1 |
| 0.158280792 | 0.07594182 | 0.037138565 | Inverse variance weighted (multiplicative random effects) | MOCS2 | 0.311484742 |
| 0.034380405 | 0.147074072 | 0.815169353 | Inverse variance weighted (multiplicative random effects) | MOG | 1 |
| 0.527726976 | 0.850916076 | 0.535134766 | Wald ratio | MPHOSPH8 | 0.971281608 |
| -0.109009382 | 0.344113257 | 0.751407929 | Inverse variance weighted (multiplicative random effects) | MPI | 1 |
| 0.011728363 | 0.085328253 | 0.89067516 | Inverse variance weighted (multiplicative random effects) | MPO | 1 |
| 0.12505033 | 0.20704138 | 0.545852195 | Inverse variance weighted (multiplicative random effects) | MRC1 | 0.975364036 |
| -0.091157922 | 0.05256517 | 0.082884613 | Inverse variance weighted (multiplicative random effects) | MRI1 | 0.516101454 |
| 0.064142214 | 0.035221275 | 0.068588418 | Inverse variance weighted (multiplicative random effects) | MSLN | 0.466783731 |
| -0.003974184 | 0.046996509 | 0.932608503 | Inverse variance weighted (multiplicative random effects) | MSR1 | 1 |
| -0.64176034 | 0.525797332 | 0.222257595 | Wald ratio | MSRA | 0.783552201 |
| 0.006652893 | 0.026629993 | 0.802721094 | Inverse variance weighted (multiplicative random effects) | MST1 | 1 |
| 0.079540931 | 0.189299987 | 0.674350634 | Inverse variance weighted (multiplicative random effects) | MSTN | 1 |
| 0.24508281 | 0.152566027 | 0.10818498 | Wald ratio | MTHFD2 | 0.587532004 |
| 0.038194655 | 0.060229192 | 0.525979541 | Inverse variance weighted (multiplicative random effects) | MTHFSD | 0.967500298 |
| 0.083332561 | 0.080620164 | 0.301302548 | Inverse variance weighted (multiplicative random effects) | MTIF3 | 0.869694804 |
| 1.328136203 | 0.630618484 | 0.035196959 | Wald ratio | MTR | 0.297600307 |
| 0.612668841 | 0.585257936 | 0.29517541 | Wald ratio | MTSS1 | 0.864739228 |
| 0.090475171 | 0.116893531 | 0.438932929 | Inverse variance weighted (multiplicative random effects) | MTSS2 | 0.934538753 |
| -0.048526698 | 0.197130907 | 0.805554632 | Inverse variance weighted (multiplicative random effects) | MTUS1 | 1 |
| 0.081295114 | 0.159494166 | 0.610257536 | Inverse variance weighted (multiplicative random effects) | MUC13 | 0.99391765 |
| 0.30380294 | 0.267101487 | 0.255368435 | Inverse variance weighted (multiplicative random effects) | MUC16 | 0.809704795 |
| -0.080378403 | 0.0961138 | 0.402995326 | Inverse variance weighted (multiplicative random effects) | MUC2 | 0.922145522 |
| -0.235147155 | 0.186601347 | 0.207612419 | Inverse variance weighted (multiplicative random effects) | MVK | 0.769129582 |
| 0.102500147 | 0.467725516 | 0.826536367 | Wald ratio | MXRA8 | 1 |
| -0.647763098 | 0.74885543 | 0.387036479 | Wald ratio | MYBPC2 | 0.91224413 |
| -0.444066205 | 0.429265955 | 0.300912736 | Wald ratio | MYDGF | 0.869694804 |
| 0.123138291 | 0.562928879 | 0.826848121 | Wald ratio | MYO9B | 1 |
| 0.136986434 | 0.014678754 | 1.04E-20 | Inverse variance weighted (multiplicative random effects) | MYOC | 7.43E-19 |
| -0.049765166 | 0.650812925 | 0.939048234 | Inverse variance weighted (multiplicative random effects) | MYOM2 | 1 |
| -0.408040814 | 0.375007268 | 0.276556372 | Wald ratio | MYOM3 | 0.845937138 |
| 0.265999744 | 0.305323517 | 0.38364157 | Wald ratio | MZB1 | 0.91151664 |
| 0.05252436 | 0.216311144 | 0.808145958 | Wald ratio | NAA80 | 1 |
| -0.070004075 | 0.055699416 | 0.208819251 | Inverse variance weighted (multiplicative random effects) | NAAA | 0.769129582 |
| 0.08632037 | 0.112019841 | 0.44095528 | Inverse variance weighted (multiplicative random effects) | NADK | 0.934538753 |
| 0.07056256 | 0.186765963 | 0.70556971 | Inverse variance weighted (multiplicative random effects) | NAGA | 1 |
| 0.000258306 | 0.08408023 | 0.997548791 | Inverse variance weighted (multiplicative random effects) | NAGK | 1 |
| -0.22534487 | 0.116869812 | 0.05383398 | Inverse variance weighted (multiplicative random effects) | NAGPA | 0.411671611 |
| 0.104031337 | 0.076439027 | 0.173522698 | Inverse variance weighted (multiplicative random effects) | NAP1L4 | 0.72933915 |
| 0.128997946 | 0.02696007 | 1.71E-06 | Inverse variance weighted (multiplicative random effects) | NAPRT | 6.03E-05 |
| -0.099471701 | 0.003051546 | 4.50E-233 | Inverse variance weighted (multiplicative random effects) | NBL1 | 1.56E-230 |
| 0.052442961 | 0.101738761 | 0.606226812 | Inverse variance weighted (multiplicative random effects) | NBN | 0.991998653 |
| 0.028002705 | 0.083468387 | 0.737256432 | Inverse variance weighted (multiplicative random effects) | NCAM1 | 1 |
| -0.015631174 | 0.054374928 | 0.77375228 | Inverse variance weighted (multiplicative random effects) | NCAM2 | 1 |
| -0.077507542 | 0.217238857 | 0.721252849 | Wald ratio | NCAN | 1 |
| -0.512257254 | 0.001306398 | 0 | Inverse variance weighted (multiplicative random effects) | NCF2 | 0 |
| -0.196444019 | 0.049417877 | 7.03E-05 | Inverse variance weighted (multiplicative random effects) | NCR1 | 0.00185177 |
| -0.015270339 | 0.053370144 | 0.774785166 | Inverse variance weighted (multiplicative random effects) | NCR3LG1 | 1 |
| -0.244171679 | 0.014774464 | 2.36E-61 | Inverse variance weighted (multiplicative random effects) | NCS1 | 3.27E-59 |
| -0.002324673 | 0.116152751 | 0.984032261 | Inverse variance weighted (multiplicative random effects) | NECTIN2 | 1 |
| -0.0007163 | 0.041591628 | 0.986259343 | Inverse variance weighted (multiplicative random effects) | NECTIN4 | 1 |
| -0.057768289 | 0.057967812 | 0.318979085 | Inverse variance weighted (multiplicative random effects) | NELL1 | 0.873862934 |
| -0.02263724 | 0.04922533 | 0.64560972 | Inverse variance weighted (multiplicative random effects) | NELL2 | 0.998066246 |
| -0.037723286 | 0.377309808 | 0.920360515 | Wald ratio | NEO1 | 1 |
| 0.142477674 | 0.275330942 | 0.604822603 | Wald ratio | NEXN | 0.991807207 |
| -0.06080445 | 0.048309259 | 0.208156775 | Inverse variance weighted (multiplicative random effects) | NFASC | 0.769129582 |
| 0.229732752 | 0.235434971 | 0.329173464 | Inverse variance weighted (multiplicative random effects) | NFATC1 | 0.880052449 |
| -0.236410067 | 0.464381258 | 0.610691839 | Wald ratio | NFE2 | 0.99391765 |
| -0.032642064 | 0.250598372 | 0.896363502 | Wald ratio | NFKB1 | 1 |
| -0.045722705 | 0.110899698 | 0.680127747 | Wald ratio | NFKBIE | 1 |
| 0.081789941 | 0.318027449 | 0.797040693 | Wald ratio | NFU1 | 1 |
| 0.324852406 | 0.104199388 | 0.001823277 | Inverse variance weighted (multiplicative random effects) | NGRN | 0.031085385 |
| 0.152718252 | 0.102780159 | 0.137312745 | Inverse variance weighted (multiplicative random effects) | NHLRC3 | 0.653345785 |
| 0.038364167 | 0.135062556 | 0.776374059 | Inverse variance weighted (multiplicative random effects) | NID1 | 1 |
| 0.00855145 | 0.043313344 | 0.843489306 | Inverse variance weighted (multiplicative random effects) | NID2 | 1 |
| 0.216465587 | 0.189689633 | 0.253804739 | Inverse variance weighted (multiplicative random effects) | NINJ1 | 0.807778299 |
| 0.161251189 | 0.223306679 | 0.470229044 | Wald ratio | NIT1 | 0.940832265 |
| 0.30290219 | 0.291519838 | 0.298783873 | Inverse variance weighted (multiplicative random effects) | NIT2 | 0.867975498 |
| -0.139561899 | 0.088682749 | 0.11555198 | Inverse variance weighted (multiplicative random effects) | NME3 | 0.600870294 |
| 0.056367458 | 0.095990794 | 0.557057753 | Inverse variance weighted (multiplicative random effects) | NMI | 0.979442202 |
| 0.270146307 | 0.283435732 | 0.340532774 | Inverse variance weighted (multiplicative random effects) | NMNAT1 | 0.897122605 |
| -0.103644846 | 0.143919445 | 0.471427265 | Inverse variance weighted (multiplicative random effects) | NOMO1 | 0.940832265 |
| 0.197370786 | 0.194061572 | 0.309128506 | Inverse variance weighted (multiplicative random effects) | NOS1 | 0.869694804 |
| 0.06992798 | 0.429999369 | 0.870814924 | Inverse variance weighted (multiplicative random effects) | NOS2 | 1 |
| 0.073622102 | 0.772308266 | 0.924054817 | Wald ratio | NOTCH1 | 1 |
| -0.064108168 | 0.076122689 | 0.399693371 | Inverse variance weighted (multiplicative random effects) | NOTCH2 | 0.917383701 |
| 0.350190716 | 0.093161309 | 0.000170613 | Inverse variance weighted (multiplicative random effects) | NOTCH3 | 0.004126459 |
| -0.094195794 | 0.094467579 | 0.318704822 | Inverse variance weighted (multiplicative random effects) | NPC2 | 0.873862934 |
| -0.008690526 | 0.024967478 | 0.727784733 | Inverse variance weighted (multiplicative random effects) | NPDC1 | 1 |
| -0.02720602 | 0.043567079 | 0.532323699 | Inverse variance weighted (multiplicative random effects) | NPHS1 | 0.971281608 |
| -0.031797429 | 0.133451936 | 0.811672637 | Inverse variance weighted (multiplicative random effects) | NPL | 1 |
| -0.065494404 | 0.261494237 | 0.802229987 | Inverse variance weighted (multiplicative random effects) | NPPB | 1 |
| 0.270895257 | 1.097391509 | 0.805021377 | Inverse variance weighted (multiplicative random effects) | NPPC | 1 |
| -0.12578423 | 0.074497002 | 0.091325477 | Inverse variance weighted (multiplicative random effects) | NPTX1 | 0.538121793 |
| -0.057117265 | 0.100257101 | 0.568875815 | Inverse variance weighted (multiplicative random effects) | NPTX2 | 0.989304274 |
| -0.002687405 | 0.026936677 | 0.920528896 | Inverse variance weighted (multiplicative random effects) | NPTXR | 1 |
| 0.138075705 | 0.278529517 | 0.62008424 | Wald ratio | NPY | 0.99750597 |
| -0.005771151 | 0.067663026 | 0.93202883 | Inverse variance weighted (multiplicative random effects) | NRCAM | 1 |
| 0.064495945 | 0.072898739 | 0.376300469 | Inverse variance weighted (multiplicative random effects) | NRP1 | 0.909318524 |
| -0.36211658 | 0.127631483 | 0.004551049 | Inverse variance weighted (multiplicative random effects) | NRP2 | 0.066197083 |
| -0.119776051 | 0.501769472 | 0.811332533 | Wald ratio | NRTN | 1 |
| 0.136058142 | 0.381162047 | 0.721124622 | Wald ratio | NSFL1C | 1 |
| 0.091778096 | 0.096096534 | 0.339546616 | Inverse variance weighted (multiplicative random effects) | NT5C | 0.897122605 |
| 0.420449943 | 0.283383041 | 0.137893654 | Wald ratio | NT5C3A | 0.653345785 |
| 0.047678394 | 0.065769816 | 0.468496084 | Inverse variance weighted (multiplicative random effects) | NT5E | 0.940832265 |
| -0.132502427 | 0.127013937 | 0.296850251 | Inverse variance weighted (multiplicative random effects) | NTF3 | 0.865986706 |
| -0.060596704 | 0.186274038 | 0.744946884 | Inverse variance weighted (multiplicative random effects) | NTproBNP | 1 |
| -0.092582497 | 0.191948456 | 0.629571546 | Inverse variance weighted (multiplicative random effects) | NTRK2 | 0.998066246 |
| 0.055056532 | 0.133896932 | 0.680936215 | Inverse variance weighted (multiplicative random effects) | NTRK3 | 1 |
| -0.0792232 | 0.088639053 | 0.371443311 | Inverse variance weighted (multiplicative random effects) | NUB1 | 0.909083377 |
| 0.021698037 | 0.096714981 | 0.822484724 | Inverse variance weighted (multiplicative random effects) | NUCB2 | 1 |
| -0.147177928 | 0.198561274 | 0.458559021 | Wald ratio | NUDT15 | 0.940832265 |
| 0.153151131 | 0.196169982 | 0.434975284 | Inverse variance weighted (multiplicative random effects) | NUDT16 | 0.933284251 |
| 0.068758798 | 0.115532483 | 0.551745288 | Inverse variance weighted (multiplicative random effects) | NUDT2 | 0.977576895 |
| 0.416942555 | 0.502081355 | 0.406296684 | Wald ratio | NUDT5 | 0.922595089 |
| -0.209845112 | 0.372730389 | 0.573438714 | Inverse variance weighted (multiplicative random effects) | NUMB | 0.989304274 |
| 0.171739211 | 0.072867993 | 0.018430497 | Inverse variance weighted (multiplicative random effects) | NXPH3 | 0.188844504 |
| 0.030748762 | 0.04497454 | 0.494169278 | Inverse variance weighted (multiplicative random effects) | OBP2B | 0.949974212 |
| 0.297780229 | 0.303838015 | 0.327055293 | Inverse variance weighted (multiplicative random effects) | OCLN | 0.878188209 |
| 0.103292821 | 0.082407776 | 0.210047306 | Inverse variance weighted (multiplicative random effects) | ODAM | 0.769187318 |
| 0.421678258 | 0.093342354 | 6.26E-06 | Inverse variance weighted (multiplicative random effects) | OGA | 0.000197161 |
| 0.333967895 | 0.015264944 | 4.20E-106 | Inverse variance weighted (multiplicative random effects) | OGFR | 8.74E-104 |
| -0.007830457 | 0.074714263 | 0.916530236 | Inverse variance weighted (multiplicative random effects) | OGN | 1 |
| 0.08700635 | 0.018059531 | 1.45E-06 | Inverse variance weighted (multiplicative random effects) | OLFM4 | 5.21E-05 |
| 0.940843404 | 0.535587421 | 0.078976287 | Wald ratio | OLR1 | 0.50235681 |
| -0.761723792 | 0.46137841 | 0.098743869 | Wald ratio | OMD | 0.561167345 |
| 0.712071224 | 0.367818856 | 0.052876386 | Wald ratio | OMG | 0.407344009 |
| -0.062461846 | 0.116543687 | 0.591991583 | Wald ratio | OMP | 0.989304274 |
| -0.069136066 | 0.155675791 | 0.656968209 | Wald ratio | OPLAH | 0.998066246 |
| 0.038124784 | 0.058190288 | 0.512355508 | Inverse variance weighted (multiplicative random effects) | OPTC | 0.956342919 |
| -0.072419778 | 0.108466292 | 0.504343742 | Inverse variance weighted (multiplicative random effects) | ORM1 | 0.956220371 |
| 0.002349051 | 0.045817976 | 0.959111017 | Inverse variance weighted (multiplicative random effects) | OSCAR | 1 |
| -0.495709073 | 0.413847919 | 0.230992972 | Wald ratio | OSM | 0.793187763 |
| -0.089299538 | 0.088005496 | 0.310246897 | Inverse variance weighted (multiplicative random effects) | OSMR | 0.869694804 |
| 0.426090906 | 0.351836661 | 0.225877203 | Wald ratio | OSTN | 0.786975851 |
| -0.153308803 | 0.499056029 | 0.75869299 | Wald ratio | OTOA | 1 |
| 1.667148163 | 0.942956533 | 0.077060724 | Wald ratio | OTUD7B | 0.496242433 |
| 0.37425009 | 0.664082644 | 0.573053948 | Wald ratio | OXCT1 | 0.989304274 |
| 0.027651912 | 0.075437715 | 0.713952561 | Inverse variance weighted (multiplicative random effects) | OXT | 1 |
| 0.205134515 | 0.456793814 | 0.653377819 | Wald ratio | P4HB | 0.998066246 |
| 0.128749017 | 0.168020213 | 0.443515068 | Wald ratio | PACS2 | 0.934538753 |
| -0.107433407 | 0.235573867 | 0.648354194 | Wald ratio | PADI2 | 0.998066246 |
| -0.178370615 | 0.071379082 | 0.012457243 | Inverse variance weighted (multiplicative random effects) | PADI4 | 0.143950364 |
| -0.098913675 | 0.063675666 | 0.120327998 | Inverse variance weighted (multiplicative random effects) | PAEP | 0.618769297 |
| -0.26121367 | 0.640330578 | 0.68332092 | Wald ratio | PAFAH2 | 1 |
| 0.546292445 | 0.408193485 | 0.180792991 | Wald ratio | PAG1 | 0.740254766 |
| -0.029330433 | 0.258919931 | 0.909808624 | Inverse variance weighted (multiplicative random effects) | PALM | 1 |
| 0.044307087 | 0.135094539 | 0.742933614 | Inverse variance weighted (multiplicative random effects) | PALM2 | 1 |
| 0.002781816 | 0.05382174 | 0.95877911 | Inverse variance weighted (multiplicative random effects) | PAM | 1 |
| -0.042213915 | 0.04217386 | 0.316851099 | Inverse variance weighted (multiplicative random effects) | PAMR1 | 0.873862934 |
| -0.110968079 | 0.10810158 | 0.304648089 | Inverse variance weighted (multiplicative random effects) | PAPPA | 0.869694804 |
| 0.352053863 | 0.596245352 | 0.554888105 | Wald ratio | PARD3 | 0.977576895 |
| -0.024593949 | 0.154977226 | 0.873909999 | Wald ratio | PARK7 | 1 |
| 0.098085995 | 0.201584809 | 0.626560014 | Wald ratio | PARP1 | 0.998066246 |
| 0.321525867 | 0.18126553 | 0.076098913 | Wald ratio | PAXX | 0.496193541 |
| -0.072629118 | 0.104891972 | 0.488674691 | Inverse variance weighted (multiplicative random effects) | PBLD | 0.946123472 |
| 0.491648783 | 0.645090554 | 0.445976944 | Wald ratio | PBXIP1 | 0.935865266 |
| 0.177555961 | 0.164800282 | 0.281301233 | Wald ratio | PCBD1 | 0.847831902 |
| -0.574376863 | 0.353840254 | 0.104532549 | Wald ratio | PCDH1 | 0.572744944 |
| -0.154986484 | 0.056827772 | 0.00638546 | Inverse variance weighted (multiplicative random effects) | PCDH12 | 0.083533068 |
| 0.225024746 | 0.050175329 | 7.30E-06 | Inverse variance weighted (multiplicative random effects) | PCDH17 | 0.000226605 |
| 0.249442578 | 0.274678211 | 0.363811362 | Wald ratio | PCDH7 | 0.901939968 |
| -0.203655365 | 0.088883985 | 0.021949001 | Inverse variance weighted (multiplicative random effects) | PCDH9 | 0.214337661 |
| 0.054826577 | 0.053192864 | 0.302675418 | Inverse variance weighted (multiplicative random effects) | PCDHB15 | 0.869694804 |
| 0.076496961 | 0.166500338 | 0.645918033 | Wald ratio | PCOLCE | 0.998066246 |
| 0.000346117 | 0.074510549 | 0.996293669 | Inverse variance weighted (multiplicative random effects) | PCSK7 | 1 |
| 0.092583229 | 0.009372277 | 5.16E-23 | Inverse variance weighted (multiplicative random effects) | PCSK9 | 3.84E-21 |
| -0.219402113 | 0.530406648 | 0.679131274 | Wald ratio | PCYT2 | 1 |
| -0.20970334 | 0.235785106 | 0.373797107 | Inverse variance weighted (multiplicative random effects) | PDCD1 | 0.909083377 |
| 0.011983697 | 0.088642671 | 0.892460815 | Inverse variance weighted (multiplicative random effects) | PDCD1LG2 | 1 |
| 0.094232516 | 0.077136857 | 0.221848477 | Inverse variance weighted (multiplicative random effects) | PDCD5 | 0.783552201 |
| 0.011955743 | 0.038300545 | 0.754922002 | Inverse variance weighted (multiplicative random effects) | PDCD6 | 1 |
| 0.004172619 | 0.043776235 | 0.924063047 | Inverse variance weighted (multiplicative random effects) | PDE5A | 1 |
| 0.048092916 | 1.121575852 | 0.965797374 | Inverse variance weighted (multiplicative random effects) | PDGFA | 1 |
| -0.234925951 | 0.761820711 | 0.757797513 | Inverse variance weighted (multiplicative random effects) | PDGFC | 1 |
| 0.051846987 | 0.101978105 | 0.611163786 | Inverse variance weighted (multiplicative random effects) | PDGFRA | 0.99391765 |
| 0.011183228 | 0.043502985 | 0.797126239 | Inverse variance weighted (multiplicative random effects) | PDGFRB | 1 |
| -0.242824361 | 0.653185316 | 0.710075894 | Wald ratio | PDIA3 | 1 |
| 0.156068859 | 0.378894313 | 0.680408288 | Wald ratio | PDIA4 | 1 |
| -0.085425216 | 0.013758956 | 5.34E-10 | Inverse variance weighted (multiplicative random effects) | PDIA5 | 2.27E-08 |
| 0.144083892 | 0.681179313 | 0.832480561 | Wald ratio | PDLIM7 | 1 |
| 0.021791893 | 0.102820805 | 0.83215345 | Inverse variance weighted (multiplicative random effects) | PDZD2 | 1 |
| -0.034167669 | 0.110047233 | 0.756194553 | Inverse variance weighted (multiplicative random effects) | PEAR1 | 1 |
| 0.099088334 | 0.111617475 | 0.374675623 | Inverse variance weighted (multiplicative random effects) | PEBP1 | 0.909083377 |
| 0.002458158 | 0.031959407 | 0.938691156 | Inverse variance weighted (multiplicative random effects) | PECR | 1 |
| -0.067031504 | 0.075972177 | 0.377605682 | Inverse variance weighted (multiplicative random effects) | PENK | 0.909825428 |
| -0.024326428 | 0.007470392 | 0.00112843 | Inverse variance weighted (multiplicative random effects) | PEPD | 0.021732731 |
| -0.04588301 | 0.088623969 | 0.604648844 | Inverse variance weighted (multiplicative random effects) | PER3 | 0.991807207 |
| -0.332056826 | 0.383512552 | 0.386583176 | Wald ratio | PF4 | 0.91224413 |
| 0.000506217 | 0.152208377 | 0.99734639 | Wald ratio | PFKFB2 | 1 |
| 0.697376026 | 0.592531855 | 0.239218388 | Wald ratio | PGA4 | 0.793187763 |
| 0.056489938 | 0.130623066 | 0.665403299 | Inverse variance weighted (multiplicative random effects) | PGD | 0.999439337 |
| 0.266663052 | 1.190440688 | 0.822754468 | Wald ratio | PGF | 1 |
| 0.003964079 | 0.042690099 | 0.926017078 | Inverse variance weighted (multiplicative random effects) | PGLYRP1 | 1 |
| 0.002372569 | 0.045774105 | 0.958662453 | Inverse variance weighted (multiplicative random effects) | PGLYRP2 | 1 |
| -0.029325705 | 0.102905707 | 0.775662171 | Inverse variance weighted (multiplicative random effects) | PGLYRP4 | 1 |
| -0.372457043 | 0.582922145 | 0.522856645 | Wald ratio | PHACTR2 | 0.967500298 |
| 1.836509415 | 0.657295895 | 0.005205443 | Wald ratio | PHLDB1 | 0.072988338 |
| -0.10069258 | 0.081870851 | 0.21873629 | Inverse variance weighted (multiplicative random effects) | PHYKPL | 0.780397055 |
| -0.000604229 | 0.071797449 | 0.993285288 | Inverse variance weighted (multiplicative random effects) | PI16 | 1 |
| -0.12422946 | 0.086766716 | 0.152211374 | Inverse variance weighted (multiplicative random effects) | PI3 | 0.689161822 |
| 0.071858469 | 0.478764191 | 0.880692371 | Wald ratio | PIBF1 | 1 |
| -0.692795013 | 0.175406282 | 7.83E-05 | Inverse variance weighted (multiplicative random effects) | PIGR | 0.002009706 |
| 0.19630119 | 0.12042881 | 0.103097586 | Inverse variance weighted (multiplicative random effects) | PIK3AP1 | 0.568814265 |
| 0.001563164 | 0.05069424 | 0.975401022 | Inverse variance weighted (multiplicative random effects) | PIK3IP1 | 1 |
| -0.09364168 | 0.053771552 | 0.081600786 | Inverse variance weighted (multiplicative random effects) | PILRA | 0.512778355 |
| 0.022340114 | 0.060525747 | 0.712052446 | Inverse variance weighted (multiplicative random effects) | PILRB | 1 |
| -0.033071015 | 0.044801254 | 0.460410157 | Inverse variance weighted (multiplicative random effects) | PINLYP | 0.940832265 |
| 0.115830236 | 0.047745479 | 0.015266532 | Inverse variance weighted (multiplicative random effects) | PKD1 | 0.167128349 |
| 0.857858727 | 0.405043911 | 0.034180144 | Wald ratio | PKD2 | 0.291371719 |
| 0.040194882 | 0.136908304 | 0.769071368 | Inverse variance weighted (multiplicative random effects) | PKLR | 1 |
| 0.14083903 | 0.214956164 | 0.512339738 | Wald ratio | PKN3 | 0.956342919 |
| -0.189234444 | 0.441713925 | 0.668353005 | Inverse variance weighted (multiplicative random effects) | PLA2G10 | 0.999439337 |
| -0.070610828 | 0.134820616 | 0.600460014 | Inverse variance weighted (multiplicative random effects) | PLA2G15 | 0.989304274 |
| 0.114193395 | 0.501678433 | 0.819939579 | Wald ratio | PLA2G1B | 1 |
| -0.059463305 | 0.056292293 | 0.290816931 | Inverse variance weighted (multiplicative random effects) | PLA2G2A | 0.858013073 |
| -0.669064126 | 0.475267971 | 0.159201596 | Wald ratio | PLA2G4A | 0.698760494 |
| 0.626690707 | 0.002867848 | 0 | Inverse variance weighted (multiplicative random effects) | PLA2G7 | 0 |
| 0.227585149 | 0.570134214 | 0.689761905 | Wald ratio | PLAT | 1 |
| -0.112392685 | 0.214074858 | 0.599572201 | Inverse variance weighted (multiplicative random effects) | PLAU | 0.989304274 |
| -0.307492684 | 0.115171192 | 0.007587949 | Inverse variance weighted (multiplicative random effects) | PLAUR | 0.095654148 |
| 0.030518395 | 0.048353213 | 0.527938865 | Inverse variance weighted (multiplicative random effects) | PLB1 | 0.967500298 |
| 0.128427032 | 0.111450592 | 0.249188546 | Inverse variance weighted (multiplicative random effects) | PLCB2 | 0.802340828 |
| -0.280070093 | 0.510839739 | 0.583517289 | Inverse variance weighted (multiplicative random effects) | PLEKHO1 | 0.989304274 |
| -0.109049484 | 0.04854249 | 0.024673606 | Inverse variance weighted (multiplicative random effects) | PLG | 0.230139462 |
| -0.095439897 | 0.396464472 | 0.809766331 | Wald ratio | PLIN3 | 1 |
| -0.240810573 | 0.650341895 | 0.711171611 | Wald ratio | PLPBP | 1 |
| -0.48899193 | 0.280169869 | 0.080925548 | Wald ratio | PLSCR3 | 0.511857606 |
| 0.084842082 | 0.033325044 | 0.010899778 | Inverse variance weighted (multiplicative random effects) | PLTP | 0.130712361 |
| -0.088188775 | 0.066258527 | 0.183195591 | Inverse variance weighted (multiplicative random effects) | PLXDC1 | 0.741887316 |
| -0.020270572 | 0.13312337 | 0.87897472 | Inverse variance weighted (multiplicative random effects) | PLXDC2 | 1 |
| -0.270423806 | 0.316972402 | 0.393578224 | Wald ratio | PLXNA4 | 0.915857498 |
| 0.016089947 | 0.02273677 | 0.479155213 | Inverse variance weighted (multiplicative random effects) | PLXNB2 | 0.940832265 |
| 0.022162999 | 0.055281657 | 0.68848599 | Inverse variance weighted (multiplicative random effects) | PM20D1 | 1 |
| 0.142028711 | 0.118974427 | 0.232565943 | Inverse variance weighted (multiplicative random effects) | PMM2 | 0.793187763 |
| -0.425866811 | 0.647539054 | 0.5107504 | Wald ratio | PMS1 | 0.956220371 |
| 0.20242107 | 0.092347379 | 0.028382936 | Inverse variance weighted (multiplicative random effects) | PMVK | 0.253375564 |
| 0.120452273 | 0.194027249 | 0.534730691 | Inverse variance weighted (multiplicative random effects) | PNLIPRP1 | 0.971281608 |
| -0.015609961 | 0.042464507 | 0.713171375 | Inverse variance weighted (multiplicative random effects) | PNLIPRP2 | 1 |
| 0.335720807 | 0.575954247 | 0.559964038 | Inverse variance weighted (multiplicative random effects) | PNMA1 | 0.981392512 |
| 0.148371988 | 0.129447838 | 0.251716001 | Inverse variance weighted (multiplicative random effects) | PODXL | 0.805707749 |
| 0.018609733 | 0.084470316 | 0.825629013 | Inverse variance weighted (multiplicative random effects) | PODXL2 | 1 |
| 0.31494272 | 0.196577649 | 0.109127122 | Inverse variance weighted (multiplicative random effects) | POMC | 0.590852599 |
| -0.02372377 | 0.033605096 | 0.480214565 | Inverse variance weighted (multiplicative random effects) | PON1 | 0.940832265 |
| -0.037537574 | 0.064846111 | 0.562675791 | Inverse variance weighted (multiplicative random effects) | PON2 | 0.983500542 |
| 0.101627449 | 0.057543027 | 0.077377005 | Inverse variance weighted (multiplicative random effects) | PON3 | 0.496668857 |
| -0.001083531 | 0.101794874 | 0.991507268 | Inverse variance weighted (multiplicative random effects) | POSTN | 1 |
| -0.031957459 | 0.155257297 | 0.836919431 | Wald ratio | PPBP | 1 |
| 0.081349313 | 0.044602198 | 0.068169349 | Inverse variance weighted (multiplicative random effects) | PPCDC | 0.466783731 |
| 0.570866866 | 0.663204344 | 0.389364347 | Wald ratio | PPIE | 0.91224413 |
| 0.190812537 | 0.349114069 | 0.584679576 | Wald ratio | PPL | 0.989304274 |
| 0.219133207 | 0.493180989 | 0.656806801 | Wald ratio | PPM1F | 0.998066246 |
| -0.938786017 | 0.766564292 | 0.2207007 | Wald ratio | PPME1 | 0.783374499 |
| 0.340080641 | 0.056776857 | 2.10E-09 | Inverse variance weighted (multiplicative random effects) | PPP1R14A | 8.74E-08 |
| -0.259122731 | 0.470821836 | 0.582070694 | Wald ratio | PPP1R14D | 0.989304274 |
| 0.133913067 | 0.410668376 | 0.74435955 | Wald ratio | PPP2R5A | 1 |
| 0.058687632 | 0.082307533 | 0.47582801 | Inverse variance weighted (multiplicative random effects) | PRAP1 | 0.940832265 |
| 1.236920999 | 1.437276525 | 0.389458071 | Wald ratio | PRCP | 0.91224413 |
| -0.09017832 | 0.542531813 | 0.867985732 | Wald ratio | PRDX1 | 1 |
| 0.050655421 | 0.119202833 | 0.670872044 | Inverse variance weighted (multiplicative random effects) | PRDX2 | 1 |
| -0.601497413 | 0.476591698 | 0.206919576 | Wald ratio | PRDX3 | 0.769129582 |
| 0.308268227 | 0.136927999 | 0.024365515 | Inverse variance weighted (multiplicative random effects) | PRDX5 | 0.229322497 |
| 0.40418741 | 0.234778959 | 0.085148216 | Wald ratio | PRDX6 | 0.516101454 |
| 0.129133721 | 0.220778348 | 0.558613539 | Inverse variance weighted (multiplicative random effects) | PRELP | 0.980519968 |
| 0.912845671 | 0.372037301 | 0.014141841 | Wald ratio | PRG2 | 0.157299619 |
| 0.031819652 | 0.0435794 | 0.465296359 | Inverse variance weighted (multiplicative random effects) | PRG3 | 0.940832265 |
| -0.192032157 | 0.083052625 | 0.020768072 | Inverse variance weighted (multiplicative random effects) | PRKAB1 | 0.206687032 |
| 0.313890661 | 0.407405681 | 0.441025794 | Wald ratio | PRKAR2A | 0.934538753 |
| 0.085931384 | 0.732354407 | 0.906593963 | Wald ratio | PRKD2 | 1 |
| -0.483290637 | 0.184614153 | 0.008848783 | Inverse variance weighted (multiplicative random effects) | PRKG1 | 0.109556362 |
| 0.016872902 | 0.090134599 | 0.851506389 | Inverse variance weighted (multiplicative random effects) | PRND | 1 |
| 0.190385826 | 0.163685985 | 0.244782348 | Inverse variance weighted (multiplicative random effects) | PROC | 0.798036494 |
| 0.016283204 | 0.053907521 | 0.762607772 | Inverse variance weighted (multiplicative random effects) | PROCR | 1 |
| -0.210223937 | 0.048312569 | 1.35E-05 | Inverse variance weighted (multiplicative random effects) | PROK1 | 0.000407904 |
| -0.300343361 | 0.225097888 | 0.182112471 | Inverse variance weighted (multiplicative random effects) | PROS1 | 0.741106653 |
| 0.046583541 | 0.061681705 | 0.450114231 | Inverse variance weighted (multiplicative random effects) | PRR4 | 0.938113829 |
| 0.076596362 | 0.073028445 | 0.294244152 | Inverse variance weighted (multiplicative random effects) | PRRT3 | 0.864306126 |
| -0.186280615 | 0.084906584 | 0.028239161 | Inverse variance weighted (multiplicative random effects) | PRSS2 | 0.253178686 |
| 0.07849963 | 0.172865135 | 0.649750309 | Inverse variance weighted (multiplicative random effects) | PRSS22 | 0.998066246 |
| 0.109988983 | 0.150145417 | 0.463833109 | Inverse variance weighted (multiplicative random effects) | PRSS27 | 0.940832265 |
| -0.023420941 | 0.0503008 | 0.641489191 | Inverse variance weighted (multiplicative random effects) | PRSS53 | 0.998066246 |
| -0.05861268 | 0.21453095 | 0.784689361 | Inverse variance weighted (multiplicative random effects) | PRSS8 | 1 |
| -0.021290359 | 0.039946171 | 0.594050043 | Inverse variance weighted (multiplicative random effects) | PRTFDC1 | 0.989304274 |
| 0.119882196 | 0.049629563 | 0.015711895 | Inverse variance weighted (multiplicative random effects) | PRTG | 0.169330273 |
| 0.035662856 | 0.043657182 | 0.413994691 | Inverse variance weighted (multiplicative random effects) | PRTN3 | 0.923405266 |
| -0.079233763 | 0.086923174 | 0.362012056 | Inverse variance weighted (multiplicative random effects) | PSAP | 0.900699853 |
| -0.114516575 | 0.062289216 | 0.065993839 | Inverse variance weighted (multiplicative random effects) | PSAPL1 | 0.462375861 |
| 0.013025699 | 0.04528379 | 0.773617769 | Inverse variance weighted (multiplicative random effects) | PSCA | 1 |
| -0.008004472 | 0.041096297 | 0.845570223 | Inverse variance weighted (multiplicative random effects) | PSG1 | 1 |
| -0.031166161 | 0.152922891 | 0.838507359 | Wald ratio | PSMD9 | 1 |
| 0.145898507 | 0.328371889 | 0.656819367 | Wald ratio | PSME1 | 0.998066246 |
| 0.132114657 | 0.297348754 | 0.656819367 | Wald ratio | PSME2 | 0.998066246 |
| -1.843504348 | 1.334416201 | 0.167123351 | Wald ratio | PSMG3 | 0.712329039 |
| 0.102998313 | 0.281252762 | 0.714206421 | Inverse variance weighted (multiplicative random effects) | PSMG4 | 1 |
| 0.003895664 | 0.089064879 | 0.965111958 | Inverse variance weighted (multiplicative random effects) | PSRC1 | 1 |
| -0.195121865 | 0.192096466 | 0.30974875 | Wald ratio | PSTPIP2 | 0.869694804 |
| 0.131659333 | 0.077357221 | 0.088761809 | Inverse variance weighted (multiplicative random effects) | PTGDS | 0.525995904 |
| -0.916552319 | 0.484954595 | 0.058761232 | Wald ratio | PTGES2 | 0.431167608 |
| -0.007968276 | 0.06772891 | 0.906345338 | Inverse variance weighted (multiplicative random effects) | PTGR1 | 1 |
| -0.05414944 | 0.085643644 | 0.527214028 | Inverse variance weighted (multiplicative random effects) | PTH1R | 0.967500298 |
| -0.258057908 | 0.531227967 | 0.627125843 | Wald ratio | PTK7 | 0.998066246 |
| 0.707242757 | 0.41132818 | 0.085539313 | Wald ratio | PTN | 0.516101454 |
| 0.102465133 | 0.031318259 | 0.001068886 | Inverse variance weighted (multiplicative random effects) | PTPRB | 0.020945345 |
| 0.054408713 | 0.100951043 | 0.589913506 | Inverse variance weighted (multiplicative random effects) | PTPRC | 0.989304274 |
| -0.592698054 | 0.409429724 | 0.147723767 | Wald ratio | PTPRF | 0.673782242 |
| -0.019725235 | 0.048685841 | 0.68536474 | Inverse variance weighted (multiplicative random effects) | PTPRH | 1 |
| -0.001441542 | 0.337200927 | 0.996589034 | Wald ratio | PTPRK | 1 |
| -0.503592151 | 0.496777578 | 0.310717545 | Inverse variance weighted (multiplicative random effects) | PTPRM | 0.86984185 |
| 0.443459957 | 0.578995891 | 0.443728607 | Wald ratio | PTPRN2 | 0.934538753 |
| -0.082842411 | 0.126843246 | 0.51368629 | Inverse variance weighted (multiplicative random effects) | PTPRR | 0.957408138 |
| 0.102569693 | 0.046892953 | 0.028719485 | Inverse variance weighted (multiplicative random effects) | PTPRS | 0.253827031 |
| -0.280777914 | 0.082990227 | 0.000716295 | Inverse variance weighted (multiplicative random effects) | PTPRZ1 | 0.014751428 |
| 0.084483504 | 0.149741953 | 0.572622143 | Inverse variance weighted (multiplicative random effects) | PTS | 0.989304274 |
| -0.683539993 | 0.771193264 | 0.375433952 | Inverse variance weighted (multiplicative random effects) | PTX3 | 0.909083377 |
| 0.012326259 | 0.023869564 | 0.605574592 | Inverse variance weighted (multiplicative random effects) | PVALB | 0.991807207 |
| -0.064193353 | 0.081793144 | 0.432555827 | Inverse variance weighted (multiplicative random effects) | PVR | 0.931458519 |
| -0.068458504 | 0.150840202 | 0.649938328 | Inverse variance weighted (multiplicative random effects) | PXDNL | 0.998066246 |
| -0.162015823 | 0.091612757 | 0.07697979 | Inverse variance weighted (multiplicative random effects) | PXN | 0.496242433 |
| -0.087093316 | 0.056588362 | 0.123787787 | Inverse variance weighted (multiplicative random effects) | PYDC1 | 0.624054668 |
| 0.128402439 | 0.045193093 | 0.004494467 | Inverse variance weighted (multiplicative random effects) | PYY | 0.066197083 |
| -0.00250913 | 0.048360275 | 0.95862104 | Inverse variance weighted (multiplicative random effects) | PZP | 1 |
| 0.185293752 | 0.026237599 | 1.64E-12 | Inverse variance weighted (multiplicative random effects) | QDPR | 8.32E-11 |
| -0.161669589 | 0.059389169 | 0.006484758 | Inverse variance weighted (multiplicative random effects) | QPCT | 0.084301859 |
| 0.039827444 | 0.061810575 | 0.519350591 | Inverse variance weighted (multiplicative random effects) | QSOX1 | 0.964751452 |
| -0.018263614 | 0.049427678 | 0.711753759 | Inverse variance weighted (multiplicative random effects) | RAB11FIP3 | 1 |
| 0.121344948 | 0.199295753 | 0.542611468 | Wald ratio | RAB2B | 0.972958495 |
| -0.023478647 | 0.019978 | 0.239904681 | Inverse variance weighted (multiplicative random effects) | RAB44 | 0.793187763 |
| -1.245397185 | 0.503624717 | 0.013403383 | Wald ratio | RAB6A | 0.151516509 |
| 0.495791896 | 0.417879949 | 0.235446307 | Wald ratio | RABEP1 | 0.793187763 |
| 0.156957211 | 0.171873768 | 0.361130983 | Inverse variance weighted (multiplicative random effects) | RABEPK | 0.900699853 |
| 0.671508859 | 0.409933807 | 0.101402723 | Wald ratio | RABGAP1L | 0.562880046 |
| -0.229935402 | 0.250459757 | 0.358590972 | Wald ratio | RALB | 0.900699853 |
| -1.272509924 | 0.42113776 | 0.002514424 | Wald ratio | RALY | 0.04023079 |
| 0.459498103 | 0.499248457 | 0.357374417 | Wald ratio | RANBP1 | 0.900699853 |
| 0.674324354 | 0.612649207 | 0.271040484 | Wald ratio | RAPGEF2 | 0.838934831 |
| 0.007316656 | 0.09145335 | 0.936233882 | Inverse variance weighted (multiplicative random effects) | RARRES1 | 1 |
| -0.034608514 | 0.148616802 | 0.815861729 | Wald ratio | RARRES2 | 1 |
| 0.0437637 | 0.220015293 | 0.842331518 | Inverse variance weighted (multiplicative random effects) | RASSF2 | 1 |
| 0.008464972 | 0.01925413 | 0.660194606 | Inverse variance weighted (multiplicative random effects) | RBKS | 0.998066246 |
| -0.068081305 | 0.156004197 | 0.662541547 | Wald ratio | RBM17 | 0.998613346 |
| 0.003387207 | 0.264259426 | 0.989773207 | Wald ratio | RBP1 | 1 |
| 0.336422825 | 0.474425193 | 0.478252063 | Wald ratio | RBP2 | 0.940832265 |
| 0.072012283 | 0.155068817 | 0.642368929 | Inverse variance weighted (multiplicative random effects) | RBP5 | 0.998066246 |
| -0.140136752 | 0.056449275 | 0.013045481 | Inverse variance weighted (multiplicative random effects) | RBP7 | 0.148276502 |
| 0.636650528 | 0.399043308 | 0.110613388 | Wald ratio | RBPMS2 | 0.592978989 |
| 0.085514934 | 0.106109736 | 0.420294546 | Inverse variance weighted (multiplicative random effects) | RECK | 0.927204739 |
| 0.102660885 | 0.13876866 | 0.459422102 | Wald ratio | REEP4 | 0.940832265 |
| 0.035690603 | 0.06599117 | 0.588618512 | Inverse variance weighted (multiplicative random effects) | REG1A | 0.989304274 |
| 0.045104261 | 0.034178047 | 0.186940154 | Inverse variance weighted (multiplicative random effects) | REG1B | 0.745928931 |
| -0.004034467 | 0.133545632 | 0.975899252 | Inverse variance weighted (multiplicative random effects) | REG3A | 1 |
| 0.032875195 | 0.100433483 | 0.74341602 | Inverse variance weighted (multiplicative random effects) | REG3G | 1 |
| -0.05225846 | 0.061952697 | 0.398936595 | Inverse variance weighted (multiplicative random effects) | REG4 | 0.917383701 |
| -0.114816295 | 0.063562537 | 0.070863423 | Inverse variance weighted (multiplicative random effects) | RELT | 0.47547071 |
| 0.091767773 | 0.155250922 | 0.554457939 | Inverse variance weighted (multiplicative random effects) | REN | 0.977576895 |
| 0.074171909 | 0.059794674 | 0.214811432 | Inverse variance weighted (multiplicative random effects) | RET | 0.778234308 |
| -0.001724766 | 0.082383273 | 0.983296812 | Inverse variance weighted (multiplicative random effects) | RETN | 1 |
| 0.12937935 | 0.092717345 | 0.162890155 | Inverse variance weighted (multiplicative random effects) | RGMA | 0.701473133 |
| 0.252264593 | 0.117938742 | 0.032440047 | Inverse variance weighted (multiplicative random effects) | RGMB | 0.280100852 |
| -0.080034911 | 0.067886598 | 0.238418052 | Inverse variance weighted (multiplicative random effects) | RIDA | 0.793187763 |
| -0.681072936 | 0.548895287 | 0.214677175 | Wald ratio | RILP | 0.778234308 |
| 0.522953489 | 0.538396126 | 0.33139028 | Wald ratio | RILPL2 | 0.882575905 |
| 0.090175048 | 0.131789314 | 0.493825891 | Inverse variance weighted (multiplicative random effects) | RLN2 | 0.949974212 |
| 0.059198235 | 0.17893661 | 0.740770214 | Inverse variance weighted (multiplicative random effects) | RNASE1 | 1 |
| 0.001554849 | 0.046883781 | 0.97354389 | Inverse variance weighted (multiplicative random effects) | RNASE10 | 1 |
| -0.074372637 | 0.073051425 | 0.308637066 | Inverse variance weighted (multiplicative random effects) | RNASE3 | 0.869694804 |
| -0.119953824 | 0.037496443 | 0.001378707 | Inverse variance weighted (multiplicative random effects) | RNASE4 | 0.025155358 |
| -0.015994882 | 0.057350291 | 0.780323091 | Inverse variance weighted (multiplicative random effects) | RNASE6 | 1 |
| 0.437467884 | 0.301247601 | 0.146449585 | Wald ratio | RNASEH2A | 0.670958453 |
| -0.05032125 | 0.115001855 | 0.661698727 | Inverse variance weighted (multiplicative random effects) | RNASET2 | 0.998066246 |
| -0.098638004 | 0.040155579 | 0.014033841 | Inverse variance weighted (multiplicative random effects) | RNF149 | 0.156937582 |
| -0.361302371 | 0.026106452 | 1.47E-43 | Inverse variance weighted (multiplicative random effects) | RNF43 | 1.70E-41 |
| -0.006743485 | 0.078610058 | 0.931638126 | Inverse variance weighted (multiplicative random effects) | ROBO1 | 1 |
| -0.377435725 | 0.576493664 | 0.512654978 | Wald ratio | ROBO2 | 0.956342919 |
| 0.414417388 | 0.326826302 | 0.204796162 | Wald ratio | ROBO4 | 0.767524355 |
| -0.077587451 | 0.06244921 | 0.214085755 | Inverse variance weighted (multiplicative random effects) | ROR1 | 0.778234308 |
| -0.295317003 | 0.359586397 | 0.411493312 | Wald ratio | RPA2 | 0.923405266 |
| 0.135037208 | 0.502357251 | 0.788078111 | Wald ratio | RPE | 1 |
| -0.008599432 | 0.526468051 | 0.986967778 | Wald ratio | RPL14 | 1 |
| -0.382245386 | 0.580834789 | 0.510476076 | Wald ratio | RRM2 | 0.956220371 |
| -0.021614383 | 0.30313341 | 0.943156447 | Wald ratio | RRM2B | 1 |
| 0.25722549 | 0.137312776 | 0.061029517 | Inverse variance weighted (multiplicative random effects) | RSPO1 | 0.442304515 |
| 0.053939633 | 0.181564783 | 0.766403888 | Wald ratio | RSPO3 | 1 |
| 0.043818437 | 0.041974955 | 0.296523043 | Inverse variance weighted (multiplicative random effects) | RTBDN | 0.865986706 |
| -0.483328385 | 0.317778198 | 0.128269514 | Wald ratio | RTN4IP1 | 0.642610785 |
| 0.08473961 | 0.08122676 | 0.296833708 | Inverse variance weighted (multiplicative random effects) | RTN4R | 0.865986706 |
| -0.051334673 | 0.441456398 | 0.907426825 | Wald ratio | RWDD1 | 1 |
| 0.4055309 | 0.200057007 | 0.042654568 | Wald ratio | S100A11 | 0.35067787 |
| 0.125677864 | 0.01056899 | 1.32E-32 | Inverse variance weighted (multiplicative random effects) | S100A12 | 1.24E-30 |
| -0.261733005 | 0.082869605 | 0.001586532 | Inverse variance weighted (multiplicative random effects) | S100A13 | 0.027965985 |
| 0.727664548 | 0.278845894 | 0.009065926 | Wald ratio | S100A16 | 0.11158063 |
| -0.037703406 | 0.27965832 | 0.892754557 | Inverse variance weighted (multiplicative random effects) | S100A3 | 1 |
| 0.027752974 | 0.136762968 | 0.839191661 | Inverse variance weighted (multiplicative random effects) | S100A4 | 1 |
| -0.233009794 | 0.146901621 | 0.112702526 | Inverse variance weighted (multiplicative random effects) | S100P | 0.594296188 |
| 0.000167791 | 0.075862963 | 0.998235268 | Inverse variance weighted (multiplicative random effects) | SAA4 | 1 |
| 0.032609508 | 0.11234509 | 0.771615824 | Inverse variance weighted (multiplicative random effects) | SAMD9L | 1 |
| -0.209456499 | 0.401621201 | 0.601999357 | Wald ratio | SARG | 0.989848745 |
| -0.078673563 | 0.061193257 | 0.198562658 | Inverse variance weighted (multiplicative random effects) | SAT2 | 0.759210165 |
| 0.04420065 | 0.088742928 | 0.618431051 | Inverse variance weighted (multiplicative random effects) | SBSN | 0.99638775 |
| 0.061255337 | 0.376514079 | 0.870761974 | Wald ratio | SCAMP3 | 1 |
| 0.191482725 | 0.051626347 | 0.00020807 | Inverse variance weighted (multiplicative random effects) | SCARA5 | 0.004924066 |
| -0.05975934 | 0.132548521 | 0.652098535 | Inverse variance weighted (multiplicative random effects) | SCARB2 | 0.998066246 |
| -0.191149176 | 0.314461538 | 0.543279096 | Inverse variance weighted (multiplicative random effects) | SCARF1 | 0.973316555 |
| -0.163804142 | 0.136485316 | 0.230077421 | Inverse variance weighted (multiplicative random effects) | SCARF2 | 0.793187763 |
| -0.009364057 | 0.610423876 | 0.987760728 | Wald ratio | SCG2 | 1 |
| 0.038024939 | 0.088784696 | 0.668445399 | Inverse variance weighted (multiplicative random effects) | SCG3 | 0.999439337 |
| 0.034840187 | 0.067872169 | 0.607727034 | Inverse variance weighted (multiplicative random effects) | SCGB1A1 | 0.992207403 |
| 0.108533962 | 0.147956162 | 0.463220081 | Inverse variance weighted (multiplicative random effects) | SCGB3A1 | 0.940832265 |
| 0.203750469 | 0.079551986 | 0.010430403 | Inverse variance weighted (multiplicative random effects) | SCGB3A2 | 0.126135108 |
| -0.050777636 | 0.370949361 | 0.891121199 | Wald ratio | SCGN | 1 |
| 0.152691167 | 0.081217746 | 0.060105051 | Inverse variance weighted (multiplicative random effects) | SCLY | 0.438661423 |
| -0.031549545 | 0.074354215 | 0.671336726 | Inverse variance weighted (multiplicative random effects) | SCN4B | 1 |
| -0.08799833 | 0.180257878 | 0.625422197 | Inverse variance weighted (multiplicative random effects) | SCPEP1 | 0.998066246 |
| -0.128520732 | 0.233486978 | 0.582017151 | Inverse variance weighted (multiplicative random effects) | SCRN1 | 0.989304274 |
| -0.279717144 | 0.200080153 | 0.162105427 | Inverse variance weighted (multiplicative random effects) | SDC1 | 0.701473133 |
| 0.435256582 | 0.433054883 | 0.31485635 | Inverse variance weighted (multiplicative random effects) | SDC4 | 0.873862934 |
| 0.064037668 | 0.04298229 | 0.136260603 | Inverse variance weighted (multiplicative random effects) | SDCCAG8 | 0.653045329 |
| 0.01122384 | 0.093854968 | 0.904810267 | Inverse variance weighted (multiplicative random effects) | SDK2 | 1 |
| -0.044947334 | 0.275214216 | 0.870268351 | Wald ratio | SEC31A | 1 |
| -0.043684336 | 0.096541251 | 0.650913148 | Wald ratio | SEL1L | 0.998066246 |
| 0.05946914 | 0.44394189 | 0.893436547 | Inverse variance weighted (multiplicative random effects) | SELE | 1 |
| 0.094322281 | 0.303969214 | 0.756331246 | Wald ratio | SELENOP | 1 |
| -0.064644042 | 0.054356387 | 0.234336177 | Inverse variance weighted (multiplicative random effects) | SELL | 0.793187763 |
| 0.184066673 | 0.130683006 | 0.15898384 | Inverse variance weighted (multiplicative random effects) | SELP | 0.698760494 |
| 0.036359256 | 0.053873117 | 0.499735884 | Inverse variance weighted (multiplicative random effects) | SELPLG | 0.956220371 |
| 0.081479653 | 0.17200862 | 0.635717294 | Inverse variance weighted (multiplicative random effects) | SEMA3F | 0.998066246 |
| 0.576152421 | 0.322758324 | 0.074246838 | Wald ratio | SEMA4D | 0.488713364 |
| 0.156300839 | 0.29494995 | 0.59616514 | Wald ratio | SEMA6C | 0.989304274 |
| 0.208771546 | 0.114758145 | 0.068876233 | Inverse variance weighted (multiplicative random effects) | SEMA7A | 0.466783731 |
| 0.19970824 | 0.048574619 | 3.93E-05 | Inverse variance weighted (multiplicative random effects) | SEPTIN8 | 0.001105543 |
| -2.096200317 | 1.590721763 | 0.187581727 | Wald ratio | SEPTIN9 | 0.745928931 |
| -0.083336723 | 0.045445684 | 0.066688826 | Inverse variance weighted (multiplicative random effects) | SERPINA1 | 0.462375861 |
| 0.02865794 | 0.043469862 | 0.509728839 | Inverse variance weighted (multiplicative random effects) | SERPINA11 | 0.956220371 |
| 0.004562232 | 0.088691476 | 0.95897543 | Inverse variance weighted (multiplicative random effects) | SERPINA12 | 1 |
| 0.088150967 | 0.068466622 | 0.197919099 | Inverse variance weighted (multiplicative random effects) | SERPINA3 | 0.759210165 |
| 0.053999244 | 0.063204913 | 0.392909981 | Inverse variance weighted (multiplicative random effects) | SERPINA4 | 0.915857498 |
| -0.098577384 | 0.073624958 | 0.180599127 | Inverse variance weighted (multiplicative random effects) | SERPINA5 | 0.740254766 |
| -0.007878929 | 0.326208841 | 0.98073055 | Inverse variance weighted (multiplicative random effects) | SERPINA6 | 1 |
| -0.06236933 | 0.068149288 | 0.36009356 | Inverse variance weighted (multiplicative random effects) | SERPINA9 | 0.900699853 |
| 0.554651131 | 0.14705933 | 0.000162195 | Inverse variance weighted (multiplicative random effects) | SERPINB1 | 0.003968996 |
| 0.144399523 | 0.542843778 | 0.790234843 | Wald ratio | SERPINB5 | 1 |
| -0.015290514 | 0.177042218 | 0.931175088 | Wald ratio | SERPINB6 | 1 |
| 0.005609359 | 0.022068296 | 0.79935513 | Inverse variance weighted (multiplicative random effects) | SERPINB8 | 1 |
| 0.059505479 | 0.235233385 | 0.800296357 | Inverse variance weighted (multiplicative random effects) | SERPINB9 | 1 |
| -0.090903837 | 0.125034081 | 0.467206066 | Inverse variance weighted (multiplicative random effects) | SERPINC1 | 0.940832265 |
| 0.321886348 | 0.341517219 | 0.345927168 | Inverse variance weighted (multiplicative random effects) | SERPIND1 | 0.897122605 |
| 0.246063635 | 0.313301533 | 0.432225421 | Wald ratio | SERPINE1 | 0.931458519 |
| -0.053911201 | 0.054343272 | 0.321173509 | Inverse variance weighted (multiplicative random effects) | SERPINE2 | 0.873862934 |
| -0.108074406 | 0.082911903 | 0.192409302 | Inverse variance weighted (multiplicative random effects) | SERPINF1 | 0.748540623 |
| 0.171199087 | 0.048526276 | 0.000418765 | Inverse variance weighted (multiplicative random effects) | SERPINF2 | 0.009344213 |
| 0.088551179 | 0.113599226 | 0.435682215 | Inverse variance weighted (multiplicative random effects) | SERPING1 | 0.933284251 |
| 0.07734588 | 0.345674416 | 0.822949071 | Wald ratio | SERPINH1 | 1 |
| 0.108084976 | 0.068839801 | 0.116393182 | Inverse variance weighted (multiplicative random effects) | SERPINI1 | 0.60373521 |
| -0.208323077 | 0.056629848 | 0.000234444 | Inverse variance weighted (multiplicative random effects) | SERPINI2 | 0.005418271 |
| 0.279779823 | 0.295193161 | 0.34323862 | Wald ratio | SESTD1 | 0.897122605 |
| 0.140045738 | 0.042074778 | 0.000873162 | Inverse variance weighted (multiplicative random effects) | SETMAR | 0.017805654 |
| 0.048814081 | 0.461815869 | 0.915820133 | Wald ratio | SEZ6 | 1 |
| -0.018688794 | 0.097356731 | 0.847771959 | Inverse variance weighted (multiplicative random effects) | SEZ6L | 1 |
| 0.053262482 | 0.098611534 | 0.589111338 | Inverse variance weighted (multiplicative random effects) | SEZ6L2 | 0.989304274 |
| 0.709549361 | 0.637564847 | 0.26574906 | Wald ratio | SF3B4 | 0.828722707 |
| 0.004202768 | 0.097087873 | 0.965471722 | Inverse variance weighted (multiplicative random effects) | SFRP1 | 1 |
| -0.131848306 | 0.140942853 | 0.349544327 | Inverse variance weighted (multiplicative random effects) | SFRP4 | 0.898270421 |
| -0.03318097 | 0.029653353 | 0.263156413 | Inverse variance weighted (multiplicative random effects) | SFTPA1 | 0.824345391 |
| -0.092825454 | 0.062923811 | 0.140157721 | Inverse variance weighted (multiplicative random effects) | SFTPA2 | 0.657666752 |
| 0.017612251 | 0.046984275 | 0.707768923 | Inverse variance weighted (multiplicative random effects) | SFTPD | 1 |
| -0.003295666 | 0.040037397 | 0.934396469 | Inverse variance weighted (multiplicative random effects) | SGSH | 1 |
| -0.128998982 | 0.440687326 | 0.769734468 | Wald ratio | SH2B3 | 1 |
| -0.018665666 | 0.110505687 | 0.865866363 | Inverse variance weighted (multiplicative random effects) | SH3BP1 | 1 |
| 0.066991655 | 0.167566039 | 0.689309312 | Inverse variance weighted (multiplicative random effects) | SH3GLB2 | 1 |
| 0.013397009 | 0.062216936 | 0.82951209 | Inverse variance weighted (multiplicative random effects) | SHBG | 1 |
| -0.431988951 | 0.285618515 | 0.13041477 | Inverse variance weighted (multiplicative random effects) | SHISA5 | 0.642802656 |
| -0.081817053 | 0.034413329 | 0.017431318 | Inverse variance weighted (multiplicative random effects) | SHMT1 | 0.182196692 |
| -0.404371547 | 0.397517149 | 0.309037833 | Wald ratio | SHPK | 0.869694804 |
| -0.058308982 | 0.014681129 | 7.14E-05 | Inverse variance weighted (multiplicative random effects) | SIAE | 0.001855431 |
| 0.092859276 | 0.076473644 | 0.224646513 | Inverse variance weighted (multiplicative random effects) | SIGLEC1 | 0.786751592 |
| -0.038377274 | 0.054835099 | 0.484010391 | Inverse variance weighted (multiplicative random effects) | SIGLEC10 | 0.942682873 |
| -0.032856793 | 0.04042118 | 0.416297869 | Inverse variance weighted (multiplicative random effects) | SIGLEC5 | 0.923975972 |
| -0.001744346 | 0.043890287 | 0.968297766 | Inverse variance weighted (multiplicative random effects) | SIGLEC6 | 1 |
| 0.036855484 | 0.076173636 | 0.628502856 | Inverse variance weighted (multiplicative random effects) | SIGLEC7 | 0.998066246 |
| -0.132780793 | 0.040166731 | 0.000947257 | Inverse variance weighted (multiplicative random effects) | SIGLEC8 | 0.018764717 |
| 0.035185047 | 0.044525538 | 0.429398299 | Inverse variance weighted (multiplicative random effects) | SIGLEC9 | 0.931333119 |
| -0.133610989 | 0.483115409 | 0.782117043 | Wald ratio | SIL1 | 1 |
| -0.029367007 | 0.02721391 | 0.28053535 | Inverse variance weighted (multiplicative random effects) | SIRPA | 0.847831902 |
| -0.091707329 | 0.036864126 | 0.012856778 | Inverse variance weighted (multiplicative random effects) | SIRPB1 | 0.147746404 |
| -0.21270631 | 0.215540001 | 0.323714677 | Inverse variance weighted (multiplicative random effects) | SIRT2 | 0.875587163 |
| -0.504940446 | 0.535234423 | 0.345476065 | Wald ratio | SKAP1 | 0.897122605 |
| -0.5007753 | 0.465675993 | 0.282207851 | Inverse variance weighted (multiplicative random effects) | SLA2 | 0.847831902 |
| 0.539707407 | 0.255867068 | 0.034916335 | Wald ratio | SLAMF1 | 0.296432559 |
| -0.211377493 | 0.052247463 | 5.22E-05 | Inverse variance weighted (multiplicative random effects) | SLAMF6 | 0.001409194 |
| -0.005093943 | 0.044043813 | 0.907924958 | Inverse variance weighted (multiplicative random effects) | SLAMF7 | 1 |
| -0.042272633 | 0.031409668 | 0.178351608 | Inverse variance weighted (multiplicative random effects) | SLAMF8 | 0.738570212 |
| -0.308086775 | 0.464349957 | 0.50702337 | Wald ratio | SLC16A1 | 0.956220371 |
| -0.429757639 | 0.50838588 | 0.397922441 | Wald ratio | SLC27A4 | 0.917383701 |
| 0.163871677 | 0.288763825 | 0.570378746 | Wald ratio | SLC39A14 | 0.989304274 |
| 0.137414076 | 0.181852993 | 0.449869418 | Inverse variance weighted (multiplicative random effects) | SLC39A5 | 0.938113829 |
| 0.082747824 | 0.886164484 | 0.925603665 | Wald ratio | SLC4A1 | 1 |
| -0.203594596 | 0.118455326 | 0.085660665 | Inverse variance weighted (multiplicative random effects) | SLC9A3R2 | 0.516101454 |
| 0.4176666 | 0.501571382 | 0.405004823 | Wald ratio | SLIT2 | 0.922595089 |
| -0.025420005 | 0.270961446 | 0.92525683 | Wald ratio | SLITRK1 | 1 |
| -0.075311035 | 0.021360292 | 0.000422287 | Inverse variance weighted (multiplicative random effects) | SLITRK6 | 0.009344213 |
| -0.4603958 | 0.605490598 | 0.447034523 | Wald ratio | SLMAP | 0.935865266 |
| 0.143692798 | 0.102004393 | 0.158926184 | Inverse variance weighted (multiplicative random effects) | SLURP1 | 0.698760494 |
| -0.142223018 | 0.175275692 | 0.417121845 | Inverse variance weighted (multiplicative random effects) | SMAD3 | 0.923975972 |
| -0.180108164 | 0.642712187 | 0.779299944 | Wald ratio | SMARCA2 | 1 |
| 0.012079041 | 0.219923755 | 0.956199198 | Inverse variance weighted (multiplicative random effects) | SMOC1 | 1 |
| -0.05533576 | 0.080312579 | 0.49082019 | Inverse variance weighted (multiplicative random effects) | SMOC2 | 0.947037101 |
| -0.001347308 | 0.213386205 | 0.994962236 | Inverse variance weighted (multiplicative random effects) | SMPD1 | 1 |
| 1.193051053 | 0.62839844 | 0.05762258 | Wald ratio | SMPD3 | 0.429854876 |
| 0.071108968 | 0.032098351 | 0.026736295 | Inverse variance weighted (multiplicative random effects) | SMPDL3A | 0.242844946 |
| 0.15493227 | 0.287549128 | 0.590022944 | Wald ratio | SNAP25 | 0.989304274 |
| 0.131651305 | 0.452226349 | 0.770960947 | Wald ratio | SNAP29 | 1 |
| -0.341429191 | 0.645808003 | 0.597023784 | Wald ratio | SNCA | 0.989304274 |
| 0.067398333 | 0.062953934 | 0.284350391 | Inverse variance weighted (multiplicative random effects) | SNCG | 0.848563578 |
| -0.19418521 | 0.257914214 | 0.451506284 | Inverse variance weighted (multiplicative random effects) | SNED1 | 0.940073144 |
| 0.076216506 | 0.085970264 | 0.375323927 | Inverse variance weighted (multiplicative random effects) | SNX15 | 0.909083377 |
| -0.108264913 | 0.452324912 | 0.810832577 | Wald ratio | SNX18 | 1 |
| 0.03234935 | 0.028832503 | 0.261873027 | Inverse variance weighted (multiplicative random effects) | SNX9 | 0.821562438 |
| 0.438055617 | 0.387562763 | 0.25835698 | Wald ratio | SOD1 | 0.815451469 |
| -0.208108281 | 0.218071804 | 0.339926333 | Inverse variance weighted (multiplicative random effects) | SOD2 | 0.897122605 |
| -0.051383038 | 0.087484944 | 0.556978211 | Inverse variance weighted (multiplicative random effects) | SOD3 | 0.979442202 |
| -0.280310617 | 0.600720883 | 0.640769109 | Wald ratio | SORBS1 | 0.998066246 |
| -0.060981354 | 0.09957999 | 0.540282654 | Inverse variance weighted (multiplicative random effects) | SORCS2 | 0.972958495 |
| -0.182611303 | 0.323874727 | 0.572867698 | Inverse variance weighted (multiplicative random effects) | SORD | 0.989304274 |
| 0.160512314 | 0.120768122 | 0.183816611 | Inverse variance weighted (multiplicative random effects) | SORT1 | 0.741887316 |
| -0.682957145 | 0.530348799 | 0.197832687 | Wald ratio | SOST | 0.759210165 |
| -0.001198729 | 0.087404287 | 0.98905755 | Inverse variance weighted (multiplicative random effects) | SPAG1 | 1 |
| 0.820216492 | 0.69221999 | 0.236054116 | Inverse variance weighted (multiplicative random effects) | SPARC | 0.793187763 |
| 0.00641417 | 0.058414095 | 0.912563891 | Inverse variance weighted (multiplicative random effects) | SPARCL1 | 1 |
| 0.065022046 | 0.061150095 | 0.287637399 | Inverse variance weighted (multiplicative random effects) | SPESP1 | 0.853474736 |
| 0.020393828 | 0.045517105 | 0.654118872 | Inverse variance weighted (multiplicative random effects) | SPINK1 | 0.998066246 |
| -0.01020825 | 0.075392054 | 0.892293885 | Inverse variance weighted (multiplicative random effects) | SPINK2 | 1 |
| 0.011295932 | 0.051891536 | 0.827675703 | Inverse variance weighted (multiplicative random effects) | SPINK4 | 1 |
| -0.05739162 | 0.066340043 | 0.386976877 | Inverse variance weighted (multiplicative random effects) | SPINK5 | 0.91224413 |
| 0.109793098 | 0.15451724 | 0.477359633 | Inverse variance weighted (multiplicative random effects) | SPINK6 | 0.940832265 |
| -0.025435322 | 0.037994476 | 0.503209816 | Inverse variance weighted (multiplicative random effects) | SPINK8 | 0.956220371 |
| -0.412951591 | 0.057291054 | 5.68E-13 | Inverse variance weighted (multiplicative random effects) | SPINT1 | 3.01E-11 |
| 0.399147694 | 0.04601024 | 4.13E-18 | Inverse variance weighted (multiplicative random effects) | SPINT2 | 2.68E-16 |
| 0.813336259 | 0.483209196 | 0.092336966 | Wald ratio | SPINT3 | 0.542544884 |
| 0.072506832 | 0.211797338 | 0.73209457 | Inverse variance weighted (multiplicative random effects) | SPOCK1 | 1 |
| -7.00E-05 | 0.029449416 | 0.998103754 | Inverse variance weighted (multiplicative random effects) | SPON1 | 1 |
| 0.038991879 | 0.098663503 | 0.692694823 | Inverse variance weighted (multiplicative random effects) | SPON2 | 1 |
| 0.618189187 | 0.088921918 | 3.60E-12 | Inverse variance weighted (multiplicative random effects) | SPP1 | 1.78E-10 |
| 0.034438203 | 0.411384144 | 0.933284617 | Wald ratio | SPRED2 | 1 |
| 0.059307465 | 0.05880961 | 0.313231021 | Inverse variance weighted (multiplicative random effects) | SPRR3 | 0.872182762 |
| -0.112098354 | 0.008884751 | 1.70E-36 | Inverse variance weighted (multiplicative random effects) | SPRY2 | 1.86E-34 |
| 0.101796347 | 0.093719059 | 0.277396631 | Inverse variance weighted (multiplicative random effects) | SPTLC1 | 0.847261368 |
| 0.536750851 | 0.537402653 | 0.317897825 | Wald ratio | SRP14 | 0.873862934 |
| -0.070646274 | 0.089653891 | 0.43070318 | Inverse variance weighted (multiplicative random effects) | SSC4D | 0.931458519 |
| -0.123785542 | 0.091755803 | 0.177312587 | Inverse variance weighted (multiplicative random effects) | SSC5D | 0.738570212 |
| -0.814466661 | 0.345977878 | 0.018567595 | Inverse variance weighted (multiplicative random effects) | SSNA1 | 0.189316658 |
| 0.195098921 | 0.537165277 | 0.716454802 | Wald ratio | ST13 | 1 |
| -0.127844639 | 0.089664273 | 0.153921835 | Inverse variance weighted (multiplicative random effects) | ST3GAL1 | 0.689994434 |
| -0.134305734 | 0.073139239 | 0.066312998 | Inverse variance weighted (multiplicative random effects) | ST6GAL1 | 0.462375861 |
| -0.123233959 | 0.100158694 | 0.218552205 | Inverse variance weighted (multiplicative random effects) | STAB2 | 0.780397055 |
| -0.370293297 | 0.524378129 | 0.480090495 | Wald ratio | STAMBP | 0.940832265 |
| -0.181295584 | 0.125334191 | 0.148037733 | Inverse variance weighted (multiplicative random effects) | STAT2 | 0.673782242 |
| 0.669995274 | 0.525239821 | 0.202097386 | Wald ratio | STC1 | 0.767524355 |
| 0.258350635 | 0.056910471 | 5.64E-06 | Inverse variance weighted (multiplicative random effects) | STC2 | 0.000180357 |
| -0.399961243 | 0.541720992 | 0.460322483 | Wald ratio | STK4 | 0.940832265 |
| 0.235431095 | 0.325766844 | 0.469865353 | Inverse variance weighted (multiplicative random effects) | STX16 | 0.940832265 |
| -1.270608031 | 0.691315794 | 0.066068885 | Wald ratio | STX4 | 0.462375861 |
| 0.007141181 | 0.051897585 | 0.890555444 | Inverse variance weighted (multiplicative random effects) | STX7 | 1 |
| 0.457427469 | 0.338671225 | 0.17680646 | Wald ratio | STX8 | 0.738468749 |
| 0.466953736 | 0.561954809 | 0.406004502 | Wald ratio | STXBP1 | 0.922595089 |
| -0.308925282 | 0.026840176 | 1.18E-30 | Inverse variance weighted (multiplicative random effects) | SUGP1 | 1.06E-28 |
| -0.448660056 | 0.281141117 | 0.11052149 | Inverse variance weighted (multiplicative random effects) | SULT1A1 | 0.592978989 |
| 0.107657959 | 0.130358275 | 0.408882318 | Inverse variance weighted (multiplicative random effects) | SULT2A1 | 0.923405266 |
| 0.005740939 | 0.048959192 | 0.906654279 | Inverse variance weighted (multiplicative random effects) | SUMF2 | 1 |
| 1.02323605 | 0.700022178 | 0.143818098 | Inverse variance weighted (multiplicative random effects) | SUOX | 0.66475921 |
| -0.132606066 | 0.153794188 | 0.388560594 | Inverse variance weighted (multiplicative random effects) | SUSD1 | 0.91224413 |
| -0.018916519 | 0.033585288 | 0.573272661 | Inverse variance weighted (multiplicative random effects) | SUSD2 | 0.989304274 |
| -0.046083077 | 0.055923784 | 0.409920475 | Inverse variance weighted (multiplicative random effects) | SUSD4 | 0.923405266 |
| -0.244028342 | 0.062528056 | 9.51E-05 | Inverse variance weighted (multiplicative random effects) | SUSD5 | 0.002412926 |
| 0.22715833 | 0.208490461 | 0.275916601 | Wald ratio | SV2A | 0.845223167 |
| -0.078073568 | 0.0506367 | 0.123113062 | Inverse variance weighted (multiplicative random effects) | SWAP70 | 0.623053939 |
| -0.675019844 | 0.588893653 | 0.251691378 | Wald ratio | TAB2 | 0.805707749 |
| 0.091645788 | 0.104040328 | 0.378389308 | Inverse variance weighted (multiplicative random effects) | TACC3 | 0.909884116 |
| 0.004950549 | 0.048879401 | 0.919327488 | Inverse variance weighted (multiplicative random effects) | TACSTD2 | 1 |
| -0.199005832 | 0.082985958 | 0.01648188 | Inverse variance weighted (multiplicative random effects) | TAFA5 | 0.175806724 |
| -0.486885957 | 0.067572852 | 5.79E-13 | Inverse variance weighted (multiplicative random effects) | TALDO1 | 3.01E-11 |
| -1.370851657 | 0.63398395 | 0.030596498 | Wald ratio | TANK | 0.268526229 |
| 0.163116243 | 0.073182413 | 0.025820592 | Inverse variance weighted (multiplicative random effects) | TBC1D17 | 0.236593975 |
| 0.033198796 | 0.185246584 | 0.857769611 | Wald ratio | TBC1D23 | 1 |
| 0.01015402 | 0.224658905 | 0.96394989 | Wald ratio | TBCA | 1 |
| 0.171388345 | 0.63650872 | 0.787727184 | Wald ratio | TBCB | 1 |
| -0.00045948 | 0.155763079 | 0.997646354 | Inverse variance weighted (multiplicative random effects) | TBCC | 1 |
| 0.050070742 | 0.094031927 | 0.594389011 | Inverse variance weighted (multiplicative random effects) | TCL1A | 0.989304274 |
| 0.005004177 | 0.060059767 | 0.933597136 | Inverse variance weighted (multiplicative random effects) | TCN1 | 1 |
| 0.083491488 | 0.058817644 | 0.155754074 | Inverse variance weighted (multiplicative random effects) | TCN2 | 0.69521132 |
| 0.682988902 | 0.560859062 | 0.223317107 | Wald ratio | TCOF1 | 0.7859553 |
| 0.107714618 | 0.071901885 | 0.134113038 | Inverse variance weighted (multiplicative random effects) | TCTN3 | 0.647227654 |
| 0.047017061 | 0.027273141 | 0.084719981 | Inverse variance weighted (multiplicative random effects) | TDGF1 | 0.516101454 |
| -0.295700444 | 0.044763133 | 3.95E-11 | Inverse variance weighted (multiplicative random effects) | TDP1 | 1.79E-09 |
| -1.099454479 | 0.221693764 | 7.07E-07 | Inverse variance weighted (multiplicative random effects) | TDRKH | 2.68E-05 |
| -0.539952379 | 0.396782829 | 0.173568692 | Wald ratio | TEF | 0.72933915 |
| -0.040828652 | 0.079299275 | 0.60664533 | Inverse variance weighted (multiplicative random effects) | TEK | 0.991998653 |
| 0.095977601 | 0.095919237 | 0.317016134 | Inverse variance weighted (multiplicative random effects) | TEX101 | 0.873862934 |
| -0.146570685 | 0.057596554 | 0.010934592 | Inverse variance weighted (multiplicative random effects) | TF | 0.130712361 |
| 0.120641442 | 0.168488779 | 0.473978609 | Inverse variance weighted (multiplicative random effects) | TFF1 | 0.940832265 |
| -0.15392442 | 0.115444322 | 0.182426253 | Inverse variance weighted (multiplicative random effects) | TFF2 | 0.741106653 |
| 0.130095609 | 0.104252467 | 0.212071352 | Inverse variance weighted (multiplicative random effects) | TFF3 | 0.77251911 |
| 0.039032674 | 0.065634316 | 0.552044566 | Inverse variance weighted (multiplicative random effects) | TFPI | 0.977576895 |
| 0.375667185 | 0.19660589 | 0.056035094 | Inverse variance weighted (multiplicative random effects) | TFPI2 | 0.423829077 |
| -0.032900821 | 0.095145074 | 0.729495756 | Inverse variance weighted (multiplicative random effects) | TFRC | 1 |
| -0.333156396 | 0.49279209 | 0.499003357 | Inverse variance weighted (multiplicative random effects) | TG | 0.956220371 |
| -0.229619282 | 0.263921108 | 0.384284009 | Wald ratio | TGFA | 0.91151664 |
| 0.131916719 | 0.139389483 | 0.343950047 | Inverse variance weighted (multiplicative random effects) | TGFB1 | 0.897122605 |
| 0.17364604 | 0.204591435 | 0.396022974 | Wald ratio | TGFB2 | 0.91729152 |
| -0.092911357 | 0.066643885 | 0.163273289 | Inverse variance weighted (multiplicative random effects) | TGFBI | 0.701670331 |
| 0.351817258 | 0.561306022 | 0.530801272 | Wald ratio | TGFBR1 | 0.971034868 |
| 0.401541835 | 0.486815892 | 0.409466383 | Inverse variance weighted (multiplicative random effects) | TGFBR2 | 0.923405266 |
| 0.225110841 | 0.11912432 | 0.058796282 | Inverse variance weighted (multiplicative random effects) | TGFBR3 | 0.431167608 |
| -0.023286871 | 0.170079768 | 0.891096127 | Wald ratio | TGM2 | 1 |
| 0.083355844 | 0.06477341 | 0.198134831 | Inverse variance weighted (multiplicative random effects) | TGOLN2 | 0.759210165 |
| -0.028860072 | 0.047304833 | 0.541804062 | Inverse variance weighted (multiplicative random effects) | THBD | 0.972958495 |
| -0.000254084 | 0.056966461 | 0.99644126 | Inverse variance weighted (multiplicative random effects) | THBS2 | 1 |
| -0.243268387 | 0.044316806 | 4.04E-08 | Inverse variance weighted (multiplicative random effects) | THBS4 | 1.61E-06 |
| -0.026961746 | 0.089821225 | 0.764046567 | Inverse variance weighted (multiplicative random effects) | THOP1 | 1 |
| -0.045171499 | 0.472232223 | 0.92379437 | Wald ratio | THPO | 1 |
| 0.047521262 | 0.201800519 | 0.813831293 | Inverse variance weighted (multiplicative random effects) | THSD1 | 1 |
| 0.063701521 | 0.013327387 | 1.76E-06 | Inverse variance weighted (multiplicative random effects) | THTPA | 6.08E-05 |
| 0.088319614 | 0.053025586 | 0.095792365 | Inverse variance weighted (multiplicative random effects) | THY1 | 0.554700584 |
| -0.076702653 | 0.157274082 | 0.625761975 | Wald ratio | TIE1 | 0.998066246 |
| 0.747330305 | 0.471158841 | 0.112704464 | Wald ratio | TIGAR | 0.594296188 |
| -0.358713939 | 0.349729251 | 0.305037536 | Wald ratio | TIGIT | 0.869694804 |
| 0.08364068 | 0.094860667 | 0.377927485 | Inverse variance weighted (multiplicative random effects) | TIMD4 | 0.909825428 |
| -0.063546748 | 0.169266289 | 0.70734463 | Wald ratio | TIMM10 | 1 |
| -0.169745601 | 0.3923695 | 0.66529325 | Inverse variance weighted (multiplicative random effects) | TIMP2 | 0.999439337 |
| 0.013440597 | 0.08777467 | 0.878298799 | Inverse variance weighted (multiplicative random effects) | TIMP3 | 1 |
| -0.034182384 | 0.102870387 | 0.739673349 | Inverse variance weighted (multiplicative random effects) | TIMP4 | 1 |
| 0.200288398 | 0.367307321 | 0.585555225 | Inverse variance weighted (multiplicative random effects) | TINAGL1 | 0.989304274 |
| -0.8691645 | 1.103379276 | 0.430854896 | Wald ratio | TJAP1 | 0.931458519 |
| 0.201642243 | 0.214695908 | 0.347628435 | Wald ratio | TK1 | 0.897122605 |
| 0.058190544 | 0.057652289 | 0.312813412 | Inverse variance weighted (multiplicative random effects) | TLR1 | 0.872182762 |
| -0.065150485 | 0.10813051 | 0.546829963 | Inverse variance weighted (multiplicative random effects) | TLR3 | 0.975364036 |
| 0.19744144 | 0.097627476 | 0.043135434 | Inverse variance weighted (multiplicative random effects) | TLR4 | 0.351441367 |
| -0.534031975 | 0.435822289 | 0.220445854 | Wald ratio | TMED8 | 0.783374499 |
| -0.203043637 | 0.28182676 | 0.471244594 | Wald ratio | TMEM106A | 0.940832265 |
| 0.025501271 | 0.04867295 | 0.600326478 | Inverse variance weighted (multiplicative random effects) | TMEM132A | 0.989304274 |
| -0.031075947 | 0.079972123 | 0.697583348 | Inverse variance weighted (multiplicative random effects) | TMEM25 | 1 |
| -0.322186444 | 0.107184522 | 0.002647921 | Inverse variance weighted (multiplicative random effects) | TMPRSS11D | 0.041904104 |
| 0.00268403 | 0.0775995 | 0.972408085 | Inverse variance weighted (multiplicative random effects) | TMPRSS15 | 1 |
| 0.020975049 | 0.049886358 | 0.674151926 | Inverse variance weighted (multiplicative random effects) | TMPRSS5 | 1 |
| -0.452394813 | 0.492877065 | 0.358689329 | Wald ratio | TMSB10 | 0.900699853 |
| -0.106219381 | 0.121033425 | 0.380158824 | Inverse variance weighted (multiplicative random effects) | TNC | 0.911419519 |
| 0.214812379 | 0.268180404 | 0.423131915 | Inverse variance weighted (multiplicative random effects) | TNF | 0.92774274 |
| 0.138161246 | 0.26058094 | 0.595969994 | Inverse variance weighted (multiplicative random effects) | TNF | 0.989304274 |
| -0.272053961 | 0.297259045 | 0.36008226 | Wald ratio | TNF | 0.900699853 |
| 0.568895088 | 0.209503207 | 0.006618615 | Inverse variance weighted (multiplicative random effects) | TNF | 0.085507567 |
| -0.095969078 | 0.082117468 | 0.242532412 | Inverse variance weighted (multiplicative random effects) | TNFAIP2 | 0.793187763 |
| -0.030397467 | 0.346217302 | 0.930036585 | Wald ratio | TNFAIP8 | 1 |
| 0.736303371 | 0.499805732 | 0.140702743 | Wald ratio | TNFAIP8L2 | 0.657666752 |
| 0.109491443 | 0.034098453 | 0.001322563 | Inverse variance weighted (multiplicative random effects) | TNFRSF10A | 0.024708167 |
| -0.005805557 | 0.066014141 | 0.929921069 | Inverse variance weighted (multiplicative random effects) | TNFRSF10B | 1 |
| 0.03128813 | 0.100624686 | 0.755847063 | Inverse variance weighted (multiplicative random effects) | TNFRSF10C | 1 |
| 0.045525673 | 0.072078802 | 0.527641927 | Inverse variance weighted (multiplicative random effects) | TNFRSF11A | 0.967500298 |
| 0.164414852 | 0.144276804 | 0.254461636 | Inverse variance weighted (multiplicative random effects) | TNFRSF11B | 0.808061378 |
| 0.092026433 | 0.335917001 | 0.784118539 | Inverse variance weighted (multiplicative random effects) | TNFRSF12A | 1 |
| 0.077091977 | 0.39201781 | 0.844098097 | Inverse variance weighted (multiplicative random effects) | TNFRSF13B | 1 |
| -0.025540258 | 0.088684382 | 0.773354028 | Inverse variance weighted (multiplicative random effects) | TNFRSF13C | 1 |
| 0.051070439 | 0.248948551 | 0.837459189 | Wald ratio | TNFRSF14 | 1 |
| -0.236884396 | 0.207544169 | 0.25371657 | Wald ratio | TNFRSF17 | 0.807778299 |
| -0.085255813 | 0.064860294 | 0.188693782 | Inverse variance weighted (multiplicative random effects) | TNFRSF19 | 0.747586793 |
| -0.24749211 | 0.441510082 | 0.575098733 | Wald ratio | TNFRSF1A | 0.989304274 |
| -0.057094979 | 0.225094761 | 0.799767021 | Inverse variance weighted (multiplicative random effects) | TNFRSF1B | 1 |
| 0.462218543 | 0.171178383 | 0.006929455 | Inverse variance weighted (multiplicative random effects) | TNFRSF21 | 0.088424949 |
| 0.081162041 | 0.07422406 | 0.274185968 | Inverse variance weighted (multiplicative random effects) | TNFRSF4 | 0.84116049 |
| -0.260423446 | 0.081915268 | 0.00147692 | Inverse variance weighted (multiplicative random effects) | TNFRSF6B | 0.026482695 |
| -0.132928543 | 0.016545637 | 9.43E-16 | Inverse variance weighted (multiplicative random effects) | TNFRSF8 | 5.16E-14 |
| -0.001991504 | 0.164766965 | 0.990356372 | Wald ratio | TNFRSF9 | 1 |
| -0.041464337 | 0.099741664 | 0.677616869 | Inverse variance weighted (multiplicative random effects) | TNFSF10 | 1 |
| -0.176445716 | 0.261249721 | 0.499427368 | Wald ratio | TNFSF11 | 0.956220371 |
| -0.11127703 | 0.060957029 | 0.067925679 | Inverse variance weighted (multiplicative random effects) | TNFSF12 | 0.466783731 |
| 0.10317373 | 0.03033434 | 0.000670861 | Inverse variance weighted (multiplicative random effects) | TNFSF13 | 0.013953906 |
| 0.04460849 | 0.130793163 | 0.733057412 | Inverse variance weighted (multiplicative random effects) | TNFSF13B | 1 |
| 0.081475231 | 0.057607961 | 0.157272345 | Inverse variance weighted (multiplicative random effects) | TNFSF14 | 0.698760494 |
| 0.031812075 | 0.089117214 | 0.721115082 | Inverse variance weighted (multiplicative random effects) | TNFSF8 | 1 |
| 0.033257441 | 0.045290951 | 0.462761943 | Inverse variance weighted (multiplicative random effects) | TNN | 0.940832265 |
| 0.013890744 | 0.093133179 | 0.881435864 | Inverse variance weighted (multiplicative random effects) | TNR | 1 |
| 0.287913373 | 0.07763188 | 0.000208326 | Inverse variance weighted (multiplicative random effects) | TNXB | 0.004924066 |
| 0.432198382 | 0.358953413 | 0.228569688 | Wald ratio | TOP2B | 0.791745345 |
| -0.102955397 | 0.054058904 | 0.056844581 | Inverse variance weighted (multiplicative random effects) | TOR1AIP1 | 0.426847399 |
| -0.217937199 | 0.491917659 | 0.657739744 | Wald ratio | TP53 | 0.998066246 |
| 0.090542454 | 0.05717383 | 0.113276902 | Inverse variance weighted (multiplicative random effects) | TP53I3 | 0.594296188 |
| -0.111712782 | 0.090441343 | 0.216757579 | Inverse variance weighted (multiplicative random effects) | TPK1 | 0.778234308 |
| 0.034144105 | 0.075052952 | 0.649157054 | Inverse variance weighted (multiplicative random effects) | TPMT | 0.998066246 |
| 0.019084027 | 0.107546962 | 0.85915626 | Inverse variance weighted (multiplicative random effects) | TPP1 | 1 |
| 0.459764989 | 0.361651218 | 0.20362411 | Wald ratio | TPPP3 | 0.767524355 |
| 0.490411396 | 0.436871849 | 0.261627784 | Wald ratio | TPR | 0.821562438 |
| -0.014402323 | 0.038665409 | 0.709531033 | Inverse variance weighted (multiplicative random effects) | TPSAB1 | 1 |
| -0.08557693 | 0.023359927 | 0.000248882 | Inverse variance weighted (multiplicative random effects) | TPSD1 | 0.005688736 |
| -0.255646722 | 0.527817562 | 0.628139801 | Wald ratio | TRAF2 | 0.998066246 |
| -0.047448123 | 0.066035964 | 0.472437076 | Inverse variance weighted (multiplicative random effects) | TRDMT1 | 0.940832265 |
| -0.012222424 | 0.04213258 | 0.771744055 | Inverse variance weighted (multiplicative random effects) | TREH | 1 |
| -0.067646213 | 0.068384249 | 0.322561624 | Inverse variance weighted (multiplicative random effects) | TREM2 | 0.874789851 |
| 0.154718169 | 0.069067341 | 0.025084039 | Inverse variance weighted (multiplicative random effects) | TREML2 | 0.231888001 |
| -0.26729628 | 0.45105974 | 0.553451434 | Inverse variance weighted (multiplicative random effects) | TRIM21 | 0.977576895 |
| -0.06293333 | 0.32293321 | 0.845486634 | Wald ratio | TRIM25 | 1 |
| 0.004992587 | 0.065189748 | 0.938953329 | Inverse variance weighted (multiplicative random effects) | TRIM40 | 1 |
| 0.159632378 | 0.049884375 | 0.001374049 | Inverse variance weighted (multiplicative random effects) | TRIM5 | 0.025155358 |
| -0.192531881 | 0.107391703 | 0.073004855 | Inverse variance weighted (multiplicative random effects) | TRIM58 | 0.485144086 |
| -0.105625816 | 0.642273569 | 0.869372092 | Wald ratio | TSC22D1 | 1 |
| -0.339790184 | 0.674150018 | 0.614242015 | Wald ratio | TSPAN1 | 0.994282983 |
| -0.95786847 | 0.519198249 | 0.065052193 | Wald ratio | TSPAN15 | 0.461803964 |
| 0.037514363 | 0.088645281 | 0.67215198 | Inverse variance weighted (multiplicative random effects) | TSPAN8 | 1 |
| 0.115376859 | 0.027979588 | 3.73E-05 | Inverse variance weighted (multiplicative random effects) | TSPYL1 | 0.001062746 |
| 0.003466236 | 0.073218534 | 0.962241481 | Inverse variance weighted (multiplicative random effects) | TST | 1 |
| 0.01705075 | 0.061927579 | 0.783059865 | Inverse variance weighted (multiplicative random effects) | TTF2 | 1 |
| 0.119158165 | 0.059891947 | 0.046640268 | Inverse variance weighted (multiplicative random effects) | TTR | 0.374562767 |
| 0.095175773 | 0.555555992 | 0.863975099 | Wald ratio | TXN | 1 |
| 0.009719443 | 0.039955465 | 0.807806389 | Inverse variance weighted (multiplicative random effects) | TXNDC15 | 1 |
| 0.142158019 | 0.302911534 | 0.638851356 | Inverse variance weighted (multiplicative random effects) | TXNDC5 | 0.998066246 |
| 1.962530123 | 0.611507439 | 0.00133044 | Wald ratio | TXNDC9 | 0.024708167 |
| -0.643807474 | 0.585477949 | 0.271494588 | Wald ratio | TXNRD1 | 0.839091744 |
| 0.086188354 | 0.163904456 | 0.598995839 | Wald ratio | TYMP | 0.989304274 |
| 0.114326301 | 0.097820706 | 0.242511161 | Inverse variance weighted (multiplicative random effects) | TYRO3 | 0.793187763 |
| 0.050346487 | 0.374830573 | 0.893151132 | Wald ratio | TYRP1 | 1 |
| -0.267274816 | 0.286573638 | 0.350997262 | Wald ratio | UBAC1 | 0.898859417 |
| 0.118430968 | 0.114532489 | 0.301118309 | Inverse variance weighted (multiplicative random effects) | UBE2L6 | 0.869694804 |
| -0.060474076 | 0.438159525 | 0.890225877 | Wald ratio | UBXN1 | 1 |
| 0.070573721 | 0.077147107 | 0.360299713 | Inverse variance weighted (multiplicative random effects) | ULBP2 | 0.900699853 |
| 0.035353119 | 0.042001154 | 0.399945934 | Inverse variance weighted (multiplicative random effects) | UMOD | 0.917383701 |
| 0.235084923 | 0.561463311 | 0.675435091 | Inverse variance weighted (multiplicative random effects) | UNC5D | 1 |
| 0.754608502 | 0.640920568 | 0.23904297 | Wald ratio | UNG | 0.793187763 |
| -0.255519082 | 0.105611078 | 0.015544662 | Inverse variance weighted (multiplicative random effects) | UPB1 | 0.1684005 |
| 0.054185235 | 0.100977467 | 0.59153898 | Inverse variance weighted (multiplicative random effects) | UROD | 0.989304274 |
| -0.525313808 | 0.288776042 | 0.068895483 | Wald ratio | UROS | 0.466783731 |
| -0.916380758 | 0.088776236 | 5.58E-25 | Inverse variance weighted (multiplicative random effects) | USP28 | 4.47E-23 |
| -0.614426013 | 0.525647589 | 0.242446571 | Wald ratio | USP8 | 0.793187763 |
| -0.241901126 | 0.107971123 | 0.025063391 | Inverse variance weighted (multiplicative random effects) | UXS1 | 0.231888001 |
| -0.273718329 | 0.359915699 | 0.446951719 | Wald ratio | VAMP5 | 0.935865266 |
| 0.025203457 | 0.181753519 | 0.889712244 | Wald ratio | VAMP8 | 1 |
| 0.075072679 | 0.225986954 | 0.739738867 | Inverse variance weighted (multiplicative random effects) | VASH1 | 1 |
| -0.01782831 | 0.097516026 | 0.854935794 | Inverse variance weighted (multiplicative random effects) | VASN | 1 |
| -0.149734404 | 0.208134995 | 0.471888317 | Wald ratio | VAT1 | 0.940832265 |
| -0.225693759 | 0.523476445 | 0.666363627 | Wald ratio | VAV3 | 0.999439337 |
| -0.287872222 | 0.008966904 | 3.89E-226 | Inverse variance weighted (multiplicative random effects) | VCAM1 | 1.16E-223 |
| 0.055676717 | 0.038991664 | 0.153316801 | Inverse variance weighted (multiplicative random effects) | VCAN | 0.689161822 |
| 0.10717883 | 0.369108799 | 0.771531724 | Inverse variance weighted (multiplicative random effects) | VCPKMT | 1 |
| -0.03305072 | 0.036040752 | 0.359123003 | Inverse variance weighted (multiplicative random effects) | VEGFA | 0.900699853 |
| 0.221646286 | 0.143465947 | 0.122360767 | Inverse variance weighted (multiplicative random effects) | VEGFB | 0.623053939 |
| 0.087933813 | 0.18150647 | 0.628054801 | Inverse variance weighted (multiplicative random effects) | VEGFC | 0.998066246 |
| 0.267059611 | 0.22812387 | 0.241728206 | Inverse variance weighted (multiplicative random effects) | VGF | 0.793187763 |
| -0.083025258 | 0.062550899 | 0.184401799 | Inverse variance weighted (multiplicative random effects) | VIT | 0.741887316 |
| -0.099212999 | 0.049085073 | 0.043254322 | Inverse variance weighted (multiplicative random effects) | VMO1 | 0.351441367 |
| -0.035984326 | 0.05646255 | 0.523920881 | Inverse variance weighted (multiplicative random effects) | VNN1 | 0.967500298 |
| 0.054176441 | 0.046757975 | 0.246596145 | Inverse variance weighted (multiplicative random effects) | VNN2 | 0.799734834 |
| 0.390445452 | 0.70647506 | 0.580491454 | Wald ratio | VPS4B | 0.989304274 |
| 0.034670765 | 0.044783729 | 0.43882307 | Inverse variance weighted (multiplicative random effects) | VSIG10 | 0.934538753 |
| 0.236199957 | 0.063841545 | 0.000215783 | Inverse variance weighted (multiplicative random effects) | VSIG10L | 0.00504301 |
| -0.052528612 | 0.16945264 | 0.756568612 | Inverse variance weighted (multiplicative random effects) | VSIG2 | 1 |
| 0.008018616 | 0.113278588 | 0.943567526 | Inverse variance weighted (multiplicative random effects) | VSIR | 1 |
| 0.149031402 | 0.000639512 | 0 | Inverse variance weighted (multiplicative random effects) | VSNL1 | 0 |
| 0.0436648 | 0.066173367 | 0.509347145 | Inverse variance weighted (multiplicative random effects) | VSTM1 | 0.956220371 |
| 0.280848105 | 0.14300119 | 0.049535146 | Inverse variance weighted (multiplicative random effects) | VSTM2L | 0.389288546 |
| 0.123602853 | 0.460324277 | 0.788304803 | Wald ratio | VTCN1 | 1 |
| 0.113978623 | 0.620025649 | 0.854147696 | Wald ratio | VTI1A | 1 |
| -0.041329724 | 0.067131857 | 0.538125576 | Inverse variance weighted (multiplicative random effects) | VWA1 | 0.972459772 |
| -0.052697202 | 0.059973861 | 0.379580504 | Inverse variance weighted (multiplicative random effects) | VWC2 | 0.911419519 |
| -0.265300653 | 0.481817693 | 0.581891073 | Inverse variance weighted (multiplicative random effects) | VWC2L | 0.989304274 |
| -0.233831751 | 0.129927885 | 0.071907369 | Inverse variance weighted (multiplicative random effects) | VWF | 0.48092388 |
| 0.060440931 | 0.090941249 | 0.506296683 | Inverse variance weighted (multiplicative random effects) | WARS | 0.956220371 |
| -0.013146991 | 0.06781788 | 0.846287624 | Inverse variance weighted (multiplicative random effects) | WASF1 | 1 |
| 0.440745091 | 0.439660407 | 0.31611805 | Wald ratio | WASF3 | 0.873862934 |
| -0.271048065 | 0.311566907 | 0.384326968 | Wald ratio | WASHC3 | 0.91151664 |
| -0.095616048 | 0.040032268 | 0.016918487 | Inverse variance weighted (multiplicative random effects) | WFDC1 | 0.17954313 |
| 0.129478567 | 0.060086785 | 0.031172846 | Inverse variance weighted (multiplicative random effects) | WFDC12 | 0.271295065 |
| 0.564698374 | 0.320243753 | 0.077843292 | Wald ratio | WFDC2 | 0.496668857 |
| 0.212487086 | 0.077452336 | 0.006079617 | Inverse variance weighted (multiplicative random effects) | WFIKKN1 | 0.081584539 |
| -0.102295572 | 0.099649271 | 0.304629511 | Inverse variance weighted (multiplicative random effects) | WFIKKN2 | 0.869694804 |
| -0.248191638 | 0.023279037 | 1.54E-26 | Inverse variance weighted (multiplicative random effects) | WIF1 | 1.28E-24 |
| -0.096931414 | 0.095828762 | 0.311774081 | Inverse variance weighted (multiplicative random effects) | WNT9A | 0.871626462 |
| 0.011894403 | 0.176424954 | 0.946248104 | Wald ratio | WWP2 | 1 |
| 0.025851869 | 0.050662795 | 0.609860038 | Inverse variance weighted (multiplicative random effects) | XCL1 | 0.99391765 |
| 0.023092244 | 0.349540271 | 0.947326382 | Wald ratio | XRCC4 | 1 |
| 0.419588146 | 0.458953351 | 0.360596891 | Wald ratio | YAP1 | 0.900699853 |
| -0.307632578 | 0.384128692 | 0.423213794 | Wald ratio | YES1 | 0.92774274 |
| 0.937063808 | 0.536959049 | 0.080962093 | Wald ratio | YJU2 | 0.511857606 |
| 0.146575738 | 0.066627657 | 0.027812333 | Inverse variance weighted (multiplicative random effects) | YOD1 | 0.2504314 |
| 0.077730581 | 0.082688349 | 0.347195642 | Inverse variance weighted (multiplicative random effects) | ZBP1 | 0.897122605 |
| 0.018538331 | 0.112517822 | 0.869133625 | Inverse variance weighted (multiplicative random effects) | ZBTB16 | 1 |
| -0.600040426 | 0.620714012 | 0.333697097 | Wald ratio | ZBTB17 | 0.88758307 |
| 0.038750912 | 0.091939849 | 0.673403784 | Inverse variance weighted (multiplicative random effects) | ZFYVE19 | 1 |
| 0.029521629 | 0.04291731 | 0.491533197 | Inverse variance weighted (multiplicative random effects) | ZP3 | 0.947533873 |
| 0.674669229 | 0.563610376 | 0.231287462 | Wald ratio | ZPR1 | 0.793187763 |
