# Supplementary material for: Blood metabolites as mediators in erectile dysfunction: insights from a multi-center proteomics and genetic study
Source: Front Pharmacol. 2025 Jun 2;16:1568780. doi: 10.3389/fphar.2025.1568780 (PMC12171135; doi:10.3389/fphar.2025.1568780)
Supplement: Supplementary file 5 [file Supplementaryfile4.docx]

| Table. | | | | | |
| --- | --- | --- | --- | --- | --- |
| Gene | b_SMR | se_SMR | p_SMR | p_HEIDI | nsnp_HEIDI |
| AGRN | 0.0443358 | 0.078146 | 0.5704793 | 0.2310702 | 20 |
| TNFRSF4 | 0.0952924 | 0.288789 | 0.7414211 | 0.9983928 | 20 |
| CPTP | 0.0164346 | 0.518285 | 0.9747036 | 0.2394735 | 20 |
| MXRA8 | 0.0500925 | 0.463305 | 0.9139005 | 0.5263463 | 20 |
| VWA1 | -0.100943 | 0.104719 | 0.3350778 | 0.3371389 | 20 |
| NADK | 0.0467498 | 0.114058 | 0.6818963 | 0.7768529 | 20 |
| TNFRSF14 | 0.0510704 | 0.24897 | 0.8374732 | 0.7349436 | 20 |
| PER3 | -0.00931784 | 0.077583 | 0.9044026 | 0.316591 | 20 |
| TNFRSF9 | -0.0019915 | 0.164767 | 0.9903564 | 0.4892108 | 20 |
| PARK7 | -0.0245939 | 0.154982 | 0.8739135 | 0.46672 | 20 |
| ENO1 | -0.600357 | 0.327574 | 0.06684197 | 0.9836483 | 4 |
| CA6 | -0.100841 | 0.0465996 | 0.03046524 | 0.4856705 | 20 |
| NMNAT1 | 0.784032 | 0.632135 | 0.2148674 | 0.7107269 | 18 |
| RBP7 | -0.0816239 | 0.104794 | 0.4360404 | 0.3070741 | 20 |
| PGD | 0.0820806 | 0.0805244 | 0.3080488 | 0.6459311 | 20 |
| ANGPTL7 | 0.0772102 | 0.194888 | 0.6919747 | 0.3749389 | 10 |
| DRAXIN | -0.0969081 | 0.115054 | 0.3996283 | 0.6644045 | 20 |
| NPPB | -0.061788 | 0.161772 | 0.7025027 | 0.9181472 | 20 |
| NTproBNP | -0.0550882 | 0.113249 | 0.6266601 | 0.7741378 | 20 |
| TNFRSF8 | -0.142155 | 0.18766 | 0.4487423 | 0.4303953 | 20 |
| TNFRSF1B | 0.0351974 | 0.245286 | 0.8858991 | 0.2223607 | 20 |
| LRRC38 | 0.00999098 | 0.0866166 | 0.9081699 | 0.7387258 | 20 |
| CTRC | -0.00930882 | 0.0789779 | 0.9061738 | 0.6520001 | 20 |
| CELA2A | -0.629848 | 0.50544 | 0.2127135 | 0.5821175 | 20 |
| CASP9 | -0.388585 | 0.36225 | 0.2834067 | 0.3546232 | 20 |
| DDI2 | -0.820555 | 0.910879 | 0.3676741 | 0.1540215 | 18 |
| ZBTB17 | -0.60004 | 0.628434 | 0.3396696 | 0.606023 | 20 |
| EPHA2 | -0.0700014 | 0.231614 | 0.7624742 | 0.9862479 | 20 |
| PADI2 | -0.107433 | 0.235677 | 0.6484972 | 0.3095035 | 20 |
| PADI4 | -0.148869 | 0.112859 | 0.1871428 | 0.6787648 | 20 |
| IGSF21 | 0.104082 | 0.208799 | 0.6181465 | 0.1524823 | 20 |
| AKR7L | -0.440758 | 0.184931 | 0.01715519 | 0.1161264 | 20 |
| NBL1 | -0.101423 | 0.28577 | 0.7226552 | 0.218604 | 20 |
| PLA2G2A | -0.0546806 | 0.0439698 | 0.2136487 | 0.7875404 | 20 |
| CDA | -0.113935 | 0.0723724 | 0.1154222 | 0.6504775 | 20 |
| EIF4G3 | -0.400311 | 0.403701 | 0.3213911 | 0.9310598 | 20 |
| ECE1 | -0.678068 | 0.549895 | 0.2175435 | 0.7677421 | 5 |
| HSPG2 | -0.14317 | 0.172461 | 0.4064495 | 0.1929418 | 20 |
| CELA3A | -0.0582423 | 0.143292 | 0.6844059 | 0.4533597 | 20 |
| C1QA | 0.0447577 | 0.0772868 | 0.5625136 | 0.8142109 | 20 |
| ELOA | 0.474911 | 0.339838 | 0.1622752 | 0.3505705 | 20 |
| FUCA1 | 0.0621095 | 0.0760861 | 0.4143258 | 0.1968755 | 20 |
| MYOM3 | -0.408041 | 0.376952 | 0.2790424 | 0.9987389 | 20 |
| IL22RA1 | 0.042275 | 0.122221 | 0.7294255 | 0.9111749 | 20 |
| IFNLR1 | 0.0277222 | 0.0799012 | 0.7286243 | 0.9554824 | 14 |
| LDLRAP1 | -0.113702 | 0.462473 | 0.8057931 | 0.9497412 | 15 |
| PAFAH2 | -0.261214 | 0.641048 | 0.6836563 | 0.5162306 | 9 |
| EXTL1 | 0.0685977 | 0.0878612 | 0.4349488 | 0.2389518 | 20 |
| CEP85 | 0.0808045 | 0.352825 | 0.8188522 | 0.2363955 | 20 |
| CD164L2 | -0.0209396 | 0.0593432 | 0.7241965 | 0.2939488 | 20 |
| FGR | -0.228441 | 0.340955 | 0.5028558 | 0.2331506 | 20 |
| RPA2 | -0.295317 | 0.360279 | 0.412393 | 0.2513266 | 20 |
| ATP5IF1 | -0.176992 | 0.272003 | 0.5152415 | 0.1301926 | 20 |
| MECR | -0.489968 | 0.470704 | 0.29791 | 0.5844596 | 20 |
| FABP3 | -0.353019 | 0.408656 | 0.3876683 | 0.216146 | 20 |
| TINAGL1 | -0.0727005 | 0.340177 | 0.8307704 | 0.005784879 | 20 |
| AK2 | -0.72895 | 0.857019 | 0.3950112 | 0.8003742 | 4 |
| CSF3R | 0.0119947 | 0.0542118 | 0.8248926 | 0.4356371 | 15 |
| RSPO1 | 0.268984 | 0.184804 | 0.1455286 | 0.1239541 | 20 |
| PPIE | 0.570867 | 0.671063 | 0.3949415 | 0.05184927 | 15 |
| GUCA2A | -0.0483177 | 0.209788 | 0.8178456 | 0.8763663 | 20 |
| FOXJ3 | -0.15544 | 0.411191 | 0.7054123 | 0.7681109 | 20 |
| ERMAP | 0.67273 | 0.318786 | 0.03483338 | 0.09428693 | 20 |
| TIE1 | -0.0767027 | 0.157301 | 0.6258207 | 0.2173795 | 20 |
| PTPRF | -0.592698 | 0.413388 | 0.1516413 | 0.7014672 | 20 |
| UROD | -0.0148323 | 0.0687852 | 0.8292751 | 0.2039919 | 20 |
| PRDX1 | -0.0901783 | 0.542631 | 0.8680095 | 0.8583025 | 13 |
| TSPAN1 | -0.869789 | 0.5917 | 0.1415662 | 0.5162056 | 19 |
| SCP2 | 1.38694 | 0.713794 | 0.05200946 | 0.7927451 | 20 |
| PCSK9 | 0.0897484 | 0.0666277 | 0.177976 | 0.527382 | 19 |
| C8B | 0.0387656 | 0.0561458 | 0.4899142 | 0.6852212 | 20 |
| TACSTD2 | -0.0337254 | 0.0464284 | 0.4675955 | 0.4766712 | 20 |
| ANGPTL3 | -0.186384 | 0.111487 | 0.09456341 | 0.7055172 | 20 |
| ROR1 | -0.0723035 | 0.0790885 | 0.3606063 | 0.4045817 | 20 |
| DNAJC6 | 0.000809382 | 0.36899 | 0.9982498 | 0.229979 | 20 |
| LEPR | -0.0657379 | 0.159272 | 0.6797965 | 0.5907101 | 20 |
| INSL5 | 0.00156805 | 0.16371 | 0.9923578 | 0.0939905 | 20 |
| ACADM | -0.0288378 | 0.229215 | 0.8998814 | 0.6132969 | 20 |
| NEXN | 0.142478 | 0.275659 | 0.6052529 | 0.3535851 | 20 |
| GIPC2 | 0.150604 | 0.115668 | 0.1929029 | 0.3710479 | 20 |
| CTBS | -0.00751345 | 0.0817341 | 0.9267573 | 0.9415012 | 20 |
| DDAH1 | -0.181684 | 0.182893 | 0.3205204 | 0.5121592 | 20 |
| CCN1 | -0.418692 | 0.296907 | 0.1584874 | 0.9355546 | 20 |
| COL24A1 | -0.0626185 | 0.212364 | 0.7680976 | 0.6485515 | 20 |
| GBP1 | -0.0658391 | 0.0647982 | 0.3095992 | 0.6892234 | 20 |
| GBP2 | -0.0892976 | 0.26347 | 0.7346633 | 0.900365 | 20 |
| GBP4 | 0.0363254 | 0.0357067 | 0.3089982 | 0.246827 | 20 |
| TGFBR3 | 0.0807825 | 0.204734 | 0.6931574 | 0.6583982 | 20 |
| EVI5 | 0.0166796 | 0.229015 | 0.9419399 | 0.3431669 | 20 |
| GCLM | 1.06819 | 0.489572 | 0.0291174 | 0.9329575 | 16 |
| F3 | -0.146442 | 0.157699 | 0.3530905 | 0.2243906 | 20 |
| VCAM1 | -0.282281 | 0.327594 | 0.3888636 | 0.482087 | 20 |
| AMY2B | -0.111584 | 0.0781145 | 0.1531562 | 0.1261798 | 20 |
| AMY2A | -0.00282355 | 0.079579 | 0.9716961 | 0.565465 | 20 |
| AMY1A | 0.0234024 | 0.0924126 | 0.8000842 | 0.2318344 | 20 |
| AMY1B | 0.0234024 | 0.0924126 | 0.8000842 | 0.2318344 | 20 |
| AMY1C | 0.0234024 | 0.0924126 | 0.8000842 | 0.2294284 | 20 |
| VAV3 | -0.225694 | 0.524479 | 0.666963 | 0.3216432 | 20 |
| CELSR2 | 0.0904911 | 0.0754268 | 0.230248 | 0.372852 | 20 |
| PSRC1 | -0.0556754 | 0.173939 | 0.7489037 | 0.1753414 | 19 |
| SORT1 | 0.0833269 | 0.184568 | 0.6516517 | 0.1337522 | 20 |
| AMIGO1 | 0.104532 | 0.622667 | 0.8666795 | 0.8047107 | 20 |
| GSTM4 | 0.0974494 | 0.155404 | 0.5306113 | 0.9611915 | 20 |
| CSF1 | -0.0144946 | 0.153361 | 0.9247015 | 0.4706827 | 20 |
| PROK1 | -0.0838012 | 0.0846683 | 0.3222919 | 0.9806963 | 20 |
| RHOC | -0.68867 | 0.356347 | 0.05328777 | 0.576682 | 20 |
| SLC16A1 | -0.308087 | 0.465952 | 0.5084853 | 0.5847392 | 20 |
| BCL2L15 | -0.439985 | 0.176961 | 0.01290664 | 0.4614321 | 20 |
| CD58 | -0.0773088 | 0.145116 | 0.5942141 | 0.8641906 | 20 |
| IGSF3 | 0.241116 | 0.342884 | 0.481932 | 0.8812642 | 20 |
| CD2 | 0.332286 | 0.778728 | 0.6695951 | 0.3999933 | 6 |
| CD101 | -0.0140726 | 0.0367136 | 0.7014915 | 0.9787439 | 20 |
| TTF2 | 0.0386457 | 0.0700319 | 0.5810655 | 0.1606761 | 20 |
| VTCN1 | 0.123603 | 0.460487 | 0.788378 | 0.97984 | 20 |
| MAN1A2 | -0.0221835 | 0.15697 | 0.8876143 | 0.9197737 | 20 |
| REG4 | -0.00386976 | 0.150507 | 0.9794875 | 0.4880006 | 13 |
| NOTCH2 | -0.113178 | 0.233333 | 0.6276402 | 0.07478384 | 20 |
| ACP6 | -0.011766 | 0.0320334 | 0.7133928 | 0.8518033 | 20 |
| BOLA1 | 0.353285 | 0.320836 | 0.2708372 | 0.8887343 | 20 |
| SV2A | 0.227158 | 0.20877 | 0.2765606 | 0.7253614 | 20 |
| SF3B4 | 0.709549 | 0.645773 | 0.2718728 | 0.749056 | 20 |
| PLEKHO1 | -0.680659 | 0.527165 | 0.1966446 | 0.9585181 | 20 |
| CA14 | 0.134037 | 0.164225 | 0.4143968 | 0.09615024 | 20 |
| ECM1 | 0.0704987 | 0.0427469 | 0.09910461 | 0.3537495 | 20 |
| ADAMTSL4 | 0.13345 | 0.15817 | 0.3988296 | 0.169331 | 20 |
| ENSA | -0.356217 | 0.492373 | 0.469391 | 0.4545586 | 20 |
| CTSS | 0.0330213 | 0.0700175 | 0.6372022 | 0.5289925 | 20 |
| MINDY1 | -0.035699 | 0.160601 | 0.8240928 | 0.04457075 | 19 |
| MENT | 0.064926 | 1.1698 | 0.9557386 | 0.9805017 | 20 |
| SEMA6C | 0.156301 | 0.29524 | 0.5965265 | 0.1216496 | 20 |
| TNFAIP8L2 | 0.736303 | 0.510476 | 0.1491939 | 0.9813627 | 20 |
| TDRKH | -0.0936981 | 0.0910974 | 0.3036917 | 0.6384752 | 20 |
| S100A11 | 0.405531 | 0.201325 | 0.04397717 | 0.09237214 | 20 |
| CRNN | 0.0044061 | 0.0493176 | 0.9288106 | 0.7418176 | 20 |
| SPRR3 | 0.0371872 | 0.118267 | 0.7531908 | 0.9353518 | 20 |
| PGLYRP4 | -0.0950544 | 0.104234 | 0.3618048 | 0.2171529 | 20 |
| S100A12 | 0.121195 | 0.138685 | 0.3821824 | 0.3068386 | 12 |
| S100A4 | -0.0367273 | 0.196852 | 0.8519954 | 0.8306982 | 20 |
| S100A3 | -0.0356978 | 0.247181 | 0.8851692 | 0.9880127 | 20 |
| S100A16 | 0.727665 | 0.284167 | 0.0104463 | 0.04610392 | 20 |
| S100A14 | 0.445491 | 0.165796 | 0.007210014 | 0.144252 | 20 |
| S100A13 | -0.406405 | 0.202234 | 0.044476 | 0.8994762 | 20 |
| IL6R | 0.0279225 | 0.0281902 | 0.3219278 | 0.9912742 | 20 |
| PMVK | 0.251142 | 0.365474 | 0.4919764 | 0.6612173 | 6 |
| PBXIP1 | 0.491649 | 0.650489 | 0.4497603 | 0.712852 | 20 |
| ADAM15 | 0.100803 | 0.0402304 | 0.01222293 | 0.5227669 | 20 |
| EFNA1 | 0.0405627 | 0.0896342 | 0.6508828 | 0.4048242 | 20 |
| SCAMP3 | 0.0612553 | 0.376565 | 0.8707792 | 0.8670444 | 20 |
| PKLR | 0.00573332 | 0.141532 | 0.9676872 | 0.5026328 | 20 |
| BGLAP | 0.0514195 | 0.337913 | 0.8790547 | 0.9400445 | 20 |
| BCAN | -0.0257843 | 0.133507 | 0.8468567 | 0.5890276 | 18 |
| HDGF | -0.0308282 | 0.0261731 | 0.2388531 | 0.1470318 | 20 |
| PEAR1 | -0.132916 | 0.138152 | 0.3359978 | 0.4757821 | 20 |
| FCRL5 | 0.00884915 | 0.0736763 | 0.9043972 | 0.3597263 | 20 |
| FCRL3 | -0.0142976 | 0.0297633 | 0.6309588 | 0.4620534 | 20 |
| FCRL2 | -0.00423114 | 0.0528534 | 0.936194 | 0.5801358 | 20 |
| FCRL1 | 0.0462152 | 0.0803842 | 0.5653393 | 0.2826968 | 20 |
| CD5L | -0.0364659 | 0.142305 | 0.7977567 | 0.5368807 | 20 |
| CD1C | 0.147211 | 0.313506 | 0.638667 | 0.66385 | 20 |
| MNDA | 0.69677 | 0.723482 | 0.3355077 | 0.2121359 | 20 |
| FCER1A | 0.234888 | 0.202931 | 0.2470776 | 0.9824594 | 20 |
| APCS | -0.269822 | 0.151235 | 0.07440253 | 0.3202936 | 17 |
| FCRL6 | 0.0190205 | 0.0449266 | 0.6720268 | 0.4016391 | 20 |
| SLAMF8 | -0.0383853 | 0.0328315 | 0.2423383 | 0.09629209 | 17 |
| IGSF9 | 0.0252648 | 0.133531 | 0.8499313 | 0.2095264 | 20 |
| IGSF8 | -0.175138 | 0.207632 | 0.3989494 | 0.3461661 | 20 |
| SLAMF6 | -0.218638 | 0.167693 | 0.1923015 | 0.1372244 | 18 |
| CD84 | 0.136 | 0.226128 | 0.5475537 | 0.932669 | 20 |
| SLAMF1 | 0.539707 | 0.259145 | 0.03728382 | 0.4386027 | 20 |
| CD48 | 0.0115925 | 0.0549084 | 0.83279 | 0.5015932 | 20 |
| SLAMF7 | 0.0191232 | 0.0486236 | 0.6941049 | 0.1923324 | 20 |
| LY9 | -0.0183079 | 0.0527049 | 0.7283158 | 0.4557209 | 20 |
| CD244 | -0.0874039 | 0.0890876 | 0.3265434 | 0.582198 | 20 |
| F11R | 0.016905 | 0.471821 | 0.9714186 | 0.6877059 | 20 |
| NECTIN4 | 0.0148405 | 0.0941264 | 0.8747198 | 0.2733633 | 20 |
| NIT1 | 0.161251 | 0.223364 | 0.470342 | 0.01080905 | 20 |
| ADAMTS4 | 0.0604578 | 0.242397 | 0.8030387 | 0.8761684 | 20 |
| APOA2 | 0.0213785 | 0.43522 | 0.9608228 | 0.9810274 | 20 |
| FCGR2A | 0.0215994 | 0.0314642 | 0.4924135 | 0.7599722 | 20 |
| FCGR2B | 0.0674991 | 0.0631447 | 0.2850886 | 0.1429846 | 20 |
| FCGR3B | -0.0706255 | 0.0394041 | 0.07307844 | 0.218026 | 20 |
| FCRLB | -0.0237551 | 0.044925 | 0.5969626 | 0.5376731 | 20 |
| GPA33 | 0.11529 | 0.155302 | 0.4578693 | 0.958279 | 18 |
| CREG1 | -0.141473 | 0.18727 | 0.449979 | 0.07617321 | 20 |
| XCL1 | 0.0141863 | 0.0406443 | 0.7270627 | 0.2720642 | 20 |
| DPT | 0.0233631 | 0.0864795 | 0.787039 | 0.6013762 | 20 |
| SELP | 0.222666 | 0.109011 | 0.04109156 | 0.3425331 | 18 |
| SELL | -0.0810293 | 0.0802686 | 0.3127461 | 0.8946018 | 20 |
| SELE | -0.111038 | 0.319952 | 0.7285567 | 0.8150226 | 20 |
| MYOC | 0.125453 | 0.111515 | 0.2605941 | 0.08800865 | 20 |
| FASLG | -0.711336 | 0.430771 | 0.09867544 | 0.5470925 | 20 |
| PRDX6 | 0.404187 | 0.235829 | 0.08654637 | 0.9388929 | 20 |
| SERPINC1 | 0.051727 | 0.343734 | 0.8803816 | 0.06477301 | 4 |
| RABGAP1L | 0.671509 | 0.415644 | 0.1061835 | 0.2643708 | 16 |
| CACYBP | -0.484063 | 0.304347 | 0.1117234 | 0.8983551 | 20 |
| TNN | 0.0292512 | 0.0335828 | 0.3837436 | 0.4432542 | 20 |
| TNR | 0.0381795 | 0.07621 | 0.6163867 | 0.4941857 | 20 |
| ANGPTL1 | -0.0663035 | 0.100657 | 0.5100859 | 0.5809202 | 20 |
| TOR1AIP1 | -0.034984 | 0.0403628 | 0.3860856 | 0.4377612 | 20 |
| QSOX1 | -0.0115637 | 0.0492527 | 0.8143774 | 0.9469743 | 20 |
| NPL | 0.559233 | 0.262172 | 0.03291848 | 0.03885361 | 20 |
| NCF2 | -0.512904 | 0.316955 | 0.105615 | 0.4472837 | 9 |
| TPR | 0.490411 | 0.440988 | 0.2661067 | 0.9469675 | 5 |
| PLA2G4A | -0.669064 | 0.480502 | 0.1637926 | 0.6508965 | 16 |
| CFH | 0.0213837 | 0.0998716 | 0.8304595 | 0.9869688 | 20 |
| CFHR2 | 0.0255392 | 0.0302242 | 0.3981147 | 0.8225397 | 20 |
| CFHR4 | 0.0249425 | 0.039926 | 0.532156 | 0.9651233 | 20 |
| CFHR5 | 0.0354066 | 0.0806296 | 0.6605699 | 0.6126659 | 20 |
| F13B | -0.0324178 | 0.0603839 | 0.5913627 | 0.03713541 | 20 |
| PTPRC | 0.143002 | 0.0860603 | 0.09658358 | 0.9816598 | 20 |
| LMOD1 | 0.120425 | 0.170991 | 0.4812607 | 0.3473912 | 20 |
| LMOD1 | 0.114396 | 0.162424 | 0.4812445 | 0.3697804 | 20 |
| LMOD1 | 0.113861 | 0.161663 | 0.4812404 | 0.3526606 | 20 |
| LMOD1 | 0.122214 | 0.173535 | 0.481271 | 0.3615864 | 20 |
| CHI3L1 | 0.0273278 | 0.0477277 | 0.5669306 | 0.3886387 | 20 |
| CHIT1 | 0.026649 | 0.0337072 | 0.4291751 | 0.7392073 | 20 |
| PRELP | 0.209106 | 0.0849924 | 0.01388238 | 0.110478 | 6 |
| OPTC | 0.00163216 | 0.0862981 | 0.9849104 | 0.6719266 | 20 |
| REN | 0.00156011 | 0.108025 | 0.9884773 | 0.7371536 | 20 |
| NFASC | -0.116881 | 0.0995639 | 0.2404241 | 0.3821984 | 19 |
| CNTN2 | -0.0467859 | 0.0379835 | 0.2180449 | 0.9554132 | 20 |
| PM20D1 | 0.0309383 | 0.036641 | 0.3984658 | 0.1656044 | 20 |
| CTSE | -0.350799 | 0.460061 | 0.4457591 | 0.6273959 | 20 |
| MAPKAPK2 | 0.0967076 | 0.209129 | 0.6437723 | 0.6893289 | 20 |
| IL10 | 0.354818 | 0.281616 | 0.2076933 | 0.5770283 | 20 |
| IL19 | -0.107972 | 0.0903426 | 0.2320306 | 0.001755902 | 20 |
| PIGR | -0.81235 | 0.358412 | 0.02341911 | 0.2201271 | 20 |
| FCAMR | -0.173231 | 0.0780504 | 0.02645429 | 0.6179513 | 20 |
| SARG | -0.209457 | 0.402248 | 0.6025659 | 0.9958553 | 6 |
| YOD1 | 0.153922 | 0.271603 | 0.5709066 | 0.02969753 | 20 |
| PFKFB2 | 0.000506217 | 0.152208 | 0.9973464 | 0.08173165 | 20 |
| C4BPB | -0.140936 | 0.204788 | 0.491325 | 0.5305271 | 11 |
| CD55 | 0.0183627 | 0.0668248 | 0.7834785 | 0.8865087 | 20 |
| CR2 | -0.0556262 | 0.197985 | 0.7787398 | 0.4965466 | 20 |
| CR1 | 0.0232596 | 0.0671515 | 0.7290605 | 0.4513381 | 20 |
| CD46 | 0.295486 | 0.324783 | 0.3629298 | 0.4513698 | 20 |
| CD34 | -1.1611 | 0.510814 | 0.02302343 | 0.5077942 | 20 |
| PPP2R5A | 0.133913 | 0.410835 | 0.7444598 | 0.8049323 | 20 |
| CENPF | 0.00612723 | 0.65441 | 0.9925295 | 0.8431633 | 20 |
| TGFB2 | 0.173646 | 0.204858 | 0.3966368 | 0.8970411 | 20 |
| SUSD4 | -0.0136936 | 0.0766683 | 0.8582453 | 0.4576195 | 20 |
| ENAH | 0.00589933 | 0.28036 | 0.9832122 | 0.2092392 | 20 |
| LEFTY2 | -0.0177915 | 0.0410511 | 0.6647246 | 0.8509114 | 20 |
| PARP1 | 0.098086 | 0.201681 | 0.6267252 | 0.5776161 | 20 |
| WNT9A | -0.0471352 | 0.224715 | 0.8338586 | 0.5835222 | 20 |
| GALNT2 | -0.0924765 | 0.0980033 | 0.3453711 | 0.5163198 | 20 |
| AGT | -0.0263938 | 0.0517107 | 0.6097628 | 0.8419531 | 20 |
| EGLN1 | -0.351519 | 0.246536 | 0.1539166 | 0.9660745 | 20 |
| NID1 | 0.0131814 | 0.125147 | 0.916116 | 0.9929424 | 7 |
| LGALS8 | -0.0917732 | 0.111149 | 0.4089857 | 0.9741376 | 20 |
| MTR | 1.32814 | 0.671837 | 0.04805603 | 0.6596307 | 13 |
| CEP170 | -0.23431 | 0.436606 | 0.5915007 | 0.5070798 | 20 |
| SDCCAG8 | 0.0450832 | 0.141287 | 0.7496587 | 0.9462533 | 20 |
| AKT3 | -0.0887339 | 0.521749 | 0.8649551 | 0.8255172 | 20 |
| EFCAB2 | -0.0732313 | 0.0745641 | 0.3260377 | 0.4182314 | 20 |
| TRIM58 | -0.217861 | 0.150054 | 0.1465351 | 0.8682978 | 20 |
| LYPD8 | -0.0161047 | 0.069175 | 0.8159083 | 0.3475277 | 20 |
| ACP1 | 0.0358558 | 0.0783223 | 0.6470977 | 0.01709781 | 20 |
| RRM2 | -0.382245 | 0.584585 | 0.5131928 | 0.6087706 | 7 |
| HPCAL1 | 0.0667602 | 0.183271 | 0.7156567 | 0.1966818 | 17 |
| VSNL1 | 0.153667 | 0.0920615 | 0.09508119 | 0.9905298 | 20 |
| MATN3 | 0.0468613 | 0.0550263 | 0.3944269 | 0.5648012 | 20 |
| SDC1 | -0.374488 | 0.221149 | 0.09038499 | 0.5929512 | 20 |
| HS1BP3 | -0.0075082 | 0.157279 | 0.9619249 | 0.03392125 | 9 |
| FKBP1B | -0.0786195 | 0.153002 | 0.6073603 | 0.1923605 | 20 |
| TP53I3 | 0.0935552 | 0.0592553 | 0.1143703 | 0.8561302 | 20 |
| POMC | 0.605722 | 0.368414 | 0.1001482 | 0.1800046 | 20 |
| DTNB | 1.67356 | 1.72468 | 0.3318664 | 0.1552428 | 4 |
| KHK | -0.0120041 | 0.0706065 | 0.8649989 | 0.3286714 | 20 |
| CGREF1 | 0.000725879 | 0.0532889 | 0.9891319 | 0.2763851 | 20 |
| ATRAID | -0.0567645 | 0.117663 | 0.6294992 | 0.2996341 | 20 |
| RBKS | 0.0688984 | 0.0982623 | 0.483198 | 0.8026332 | 17 |
| PLB1 | 0.0124101 | 0.0312382 | 0.6911666 | 0.2425326 | 20 |
| EHD3 | -0.0932634 | 0.165876 | 0.5739461 | 0.9757412 | 20 |
| DPY30 | -0.6804 | 0.668172 | 0.3085349 | 0.4087319 | 20 |
| CRIM1 | -0.361009 | 0.219074 | 0.0993752 | 0.4978787 | 20 |
| VIT | -0.0176169 | 0.0669073 | 0.7923168 | 0.4405796 | 20 |
| EIF2AK2 | -0.231191 | 0.191234 | 0.2266847 | 0.1301348 | 20 |
| QPCT | -0.119899 | 0.0708325 | 0.09051012 | 0.6238861 | 20 |
| MCFD2 | 0.0242219 | 0.214298 | 0.9100075 | 0.5335513 | 20 |
| CHAC2 | -0.162839 | 0.207407 | 0.4323846 | 0.8441494 | 20 |
| EFEMP1 | 0.3702 | 0.151047 | 0.01424993 | 0.09273683 | 20 |
| COMMD1 | -0.000268172 | 0.113063 | 0.9981075 | 0.2335158 | 20 |
| EHBP1 | 0.247968 | 0.195285 | 0.2041647 | 0.1245128 | 20 |
| MDH1 | 0.297236 | 0.408488 | 0.4668277 | 0.05418586 | 20 |
| SPRED2 | 0.0344382 | 0.411401 | 0.9332873 | 0.9029864 | 20 |
| ARHGAP25 | 0.0448116 | 0.311576 | 0.8856406 | 0.7488434 | 20 |
| BMP10 | 0.141498 | 0.082329 | 0.08566996 | 0.5394939 | 4 |
| NFU1 | 0.0817899 | 0.318069 | 0.7970666 | 0.7314565 | 20 |
| ANXA4 | 0.0495854 | 0.564765 | 0.930037 | 0.8095676 | 16 |
| TGFA | -0.229619 | 0.264361 | 0.3850757 | 0.3030848 | 20 |
| CD207 | -0.00582179 | 0.0695543 | 0.9332939 | 0.4182694 | 20 |
| NAGK | 0.0218203 | 0.133978 | 0.8706244 | 0.3624431 | 20 |
| MCEE | 0.0827789 | 0.151991 | 0.5860088 | 0.2091201 | 20 |
| STAMBP | -0.370293 | 0.528073 | 0.4831685 | 0.6108216 | 5 |
| MTHFD2 | -0.305455 | 0.332184 | 0.3578165 | 0.9857421 | 5 |
| REG3G | -0.0428733 | 0.107464 | 0.6899263 | 0.5788472 | 20 |
| REG1B | 0.0700648 | 0.0845103 | 0.4070661 | 0.5891031 | 20 |
| REG1A | 0.0961794 | 0.116026 | 0.4071344 | 0.5181051 | 20 |
| REG3A | 0.00885694 | 0.195252 | 0.9638191 | 0.7292181 | 20 |
| TMSB10 | -0.452395 | 0.499112 | 0.3647246 | 0.5459408 | 7 |
| TGOLN2 | -0.0161068 | 0.0399565 | 0.6868689 | 0.03109075 | 20 |
| CAPG | -0.0482222 | 0.0335408 | 0.1505142 | 0.6901353 | 20 |
| VAMP8 | 0.0252035 | 0.181758 | 0.8897147 | 0.4191145 | 20 |
| VAMP5 | -0.273718 | 0.360947 | 0.448251 | 0.03811913 | 20 |
| GNLY | 0.0174069 | 0.0452675 | 0.700583 | 0.4392156 | 20 |
| IMMT | 0.324403 | 0.249434 | 0.1934098 | 0.02544345 | 20 |
| CD8A | -0.140924 | 0.132569 | 0.2877696 | 0.7571378 | 20 |
| FABP1 | -0.259357 | 0.120593 | 0.03150235 | 0.8509927 | 20 |
| EIF2AK3 | -0.00520331 | 0.24054 | 0.9827416 | 0.6898771 | 20 |
| MITD1 | 0.314772 | 0.38598 | 0.4147789 | 0.1704331 | 20 |
| TXNDC9 | 1.96253 | 0.675776 | 0.003682934 | 0.1886682 | 13 |
| RNF149 | -0.0907455 | 0.101671 | 0.3721019 | 0.6563152 | 20 |
| IL1R2 | -0.0846482 | 0.0553261 | 0.1260202 | 0.3276352 | 20 |
| IL1R1 | -0.199603 | 0.174091 | 0.2515691 | 0.5485734 | 20 |
| IL1RL2 | -0.0118206 | 0.065621 | 0.8570471 | 0.2928967 | 20 |
| IL1RL1 | 0.000460108 | 0.0439377 | 0.9916448 | 0.9599692 | 20 |
| IL18R1 | 0.0181564 | 0.0404672 | 0.6536702 | 0.8707561 | 20 |
| UXS1 | -0.173058 | 0.212953 | 0.4164143 | 0.5842115 | 20 |
| EDAR | -0.0962023 | 0.0979513 | 0.3260288 | 0.984271 | 20 |
| MERTK | 0.087701 | 0.113478 | 0.4396137 | 0.2590275 | 20 |
| IL36G | 0.204002 | 0.49966 | 0.6830668 | 0.7995633 | 20 |
| IL36A | 0.194332 | 0.559298 | 0.7282475 | 0.3929833 | 20 |
| IL1RN | 0.0546249 | 0.143257 | 0.7029747 | 0.5704311 | 20 |
| DPP10 | -0.0146977 | 0.293504 | 0.9600613 | 0.1904313 | 20 |
| MARCO | 0.0216899 | 0.112021 | 0.8464707 | 0.02223112 | 20 |
| C1QL2 | 0.161173 | 0.11016 | 0.1434479 | 0.2089398 | 20 |
| DBI | -0.0369962 | 0.116868 | 0.7515743 | 0.5219035 | 20 |
| RALB | -0.229935 | 0.251003 | 0.359632 | 0.872744 | 20 |
| INHBB | -0.0956393 | 0.0786239 | 0.2238271 | 0.8007425 | 20 |
| PROC | -0.0770026 | 0.149208 | 0.6058011 | 0.7225305 | 20 |
| HS6ST1 | 0.0973057 | 0.139343 | 0.4849795 | 0.0707053 | 20 |
| DARS1 | -0.0783217 | 0.18138 | 0.66588 | 0.9372088 | 9 |
| HNMT | -0.00290778 | 0.0351799 | 0.9341263 | 0.882632 | 20 |
| KYNU | -0.164033 | 0.121758 | 0.1779141 | 0.8457849 | 20 |
| NMI | 0.0794007 | 0.0629672 | 0.2073141 | 0.03207947 | 20 |
| GALNT5 | -0.120438 | 0.167361 | 0.4717521 | 0.2271813 | 20 |
| CD302 | 0.0873513 | 0.118112 | 0.4595659 | 0.4728666 | 20 |
| LY75 | -0.0242603 | 0.0314486 | 0.4404535 | 0.0209284 | 20 |
| ITGB6 | -0.175521 | 0.106303 | 0.09871064 | 0.9791575 | 20 |
| TANK | -1.37085 | 0.673645 | 0.04185325 | 0.9667258 | 15 |
| DPP4 | -0.0669389 | 0.125199 | 0.592884 | 0.426454 | 20 |
| FAP | -0.0093548 | 0.109184 | 0.9317217 | 0.7021692 | 20 |
| GALNT3 | -0.0192602 | 0.0819593 | 0.8142114 | 0.605329 | 20 |
| GORASP2 | -0.859468 | 0.584427 | 0.141395 | 0.3721507 | 20 |
| METAP1D | 0.620249 | 0.578978 | 0.2840423 | 0.2547527 | 20 |
| ITGA6 | -0.13575 | 0.138749 | 0.3278825 | 0.6816404 | 20 |
| FKBP7 | 0.227328 | 0.861546 | 0.7918872 | 0.5787136 | 6 |
| SESTD1 | 0.27978 | 0.295998 | 0.3445523 | 0.4116041 | 20 |
| FRZB | 0.1476 | 0.0992905 | 0.1371337 | 0.3532106 | 20 |
| ITGAV | 0.0161657 | 0.243243 | 0.9470123 | 0.09093134 | 20 |
| FAM171B | -0.206755 | 0.0894057 | 0.02074761 | 0.8163128 | 20 |
| TFPI | 0.0373922 | 0.0888774 | 0.6739617 | 0.5755493 | 20 |
| PMS1 | -0.425867 | 0.651932 | 0.5136031 | 0.7332003 | 14 |
| MSTN | 0.389366 | 0.882826 | 0.6591804 | 0.04961444 | 7 |
| INPP1 | 0.186262 | 0.284509 | 0.5126738 | 0.265122 | 16 |
| C2orf69 | 0.00978293 | 0.423811 | 0.9815839 | 0.988672 | 20 |
| CASP10 | 0.0255882 | 0.156426 | 0.8700615 | 0.2182836 | 20 |
| CASP8 | -0.168785 | 0.149233 | 0.2580497 | 0.3707761 | 20 |
| CD28 | -0.197489 | 0.166789 | 0.236388 | 0.1271214 | 20 |
| NRP2 | -0.401545 | 0.198448 | 0.04302893 | 0.6014401 | 20 |
| ADAM23 | -0.0570091 | 0.0686378 | 0.4062118 | 0.4519684 | 20 |
| CRYGD | 0.0124219 | 0.0428966 | 0.7721387 | 0.2194348 | 20 |
| MAP2 | -1.04647 | 0.909764 | 0.2500342 | 0.9085741 | 20 |
| RPE | 0.135037 | 0.502681 | 0.7882114 | 0.5141045 | 20 |
| ERBB4 | 0.249639 | 0.223688 | 0.2644167 | 0.7062912 | 20 |
| VWC2L | -0.525182 | 0.329144 | 0.1105785 | 0.2215181 | 20 |
| FN1 | -0.063569 | 0.0781276 | 0.4158421 | 0.004482161 | 20 |
| PECR | 0.00338617 | 0.123216 | 0.9780756 | 0.5430541 | 20 |
| IGFBP2 | 1.07917 | 0.461142 | 0.01927263 | 0.3203162 | 7 |
| DNPEP | 0.317276 | 0.673407 | 0.6375336 | 0.08193329 | 9 |
| EPHA4 | 0.129193 | 0.204006 | 0.526553 | 0.5184256 | 20 |
| SCG2 | -0.00936406 | 0.610426 | 0.9877608 | 0.250401 | 8 |
| SERPINE2 | -0.0382558 | 0.0566048 | 0.4991412 | 0.3491443 | 20 |
| CCL20 | -0.0666625 | 0.343859 | 0.8462809 | 0.4082129 | 20 |
| DNER | 0.0538999 | 0.113725 | 0.6355374 | 0.6854682 | 20 |
| B3GNT7 | 0.0199859 | 0.0409168 | 0.6252295 | 0.2345286 | 20 |
| NPPC | -0.721866 | 0.685205 | 0.2921099 | 0.8133781 | 10 |
| ALPP | -0.00409997 | 0.0625697 | 0.9477548 | 0.8016694 | 20 |
| EFHD1 | -0.0995328 | 0.161988 | 0.5389221 | 0.4197428 | 20 |
| INPP5D | -0.0439698 | 0.192272 | 0.8191135 | 0.4259303 | 20 |
| COL6A3 | 0.212121 | 0.316281 | 0.5024278 | 0.5086897 | 18 |
| LRRFIP1 | -0.376202 | 0.570577 | 0.5096799 | 0.9495864 | 20 |
| SCLY | 0.198808 | 0.178175 | 0.2645068 | 0.6600474 | 20 |
| GPC1 | -0.169293 | 0.0858243 | 0.04854587 | 0.983134 | 20 |
| AGXT | -0.0840151 | 0.106939 | 0.4320807 | 0.03211483 | 20 |
| SNED1 | -0.0611126 | 0.2297 | 0.7901974 | 0.8582813 | 20 |
| DTYMK | -1.14393 | 0.565937 | 0.04324896 | 0.9830271 | 20 |
| PDCD1 | 0.0883084 | 0.156165 | 0.5717465 | 0.203716 | 20 |
| CHL1 | -0.0979393 | 0.112318 | 0.3832199 | 0.5803215 | 20 |
| CNTN4 | -0.126 | 0.115081 | 0.27357 | 0.9578867 | 20 |
| IL5RA | 0.131504 | 0.106109 | 0.2152218 | 0.104244 | 20 |
| LRRN1 | -0.0179602 | 0.0361426 | 0.6192401 | 0.5988204 | 20 |
| SETMAR | 0.12373 | 0.131816 | 0.3479074 | 0.5766351 | 20 |
| ITPR1 | 0.0814985 | 0.412706 | 0.843457 | 0.8815712 | 20 |
| CRELD1 | -0.0315053 | 0.0459127 | 0.4925864 | 0.7748573 | 20 |
| PRRT3 | 0.126192 | 0.179718 | 0.482577 | 0.8046036 | 20 |
| GHRL | -0.0239958 | 0.0661537 | 0.716808 | 0.2040019 | 10 |
| TIMP4 | -0.00446106 | 0.0875012 | 0.9593392 | 0.03773917 | 20 |
| FBLN2 | -0.17217 | 0.109126 | 0.114632 | 0.8837545 | 20 |
| BTD | -0.066597 | 0.0402779 | 0.09824144 | 0.04362018 | 20 |
| TOP2B | 0.432198 | 0.361386 | 0.2317172 | 0.07704949 | 20 |
| CMC1 | -0.227922 | 0.475316 | 0.6315713 | 0.443694 | 7 |
| TGFBR2 | -0.417675 | 0.601513 | 0.4874479 | 0.4521576 | 9 |
| GLB1 | -0.0618064 | 0.127913 | 0.628959 | 0.2666038 | 20 |
| SUSD5 | -0.162506 | 0.0849277 | 0.05568896 | 0.500284 | 20 |
| ACAA1 | 0.0830857 | 0.29347 | 0.7770885 | 0.2868889 | 20 |
| RPL14 | -0.00859943 | 0.52647 | 0.9869678 | 0.1201842 | 10 |
| GASK1A | -0.0571189 | 0.0398974 | 0.1522454 | 0.3322368 | 20 |
| CLEC3B | 0.158569 | 0.13047 | 0.2242279 | 0.8337725 | 20 |
| CDCP1 | 0.144825 | 0.119067 | 0.223861 | 0.5512831 | 20 |
| LZTFL1 | -0.0715458 | 0.159001 | 0.6527304 | 0.9875225 | 20 |
| TDGF1 | 0.0471298 | 0.0233469 | 0.04352064 | 0.753254 | 20 |
| PTH1R | -0.0975366 | 0.189806 | 0.6073391 | 0.287708 | 5 |
| SPINK8 | -0.0228555 | 0.0762028 | 0.7642303 | 0.1533415 | 20 |
| SHISA5 | 0.305949 | 0.518924 | 0.5554702 | 0.5215758 | 16 |
| DAG1 | 0.139997 | 0.483744 | 0.7722736 | 0.7617721 | 20 |
| MST1 | -0.018342 | 0.0275989 | 0.506312 | 0.5510141 | 20 |
| SEMA3F | -0.0337194 | 0.238991 | 0.8877982 | 0.7485591 | 20 |
| NAA80 | 0.0525244 | 0.216339 | 0.80817 | 0.4800938 | 17 |
| HYAL1 | -0.0624055 | 0.107091 | 0.5600722 | 0.3787839 | 20 |
| MANF | 0.376434 | 0.431166 | 0.382629 | 0.8197943 | 20 |
| ABHD14B | 0.0310534 | 0.111473 | 0.7805715 | 0.5957224 | 16 |
| ACY1 | 0.111418 | 0.359118 | 0.7563667 | 0.8651421 | 13 |
| TWF2 | 0.181242 | 0.503517 | 0.7188836 | 0.8422394 | 20 |
| ITIH1 | -0.107698 | 0.20964 | 0.607441 | 0.9361358 | 20 |
| ITIH3 | 0.0402354 | 0.0938923 | 0.6682675 | 0.9836749 | 20 |
| ITIH4 | 0.0771097 | 0.11818 | 0.514094 | 0.8532512 | 20 |
| IL17RB | -0.00754469 | 0.0368275 | 0.837677 | 0.6438762 | 20 |
| SLMAP | -0.460396 | 0.610384 | 0.450686 | 0.7018057 | 20 |
| FAM3D | 0.139551 | 0.0871 | 0.1091131 | 0.95756 | 20 |
| FHIT | 0.110863 | 0.338677 | 0.7434106 | 0.9065459 | 20 |
| LRIG1 | 0.027802 | 0.0382134 | 0.4668916 | 0.1947809 | 20 |
| CNTN3 | 0.0355214 | 0.133567 | 0.7902827 | 0.7555082 | 20 |
| ROBO2 | -0.377436 | 0.578561 | 0.5141627 | 0.09457961 | 20 |
| ROBO1 | 0.0211664 | 0.140139 | 0.8799459 | 0.7969555 | 20 |
| PROS1 | -0.576142 | 0.493018 | 0.2425634 | 0.05704098 | 16 |
| CPOX | -0.164917 | 0.217881 | 0.4491018 | 0.6572073 | 20 |
| DCBLD2 | -0.0741571 | 0.340827 | 0.8277566 | 0.5021787 | 12 |
| TBC1D23 | 0.0331988 | 0.185254 | 0.8577752 | 0.8895392 | 20 |
| NIT2 | 0.174162 | 0.381307 | 0.6478503 | 0.5340989 | 20 |
| ALCAM | -0.228562 | 0.25545 | 0.3709249 | 0.7344006 | 20 |
| CD200 | 0.0237035 | 0.149292 | 0.8738478 | 0.2338215 | 20 |
| CCDC80 | -0.357258 | 0.418651 | 0.3934629 | 0.4855751 | 20 |
| CD200R1 | 0.00770194 | 0.0536687 | 0.8858881 | 0.4594307 | 20 |
| BOC | 0.0871801 | 0.153176 | 0.5692554 | 0.5934753 | 20 |
| TIGIT | -0.358714 | 0.351183 | 0.3070443 | 0.4818312 | 20 |
| CD80 | 0.0882181 | 0.0749463 | 0.2391618 | 0.6833781 | 20 |
| FSTL1 | 0.132684 | 0.193016 | 0.4918156 | 0.778011 | 20 |
| HCLS1 | -0.161434 | 0.368272 | 0.6611278 | 0.05792885 | 20 |
| CD86 | 0.563973 | 0.236458 | 0.01707509 | 0.112347 | 20 |
| PDIA5 | -0.0879799 | 0.117621 | 0.4544638 | 0.1703262 | 20 |
| ITGB5 | -0.0248669 | 0.406313 | 0.9511987 | 0.1650516 | 20 |
| MUC13 | 0.156689 | 0.147958 | 0.289594 | 0.540876 | 20 |
| HEG1 | -0.223095 | 0.270086 | 0.4087967 | 0.6559645 | 20 |
| CHCHD6 | -0.143914 | 0.122189 | 0.2388748 | 0.5563568 | 20 |
| PODXL2 | 0.0155912 | 0.0579721 | 0.7879736 | 0.5299125 | 20 |
| MGLL | 0.44438 | 0.480026 | 0.3545803 | 0.441349 | 20 |
| NUDT16 | 0.0974197 | 0.114843 | 0.3962769 | 0.3907166 | 20 |
| TF | -0.137814 | 0.0939064 | 0.1422219 | 0.08581973 | 20 |
| AMOTL2 | -0.106373 | 0.477129 | 0.8235799 | 0.1793294 | 20 |
| IL20RB | -0.10222 | 0.254002 | 0.6873622 | 0.1226327 | 20 |
| RBP2 | 0.336423 | 0.475965 | 0.4796769 | 0.7861701 | 20 |
| RBP1 | 0.00338721 | 0.26426 | 0.9897732 | 0.2931688 | 20 |
| CLSTN2 | -0.0208688 | 0.0945265 | 0.8252699 | 0.7027211 | 20 |
| CPB1 | 0.192344 | 0.265487 | 0.4687613 | 0.440733 | 20 |
| MME | 0.234477 | 0.536415 | 0.6620257 | 0.2627682 | 10 |
| PTX3 | -1.1745 | 0.472829 | 0.01299229 | 0.1248341 | 17 |
| LXN | -0.124734 | 0.169802 | 0.4625932 | 0.7894317 | 20 |
| RARRES1 | 0.0518732 | 0.0622509 | 0.4046795 | 0.4584654 | 20 |
| BCHE | 0.0665658 | 0.0639548 | 0.2979564 | 0.229912 | 20 |
| SERPINI2 | -0.164482 | 0.0966352 | 0.08873834 | 0.07090272 | 20 |
| SERPINI1 | 0.0795758 | 0.0743346 | 0.2843909 | 0.8496792 | 20 |
| TNFSF10 | -0.0125203 | 0.135018 | 0.9261175 | 0.8008999 | 20 |
| LAMP3 | 0.198606 | 0.130281 | 0.127396 | 0.5639011 | 20 |
| THPO | -0.0451715 | 0.472264 | 0.9237995 | 0.8216662 | 17 |
| AHSG | 0.00316829 | 0.0432172 | 0.9415588 | 0.9441933 | 20 |
| FETUB | 0.0885304 | 0.088943 | 0.3195604 | 0.9020289 | 18 |
| HRG | -0.0102321 | 0.0634308 | 0.8718481 | 0.8831188 | 20 |
| ADIPOQ | 0.192545 | 0.150289 | 0.2001369 | 0.9430728 | 17 |
| ST6GAL1 | -0.0735192 | 0.172832 | 0.67056 | 0.06958291 | 20 |
| MASP1 | 0.0380113 | 0.14265 | 0.7898812 | 0.4779073 | 20 |
| IL1RAP | -0.034033 | 0.0293224 | 0.2457843 | 0.3585475 | 20 |
| OSTN | 0.426091 | 0.353599 | 0.2281989 | 0.6133503 | 20 |
| CCDC50 | -0.103068 | 0.165944 | 0.5345328 | 0.1980932 | 17 |
| GP5 | 0.0341786 | 0.247675 | 0.8902425 | 0.6169402 | 20 |
| APOD | 0.0446966 | 0.133021 | 0.7368617 | 0.5049474 | 20 |
| TFRC | -0.0221972 | 0.0543301 | 0.6828617 | 0.8219626 | 20 |
| MELTF | -0.0992226 | 0.0704493 | 0.1590048 | 0.04494595 | 20 |
| IDUA | 0.0643954 | 0.0590104 | 0.2751603 | 0.1067627 | 20 |
| SPON2 | 0.0923416 | 0.143363 | 0.5195039 | 0.9463302 | 20 |
| TACC3 | 0.0660123 | 0.146064 | 0.6513117 | 0.997258 | 20 |
| ADD1 | -0.192993 | 0.208801 | 0.3553354 | 0.7687015 | 20 |
| HGFAC | -0.0263667 | 0.0689674 | 0.7022345 | 0.4047187 | 20 |
| LRPAP1 | 0.0302656 | 0.171473 | 0.8598981 | 0.3245447 | 20 |
| LYAR | -0.943315 | 0.562466 | 0.09352195 | 0.9764645 | 20 |
| CYTL1 | -0.0780006 | 0.0437365 | 0.0745179 | 0.1598621 | 13 |
| MAN2B2 | -0.0408524 | 0.0350551 | 0.2438661 | 0.339256 | 20 |
| S100P | -0.190928 | 0.171591 | 0.2658407 | 0.5324141 | 20 |
| GRPEL1 | 0.0274158 | 0.53603 | 0.9592092 | 0.3697709 | 20 |
| SORCS2 | -0.0156859 | 0.0643737 | 0.8074867 | 0.9634845 | 20 |
| PSAPL1 | -0.0866567 | 0.0588467 | 0.1408633 | 0.01311312 | 20 |
| AFAP1 | -0.0385972 | 0.0298632 | 0.1961953 | 0.4142756 | 20 |
| BST1 | -0.00345489 | 0.0286818 | 0.904122 | 0.8758324 | 20 |
| CD38 | -0.147404 | 0.0824895 | 0.07394676 | 0.3915475 | 20 |
| FGFBP1 | -0.282023 | 0.26675 | 0.2903951 | 0.1767476 | 20 |
| FGFBP2 | -0.129674 | 0.0530124 | 0.01444058 | 0.7902803 | 20 |
| QDPR | 0.165341 | 0.0777865 | 0.03353898 | 0.4428491 | 20 |
| LAP3 | -0.799617 | 0.960029 | 0.4048959 | 0.4101717 | 16 |
| SLIT2 | 0.417667 | 0.504835 | 0.4080482 | 0.9823219 | 20 |
| SOD3 | -0.0451818 | 0.0495134 | 0.3614973 | 0.9434036 | 7 |
| PCDH7 | 0.249443 | 0.275253 | 0.3648137 | 0.882878 | 20 |
| TLR1 | 0.0366024 | 0.078659 | 0.641695 | 0.9208566 | 20 |
| KLB | 0.010107 | 0.0386919 | 0.7939249 | 0.227343 | 20 |
| GNPDA2 | 0.0196744 | 0.126572 | 0.8764736 | 0.8248404 | 20 |
| PDGFRA | 0.0795054 | 0.103794 | 0.4436811 | 0.4072985 | 20 |
| KIT | -0.849012 | 0.51533 | 0.09945266 | 0.02959725 | 20 |
| KDR | 0.00996415 | 0.0591028 | 0.8661189 | 0.1971254 | 18 |
| SPINK2 | 0.0654157 | 0.0808102 | 0.418229 | 0.5848859 | 14 |
| IGFBP7 | 0.028057 | 0.0828558 | 0.7348924 | 0.1682361 | 20 |
| TMPRSS11D | -0.381661 | 0.232065 | 0.1000462 | 0.569526 | 8 |
| ODAM | 0.213555 | 0.110391 | 0.05304817 | 0.4609359 | 20 |
| AMBN | -0.0332144 | 0.390957 | 0.9322959 | 0.5954313 | 20 |
| GC | -0.00811454 | 0.029208 | 0.7811509 | 0.7710063 | 20 |
| AFP | -0.292627 | 0.156351 | 0.0612622 | 0.2115721 | 20 |
| AFM | -0.145813 | 0.372226 | 0.6952567 | 0.3233196 | 20 |
| CXCL8 | -0.524336 | 0.419951 | 0.211824 | 0.1309855 | 20 |
| CXCL8 | -0.527693 | 0.417081 | 0.2057976 | 0.1582396 | 20 |
| CXCL8 | -0.504049 | 0.403407 | 0.21149 | 0.1159796 | 20 |
| CXCL8 | -0.504432 | 0.421585 | 0.2314969 | 0.1189171 | 20 |
| CXCL6 | 0.00867162 | 0.0522814 | 0.8682637 | 0.2250462 | 20 |
| CXCL1 | 0.0165677 | 0.0481275 | 0.7306618 | 0.369234 | 20 |
| PF4 | -0.332057 | 0.384716 | 0.3880709 | 0.1928321 | 20 |
| PPBP | -0.0319575 | 0.155325 | 0.8369898 | 0.6806729 | 4 |
| CXCL5 | -0.102359 | 0.0774536 | 0.1863183 | 0.8066175 | 20 |
| EPGN | -0.879324 | 0.610182 | 0.1495603 | 0.2904728 | 8 |
| EREG | 0.371829 | 0.97326 | 0.7024281 | 0.944572 | 11 |
| AREG | 0.260712 | 0.201233 | 0.1951235 | 0.9364632 | 20 |
| BTC | 0.0196594 | 0.0695271 | 0.7773611 | 0.8671169 | 19 |
| NAAA | -0.0132747 | 0.0469338 | 0.7773004 | 0.2922932 | 20 |
| CXCL9 | -0.0315635 | 0.211508 | 0.8813713 | 0.3800129 | 20 |
| ART3 | 0.0101698 | 0.120324 | 0.9326432 | 0.04075608 | 20 |
| CXCL10 | 0.100628 | 0.0784287 | 0.199475 | 0.189373 | 4 |
| CXCL11 | -0.212804 | 0.127924 | 0.09620879 | 0.8074583 | 20 |
| SCARB2 | -0.14789 | 0.132567 | 0.2645973 | 0.05768246 | 15 |
| CXCL13 | -0.0720711 | 0.525493 | 0.8909127 | 0.9999997 | 20 |
| ANXA3 | -0.128082 | 0.325721 | 0.6941518 | 0.712266 | 20 |
| FGF5 | 0.049884 | 0.0450419 | 0.2680765 | 0.06457125 | 20 |
| SEC31A | -0.0449473 | 0.275237 | 0.8702789 | 0.7976097 | 7 |
| HPSE | 0.0803606 | 0.10725 | 0.4536868 | 0.9102923 | 9 |
| SPARCL1 | -0.0336365 | 0.0409518 | 0.4114359 | 0.01951833 | 20 |
| DMP1 | 0.26325 | 0.574514 | 0.646799 | 0.6607079 | 20 |
| MEPE | -0.0847111 | 0.196389 | 0.666218 | 0.1663682 | 20 |
| SPP1 | 0.60055 | 0.323028 | 0.06300946 | 0.4911566 | 20 |
| PKD2 | 0.857859 | 0.412023 | 0.03733643 | 0.02598999 | 20 |
| FAM13A | -0.0385078 | 0.257796 | 0.8812591 | 0.7450232 | 20 |
| SNCA | -0.341429 | 0.648134 | 0.5983406 | 0.5486344 | 20 |
| HPGDS | -0.0378615 | 0.0640794 | 0.5546193 | 0.255784 | 20 |
| ADH4 | 0.345947 | 0.223678 | 0.1219533 | 0.6500099 | 20 |
| ADH1B | -0.606153 | 0.83186 | 0.4662023 | 0.06524455 | 11 |
| DAPP1 | -0.0688456 | 0.285521 | 0.8094602 | 0.3684201 | 20 |
| DNAJB14 | -0.629288 | 0.58944 | 0.2857001 | 0.1843127 | 20 |
| BANK1 | 0.307836 | 0.268713 | 0.251963 | 0.04169297 | 7 |
| NFKB1 | -0.0326421 | 0.250608 | 0.8963674 | 0.353916 | 20 |
| HADH | 0.632368 | 0.693277 | 0.3616932 | 0.3616788 | 20 |
| CFI | 0.0163872 | 0.132184 | 0.9013368 | 0.7180638 | 20 |
| EGF | -0.0846434 | 0.220939 | 0.7016404 | 0.2702709 | 20 |
| ENPEP | 0.51367 | 0.435063 | 0.2377296 | 0.4407553 | 9 |
| FABP2 | -0.045536 | 0.138783 | 0.7428294 | 0.876877 | 17 |
| PDE5A | -0.0264198 | 0.188406 | 0.8884799 | 0.9962783 | 20 |
| ANXA5 | -0.0555021 | 0.27306 | 0.8389319 | 0.492562 | 20 |
| FGF2 | 0.00639782 | 0.0469844 | 0.8916877 | 0.7197801 | 20 |
| CLGN | 0.0797348 | 0.0433588 | 0.06592216 | 0.3470565 | 20 |
| ARFIP1 | 0.00164205 | 0.179502 | 0.9927012 | 0.07922788 | 20 |
| FGA | 0.377702 | 0.368376 | 0.3052138 | 0.2682333 | 4 |
| CTSO | 0.0316836 | 0.171985 | 0.8538389 | 0.1476962 | 20 |
| PDGFC | -0.735445 | 0.486176 | 0.1303525 | 0.2437642 | 5 |
| RAPGEF2 | 0.674324 | 0.624147 | 0.279967 | 0.669237 | 20 |
| CPE | 0.040174 | 0.124822 | 0.7475652 | 0.413946 | 20 |
| ANXA10 | 0.554681 | 0.569542 | 0.3301026 | 0.6518339 | 20 |
| GALNT7 | -0.311304 | 0.450994 | 0.4900293 | 0.5412358 | 20 |
| SCRG1 | -0.489359 | 0.265192 | 0.06499377 | 0.297761 | 20 |
| VEGFC | 1.39258 | 1.24965 | 0.2651212 | 0.604972 | 19 |
| DCTD | -0.402342 | 0.55503 | 0.4685135 | 0.9136241 | 20 |
| ENPP6 | 0.0446082 | 0.0976137 | 0.6476807 | 0.5027551 | 20 |
| CASP3 | 0.254124 | 0.249525 | 0.3084742 | 0.8513001 | 20 |
| TLR3 | 0.0473685 | 0.0266342 | 0.07532375 | 0.3705454 | 20 |
| KLKB1 | 0.0467528 | 0.0768979 | 0.543197 | 0.3647533 | 20 |
| F11 | 0.0491973 | 0.0748069 | 0.5107584 | 0.7910513 | 20 |
| PDCD6 | -0.00977473 | 0.0329388 | 0.766654 | 0.9994973 | 20 |
| ADAMTS16 | -0.0889924 | 0.247289 | 0.7189426 | 0.281592 | 17 |
| CDH6 | -0.0182264 | 0.0559089 | 0.7444231 | 0.4619453 | 20 |
| PDZD2 | 0.0701324 | 0.125106 | 0.5750806 | 0.7160713 | 20 |
| DNAJC21 | -0.512212 | 0.271318 | 0.05904399 | 0.8540701 | 20 |
| IL7R | 0.0664282 | 0.0341093 | 0.05147353 | 0.9514273 | 20 |
| GDNF | 0.153879 | 0.136924 | 0.2610841 | 0.04979366 | 20 |
| LIFR | -0.141317 | 0.145318 | 0.3308178 | 0.5980211 | 17 |
| OSMR | 0.0161007 | 0.0601747 | 0.7890329 | 0.03625945 | 20 |
| C9 | 0.205895 | 0.324598 | 0.5258801 | 0.726578 | 20 |
| C7 | -0.0350198 | 0.0347608 | 0.3137177 | 0.894657 | 20 |
| OXCT1 | 0.37425 | 0.667445 | 0.5749883 | 0.9840709 | 20 |
| GHR | 0.0285553 | 0.0473093 | 0.5461181 | 0.3797118 | 20 |
| SELENOP | 0.0943223 | 0.304069 | 0.7564084 | 0.7869598 | 20 |
| CCL28 | -0.0643324 | 0.504168 | 0.8984647 | 0.731071 | 20 |
| ITGA2 | 0.0170508 | 0.050673 | 0.7365042 | 0.04271047 | 20 |
| MOCS2 | -0.0146877 | 0.0654031 | 0.8223122 | 0.1223875 | 20 |
| FST | 0.149822 | 0.360458 | 0.6776717 | 0.2295491 | 20 |
| SNX18 | -0.108265 | 0.452455 | 0.8108859 | 0.6293352 | 20 |
| ESM1 | -0.552448 | 0.172396 | 0.001352799 | 0.07137168 | 20 |
| GZMA | -1.01059 | 0.524109 | 0.05383001 | 0.5881073 | 20 |
| IL31RA | 0.00938433 | 0.0494714 | 0.8495504 | 0.6169205 | 20 |
| IL6ST | -0.111807 | 0.168208 | 0.5062442 | 0.7646311 | 20 |
| OCLN | 0.201084 | 0.239252 | 0.4006453 | 0.2759233 | 11 |
| CERT | -0.254186 | 0.556583 | 0.6478934 | 0.4603082 | 20 |
| IQGAP2 | 0.598954 | 0.201838 | 0.003002255 | 0.9981161 | 20 |
| F2R | 0.10931 | 0.239893 | 0.6486326 | 0.3562967 | 20 |
| CRHBP | 0.0846224 | 0.0509382 | 0.09665827 | 0.1012232 | 20 |
| TBCA | 0.010154 | 0.22466 | 0.96395 | 0.8742711 | 20 |
| AP3B1 | 0.911322 | 0.653169 | 0.162946 | 0.0246031 | 18 |
| ARSB | -0.00910411 | 0.0981211 | 0.9260748 | 0.7804333 | 20 |
| THBS4 | -0.231315 | 0.125228 | 0.06472622 | 0.7050429 | 20 |
| XRCC4 | 0.0230922 | 0.349551 | 0.947328 | 0.8496059 | 20 |
| VCAN | -0.00259055 | 0.0439278 | 0.9529736 | 0.2587573 | 20 |
| EDIL3 | 0.462355 | 0.381775 | 0.2258693 | 0.8649283 | 10 |
| CETN3 | -0.0522035 | 0.209672 | 0.8033787 | 0.938458 | 20 |
| LYSMD3 | 0.475872 | 0.530316 | 0.3695395 | 0.8297684 | 20 |
| FAM172A | 0.0642612 | 0.379747 | 0.8656226 | 0.2724007 | 20 |
| GLRX | 0.078131 | 0.0748552 | 0.2965954 | 0.5853596 | 20 |
| RGMB | 0.179611 | 0.167796 | 0.2844338 | 0.9883131 | 20 |
| PAM | -0.0348837 | 0.0550156 | 0.5260361 | 0.3670475 | 20 |
| TNFAIP8 | -0.0303975 | 0.34623 | 0.9300391 | 0.8419691 | 20 |
| SNX2 | -0.191947 | 0.307537 | 0.5325349 | 0.08631086 | 20 |
| LMNB1 | 0.311491 | 0.731481 | 0.6702266 | 0.1539426 | 20 |
| MEGF10 | -0.0712733 | 0.0739436 | 0.3351025 | 0.3895935 | 20 |
| FBN2 | -0.256803 | 0.100184 | 0.01036769 | 0.02374864 | 20 |
| CSF2 | -0.0899531 | 0.125045 | 0.4719146 | 0.4225044 | 20 |
| SEPTIN8 | 0.178285 | 0.220653 | 0.4190989 | 0.9214159 | 20 |
| TXNDC15 | 0.00153538 | 0.0303859 | 0.9597004 | 0.05055314 | 20 |
| CXCL14 | -0.550577 | 0.405164 | 0.1741786 | 0.2647802 | 20 |
| LECT2 | 0.0346423 | 0.0376366 | 0.3573415 | 0.3564069 | 20 |
| TGFBI | -0.0694403 | 0.0525645 | 0.1864853 | 0.8763263 | 20 |
| SPOCK1 | -0.118426 | 0.18438 | 0.5206843 | 0.7809142 | 3 |
| GFRA3 | -0.084152 | 0.382811 | 0.8260062 | 0.570066 | 20 |
| SIL1 | -0.133611 | 0.483437 | 0.7822584 | 0.8126941 | 20 |
| MZB1 | 0.266 | 0.30603 | 0.3847411 | 0.9131018 | 20 |
| HBEGF | 0.677118 | 0.587804 | 0.2493438 | 0.2443752 | 20 |
| CD14 | 0.0686994 | 0.0977661 | 0.4822483 | 0.6477674 | 20 |
| PCDHB15 | -0.0549965 | 0.0804492 | 0.4942165 | 0.1573351 | 20 |
| PCDH1 | -0.574377 | 0.357733 | 0.1083612 | 0.9084773 | 20 |
| PCDH12 | -0.121909 | 0.0871864 | 0.1620346 | 0.4667997 | 20 |
| GNPDA1 | -0.100419 | 0.283148 | 0.7228501 | 0.9435243 | 20 |
| SPINK1 | -0.0885878 | 0.0767445 | 0.2483684 | 0.2824151 | 20 |
| SCGB3A2 | 0.151219 | 0.149167 | 0.3106967 | 0.1489769 | 20 |
| SPINK5 | -0.0783797 | 0.0720841 | 0.2768878 | 0.707717 | 20 |
| SPINK6 | -0.0373431 | 0.0799867 | 0.6405957 | 0.9028164 | 20 |
| CSF1R | -0.202783 | 0.225055 | 0.3675688 | 0.4972833 | 20 |
| PDGFRB | 0.0178944 | 0.0246898 | 0.4685932 | 0.2548992 | 20 |
| TCOF1 | 0.658209 | 0.916007 | 0.4724103 | 0.7954317 | 8 |
| CD74 | 1.12628 | 0.672475 | 0.09396769 | 0.4072853 | 17 |
| GM2A | 0.0317473 | 0.0432853 | 0.4632888 | 0.224168 | 20 |
| SPARC | 1.89528 | 1.15972 | 0.1022055 | 0.03110628 | 4 |
| ATOX1 | -0.157904 | 0.404912 | 0.696558 | 0.7022861 | 20 |
| GALNT10 | 0.164159 | 0.0783656 | 0.03618983 | 0.1799453 | 20 |
| TIMD4 | 0.0216685 | 0.130026 | 0.8676476 | 0.8438711 | 20 |
| HAVCR1 | -0.0405682 | 0.0605698 | 0.5030008 | 0.591286 | 20 |
| HAVCR2 | -0.0244759 | 0.0930772 | 0.7925791 | 0.11135 | 12 |
| IL12B | 0.0456138 | 0.0586203 | 0.4364963 | 0.1519195 | 20 |
| IL12B | 0.04701 | 0.0604149 | 0.4364985 | 0.1690788 | 20 |
| FABP6 | 0.192548 | 0.216927 | 0.3747473 | 0.1292147 | 20 |
| STC2 | 0.214651 | 0.430108 | 0.6177352 | 0.2355252 | 18 |
| CDHR2 | -0.124018 | 0.279521 | 0.6572735 | 0.8505726 | 20 |
| FGFR4 | 0.0171575 | 0.0298971 | 0.5660441 | 0.1883956 | 20 |
| F12 | 0.0374627 | 0.0274442 | 0.1722374 | 0.8721297 | 20 |
| DBN1 | 0.483303 | 0.61337 | 0.4307273 | 0.6736658 | 12 |
| PDLIM7 | 0.144084 | 0.681663 | 0.8325977 | 0.7640157 | 7 |
| PHYKPL | -0.0603122 | 0.139341 | 0.6651313 | 0.1498177 | 13 |
| MAPK9 | -0.0605727 | 0.251995 | 0.8100412 | 0.07992056 | 20 |
| SCGB3A1 | 0.0170702 | 0.255445 | 0.9467205 | 0.8962097 | 20 |
| FLT4 | -0.019932 | 0.0976062 | 0.8381905 | 0.3428349 | 20 |
| SERPINB1 | 0.400983 | 0.383313 | 0.295516 | 0.7490865 | 20 |
| SERPINB9 | -0.0953417 | 0.274667 | 0.7285029 | 0.1953977 | 20 |
| SERPINB6 | -0.0152905 | 0.177044 | 0.9311757 | 0.3203057 | 20 |
| PSMG4 | 0.227745 | 0.213933 | 0.2870732 | 0.1461442 | 20 |
| ECI2 | -0.0221582 | 0.118574 | 0.8517603 | 0.3463795 | 20 |
| BMP6 | -0.113434 | 0.212904 | 0.5941779 | 0.227124 | 20 |
| TXNDC5 | 0.0193479 | 0.247913 | 0.9377938 | 0.9878528 | 20 |
| EDN1 | 0.0923869 | 0.133166 | 0.4878248 | 0.09514728 | 20 |
| CD83 | -0.0789861 | 0.175058 | 0.6518458 | 0.3774602 | 20 |
| GMPR | 0.124925 | 0.112848 | 0.2682841 | 0.2675935 | 20 |
| TPMT | 0.0862514 | 0.0758286 | 0.2553492 | 0.3070385 | 20 |
| ALDH5A1 | 0.0191296 | 0.202811 | 0.9248529 | 0.3662674 | 20 |
| KIAA0319 | 0.0204834 | 0.0964708 | 0.8318516 | 0.4257486 | 20 |
| SCGN | -0.0507776 | 0.370979 | 0.8911297 | 0.6055463 | 20 |
| BTN3A2 | 0.0289695 | 0.0522975 | 0.5796223 | 0.2160854 | 20 |
| BTN2A1 | -0.105034 | 0.139568 | 0.4517101 | 0.5488 | 20 |
| BTN1A1 | -0.222675 | 0.12413 | 0.07283298 | 0.7894002 | 20 |
| MOG | -0.108102 | 0.210537 | 0.6076296 | 0.3609479 | 20 |
| HLA-A | -0.0555327 | 0.030427 | 0.06798445 | 0.4953234 | 20 |
| TRIM40 | 0.00742641 | 0.114134 | 0.9481202 | 0.5018683 | 20 |
| HLA-E | -0.055371 | 0.124199 | 0.6557243 | 0.9953232 | 20 |
| DDR1 | 0.041868 | 0.08222 | 0.6105984 | 0.7027732 | 20 |
| HCG22 | 0.0460203 | 0.0746003 | 0.5373062 | 0.9974572 | 20 |
| CDSN | 0.0447981 | 0.045678 | 0.3267225 | 0.8221545 | 20 |
| MICA | 0.00346668 | 0.027163 | 0.8984457 | 0.9201535 | 20 |
| MICB | 0.00346668 | 0.027163 | 0.8984457 | 0.9201535 | 20 |
| ATP6V1G2 | -0.13129 | 0.241988 | 0.5874407 | 0.1158424 | 20 |
| LTA | -0.0351399 | 0.0420314 | 0.4031322 | 0.9299854 | 20 |
| TNF | -0.279254 | 0.306063 | 0.3615544 | 0.6453942 | 20 |
| TNF | -0.304743 | 0.33419 | 0.3618294 | 0.7856397 | 20 |
| TNF | -0.272054 | 0.298138 | 0.3614998 | 0.883934 | 20 |
| TNF | -0.16816 | 0.357214 | 0.6378167 | 0.7053174 | 20 |
| LTB | -0.14911 | 0.176045 | 0.3969974 | 0.8230668 | 20 |
| AIF1 | 0.259507 | 0.260196 | 0.3185936 | 0.5774055 | 20 |
| APOM | -0.168013 | 0.230319 | 0.4657085 | 0.04484074 | 20 |
| HSPA1A | -0.360958 | 0.517979 | 0.4858917 | 0.1555455 | 14 |
| C2 | 0.181579 | 0.0937125 | 0.05267041 | 0.4630615 | 20 |
| CFB | 0.120526 | 0.0915173 | 0.1878459 | 0.3284889 | 20 |
| DXO | 0.0769513 | 0.0718056 | 0.2838717 | 0.1586261 | 20 |
| TNXB | -0.0188565 | 0.128901 | 0.8836955 | 0.7758222 | 20 |
| FKBPL | -0.964762 | 0.738266 | 0.191283 | 0.8985766 | 6 |
| AGER | -0.223353 | 0.219069 | 0.3079409 | 0.7928726 | 20 |
| HLA-DRA | -0.00977266 | 0.0530157 | 0.8537504 | 0.1303364 | 20 |
| MLN | -0.0481883 | 0.0710809 | 0.4978123 | 0.8075085 | 20 |
| FKBP5 | -0.089611 | 0.154585 | 0.5621254 | 0.170653 | 20 |
| CLPS | 0.000433509 | 0.0525235 | 0.9934146 | 0.9786896 | 20 |
| MAPK13 | -0.0437957 | 0.530922 | 0.9342572 | 0.7184095 | 20 |
| CDKN1A | -0.00659136 | 0.234196 | 0.9775468 | 0.6374826 | 20 |
| RAB44 | -0.0156642 | 0.260276 | 0.9520097 | 0.385493 | 20 |
| PI16 | 0.0424836 | 0.106455 | 0.6898383 | 0.1780797 | 20 |
| MDGA1 | -0.0209497 | 0.0267921 | 0.4342534 | 0.2505726 | 20 |
| GLO1 | -0.0226475 | 0.0895472 | 0.8003366 | 0.6806239 | 20 |
| TREM2 | -0.0156557 | 0.110003 | 0.8868263 | 0.03915649 | 7 |
| TREML2 | 0.185161 | 0.112417 | 0.09954005 | 0.2456781 | 20 |
| TBCC | 0.0496173 | 0.164694 | 0.7632096 | 0.2008255 | 9 |
| PTK7 | -0.258058 | 0.532344 | 0.6278484 | 0.9504012 | 20 |
| DNPH1 | -0.0211765 | 0.110979 | 0.8486699 | 0.9426728 | 20 |
| TJAP1 | -0.869164 | 1.11092 | 0.4339891 | 0.5218486 | 9 |
| VEGFA | 0.00100227 | 0.0415902 | 0.9807739 | 0.4550593 | 20 |
| NFKBIE | -0.0457227 | 0.110912 | 0.6801602 | 0.5153431 | 20 |
| CLIC5 | 0.0508552 | 0.245867 | 0.8361345 | 0.6604237 | 20 |
| ENPP5 | 0.00821696 | 0.0405208 | 0.8393039 | 0.7513022 | 20 |
| PLA2G7 | 0.624281 | 0.343882 | 0.06946413 | 0.06928015 | 20 |
| MEP1A | -0.142074 | 0.152049 | 0.3500991 | 0.1363107 | 20 |
| TNFRSF21 | 0.30631 | 0.271656 | 0.2595033 | 0.5871563 | 15 |
| CD2AP | 0.0821542 | 0.10902 | 0.4511069 | 0.1335051 | 20 |
| MMUT | 0.100424 | 0.286463 | 0.7259149 | 0.2875799 | 20 |
| CRISP2 | -0.0563299 | 0.0584497 | 0.3351801 | 0.6384406 | 20 |
| CRISP3 | -0.0511741 | 0.0501101 | 0.3071444 | 0.4570151 | 20 |
| IL17F | -0.141815 | 0.216617 | 0.5126715 | 0.7937036 | 20 |
| GSTA1 | -0.026405 | 0.0913116 | 0.772448 | 0.03197289 | 20 |
| GSTA3 | -0.0266299 | 0.0920897 | 0.7724484 | 0.04435828 | 20 |
| GFRAL | 0.00934542 | 0.095579 | 0.9221094 | 0.9863697 | 15 |
| ADGRB3 | 0.00091303 | 0.0312767 | 0.9767114 | 0.7536252 | 20 |
| COL9A1 | 0.160279 | 0.199173 | 0.4209795 | 0.2541679 | 20 |
| CD109 | 0.0516825 | 0.036124 | 0.1525171 | 0.2871963 | 20 |
| NT5E | 0.080693 | 0.0501722 | 0.107765 | 0.9871309 | 14 |
| CGA | -1.3226 | 0.863524 | 0.1256141 | 0.8513527 | 20 |
| GRIK2 | -0.0191243 | 0.0965552 | 0.842993 | 0.9262774 | 20 |
| RTN4IP1 | -0.483328 | 0.320236 | 0.1312252 | 0.6281466 | 20 |
| CD164 | 0.461383 | 0.198187 | 0.01991145 | 0.9072382 | 20 |
| WASF1 | -0.000853248 | 0.0993753 | 0.9931494 | 0.5049193 | 20 |
| LAMA4 | 0.0346506 | 0.171498 | 0.8398806 | 0.4688514 | 20 |
| TSPYL1 | 0.105648 | 0.207607 | 0.6108348 | 0.3235888 | 20 |
| RWDD1 | -0.0513347 | 0.441467 | 0.907429 | 0.5624243 | 20 |
| SMPDL3A | 0.0311914 | 0.0363922 | 0.391395 | 0.4442589 | 20 |
| HDDC2 | 0.014226 | 0.0539195 | 0.791905 | 0.4028134 | 20 |
| RSPO3 | 0.0539396 | 0.181581 | 0.7664237 | 0.123403 | 20 |
| PTPRK | -0.00144154 | 0.337201 | 0.996589 | 0.4063559 | 20 |
| ARG1 | -0.0279572 | 0.14248 | 0.8444397 | 0.3609946 | 20 |
| CCN2 | 0.016428 | 0.0914249 | 0.8573968 | 0.002016379 | 20 |
| STX7 | 0.0546708 | 0.0483998 | 0.258659 | 0.1848045 | 20 |
| VNN1 | -0.00532593 | 0.0338263 | 0.8748907 | 0.5069213 | 20 |
| VNN2 | 0.0650131 | 0.0430092 | 0.130633 | 0.2991622 | 20 |
| IFNGR1 | -0.124374 | 0.166672 | 0.4555356 | 0.8725756 | 20 |
| PHACTR2 | -0.0489577 | 0.145332 | 0.7362175 | 0.8615967 | 20 |
| TAB2 | -0.67502 | 0.598909 | 0.2597075 | 0.7158627 | 20 |
| LATS1 | -0.436612 | 0.459093 | 0.3415884 | 0.6706921 | 20 |
| LRP11 | 0.0426705 | 0.0483763 | 0.3777482 | 0.7842364 | 20 |
| ULBP2 | 0.103102 | 0.0561367 | 0.06626601 | 0.07750618 | 20 |
| AKAP12 | -0.84635 | 0.849724 | 0.3192361 | 0.9855428 | 16 |
| IPCEF1 | -0.264634 | 0.347781 | 0.4467041 | 0.6215959 | 20 |
| SNX9 | 0.0148707 | 0.344634 | 0.9655826 | 0.3098703 | 20 |
| DYNLT1 | -0.0279283 | 0.353432 | 0.9370164 | 0.4899443 | 20 |
| EZR | 0.790624 | 0.423232 | 0.06175293 | 0.2768734 | 20 |
| FNDC1 | 0.195722 | 0.0699012 | 0.005110629 | 0.08301691 | 15 |
| SOD2 | -0.300229 | 0.190631 | 0.1152745 | 0.1607534 | 20 |
| IGF2R | -0.105112 | 0.0796677 | 0.1870431 | 0.1005485 | 20 |
| LPA | 0.0738609 | 0.0487063 | 0.1294044 | 0.001308853 | 20 |
| PLG | -0.170696 | 0.207366 | 0.4104137 | 0.4657528 | 10 |
| RNASET2 | -0.00895854 | 0.070487 | 0.8988653 | 0.01143681 | 20 |
| CEP43 | 1.21879 | 0.459726 | 0.0080223 | 0.9619145 | 14 |
| SMOC2 | 0.00798466 | 0.0782529 | 0.9187275 | 0.7017409 | 20 |
| THBS2 | -0.0335636 | 0.0627183 | 0.5925474 | 0.8531501 | 20 |
| DLL1 | -0.116584 | 0.182707 | 0.5234135 | 0.1703437 | 20 |
| PDGFA | -0.347006 | 0.636372 | 0.585555 | 0.291847 | 14 |
| C7orf50 | 0.182747 | 0.0867623 | 0.03517838 | 0.04209412 | 20 |
| MICALL2 | 0.208886 | 0.415102 | 0.6148128 | 0.8054273 | 20 |
| MAD1L1 | 0.105472 | 0.297761 | 0.7231779 | 0.2242385 | 20 |
| COL28A1 | 0.0428238 | 0.0354562 | 0.2271259 | 0.1402721 | 20 |
| ANKMY2 | -0.0500992 | 0.363787 | 0.8904652 | 0.9008095 | 17 |
| AGR2 | -0.445167 | 0.267243 | 0.09575816 | 0.480655 | 20 |
| GPNMB | -0.168944 | 0.0973631 | 0.0827071 | 0.1461142 | 16 |
| NPY | 0.271398 | 0.185087 | 0.1425594 | 0.05697749 | 20 |
| CPVL | 0.0190695 | 0.0506917 | 0.7067786 | 0.3822222 | 20 |
| SCRN1 | 0.304744 | 0.324855 | 0.3481966 | 0.4320961 | 20 |
| GGCT | 0.386953 | 0.261373 | 0.1387495 | 0.997843 | 20 |
| NT5C3A | 0.42045 | 0.285079 | 0.1402512 | 0.4850739 | 20 |
| BMPER | 0.0333486 | 0.153383 | 0.8278807 | 0.2621331 | 20 |
| SFRP4 | -0.0831114 | 0.142359 | 0.5593457 | 0.2484271 | 20 |
| DBNL | -0.628009 | 0.540512 | 0.2452854 | 0.1053305 | 20 |
| IGFBP1 | 0.0765233 | 0.641944 | 0.9051126 | 0.4309583 | 20 |
| IGFBP3 | 0.0683813 | 0.0648043 | 0.2913357 | 0.1494331 | 20 |
| VWC2 | -0.0141456 | 0.0622105 | 0.8201255 | 0.2475955 | 20 |
| DDC | -0.0830853 | 0.101615 | 0.413559 | 0.3077001 | 20 |
| EGFR | 0.0168786 | 0.167682 | 0.9198216 | 0.4695022 | 20 |
| SUMF2 | 0.0627683 | 0.0901728 | 0.4863725 | 0.8830257 | 20 |
| GUSB | -0.102125 | 0.245685 | 0.6776464 | 0.3272158 | 20 |
| ELN | 0.60228 | 0.437314 | 0.1684423 | 0.3451811 | 20 |
| LAT2 | 1.29355 | 0.610763 | 0.0341812 | 0.5785329 | 13 |
| CLIP2 | -0.174751 | 0.209814 | 0.4049098 | 0.9886911 | 20 |
| HIP1 | -0.235031 | 0.677951 | 0.7288333 | 0.9860079 | 15 |
| CCL26 | 0.0571319 | 0.116805 | 0.6247545 | 0.09084749 | 10 |
| CCL24 | 0.0058627 | 0.0399744 | 0.8833994 | 0.9781786 | 20 |
| HSPB1 | 0.00934551 | 0.0664695 | 0.8881872 | 0.2320364 | 20 |
| SSC4D | 0.0212557 | 0.10384 | 0.8378092 | 0.1867185 | 20 |
| ZP3 | 0.00890274 | 0.0270072 | 0.7416694 | 0.6604015 | 20 |
| CD36 | -0.0528772 | 0.105826 | 0.6173118 | 0.2314916 | 20 |
| HGF | 0.0434997 | 0.377555 | 0.9082752 | 0.6111284 | 20 |
| ADAM22 | 0.115875 | 0.0507556 | 0.02243018 | 0.03796838 | 20 |
| SAMD9L | 0.0456064 | 0.127796 | 0.721189 | 0.7583402 | 20 |
| TFPI2 | 0.267356 | 0.216131 | 0.2160831 | 0.5247361 | 8 |
| PON1 | -0.0482442 | 0.0393412 | 0.2200854 | 0.4518358 | 20 |
| PON3 | 0.0578184 | 0.110033 | 0.5992608 | 0.4338596 | 20 |
| PON2 | 0.00370077 | 0.0320747 | 0.9081443 | 0.3461334 | 20 |
| NPTX2 | -0.0440081 | 0.0891442 | 0.6215368 | 0.3878946 | 20 |
| CNPY4 | 0.0856458 | 0.186455 | 0.6459921 | 0.8672631 | 20 |
| PILRB | -0.0284775 | 0.026939 | 0.29046 | 0.5930882 | 20 |
| PILRA | -0.0326372 | 0.0308741 | 0.2904639 | 0.5951188 | 20 |
| LRCH4 | -0.483542 | 0.422833 | 0.2527988 | 0.9541553 | 20 |
| PCOLCE | 0.076497 | 0.166585 | 0.6460862 | 0.5344695 | 20 |
| EPO | 0.169969 | 0.370388 | 0.6463103 | 0.5458946 | 20 |
| EPHB4 | 0.13727 | 0.185387 | 0.4590272 | 0.1891917 | 20 |
| ACHE | -0.044416 | 0.0914472 | 0.6271794 | 0.7536297 | 20 |
| SERPINE1 | 0.246064 | 0.3139 | 0.4331037 | 0.995076 | 20 |
| VGF | 0.375146 | 0.407388 | 0.3571255 | 0.9899688 | 20 |
| FIS1 | -0.0714004 | 0.174585 | 0.6825594 | 0.2231679 | 20 |
| NAMPT | 0.405769 | 0.641264 | 0.5268875 | 0.3636395 | 9 |
| LAMB1 | -0.0366523 | 0.0448243 | 0.4135346 | 0.9497891 | 20 |
| NRCAM | 0.00790791 | 0.103523 | 0.9391105 | 0.3521376 | 20 |
| MET | 0.149832 | 0.18036 | 0.4061232 | 0.3385468 | 17 |
| FAM3C | -0.0353736 | 0.29059 | 0.9031128 | 0.5839196 | 17 |
| PTPRZ1 | -0.219814 | 0.170276 | 0.1967286 | 0.8933079 | 20 |
| GPR37 | 0.0100371 | 0.0464187 | 0.828808 | 0.2742179 | 20 |
| CPA2 | -0.19986 | 0.0788916 | 0.01129794 | 0.0595351 | 20 |
| CPA4 | -0.11943 | 0.0549385 | 0.02971344 | 0.5908961 | 20 |
| CPA1 | 0.118443 | 0.180903 | 0.5126406 | 0.03071225 | 20 |
| PODXL | 0.115402 | 0.123736 | 0.3509996 | 0.7923175 | 20 |
| PLXNA4 | -0.270424 | 0.317888 | 0.3949417 | 0.9257854 | 20 |
| AKR1B1 | 0.163313 | 0.220973 | 0.4598685 | 0.4312594 | 12 |
| PTN | 0.216762 | 0.190491 | 0.2551573 | 0.02212039 | 20 |
| CLEC5A | -0.00316884 | 0.106079 | 0.9761687 | 0.4666117 | 20 |
| PRSS2 | -0.213967 | 0.210998 | 0.3105503 | 0.007261167 | 20 |
| EPHB6 | 0.0213201 | 0.101426 | 0.8335091 | 0.9069456 | 20 |
| KEL | 0.0354082 | 0.151218 | 0.8148657 | 0.9690501 | 10 |
| EPHA1 | 0.220525 | 0.156179 | 0.1579477 | 0.4106852 | 20 |
| ARHGEF5 | 0.026266 | 0.0729626 | 0.7188525 | 0.3943652 | 20 |
| TPK1 | -0.0568292 | 0.0908053 | 0.5314224 | 0.4056411 | 20 |
| CNTNAP2 | -0.0715533 | 0.0579394 | 0.2168419 | 0.4365815 | 20 |
| PDIA4 | 0.156069 | 0.379124 | 0.6805912 | 0.2674598 | 20 |
| RARRES2 | -0.0346085 | 0.148623 | 0.8158692 | 0.7143205 | 20 |
| GIMAP8 | -0.0254326 | 0.275099 | 0.9263414 | 0.9112069 | 9 |
| GIMAP7 | -0.0165687 | 0.0500944 | 0.7408334 | 0.1295366 | 20 |
| AOC1 | 0.105207 | 0.111143 | 0.3438475 | 0.04740418 | 20 |
| NOS3 | -1.17646 | 0.668127 | 0.07826784 | 0.2281286 | 15 |
| NUB1 | -0.126772 | 0.16049 | 0.4295839 | 0.8549531 | 11 |
| DPP6 | 0.563562 | 0.54376 | 0.3000074 | 0.5713268 | 20 |
| DNAJB6 | 0.0765529 | 0.151594 | 0.6135676 | 0.6261397 | 20 |
| PTPRN2 | -0.147175 | 0.637355 | 0.8173807 | 0.2615579 | 9 |
| ESYT2 | -0.0277791 | 0.311373 | 0.9289113 | 0.6100227 | 20 |
| ARHGEF10 | -0.101097 | 0.102457 | 0.3237778 | 0.6183693 | 20 |
| MYOM2 | -1.04836 | 0.758209 | 0.1667632 | 0.03926151 | 13 |
| ANGPT2 | -0.186813 | 0.187896 | 0.3201068 | 0.1256156 | 20 |
| DEFA1 | -0.122693 | 0.141517 | 0.3859498 | 0.05376828 | 20 |
| DEFA1B | -0.122693 | 0.141517 | 0.3859498 | 0.05376828 | 20 |
| DEFB104B | 0.0788104 | 0.346573 | 0.8201131 | 0.9805501 | 3 |
| DEFB104A | 0.0788104 | 0.346573 | 0.8201131 | 0.1957801 | 4 |
| DEFB4A | -0.240843 | 0.287807 | 0.4026932 | 0.07699194 | 3 |
| ERI1 | 0.836842 | 0.423145 | 0.04796598 | 0.6213289 | 20 |
| MSRA | -0.64176 | 0.532716 | 0.2283211 | 0.5157313 | 20 |
| CTSB | 0.0446124 | 0.060613 | 0.4617181 | 0.3617986 | 20 |
| MSR1 | -0.0117864 | 0.0989453 | 0.9051799 | 0.7398758 | 4 |
| MTUS1 | -0.302461 | 0.246274 | 0.2193903 | 0.2300591 | 20 |
| FGL1 | -0.00853505 | 0.0466861 | 0.8549409 | 0.5527977 | 20 |
| ASAH1 | 0.0799602 | 0.0602783 | 0.1846689 | 0.4427958 | 20 |
| LPL | -0.133285 | 0.214571 | 0.5344883 | 0.2753221 | 20 |
| GFRA2 | -0.0531225 | 0.092198 | 0.5644949 | 0.4939343 | 20 |
| DOK2 | -0.15452 | 0.461334 | 0.7376692 | 0.3348282 | 20 |
| REEP4 | 0.102661 | 0.138853 | 0.4596934 | 0.7631916 | 20 |
| SLC39A14 | 0.164472 | 0.290138 | 0.5707978 | 0.1922744 | 20 |
| TNFRSF10B | 0.070643 | 0.106512 | 0.5071786 | 0.2924394 | 20 |
| TNFRSF10C | -0.0179108 | 0.110894 | 0.8716889 | 0.9430651 | 20 |
| TNFRSF10A | 0.0672343 | 0.0617878 | 0.2765301 | 0.8467491 | 20 |
| STC1 | 0.669995 | 0.530718 | 0.2067938 | 0.4420037 | 18 |
| BNIP3L | 0.521946 | 0.585331 | 0.3725474 | 0.9053044 | 20 |
| EPHX2 | 0.33565 | 0.329405 | 0.3082216 | 0.06527662 | 20 |
| CLU | 0.0280988 | 0.306355 | 0.9269208 | 0.2320091 | 20 |
| SCARA5 | 0.186372 | 0.0932446 | 0.04563634 | 0.9147424 | 20 |
| GSR | -0.297905 | 0.292394 | 0.3082755 | 0.482389 | 20 |
| UNC5D | 0.0596607 | 0.450658 | 0.8946791 | 0.7420822 | 20 |
| PLPBP | -0.240811 | 0.651712 | 0.7117515 | 0.9892082 | 9 |
| DDHD2 | 0.0777441 | 0.334415 | 0.8161669 | 0.4643425 | 20 |
| IDO1 | 0.0267833 | 0.119022 | 0.821958 | 0.1836917 | 20 |
| IDO1 | -0.0128776 | 0.11933 | 0.9140627 | 0.3050565 | 20 |
| IDO1 | 0.0275529 | 0.122443 | 0.8219582 | 0.180837 | 20 |
| IDO1 | -0.0124141 | 0.115035 | 0.9140626 | 0.3091852 | 20 |
| SFRP1 | 0.119219 | 0.151626 | 0.4317086 | 0.6581382 | 20 |
| PLAT | 0.227585 | 0.570429 | 0.6899136 | 0.4505385 | 20 |
| DKK4 | -0.0556902 | 0.243257 | 0.8189186 | 0.1566349 | 20 |
| FNTA | 0.385199 | 0.493903 | 0.4354448 | 0.1483189 | 20 |
| PXDNL | -0.158558 | 0.11786 | 0.178527 | 0.2403854 | 20 |
| PENK | -0.00458928 | 0.0948604 | 0.961414 | 0.04897561 | 20 |
| GGH | 0.00743283 | 0.0664657 | 0.9109586 | 0.3502185 | 20 |
| CRH | 0.00146429 | 0.0790374 | 0.9852188 | 0.8311348 | 20 |
| LACTB2 | -0.0883242 | 0.256678 | 0.7307672 | 0.5285293 | 5 |
| LY96 | 0.722784 | 0.460237 | 0.1163081 | 0.5952429 | 20 |
| IL7 | 0.310616 | 0.27342 | 0.2559401 | 0.3319994 | 20 |
| PAG1 | 0.546292 | 0.412013 | 0.1848697 | 0.8573943 | 20 |
| FABP5 | -0.140953 | 0.205425 | 0.492616 | 0.536509 | 10 |
| FABP9 | 0.355518 | 0.370528 | 0.3373108 | 0.3857716 | 20 |
| FABP4 | 0.387119 | 0.344422 | 0.2610273 | 0.3384431 | 20 |
| IMPA1 | -0.117172 | 0.137919 | 0.3955606 | 0.4515973 | 20 |
| CA13 | -0.121335 | 0.126569 | 0.3377369 | 0.2269504 | 20 |
| CA1 | -0.255175 | 0.342624 | 0.4564124 | 0.7040467 | 20 |
| CA3 | 0.337843 | 0.2195 | 0.1237681 | 0.7663497 | 20 |
| CA2 | -0.3706 | 0.289019 | 0.1997489 | 0.7694726 | 20 |
| DECR1 | -0.578288 | 0.431444 | 0.18013 | 0.6051223 | 14 |
| CALB1 | 0.136709 | 0.475774 | 0.7738512 | 0.8857409 | 19 |
| CDH17 | 0.141544 | 0.106972 | 0.1857733 | 0.4653388 | 20 |
| CPQ | -0.0222491 | 0.077496 | 0.7740357 | 0.1946254 | 18 |
| MATN2 | -0.0140643 | 0.0728168 | 0.8468442 | 0.2296101 | 20 |
| RIDA | -0.0625691 | 0.0965924 | 0.5171372 | 0.8517632 | 20 |
| SPAG1 | 0.0908398 | 0.0405613 | 0.0251189 | 0.6796957 | 20 |
| RRM2B | -0.0216144 | 0.303138 | 0.9431572 | 0.3880075 | 20 |
| CTHRC1 | 0.00356067 | 0.175674 | 0.983829 | 0.9697131 | 20 |
| ANGPT1 | 0.333897 | 0.526461 | 0.5259307 | 0.7145607 | 20 |
| EBAG9 | 0.226283 | 0.430783 | 0.5993854 | 0.704791 | 20 |
| TNFRSF11B | 0.238233 | 0.152865 | 0.1191261 | 0.7040734 | 20 |
| CCN3 | -0.270314 | 0.129384 | 0.03668654 | 0.09970636 | 20 |
| ENPP2 | 0.0324591 | 0.115061 | 0.777863 | 0.03773707 | 20 |
| MTSS1 | 0.612669 | 0.592441 | 0.3010693 | 0.5156091 | 8 |
| TG | -0.000746877 | 0.381468 | 0.9984378 | 0.4527414 | 18 |
| CCN4 | -0.0246554 | 0.0658741 | 0.7081952 | 0.9692968 | 20 |
| ST3GAL1 | -0.0938861 | 0.123167 | 0.445902 | 0.2972197 | 20 |
| PSCA | 0.024633 | 0.0245025 | 0.3147395 | 0.3977374 | 20 |
| SLURP1 | 0.111087 | 0.0703857 | 0.1145052 | 0.004243757 | 20 |
| LY6D | -0.0486128 | 0.0663362 | 0.4636653 | 0.1475442 | 20 |
| GPIHBP1 | 0.0932235 | 0.105245 | 0.3757404 | 0.7126511 | 20 |
| NAPRT | 0.140462 | 0.0844415 | 0.0962264 | 0.9254835 | 20 |
| EPPK1 | -0.10127 | 0.0770108 | 0.1885041 | 0.4901542 | 20 |
| OPLAH | -0.0691361 | 0.155702 | 0.6570214 | 0.1383621 | 20 |
| SMARCA2 | -0.180108 | 0.643377 | 0.7795219 | 0.7803586 | 20 |
| INSL4 | 0.0956163 | 0.18726 | 0.6096267 | 0.8166944 | 20 |
| RLN2 | 0.172393 | 0.231762 | 0.4569737 | 0.7015827 | 20 |
| CD274 | 0.0382411 | 0.093746 | 0.6833308 | 0.5980751 | 20 |
| PDCD1LG2 | -0.0630802 | 0.0480419 | 0.1891747 | 0.9824361 | 20 |
| TYRP1 | 0.0503465 | 0.374863 | 0.8931604 | 0.6489111 | 20 |
| TEK | -0.0893072 | 0.0771748 | 0.2471879 | 0.2022794 | 20 |
| DDX58 | 0.131598 | 0.10702 | 0.2188243 | 0.9537095 | 20 |
| B4GALT1 | 0.157846 | 0.132083 | 0.2320698 | 0.6923832 | 20 |
| SPINK4 | 0.0160529 | 0.0364562 | 0.6596961 | 0.3901932 | 20 |
| NUDT2 | 0.0887489 | 0.113296 | 0.4334307 | 0.6550675 | 20 |
| CCL27 | 0.0402518 | 0.138198 | 0.7708506 | 0.6223699 | 20 |
| CCL19 | 0.1894 | 0.375598 | 0.6140773 | 0.5622926 | 20 |
| CCL21 | 0.552549 | 0.308383 | 0.07317066 | 0.7071416 | 20 |
| CD72 | 0.0183593 | 0.213902 | 0.931601 | 0.7070322 | 20 |
| CA9 | -0.382919 | 0.314431 | 0.2232952 | 0.4034172 | 20 |
| RECK | 0.429559 | 0.258835 | 0.09699824 | 0.4271845 | 20 |
| GRHPR | 0.0927559 | 0.109298 | 0.3960749 | 0.9630986 | 20 |
| IGFBPL1 | 0.016189 | 0.0669194 | 0.8088434 | 0.478562 | 20 |
| FXN | -0.433145 | 0.370461 | 0.242321 | 0.914922 | 20 |
| MAMDC2 | -0.112295 | 0.145838 | 0.4413024 | 0.9044919 | 20 |
| CEMIP2 | 0.345527 | 0.198746 | 0.08211748 | 0.3729478 | 20 |
| ALDH1A1 | 0.40923 | 0.377298 | 0.2780833 | 0.7822696 | 20 |
| GCNT1 | 0.0537948 | 0.0782721 | 0.4919066 | 0.2862302 | 20 |
| NTRK2 | -0.119974 | 0.346172 | 0.7289128 | 0.5066345 | 6 |
| CTSL | 0.0882169 | 0.192961 | 0.6475448 | 0.7612014 | 20 |
| SEMA4D | 0.576152 | 0.327001 | 0.0780816 | 0.4600422 | 20 |
| SPTLC1 | 0.0714283 | 0.176584 | 0.685845 | 0.9951846 | 20 |
| OGN | -0.0979782 | 0.0521159 | 0.06010735 | 0.2346316 | 20 |
| OMD | -0.761724 | 0.469178 | 0.1044767 | 0.09925315 | 20 |
| ASPN | 0.214405 | 0.123612 | 0.0828277 | 0.1453933 | 20 |
| NINJ1 | 0.248369 | 0.126158 | 0.04898684 | 0.8225364 | 20 |
| FBP1 | 0.0742071 | 0.109933 | 0.4996637 | 0.3691664 | 20 |
| CTSV | 0.136596 | 0.130731 | 0.2960854 | 0.01571688 | 20 |
| COL15A1 | -0.0093411 | 0.151733 | 0.9509111 | 0.8636499 | 20 |
| TGFBR1 | 0.351817 | 0.564149 | 0.5328744 | 0.3196753 | 6 |
| RAD23B | 0.255637 | 0.449056 | 0.5691689 | 0.1486395 | 20 |
| PALM2 | -0.0318081 | 0.143173 | 0.8241849 | 2.92E-05 | 20 |
| TXN | 0.0951758 | 0.555675 | 0.864004 | 0.6075513 | 11 |
| PTGR1 | 0.0777064 | 0.106367 | 0.4650556 | 0.9822607 | 20 |
| SUSD1 | -0.0139661 | 0.267875 | 0.9584198 | 0.05319651 | 20 |
| HSDL2 | -0.0230835 | 0.0475021 | 0.6270043 | 0.6821637 | 20 |
| AMBP | -0.155725 | 0.255446 | 0.542114 | 0.9195457 | 4 |
| ORM1 | -0.049087 | 0.137104 | 0.7203227 | 0.5151666 | 20 |
| TNFSF8 | 0.045575 | 0.123003 | 0.7109956 | 0.9982726 | 20 |
| TNC | -0.148957 | 0.131787 | 0.2583546 | 0.8220787 | 20 |
| PAPPA | -0.187421 | 0.449927 | 0.6770008 | 0.8148032 | 20 |
| TLR4 | 0.162125 | 0.145578 | 0.2654247 | 0.6739454 | 20 |
| MEGF9 | 0.0778429 | 0.094912 | 0.4121254 | 0.8395014 | 20 |
| C5 | 0.353968 | 0.201046 | 0.07830151 | 0.2887029 | 20 |
| GSN | -0.567467 | 0.554485 | 0.3061122 | 0.7152595 | 19 |
| RABEPK | 0.219119 | 0.209775 | 0.2962343 | 0.2567141 | 20 |
| ANGPTL2 | 0.326325 | 0.693458 | 0.6379432 | 0.6518194 | 20 |
| STXBP1 | 0.466954 | 0.565804 | 0.4092058 | 0.7461309 | 20 |
| ENG | -0.0538253 | 0.111305 | 0.6286803 | 0.7764071 | 20 |
| PTGES2 | -0.916552 | 0.492929 | 0.06297053 | 0.152574 | 12 |
| LCN2 | -0.216713 | 0.761996 | 0.7761021 | 0.4328199 | 10 |
| DNM1 | 0.115365 | 0.106765 | 0.2798944 | 0.7248485 | 20 |
| SLC27A4 | -0.429758 | 0.512148 | 0.4013977 | 0.2737709 | 20 |
| PKN3 | 0.140839 | 0.215072 | 0.5125665 | 0.9219333 | 20 |
| KYAT1 | 0.118092 | 0.247989 | 0.633932 | 0.2194119 | 20 |
| SH3GLB2 | 0.0494512 | 0.107149 | 0.644428 | 0.3596066 | 20 |
| NCS1 | -0.238736 | 0.251146 | 0.3418141 | 0.9548965 | 13 |
| HMCN2 | 0.0550723 | 0.0456109 | 0.2272635 | 0.8482771 | 20 |
| ABL1 | 0.467118 | 0.443425 | 0.2921424 | 0.5774837 | 10 |
| AIF1L | -0.0283303 | 0.104494 | 0.7863005 | 0.7560897 | 20 |
| OBP2B | 0.0505708 | 0.0437728 | 0.2479661 | 0.9524477 | 20 |
| ABO | 0.0416146 | 0.032851 | 0.2052373 | 0.2705177 | 20 |
| ADAMTS13 | -0.136527 | 0.090846 | 0.132879 | 0.2134841 | 20 |
| ADAMTSL2 | -0.0288653 | 0.286173 | 0.9196563 | 0.1750016 | 20 |
| DBH | -0.0262491 | 0.032488 | 0.419112 | 0.8351661 | 20 |
| COL5A1 | 0.179878 | 0.129047 | 0.1633484 | 0.9003223 | 20 |
| FCN2 | 0.00973341 | 0.045856 | 0.8319037 | 0.5317359 | 20 |
| FCN1 | -0.00376466 | 0.0674779 | 0.9555084 | 0.8580174 | 20 |
| PAEP | -0.0860562 | 0.0487034 | 0.07723777 | 0.6027506 | 20 |
| UBAC1 | -0.267275 | 0.28807 | 0.3535058 | 0.1611069 | 7 |
| ENTR1 | -0.140058 | 0.161786 | 0.3866526 | 0.8847294 | 20 |
| NOTCH1 | 0.0736221 | 0.772353 | 0.9240592 | 0.3251399 | 20 |
| EGFL7 | -0.144295 | 0.132315 | 0.2754743 | 0.3006276 | 20 |
| LCN15 | 0.0216256 | 0.0473708 | 0.6480185 | 0.9356591 | 20 |
| MAMDC4 | -0.159575 | 0.153667 | 0.2990616 | 0.1906391 | 20 |
| EDF1 | -0.143905 | 0.68375 | 0.8333051 | 0.4092232 | 20 |
| TRAF2 | -0.255647 | 0.528879 | 0.62883 | 0.2142355 | 20 |
| PTGDS | 0.095474 | 0.14636 | 0.5141916 | 0.5516789 | 20 |
| PAXX | 0.321526 | 0.181952 | 0.07721293 | 0.261955 | 20 |
| ABCA2 | 0.626382 | 0.507263 | 0.2168944 | 0.4893941 | 18 |
| NPDC1 | -0.0126632 | 0.265999 | 0.96203 | 0.4159236 | 20 |
| ENTPD2 | -0.123187 | 0.105246 | 0.2418138 | 0.2527105 | 9 |
| DPP7 | -0.0523317 | 0.0593795 | 0.378151 | 0.5458039 | 20 |
| SSNA1 | -0.615681 | 0.632665 | 0.3304761 | 0.6783981 | 14 |
| IDI2 | -0.087546 | 0.105375 | 0.4060849 | 0.1049585 | 20 |
| AKR1C4 | 1.0235 | 0.595629 | 0.08573311 | 0.1610579 | 20 |
| IL15RA | -0.049239 | 0.120313 | 0.6823501 | 0.9388601 | 20 |
| IL2RA | -0.0369437 | 0.0598371 | 0.5369677 | 0.2066063 | 20 |
| RBM17 | -0.0680813 | 0.156045 | 0.6626246 | 0.7725537 | 20 |
| ITIH5 | 0.112874 | 0.244277 | 0.6440272 | 0.9821449 | 7 |
| ECHDC3 | 0.0766285 | 0.0679969 | 0.2597676 | 0.8699212 | 20 |
| NUDT5 | 0.416943 | 0.504969 | 0.4089857 | 0.1049445 | 20 |
| CDNF | 0.0321848 | 0.0579262 | 0.578473 | 0.3328596 | 12 |
| TRDMT1 | -0.0234281 | 0.196209 | 0.9049556 | 0.02312218 | 20 |
| MRC1 | 0.148284 | 0.258672 | 0.5664746 | 0.4302686 | 20 |
| PLXDC2 | -0.160304 | 0.396272 | 0.6858219 | 0.3474627 | 19 |
| PRTFDC1 | -0.0305365 | 0.0969936 | 0.7528905 | 0.8296955 | 14 |
| GPR158 | 0.100801 | 0.454902 | 0.8246349 | 0.3409907 | 20 |
| APBB1IP | 0.0280235 | 0.141353 | 0.8428475 | 0.6326267 | 20 |
| BAMBI | -0.0685898 | 0.678937 | 0.9195304 | 0.7610958 | 20 |
| LYZL2 | 0.0161501 | 0.130948 | 0.9018441 | 0.966551 | 20 |
| NRP1 | 0.0484671 | 0.0971762 | 0.617952 | 0.03628414 | 20 |
| PARD3 | 0.352054 | 0.599023 | 0.5567244 | 0.9467975 | 16 |
| RET | 0.191367 | 0.0857679 | 0.0256667 | 0.04404997 | 20 |
| CXCL12 | 0.137997 | 0.156218 | 0.377042 | 0.4560209 | 20 |
| ASAH2 | -0.0395001 | 0.051355 | 0.4417995 | 0.7779116 | 20 |
| PRKG1 | -0.338673 | 0.430587 | 0.4315521 | 0.6177522 | 20 |
| DKK1 | 0.235054 | 0.280848 | 0.4026233 | 0.7748765 | 20 |
| MBL2 | -0.0138943 | 0.0331742 | 0.675342 | 0.4459002 | 20 |
| PBLD | -0.0109223 | 0.0536279 | 0.8386121 | 0.2090998 | 20 |
| KIFBP | 0.388986 | 0.636228 | 0.5409391 | 0.6782617 | 20 |
| TSPAN15 | -0.957868 | 0.536037 | 0.07394642 | 0.8642885 | 20 |
| PCBD1 | 0.177556 | 0.164975 | 0.2818106 | 0.3684763 | 20 |
| CDH23 | -0.0541522 | 0.241953 | 0.8229029 | 0.6268701 | 20 |
| VSIR | 0.0292695 | 0.0945361 | 0.7568563 | 0.2708509 | 20 |
| PSAP | -0.0968376 | 0.140783 | 0.4915472 | 0.2760448 | 20 |
| PLAU | -0.223803 | 0.215098 | 0.2981199 | 0.7047541 | 8 |
| DUSP29 | -0.0347073 | 0.208037 | 0.867502 | 0.9715993 | 20 |
| DUSP13 | 0.0498769 | 0.107442 | 0.6424879 | 0.8822822 | 20 |
| SFTPA2 | -0.00296405 | 0.0434574 | 0.9456217 | 0.8488466 | 20 |
| SFTPA1 | -0.0176529 | 0.121688 | 0.884658 | 0.05313777 | 20 |
| SFTPD | -0.0582412 | 0.0384011 | 0.1293542 | 0.6413437 | 20 |
| ANXA11 | 0.19481 | 0.467683 | 0.6770126 | 0.1320122 | 20 |
| CDHR1 | 0.191419 | 0.130586 | 0.1426899 | 0.2987449 | 20 |
| SNCG | 0.0220905 | 0.0335384 | 0.5101128 | 0.9371254 | 20 |
| LIPF | 0.133723 | 0.110051 | 0.2243252 | 0.5925252 | 20 |
| ACTA2 | -0.102158 | 0.474389 | 0.8294971 | 0.37746 | 18 |
| FAS | -0.124498 | 0.112348 | 0.267799 | 0.04966933 | 20 |
| IFIT3 | 0.548668 | 0.322771 | 0.08915632 | 0.8475369 | 20 |
| FGFBP3 | -0.09087 | 0.0825524 | 0.2710032 | 0.7939056 | 20 |
| SORBS1 | -0.280311 | 0.602352 | 0.6416736 | 0.131056 | 20 |
| TCTN3 | 0.0107248 | 0.0488015 | 0.8260553 | 0.68611 | 20 |
| BLNK | -0.120524 | 0.438436 | 0.7833973 | 0.866472 | 20 |
| PIK3AP1 | 0.102976 | 0.15482 | 0.5059666 | 0.9563034 | 20 |
| CRTAC1 | -0.0312573 | 0.0498412 | 0.5305684 | 0.3168512 | 20 |
| DNMBP | 0.680004 | 0.327978 | 0.03814201 | 0.1077488 | 20 |
| KAZALD1 | 0.0429873 | 0.0311849 | 0.1680592 | 0.01002686 | 20 |
| OGA | 0.44492 | 0.234762 | 0.05806613 | 0.4939711 | 20 |
| VTI1A | 0.113979 | 0.620216 | 0.854192 | 0.2585575 | 13 |
| CASP7 | -0.162715 | 0.103673 | 0.1165318 | 0.6356904 | 5 |
| GFRA1 | 0.00149697 | 0.120755 | 0.9901091 | 0.3762496 | 20 |
| PNLIPRP1 | 0.223291 | 0.131548 | 0.0896199 | 0.7917516 | 20 |
| PNLIPRP2 | 0.0204365 | 0.0261305 | 0.4341593 | 0.02736818 | 20 |
| PRDX3 | -0.601497 | 0.482627 | 0.2126547 | 0.4765347 | 20 |
| GRK5 | -0.111636 | 0.139046 | 0.4220499 | 0.2209199 | 20 |
| BAG3 | 0.555071 | 0.442112 | 0.2092986 | 0.8879747 | 20 |
| FGFR2 | 0.252196 | 0.257486 | 0.3273539 | 0.5014695 | 20 |
| ACADSB | 0.0207252 | 0.130506 | 0.8738218 | 0.4687546 | 20 |
| CPXM2 | 0.0289072 | 0.0601798 | 0.6309806 | 0.02787766 | 20 |
| LHPP | -0.0248885 | 0.0752816 | 0.7409426 | 0.9221993 | 20 |
| UROS | -0.525314 | 0.291013 | 0.07105567 | 0.5236053 | 20 |
| ADAM12 | -0.0557121 | 0.226502 | 0.8057076 | 0.3970939 | 20 |
| MGMT | -0.00508433 | 0.0699738 | 0.9420763 | 0.05680413 | 20 |
| ADAM8 | 0.0113904 | 0.0686244 | 0.8681714 | 0.4041381 | 20 |
| PRAP1 | -0.00056713 | 0.118666 | 0.9961867 | 0.9627453 | 20 |
| FUOM | 0.331796 | 0.355135 | 0.3501588 | 0.3567627 | 20 |
| ECHS1 | 0.206036 | 0.18771 | 0.2723681 | 0.76577 | 20 |
| CDHR5 | -0.0885793 | 0.0549827 | 0.1071711 | 0.1050613 | 20 |
| EPS8L2 | 0.206397 | 0.109372 | 0.05914559 | 0.7884306 | 20 |
| TALDO1 | -0.192598 | 0.798123 | 0.8093121 | 0.1912462 | 9 |
| MUC2 | -0.0644125 | 0.126519 | 0.6106716 | 0.08143811 | 20 |
| BRSK2 | -0.00955105 | 0.0490313 | 0.8455533 | 0.132326 | 20 |
| CTSD | 0.0935672 | 0.121305 | 0.4405077 | 0.4397434 | 20 |
| NAP1L4 | 0.105214 | 0.126081 | 0.4040006 | 0.3874131 | 20 |
| ART5 | -0.256923 | 0.157473 | 0.1027773 | 0.07578657 | 20 |
| TRIM21 | 0.201464 | 0.689673 | 0.770199 | 0.5393733 | 20 |
| TRIM5 | 0.146231 | 0.109192 | 0.1805017 | 0.8250541 | 20 |
| SMPD1 | 0.11065 | 0.07976 | 0.1653561 | 0.6262088 | 20 |
| TPP1 | -0.187113 | 0.194947 | 0.3371484 | 0.3614738 | 20 |
| CYB5R2 | 0.0672818 | 0.115929 | 0.5616638 | 0.2084408 | 20 |
| SWAP70 | -0.0306658 | 0.227851 | 0.8929383 | 0.803658 | 20 |
| ADM | -0.00216319 | 0.179605 | 0.9903904 | 0.9675336 | 20 |
| AMPD3 | -0.0677895 | 0.0826155 | 0.4119074 | 0.2502964 | 8 |
| LYVE1 | -0.0939337 | 0.159821 | 0.5567059 | 0.4358899 | 20 |
| DKK3 | 0.142115 | 0.0802768 | 0.07667632 | 0.211645 | 20 |
| SPON1 | -0.029807 | 0.0994253 | 0.7643349 | 0.2281911 | 20 |
| CALCB | 0.405249 | 0.311354 | 0.1930643 | 0.7905253 | 8 |
| CALCA | -0.0364071 | 0.285747 | 0.8986157 | 0.8716198 | 20 |
| NUCB2 | -0.0283663 | 0.0706863 | 0.6882006 | 0.9535912 | 20 |
| NCR3LG1 | 0.0333103 | 0.0794024 | 0.6748413 | 0.6061888 | 20 |
| SAA4 | 0.0226814 | 0.083059 | 0.7847948 | 0.6775332 | 20 |
| NELL1 | 0.139048 | 0.0833908 | 0.09542879 | 0.3175373 | 20 |
| GAS2 | -0.600991 | 0.273501 | 0.02799202 | 0.5698422 | 12 |
| LUZP2 | -0.0571921 | 0.20504 | 0.7802971 | 0.8891323 | 20 |
| BDNF | -0.268316 | 0.387395 | 0.4885501 | 0.9501422 | 6 |
| FSHB | -0.388708 | 0.257935 | 0.1318109 | 0.0207881 | 20 |
| CD59 | -0.153022 | 0.119649 | 0.2009232 | 0.1551763 | 20 |
| CAT | -0.14833 | 0.191756 | 0.4392042 | 0.1143738 | 20 |
| PAMR1 | -0.0479164 | 0.0942888 | 0.6113219 | 0.848954 | 17 |
| MDK | -0.00385205 | 0.434597 | 0.992928 | 0.534404 | 6 |
| F2 | 0.0139598 | 0.274139 | 0.9593874 | 0.7757077 | 8 |
| FOLH1 | -0.164746 | 0.385317 | 0.6689716 | 0.5738426 | 12 |
| PRG3 | 0.053567 | 0.208581 | 0.7973206 | 0.9810229 | 20 |
| PRG2 | 0.912846 | 0.378454 | 0.01586355 | 0.02415564 | 20 |
| TIMM10 | -0.0635467 | 0.169287 | 0.7073786 | 0.4452748 | 20 |
| UBE2L6 | 0.0916103 | 0.124013 | 0.4600803 | 0.5495551 | 20 |
| SERPING1 | 0.206443 | 0.149168 | 0.1663697 | 0.2053671 | 15 |
| CBLIF | 0.573406 | 0.505212 | 0.2563828 | 0.223371 | 7 |
| TCN1 | 0.0477788 | 0.0613312 | 0.4359629 | 0.2028669 | 20 |
| TMEM132A | -0.0218994 | 0.0476664 | 0.6459252 | 0.259076 | 20 |
| CD6 | -0.00996091 | 0.0410389 | 0.8082232 | 0.2819746 | 20 |
| CD5 | 0.171294 | 0.230814 | 0.4580093 | 0.7134244 | 20 |
| PGA4 | 0.2616 | 0.561563 | 0.6413279 | 0.0636301 | 14 |
| ASRGL1 | 0.00871117 | 0.0834134 | 0.9168252 | 0.6212846 | 20 |
| SCGB1A1 | 0.0335402 | 0.0888569 | 0.7058297 | 0.6582423 | 20 |
| AHNAK | 0.173837 | 0.2549 | 0.4952519 | 0.594207 | 19 |
| VEGFB | 0.391961 | 0.280547 | 0.1623751 | 0.0894308 | 20 |
| PRDX5 | 0.65476 | 0.463895 | 0.1581148 | 0.1854375 | 20 |
| GPHA2 | 0.0400859 | 0.221991 | 0.8567014 | 0.09001249 | 20 |
| SNX15 | 0.11346 | 0.0805617 | 0.1590236 | 0.3883442 | 20 |
| LTBP3 | -0.0390801 | 0.0714206 | 0.5842537 | 0.9906575 | 20 |
| CST6 | -0.0251126 | 0.154222 | 0.870649 | 0.2391293 | 20 |
| CD248 | 0.715738 | 1.05472 | 0.4973904 | 0.9018114 | 10 |
| B4GAT1 | -0.125525 | 0.106475 | 0.2384333 | 0.8534613 | 20 |
| CTSF | -0.0570773 | 0.160681 | 0.7224236 | 0.2020254 | 20 |
| CCS | -0.035301 | 0.0596208 | 0.5537892 | 0.5214174 | 20 |
| GSTP1 | 0.0637519 | 0.145544 | 0.6613673 | 0.7756609 | 20 |
| ACY3 | 0.130423 | 0.12104 | 0.2812463 | 0.4332062 | 20 |
| GAL | 0.136791 | 0.459204 | 0.7657897 | 0.8992137 | 20 |
| IGHMBP2 | 0.111429 | 0.365141 | 0.7602387 | 0.04850797 | 20 |
| FADD | 0.169894 | 0.35963 | 0.6366316 | 0.4458973 | 20 |
| IL18BP | 0.12152 | 0.238579 | 0.6105088 | 0.3528553 | 14 |
| FOLR3 | 0.0515981 | 0.0313389 | 0.09966995 | 0.4523051 | 20 |
| FOLR1 | 0.462354 | 0.309025 | 0.1346098 | 0.4931831 | 20 |
| FOLR2 | -0.0732298 | 0.234222 | 0.754546 | 0.9565144 | 20 |
| RELT | -0.127392 | 0.0789417 | 0.1065816 | 0.9170768 | 20 |
| RAB6A | -1.2454 | 0.533609 | 0.0196003 | 0.5784969 | 20 |
| PPME1 | -0.938786 | 0.781094 | 0.2294079 | 0.003257051 | 18 |
| CHRDL2 | -0.0197798 | 0.107647 | 0.8542125 | 0.4351618 | 20 |
| SERPINH1 | 0.283816 | 0.212478 | 0.1816339 | 0.8544518 | 20 |
| OMP | -0.0624618 | 0.116577 | 0.5920985 | 0.652936 | 20 |
| AAMDC | -0.00332937 | 0.03411 | 0.9222445 | 0.853794 | 20 |
| PRCP | 0.16522 | 0.416333 | 0.691481 | 0.08036413 | 20 |
| CTSC | -0.0241327 | 0.0567011 | 0.6703897 | 0.1011482 | 20 |
| CWC15 | 0.697391 | 0.530932 | 0.1890064 | 0.6404623 | 20 |
| CNTN5 | -0.0591135 | 0.0592723 | 0.3186089 | 0.939779 | 20 |
| YAP1 | 0.419588 | 0.461301 | 0.3630462 | 0.9901357 | 20 |
| MMP7 | -0.0853571 | 0.101856 | 0.4020206 | 0.04578756 | 20 |
| MMP8 | 0.153785 | 0.0834046 | 0.06520587 | 0.5895449 | 20 |
| MMP10 | -0.00304284 | 0.100703 | 0.9758948 | 0.6972906 | 20 |
| MMP1 | 0.00404651 | 0.0663353 | 0.9513585 | 0.690327 | 20 |
| MMP3 | -0.00732184 | 0.0603041 | 0.903362 | 0.2467251 | 20 |
| MMP12 | -0.00709719 | 0.0491928 | 0.8852851 | 0.7120015 | 20 |
| MMP13 | -0.283456 | 0.209325 | 0.1756904 | 0.7170945 | 20 |
| CASP1 | -0.441818 | 0.528215 | 0.40291 | 0.4492596 | 15 |
| FDX1 | 0.19364 | 0.548321 | 0.7239756 | 0.8606667 | 20 |
| LAYN | -0.0771583 | 0.0788856 | 0.328023 | 0.09425251 | 20 |
| IL18 | -0.00314128 | 0.0974393 | 0.974282 | 0.819868 | 20 |
| PTS | 0.049667 | 0.137585 | 0.7181051 | 0.1302928 | 20 |
| NCAM1 | -0.109693 | 0.0991083 | 0.2683795 | 0.08644664 | 20 |
| TMPRSS5 | 0.0054921 | 0.0374412 | 0.8833799 | 0.007001898 | 20 |
| USP28 | -0.841585 | 0.421973 | 0.04610811 | 0.7734472 | 20 |
| ZBTB16 | 0.0590366 | 0.0994169 | 0.5526269 | 0.05645999 | 20 |
| ZPR1 | 0.674669 | 0.572894 | 0.2389356 | 0.08629758 | 20 |
| APOA4 | -0.180396 | 0.202451 | 0.3728967 | 0.6527283 | 20 |
| APOA1 | 0.638131 | 0.458159 | 0.1636753 | 0.876852 | 20 |
| PCSK7 | -0.0297488 | 0.0829409 | 0.7198389 | 0.1718775 | 20 |
| IL10RA | 0.378984 | 0.189564 | 0.04558223 | 0.5143849 | 20 |
| SCN4B | 0.0372854 | 0.0452756 | 0.4102121 | 0.5474987 | 20 |
| TMEM25 | -0.233227 | 0.106077 | 0.027902 | 0.2228232 | 20 |
| PHLDB1 | 1.83651 | 0.72961 | 0.0118321 | 0.4181407 | 20 |
| TREH | -0.0486615 | 0.0343784 | 0.1569313 | 0.01643458 | 20 |
| HYOU1 | 0.109727 | 0.170595 | 0.5200935 | 0.5016101 | 20 |
| HMBS | -0.244122 | 0.319446 | 0.4447469 | 0.5991531 | 20 |
| C2CD2L | -0.240482 | 0.533826 | 0.6523584 | 0.5529825 | 18 |
| MCAM | -0.44118 | 0.208797 | 0.03460391 | 0.498876 | 11 |
| C1QTNF5 | 0.106985 | 0.222316 | 0.6303532 | 0.256945 | 20 |
| THY1 | 0.0727536 | 0.0851913 | 0.3931032 | 0.4229846 | 13 |
| NECTIN1 | -0.336775 | 0.670143 | 0.615286 | 0.9506724 | 11 |
| CRTAM | -0.0748281 | 0.0929061 | 0.4205793 | 0.09412248 | 20 |
| CLMP | -0.0252359 | 0.0879723 | 0.7742177 | 0.2334844 | 20 |
| SIAE | -0.0456601 | 0.132662 | 0.7307075 | 0.8082555 | 20 |
| VSIG2 | -0.139012 | 0.148646 | 0.3496911 | 0.6926023 | 20 |
| ESAM | 0.186713 | 0.199768 | 0.3499687 | 0.9086831 | 20 |
| ROBO4 | 0.414417 | 0.328669 | 0.2073457 | 0.8917042 | 20 |
| ACRV1 | 0.203503 | 0.604445 | 0.7363603 | 0.6663137 | 20 |
| CDON | -0.15561 | 0.291828 | 0.5938771 | 0.3404712 | 11 |
| ADAMTS8 | 0.0443467 | 0.0659891 | 0.5015642 | 0.3454531 | 20 |
| ADAMTS15 | -0.284161 | 0.343806 | 0.408513 | 0.192646 | 18 |
| JAM3 | 0.22335 | 0.255765 | 0.3825208 | 0.2696178 | 20 |
| LRTM2 | -0.0508834 | 0.130705 | 0.6970555 | 0.1053145 | 20 |
| FKBP4 | 1.03059 | 0.59691 | 0.08425135 | 0.03067626 | 8 |
| CRACR2A | -0.0367037 | 0.226829 | 0.8714537 | 0.1973409 | 20 |
| TIGAR | 0.74733 | 0.481858 | 0.1209177 | 0.01133035 | 11 |
| FGF23 | 0.248992 | 0.276094 | 0.3671436 | 0.376823 | 20 |
| NTF3 | -0.171988 | 0.23197 | 0.4584361 | 0.2302 | 20 |
| VWF | -0.308441 | 0.314016 | 0.3259775 | 0.3642173 | 20 |
| LTBR | -0.0430616 | 0.100776 | 0.6691597 | 0.7082556 | 20 |
| CD27 | 0.170723 | 0.124653 | 0.1708165 | 0.07469575 | 20 |
| ACRBP | 0.34418 | 0.199333 | 0.08422916 | 0.007368849 | 20 |
| LAG3 | 0.0835563 | 0.165241 | 0.6130934 | 0.08267397 | 9 |
| CD4 | -0.0322353 | 0.0874366 | 0.712373 | 0.350585 | 20 |
| ENO2 | -0.267703 | 0.232489 | 0.2495406 | 0.4364596 | 6 |
| C1S | 0.219411 | 0.25602 | 0.3914406 | 0.009481999 | 12 |
| C1R | -0.453959 | 0.497065 | 0.3610954 | 0.1153638 | 5 |
| C1RL | -0.198791 | 0.316627 | 0.530109 | 0.1411818 | 5 |
| CLSTN3 | 0.00543623 | 0.411733 | 0.9894656 | 0.3663662 | 4 |
| KLRB1 | -1.34633 | 0.938357 | 0.151352 | 0.009258173 | 6 |
| KLRF1 | -0.0461087 | 0.201014 | 0.8185736 | 0.2874113 | 17 |
| CLEC12A | 0.0462084 | 0.0674157 | 0.4930761 | 0.04383145 | 20 |
| CLEC1B | -0.440439 | 0.198398 | 0.02642019 | 0.967163 | 20 |
| CLEC1A | 0.0107117 | 0.0936499 | 0.9089364 | 0.4154196 | 20 |
| CLEC7A | 0.0366615 | 0.0386606 | 0.3429824 | 0.6012404 | 20 |
| OLR1 | 0.281803 | 0.32032 | 0.3789924 | 0.771963 | 20 |
| KLRD1 | 0.00463107 | 0.0692643 | 0.9466925 | 0.2741817 | 20 |
| KLRK1 | -0.0635019 | 0.0575894 | 0.2701719 | 0.2852522 | 20 |
| PRR4 | 0.00578014 | 0.0373857 | 0.8771301 | 0.8538111 | 20 |
| HEBP1 | 0.20877 | 0.29981 | 0.486215 | 0.8425285 | 12 |
| BCAT1 | -0.00769078 | 0.119829 | 0.948826 | 0.8317771 | 20 |
| MANSC4 | 0.0164467 | 0.0374714 | 0.6607247 | 0.01124597 | 20 |
| CNTN1 | 0.0113808 | 0.146024 | 0.9378776 | 0.3132187 | 20 |
| IRAK4 | 0.539303 | 0.709765 | 0.4473542 | 0.3793259 | 11 |
| NELL2 | -0.00134781 | 0.172331 | 0.9937597 | 0.1719239 | 20 |
| AMIGO2 | 0.0503406 | 0.182093 | 0.7821985 | 0.9400636 | 20 |
| ENDOU | 0.0245209 | 0.101313 | 0.8087554 | 0.3709444 | 20 |
| COL2A1 | 0.0296417 | 0.0383917 | 0.4400636 | 0.7055765 | 20 |
| CACNB3 | -0.102726 | 0.308967 | 0.7395247 | 0.8298612 | 20 |
| GPD1 | 0.251593 | 0.302226 | 0.4051458 | 0.322089 | 13 |
| BIN2 | 0.243512 | 0.305383 | 0.4252193 | 0.164086 | 20 |
| ACVRL1 | 0.0924597 | 0.0808688 | 0.2529021 | 0.607775 | 20 |
| KRT5 | -0.459054 | 0.293382 | 0.1176538 | 0.2933045 | 20 |
| KRT18 | 0.376902 | 0.61965 | 0.5430218 | 0.6014707 | 20 |
| IGFBP6 | 0.114593 | 0.298355 | 0.7009163 | 0.633565 | 20 |
| ITGB7 | 0.125586 | 0.231014 | 0.5866975 | 0.4026 | 20 |
| CALCOCO1 | -0.541201 | 0.229216 | 0.01822112 | 0.3636881 | 17 |
| NFE2 | -0.23641 | 0.465171 | 0.611298 | 0.5858124 | 20 |
| ITGA5 | 0.182465 | 0.484429 | 0.7064263 | 0.2038054 | 9 |
| LACRT | -0.0527939 | 0.453291 | 0.9072815 | 0.05348952 | 20 |
| CD63 | 0.20524 | 0.562868 | 0.7153851 | 0.9462833 | 10 |
| ERBB3 | 0.255063 | 0.374201 | 0.4954798 | 0.009969512 | 13 |
| SLC39A5 | 0.203294 | 0.18224 | 0.2646231 | 0.5290713 | 14 |
| STAT2 | -0.24195 | 0.135207 | 0.07353816 | 0.4717531 | 20 |
| APOF | -0.0331338 | 0.178327 | 0.8525987 | 0.6843823 | 20 |
| INHBC | 0.0450475 | 0.0509292 | 0.37642 | 0.2784242 | 20 |
| DTX3 | 0.0534318 | 0.307413 | 0.8620141 | 0.7846405 | 20 |
| LRIG3 | 0.376865 | 0.408048 | 0.3557058 | 0.9332993 | 20 |
| WIF1 | -0.238259 | 0.151564 | 0.1159511 | 0.8817897 | 20 |
| IFNG | -1.27419 | 0.735076 | 0.08302329 | 0.003331073 | 19 |
| IL22 | -0.299768 | 0.3067 | 0.3283706 | 0.6365462 | 20 |
| MDM1 | -0.0598813 | 0.157339 | 0.7035084 | 0.01514066 | 20 |
| CPM | 0.202956 | 0.534819 | 0.7043272 | 0.5489299 | 17 |
| PTPRB | -0.0545428 | 0.0919514 | 0.5530675 | 0.1868716 | 20 |
| TSPAN8 | -0.0740916 | 0.0626584 | 0.2370198 | 0.04959741 | 20 |
| KITLG | -0.265826 | 0.559575 | 0.6347519 | 0.8193373 | 3 |
| DCN | 0.76074 | 0.724468 | 0.2936875 | 0.2301015 | 20 |
| CRADD | 0.201679 | 0.408695 | 0.6216791 | 0.8820964 | 20 |
| LTA4H | -0.0984035 | 0.283777 | 0.728769 | 0.005265298 | 20 |
| WASHC3 | -0.271048 | 0.312554 | 0.3858295 | 0.8102338 | 20 |
| STAB2 | -0.0457641 | 0.0694756 | 0.5100833 | 0.8884605 | 20 |
| GLT8D2 | -0.0377698 | 0.108707 | 0.7282566 | 0.4211936 | 20 |
| TXNRD1 | -0.643807 | 0.594663 | 0.2789669 | 0.463201 | 19 |
| APPL2 | -0.072608 | 0.222248 | 0.7438958 | 0.05736256 | 20 |
| CKAP4 | 0.0857216 | 0.144719 | 0.5536276 | 0.4803037 | 20 |
| SELPLG | 0.0276312 | 0.0371499 | 0.4570123 | 0.5470536 | 20 |
| UNG | 0.754608 | 0.653493 | 0.2482004 | 0.6289422 | 4 |
| MVK | -0.00479995 | 0.339245 | 0.9887112 | 0.4859295 | 20 |
| SH2B3 | -0.128999 | 0.440936 | 0.7698609 | 0.7593355 | 20 |
| NOS1 | 0.220747 | 0.227449 | 0.3317797 | 0.9950858 | 20 |
| VSIG10 | 0.0284158 | 0.0407645 | 0.4857578 | 0.2395447 | 20 |
| PEBP1 | -0.0333496 | 0.364412 | 0.9270823 | 0.8935853 | 11 |
| PRKAB1 | -0.218021 | 0.153815 | 0.1563605 | 0.9857546 | 20 |
| CIT | 0.401338 | 0.548915 | 0.4646891 | 0.6780686 | 13 |
| PXN | -0.0554898 | 0.0447872 | 0.2153586 | 0.02279716 | 20 |
| PLA2G1B | 0.114193 | 0.501905 | 0.8200194 | 0.1006637 | 20 |
| PSMD9 | -0.0311662 | 0.152928 | 0.838513 | 0.5252876 | 20 |
| HIP1R | 0.15613 | 0.318864 | 0.6243866 | 0.1927748 | 20 |
| RILPL2 | 0.522953 | 0.540704 | 0.3334582 | 0.4244603 | 8 |
| ADGRD1 | -0.0803348 | 0.0757054 | 0.2886212 | 0.06710955 | 20 |
| GOLGA3 | 0.0180403 | 0.262473 | 0.9452029 | 0.1042668 | 20 |
| MPHOSPH8 | 0.527727 | 0.853811 | 0.5365201 | 0.7981651 | 20 |
| IL17D | -0.000514626 | 0.081598 | 0.9949679 | 0.713259 | 20 |
| TNFRSF19 | -0.116087 | 0.117169 | 0.3218016 | 0.8206107 | 20 |
| C1QTNF9 | 0.0028763 | 0.0324144 | 0.9292922 | 0.3666582 | 20 |
| WASF3 | 0.210037 | 0.215434 | 0.3295859 | 0.06112867 | 20 |
| MTIF3 | 0.0417229 | 0.260563 | 0.872782 | 0.1672292 | 20 |
| FLT3 | 0.453258 | 0.349077 | 0.1941333 | 0.7826952 | 20 |
| POSTN | 0.0139397 | 0.112161 | 0.9010911 | 0.8443983 | 20 |
| NHLRC3 | 0.0145273 | 0.157627 | 0.9265689 | 0.9199587 | 20 |
| FOXO1 | -0.439306 | 0.41049 | 0.2845288 | 0.8878518 | 18 |
| TNFSF11 | -0.176446 | 0.261509 | 0.4998532 | 0.4614133 | 20 |
| TSC22D1 | -0.105626 | 0.642426 | 0.8694029 | 0.9890864 | 20 |
| CPB2 | 0.0956371 | 0.0539501 | 0.07627898 | 0.3826604 | 20 |
| LCP1 | -0.0811267 | 0.037851 | 0.03208765 | 0.09300139 | 20 |
| NUDT15 | -0.185264 | 0.23869 | 0.4376479 | 0.9380636 | 5 |
| THSD1 | -0.755197 | 0.377378 | 0.04537411 | 0.6297494 | 7 |
| OLFM4 | 0.0137506 | 0.0358841 | 0.701576 | 0.8963163 | 15 |
| PCDH17 | 0.19956 | 0.250405 | 0.4254819 | 0.627869 | 12 |
| PCDH9 | -0.202966 | 0.209088 | 0.3316868 | 0.3360291 | 10 |
| PIBF1 | 0.0718585 | 0.478847 | 0.8807129 | 0.713471 | 20 |
| SPRY2 | -0.117184 | 0.364987 | 0.7481626 | 0.9244675 | 20 |
| SLITRK1 | -0.02542 | 0.270966 | 0.9252581 | 0.8060805 | 20 |
| SLITRK6 | -0.0571366 | 0.0807077 | 0.4789799 | 0.7360139 | 17 |
| GPC5 | -0.0486604 | 0.0349743 | 0.1641293 | 0.000623749 | 20 |
| GGACT | -0.022594 | 0.0640702 | 0.7243547 | 0.9021413 | 20 |
| ITGBL1 | 0.0797899 | 0.109317 | 0.4654529 | 0.965682 | 20 |
| TNFSF13B | 0.222878 | 0.226103 | 0.3242636 | 0.4528036 | 19 |
| COL4A1 | 0.120428 | 0.173621 | 0.4879161 | 0.09676808 | 20 |
| ING1 | -0.61921 | 0.549737 | 0.2600072 | 0.1340887 | 20 |
| F7 | 0.08399 | 0.0662197 | 0.2046715 | 0.05950691 | 20 |
| F10 | -0.21712 | 0.159764 | 0.1741472 | 0.1713941 | 20 |
| LAMP1 | 0.161188 | 0.455954 | 0.7236993 | 0.4258296 | 20 |
| GAS6 | 0.0128621 | 0.0884955 | 0.8844413 | 0.2515975 | 20 |
| APEX1 | -0.0672732 | 0.0735377 | 0.3602896 | 0.249115 | 20 |
| RNASE10 | 0.0190587 | 0.0358748 | 0.5952425 | 0.401554 | 20 |
| RNASE4 | -0.129138 | 0.0911955 | 0.1567594 | 0.6119696 | 20 |
| ANG | 0.0182398 | 0.0774311 | 0.8137729 | 0.5136189 | 20 |
| EDDM3B | 0.124184 | 0.212015 | 0.5580562 | 0.7970864 | 20 |
| RNASE6 | -0.0313019 | 0.0744309 | 0.6740841 | 0.1712251 | 20 |
| RNASE1 | -0.0520551 | 0.148573 | 0.7260625 | 0.3388762 | 14 |
| RNASE3 | -0.200899 | 0.145892 | 0.168501 | 0.195258 | 20 |
| RAB2B | 0.115673 | 0.1995 | 0.5620416 | 0.8706239 | 20 |
| THTPA | 0.060893 | 0.0650094 | 0.3489233 | 0.9996295 | 16 |
| AP1G2 | 0.237685 | 0.254238 | 0.3498438 | 0.9991128 | 15 |
| DHRS4L2 | 0.00939783 | 0.160256 | 0.9532369 | 0.008333524 | 20 |
| PSME1 | 0.145899 | 0.328635 | 0.6570766 | 0.8894857 | 20 |
| PSME2 | 0.132115 | 0.297541 | 0.6570269 | 0.8905512 | 20 |
| GMPR2 | 0.561779 | 0.413318 | 0.1740853 | 0.6646795 | 20 |
| GZMH | 0.324963 | 0.237792 | 0.1717556 | 0.1307888 | 8 |
| GZMB | -0.0175055 | 0.0993779 | 0.8601753 | 0.1177807 | 20 |
| COCH | 0.0165203 | 0.0917631 | 0.8571277 | 0.9895773 | 20 |
| CLEC14A | -0.0975497 | 0.343547 | 0.7764499 | 0.6132155 | 20 |
| VCPKMT | 0.592926 | 0.808095 | 0.4631115 | 0.5723615 | 9 |
| MAP4K5 | 0.111414 | 0.23593 | 0.6367607 | 0.6038129 | 20 |
| NID2 | 0.019159 | 0.0496757 | 0.6997328 | 0.2373395 | 20 |
| LGALS3 | -0.025651 | 0.0535367 | 0.6318471 | 0.09205693 | 20 |
| HSPA2 | 0.415811 | 0.360462 | 0.2486838 | 0.4894221 | 20 |
| MAX | 0.0614669 | 0.368124 | 0.8673911 | 0.6910734 | 20 |
| FUT8 | 0.0390329 | 0.03987 | 0.3275782 | 0.1606421 | 20 |
| ARG2 | 0.00504964 | 0.472299 | 0.9914695 | 0.3803704 | 20 |
| SMOC1 | -0.319907 | 0.14428 | 0.02660502 | 0.1205821 | 20 |
| NUMB | 0.0482744 | 0.41531 | 0.9074647 | 0.2354903 | 20 |
| PNMA1 | 0.0893861 | 0.35023 | 0.7985524 | 0.9925938 | 20 |
| ENTPD5 | 0.0767235 | 0.0569779 | 0.1781254 | 0.8897413 | 20 |
| LTBP2 | -0.0189498 | 0.299437 | 0.9495398 | 0.2931607 | 20 |
| PGF | -0.44001 | 0.187375 | 0.01885998 | 0.3202561 | 20 |
| VASH1 | 0.192752 | 0.225795 | 0.3932936 | 0.2796523 | 20 |
| TMED8 | -0.534032 | 0.440177 | 0.2250451 | 0.7647031 | 20 |
| SEL1L | -0.0436843 | 0.0965475 | 0.6509342 | 0.9822162 | 20 |
| FLRT2 | -0.0131782 | 0.0533621 | 0.8049404 | 0.6535719 | 20 |
| TDP1 | -0.312399 | 0.296434 | 0.2919478 | 0.8049175 | 20 |
| ATXN3 | -0.10169 | 0.111896 | 0.363459 | 0.000695458 | 20 |
| LGMN | 0.123791 | 0.113994 | 0.2775027 | 0.6014756 | 11 |
| CHGA | -0.207935 | 0.166905 | 0.2128277 | 0.6056675 | 20 |
| SERPINA6 | -0.13284 | 0.133231 | 0.318733 | 0.03732107 | 20 |
| SERPINA1 | -0.172016 | 0.398698 | 0.6661451 | 0.1881811 | 20 |
| SERPINA11 | 0.012077 | 0.0457547 | 0.7918171 | 0.112203 | 20 |
| SERPINA9 | 0.165211 | 0.0718218 | 0.0214318 | 0.07104149 | 20 |
| SERPINA12 | -0.0934982 | 0.0450315 | 0.03786761 | 0.9417418 | 20 |
| SERPINA4 | 0.052997 | 0.0519588 | 0.3077382 | 0.8055718 | 20 |
| SERPINA5 | -0.123103 | 0.142859 | 0.3888472 | 0.1487806 | 20 |
| SERPINA3 | 0.0673088 | 0.0907827 | 0.458434 | 0.6600655 | 20 |
| GLRX5 | -0.168109 | 0.22132 | 0.4475075 | 0.7174409 | 20 |
| TCL1A | 0.0624433 | 0.0936092 | 0.5047317 | 0.2802223 | 20 |
| WARS | 0.305399 | 0.134656 | 0.02332923 | 0.6622253 | 20 |
| DLK1 | 0.0138297 | 0.0581489 | 0.8120102 | 0.5114575 | 20 |
| AMN | 0.0911335 | 0.0389892 | 0.01941821 | 0.211659 | 20 |
| TNFAIP2 | 0.206828 | 0.677863 | 0.7602762 | 0.2336902 | 10 |
| EIF5 | -0.0925046 | 0.417858 | 0.824798 | 0.07893902 | 15 |
| AHNAK2 | -0.0617703 | 0.133339 | 0.6431785 | 0.5091553 | 20 |
| PACS2 | 0.128749 | 0.168127 | 0.4438035 | 0.0577173 | 20 |
| CRIP2 | 0.00352002 | 0.196908 | 0.9857374 | 0.6413608 | 20 |
| SRP14 | 0.536751 | 0.543657 | 0.3234968 | 0.2128078 | 20 |
| PLCB2 | 0.168711 | 0.228169 | 0.4596555 | 0.5395576 | 20 |
| GCHFR | 0.0532957 | 0.257302 | 0.8359059 | 0.5320759 | 20 |
| ZFYVE19 | 0.0451458 | 0.0763401 | 0.5542675 | 0.4265188 | 20 |
| PPP1R14D | -0.259123 | 0.47203 | 0.5830369 | 0.3628594 | 20 |
| SPINT1 | -0.430189 | 0.124345 | 0.000540884 | 0.0412414 | 20 |
| TYRO3 | 0.0180281 | 0.0476762 | 0.7053297 | 0.9384002 | 20 |
| CAPN3 | -0.182871 | 1.27247 | 0.8857267 | 0.2489749 | 12 |
| CKMT1B | 0.429613 | 0.38853 | 0.2688397 | 0.01989399 | 20 |
| CKMT1A | 0.429613 | 0.38853 | 0.2688397 | 0.01989399 | 20 |
| PDIA3 | -0.242824 | 0.654141 | 0.7104806 | 0.5824369 | 20 |
| GOLM2 | 0.145461 | 0.706399 | 0.8368545 | 0.8464193 | 20 |
| SORD | -0.365987 | 0.204734 | 0.07383745 | 0.02832952 | 20 |
| CEP152 | 0.264896 | 0.429728 | 0.5376124 | 0.9013833 | 14 |
| USP8 | -0.614426 | 0.529509 | 0.2458989 | 0.01559133 | 20 |
| SCG3 | -0.00525153 | 0.0576247 | 0.9273867 | 0.04417425 | 20 |
| PRTG | -0.0628488 | 0.0667016 | 0.3460706 | 0.4700257 | 20 |
| ANXA2 | 0.0757311 | 0.0663531 | 0.2537309 | 0.1851133 | 20 |
| CA12 | -0.0461412 | 0.103603 | 0.6560559 | 0.09660344 | 20 |
| DAPK2 | 0.0745245 | 0.0758236 | 0.325673 | 0.04465368 | 20 |
| RBPMS2 | 0.636651 | 0.403798 | 0.1148747 | 0.04005241 | 19 |
| CILP | 0.0928431 | 0.0990362 | 0.3485189 | 0.4685768 | 20 |
| IGDCC4 | 0.163636 | 0.179758 | 0.3626575 | 0.6849389 | 20 |
| MEGF11 | 0.214903 | 0.224064 | 0.3375023 | 0.08235767 | 20 |
| MAP2K1 | -1.19961 | 0.673169 | 0.07474387 | 0.3034384 | 19 |
| SMAD3 | -0.221594 | 0.130853 | 0.09036888 | 0.6943631 | 20 |
| ITGA11 | -0.162866 | 0.139395 | 0.2426556 | 0.8318245 | 20 |
| SPESP1 | 0.524616 | 0.459212 | 0.2532765 | 0.6294011 | 5 |
| NEO1 | -0.0377233 | 0.377333 | 0.9203654 | 0.8742176 | 20 |
| CD276 | -0.0208116 | 0.0323743 | 0.520326 | 0.9555266 | 20 |
| ISLR2 | -0.0991341 | 0.129009 | 0.4422329 | 0.8889331 | 20 |
| SEMA7A | 0.244734 | 0.234937 | 0.29755 | 0.2965396 | 20 |
| MPI | -0.120273 | 0.143229 | 0.4010612 | 0.1233503 | 20 |
| PPCDC | 0.0271619 | 0.0699773 | 0.697903 | 0.4581608 | 20 |
| CSPG4 | 0.0991682 | 0.259246 | 0.7020716 | 0.1292779 | 20 |
| DNAJA4 | 0.125146 | 0.304321 | 0.6809043 | 0.6233436 | 20 |
| CTSH | -0.0235889 | 0.0332612 | 0.4781997 | 0.761532 | 20 |
| MESD | 0.709034 | 0.879399 | 0.4200862 | 0.6016896 | 10 |
| IL16 | -0.065355 | 0.0706814 | 0.3551525 | 0.5544974 | 20 |
| NTRK3 | 0.211981 | 0.165928 | 0.2014091 | 0.120375 | 20 |
| ACAN | -0.1954 | 0.327447 | 0.5506826 | 0.4957725 | 20 |
| MFGE8 | 0.124361 | 0.0892455 | 0.1634759 | 0.4606411 | 20 |
| ANPEP | 0.0678779 | 0.102785 | 0.5090066 | 0.3699513 | 12 |
| FURIN | 0.325235 | 0.166287 | 0.05048076 | 0.5174575 | 20 |
| FES | -0.243053 | 0.218006 | 0.2648975 | 0.308456 | 20 |
| RGMA | 0.0515896 | 0.12619 | 0.682668 | 0.8488896 | 20 |
| IGF1R | 0.0470101 | 0.281915 | 0.8675643 | 0.9443188 | 20 |
| HBZ | -0.0259931 | 0.0290865 | 0.3715103 | 0.494977 | 20 |
| HBQ1 | -0.162681 | 0.0825838 | 0.04885128 | 0.3522967 | 20 |
| AXIN1 | 0.332007 | 0.602075 | 0.5813336 | 0.5784838 | 20 |
| RAB11FIP3 | 0.0177114 | 0.465485 | 0.9696482 | 0.8109358 | 15 |
| WFIKKN1 | 0.206457 | 0.114675 | 0.07180221 | 0.2059269 | 20 |
| MSLN | 0.0156002 | 0.0496294 | 0.7532668 | 0.9651259 | 20 |
| TPSAB1 | -0.0313779 | 0.0376863 | 0.4050649 | 0.2552405 | 20 |
| TPSD1 | 0.0661836 | 0.0862212 | 0.4427239 | 0.05885194 | 20 |
| JPT2 | -0.0802427 | 0.635268 | 0.899484 | 0.6767256 | 20 |
| NME3 | 0.311031 | 0.217571 | 0.1528427 | 0.1395746 | 20 |
| HAGH | 0.251176 | 0.406124 | 0.5362654 | 0.8225535 | 16 |
| GFER | -0.170489 | 0.373322 | 0.6479015 | 0.9216512 | 20 |
| SLC9A3R2 | -0.220468 | 0.247052 | 0.3721818 | 0.623807 | 20 |
| PKD1 | 0.164287 | 0.111059 | 0.1390677 | 0.2065876 | 4 |
| PRSS27 | 0.130489 | 0.116945 | 0.2645006 | 0.9726486 | 20 |
| PRSS22 | 0.0419273 | 0.123082 | 0.7333708 | 0.2388747 | 20 |
| TNFRSF12A | -0.400166 | 0.436753 | 0.3595466 | 0.1296601 | 20 |
| IL32 | 0.188187 | 0.169435 | 0.266709 | 0.8091862 | 20 |
| VASN | -0.0605532 | 0.0721643 | 0.4014124 | 0.7994028 | 20 |
| HMOX2 | -0.0315402 | 0.277819 | 0.9096121 | 0.3464057 | 20 |
| PPL | 0.212174 | 0.373632 | 0.5701239 | 0.2923983 | 20 |
| NAGPA | -0.252075 | 0.0983395 | 0.01036778 | 0.7642681 | 20 |
| PMM2 | 0.0370372 | 0.0955721 | 0.6983624 | 0.8791381 | 18 |
| CARHSP1 | 0.744303 | 0.378971 | 0.04952889 | 0.9751913 | 20 |
| TNFRSF17 | -0.236884 | 0.207959 | 0.254665 | 0.03095485 | 20 |
| CPPED1 | -0.0455375 | 0.0470815 | 0.3334413 | 0.2740073 | 20 |
| PLA2G10 | 0.429014 | 0.345545 | 0.2144002 | 0.05304012 | 8 |
| NOMO1 | 0.103321 | 0.263121 | 0.6945607 | 0.6123989 | 20 |
| CEP20 | -0.269587 | 0.380725 | 0.4788906 | 0.3533925 | 20 |
| COQ7 | 0.292282 | 0.251353 | 0.2448968 | 0.529081 | 20 |
| GP2 | -0.120198 | 0.123111 | 0.3288958 | 0.6100978 | 20 |
| UMOD | 0.0623376 | 0.0351591 | 0.07622637 | 0.9303064 | 20 |
| CRYM | 0.046185 | 0.0747476 | 0.5366551 | 0.6456807 | 20 |
| OTOA | -0.153309 | 0.499504 | 0.7589025 | 0.2498226 | 20 |
| IL4R | -0.000462864 | 0.0949863 | 0.996112 | 0.2040439 | 20 |
| APOBR | -0.0279145 | 0.0254542 | 0.2727928 | 0.5325835 | 20 |
| IL27 | -0.361928 | 0.406888 | 0.3737325 | 0.3645019 | 20 |
| SULT1A1 | -0.370125 | 0.301097 | 0.218976 | 0.8713596 | 20 |
| ATXN2L | 0.979734 | 0.608042 | 0.107116 | 0.8392521 | 20 |
| LAT | -0.0810392 | 0.681857 | 0.9053936 | 0.2482441 | 14 |
| BOLA2 | 0.167567 | 0.374474 | 0.6545341 | 0.995865 | 7 |
| SEZ6L2 | -0.0372553 | 0.190126 | 0.8446486 | 0.7269421 | 20 |
| BOLA2B | 0.132627 | 0.204213 | 0.5160448 | 0.5965388 | 14 |
| DCTPP1 | 0.530562 | 0.566046 | 0.3485977 | 0.2094518 | 20 |
| ITGAL | 0.077038 | 0.185404 | 0.6777649 | 0.5299019 | 20 |
| CTF1 | 0.834174 | 0.56809 | 0.1420004 | 0.932434 | 20 |
| STX4 | -1.27061 | 0.728255 | 0.08103194 | 0.7667197 | 14 |
| PRSS53 | -0.0271761 | 0.0328579 | 0.4081912 | 0.6603648 | 20 |
| PRSS8 | -0.232003 | 0.264584 | 0.3805627 | 0.8424018 | 20 |
| PYDC1 | -0.0969342 | 0.0957285 | 0.3112534 | 0.2330832 | 20 |
| ITGAM | -0.062273 | 0.177274 | 0.725378 | 0.5247781 | 20 |
| AHSP | -0.424293 | 0.584425 | 0.4678378 | 0.8481951 | 8 |
| LPCAT2 | 0.0424475 | 0.349268 | 0.903269 | 0.4859091 | 20 |
| CES1 | 0.0343021 | 0.0624666 | 0.5829181 | 0.386438 | 20 |
| AMFR | -0.280885 | 0.733388 | 0.7017228 | 0.780415 | 15 |
| ARL2BP | 0.567358 | 0.300139 | 0.05871594 | 0.2790479 | 4 |
| CCL22 | 0.380236 | 0.468474 | 0.4169936 | 0.2215254 | 7 |
| CX3CL1 | 0.0424228 | 0.137469 | 0.7576261 | 0.2453528 | 20 |
| CCL17 | -0.122425 | 0.171105 | 0.4743011 | 0.8160424 | 20 |
| CIAPIN1 | 0.2587 | 0.512989 | 0.6140511 | 0.6646571 | 20 |
| ADGRG1 | 0.370999 | 0.404048 | 0.3585107 | 0.8755446 | 20 |
| CDH5 | 0.0321212 | 0.130427 | 0.8054677 | 0.08103073 | 20 |
| CES2 | -0.125682 | 0.223592 | 0.574046 | 0.4121624 | 20 |
| CES3 | 0.228222 | 0.267054 | 0.3927774 | 0.6152615 | 15 |
| TPPP3 | 0.459765 | 0.366193 | 0.2092878 | 0.9487724 | 20 |
| AGRP | 0.158976 | 0.379494 | 0.6752774 | 0.5431404 | 18 |
| CTRL | -0.595812 | 0.409756 | 0.1459285 | 0.2979414 | 20 |
| LCAT | 0.110902 | 0.266932 | 0.6777969 | 0.570143 | 16 |
| DPEP2 | -0.0790097 | 0.141883 | 0.5776207 | 0.06279864 | 3 |
| PLA2G15 | -0.133588 | 0.126124 | 0.2895158 | 0.02086631 | 20 |
| SMPD3 | 1.19305 | 0.660586 | 0.07091078 | 0.4132162 | 20 |
| CDH3 | 0.151093 | 0.354931 | 0.6703285 | 0.1985424 | 20 |
| CDH1 | 0.763211 | 0.630132 | 0.2258215 | 0.06129136 | 20 |
| WWP2 | 0.0118944 | 0.176426 | 0.9462484 | 0.2382493 | 20 |
| IL34 | -0.0628401 | 0.0428757 | 0.1427477 | 0.3730953 | 20 |
| MTSS2 | 0.188562 | 0.401626 | 0.6387137 | 0.8423005 | 5 |
| CALB2 | -0.162648 | 0.468257 | 0.7283305 | 0.6369861 | 20 |
| IST1 | -0.400434 | 0.274077 | 0.1440071 | 0.03435463 | 20 |
| CTRB1 | 0.0766584 | 0.06256 | 0.2204407 | 0.4633786 | 20 |
| CNTNAP4 | 0.610387 | 0.59962 | 0.3086982 | 0.1527464 | 20 |
| HSBP1 | -0.0774252 | 0.0536934 | 0.149306 | 0.8682593 | 20 |
| WFDC1 | -0.0903429 | 0.0886366 | 0.3080838 | 0.09887839 | 20 |
| MTHFSD | 0.0522873 | 0.0647436 | 0.4193169 | 0.1669115 | 20 |
| CA5A | -0.0131675 | 0.0490565 | 0.7883803 | 0.809522 | 20 |
| IL17C | -0.0146386 | 0.237528 | 0.9508585 | 0.638611 | 20 |
| APRT | 0.294163 | 0.923563 | 0.7500986 | 0.3703178 | 12 |
| CDH15 | -0.0940312 | 0.0869259 | 0.2793684 | 0.7598499 | 20 |
| DPEP1 | -0.0315357 | 0.0361488 | 0.3829984 | 0.6991453 | 20 |
| CHMP1A | 0.161724 | 0.304374 | 0.5951892 | 0.4771459 | 20 |
| DOC2B | -0.815293 | 0.473327 | 0.08498421 | 0.7023837 | 20 |
| SCARF1 | 0.0199983 | 0.0779579 | 0.7975439 | 0.882741 | 20 |
| RILP | -0.681073 | 0.553622 | 0.2186172 | 0.657792 | 6 |
| SERPINF2 | 0.21232 | 0.176834 | 0.2298765 | 0.5491171 | 20 |
| SERPINF1 | -0.113764 | 0.101895 | 0.2642154 | 0.5274586 | 20 |
| SHPK | -0.404372 | 0.399444 | 0.311378 | 0.3201393 | 20 |
| CAMKK1 | 0.0113471 | 0.103307 | 0.9125374 | 0.3835569 | 20 |
| CXCL16 | 0.0744488 | 0.150381 | 0.6205517 | 0.8422104 | 20 |
| VMO1 | -0.0185013 | 0.0381878 | 0.6280431 | 0.6145761 | 20 |
| MINK1 | -0.0490402 | 0.505839 | 0.9227676 | 0.7393038 | 20 |
| GP1BA | 0.0568996 | 0.228162 | 0.8030654 | 0.9085576 | 20 |
| ENO3 | 0.104493 | 0.324633 | 0.7475427 | 0.5857755 | 20 |
| KIF1C | 0.287365 | 0.870882 | 0.7414225 | 0.472944 | 15 |
| RABEP1 | 0.495792 | 0.421173 | 0.2391283 | 0.9170596 | 20 |
| CLEC10A | -0.0249339 | 0.0539754 | 0.6441171 | 0.5060544 | 20 |
| ASGR2 | 0.117111 | 0.101113 | 0.2467762 | 0.5179484 | 20 |
| BAP18 | -0.716723 | 0.375442 | 0.0562609 | 0.9647391 | 20 |
| ASGR1 | -0.125905 | 0.169755 | 0.4582767 | 0.4260785 | 20 |
| PLSCR3 | -0.488992 | 0.285906 | 0.08720599 | 0.04813086 | 13 |
| TNFSF12 | -0.0467334 | 0.0884019 | 0.5970506 | 0.7884972 | 20 |
| TNFSF13 | 0.0822102 | 0.102092 | 0.4206708 | 0.6213987 | 20 |
| SHBG | 0.00880771 | 0.107826 | 0.9348977 | 0.2317384 | 20 |
| SAT2 | -0.034327 | 0.0620523 | 0.5801304 | 0.9144102 | 20 |
| TP53 | -0.217937 | 0.492811 | 0.6583206 | 0.2888856 | 18 |
| STX8 | 0.457427 | 0.340944 | 0.1797099 | 0.1142619 | 20 |
| HS3ST3B1 | -0.0293623 | 0.0933752 | 0.7531757 | 0.943559 | 20 |
| TNFRSF13B | 0.368286 | 0.274462 | 0.1796458 | 0.170209 | 20 |
| SHMT1 | -0.00122063 | 0.0416171 | 0.9766013 | 0.6698789 | 20 |
| MFAP4 | -0.070146 | 0.141923 | 0.6211273 | 0.5640447 | 12 |
| ALDH3A1 | 0.0601932 | 0.0624686 | 0.3352594 | 0.9126091 | 20 |
| LGALS9 | -0.0137427 | 0.0682965 | 0.8405252 | 0.7712845 | 20 |
| NOS2 | -0.366079 | 0.412456 | 0.3747779 | 0.3413137 | 20 |
| SEZ6 | 0.0488141 | 0.461854 | 0.915827 | 0.6950756 | 20 |
| GIT1 | -1.14886 | 0.769074 | 0.1352221 | 0.1313524 | 8 |
| BLMH | 0.0746519 | 0.0516826 | 0.1486184 | 0.3436708 | 20 |
| OMG | 0.712071 | 0.37267 | 0.05603927 | 0.8822844 | 20 |
| CCL2 | 0.437307 | 0.513611 | 0.3945274 | 0.544786 | 20 |
| CCL7 | -0.0399244 | 0.277355 | 0.8855425 | 0.2228511 | 20 |
| CCL11 | 0.196163 | 0.295637 | 0.506994 | 0.2435242 | 20 |
| CCL8 | -0.00991712 | 0.0368505 | 0.7878393 | 0.4787774 | 20 |
| CCL13 | -0.0403175 | 0.137439 | 0.7692567 | 0.4599915 | 20 |
| CCL5 | -0.0351706 | 0.171264 | 0.8372912 | 0.5347866 | 20 |
| CCL16 | -0.0215853 | 0.043107 | 0.6165559 | 0.4836445 | 20 |
| CCL14 | -0.00244085 | 0.0532672 | 0.9634514 | 0.5170324 | 20 |
| CCL15 | -0.0328306 | 0.0297222 | 0.2693413 | 0.7726276 | 20 |
| CCL23 | 0.0874726 | 0.0767889 | 0.2546488 | 0.8514409 | 20 |
| CCL18 | 0.0226376 | 0.0554589 | 0.6831363 | 0.445375 | 20 |
| CCL3 | -0.0130857 | 0.0922148 | 0.8871551 | 0.5168662 | 20 |
| CCL4 | 0.0284401 | 0.0829509 | 0.7317073 | 0.06258589 | 20 |
| PLXDC1 | -0.178302 | 0.16743 | 0.2869047 | 0.8988588 | 15 |
| ERBB2 | -0.105448 | 0.184912 | 0.5685003 | 0.767662 | 20 |
| CSF3 | 0.368551 | 0.389462 | 0.3439915 | 0.175924 | 20 |
| IGFBP4 | 0.0273761 | 0.679095 | 0.9678439 | 0.6523288 | 13 |
| KRT19 | 0.0387986 | 0.202429 | 0.8480047 | 0.415019 | 20 |
| CNP | 0.487205 | 0.354179 | 0.168949 | 0.9576975 | 20 |
| AOC3 | -0.0194502 | 0.0645288 | 0.7630947 | 0.5227081 | 20 |
| AARSD1 | 0.677495 | 0.227976 | 0.0029607 | 0.1464863 | 20 |
| VAT1 | -0.149734 | 0.208308 | 0.4722554 | 0.07613103 | 20 |
| TMEM106A | -0.203044 | 0.282253 | 0.4719149 | 0.0195424 | 20 |
| SOST | -0.682957 | 0.536419 | 0.202955 | 0.1694279 | 20 |
| CD300LG | -0.127617 | 0.0745715 | 0.08701913 | 0.2897363 | 20 |
| PYY | 0.132291 | 0.125445 | 0.2916189 | 0.4803573 | 20 |
| SLC4A1 | 0.0827478 | 0.886224 | 0.9256086 | 0.6890673 | 20 |
| GRN | -0.0396631 | 0.120074 | 0.7411568 | 0.1758064 | 20 |
| GFAP | -0.685357 | 0.412903 | 0.09694459 | 0.8003914 | 11 |
| HEXIM1 | 0.144833 | 0.43674 | 0.7401739 | 0.2595348 | 20 |
| LRRC37A2 | 0.00401744 | 0.0477652 | 0.9329705 | 0.9475993 | 5 |
| CDC27 | -0.0380032 | 0.393267 | 0.9230167 | 0.8121866 | 20 |
| SKAP1 | -0.50494 | 0.53988 | 0.3496427 | 0.5492801 | 20 |
| CALCOCO2 | -0.428207 | 0.509088 | 0.4002778 | 0.318435 | 20 |
| GIP | 1.51897 | 0.690679 | 0.02786056 | 0.4307623 | 20 |
| NXPH3 | 0.269201 | 0.192321 | 0.1615878 | 0.009421192 | 13 |
| COL1A1 | -0.944845 | 0.679837 | 0.1645865 | 0.8212568 | 20 |
| CHAD | -0.145466 | 0.0908496 | 0.1093384 | 0.4170602 | 20 |
| WFIKKN2 | -0.0143766 | 0.0439613 | 0.7436459 | 0.004146194 | 20 |
| TRIM25 | -0.0629333 | 0.322976 | 0.8455069 | 0.2961945 | 20 |
| SCPEP1 | -0.000689651 | 0.10016 | 0.9945062 | 0.2546665 | 20 |
| LPO | -0.14045 | 0.100318 | 0.1615008 | 0.7952327 | 20 |
| MPO | 0.11054 | 0.100348 | 0.2706483 | 0.937014 | 20 |
| RNF43 | -0.34422 | 0.284458 | 0.2262433 | 0.3760169 | 20 |
| CA4 | -0.0118287 | 0.0833954 | 0.8872076 | 0.7087031 | 20 |
| ACE | -0.0874544 | 0.0639067 | 0.1711649 | 0.1960221 | 20 |
| CD79B | 0.215969 | 0.180673 | 0.2319459 | 0.9284887 | 20 |
| ICAM2 | 0.284544 | 0.515299 | 0.5808165 | 0.2263145 | 20 |
| ERN1 | -0.243561 | 0.245895 | 0.3219267 | 0.3613178 | 20 |
| MILR1 | -0.0576198 | 0.0727862 | 0.4285759 | 0.8176035 | 20 |
| APOH | 0.126081 | 0.0992542 | 0.2039821 | 0.8228541 | 15 |
| FAM20A | 0.0368049 | 0.128202 | 0.7740475 | 0.960308 | 20 |
| SDK2 | 0.0268847 | 0.060447 | 0.6564899 | 0.6514754 | 20 |
| GPRC5C | -0.217265 | 0.25268 | 0.3898757 | 0.4042757 | 20 |
| CD300A | -0.188231 | 0.134919 | 0.1629731 | 0.184033 | 20 |
| CD300C | -0.0356608 | 0.0601434 | 0.5532284 | 0.9105154 | 20 |
| CD300E | 0.0317567 | 0.086132 | 0.7123525 | 0.2062378 | 20 |
| CD300LF | 0.0482449 | 0.0260228 | 0.06374688 | 0.5480129 | 20 |
| SLC9A3R1 | -0.566389 | 0.321886 | 0.07847584 | 0.6217853 | 13 |
| NT5C | 0.0902522 | 0.0645728 | 0.1622087 | 0.7774608 | 20 |
| ACOX1 | 0.0121738 | 0.427704 | 0.9772928 | 0.1505187 | 20 |
| SEPTIN9 | 0.0587849 | 1.31833 | 0.9644338 | 0.2919348 | 18 |
| TK1 | 0.201642 | 0.215617 | 0.3496908 | 0.6690999 | 15 |
| TIMP2 | 0.0495371 | 0.308359 | 0.8723708 | 0.5948209 | 20 |
| LGALS3BP | 0.075787 | 0.317034 | 0.811067 | 0.5232506 | 17 |
| CANT1 | 0.170511 | 0.224639 | 0.4478263 | 0.6337775 | 20 |
| C1QTNF1 | 0.0871887 | 0.175784 | 0.6198952 | 0.6248187 | 20 |
| ENPP7 | 0.0131601 | 0.0309795 | 0.6709824 | 0.8916467 | 20 |
| SGSH | -0.0280279 | 0.0336754 | 0.4052416 | 0.7172083 | 20 |
| NPTX1 | -0.0978097 | 0.0694347 | 0.1589366 | 0.5611117 | 20 |
| CHMP6 | -0.0648378 | 0.163689 | 0.6920281 | 0.4873582 | 20 |
| BAIAP2 | -0.129655 | 0.349613 | 0.7107477 | 0.8682136 | 20 |
| P4HB | 0.205135 | 0.457357 | 0.6537769 | 0.3642009 | 20 |
| ASPSCR1 | -0.0663441 | 0.139207 | 0.633658 | 0.3498524 | 20 |
| DCXR | -0.0581277 | 0.193942 | 0.7643924 | 0.6516424 | 6 |
| CD7 | -0.0294503 | 0.0667022 | 0.6588375 | 0.1380482 | 20 |
| COLEC12 | -0.0559315 | 0.237926 | 0.814147 | 0.5172858 | 20 |
| CLUL1 | -0.0163406 | 0.048753 | 0.7374956 | 0.8547907 | 20 |
| YES1 | -0.307633 | 0.385465 | 0.4248225 | 0.395675 | 20 |
| PTPRM | -0.00143874 | 0.651489 | 0.998238 | 0.2494552 | 16 |
| CDH2 | -0.22489 | 0.335648 | 0.5028466 | 0.3028976 | 20 |
| DSC2 | 0.015901 | 0.136493 | 0.9072587 | 0.8311586 | 20 |
| DSG3 | 0.0480492 | 0.1141 | 0.6736714 | 0.8897806 | 20 |
| DSG2 | -0.0923413 | 0.132399 | 0.485524 | 0.963856 | 20 |
| TTR | 0.142568 | 0.242135 | 0.5559985 | 0.8768497 | 20 |
| MEP1B | -0.0337513 | 0.0267706 | 0.207396 | 0.175707 | 20 |
| PSTPIP2 | -0.195122 | 0.192349 | 0.3103832 | 0.887627 | 20 |
| ELAC1 | 0.0822829 | 0.497647 | 0.8686733 | 0.9781962 | 6 |
| DCC | 0.302509 | 0.134466 | 0.02446793 | 0.07034068 | 20 |
| GRP | 0.0264111 | 0.0716196 | 0.7122993 | 0.8430128 | 20 |
| TNFRSF11A | 0.0252923 | 0.073497 | 0.7307509 | 0.0385331 | 20 |
| BCL2 | -0.58072 | 0.800931 | 0.4684176 | 0.4921591 | 10 |
| VPS4B | 0.390445 | 0.709511 | 0.582112 | 0.2274918 | 8 |
| SERPINB5 | 0.1444 | 0.543266 | 0.7903942 | 0.7337514 | 15 |
| CD226 | -0.182969 | 0.10897 | 0.09313767 | 0.5101021 | 20 |
| CNDP1 | -0.0144348 | 0.101869 | 0.8873179 | 0.6216485 | 20 |
| NFATC1 | 0.0263945 | 0.262263 | 0.9198352 | 0.7453168 | 20 |
| BSG | -0.328567 | 0.209071 | 0.1160533 | 0.8961544 | 20 |
| FSTL3 | -0.0873754 | 0.337925 | 0.7959717 | 0.77544 | 20 |
| PALM | -0.447014 | 0.218456 | 0.04073311 | 0.3062695 | 20 |
| AZU1 | -0.3442 | 0.220031 | 0.1177419 | 0.4072081 | 9 |
| PRTN3 | 0.0260044 | 0.0685161 | 0.7042887 | 0.3094547 | 20 |
| CFD | -0.152532 | 0.210847 | 0.4694198 | 0.533934 | 20 |
| ARHGAP45 | 0.228287 | 0.416139 | 0.5832914 | 0.6920024 | 20 |
| ADAMTSL5 | 0.03055 | 0.0720333 | 0.6714858 | 0.9278746 | 17 |
| LMNB2 | -0.662798 | 0.713555 | 0.3529582 | 0.9615509 | 20 |
| THOP1 | -0.0444135 | 0.109874 | 0.6860499 | 0.2626409 | 20 |
| GIPC3 | 0.0054076 | 0.118796 | 0.9636927 | 0.9125414 | 20 |
| EBI3 | -0.072605 | 0.0749367 | 0.3326033 | 0.486828 | 20 |
| YJU2 | 0.937064 | 0.555412 | 0.09157448 | 0.913105 | 20 |
| HDGFL2 | 0.144924 | 0.645214 | 0.8222792 | 0.7129358 | 20 |
| LRG1 | 0.0569289 | 0.0519149 | 0.2728243 | 0.3033479 | 19 |
| MYDGF | -0.444066 | 0.43092 | 0.3027716 | 0.04479946 | 16 |
| PLIN3 | -0.0954399 | 0.396632 | 0.809845 | 0.02264535 | 20 |
| PTPRS | 0.0473698 | 0.0768246 | 0.5375008 | 0.6803676 | 20 |
| LONP1 | 0.0580287 | 0.164724 | 0.7246292 | 0.971814 | 20 |
| NRTN | -0.119776 | 0.502051 | 0.8114363 | 0.765791 | 14 |
| FUT3 | -0.016659 | 0.0431772 | 0.699623 | 0.8816889 | 20 |
| FUT5 | -0.016659 | 0.0431772 | 0.699623 | 0.8816889 | 20 |
| CAPS | -0.208009 | 0.266112 | 0.4344158 | 0.3310976 | 20 |
| CD70 | -0.0216006 | 0.0384621 | 0.5743834 | 0.1092475 | 20 |
| TNFSF14 | 0.0529125 | 0.109793 | 0.6298549 | 0.9848262 | 20 |
| C3 | -0.0674371 | 0.222308 | 0.761623 | 0.2689111 | 20 |
| ADGRE1 | 0.0417737 | 0.0931687 | 0.6538889 | 0.6872896 | 20 |
| RETN | -0.0286962 | 0.168889 | 0.8650797 | 0.1363789 | 3 |
| MCEMP1 | -0.0756824 | 0.120121 | 0.5286625 | 0.1951369 | 8 |
| FCER2 | -0.0139246 | 0.0983845 | 0.887449 | 0.8701903 | 20 |
| CLEC4G | -0.236076 | 0.14654 | 0.1071805 | 0.869067 | 20 |
| CD209 | -0.000867468 | 0.0757302 | 0.9908607 | 0.4210241 | 20 |
| CLEC4M | -0.000984983 | 0.0859893 | 0.9908607 | 0.3531087 | 20 |
| CCL25 | -0.0316983 | 0.0413595 | 0.4434327 | 0.01966941 | 20 |
| ANGPTL4 | -0.0442662 | 0.101921 | 0.6640572 | 0.673258 | 20 |
| MUC16 | 0.0642577 | 0.401146 | 0.8727349 | 0.4485483 | 20 |
| ICAM1 | 0.0280509 | 0.0701651 | 0.6893161 | 0.6763462 | 20 |
| ICAM4 | -0.101598 | 0.254193 | 0.6893861 | 0.5529499 | 20 |
| ICAM5 | -0.00659888 | 0.0399501 | 0.8688037 | 0.5174333 | 20 |
| ICAM3 | 0.0410144 | 0.0963141 | 0.6702245 | 0.4874083 | 20 |
| LDLR | 0.708891 | 1.19256 | 0.5522258 | 0.4582636 | 13 |
| ACP5 | 0.0467511 | 0.0798044 | 0.557996 | 0.8732741 | 20 |
| PRDX2 | -0.387545 | 0.393874 | 0.3251494 | 0.1936671 | 3 |
| RNASEH2A | 0.437468 | 0.302969 | 0.1487564 | 0.06787138 | 20 |
| RTBDN | 0.0269927 | 0.175619 | 0.8778459 | 0.7027143 | 20 |
| FARSA | 0.826615 | 0.529106 | 0.1182206 | 0.5980807 | 15 |
| MRI1 | -0.0643736 | 0.0824712 | 0.4350623 | 0.488246 | 19 |
| CC2D1A | 0.445197 | 0.652841 | 0.4952785 | 0.9378211 | 10 |
| ADGRE5 | 0.0399343 | 0.123376 | 0.7461809 | 0.137983 | 10 |
| DNAJB1 | 1.67351 | 0.614592 | 0.00646989 | 0.1426839 | 20 |
| ADGRE2 | 0.0746526 | 0.118795 | 0.5297317 | 0.905897 | 20 |
| NOTCH3 | 0.256785 | 0.463636 | 0.5796806 | 0.1158965 | 20 |
| PGLYRP2 | -0.0181704 | 0.0305766 | 0.5523381 | 0.2446562 | 20 |
| MYO9B | 0.123138 | 0.563232 | 0.82694 | 0.4358704 | 20 |
| BST2 | -0.175544 | 0.140084 | 0.2101568 | 0.4231256 | 20 |
| IL12RB1 | -0.0358544 | 0.0762155 | 0.638045 | 0.09744801 | 20 |
| IFI30 | 0.00648249 | 0.0664659 | 0.9223047 | 0.3583397 | 20 |
| GDF15 | 0.285959 | 0.136882 | 0.03669982 | 0.3805589 | 20 |
| LRRC25 | -0.0136061 | 0.085175 | 0.873084 | 0.744252 | 20 |
| COMP | 0.124997 | 0.156456 | 0.4243312 | 0.8272754 | 20 |
| NCAN | -0.0775075 | 0.217269 | 0.7212897 | 0.8288189 | 20 |
| SUGP1 | -0.31121 | 0.146201 | 0.03328391 | 0.2136209 | 20 |
| C19orf12 | -0.438718 | 0.495614 | 0.376048 | 0.8261252 | 20 |
| PDCD5 | 0.0831648 | 0.0875421 | 0.3421132 | 0.5274563 | 20 |
| PEPD | 0.0951155 | 0.0840219 | 0.2576211 | 0.1652617 | 20 |
| FXYD5 | 0.0724374 | 0.0850914 | 0.3946088 | 0.6251113 | 20 |
| CD22 | 0.0127424 | 0.112003 | 0.9094216 | 0.7429909 | 20 |
| SBSN | 0.0223139 | 0.0565017 | 0.6928981 | 0.7806774 | 20 |
| HSPB6 | 0.296719 | 0.728575 | 0.6838172 | 0.9016092 | 12 |
| NPHS1 | -0.00440767 | 0.0641534 | 0.9452244 | 0.3191751 | 20 |
| KIRREL2 | 0.044963 | 0.157627 | 0.7754528 | 0.8708328 | 20 |
| TBCB | 0.171388 | 0.637117 | 0.787925 | 0.9012304 | 9 |
| SPINT2 | 0.427848 | 0.172688 | 0.01322747 | 0.02261425 | 20 |
| PPP1R14A | 0.319597 | 0.130517 | 0.0143372 | 0.002817175 | 20 |
| LGALS7 | 0.121516 | 0.126499 | 0.3367476 | 0.5462 | 20 |
| LGALS7B | 0.121516 | 0.126499 | 0.3367476 | 0.5462 | 20 |
| LGALS4 | 0.194867 | 0.332068 | 0.5573176 | 0.4468756 | 20 |
| SIRT2 | -0.0934432 | 0.206458 | 0.6508357 | 0.9305382 | 12 |
| CCER2 | 0.0711654 | 0.0977535 | 0.4666083 | 0.5016323 | 16 |
| IFNL1 | -0.473466 | 0.671164 | 0.4805361 | 0.1578631 | 20 |
| GMFG | 0.306532 | 0.438959 | 0.4849799 | 0.08063542 | 20 |
| CLC | 0.816486 | 0.551905 | 0.1390342 | 0.9830567 | 5 |
| BLVRB | -0.169627 | 0.0940099 | 0.07117631 | 0.69118 | 6 |
| MIA | 0.0347953 | 0.0311022 | 0.2632504 | 0.4170232 | 20 |
| AXL | 0.0996371 | 0.169672 | 0.5570478 | 0.241649 | 20 |
| TGFB1 | 0.0400658 | 0.120283 | 0.739061 | 0.4562628 | 20 |
| CEACAM21 | -0.0203777 | 0.02565 | 0.4269313 | 0.6194286 | 20 |
| CEACAM5 | 0.102321 | 0.101148 | 0.3117274 | 0.05009449 | 18 |
| CEACAM6 | 0.545815 | 0.262665 | 0.03771065 | 0.3979389 | 20 |
| CXCL17 | 0.104422 | 0.456057 | 0.8188941 | 0.9171856 | 20 |
| CEACAM1 | -0.015652 | 0.111819 | 0.8886789 | 0.6395202 | 20 |
| CEACAM8 | -0.309712 | 0.598828 | 0.6050187 | 0.855031 | 20 |
| PSG1 | 0.0171992 | 0.0399642 | 0.6669295 | 0.6601807 | 20 |
| CD177 | 0.0322485 | 0.0583759 | 0.5806553 | 0.8877998 | 20 |
| TEX101 | 0.12489 | 0.13042 | 0.3382638 | 0.584301 | 20 |
| LYPD3 | 0.0414301 | 0.103028 | 0.6875911 | 0.6879755 | 20 |
| PINLYP | 0.0328977 | 0.0373697 | 0.3786798 | 0.2635661 | 20 |
| PLAUR | -0.200259 | 0.25828 | 0.4381281 | 0.1202771 | 19 |
| CEACAM20 | 0.201006 | 0.252818 | 0.4265781 | 0.725777 | 20 |
| PVR | -0.0206582 | 0.0436291 | 0.6358592 | 0.1454706 | 20 |
| CEACAM19 | 0.043882 | 0.227883 | 0.8473005 | 0.773658 | 20 |
| CEACAM16 | -0.0301937 | 0.0867179 | 0.7277026 | 0.4924596 | 20 |
| BCAM | 0.20559 | 0.178466 | 0.2493282 | 0.80154 | 20 |
| NECTIN2 | -0.114326 | 0.157368 | 0.4675379 | 0.4976115 | 20 |
| APOE | 0.0197028 | 0.0370648 | 0.5950191 | 0.09088129 | 20 |
| APOC1 | 0.128523 | 0.0851233 | 0.1310817 | 0.1768693 | 20 |
| PGLYRP1 | -0.00588234 | 0.114819 | 0.9591409 | 0.8024288 | 20 |
| PRKD2 | 0.0859314 | 0.732512 | 0.9066139 | 0.4737827 | 20 |
| SULT2A1 | 0.0810365 | 0.106569 | 0.4470079 | 0.1207756 | 20 |
| CA11 | -0.133973 | 0.400804 | 0.7381821 | 0.03404109 | 20 |
| FGF21 | 0.364344 | 0.235841 | 0.1223775 | 0.5742522 | 20 |
| HSD17B14 | 0.0849663 | 0.0463317 | 0.06667309 | 0.3026847 | 20 |
| LHB | 0.20932 | 0.417637 | 0.6162297 | 0.3680551 | 20 |
| CGB3 | -1.05901 | 0.483527 | 0.02851095 | 0.5265469 | 20 |
| CGB5 | -1.05901 | 0.483527 | 0.02851095 | 0.5265469 | 20 |
| CGB8 | -1.05901 | 0.483527 | 0.02851095 | 0.5265469 | 20 |
| HRC | 0.30194 | 0.287854 | 0.2942081 | 0.3733064 | 20 |
| DKKL1 | 0.00470094 | 0.0300302 | 0.8756069 | 0.4685699 | 20 |
| FLT3LG | -0.354924 | 0.369256 | 0.3364579 | 0.4302625 | 5 |
| TBC1D17 | 0.00285158 | 0.0708529 | 0.9678967 | 0.149114 | 20 |
| MYBPC2 | -0.647763 | 0.757639 | 0.3925646 | 0.1532375 | 11 |
| CLEC11A | 0.00343896 | 0.106156 | 0.9741569 | 0.1108705 | 10 |
| KLK1 | -0.0735557 | 0.152117 | 0.628708 | 0.5429477 | 20 |
| KLK15 | 0.00315868 | 0.0376401 | 0.9331218 | 0.6033652 | 20 |
| KLK3 | 1.25884 | 0.549795 | 0.02204097 | 0.5330369 | 20 |
| KLK4 | -0.0603979 | 0.0718676 | 0.4006816 | 0.4558973 | 20 |
| KLK6 | 0.0379681 | 0.154783 | 0.8062253 | 0.4827005 | 20 |
| KLK7 | -0.0344609 | 0.0666035 | 0.6048749 | 0.3437953 | 13 |
| KLK8 | -0.092029 | 0.109508 | 0.4006922 | 0.1044184 | 12 |
| KLK10 | 0.0469686 | 0.0485235 | 0.3330665 | 0.3292729 | 20 |
| KLK11 | 0.0643847 | 0.0747094 | 0.388797 | 0.6571399 | 12 |
| KLK12 | -0.033919 | 0.0244671 | 0.1656523 | 0.04661802 | 20 |
| KLK13 | -0.149428 | 0.0655479 | 0.02262658 | 0.4086964 | 20 |
| KLK14 | -0.137999 | 0.0809464 | 0.0882278 | 0.1019965 | 20 |
| SIGLEC9 | 0.00833283 | 0.0509815 | 0.8701656 | 0.8179474 | 20 |
| SIGLEC7 | 0.0307197 | 0.0902993 | 0.7337073 | 0.3024222 | 15 |
| CD33 | 0.0147167 | 0.0248658 | 0.5539563 | 0.3543511 | 20 |
| VSIG10L | 0.261439 | 0.138192 | 0.0585111 | 0.937974 | 20 |
| SIGLEC10 | -0.113216 | 0.0723603 | 0.1176728 | 0.07241276 | 11 |
| SIGLEC8 | -0.0650125 | 0.059981 | 0.2784159 | 0.8323005 | 20 |
| SIGLEC6 | 0.0346057 | 0.0903893 | 0.7018295 | 0.4093381 | 6 |
| SIGLEC5 | -0.00431692 | 0.0315721 | 0.8912425 | 0.2292441 | 20 |
| VSTM1 | 0.00454874 | 0.0370473 | 0.9022797 | 0.2266005 | 20 |
| OSCAR | -0.0477988 | 0.0548341 | 0.3833731 | 0.2296755 | 20 |
| LILRA6 | -0.0314864 | 0.0363879 | 0.3868751 | 0.2903797 | 19 |
| LILRB5 | -0.0239151 | 0.0249229 | 0.3372745 | 0.3864804 | 20 |
| LILRB2 | 0.0110781 | 0.0274283 | 0.6862921 | 0.4965707 | 20 |
| LILRA3 | 0.0114346 | 0.0254182 | 0.6528107 | 0.5710061 | 20 |
| LILRA5 | 0.0529921 | 0.0802929 | 0.5092636 | 0.6012389 | 20 |
| LAIR1 | -0.00294085 | 0.0568589 | 0.9587502 | 0.5109453 | 20 |
| LAIR2 | 0.0269578 | 0.0471674 | 0.5676367 | 0.275358 | 20 |
| KIR2DL2 | -0.436155 | 0.185337 | 0.0186073 | 0.719746 | 20 |
| LILRA2 | 0.00394618 | 0.0440169 | 0.9285641 | 0.02951248 | 20 |
| LILRB1 | 0.0207718 | 0.0461744 | 0.6528144 | 0.5710552 | 20 |
| LILRB4 | -0.106463 | 0.142774 | 0.455863 | 0.03939888 | 19 |
| KIR3DL1 | -0.0195208 | 0.0476401 | 0.6819853 | 0.03205149 | 18 |
| KIR2DL3 | -0.284334 | 0.169611 | 0.09366297 | 0.1068995 | 20 |
| KIR2DS4 | -0.0348559 | 0.0280848 | 0.2145705 | 0.1118788 | 20 |
| KIR3DL2 | 0.0804184 | 0.238458 | 0.7359335 | 0.6661179 | 20 |
| FCAR | 0.0196996 | 0.0371452 | 0.5958767 | 0.08019883 | 20 |
| NCR1 | -0.186339 | 0.11369 | 0.1012121 | 0.4537683 | 20 |
| GP6 | 0.0441509 | 0.105578 | 0.6758128 | 0.7370938 | 20 |
| PTPRH | -0.0605927 | 0.0478113 | 0.2050377 | 0.2886727 | 20 |
| SSC5D | -0.101369 | 0.060951 | 0.0962864 | 0.2509728 | 20 |
| A1BG | 0.160416 | 0.323548 | 0.6200331 | 0.06385929 | 20 |
| NSFL1C | 0.136058 | 0.381378 | 0.7212761 | 0.5071543 | 20 |
| SIRPB1 | -0.0487445 | 0.0376799 | 0.1957881 | 0.5125409 | 20 |
| SIRPA | 0.00729117 | 0.024004 | 0.76132 | 0.9555016 | 20 |
| CPXM1 | 0.0262114 | 0.0904462 | 0.7719687 | 0.3488322 | 20 |
| OXT | 0.0410436 | 0.0498609 | 0.4104158 | 0.01899704 | 20 |
| ITPA | 0.0097834 | 0.050266 | 0.8456805 | 0.6679405 | 20 |
| ATRN | 0.0907016 | 0.0423419 | 0.03218344 | 0.5987292 | 20 |
| SIGLEC1 | 0.168882 | 0.148773 | 0.2563065 | 0.1222195 | 4 |
| MAVS | 0.146132 | 0.333091 | 0.6608687 | 0.8982755 | 20 |
| PRND | -0.00487763 | 0.103732 | 0.962496 | 0.3363448 | 20 |
| RASSF2 | 0.170022 | 0.31916 | 0.5942288 | 0.4395233 | 20 |
| CHGB | -0.059189 | 0.0469957 | 0.2078657 | 0.8965478 | 20 |
| SNAP25 | 0.154932 | 0.287772 | 0.5903116 | 0.894979 | 20 |
| ISM1 | 0.074585 | 0.168631 | 0.6582739 | 0.5023347 | 20 |
| DTD1 | 0.0633143 | 0.181849 | 0.7277124 | 0.7274278 | 20 |
| THBD | -0.055705 | 0.158593 | 0.725405 | 0.9490761 | 20 |
| CST3 | -0.123331 | 0.104045 | 0.2358743 | 0.2326674 | 20 |
| CST1 | -0.0470383 | 0.0622406 | 0.4497989 | 0.7327128 | 20 |
| CST5 | -0.0581686 | 0.039095 | 0.1367832 | 0.007690384 | 20 |
| CST7 | -0.0236983 | 0.0564017 | 0.6743608 | 0.5138916 | 20 |
| ENTPD6 | -0.0103366 | 0.0514606 | 0.8408043 | 0.3642425 | 20 |
| BPIFB2 | 0.135895 | 0.115252 | 0.2383533 | 0.9548772 | 20 |
| BPIFA2 | 0.234998 | 0.206705 | 0.25559 | 0.9120119 | 20 |
| BPIFB1 | -0.0704054 | 0.0681428 | 0.3015084 | 0.2186995 | 20 |
| RALY | -1.27251 | 0.442262 | 0.004011213 | 0.8197317 | 20 |
| AHCY | 0.02235 | 0.107426 | 0.8351898 | 0.4343144 | 9 |
| PROCR | 0.039377 | 0.0575701 | 0.493985 | 0.5478147 | 20 |
| SLA2 | -0.502838 | 0.532348 | 0.3448801 | 0.3840168 | 9 |
| VSTM2L | 0.158488 | 0.268723 | 0.5553368 | 0.1243337 | 20 |
| TGM2 | -0.0232869 | 0.170083 | 0.8910981 | 0.9769974 | 20 |
| LBP | -0.00597286 | 0.034832 | 0.8638493 | 0.818307 | 20 |
| ADA | -0.134449 | 0.0534313 | 0.0118596 | 0.9372214 | 20 |
| CCN5 | -0.116069 | 0.143129 | 0.417401 | 0.5830326 | 20 |
| STK4 | -0.399961 | 0.544686 | 0.462768 | 0.8033256 | 13 |
| WFDC12 | 0.166793 | 0.0671326 | 0.01297231 | 0.2525888 | 20 |
| PI3 | -0.100977 | 0.102002 | 0.3221945 | 0.1476975 | 20 |
| SDC4 | 0.190841 | 0.128117 | 0.1363335 | 0.3516024 | 18 |
| WFDC2 | 0.564698 | 0.324611 | 0.08192613 | 0.6152454 | 20 |
| SPINT3 | -0.226202 | 0.315088 | 0.472818 | 0.1477714 | 20 |
| PLTP | 0.0960711 | 0.0740838 | 0.1947034 | 0.2399432 | 20 |
| MMP9 | 0.131256 | 0.199615 | 0.5108311 | 0.1909467 | 20 |
| CD40 | -0.0425213 | 0.0755569 | 0.5735904 | 0.9301436 | 20 |
| CEBPB | -0.356052 | 0.579192 | 0.5387265 | 0.8608396 | 20 |
| CBLN4 | 0.142213 | 0.183127 | 0.4374044 | 0.8044982 | 12 |
| ZBP1 | 0.104027 | 0.119841 | 0.3853713 | 0.823009 | 9 |
| STX16 | 0.456491 | 0.475842 | 0.337391 | 0.9216759 | 20 |
| GNAS | -0.276543 | 0.385633 | 0.4733037 | 0.9521077 | 20 |
| CTSZ | -0.0239647 | 0.094077 | 0.798928 | 0.8482356 | 20 |
| OGFR | 0.309529 | 0.373788 | 0.4076216 | 0.1731702 | 20 |
| TNFRSF6B | -0.335634 | 0.105502 | 0.001466093 | 0.240865 | 20 |
| CXADR | -0.287138 | 0.247764 | 0.2464886 | 0.5250876 | 20 |
| TMPRSS15 | -0.0421774 | 0.292625 | 0.885394 | 0.6047888 | 20 |
| NCAM2 | 0.0114242 | 0.05766 | 0.8429429 | 0.9455777 | 20 |
| JAM2 | -0.727712 | 0.409184 | 0.07533029 | 0.07623873 | 20 |
| APP | 0.437334 | 0.419981 | 0.297728 | 0.6845899 | 20 |
| ADAMTS1 | -0.150352 | 0.790242 | 0.8491049 | 0.2897563 | 5 |
| BACH1 | 0.201718 | 0.695278 | 0.7717203 | 0.9450791 | 20 |
| SOD1 | 0.438056 | 0.391468 | 0.2631373 | 0.950994 | 20 |
| IL10RB | 0.0109253 | 0.053494 | 0.8381711 | 0.8298751 | 20 |
| IFNAR1 | -0.00803857 | 0.0696804 | 0.908157 | 0.867165 | 20 |
| IFNGR2 | -0.0175171 | 0.0255306 | 0.4926365 | 0.6365467 | 20 |
| GART | -0.221779 | 0.28567 | 0.4375452 | 0.5594498 | 20 |
| CRYZL1 | -0.349869 | 0.272945 | 0.1999022 | 0.4890881 | 20 |
| DSCAM | -0.00223395 | 0.199684 | 0.9910739 | 0.3443934 | 20 |
| FAM3B | 0.0269759 | 0.0616431 | 0.6616661 | 0.4814455 | 20 |
| TFF3 | 0.176638 | 0.239081 | 0.4600173 | 0.1286688 | 19 |
| TFF2 | -0.0306352 | 0.241702 | 0.89914 | 0.3777216 | 20 |
| TFF1 | 0.198589 | 0.120687 | 0.09986979 | 0.9893978 | 20 |
| CBS | 0.318634 | 0.459447 | 0.4879852 | 0.5165421 | 20 |
| CSTB | 0.037203 | 0.0372775 | 0.3182788 | 0.5776784 | 20 |
| GATD3 | 0.0385174 | 0.330593 | 0.9072483 | 0.08176473 | 20 |
| ICOSLG | -0.12487 | 0.141346 | 0.3769986 | 0.9631891 | 20 |
| ITGB2 | -0.13639 | 0.237238 | 0.5653529 | 0.3558218 | 20 |
| COL18A1 | 0.0751011 | 0.180223 | 0.6768892 | 0.8340566 | 20 |
| FTCD | 0.0223578 | 0.3005 | 0.9406905 | 0.8152678 | 10 |
| IL17RA | 0.0247511 | 0.0335158 | 0.4602166 | 0.241406 | 20 |
| ADA2 | -0.201451 | 0.143084 | 0.1591537 | 0.6640723 | 16 |
| BID | -0.392473 | 0.299119 | 0.1894874 | 0.6881582 | 6 |
| DGCR6 | 0.131153 | 0.597682 | 0.8263106 | 0.1367803 | 20 |
| GP1BB | 0.709939 | 0.590349 | 0.2291407 | 0.2773872 | 19 |
| COMT | 0.0203038 | 0.0642169 | 0.7518697 | 0.7293569 | 20 |
| RANBP1 | 0.459498 | 0.503076 | 0.3610446 | 0.1139571 | 20 |
| RTN4R | 0.111866 | 0.112676 | 0.3208042 | 0.07247563 | 20 |
| SCARF2 | -0.130636 | 0.098586 | 0.1851384 | 0.4847894 | 20 |
| SNAP29 | 0.131651 | 0.452438 | 0.7710653 | 0.2965636 | 19 |
| PPM1F | 0.219133 | 0.494224 | 0.657485 | 0.5237438 | 20 |
| IGLC2 | -0.0374443 | 0.16283 | 0.8181236 | 0.59695 | 20 |
| BCR | -1.10207 | 0.417135 | 0.00824181 | 0.4422391 | 10 |
| CHCHD10 | -0.489623 | 0.309201 | 0.1133053 | 0.6716735 | 20 |
| MIF | -0.211263 | 0.271262 | 0.4360887 | 0.5758651 | 20 |
| GSTT2B | -0.0190742 | 0.0285359 | 0.5038607 | 0.6913865 | 20 |
| DDT | -0.109088 | 0.152545 | 0.4745336 | 0.766599 | 20 |
| SUSD2 | -0.0732903 | 0.0447942 | 0.1018068 | 0.8516865 | 20 |
| GGT5 | -0.0387342 | 0.101017 | 0.7013923 | 0.8779531 | 20 |
| UPB1 | -0.645854 | 0.533742 | 0.2262601 | 0.9532843 | 3 |
| GGT1 | -0.00402262 | 0.119384 | 0.9731207 | 0.7717333 | 20 |
| SEZ6L | -0.0770481 | 0.135279 | 0.5689834 | 0.01321457 | 8 |
| CRYBB1 | -0.00129642 | 0.184288 | 0.9943871 | 0.04042512 | 20 |
| LIF | 0.234296 | 0.619919 | 0.7054708 | 0.3137441 | 20 |
| OSM | -0.495709 | 0.418957 | 0.2367302 | 0.3585954 | 18 |
| TCN2 | 0.0843973 | 0.057819 | 0.1443779 | 0.2310604 | 20 |
| PIK3IP1 | 0.00349988 | 0.102544 | 0.972773 | 0.4445149 | 20 |
| TIMP3 | -0.0274363 | 0.0494496 | 0.5790075 | 0.6451385 | 20 |
| HMOX1 | -0.125878 | 0.43296 | 0.7712516 | 0.602014 | 20 |
| MB | 0.0548544 | 0.762731 | 0.9426668 | 0.9494707 | 10 |
| APOL1 | 0.0917036 | 0.123811 | 0.4588916 | 0.5953551 | 19 |
| PVALB | 0.00874879 | 0.0428254 | 0.8381269 | 0.9650532 | 20 |
| CSF2RB | -0.047313 | 0.0259869 | 0.06866081 | 0.7764361 | 20 |
| TST | 0.0659112 | 0.322675 | 0.8381465 | 0.9286103 | 20 |
| C1QTNF6 | -0.757833 | 0.510988 | 0.1380547 | 0.8938456 | 15 |
| SH3BP1 | 0.0668264 | 0.144882 | 0.6446211 | 0.2763439 | 20 |
| LGALS1 | -0.0927155 | 0.108883 | 0.3944817 | 0.14557 | 20 |
| ANKRD54 | -0.634665 | 0.41684 | 0.1278677 | 0.7326429 | 20 |
| NPTXR | -0.0100638 | 0.0341471 | 0.7682086 | 0.6266222 | 20 |
| GRAP2 | 0.169564 | 0.327734 | 0.6048887 | 0.9823675 | 9 |
| ST13 | 0.195099 | 0.537831 | 0.7167907 | 0.1133705 | 20 |
| TEF | -0.539952 | 0.401412 | 0.1785821 | 0.6107814 | 20 |
| CCDC134 | -0.465362 | 0.508148 | 0.3597724 | 0.9479396 | 20 |
| TNFRSF13C | -0.0359805 | 0.0632296 | 0.5693257 | 0.08375686 | 20 |
| NAGA | 0.520304 | 0.324199 | 0.108518 | 0.2742008 | 10 |
| ATXN10 | 0.235142 | 0.306169 | 0.4424788 | 0.2099796 | 20 |
| TAFA5 | -0.261484 | 0.168436 | 0.1205604 | 0.1577518 | 20 |
| CRELD2 | 0.00284347 | 0.112769 | 0.9798834 | 0.8883299 | 20 |
| PLXNB2 | 0.0272546 | 0.0402535 | 0.4983596 | 0.9799176 | 20 |
| TYMP | 0.0861884 | 0.163942 | 0.5990802 | 0.9210842 | 20 |
| ARSA | -0.0547091 | 0.079075 | 0.4890228 | 0.1771999 | 20 |
